# Experimental and computational studies of the mechanism of iron-catalysed C–H activation/functionalisation with allyl electrophiles†

**DOI:** 10.1039/d1sc01661j

**Published:** 2021-06-17

**Authors:** Joshua C. DeMuth, Zhihui Song, Stephanie H. Carpenter, Theresa E. Boddie, Aleksa Radović, Tessa M. Baker, Osvaldo Gutierrez, Michael L. Neidig

**Affiliations:** Department of Chemistry, University of Rochester Rochester New York 14627 USA neidig@chem.rochester.edu; Department of Chemistry and Biochemistry, University of Maryland College Park Maryland 20742 USA ogs@umd.edu

## Abstract

Synthetic methods that utilise iron to facilitate C–H bond activation to yield new C–C and C–heteroatom bonds continue to attract significant interest. However, the development of these systems is still hampered by a limited molecular-level understanding of the key iron intermediates and reaction pathways that enable selective product formation. While recent studies have established the mechanism for iron-catalysed C–H arylation from aryl-nucleophiles, the underlying mechanistic pathway of iron-catalysed C–H activation/functionalisation systems which utilise electrophiles to establish C–C and C–heteroatom bonds has not been determined. The present study focuses on an iron-catalysed C–H allylation system, which utilises allyl chlorides as electrophiles to establish a C–allyl bond. Freeze-trapped inorganic spectroscopic methods (^57^Fe Mössbauer, EPR, and MCD) are combined with correlated reaction studies and kinetic analyses to reveal a unique and rapid reaction pathway by which the allyl electrophile reacts with a C–H activated iron intermediate. Supporting computational analysis defines this novel reaction coordinate as an inner-sphere radical process which features a partial iron–bisphosphine dissociation. Highlighting the role of the bisphosphine in this reaction pathway, a complementary study performed on the reaction of allyl electrophile with an analogous C–H activated intermediate bearing a more rigid bisphosphine ligand exhibits stifled yield and selectivity towards allylated product. An additional spectroscopic analysis of an iron-catalysed C–H amination system, which incorporates *N*-chloromorpholine as the C–N bond-forming electrophile, reveals a rapid reaction of electrophile with an analogous C–H activated iron intermediate consistent with the inner-sphere radical process defined for the C–H allylation system, demonstrating the prevalence of this novel reaction coordinate in this sub-class of iron-catalysed C–H functionalisation systems. Overall, these results provide a critical mechanistic foundation for the rational design and development of improved systems that are efficient, selective, and useful across a broad range of C–H functionalisations.

## Abstract 2

Experimental and computational studies support an inner-sphere radical pathway for iron-catalysed C–H activation/functionalisation with allyl electrophiles.

## Graphical abstract

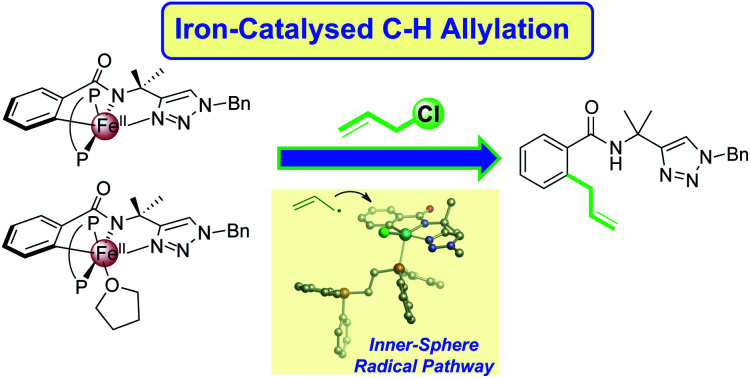

## Introduction

The synthesis of new molecules through direct C–H bond functionalisation represents an attractive and atom-economical approach for the introduction of molecular complexity. Despite this, the selective functionalisation of C–H bonds has presented a challenge for synthetic design as these bonds are highly unreactive.^1^ Efforts to solve this reactivity issue have included significant research towards the utilisation of precious metal catalysts which facilitate C–H bond activation and establish new C–C, C–O, C–N, and C–B bonds.^2–5^

**Scheme 1:**
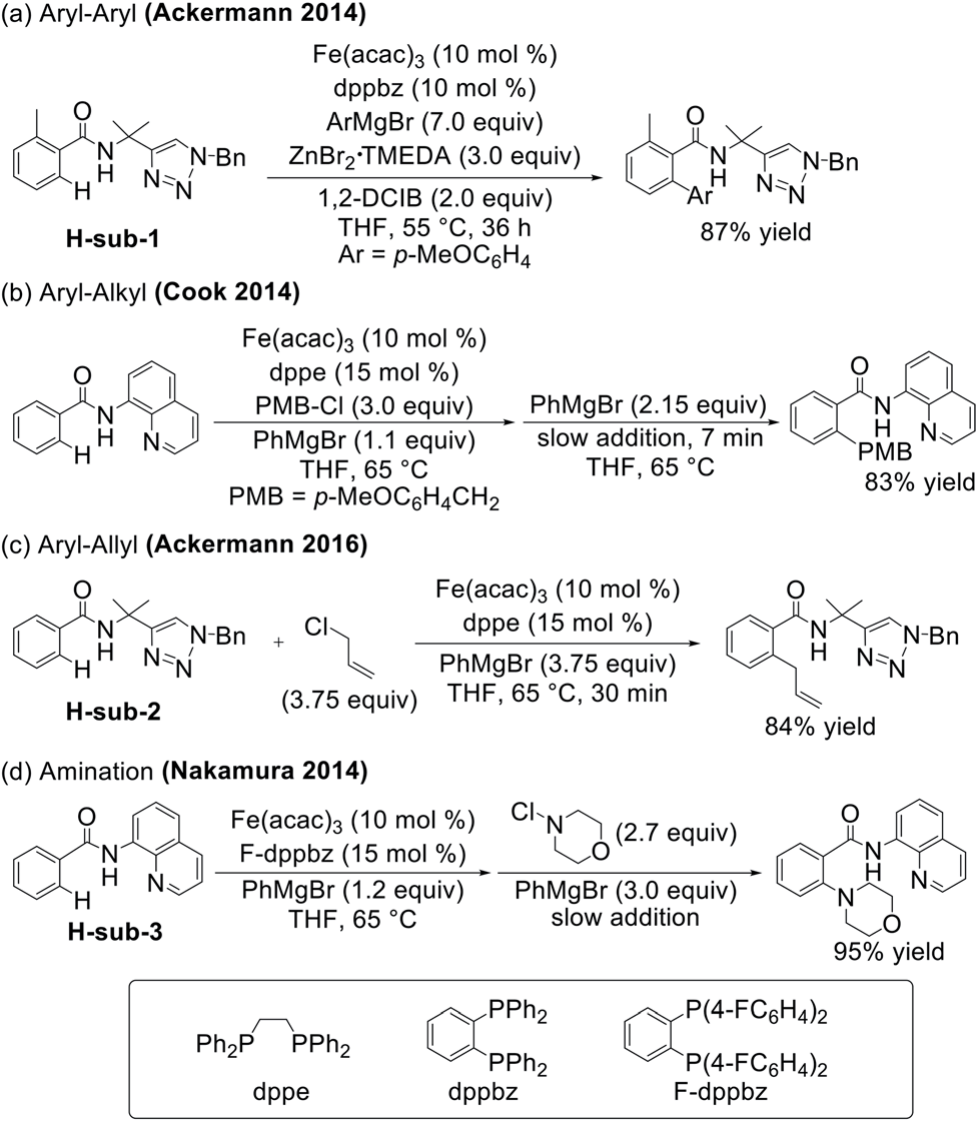
Examples of iron-catalysed C–H activation/functionalisation reactions.

Recent efforts in C–H functionalisation methodologies have focused more heavily on the use of base metal catalysts in order to develop systems which utilise metals that have high natural abundance and low toxicity relative to their precious metal counterparts.^6–8^ Of particular note are iron-catalysed systems for C–H activation/functionalisation which have rapidly expanded over the last decade to introduce a broad range of functionalities from C–C bond-forming arylation,^9–15^ alkenylation,^12,16^ and alkylation^17–21^ to C–heteroatom forming amination,^22,23^ borylation,^24,25^ and halogenation^26,27^ among many others^28–34^ (Scheme 1). Central to many iron-catalysed C–H functionalisations, the selectivity and yields of these systems are reliant upon the incorporation of directing groups, with triazole and quinoline groups particularly widely used.^18,22,35–37^

**Scheme 2:**
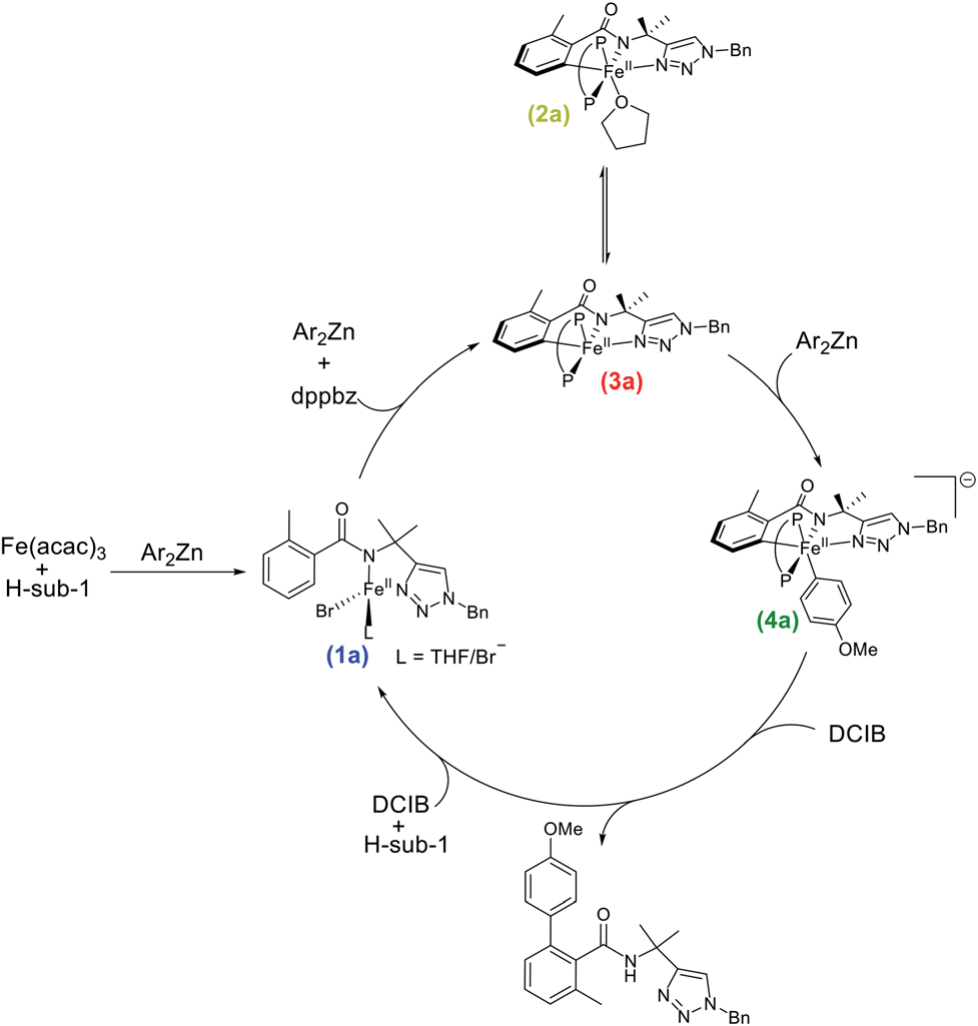
Experimentally determined reaction mechanism for iron-catalysed, triazole-directed C–H arylation.^38^ (Ar = *p*-MeOC_6_H_4_) (P–P = dppbz).

Despite the impressive developments in selective iron-catalysed C–H activation/functionalisation methods utilising directing groups, a molecular-level understanding of the underlying reaction mechanisms and key iron intermediates involved in catalysis remains underdeveloped. In turn, this lack of fundamental insight has hindered the rational design of the next generation of iron catalysts employed for C–H functionalisation. Motivated by this challenge, our group recently reported the first direct spectroscopic and crystallographic studies identifying the key cyclometalated iron intermediates in the iron-catalysed, triazole-directed C–H arylation of benzamides (Scheme 2).^38^ Notable insights from this work included: (1) a cyclometalated and arylated low-spin iron(ii) intermediate (**4a**) is responsible for the key C–C bond-forming step *via* reaction with oxidant (1,2-dichloroisobutane, DCIB) at a rate consistent with catalysis; (2) the rate-determining step is transmetalation to generate **4a** whereas both the C–H activation and reductive elimination steps were facile and (3) identification of an overarching reaction mechanism consistent with an iron(ii)/iron(iii)/iron(i) cycle (Scheme 2).

While a critical advance in our understanding of the mechanistic pathways effective for iron-catalysed, triazole-directed C–H arylation, many alternative C–H functionalisations with iron have been reported. Whereas the C–H arylation system forms a C–C bond to a nucleophile, which is also required for C–H activation (Scheme 2), there are numerous examples of C–H functionalisation reactions which incorporate aryl nucleophiles into the reaction protocol but establish C–C and C–heteroatom bonds with external electrophiles. Key examples include Cook's use of an alkyl chloride for benzoquinoline alkylation,^18^ Nakamura's use of *N*-chloromorpholine for C–H amination,^22^ and Ackermann's use of allyl chloride to facilitate C–allyl bond formation (Scheme 1).^36^ This important sub-class of C–H activation/functionalisation reactions which utilise electrophiles to achieve C–C and C–heteroatom bond formation enable a wide range of molecular complexity to be introduced. This unique reactivity suggests these reactions undergo a fundamentally distinct reaction coordinate to achieve C–C and C–heteroatom bond formation compared to that identified for C–H nucleophilic arylation. Defining the unique reaction pathway which enables product formation upon reaction with electrophiles (as opposed to nucleophiles in C–H arylation) is essential in order to provide the necessary fundamental foundation to facilitate further method development in iron-catalysed C–H activation/functionalisation.

Towards this goal, we investigate the mechanism of the iron-catalysed C–H allylation system reported by Ackermann and co-workers (Scheme 1c) as a representative example in this important C–H functionalisation sub-class utilising electrophiles.^36^ Detailed spectroscopic studies enable the identification of the key iron intermediates in this system and, combined with correlated reactivity studies, identify a novel reaction pathway of a cyclometalated iron intermediate with allyl chloride that enables the efficient and selective formation of the desired product over the undesired competing C–H arylation reaction. Computational studies provide further insight into this novel reaction coordinate, identifying a radical pathway that leads to C–H allylation and highlights the importance of the bisphosphine ligand coordination flexibility in this transformation. Lastly, additional spectroscopic studies of Nakamura's C–H amination system (Scheme 1d) demonstrate the broader importance of this reaction pathway across C–H activation/functionalisation reactions which utilise electrophiles to establish C–C and C–heteroatom bond formation.

## Results and discussion

### Iron speciation in triazole-directed C–H allylation

Towards the goal of defining the unique reaction pathway that enables C–H allylation, initial studies focused on identifying the key iron intermediates present in iron-catalysed, triazole-assisted C–H allylation (Scheme 1c).^36^ Stoichiometric reactions of Fe(acac)_3_, dppe, and H-sub-2 (see Scheme 1) with various equivalents of phenylmagnesium bromide (PhMgBr) were performed under catalytically relevant conditions (*i.e.* solvent, temperature, iron concentration, *etc.*) to identify the iron species accessible *in situ*.

**Fig. 1:**
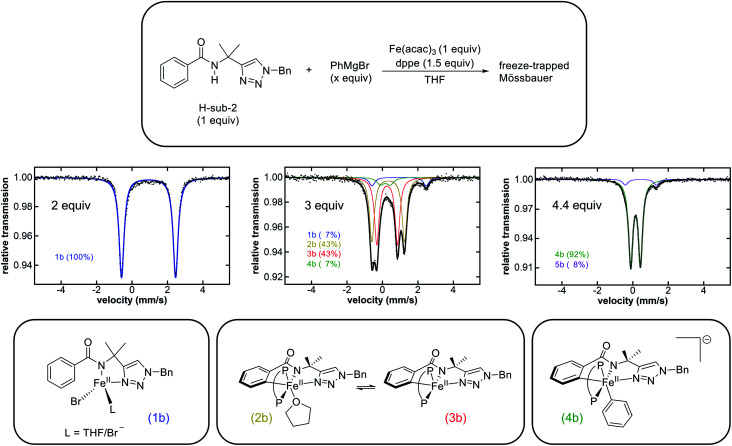
Reaction scheme of H-sub-2, Fe(acac)_3_, and dppe (P–P) with various stoichiometric equivalents of PhMgBr (top). Freeze-trapped 80 K Mössbauer spectra (middle) following the addition of PhMgBr. Proposed structures (bottom) of the major species in each stoichiometric reaction. The reaction with 2 equivalents of PhMgBr was performed without dppe at RT. The reactions with 3 equivalents and 4.4 equivalents of PhMgBr were performed at RT and 65 °C, respectively.

Whereas the reaction of Fe(acac)_3_ with 1 equivalent of PhMgBr simply reduces the iron(iii) salt to iron(ii),^38^ the dropwise addition of a mixture of PhMgBr (2 equivalents) and H-sub-2 (1 equivalent) in THF to a solution of Fe(acac)_3_ (1 equivalent) in THF at either room temperature (RT) or 65 °C (the temperature employed in catalysis) results in a yellow solution containing a single iron species as determined by freeze-quench 80 K ^57^Fe Mössbauer spectroscopy (*δ* = 0.94 mm s^−1^ and Δ*E*_Q_ = 3.05 mm s^−1^) (**1b**, Fig. 1 and ESI, Fig. S1†). Performing this reaction with dppe yields the same predominant iron species (ESI, Fig. S2†), indicating that dppe is not coordinated to **1b**. The Mössbauer parameters of **1b** are indicative of a substrate-bound high-spin iron(ii) complex analogous to that present in the triazole-directed arylation system (Scheme 2, **1a**, *δ* = 0.94 mm s^−1^ and Δ*E*_Q_ = 3.14 mm s^−1^).^38^ This is consistent with the ability to alternatively form **1b** using FeCl_2_ and deprotonated H-sub-2 (ESI, Fig. S3†) and was further confirmed by the 5 K, 7 T near-infrared magnetic circular dichroism (NIR-MCD) spectrum of *in situ* generated **1b** (ESI, Fig. S4†). The NIR-MCD spectrum contained two low-energy ligand-field transitions at ∼6750 cm^−1^ and ∼7450 cm^−1^ (10*Dq*(*T*_*d*_) = 7055 cm^−1^), analogous to the four-coordinate distorted tetrahedral complex **1a** (Scheme 2) in the C–H arylation system.^38^

In order to target potential C–H activated, cyclometalated intermediates, the stoichiometric reactions were extended to the use of more than 2 equivalents of Grignard reagent. The dropwise addition of a solution of H-sub-2 (1 equivalent) and PhMgBr (3 equivalents) to a solution of Fe(acac)_3_ (1 equivalent) and dppe (1.5 equivalents) at either RT (Fig. 1) or 65 °C (ESI, Fig. S5†) resulted in a colour change of the solution from yellow to dark green. The corresponding 80 K ^57^Fe Mössbauer spectrum of the freeze-quenched reaction solution following addition of PhMgBr at RT revealed two major iron species, ascribed as **2b** (43% of total iron) and **3b** (43% of total iron), with parameters *δ* = 0.33 mm s^−1^ and Δ*E*_Q_ = 1.84 mm s^−1^ and *δ* = 0.27 mm s^−1^ and Δ*E*_Q_ = 1.17 mm s^−1^ respectively (Fig. 1). Additionally, two minor species were present and assigned as **1b** and a new species **4b** (*vide infra*). When the reaction was performed at 65 °C, the same four iron species are also observed with the amount of **2b** and **3b** reduced slightly (ESI, Fig. S5†). The corresponding 10 K EPR analysis indicated that no EPR active iron species are observed to form at either RT or 65 °C. The observed ^57^Fe Mössbauer parameters (Table 1) allow these species to be assigned as the direct analogues to complexes **2a** and **3a** (*δ* = 0.30 mm s^−1^ and Δ*E*_Q_ = 1.92 mm s^−1^ and *δ* = 0.24 mm s^−1^ and Δ*E*_Q_ = 1.19 mm s^−1^, respectively) in the arylation system (Scheme 2), which are low-spin cyclometalated iron(ii) species related *via* an equilibrium based on THF coordination.^38^ Further supporting these assignments, when a solution of **2b** and **3b** was prepared in THF followed by solvent removal *in vacuo* and re-dissolution in 2-MeTHF, the **2b** concentration diminished with a concomitant increase in **3b** concentration as indicated by 80 K ^57^Fe Mössbauer spectroscopy (ESI, Fig. S6†). In addition, quenching the **2b**/**3b** reaction mixture with D_2_O led to a new aryl-deuterium signal identified by ^2^H NMR, consistent with these complexes being C–H activated and further supporting their assignments as cyclometalated species (ESI, Fig. S7†). Further consistent with these assignments, the reaction of **2b**/**3b** with 1 equivalent of Grignard reagent generates **4b** for which the structure has been previously established (*vide infra*).^38^ The corresponding ^1^H NMR spectrum of *in situ* generated **2b**/**3b** (ESI, Fig. S8†) further supported that these complexes are diamagnetic, consistent with low-spin iron(ii) complexes as previously reported for the analogous species **2a**/**3a**.^38^ The transition from a high-spin iron(ii) species **1b** to the low-spin iron(ii) species **2b**/**3b** is consistent with the strong iron–ligand chelation from the tridentate triazole-bearing benzamide and the bidentate bisphosphine ligand. Note that the C–H activation is facile at 65 °C with 84% of all iron cyclometalated within one minute (ESI, Fig. S5†). Lastly, these complexes also exhibited good thermal stability with no decomposition observed at RT over 2 h and minimal loss (∼4%) of the total amount of **2b**/**3b** 10 min after Grignard reagent addition at 65 °C (ESI, Fig. S9†). This observed rate of **2b**/**3b** decomposition at 65 °C was far slower than their reactivities with the electrophile (*vide infra*).

**Table 1:** 80 K ^57^Fe Mössbauer parameters of identified iron species in selected iron-catalysed C–H allylation and C–H amination systems

Complex	Sample	*δ* (mm s^−1^)	Δ*E*_Q_ (mm s^−1^)
**Allylation system**			
**1b**	Frozen soln	0.94	3.05
**2b**	Frozen soln	0.33	1.84
**3b**	Frozen soln	0.27	1.17
**4b**	Frozen soln	0.16	0.55
	Solid^a^	0.17	0.56
**5b**	Frozen soln	0.45	1.76

**Amination system**			
**3c**	Frozen soln	0.28	1.35

a Values obtained from ref. 38.

Following the generation of the cyclometalated iron species **2b** and **3b** upon reaction with 3 equivalents of PhMgBr, the addition of 1 equivalent of PhMgBr (4 equivalents in total) resulted in a rapid colour change from green to red. The corresponding 80 K ^57^Fe Mössbauer spectrum of the freeze-quenched solution revealed the generation of **4b** as the predominant iron species (75% of all iron) with parameters *δ* = 0.16 mm s^−1^ and Δ*E*_Q_ = 0.55 mm s^−1^ along with some remaining **2b** and **3b** as minor species (ESI, Fig. S10†). The amount of **4b** generated *in situ* could be increased to 92% by using a total of 4.4 equivalents of PhMgBr (Fig. 1). A new minor iron species **5b** (8% of all iron) with parameters *δ* = 0.45 mm s^−1^ and Δ*E*_Q_ = 1.76 mm s^−1^ was also observed. No EPR active species were observed to form by 10 K EPR spectroscopy. Complex **4b** has been previously identified by X-ray crystallography and NMR as the low-spin iron(ii) arylated analogue of **3b**, exhibiting identical ^57^Fe Mössbauer parameters as observed in these stoichiometric reactions.^38^ Additionally, no decomposition or reductive elimination of **4b** was observed even after 1 h at 65 °C (ESI, Fig. S11†). The minor species **5b** can be assigned as an Fe(η^6^-biphenyl)(dppe) complex, due to its analogous Mössbauer parameters to the iron(0) species Fe(η^6^-biaryl)(dppbz) (*δ* = 0.43 mm s^−1^ and Δ*E*_Q_ = 1.70 mm s^−1^) previously identified for the related C–H arylation system.^38^ Consistent with this assignment, **5b** can also be formed simply by the reaction of FeCl_2_ with 1 equivalent of dppe and 3 equivalents of PhMgBr (ESI, Fig. S12†).

### Kinetics for C–H activation and transmetalation

After determining the cyclometalated intermediates accessible in this system, the individual rates of C–H activation and transmetalation were evaluated to gain a better understanding of the underlying kinetics. The rate of C–H activation was determined by the reaction of *in situ* generated **1b** with excess PhMgBr (13 equivalents) at 65 °C. The resultant 80 K ^57^Fe Mössbauer analysis revealed the formation of the iron–aryl cyclometalated species **4b** within 30 s. Unfortunately, these conditions also generated a significant amount of over-reduced iron species (ESI, Fig. S13†). An alternative experiment was performed where a solution of Fe(acac)_3_ (1 equivalent) and dppe (1.5 equivalents) was mixed with a solution of H-sub-2 (1 equivalent) and PhMgBr (15 equivalents) at 65 °C. Once again, Mössbauer spectroscopy revealed the completion of the reaction within 30 s with the corresponding formation of **4b** and reduced iron side species (ESI, Fig. S14†). The generation of **4b** within 30 s in both experiments suggests that both the C–H activation and subsequent transmetalation are rapid processes with a corresponding pseudo-first-order rate constant *k* > 2 min^−1^. The rate of transmetalation from **2b**/**3b** to generate **4b** was then directly assessed by reacting *in situ* generated **2b**/**3b** with excess PhMgBr (12 equivalents) at 65 °C, monitoring the formation of **4b** by freeze-quenched 80 K ^57^Fe Mössbauer spectroscopy (ESI, Fig. S15†). Notably, the transmetalation reaction was complete within the first accessible freeze-trapped time point (*t* = 30 s).

The stoichiometric reaction studies above indicate that analogous, cyclometalated iron intermediates can form in both the allylation and arylation systems (Fig. 1 and Scheme 2),^35,36,38^ despite the use of different supporting ligands (dppbz *vs.* dppe) and nucleophiles (Ar_2_Zn *vs.* ArMgBr). The key differences observed were the increased rates of both the formation of **2b**/**3b** and their subsequent transmetalation to form **4b** in the allylation system, attributed to the use of PhMgBr (allylation system) compared to Ar_2_Zn (arylation system). However, it was also critical to evaluate if the same iron species are observed in both systems during catalysis. The 80 K ^57^Fe Mössbauer spectrum of the catalytic allylation reaction 5 minutes into catalysis indicated the presence of predominately a high-spin iron(ii) species with parameters nearly identical to **1b**, though contributions from the product bound analogue of **1b** cannot be excluded (ESI, Fig. S16†). Furthermore, no EPR active species were observed at the corresponding time point by 10 K EPR spectroscopy. This observation is consistent with C–H activation being rate-limiting in the allylation system.^36^ This is in contrast to the C–H arylation system where transmetalation to generate the arylated species was the rate-limiting step.^38^

### Identification of the reactive iron species for formation of the allylation product

**Fig. 2:**
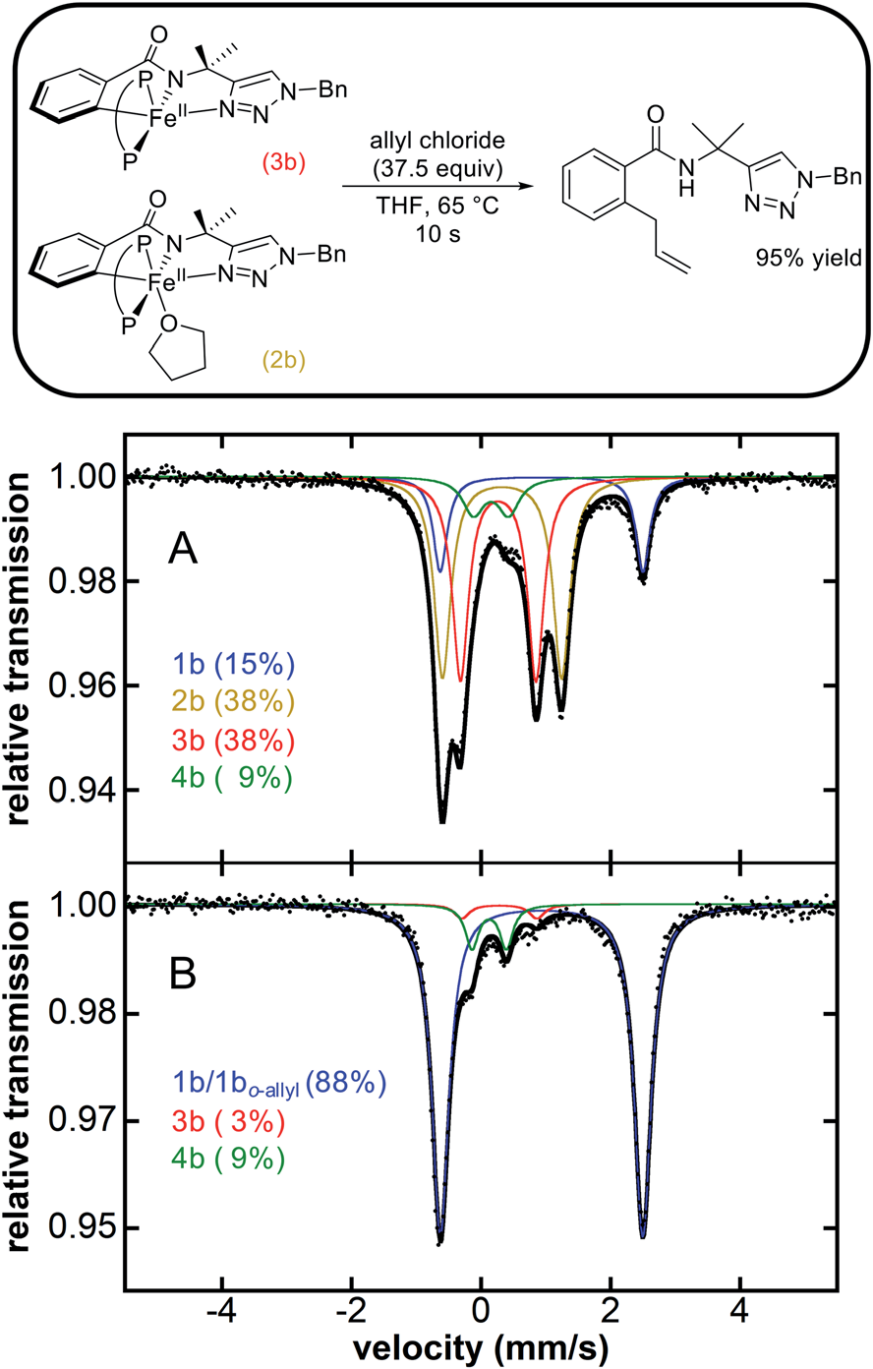
Stoichiometric reaction of **2b** and **3b** with allyl chloride to selectively generate allylated product (P–P = dppe). Corresponding iron distribution as determined by 80 K ^57^Fe Mössbauer (A) before and (B) 30 s after reaction with allyl chloride. The product yield was determined *via*^1^H NMR analysis of the reaction quenched in H_2_O at 10 s. See ESI Fig. S17† for additional reaction data.

The observation that similar cyclometalated iron intermediates are accessible in the C–H allylation system as previously observed for C–H arylation (Fig. 1 and Scheme 2) prompted studies of the reactivities of these intermediates towards allyl chloride, as the high selectivity towards C–H allylation suggests a novel reaction pathway is likely present in this system. The reaction of an *in situ* generated mixture of **2b**/**3b** at 65 °C with excess allyl chloride (37.5 equivalents) (Fig. 2) resulted in a nearly instantaneous colour change of the solution from green to yellow. Time-resolved, chemical-quench NMR analysis indicated an exceptionally rapid reaction generating 95% yield of C–H allylated benzamide (Fig. 2 and ESI Fig. S17†) within 10 s with respect to the initial amount of **2b**/**3b** present *in situ* (*k*_obs_ > 6 min^−1^). Product formation corresponded to consumption of **2b** and **3b** as confirmed by freeze-quenched 80 K ^57^Fe Mössbauer spectroscopy of the reaction. This revealed the complete consumption of **2b**/**3b** and corresponding generation of a high-spin iron(ii) species with parameters of *δ* = 0.94 mm s^−1^, Δ*E*_Q_ = 3.12 mm s^−1^ (ESI, Fig. S17†). These parameters are nearly identical to **1b** and assigned as the C–H allylated product bound analogue **1bo-allyl**.

**Scheme 3:**
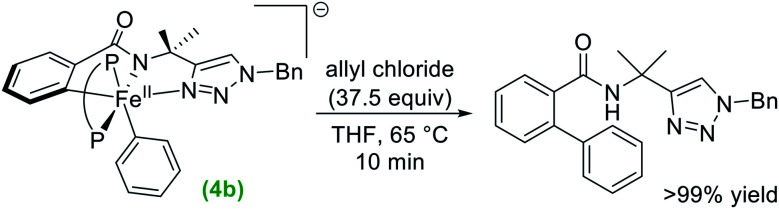
Stoichiometric reaction of *in situ* generated **4b** with allyl chloride generates arylated product (P–P = dppe).

While the reaction of **2b**/**3b** with allyl chloride leads to rapid and selective formation of allylated product consistent with their role as the key reactive intermediates (Fig. 2), it was also interesting to consider whether cyclometalated species **4b** was also reactive towards allyl chloride. Reaction of **4b** with excess allyl chloride (37.5 equivalents) at 65 °C resulted in a much slower colour change (in comparison to **2b**/**3b**; *vide supra*) of the solution from red to yellow over the course of 10 minutes (Scheme 3). Further, freeze-trapped 80 K ^57^Fe Mössbauer spectroscopy of the reaction revealed the consumption of **4b** with simultaneous generation of a high spin iron(ii) species with parameters *δ* = 0.93 mm s^−1^ and Δ*E*_Q_ = 3.17 mm s^−1^ nearly identical to **1b** (ESI, Fig. S18†) and assigned as the C–H arylated product bound analogue **1bo-aryl**.

Finally, an analysis of the kinetics was performed based on the consumption of **4b** as a function of time by freeze-quenched ^57^Fe Mössbauer spectroscopy, with an observed pseudo-first-order rate constant of *k*_obs_ = 0.32 ± 0.04 min^−1^ (ESI, Fig. S19†). The corresponding ^1^H NMR analysis of the organic reaction products indicated only the selective and complete formation of arylated product occurs (Scheme 3) with respect to **4b** at all time points, including those where the reaction is incomplete (as determined by ^57^Fe Mössbauer analysis). This observation is consistent with product formation simply due to the quenching of **4b**, an effect previously observed for the quenching of **4a** in the related arylation system.^38^ Thus, while **4b** can react with allyl chloride, this reaction is an order of magnitude slower than the corresponding reaction of **2b**/**3b** with allyl chloride and also generates the undesired, arylated product. Also note, just as the iron–aryl complex **4a** was shown to react with DCIB to generate arylated product in the C–H arylation system (*k*_obs_ = 0.18 ± 0.04 min^−1^),^38^ the iron–aryl cyclometalated intermediate **4b** can also react with DCIB to generate *ortho*-C–H arylated benzamide at a similarly slow rate (*k*_obs_ = 0.27 ± 0.04 min^−1^) (ESI, Fig. S20 and S21†). These results suggest that the arylation reaction pathway is initiated *via* oxidation by allyl chloride. Notably, when the Fe–aryl complex was prepared with tolyl- and 4-MeO-phenyl Grignard reagents, the reactions with excess allyl chloride were defined by larger pseudo-first-order rate constants *k* = 0.65 min^−1^ and 0.73 min^−1^, respectively. When compared to the rate of C–H arylation with **4b**, these results demonstrate that increasing electron-donating character on the aryl ligand accelerates the reaction.

**Scheme 4:**
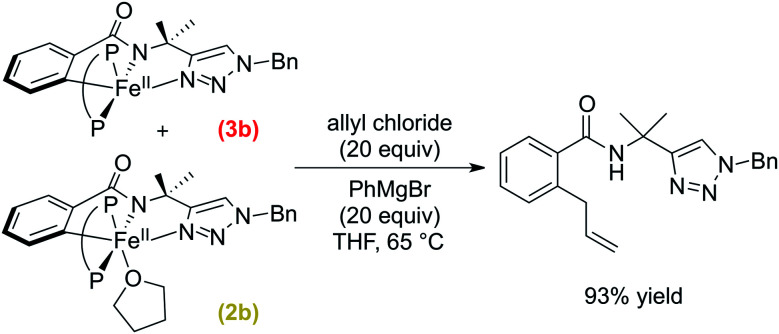
Competition experiment to determine if transmetalation of **2b**/**3b** or their reaction with allyl chloride is kinetically faster (P–P = dppe).

The reactivity and selectivity of the cyclometalated intermediates indicate catalysis proceeds *via* reaction of **2b**/**3b** with allyl chloride to achieve selective product formation. This would suggest that reaction of **2b**/**3b** with allyl chloride must also be faster than the transmetalation of **2b**/**3b** with PhMgBr in order to disfavour formation of **4b** which, following reaction with allyl chloride, would generate undesired arylated product (*vide supra*). Unfortunately, these competing rates are too fast to track by freeze-quench ^57^Fe Mössbauer at 65 °C due to the minimal freezing times required. Therefore, to probe which reaction is faster, a competition experiment was conducted by reacting a solution of *in situ* generated **2b**/**3b** with a solution containing equimolar amounts of excess allyl chloride and PhMgBr (20 equivalents of each with respect to total iron) at 65 °C (Scheme 4). ^1^H NMR analysis of the reaction quenched after 90 s revealed the formation of the C–H allylated product as the predominant product (93% yield with respect to the initial amount of **2b**/**3b**) (ESI, Fig. S22†). These results demonstrate that reaction of allyl chloride with **2b**/**3b** is faster than their transmetalation to form **4b**.

### Intermediates and reaction pathways for iron-catalysed triazole-directed C–H allylation

**Scheme 5:**
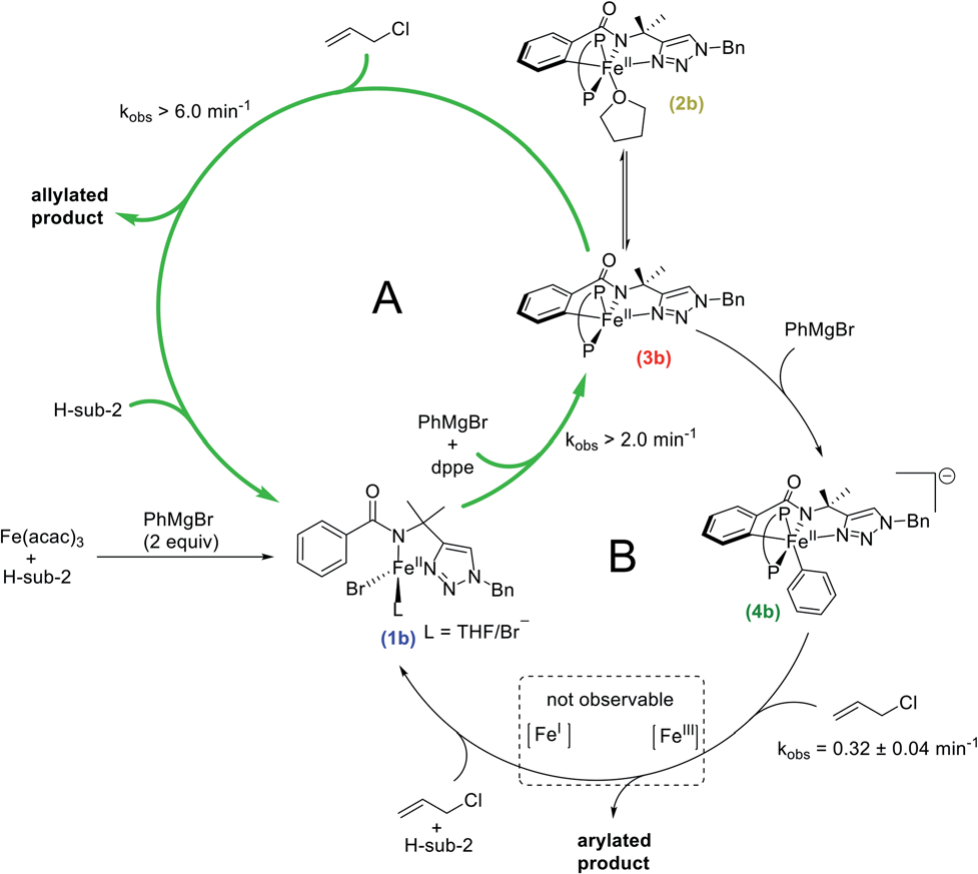
Experimentally defined intermediates and reaction pathways in iron-catalysed C–H allylation with triazole assistance (P–P = dppe). Pathway A facilitates *ortho*-C–H allylation. Pathway B facilitates *ortho*-C–H arylation.

The spectroscopic and reaction studies described above are consistent with the following mechanistic cycle for iron-catalysed C–H allylation (Scheme 5). The first two equivalents of the nucleophile (PhMgBr) serve to deprotonate the substrate amide and reduce the ferric salt to an iron(ii) species permitting the formation of a high spin iron(ii) substrate-bound species, **1b**. An additional equivalent of PhMgBr along with the supporting phosphine ligand, dppe (1.5 equivalents), activates the *ortho*-C–H position on the substrate generating two cyclometalated low-spin iron(ii) complexes, one bearing a THF ligand (**2b**) and one without (**3b**). Treatment of **2b**/**3b** with an excess of electrophile (allyl chloride) results in the generation of a high yield of *ortho*-C–H allylated product at a catalytically relevant rate. Furthermore, when **2b**/**3b** are treated with an additional equivalent of PhMgBr, another cyclometalated low-spin iron(ii) complex can be generated, which bears an iron–phenyl bond (**4b**). Treatment of **4b** with excess oxidant (allyl chloride or DCIB) leads to the formation of an *ortho*-C–H arylated product in good yield, but at reaction rates more than an order of magnitude slower than the corresponding reaction of **2b**/**3b** with the electrophile.

While the reaction of allyl chloride with the cyclometalated intermediates **2b**/**3b** is directly responsible for generating allylated product in high yields (Fig. 2), it is interesting to consider the reaction pathway involved compared to the reaction of allyl chloride with **4b**. First, both allyl chloride and DCIB are competent in reactions with **4b** to generate arylated product (Scheme 3 and ESI, Fig. S20†), where similar reaction rates are observed for both oxidants (ESI, Fig. S19 and S21†). These reactions are consistent with an oxidation process leading to an Fe(iii) intermediate that can reductively eliminate to generate product, akin to the reaction pathway previously proposed for the related C–H arylation system in which a similar reaction rate of **4a** with DCIB is observed.^38^ In stark contrast, **2b**/**3b** were found to exhibit limited to no reactivity towards DCIB beyond an existing slow (previously established) background thermal decomposition (ESI, Fig. S23†), consistent with the fact that **2b**/**3b** should be more difficult to oxidise than **4b** as they lack the additional strong donor phenyl ligand present in the latter. However, **2b**/**3b** are incredibly reactive towards allyl chloride and far more reactive than **4b** with the same substrate, suggesting that the allylation reaction with **2b**/**3b** may follow an inner-sphere reaction pathway (*e.g.* nucleophilic substitution) likely proceeding *via* an η^3^-allyl intermediate (or loosely associated allyl radical) as previously proposed based on observed regioselectivities in this system.^36^

### Quantum mechanical calculations of the nature of C–C bond formation in the C–H allylation pathway

**Fig. 3:**
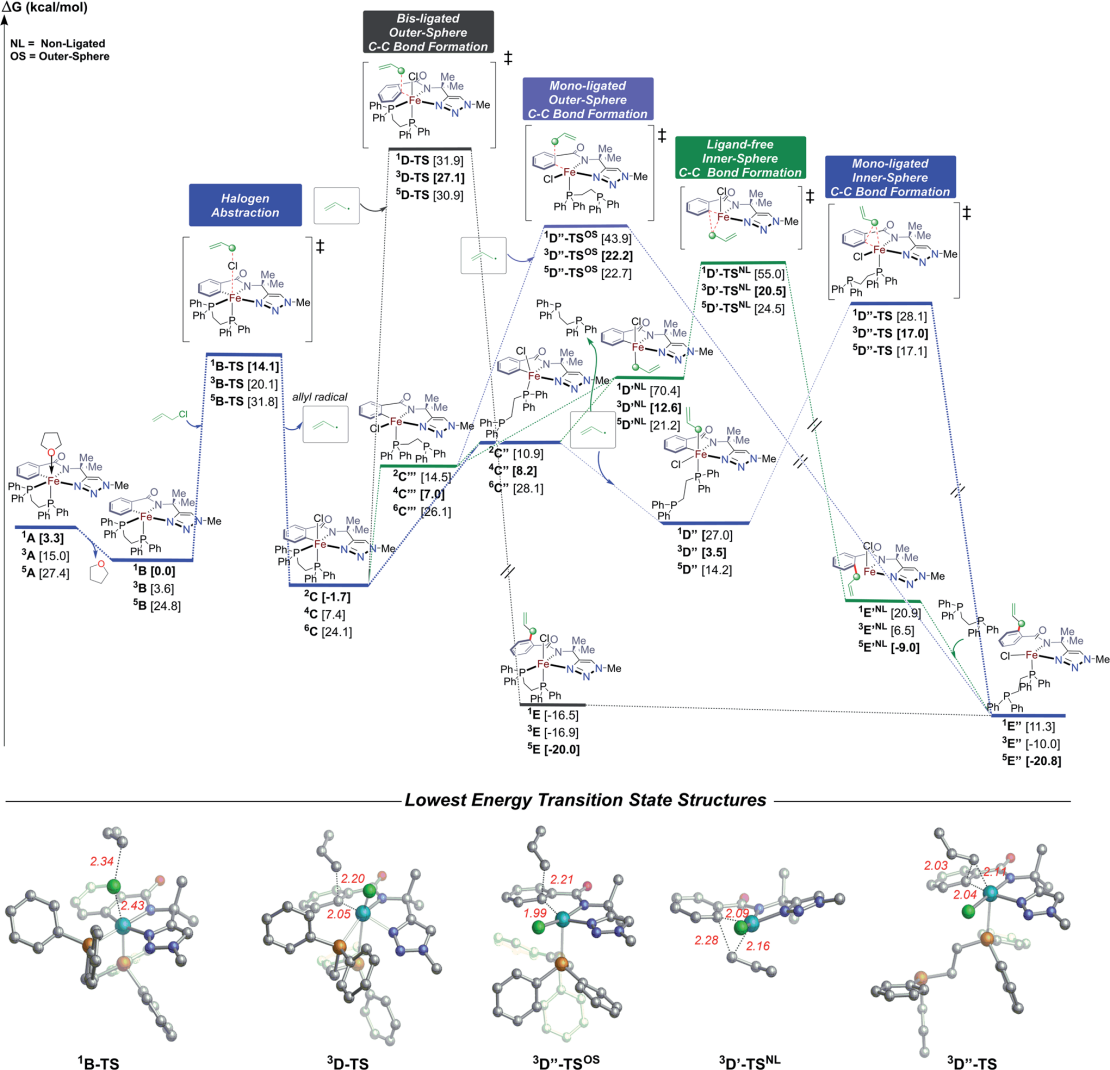
Energetic profile for the Fe-promoted C–H allylation. Free energies (kcal mol^−1^) were computed at the UPBEPBE-D3/def2-SVP-SMD(THF)//UB3LYP/def2-SVP(gas) (in bracket) levels of theory.

Having identified a novel pathway for Fe-catalysed C–H allylation, it was important to further define the details of this reaction coordinate (*i.e.* inner-sphere or outer-sphere). Towards this goal, we turned to quantum mechanical calculations (see ESI for details and method comparisons†). As shown in Fig. 3, consistent with the experiment, the calculations indicate that low spin **1A** could equilibrate to the de-solvated **1B** species (energy difference is only ∼3 kcal mol^−1^). In turn, **1B** with the axial coordinating site could then promote rapid halogen atom abstraction from allyl chloride (barrier is only ∼14 kcal mol^−1^ at this method) to form the corresponding allyl radical and **2C**, which is only 1.7 kcal mol^−1^ lower in energy in comparison to **1B**. From **2C**, we found that the lowest energy pathway (see ESI for alternative mechanism†) proceeds *via* an inner-sphere mechanism involving a partial dissociation of the bisphosphine ligand. Specifically, the six-coordinate **2C** could dissociate the axial or equatorial phosphine (to form **4C′** or **4C′′′**; see ESI†) to form, after ligand rotation, a distorted five-coordinate square pyramidal species **4C′′**. This Fe(iii) species could then trap the allyl radical from the axial position (barrierless) to form the more energetically stable six-coordinate **3D′′** species. Finally, this structure is poised to undergo reductive elimination (*via***3D′′-TS**) to form the experimentally detected high-spin iron(ii) product bound structure **5E′′**. We have also considered alternative C–C bond formation pathways including direct bis-ligated outer-sphere C–C bond formation from **2C** but this was found to be much higher in energy (>10 kcal mol^−1^; see ESI†). A similar mechanism was identified using DCIB as substrate (see ESI†). For alternative, but higher energy mechanisms see ESI.† Overall, these results show that the flexibility of the ligand is important to promote allylation and could play a major role in controlling product selectivity (allylation *vs.* arylation). These calculations are ongoing and will be reported in due course.

### Assessing the effects of bisphosphine type on C–H allylation rate and selectivity

Following the computational studies, we found that the flexibility of the supporting bisphosphine ligand plays an integral role towards reactivity in the C–H allylation pathway. Therefore, we turned to the C–H arylation system (Scheme 1a), which uses a similar substrate (H-sub-1) but employs dppbz as the supporting ligand. We were interested to see if the more rigid benzene backbone of this ligand would inhibit this system from selectively generating C–H allylated product upon the reaction of **2a**/**3a** with allyl chloride.

**Scheme 6:**
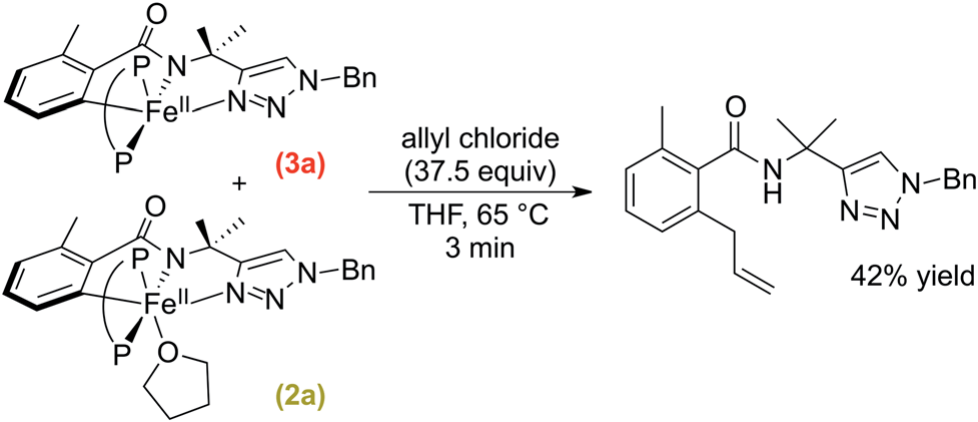
Stoichiometric reaction of **2a** and **3a** with allyl chloride to generate allylated product (P–P = dppbz).

A solution of **2a**/**3a** was prepared *in situ* following the published procedure^38^ and subsequently reacted with excess allyl chloride (37.5 equivalents) at 65 °C (Scheme 6). The reaction was monitored by freeze-quenched ^57^Fe Mössbauer spectroscopy which revealed the consumption of complexes **2a** and **3a** with the concomitant generation of a new iron species with parameters consistent with a product-bound high-spin iron(ii) complex (ESI, Fig. S24†). The consumption of **2a** and **3a** exhibited an observed pseudo-first-order rate constant of *k* = 1.5 ± 0.2 min^−1^ (ESI, Fig. S25†). A corresponding NMR study revealed that *ortho*-C–H allylated benzamide was formed but only in 42% yield with respect to consumed **2a** and **3a**.

While the above results indicate the triazole directed system reported for C–H arylation can also access the C–H allylation pathway, the reaction is slower and less selective when compared to the stoichiometric allylation reaction with **2b**/**3b** (Fig. 2). This observation is consistent with the ability of the bisphosphine to dissociate and rotate in the inner-sphere radical pathway that leads to C–H allylated product formation (Fig. 3).

### An analogous electrophile-facilitated pathway for C–N bond formation in quinoline-directed C–H amination system

Our studies have now identified a novel reaction coordinate for C–H allylation proceeding *via* an inner-sphere radical pathway, demonstrating how this particular system reacts with an electrophile to establish C–C bond formation. We were then curious to explore whether this pathway is applicable to a broader set within this sub-class of iron-catalysed C–H functionalisation reactions. Therefore, we turned our focus to the 8-aminoquinoline-directed iron-catalysed C–H amination of benzamides with an *N*-chloromorpholine electrophile reported by Nakamura and co-workers in order to define the broader importance of this new pathway (Scheme 1d).^22^

**Fig. 4:**
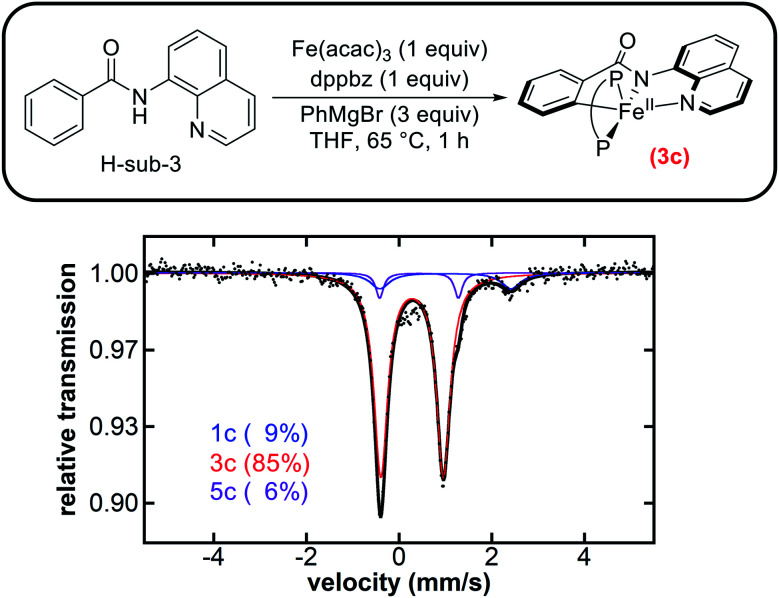
Freeze-quenched 80 K ^57^Fe Mössbauer spectrum of the *in situ* formed iron species from the reaction of Fe(acac)_3_ and dppbz (P–P) with H-sub-3 and 3 equivalents of PhMgBr.

Initial studies focused on the potential generation of cyclometalated iron species analogous to those observed in both the triazole-directed C–H allylation and arylation systems.^35,36,38^ The supporting ligand dppbz was used in place of F-dppbz for the amination studies as it was more accessible and previously employed by Nakamura and co-workers for stoichiometric amination studies with H-sub-3.^22^ A solution of Fe(acac)_3_ and dppbz was treated with quinolinamide (H-sub-3) and 3 equivalents of PhMgBr. The corresponding freeze-quenched ^57^Fe Mössbauer spectrum revealed the generation of one predominant iron species accounting for 85% of the total iron in solution with ^57^Fe Mössbauer parameters (*δ* = 0.28 mm s^−1^, Δ*E*_Q_ = 1.35 mm s^−1^) (Fig. 4), analogous to the parameters previously observed for **3a** (*vide supra*) and **3b** (Table 1). As such, this species is assigned as the five-coordinate, low-spin cyclometalated iron(ii) complex **3c**. Note that two minor iron species were present with Mössbauer parameters (*δ* = 1.00 mm s^−1^, Δ*E*_Q_ = 2.83 mm s^−1^) and (*δ* = 0.43 mm s^−1^, Δ*E*_Q_ = 1.70 mm s^−1^) analogous to those of **1b** and **5b**, and were assigned as **1c** and **5c**, respectively (Fig. 4). In addition, an assessment of the iron species present during catalysis was performed using the published reaction protocol^22^ and also revealed **3c** as the predominant species at 5 minutes into the catalytic reaction (ESI, Fig. S26†), suggesting that C–H activation is not the rate-determining step in this catalysis which was also the case for triazole-directed C–H arylation.

**Scheme 7:**
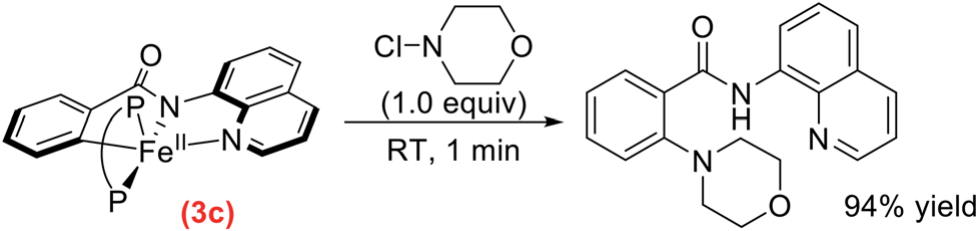
Stoichiometric reaction of **3c** with *N*-chloromorpholine to generate C–H aminated product (P–P = dppbz).

As the related intermediate in the C–H allylation system is responsible for reaction with an electrophile to generate product, the reactivity of **3c** with *N*-chloromorpholine was investigated as a possible pathway to C–N bond formation to form product. The reaction of *in situ* generated **3c** with *N*-chloromorpholine (1 equivalent) (Scheme 7) resulted in the complete consumption of **3c** within 1 minute (ESI, Fig. S27†). Product formation was monitored by LCMS and revealed the generation of C–H aminated product with 94% yield with respect to the initial amount of **3c** (ESI, Table S1†). These results reveal that the reaction of **3c** with electrophile selectively generates C–H aminated product at a rapid rate, which is consistent with the inner-sphere reaction pathway that defined the C–H allylation reaction. This demonstrates that this novel inner-sphere radical pathway is likely accessible over a broad range of systems that fall within this sub-class of reactions.

## Conclusions

The iron-catalysed system for the triazole-assisted *ortho*-C–H allylation of benzamides with allyl electrophiles accesses iron intermediates analogous to those involved in the iron-catalysed C–H arylation of benzamides.^38^ Two of these intermediates are low-spin iron(ii) complexes, which react with allyl chloride to selectively and rapidly generate *ortho*-C–H allylated product. Supporting computational studies reveal that the underlying allylation reaction pathway is consistent with an inner-sphere radical reaction mechanism, which involves the partial dissociation and rotation of the bisphosphine ligand. Alternatively, these C–H activated complexes can also transmetalate with aryl nucleophile to form another cyclometalated intermediate bearing an iron–phenyl bond. The iron–aryl intermediate reacts with oxidant to selectively generate *ortho*-C–H arylated product at a significantly slower rate than the allylation pathway. The quinoline-directed iron-catalysed C–H amination system which relies on electrophile to establish the C–N bond was also shown to follow an analogous reaction pathway to the C–H allylation system. These results demonstrate that this reaction manifold is pervasive over a broad range of iron-catalysed C–H activation/functionalisation systems, which rely on electrophiles to establish new C–C and C–heteroatom bonds. Overall, these results provide critical, molecular-level insight into intermediates and reaction pathways, which provide a foundation for the rational design and development of improved systems that are efficient, selective, and useful across a broad range of C–H functionalisations.

## Data availability

Details about computational methods, associated coordinates, and energies are available in the ESI. Additional spectroscopic data is provided in the ESI.

## Author contributions

(J. C. D., Z. S.) These authors contributed equally. J. C. D., S. H. C., T. E. B., and T. M. B. provided the experimental/spectroscopic data. Z. S. and O. G. contributed to the computational study of this paper. A. R. provided the computational Mössbauer parameter calculations. M. N. and O. G. supervised this work, and all authors contributed to the writing of this manuscript.

## Conflicts of interest

The authors declare no conflicts of interest.

## Supplementary Material

SC-012-D1SC01661J-s001

SC-012-D1SC01661J-s002
