# Supplementary material for: Experimental and computational studies of the mechanism of iron-catalysed C–H activation/functionalisation with allyl electrophiles
Source: Chem Sci. 2021 Jun 17;12(27):9398–407. doi: 10.1039/d1sc01661j (PMC8278975; doi:10.1039/d1sc01661j)
Supplement: SC-012-D1SC01661J-s001 [file SC-012-D1SC01661J-s001.pdf]

Electronic Supplementary Material (ESI) for Chemical Science.
This journal is © The Royal Society of Chemistry 2021

Electronic Supporting Information for

Experimental and Computational Studies of the Mechanism of Iron-Catalysed C-H Activation/Functionalisation with Allyl Electrophiles

Joshua C. DeMuth,‡[a] Zhihui Song,‡[b] Stephanie Carpenter,[a] Theresa E. Boddie,[a] Aleksa Radović,[a] Tessa M. Baker,[a] Osvaldo Gutierrez,*[,b] and Michael L. Neidig*[,a]

[a]Department of Chemistry, University of Rochester, Rochester, New York 14627, USA
[b]Department of Chemistry, University of Maryland, College Park, Maryland 20742, USA

Table of Contents

1 Experimental 2
1.1 General Considerations 2
1.2 Experimental Methods 2
1.2.1 Mössbauer Spectroscopy 2
1.2.2 Electron Paramagnetic Resonance (EPR) Spectroscopy 2
1.2.3 Magnetic Circular Dichroism (MCD) Spectroscopy 2
1.2.4 Liquid Chromatography Mass Spectrometry (LC-MS) 3
1.2.5 ^{1}H, ^{2}H NMR Spectroscopy 3
1.3 Stoichiometric Reaction to Generate In Situ **1b** 3
1.4 Stoichiometric Reaction to Generate In Situ **2b/3b** 3
1.4.1 Reaction of In Situ **2b/3b** with Allyl Chloride 3
1.4.2 Reaction of In Situ **2b/3b** with DCIB 3
1.5 Stoichiometric Reaction to Generate In Situ **4b** 4
1.5.1 Reaction of In Situ **4b** with Allyl Chloride 4
1.5.2 Reaction of In Situ **4b** with DCIB 4
1.6 Stoichiometric Reaction to Generate In Situ **2a/3a** 4
1.6.1 Reaction of In Situ **2a/3a** with Allyl Chloride 4
1.7 Stoichiometric Reaction to Generate In Situ **3c** 5
1.7.1 Reaction of In Situ **3c** with *N*-chloromorpholine 5
1.8 Preparation of *trans*-Fe(dppbz)$_2$(H)(Br) 5
2 Supplementary Data 6
2.1 Spectroscopic Data for the Allylation System 6
2.2 Spectroscopic Data for the Arylation System 25
2.3 Spectroscopic Data for the Amination system 26
3 X-ray Crystallography Data 29
3.1 CCDC Deposition 29
3.2 *trans*-(dppbz)$_2$Fe(H)(Br) 29
4 DFT Calculations Data 49
4.1 Computational Details 49
4.2 Complete Energetic Diagrams 50
4.3 Coordinates and Energies 54
4.4 Isomer Shift Calculations for **1b**, **2b**, **3b** and **4b** 477
5 References 478

1. Experimental

1.1 General considerations. All the starting materials were prepared following previously described methods.[1-3] All reagents were purchased from commercial sources and were employed without additional purification. Anhydrous solvents were filtered through alumina and stored on 4 Å molecular sieves under an inert nitrogen atmosphere. Enriched iron sources $^{57}Fe(acac)_3$ and $^{57}FeCl_2$ were synthesized in accordance with published procedures with ^{57}Fe metal (95% enriched) purchased from Isoflex.[4, 5] An MBraun inert-atmosphere N_2 glovebox was used to perform all moisture- and air-sensitive reactions. The glovebox was equipped with a liquid nitrogen transfer line to enable freezing of reaction samples for spectroscopic analysis. Additional general experimental details are in the supporting information.

1.2 Experimental methods

1.2.1 Mössbauer Spectroscopy. Frozen solution samples were prepared using ^{57}Fe-enriched sources $^{57}Fe(acac)_3$ or $^{57}FeCl_2$ under an inert atmosphere within a glovebox with a liquid nitrogen fill port to freeze-trap solution samples at 77 K. Samples were loaded into Delrin samples cups and then frozen in liquid nitrogen. Low temperature ^{57}Fe Mössbauer measurements were performed using a Janis SVT-400T N_2 cryostat for analysis at 80 K. Isomer shift values were measured relative to an α-Fe standard at 298 K. All Mössbauer spectra were fit using WMoss (See Co.) software. The associated parameters errors in the fit analyses include the following: $\delta \pm 0.02$ mm/s $\Delta E_Q \pm 3\%$. The multicomponent fit analyses have an associated quantitation error of ± 3%. Since only zero-field Mössbauer measurements were performed, all quadrapole splitting parameters reported herein are absolute values.

1.2.2 Electron Paramagnetic Resonance (EPR) Spectroscopy. Catalytic solution EPR samples were prepared from the reaction solutions as reported in the literature.[1, 2] In situ stoichiometric samples were prepared directly from the reaction solutions as described. EPR samples were prepared for spin integration by loading sample solution into high-precision 4 mm OD Suprasil quartz EPR tubes from Wilmad Labglass which allowed direct comparison of intensities between samples. Samples were spin integrated using a $CuSO_4{\bullet}5H_2O$ standard under non-saturating conditions. X-band EPR data was collected using a Bruker EMXplus spectrometer equipped with a 4119HS cavity and an Oxford ESR-900 helium flow cryostat.

1.2.3 Magnetic Circular Dichroism (MCD) Spectroscopy. The frozen solution sample of **1b** was prepared for MCD spectroscopy under an inert atmosphere in a glovebox equipped with a liquid nitrogen transfer port to freeze the sample to 77 K. The sample was prepared in a low-temperature optical glassing solvent mixture 3:2 (v/v) 2-Me-THF/THF and loaded into a copper cell fitted with quartz disks and a 3 mm gasket. A 5 K, 7 T near-infrared (NIR) analysis was performed using a JASCO J-730 spectropolarimeter and a liquid nitrogen cooled InSb detector. The spectrometer contained a modified sample compartment with focusing optics and an Oxford Instruments SM4000-7T superconducting magnet/cryostat. The temperature was measured to the nearest 0.001 K using a calibrated Cernox sensor inserted in the copper sample holder. The MCD spectrum was baseline-corrected against the zero-field scan.

1.2.4 Liquid Chromatography-Mass Spectrometry (LCMS). All LCMS measurements were performed using an Agilent Technologies 1260 Infinity II liquid chromatogram with a Restek Ultra C18 column and Advion Expression L electron spray ionization mass spectrometer. LCMS samples were prepared at 1 mM with phenanthroline as the internal standard.

1.2.5 ^{1}H, ^{2}H Nuclear Magnetic Resonance (NMR) All NMR analyses were performed using a Bruker 400 MHz NMR spectrometer at ambient temperature. Quantitative ^{1}H NMR analyses were performed using a 1,3,5-trimethoxybenzene internal standard. The chemical shifts (δ) are reported in parts per million (ppm), and calibrated using the internal standard (1,3,5-Trimethoxybenzene 6.08 ppm) for ^{1}H NMR or the presence of naturally abundant deuterated solvent signals (THF-d_8 1.73/3.58) for ^{2}H NMR.

1.3 Stoichiometric Reaction to Generate In Situ 1b. As an example of the general procedure to generate in situ **1b**, the reaction of Fe(acac)$_3$ with H-sub-2 and 2 equivalents of PhMgBr is described. A 20 mL scintillation vial was charged with a mixture of ^{57}Fe(acac)$_3$ (4.5 mg, 0.013 mmol), non-enriched Fe(acac)$_3$ (9.0 mg, 0.025 mmol), and then dissolved in THF (2.00 mL) and stirred at RT. A second scintillation vial was charged with H-sub-2 (12.4 mg, 0.039 mmol) and dissolved in THF, then PhMgBr (77.0 µL, 0.0761 mmol, 0.988 M in THF) was added and stirred for 15 minutes to deprotonate H-sub-2. The solution of Fe(acac)$_3$ was pre-heated to 65 °C using an oil recirculation bath, followed by an addition of the solution of H-sub-2/PhMgBr which was allowed to heat at 65 °C for at least one minute resulting in a colour change from red to yellow.

1.4 Stoichiometric Reaction to Generate 2b/3b. As an example of the general procedure to generate in situ **2b/3b**, the reaction of Fe(acac)$_3$/dppe with H-sub-2 and 3 equivalents of PhMgBr is described. A 20 mL scintillation vial was charged with a mixture of ^{57}Fe(acac)$_3$ (11.9 mg, 0.0336 mmol), non-enriched Fe(acac)$_3$ (25.2 mg, 0.0714 mmol), and dppe (62.8 mg, 0.158 mmol) and then dissolved in THF (5.50 mL) and stirred at RT. A second scintillation vial was charged with H-sub-2 (0.0338 mg, 0.106 mmol) and dissolved in THF (5.20 mL), then PhMgBr (315 µL, 0.314 mmol, 0.997 M) was added and stirred for 15 minutes to deprotonate H-sub-2. Then the solution of H-sub-2/PhMgBr was added dropwise to the solution of Fe(acac)$_3$/dppe at RT resulting in a colour change from red to yellow to dark green. The solution was heated to 65 °C for at least 10 min.

1.4.1 Reaction of In Situ 2b/3b with Allyl Chloride (37.5 equiv). Following the procedure outlined above, in duplicate an aliquot of allyl chloride (145 µL, 1.78 mmol) was added to 5.00 mL of the solution of **2b/3b** at 65 °C. At various timepoints 0.40 mL samples of the resulting reaction mixture were then transferred to separate 4 mL scintillation vials containing 0.50 mL H_2O as a quenching agent. In a duplicate experiment, 0.5-1 mL samples of reaction solution were transferred to a Delrin Mössbauer cup and freeze-quenched. The water-quenched reaction solution was filtered through a Celite pad and rinsed with DCM. The internal standard 1,3,5-trimethoxybenzene (1.00 mL, 3.94 mM in DCM) was added to the filtrate and then placed under vacuum to remove all volatiles. Finally, the remaining solids were dissolved in 1 mL of $CDCl_3$ and analyzed by ^{1}H NMR for the allylated product based on signals at chemical shifts reported in the literature.[3]

1.4.2 Reaction of 2b/3b with DCIB (20 equiv). Following the procedure outlined above, DCIB (110 µL, 0.952 mmol) was added to 6.00 mL of the solution of **2b/3b** at 65 °C. A series of 1

mL aliquots of reaction solution were transferred to a Delrin Mössbauer cups and freeze-quenched a various timepoints.

1.5 Stoichiometric Reaction to Generate In Situ 4b. As an example of the general procedure to generate in situ **4b**, the reaction of $Fe(acac)_3$/dppe with H-sub-2 and 4.2 equivalents of PhMgBr is described. A 20 mL scintillation vial was charged with a mixture of $^{57}Fe(acac)_3$ (14.9 mg, 0.0420 mmol), non-enriched $Fe(acac)_3$ (32.1 mg, 0.0909 mmol), and dppe (79.5 mg, 0.200 mmol) and then dissolved in THF (7.00 mL) and stirred at RT for 15 minutes and then pre-heated to 65 °C. A second scintillation vial was charged with H-sub-2 (43.6 mg, 0.136 mmol) and dissolved in THF (6.48 mL), then PhMgBr (520 µL, 0.558 mmol, 1.074 mM) was added and stirred for 15 minutes to deprotonate H-sub-2. Then the solution of H-sub-2/PhMgBr was added dropwise to the solution of $Fe(acac)_3$/dppe at 65 °C resulting in a colour change from red to yellow to dark green to dark red. The solution continued stirring at 65 °C for at least 10 min.

1.5.1 Reaction of In Situ 4b with Allyl Chloride. Following the procedure outlined above, in duplicate allyl chloride (145 µL, 1.78 mmol) was added to 5.0 mL of solution of **4b** at 65 °C. A series of 1.00 mL aliquots of the resulting reaction mixture were then transferred at various timepoints to 4 mL scintillation vials containing 1 mL H_2O as a quenching agent. In a duplicate experiment, 0.5-1 mL aliquots of reaction solution at various timepoints were transferred to a Delrin Mössbauer cup and freeze-quenched. The water-quenched reaction solutions were filtered through Celite pads and rinsed with DCM. The internal standard 1,3,5-trimethoxybenzene (1.00 mL, 9.42 mM in DCM) was added to the filtrate and then placed under vacuum to remove all volatiles. Finally, the remaining solids were dissolved in 1 mL of $CDCl_3$ and analyzed by ^1H NMR for the arylated product based on signals at chemical shifts reported in the literature.[2]

1.5.2 Reaction of In Situ 4b with DClB. Following the procedure outlined above at 55 °C, DClB (110 µL, 0.948 mmol) was added to 5.00 mL of the solution of **2b/3b** at 55 °C. A series of 0.60 mL aliquots of the resulting reaction mixture were then transferred at various timepoints to 4 mL scintillation vials containing 0.5 mL H_2O as a quenching agent. In a duplicate experiment, 0.5-1 mL aliquots of reaction solution at various timepoints were transferred to a Delrin Mössbauer cup and freeze-quenched. The water-quenched reaction solutions were filtered through Celite pads and rinsed with DCM. The internal standard 1,3,5-trimethoxybenzene (1.00 mL, 5.73 mM in DCM) was added to the filtrate and then placed under vacuum to remove all volatiles. Finally, the remaining solids were dissolved in 1 mL of $CDCl_3$ and analyzed by ^1H NMR for the arylated product based on signals at chemical shifts reported in the literature.[2]

1.6 Stoichiometric Reaction to Generate In Situ 2a/3a. A (mL) solution of **2a/3a** was prepared according to the published procedure.[6] A 20 mL scintillation vial was charged with a mixture of $^{57}Fe(acac)_3$ (9.3 mg, 0.026 mmol), non-enriched $Fe(acac)_3$ (15.8 mg, 0.0447 mmol) and dppbz (31.5 mg, 0.0706 mmol) and dissolved in THF (4.90 mL) while stirring at RT. A separate 20 mL scintillation vial was charged with H-sub-1 (23.6, 0.0706 mmol) and $ZnBr_2$•TMEDA (84.7 mg, 0.246 mmol) and dissolved in THF (3.00 mL) at RT with subsequent dropwise addition of 4-$MeOC_6H_4MgBr$ in THF (0.530 mL, 0.494 mmol, 0.932 M), which was subsequently stirred at RT for 10 min to generate in situ $(4\text{-}MeOC_6H_4)_2Zn$, as well as deprotonate H-sub-1. The solution of Fe/dppbz was added dropwise to the solution of H-sub-1/$(4\text{-}MeOC_6H_4)_2Zn$, causing a colour change from red to yellow to dark green, and the mixture was heated at 65 °C for at least 10 min.

1.6.1 Reaction of In Situ 2a/3a with Allyl Chloride. Following the procedure referenced above, 37.5 equivalents of allyl chloride (156 µL, 1.91 mmol) was added to 5.77 mL of solution of

2a/3a at 65 °C. A series of 1.00 mL aliquots of the resulting reaction mixture were then transferred at various timepoints to 4 mL scintillation vials containing 0.500 mL H_2O saturated with Rochelles salt as a quenching agent. Additionally, 1 mL aliquots of reaction solution at various timepoints were transferred to a Delrin Mössbauer cup and freeze-quenched. The water-quenched reaction solutions were filtered through Celite pads and rinsed with DCM. The internal standard 1,3,5-trimethoxybenzene (1.00 mL, 8.85 mM in Et_2O) was added to the filtrate and then placed under vacuum to remove all volatiles. Finally, the remaining solids were dissolved in 1 mL of $CDCl_3$ and analyzed by ^{1}H NMR. 2-allyl-*N*-(2-(1-benzyl-1*H*-1,2,3-triazol-4-yl)propan-2-yl)-6-methyl-benzamide: ^{1}H NMR (400 MHz, $CDCl_3$) δ 7.54 (s, 1H), 7.37 (d, *J* = 6.8 Hz, 3H), 7.27 (d, *J* = 6.2 Hz, 2H), 7.17 (t, *J* = 7.6 Hz, 1H), 7.01 (d, *J* = 7.5 Hz, 2H), 6.30 (s, 1H), 5.94 (ddt, *J* = 12.6, 10.2, 6.4 Hz, 1H), 5.51 (s, 2H), 5.09 – 4.86 (m, 2H), 3.33 (d, *J* = 6.2 Hz, 2H), 2.25 (s, 3H), 1.84 (s, 6H); ^{13}C NMR (400 MHz, $CDCl_3$) δ = 169.1 (C_q), 153.3 (C_q), 137.6 (CH), 137.5 (C_q), 135.9 (C_q), 134.6 (C_q), 134.5 (C_q), 129.2 (CH), 129.1 (CH), 128.9 (CH), 128.6 (CH), 128.0 (CH), 126.9 (CH), 120.7 (CH), 115.8 (CH_2), 54.1 (C_q), 51.8 (CH_2), 37.1 (CH_2), 27.7(CH_3), 19.0 (CH_3).

1.7 Stoichiometric reaction to generate in situ 3c. As an example of the general procedure to generate in situ **3c**, the reaction of $Fe(acac)_3$, dppbz, and H-sub-3 with 3 equivalents of PhMgBr is described. A 20 mL scintillation vial was charged with a mixture of $^{57}Fe(acac)_3$ (0.0154 mg, 0.0435 mmol), dppbz (0.0213 mg, 0.0477 mmol), and H-sub-3 (0.0118 mg, 0.0476 mmol) and then dissolved in THF (15.0 mL). Then a solution of PhMgBr in THF (73 µL, 0.061 mmol, 0.835 M) was added dropwise (0.33 mL/min) via syringe pump to 7.0 mL of the solution of $Fe(acac)_3$, dppe, and H-sub-3 at 65 °C. The solution continued stirring at 65 °C for 1 h and 1.00 mL of reaction solution were removed for ^{57}Fe Mössbauer and LCMS analysis (0.5 mL each) and the remaining solution was cooled to RT.

1.7.1 Reaction of in situ 3c with *N*-chloromorpholine. Following the procedure above, 1.0 equivalent of *N*-chloromorpholine (270 µL, 65.2 mM) was added to the remaining 6.00 mL solution of in situ **3c** at RT. A 500 µL aliquot of reaction solution was freeze-quenched for 80 K ^{57}Fe Mössbauer analysis after 1 min of reaction. For LCMS sample workup, 1 mL aliquots of reaction solution were quenched in 0.3 mL H_2O. The quenched solution was filtered through a Florisil pad and diluted to 2 mM, followed by addition of 1 equivalent of phenanthroline.

1.8 Preparation of *trans*-Fe(dppbz)$_2$(H)(Br). Under a nitrogen atmosphere, a 20 mL scintillation vial was charged with $Fe(acac)_3$ (142.9 mg, 0.4046 mmol), dppbz (183.3 mg, 0.4106 mmol), and H-sub-3 (114.6 mg, 0.4619 mmol), which were then dissolved in THF (8 mL) at RT. Subsequently, PhMgBr (2.195 mL, 2.2 mmol, 1.0 M) was added dropwise to the solution. The resulting solution was then heated to 65 °C and reacted for 1 hour. Then 2 mL of solution were filtered through Celite into a new vial and stored at RT. After 16 days red crystals were observed.

2. Supplementary Data

2.1 Spectroscopic Data for the Allylation System

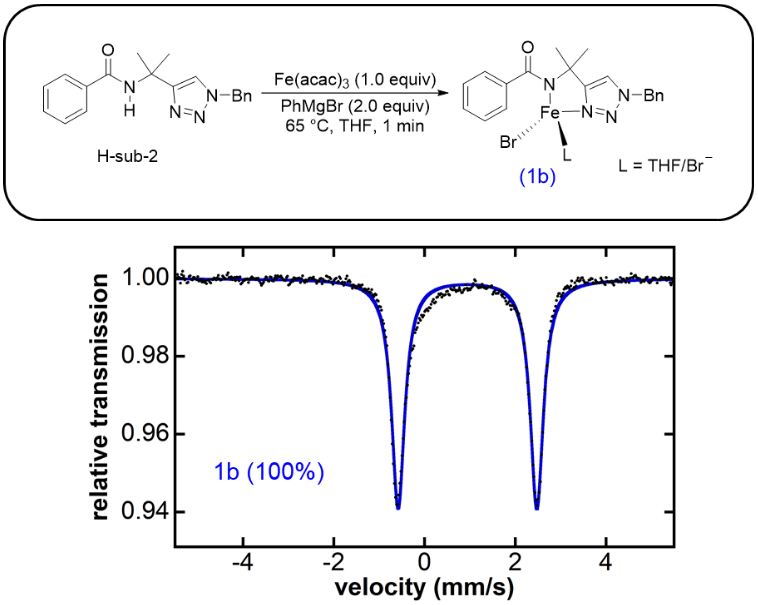

Figure S1. 80 K freeze-quenched Mössbauer spectrum of in situ generated **1b** at 1 minute following the addition of H-sub-2 (1.0 equiv) and PhMgBr (2.0 equiv) to a solution of $Fe(acac)_3$ (1.0 equiv) in THF pre-heated to 65 °C. Data (black dots) and fit component are shown. Blue component **1b** has Mössbauer parameters δ = 0.94 mm/s and ΔE_Q = 3.05 mm/s.

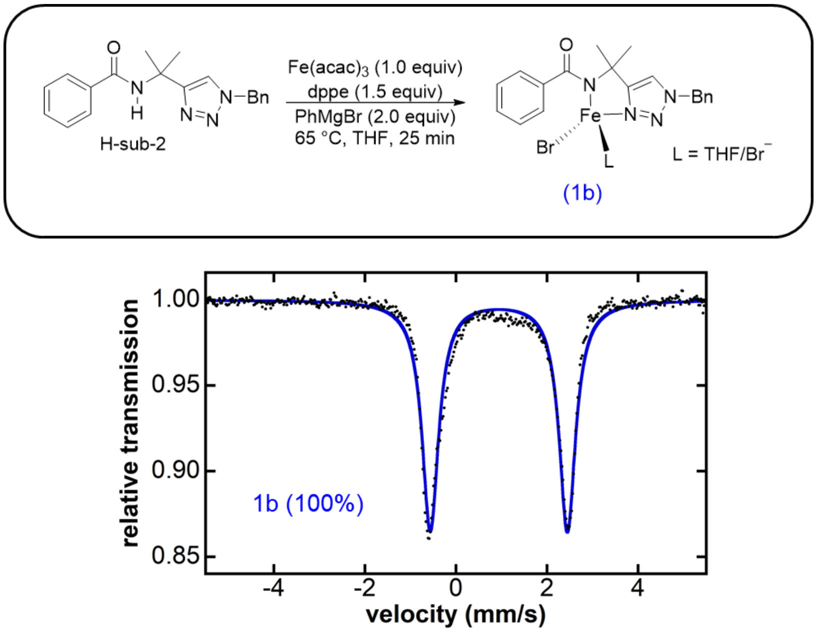

Figure S2. 80 K freeze-quenched Mössbauer spectrum of in situ generated **1b** after 25 minutes stirring at 65 °C following the addition of H-sub-2 (1.0 equiv) and PhMgBr (2.0 equiv) to a solution of $Fe(acac)_3$ (1.0 equiv) and dppe (1.5 equiv) in THF pre-heated to 65 °C. Data (black dots) and fit component are shown. Blue component **1b** has Mössbauer parameters δ = 0.94 mm/s and ΔE_Q = 3.05 mm/s.

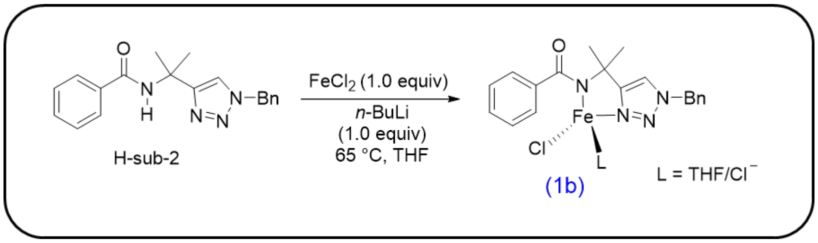

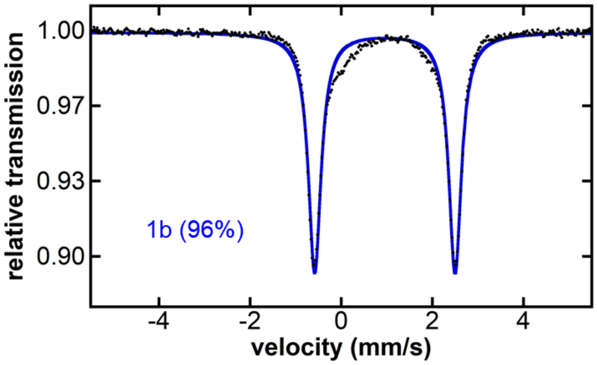

Figure S3. 80 K ^{57}Fe Mössbauer spectrum of freeze-quenched **1b** synthesized by the dropwise addition of a solution of H-sub-2 (1.0 equiv) deprotonated with *n*-BuLi (1.0 equiv) to a pre-heated solution of $FeCl_2$ (1.0 equiv), dppe (1.5 equiv) in THF at 65 °C for 30 min. Data (black dots) and fit component are shown. Blue component **1b** has Mössbauer parameters δ = 0.96 mm/s and ΔE_Q = 3.08 mm/s.

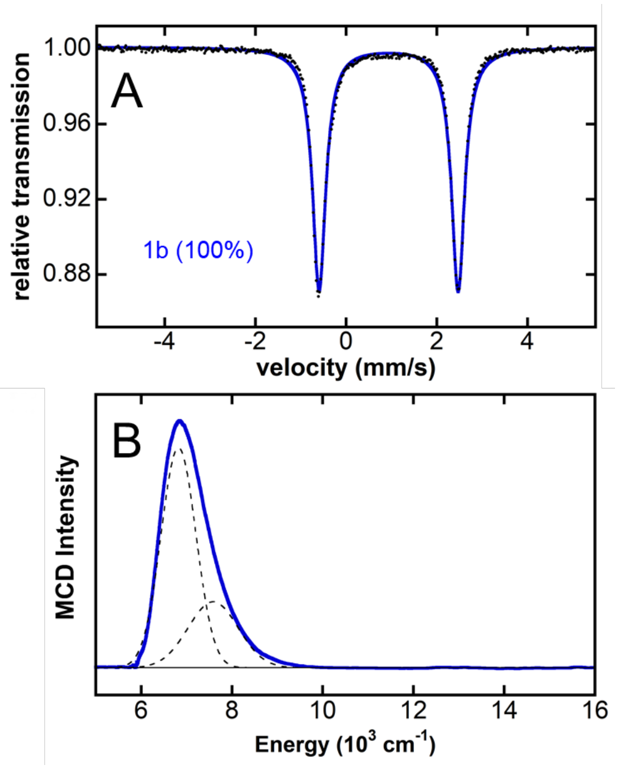

Figure S4. Freeze-quenched (A) 80 K ^{57}Fe Mössbauer spectrum and (B) 5 K, 7 T NIR-MCD spectrum of **1b** generated through the rapid addition of a solution of H-sub-2 (1 equiv) and PhMgBr (2 equiv) to a solution of $Fe(acac)_3$ (1 equiv) in THF at 65 °C and then subsequently cooled to -70 °C and diluted with 2-MeTHF resulting in a 3:2 2-MeTHF:THF solvent mixture. Data (black dots) and fit component are shown. Blue component **1b** has Mössbauer parameters δ = 0.94 mm/s and ΔE_Q = 3.06 mm/s and the corresponding MCD spectrum has fit components (dashed lines) indicating ligand field transitions at ~6750 and ~7450 cm^{-1}.

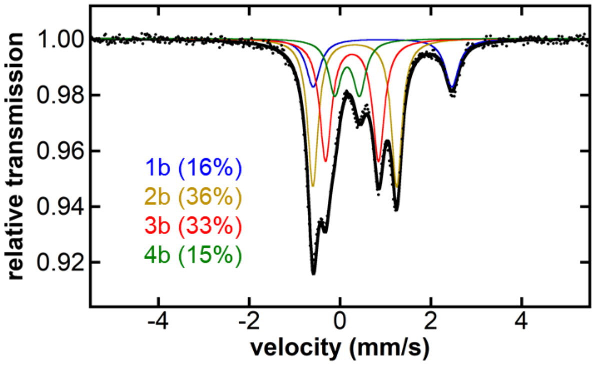

Figure S5. Freeze-trapped 80 K ^{57}Fe Mössbauer spectrum taken at 1 minute following the rapid addition of a solution of H-sub-2 (1 equiv) and PhMgBr (3 equiv) to a solution of $Fe(acac)_3$ (1 equiv) and dppe (1.5 equiv) in THF at 65 °C forming iron species **2b**, **3b**, and **4b**. Data (black dots), total fit (black line), and fit components are shown. Blue component **1b** has Mössbauer parameters δ = 0.94 mm/s and ΔE_Q = 3.05 mm/s; yellow component **2b** has Mössbauer parameters δ = 0.33 mm/s and ΔE_Q = 1.85 mm/s; red component **3b** has Mössbauer parameters δ = 0.27 mm/s and ΔE_Q = 1.17 mm/s; green component **4b** has Mössbauer parameters δ = 0.16 mm/s and ΔE_Q = 0.55 mm/s.

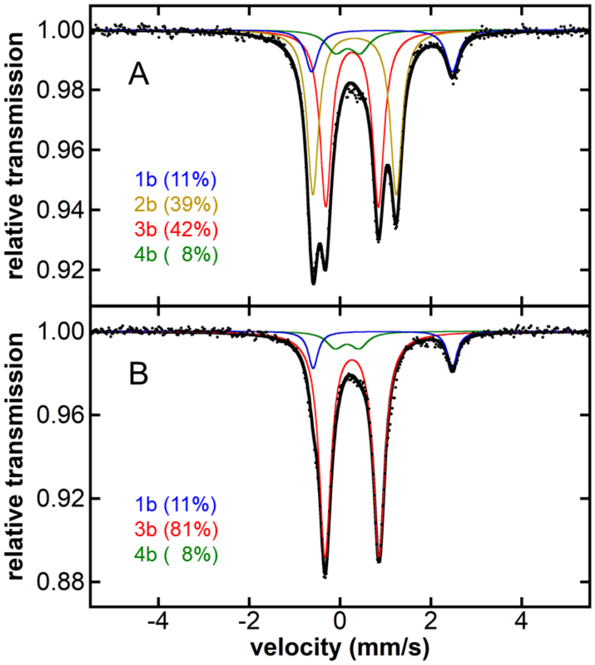

Figure S6. 80 K ^{57}Fe Mössbauer analysis of in situ generated **2b** and **3b** from the reaction of ^{57}Fe(acac)$_3$ (1 equiv) and dppe (1.5 equiv) with PhMgBr (3 equiv) and H-sub-2 (1 equiv) in THF at RT (A), and after removing solvent in vacuo and re-dissolving in 2-MeTHF (B). Data (black dots), total fit (black line), and fit components are shown. Blue component **1b** has Mössbauer parameters δ = 0.93 mm/s and ΔE_Q = 3.10 mm/s; yellow component **2b** has Mössbauer parameters δ = 0.33 mm/s and ΔE_Q = 1.84 mm/s; red component **3b** has Mössbauer parameters δ = 0.27 mm/s and ΔE_Q = 1.16 mm/s; green component **4b** has Mössbauer parameters δ = 0.17 mm/s and ΔE_Q = 0.54 mm/s.

A

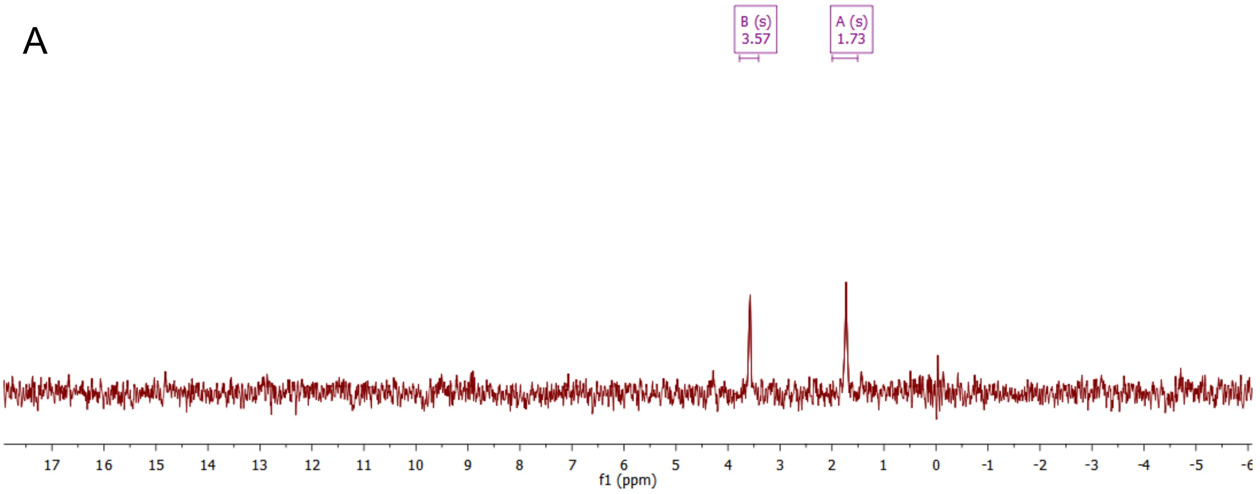

B

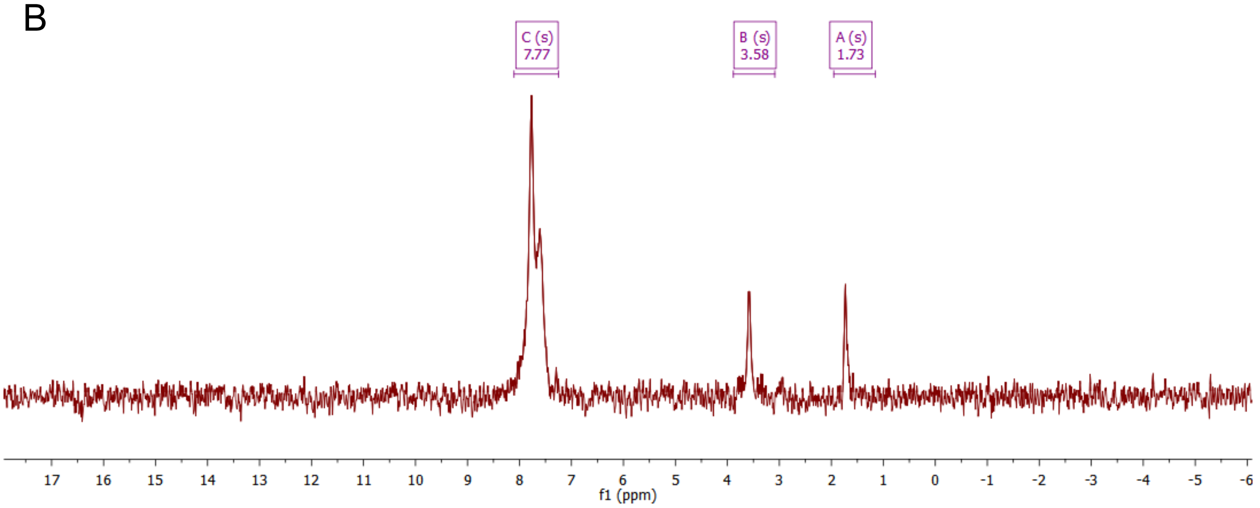

Figure S7. ^{2}H NMR spectrum of (A) in situ generated **2b** and **3b** in THF and (B) in situ generated **2b** and **3b** quenched with D_2O. The signals at 3.58 ppm and 1.73 ppm are consistent with deuterated THF signals as a result of the natural abundance of deuterium. The signal in the aryl region (7.77 ppm) for (B) is consistent with deuterium incorporation into the C-H activated position upon quenching.

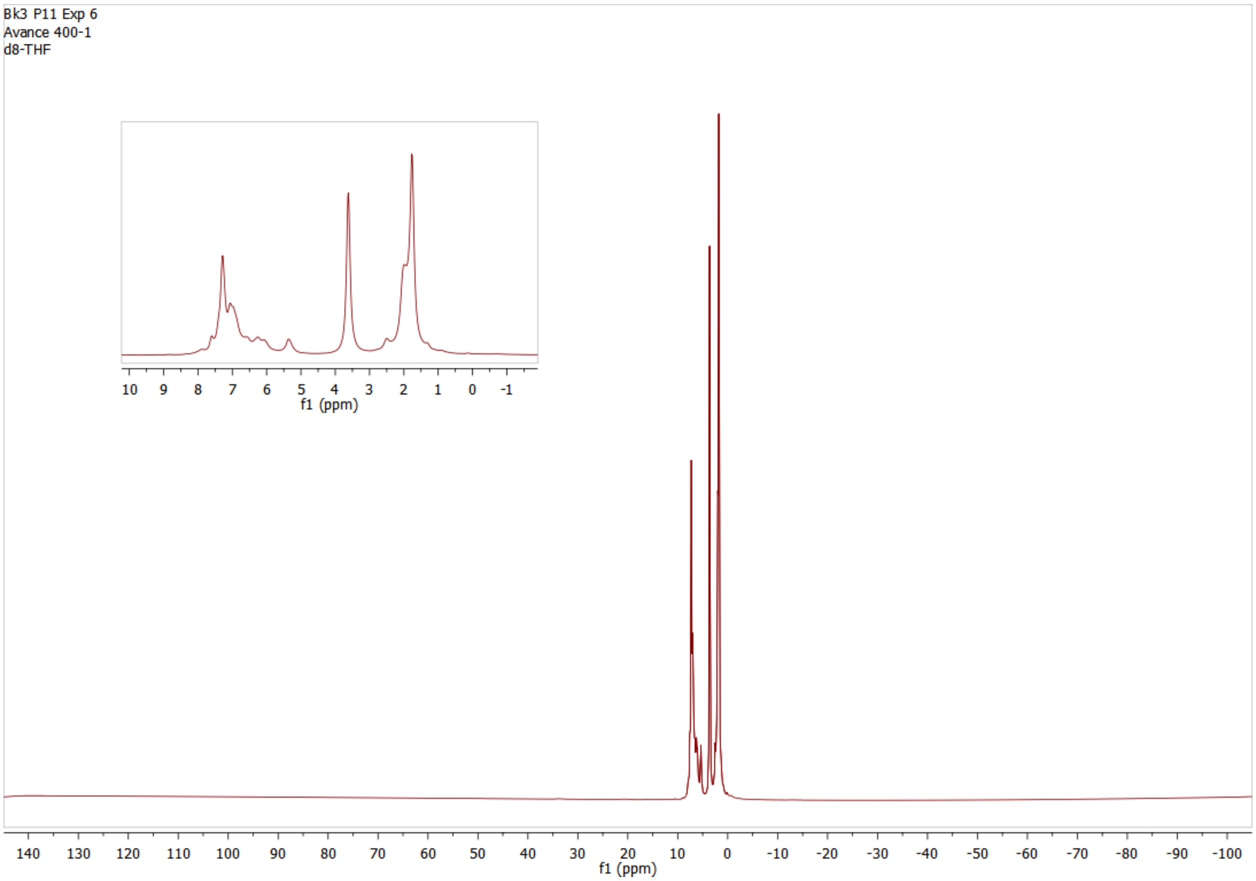

Figure S8. The ^{1}H NMR spectrum of in situ generated **2b** and **3b** in THF-d_8. The spectrum is consistent with the assignment of **2b** and **3b** as low-spin diamagnetic iron(II) species. The Evans method determined solution magnetic moment of this solution is $\mu_{eff} = 0.5\ \mu_B$ resulting from the **1b** impurity (approximately 10 %) present in this mixture. Note that the presence of two major species of low symmetry leads to multiple overlapping protons in the aryl region of the ^{1}H NMR spectrum.

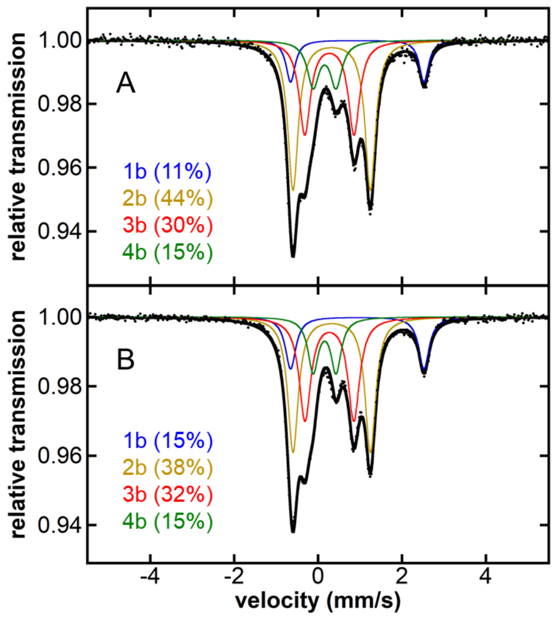

Figure S9. 80 K ^{57}Fe Mössbauer spectra of **2b** and **3b** generated via a 15 minute dropwise addition of a solution of H-sub-2 (1 equiv) and PhMgBr (3 equiv) to a pre-heated solution (65 °C) of Fe(acac)$_3$ (1 equiv) and dppe (1.5 equiv) in THF followed by additional stirring at 65 °C after the addition. Mössbauer samples were freeze-quenched at (A) 1 min and (B) 10 min following the addition of Grignard reagent and H-sub-2. The spectra show minimal decomposition of **2b** and **3b** (~4%) between these timepoints. Data (black dots), total fit (black line), and fit components are shown. Blue component **1b** has Mössbauer parameters δ = 0.94 mm/s and ΔE_Q = 3.18 mm/s; yellow component **2b** has Mössbauer parameters δ = 0.33 mm/s and ΔE_Q = 1.84 mm/s; red component **3b** has Mössbauer parameters δ = 0.27 mm/s and ΔE_Q = 1.17 mm/s; green component **4b** has Mössbauer parameters δ = 0.16 mm/s and ΔE_Q = 0.55 mm/s.

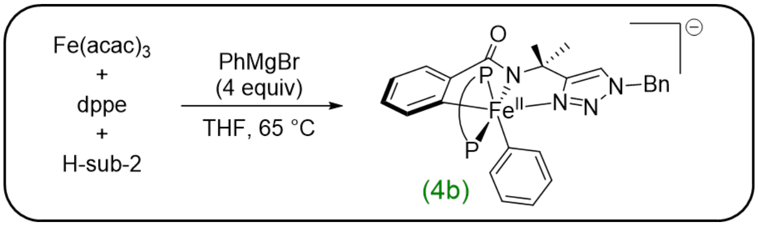

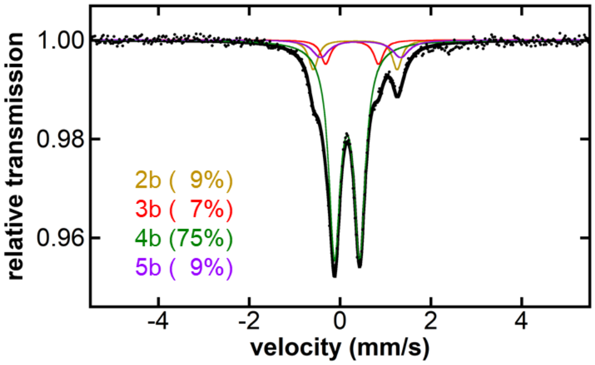

Figure S10. 80 K ^{57}Fe Mössbauer spectrum depicting the generation of in situ Fe-aryl complex **4b** through the dropwise addition of H-sub-2 (1 equiv) and PhMgBr (4 equiv) to a pre-heated solution of Fe(acac)$_3$ (1 equiv) and dppe (1.5 equiv) in THF at 65 °C and then freeze-trapped 10 min following the addition. Data (black dots), total fit (black line), and fit components are shown. Yellow component **2b** has Mössbauer parameters δ = 0.33 mm/s and ΔE_Q = 1.84 mm/s; red component **3b** has Mössbauer parameters δ = 0.27 mm/s and ΔE_Q = 1.17 mm/s; green component **4b** has Mössbauer parameters δ = 0.16 mm/s and ΔE_Q = 0.55 mm/s; purple component **5b** has Mössbauer parameters δ = 0.45 mm/s and ΔE_Q = 1.76 mm/s.

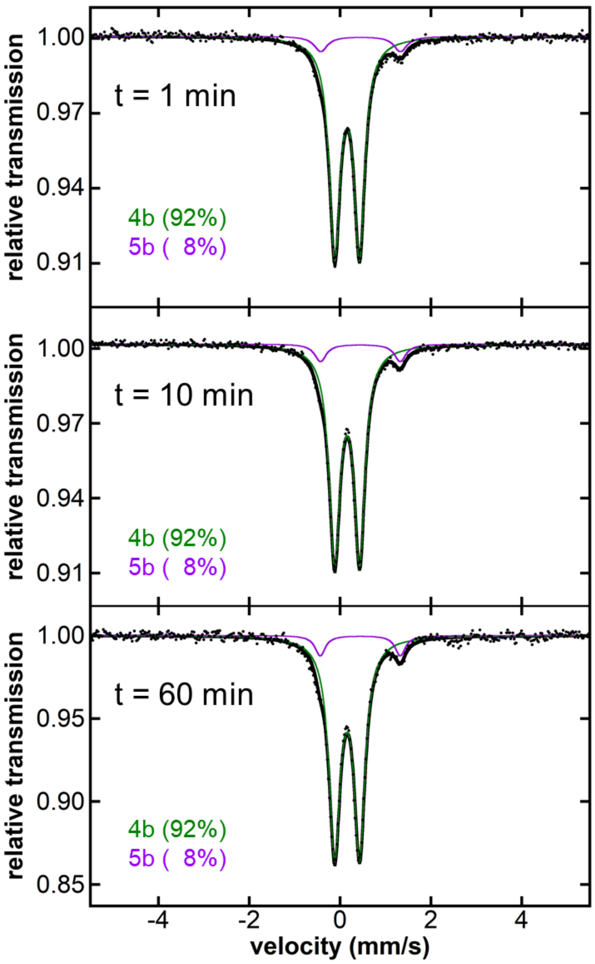

Figure S11. 80 K ^{57}Fe Mössbauer spectra of **4b** following the slow addition of a solution of H-sub-2 (1 equiv) and PhMgBr (4.4 equiv) to a pre-heated solution of $Fe(acac)_3$ (1 equiv) and dppe (1.5 equiv) in THF at 65 °C and allowed to heat at 65 °C after the addition. Samples were freeze-quenched at 1 min, 10 min, and 60 min following the addition of PhMgBr/H-sub-2, with no noticeable change to **4b** concentration highlighting the thermal stability of this complex. Data (black dots), total fit (black line), and fit components are shown. Green component **4b** has Mössbauer parameters δ = 0.16 mm/s and ΔE_Q = 0.55 mm/s; purple component **5b** has Mössbauer parameters δ = 0.45 mm/s and ΔE_Q = 1.76 mm/s.

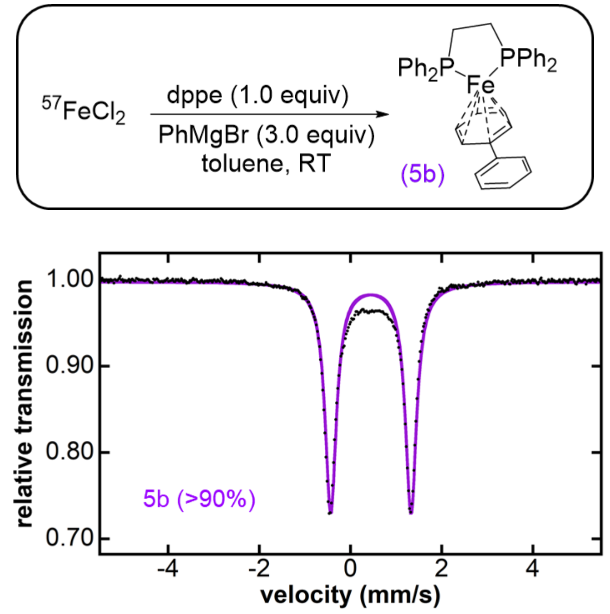

Figure S12. Freeze-trapped 80 K ^{57}Fe Mössbauer spectrum of **5b** generated in situ at room temperature from the reaction of $^{57}FeCl_2$, dppe (1 equiv), and PhMgBr (3 equiv) in toluene. Total Fe concentration is 9.5 mM. Data (black dots) and fit component are shown. Purple component **5b** has Mössbauer parameters δ = 0.45 mm/s and ΔE_Q = 1.76 mm/s.

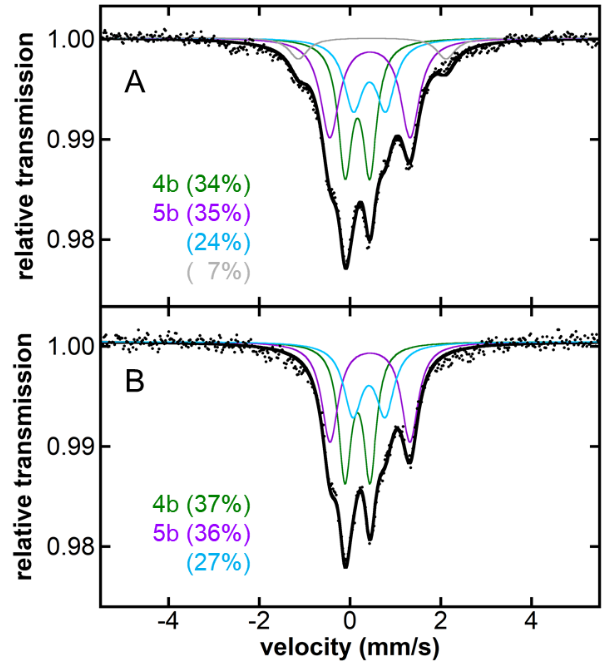

Figure S13. 80 K ^{57}Fe Mössbauer spectra following the reaction of in situ **1b** generated in the presence of dppe (1.5 equiv) with excess PhMgBr (13 equiv) in THF at 65 °C. Samples were freeze-quenched at (A) 30 s, and (B) 60 s following the addition of PhMgBr. Data (black dots), total fit (black line), and fit components are shown. Blue component has Mössbauer parameters δ = 0.42 mm/s and ΔE_Q = 0.71 mm/s.

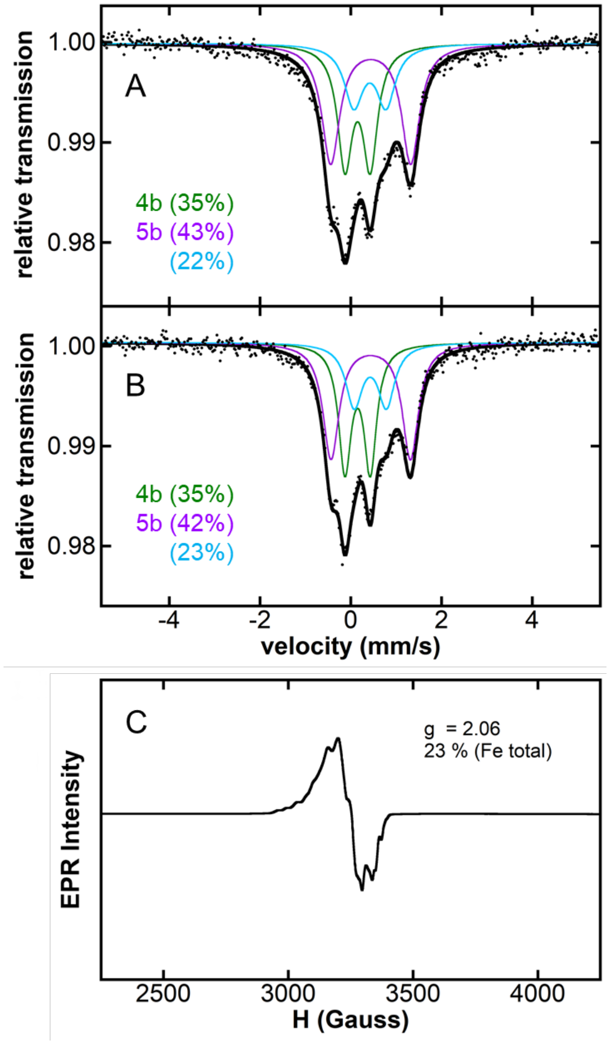

Figure S14. 80 K ^{57}Fe Mössbauer following the addition of a solution of H-sub-2 (1 equiv) and PhMgBr (15 equiv) to a solution of $Fe(acac)_3$ (1 equiv) and dppe (1.5 equiv) in THF at 65 °C. Samples were freeze-quenched at (A) 30 s, and (B) 60 s following the addition of PhMgBr/H-sub-2. Data (black dots), total fit (black line), and fit components are shown. Blue component has Mössbauer parameters δ = 0.42 mm/s and ΔE_Q = 0.71 mm/s. The 10 K EPR spectrum (C) of a sample freeze-quenched at 30 s reveals an EPR active species accounting for 23% of total iron in solution, which suggests that the blue component is an iron(I) species.

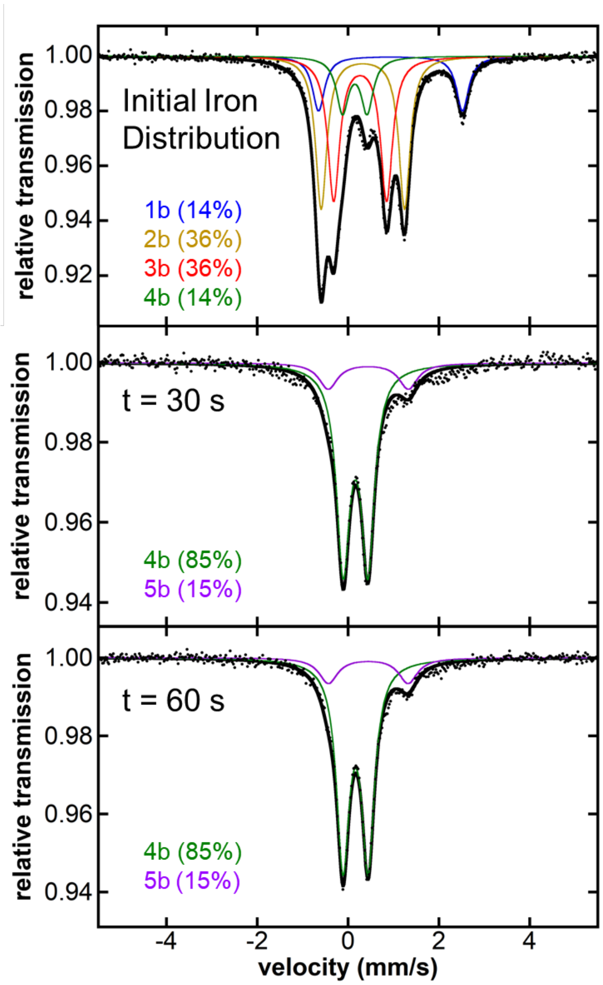

Figure S15. Reaction of in situ generated **2b/3b** with excess PgMgBr (12.0 equiv) at 65 °C monitored by freeze-trapped 80 K ^{57}Fe Mössbauer showing the consumption of **2b/3b** through a putative transmetalation process to generate **4b**. Data (black dots), total fit (black line), and fit components are shown.

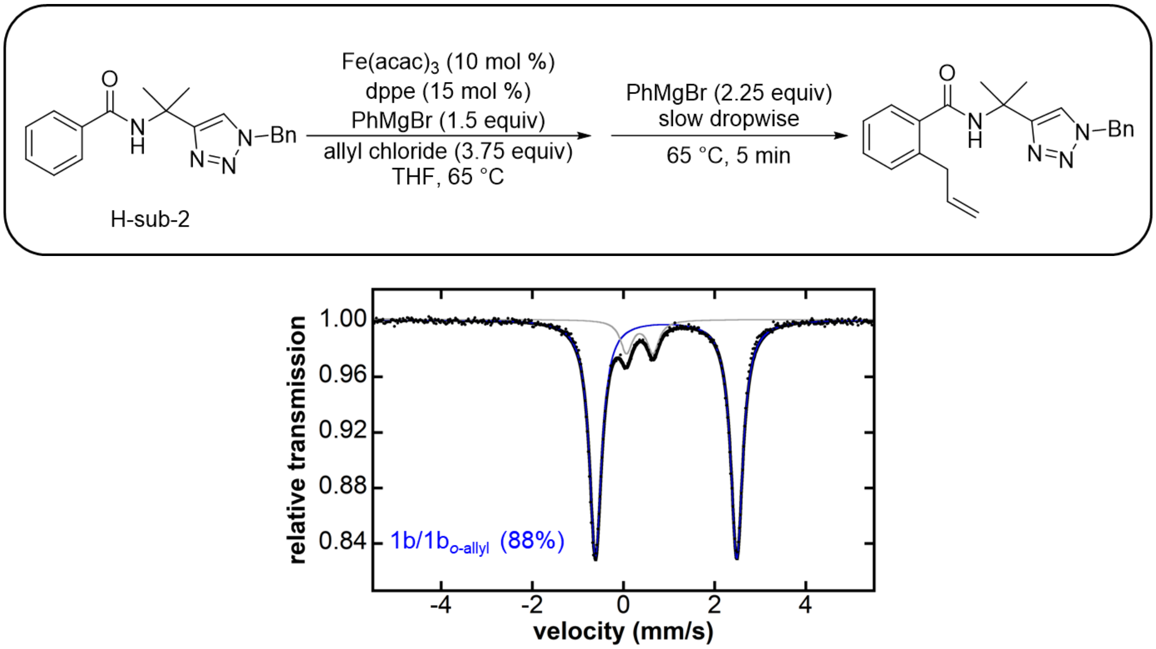

Figure S16. Iron-catalyzed C-H allylation of H-sub-2 with allyl chloride (top). Reaction freeze-quenched at 5 min for iron species analysis using 80 K ^{57}Fe Mössbauer spectroscopy. Data (black dots), total fit (black line), and fit components are shown. Blue component has Mössbauer parameters δ = 0.94 mm/s and ΔE_Q = 3.10 mm/s; gray component has Mössbauer parameters δ = 0.36 mm/s and ΔE_Q = 0.58 mm/s. Both blue and gray species are EPR silent.

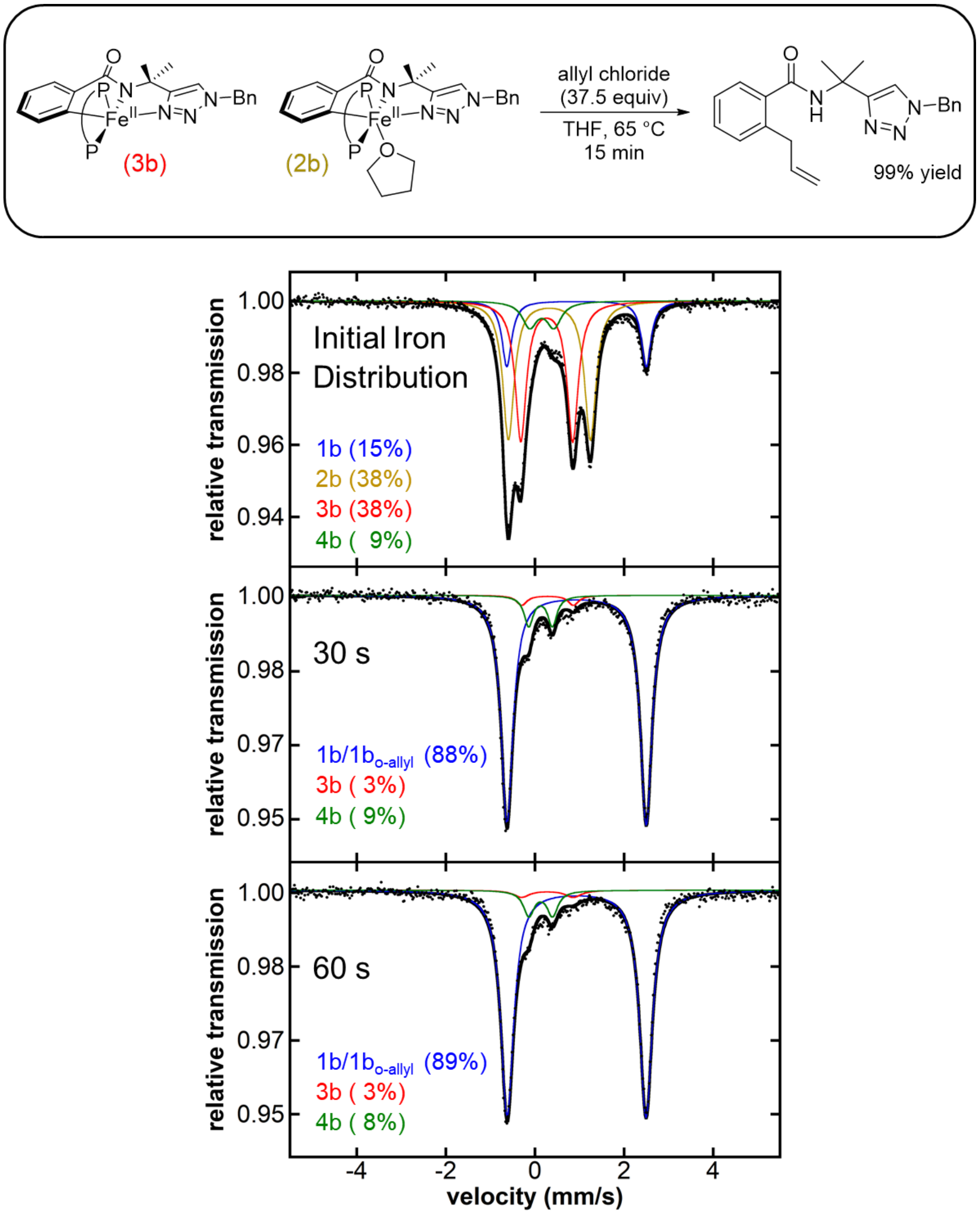

Figure S17. Reaction scheme (top) of in situ generated cyclometalated iron intermediates **2b** and **3b** treated with excess allyl chloride (P-P = dppe). Freeze-quenched 80 K ^{57}Fe Mössbauer spectra (bottom) monitoring the transformation of iron species before and after addition of electrophile (allyl chloride). The product yield was determined by ^{1}H NMR analysis following quenching of the solution in H_2O after 15 min of reaction. The yield is with respect to initial **2b/3b** concentration. Data (black dots), total fit (black line), and fit components are shown. Blue component **1b/1b$_{o\text{-}allyl}$** has Mössbauer parameters δ = 0.94 mm/s and ΔE_Q = 3.12 mm/s.

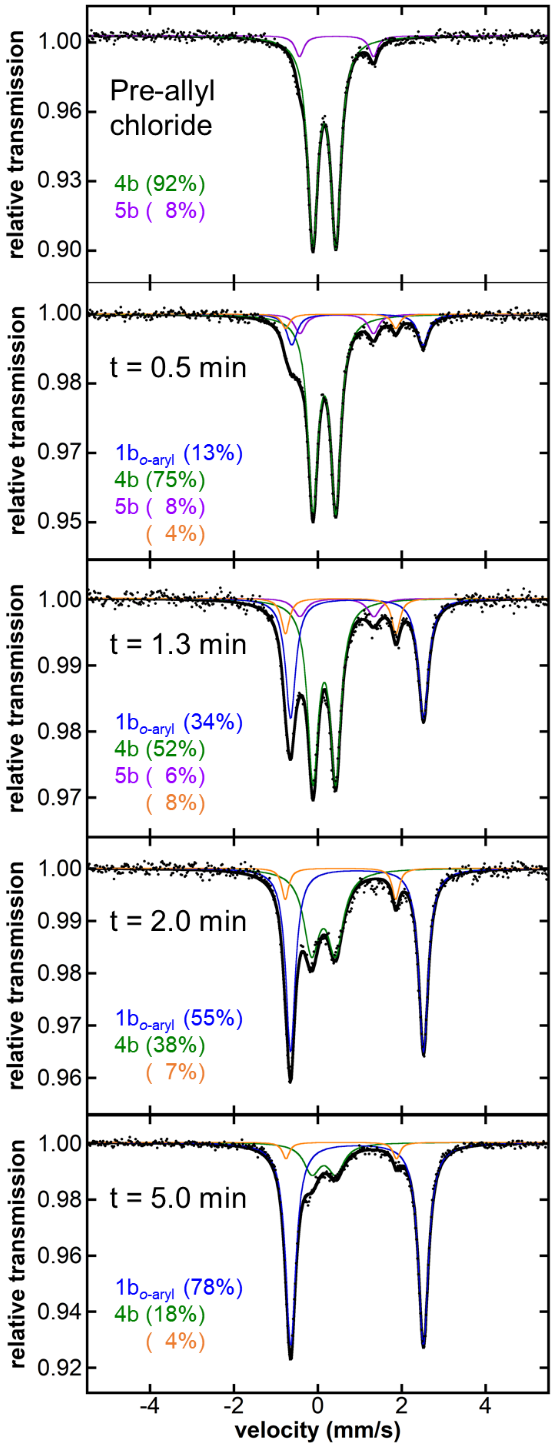

Figure S18. Iron species tracked by 80 K ^{57}Fe Mössbauer spectroscopy during the reaction of in situ **4b** with 37.5 equiv allyl chloride at 65 °C. No EPR active species observed at any time points by 5 K EPR spectroscopy. The minor orange component is assigned to a high-spin iron(II) species.

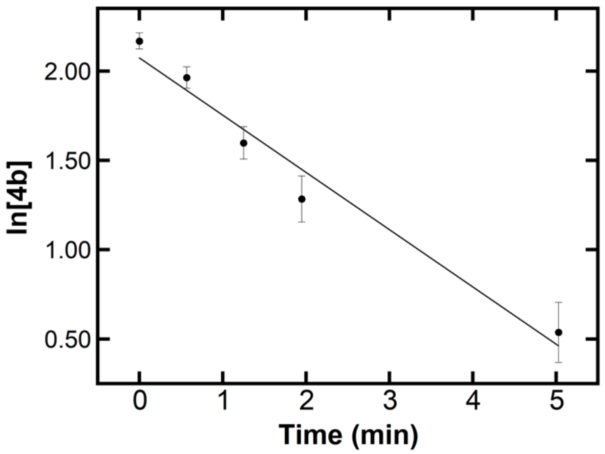

Figure S19. Pseudo-first order kinetic data fit of the consumption of **4b** with 37.5 equivalents of allyl chloride in THF at 65 °C monitored by 80 K ^{57}Fe Mössbauer spectroscopy. The observed rate constant is k = 0.32 ± 0.04 min^{-1}.

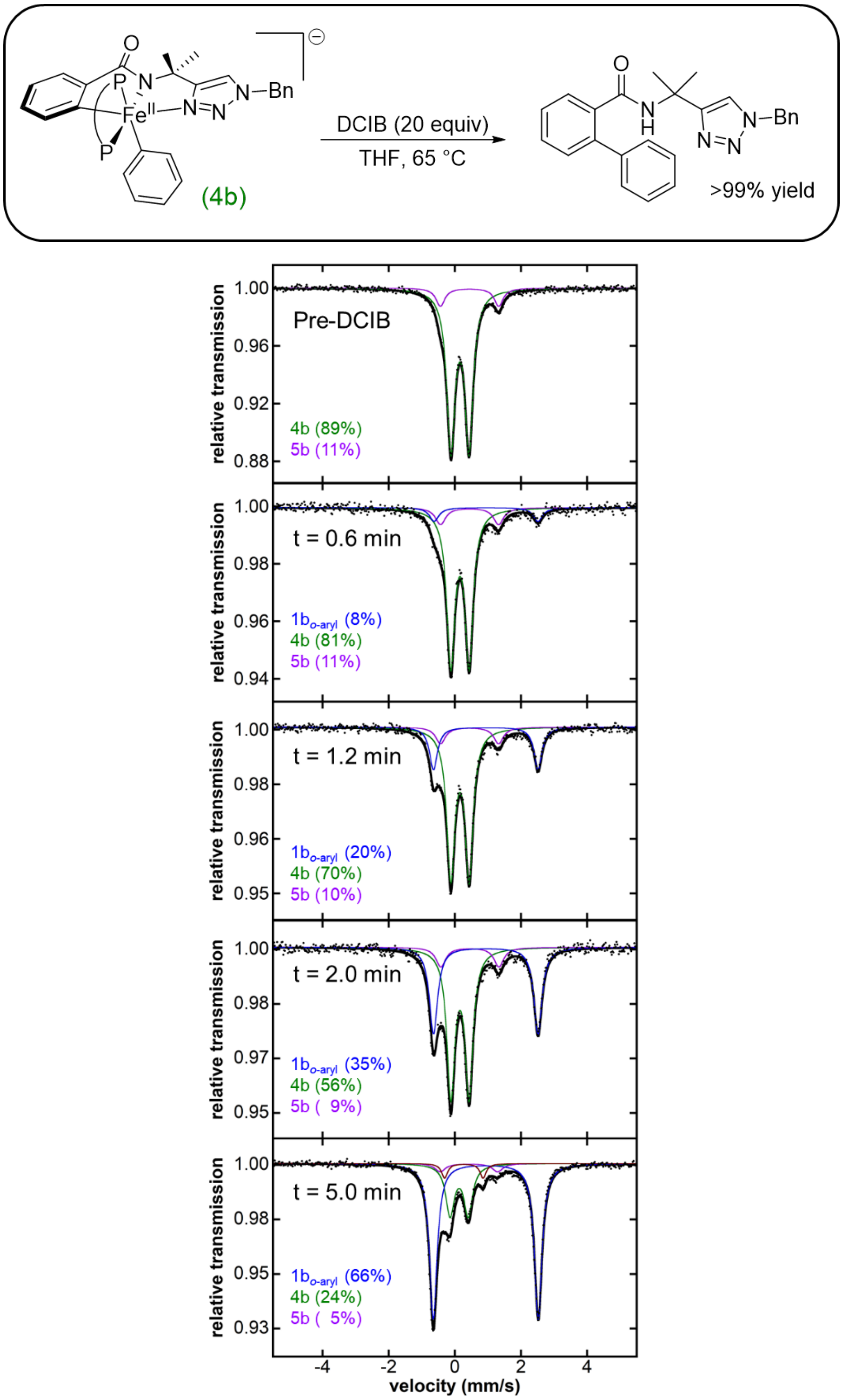

Figure S20. Iron species tracked by 80 K ^{57}Fe Mössbauer spectroscopy during the reaction of in situ **4b** with DCIB (20 equiv with respect to total iron concentration) at 55 °C (P-P = dppe). Product yield determined by ^{1}H NMR and is with respect to initial **4b** concentration. Brown species observed at 5 % of total iron species and is consistent with a slight excess of PhMgBr reacting with **1b$_{o\text{-aryl}}$**.

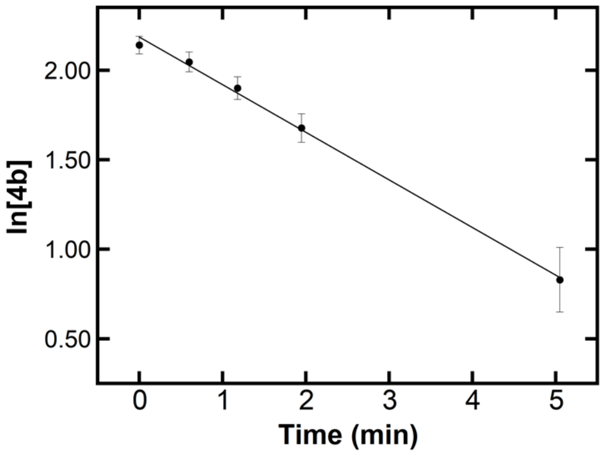

Figure S21. Pseudo-first order kinetic data fit of the consumption of **4b** with 20 equivalents of DCIB in THF at 55 °C monitored by 80K ^{57}Fe Mössbauer spectroscopy. The observed rate constant is k = 0.27 ± 0.04 min^{-1}.

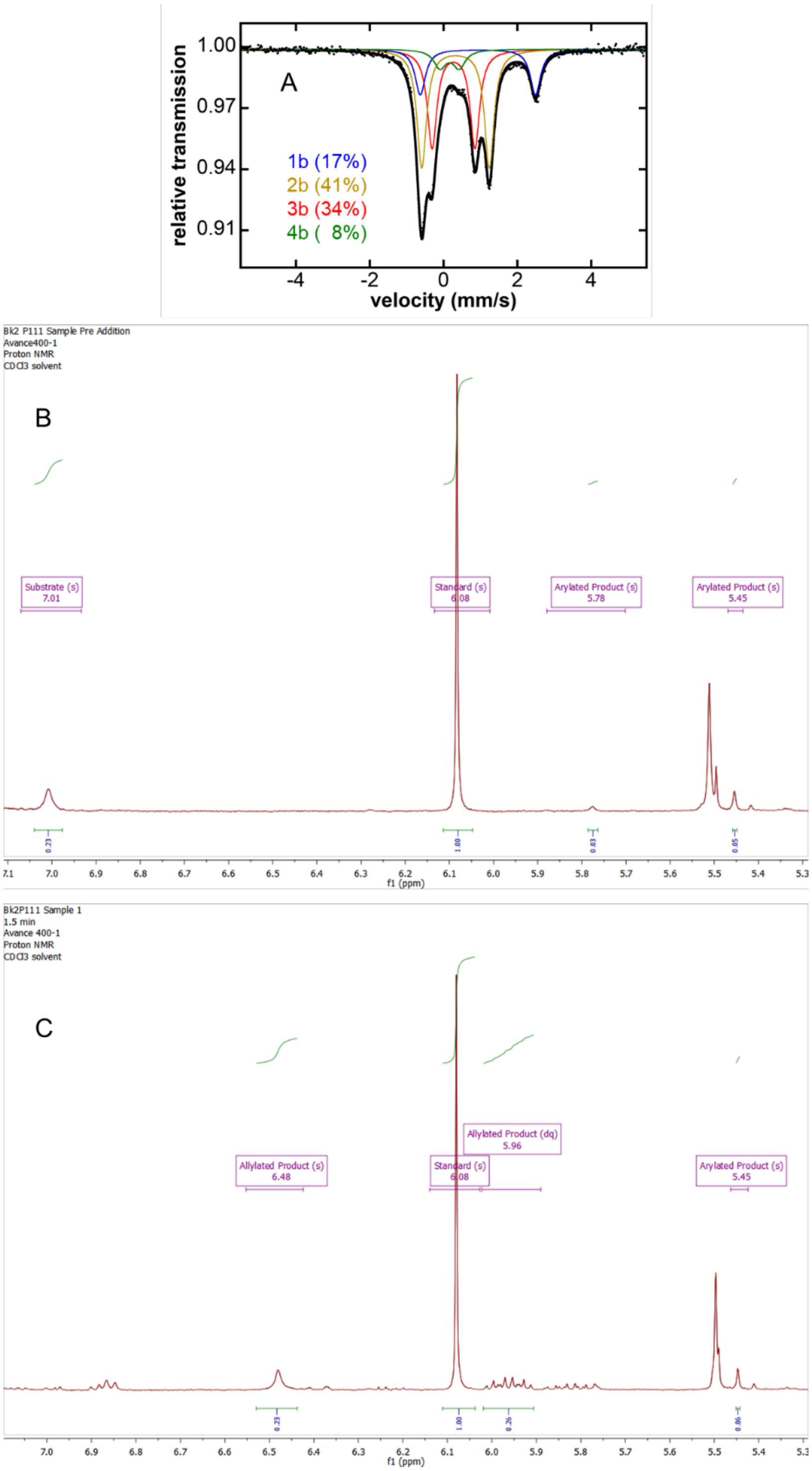

Figure S22. Competition experiment of reaction of **2b**/**3b** with allyl chloride/PhMgBr. (A) 80 K freeze-quenched ^{57}Fe Mössbauer analysis of the pre-reaction solution depicting the initial iron distribution. ^{1}H NMR analysis of (B) the solution quenched before adding the allyl chloride/PhMgBr mixture and (C) 1.5 min after adding the mixture. The yield of allylated product formed is 93% yield with respect to the initial **2b**/**3b** concentration. Additionally, there was an 8% yield of arylated product with respect to the total iron concentration, which resulted from the reductive elimination of **4b** initially present.

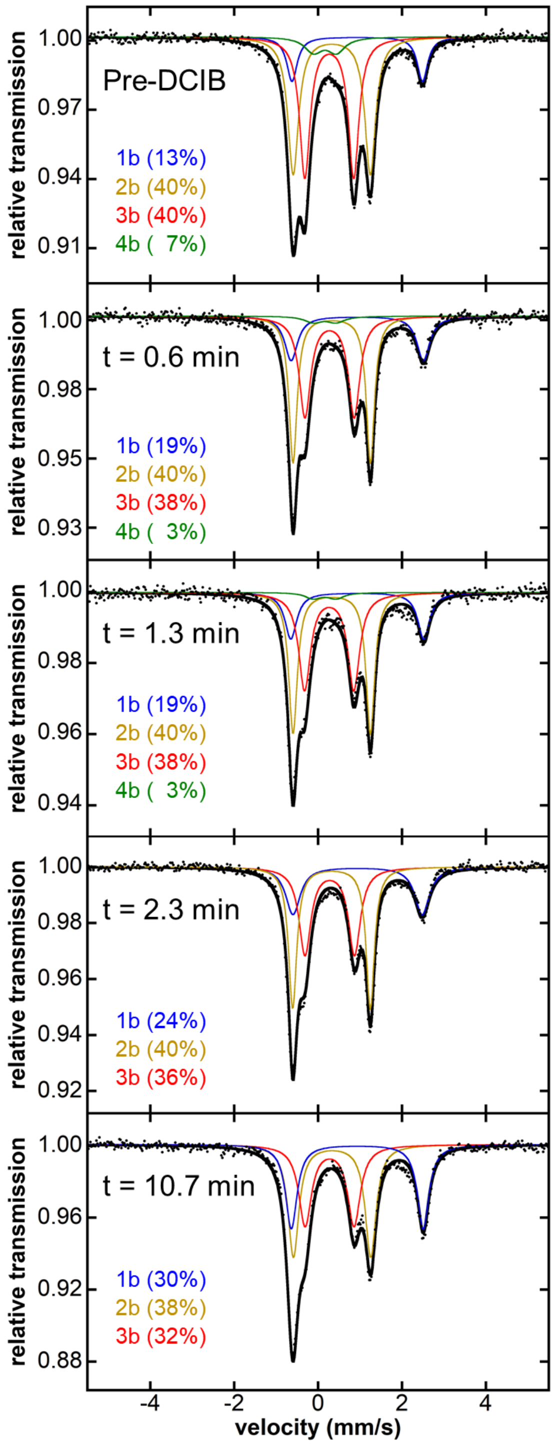

Figure S23. Reaction of in situ generated cyclometalated iron intermediates **2b** and **3b** with excess DCIB. Freeze-quenched 80 K ^{57}Fe Mössbauer spectra monitoring the transformation of iron species before and after addition of oxidant (DCIB). Data (black dots), total fit (black line), and fit components are shown. Following the addition of DCIB, the blue component comprises of a slight amount of **1b$_{o\text{-aryl}}$** as a result of the reaction between the small amount of **4b** initially present and excess DCIB which has been previously shown to form **1b$_{o\text{-aryl}}$**. The remaining increase in **1b** concentration is likely due to decomposition of **2b**/**3b** back to the unactivated intermediate.

2.2 Spectroscopic Data for the Arylation System

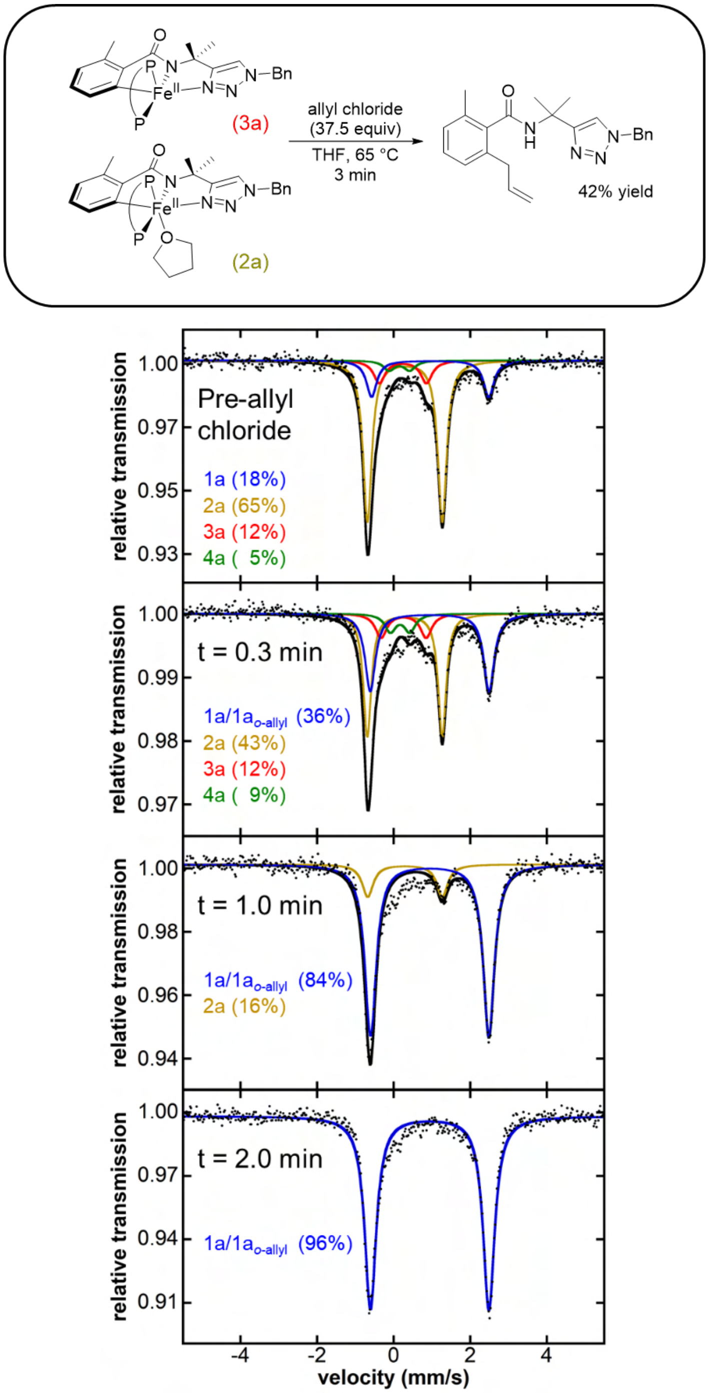

Figure S24. Reaction scheme (top) of in situ generated cyclometalated iron intermediates **2a** and **3a** treated with excess allyl chloride (P-P = dppbz). Freeze-quenched 80 K ^{57}Fe Mössbauer spectra (bottom) monitoring the transformation of iron species before and after addition of electrophile (allyl chloride). The product yield determined by ^{1}H NMR analysis is with respect to initial **2a/3a** concentration. Data (black dots), total fit (black line), and fit components are shown. Blue component **1a/1a$_{o\text{-allyl}}$** has Mössbauer parameters δ = 0.94 mm/s and ΔE_Q = 3.09 mm/s.

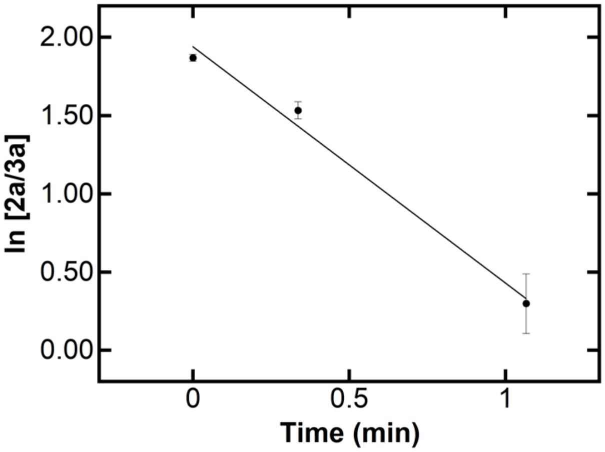

Figure S25. Pseudo-first order kinetic data fit of the consumption of **2a** and **3a** with 37.5 equivalents of allyl chloride in THF at 65 °C monitored by 80 K ^{57}Fe Mössbauer spectroscopy. The observed rate constant is k = 1.5 ± 0.2 min^{-1}.

2.3 Spectroscopic Data for the Amination System

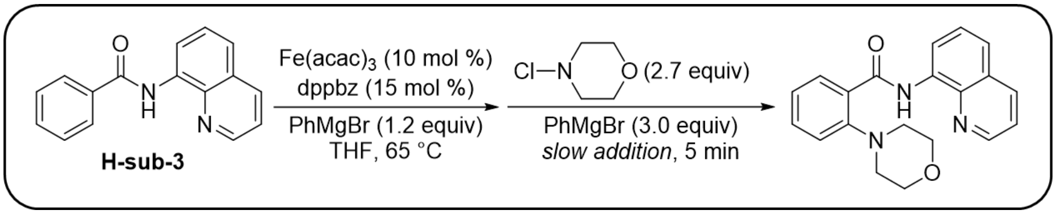

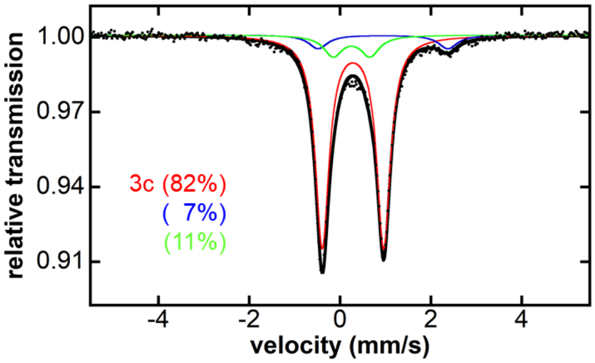

Figure S26. Iron-catalyzed C-H amination of H-sub-3 with *N*-chloromorpholine (NCl) (top). Reaction freeze-quenched at 5 min for iron species analysis using 80 K ^{57}Fe Mössbauer spectroscopy. Data (black dots), total fit (black line), and fit components are shown. Red component has Mössbauer parameters δ = 0.28 mm/s and ΔE_Q = 1.35 mm/s; blue component has Mössbauer parameters δ = 0.95 mm/s and ΔE_Q = 2.86 mm/s; green component has Mössbauer parameters δ = 0.25 mm/s and ΔE_Q = 0.82 mm/s. The red component parameters are consistent with the generation of a cyclometalated low-spin iron(II) species analogous to **3a** and **3b** and has been labeled **3c** accordingly. The blue component parameters are consistent with an iron(II) species similar to **1a** and **1b**. The green component parameters are consistent with the iron(II) species *trans*-$(dppbz)_2Fe(H)(Br)$ (See Figure S29).

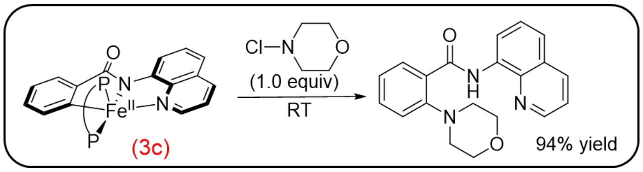

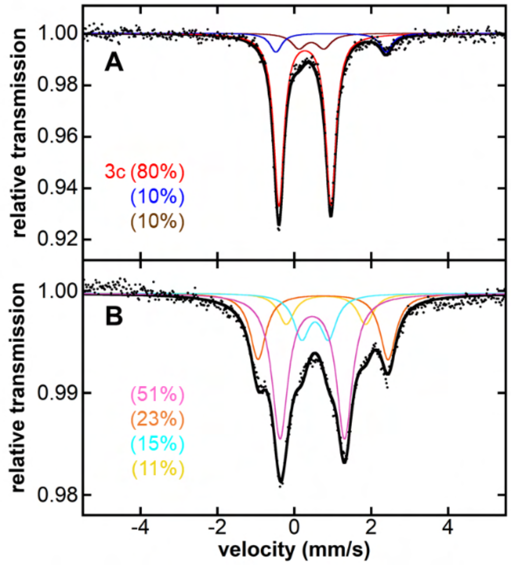

Figure S27. 80 K ^{57}Fe Mössbauer analysis of the reaction of in situ generated **3c** at 3 mM Fe_{total} with 1 equiv NCl at RT (P-P = dppbz). The initial iron distribution is shown (A) as well as a sample freeze-trapped 1 minute following the addition of NCl (B) showing complete consumption of **3c**. Corresponding LCMS confirms the reaction is complete after 1 min with the generation of aminated product with a 94% yield with respect to the initial amount of **3c** (Table S1). Data (black dots), total fit (black line), and fit components are shown. Red component has Mössbauer parameters δ = 0.28 mm/s and ΔE_Q = 1.35 mm/s. Blue component has Mössbauer parameters δ = 0.95 mm/s and ΔE_Q = 2.86 mm/s. Brown component has Mössbauer parameters δ = 0.44 mm/s and ΔE_Q = 0.65 mm/s, and has previously been identified by SCXRD as $Fe(dppbz)_2Br$.[7] The post reaction Mössbauer spectrum (B) depicts one possible fit, although due to the broadness of the data an unambiguous fit could not be established.

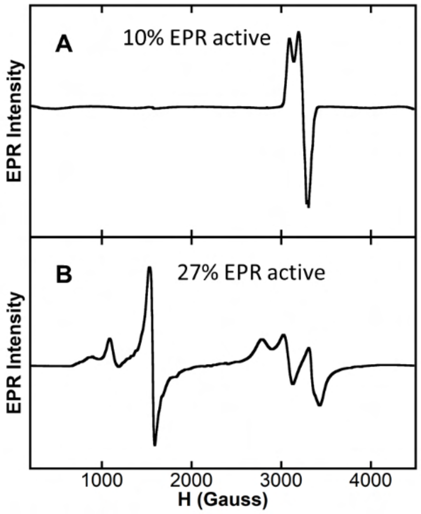

Figure S28. 10 K EPR analysis of the reaction of in situ generated **3c** at 3 mM Fe_{total} with 1 equiv of NCl at RT. The freeze-quenched EPR spectrum prior to the addition of NCl is shown (A), and 1 minute following the addition of NCl (B).

Table S1. LCMS analysis of products generated from the reaction of in situ generated **3c** with NCl (1 equiv) at RT. The reaction was monitored over the course of 10 min. Aliquots of reaction solution were quenched with H_2O and worked up for LCMS analysis to monitor H-sub-3, *ortho*-C-H aminated product (Sub-3-Am, desired product), and *ortho*-C-H phenylated product (Sub-3-Ph, side product). Yields of H-sub-3, Sub-3-Am, and Sub-3-Ph are with respect to the initial H-sub-3 concentration. Yields in parentheses are with respect to the initial **3c** concentration prior to NCl addition (see Figure S27).

Time (min)	H-sub-3 (%)	Sub-3-Am (%)	Sub-3-Ph (%)
**0**	100	0 (0%)	0
**1**	25	75 (94%)	0
**10**	25	75 (94%)	0

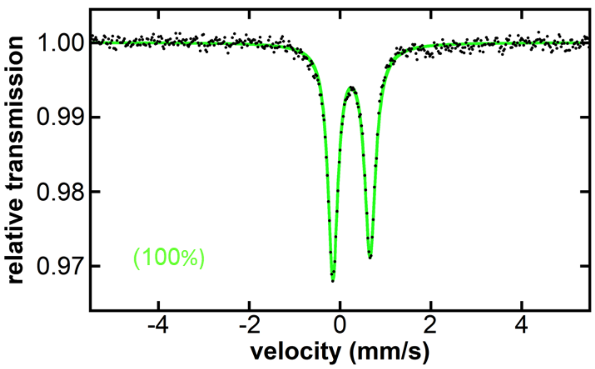

Figure S29. 80 K ^{57}Fe Mössbauer spectrum of *trans*-$(dppbz)_2Fe(H)(Br)$ obtained from purified crystalline material (For crystal structure see section 3). Data (black dots) and fit component are shown. Green component has Mössbauer parameters δ = 0.25 mm/s and ΔE_Q = 0.82 mm/s.

3. Single Crystal X-ray Diffraction Data

3.1 CCDC Deposition. The reported crystal structure has been deposited with the Cambridge Crystallographic Data Center (CCDC). The crystal structure was assigned the following CCDC deposition number:

trans-$(dppbz)_2Fe(H)(Br)$: 2035206

3.2 *trans*-$(dppbz)_2Fe(H)(Br)$

REFERENCE NUMBER: neisc20

CRYSTAL STRUCTURE REPORT

C_{60} H_{49} Br Fe P_4

or

trans-$(dppbz)_2Fe(H)(Br)$

Report prepared for:

S. Carpenter, Prof. M. Neidig

August 08, 2016

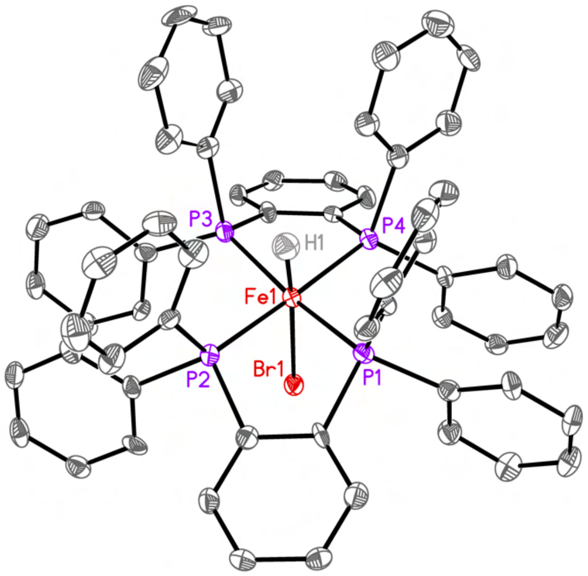

William W. Brennessel
X-ray Crystallographic Facility
Department of Chemistry, University of Rochester
120 Trustee Road
Rochester, NY 14627

Data collection

A crystal (0.28 x 0.14 x 0.10 mm^3) was placed onto the tip of a thin glass optical fiber and mounted on a Bruker SMART APEX II CCD platform diffractometer for a data collection at 100.0(5) K.[1] A preliminary set of cell constants and an orientation matrix were calculated from reflections harvested from three orthogonal wedges of reciprocal space. The full data collection was carried out using MoKα radiation (graphite monochromator) with a frame time of 60 seconds and a detector distance of 3.99 cm. A randomly oriented region of reciprocal space was surveyed: seven major sections of frames were collected with 0.50° steps in ω at seven different ϕ settings and a detector position of -38° in 2θ. The intensity data were corrected for absorption.[2] Final cell constants were calculated from the xyz centroids of 4073 strong reflections from the actual data collection after integration.[3] See Table 1 for additional crystal and refinement information.

Structure solution and refinement

The structure was solved using SHELXT-2014/5[4] and refined using SHELXL-2014/7.[5] The space group *P*-1 was determined based on intensity statistics. A direct-methods solution was calculated which provided most non-hydrogen atoms from the E-map. Full-matrix least squares / difference Fourier cycles were performed which located the remaining non-hydrogen atoms. All non-hydrogen atoms were refined with anisotropic displacement parameters. The hydrido ligand was found from the difference Fourier map and refined freely. All other hydrogen atoms were placed in ideal positions and refined as riding atoms with relative isotropic displacement parameters.

The refinement stalled at $R1$ = 0.122, at which point twin modeling was required. After the non-merohedral twin law, [1 -0.723 -0.290 / 0 1 0 / 0 0 1], a 180 degree rotation about reciprocal lattice [100], was determined,[6] the data were re-integrated,[3] and a new absorption correction was applied.[7] There were 6012 unique reflections solely in the first component, 6019 unique reflections solely in the second component, and 3318 unique overlapping reflections. The mass ratio of the two components refined to 0.523(2):468(2).

The final full matrix least squares refinement converged to $R1 = 0.0682$ (F^2, $I > 2\sigma(I)$) and $wR2 = 0.1467$ (F^2, all data).

Structure description

The structure is the one suggested. The asymmetric unit contains one iron complex in a general position.

Unless noted otherwise all structural diagrams containing thermal displacement ellipsoids are drawn at the 50 % probability level.

Data collection, structure solution, and structure refinement were conducted at the X-ray Crystallographic Facility, B51 Hutchison Hall, Department of Chemistry, University of Rochester. All publications arising from this report

MUST either 1) include William W. Brennessel as a coauthor or 2) acknowledge William W. Brennessel and the X-ray Crystallographic Facility of the Department of Chemistry at the University of Rochester.

---

[1] *APEX3*, version 2015.9-0; Bruker AXS: Madison, WI, 2015.

[2] Sheldrick, G. M. *SADABS*, version 2014/5; *J. Appl. Cryst.* **2015**, *48*, 3-10.

[3] *SAINT*, version 8.34A; Bruker AXS: Madison, WI, 2013.

[4] Sheldrick, G. M. *SHELXT-2014/5*; University of Göttingen: Göttingen, Germany, 2014.

[5] Sheldrick, G. M. *SHELXL-2014/7*; *Acta. Cryst.* **2015**, C*71*, 3-8.

[6] a) Parsons, S.; Gould, B.; Cooper, R.; Farrugia, L. *ROTAX*; University of Edinburgh: Edinburgh, Scotland, 2003; b) Sheldrick, G. M. *CELL_NOW: A program that analyzes a list of reflections to find a cell and orientation matrix despite the presence of several twin domains or other junk*, version 2008/2; University of Göttingen: Göttingen, Germany, 2008.

[7] Sheldrick, G. M. *TWINABS*, version 2012/1; University of Göttingen: Göttingen, Germany, 2012.

Some equations of interest:

$$R_{int} = \Sigma\, |F_o^2 - \langle F_o^2 \rangle| \,/\, \Sigma |F_o^2|$$

$$R1 = \Sigma ||F_o| - |F_c|| \,/\, \Sigma |F_o|$$

$$wR2 = [\Sigma[w(F_o^2 - F_c^2)^2] \,/\, \Sigma\, [w(F_o^2)^2]]^{1/2}$$

$$\text{where } w = 1 \,/\, [\sigma^2 (F_o^2) + (aP)^2 + bP] \text{ and}$$

$$P = 1/3 \max (0, F_o^2) + 2/3\, F_c^2$$

$$\text{GOF} = S = [\Sigma[w(F_o^2 - F_c^2)^2] \,/\, (m-n)]^{1/2}$$

where m = number of reflections and n = number of parameters

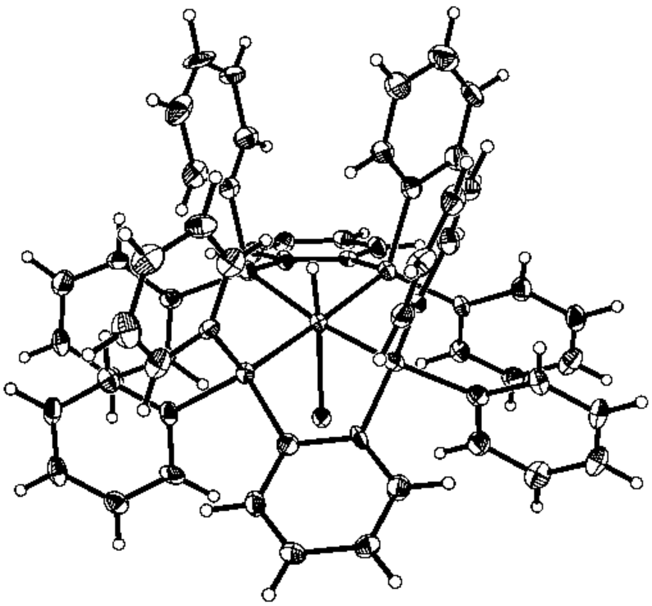

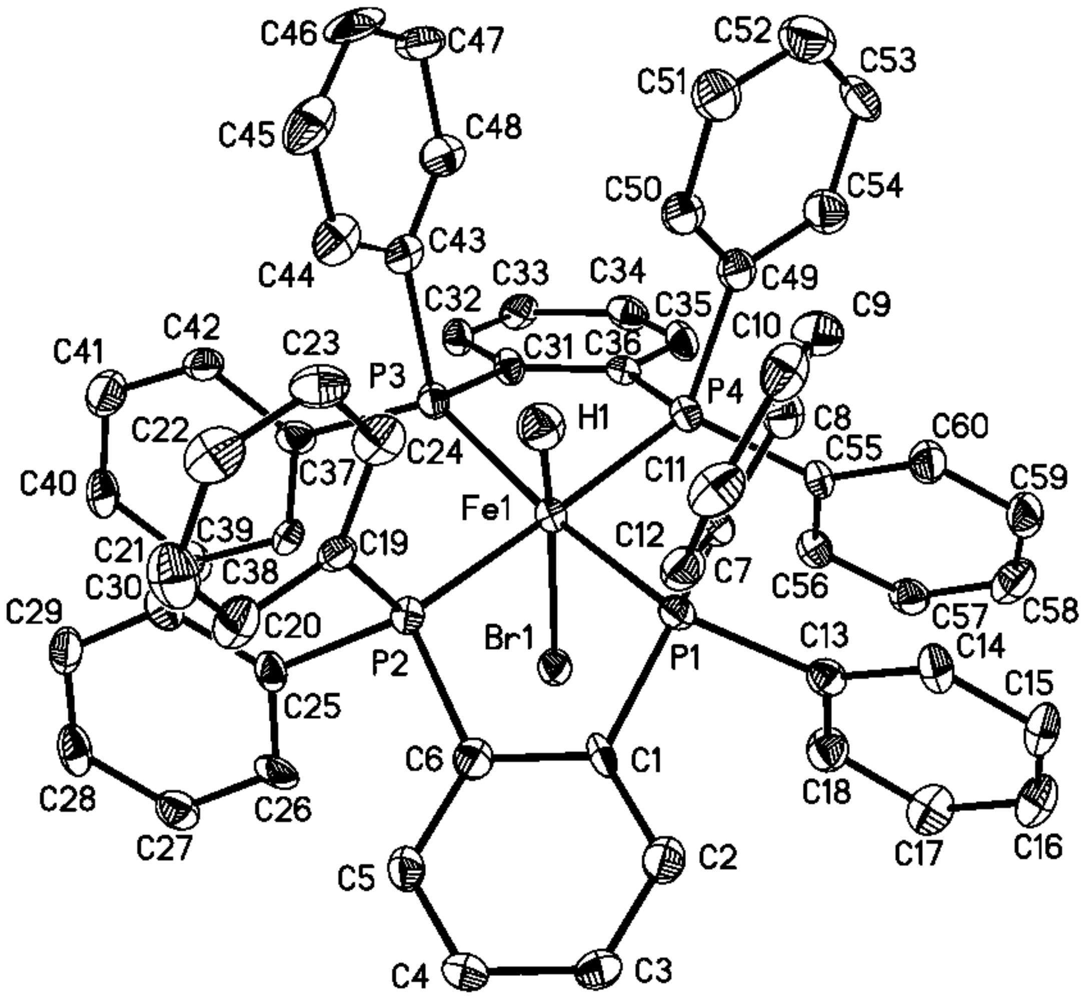
C46
C47
C52
C53
C51
C45
C48
C50
C54
C43
C44
C33
C34
C49
C32
C35
C9
C10
C42
C23
C31
C36
C41
P3
P4
C8
H1
C60
C22
C55
C59
C24
C11
C40
C37
Fe1
C12
C39
C21
C19
C7
C56
C38
C30
C58
C57
C29
C20
C14
C13
Br1
C15
P2
P1
C25
C6
C1
C18
C28
C26
C16
C17
C27
C2
C5
C4
C3

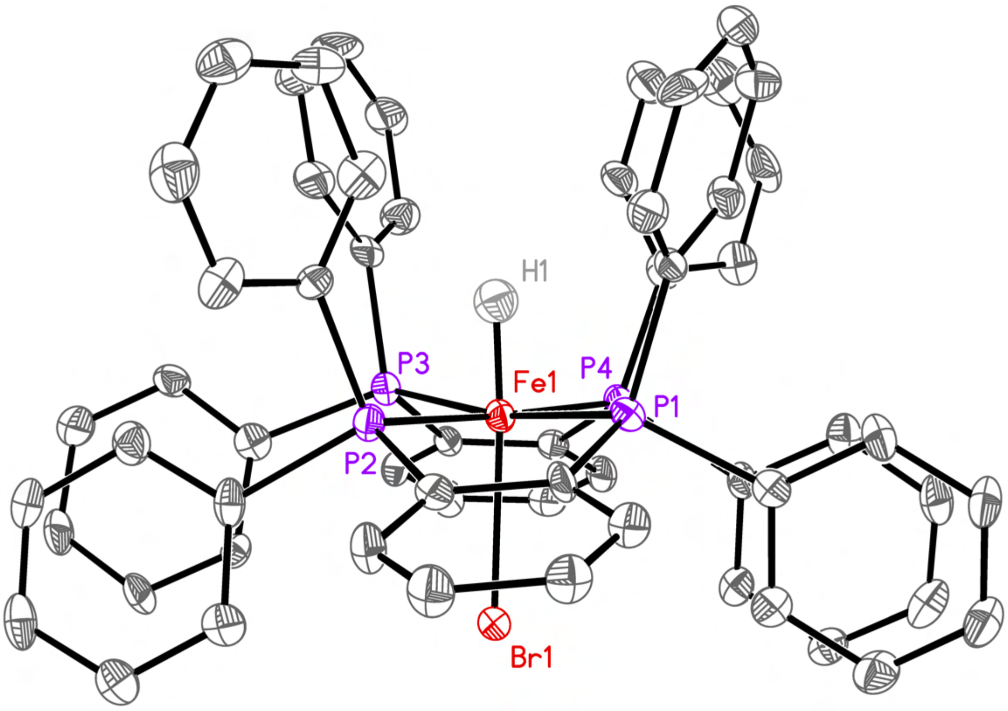
H1
P3
Fe1
P4
P1
P2
Br1

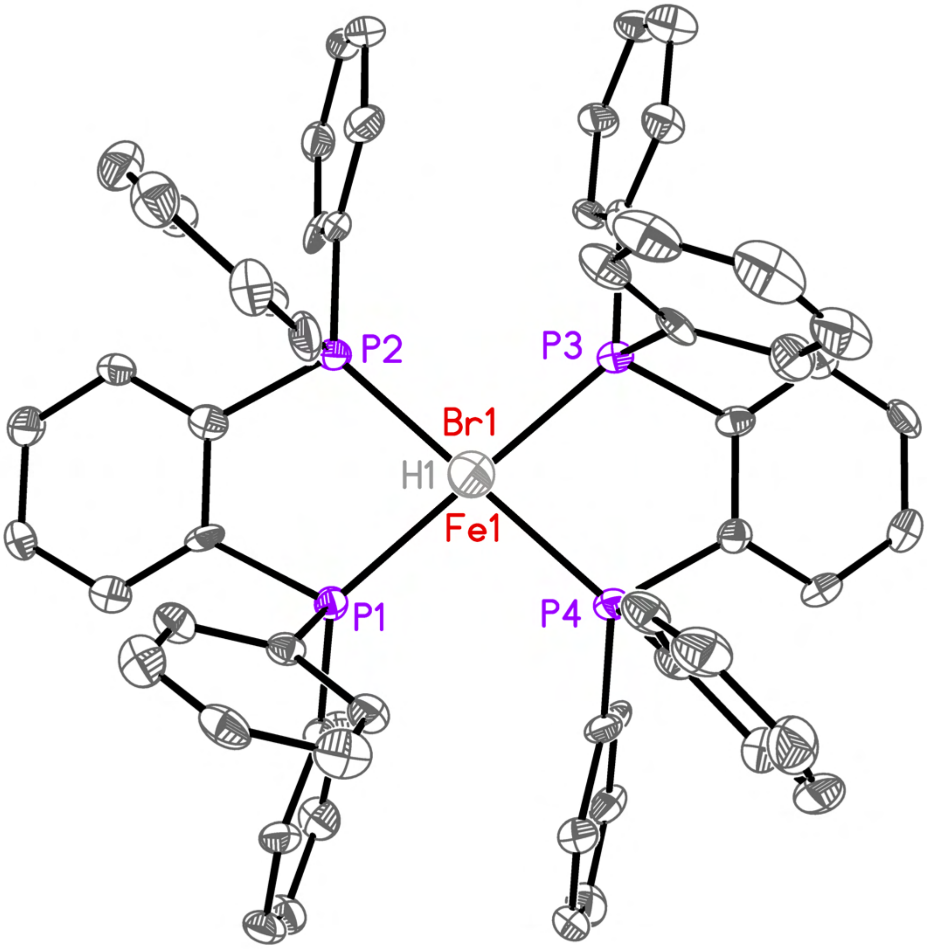
P2
P3
Br1
H1
Fe1
P1
P4

Table S72. Crystal data and structure refinement for neisc20.

Identification code	neisc20	
Empirical formula	C60 H49 Br Fe P4	
Formula weight	1029.63	
Temperature	100.0(5) K	
Wavelength	0.71073 Å	
Crystal system	triclinic	
Space group	*P*-1	
Unit cell dimensions	*a* = 10.8690(12) Å	α = 80.274(2)°
	b = 12.0085(14) Å	β = 76.661(2)°
	c = 20.318(2) Å	γ = 65.621(2)°
Volume	2342.2(5) $Å^3$	
Z	2	
Density (calculated)	1.460 Mg/m^3	
Absorption coefficient	1.353 mm^{-1}	
F(000)	1060	
Crystal colour, morphology	red, plate	
Crystal size	0.28 x 0.14 x 0.10 mm^3	
Theta range for data collection	1.868 to 25.315°	
Index ranges	$-12 \leq h \leq 13, -14 \leq k \leq 14, 0 \leq l \leq 24$	
Reflections collected	74820	
Independent reflections	8494 [*R*(int) = 0.1827]	
Observed reflections	5584	
Completeness to theta = 25.028°	100.0%	
Absorption correction	Multi-scan	
Max. and min. transmission	0.7452 and 0.6336	
Refinement method	Full-matrix least-squares on F^2	
Data / restraints / parameters	8494 / 0 / 600	
Goodness-of-fit on F^2	1.038	
Final *R* indices [*I*>2sigma(*I*)]	*R*1 = 0.0682, *wR*2 = 0.1258	
R indices (all data)	*R*1 = 0.1209, *wR*2 = 0.1467	
Largest diff. peak and hole	0.868 and -0.643 e.$Å^{-3}$	

Table S73. Atomic coordinates (x 10^4) and equivalent isotropic displacement parameters ($Å^2$x 10^3) for neisc20. U_{eq} is defined as one third of the trace of the orthogonalized U_{ij} tensor.

	x	y	z	U_{eq}
Fe1	6796(1)	2845(1)	7368(1)	16(1)
Br1	4547(1)	3716(1)	6949(1)	20(1)
P1	5896(2)	3508(2)	8402(1)	16(1)
P2	6985(2)	4647(2)	7122(1)	17(1)
P3	8024(2)	2036(2)	6395(1)	17(1)
P4	6701(2)	1006(2)	7628(1)	18(1)
C1	5224(7)	5186(6)	8332(3)	18(2)
C2	4328(7)	5925(6)	8842(4)	24(2)
C3	3903(7)	7183(6)	8761(4)	22(2)
C4	4352(7)	7718(6)	8135(4)	25(2)
C5	5201(7)	7016(6)	7625(4)	23(2)
C6	5681(7)	5727(6)	7715(3)	19(2)
C7	7150(7)	3087(6)	8964(3)	16(2)
C8	7820(7)	1850(6)	9154(3)	22(2)
C9	8821(7)	1450(7)	9559(4)	30(2)
C10	9129(7)	2308(7)	9784(3)	25(2)
C11	8491(7)	3510(7)	9611(4)	26(2)
C12	7500(7)	3914(7)	9200(3)	22(2)
C13	4446(7)	3302(6)	9007(3)	18(2)
C14	4432(8)	2985(6)	9700(4)	23(2)
C15	3271(7)	2960(7)	10135(4)	26(2)
C16	2069(8)	3286(7)	9894(4)	28(2)
C17	2054(8)	3625(7)	9217(4)	27(2)
C18	3235(7)	3625(6)	8766(4)	25(2)
C19	8504(7)	4826(6)	7286(3)	18(2)
C20	8612(8)	5965(7)	7130(4)	27(2)
C21	9727(8)	6119(7)	7245(4)	34(2)
C22	10749(8)	5158(7)	7524(4)	30(2)
C23	10650(7)	4028(7)	7685(4)	26(2)
C24	9518(8)	3872(7)	7575(4)	25(2)
C25	6789(7)	5562(6)	6295(3)	20(2)

C26	5488(7)	6265(6)	6134(4)	22(2)
C27	5370(8)	6964(6)	5519(4)	24(2)
C28	6502(8)	6927(6)	5046(4)	25(2)
C29	7783(8)	6222(6)	5195(4)	25(2)
C30	7938(8)	5519(6)	5817(4)	24(2)
C31	7411(7)	890(6)	6248(3)	17(2)
C32	7569(7)	503(6)	5614(3)	19(2)
C33	7129(7)	-405(6)	5570(4)	24(2)
C34	6555(7)	-932(6)	6153(4)	23(2)
C35	6407(7)	-543(6)	6784(4)	22(2)
C36	6820(7)	386(6)	6831(3)	18(2)
C37	8101(7)	2835(6)	5546(4)	20(2)
C38	6869(7)	3605(6)	5330(3)	17(2)
C39	6884(8)	4163(7)	4687(3)	24(2)
C40	8096(8)	3991(7)	4235(4)	27(2)
C41	9327(8)	3211(7)	4436(4)	30(2)
C42	9329(7)	2638(6)	5096(4)	22(2)
C43	9849(7)	1099(6)	6408(3)	18(2)
C44	10687(8)	1665(7)	6482(3)	27(2)
C45	12060(8)	1014(8)	6496(4)	36(2)
C46	12622(8)	-238(8)	6450(4)	41(2)
C47	11822(8)	-821(8)	6374(4)	36(2)
C48	10439(7)	-152(7)	6343(4)	27(2)
C49	8123(7)	-267(6)	7978(4)	22(2)
C50	9302(7)	-129(7)	8008(3)	24(2)
C51	10414(8)	-1085(7)	8238(4)	34(2)
C52	10325(9)	-2206(7)	8446(4)	38(2)
C53	9165(8)	-2367(7)	8440(4)	33(2)
C54	8049(8)	-1413(6)	8202(4)	28(2)
C55	5175(7)	799(6)	8147(3)	19(2)
C56	4008(7)	1112(6)	7869(4)	20(2)
C57	2846(7)	973(6)	8260(4)	24(2)
C58	2818(8)	546(7)	8938(4)	32(2)
C59	3943(8)	250(6)	9225(4)	27(2)
C60	5137(8)	377(6)	8845(4)	24(2)

Table S74. Bond lengths [Å] and angles [°] for neisc20.

Fe(1)-P(2)	2.221(2)	C(10)-C(11)	1.342(10)
Fe(1)-P(4)	2.222(2)	C(10)-H(10)	0.9500
Fe(1)-P(1)	2.231(2)	C(11)-C(12)	1.393(9)
Fe(1)-P(3)	2.234(2)	C(11)-H(11)	0.9500
Fe(1)-Br(1)	2.5183(11)	C(12)-H(12)	0.9500
Fe(1)-H(1)	1.38(6)	C(13)-C(18)	1.391(9)
P(1)-C(1)	1.830(7)	C(13)-C(14)	1.393(9)
P(1)-C(7)	1.837(7)	C(14)-C(15)	1.372(9)
P(1)-C(13)	1.839(7)	C(14)-H(14)	0.9500
P(2)-C(6)	1.833(7)	C(15)-C(16)	1.381(10)
P(2)-C(25)	1.851(7)	C(15)-H(15)	0.9500
P(2)-C(19)	1.858(7)	C(16)-C(17)	1.369(10)
P(3)-C(37)	1.829(7)	C(16)-H(16)	0.9500
P(3)-C(43)	1.836(7)	C(17)-C(18)	1.395(10)
P(3)-C(31)	1.848(7)	C(17)-H(17)	0.9500
P(4)-C(55)	1.836(7)	C(18)-H(18)	0.9500
P(4)-C(36)	1.850(7)	C(19)-C(24)	1.379(9)
P(4)-C(49)	1.850(7)	C(19)-C(20)	1.397(9)
C(1)-C(2)	1.393(10)	C(20)-C(21)	1.374(10)
C(1)-C(6)	1.395(9)	C(20)-H(20)	0.9500
C(2)-C(3)	1.377(9)	C(21)-C(22)	1.377(10)
C(2)-H(2)	0.9500	C(21)-H(21)	0.9500
C(3)-C(4)	1.402(9)	C(22)-C(23)	1.384(10)
C(3)-H(3)	0.9500	C(22)-H(22)	0.9500
C(4)-C(5)	1.360(9)	C(23)-C(24)	1.389(10)
C(4)-H(4)	0.9500	C(23)-H(23)	0.9500
C(5)-C(6)	1.408(9)	C(24)-H(24)	0.9500
C(5)-H(5)	0.9500	C(25)-C(30)	1.382(9)
C(7)-C(12)	1.384(9)	C(25)-C(26)	1.398(9)
C(7)-C(8)	1.389(9)	C(26)-C(27)	1.383(9)
C(8)-C(9)	1.393(10)	C(26)-H(26)	0.9500
C(8)-H(8)	0.9500	C(27)-C(28)	1.366(10)
C(9)-C(10)	1.378(10)	C(27)-H(27)	0.9500
C(9)-H(9)	0.9500	C(28)-C(29)	1.372(10)

C(28)-H(28)	0.9500	C(49)-C(50)	1.374(10)
C(29)-C(30)	1.400(10)	C(49)-C(54)	1.402(9)
C(29)-H(29)	0.9500	C(50)-C(51)	1.390(10)
C(30)-H(30)	0.9500	C(50)-H(50)	0.9500
C(31)-C(36)	1.383(9)	C(51)-C(52)	1.377(11)
C(31)-C(32)	1.392(9)	C(51)-H(51)	0.9500
C(32)-C(33)	1.382(9)	C(52)-C(53)	1.355(11)
C(32)-H(32)	0.9500	C(52)-H(52)	0.9500
C(33)-C(34)	1.387(9)	C(53)-C(54)	1.398(10)
C(33)-H(33)	0.9500	C(53)-H(53)	0.9500
C(34)-C(35)	1.392(10)	C(54)-H(54)	0.9500
C(34)-H(34)	0.9500	C(55)-C(56)	1.391(9)
C(35)-C(36)	1.387(9)	C(55)-C(60)	1.421(9)
C(35)-H(35)	0.9500	C(56)-C(57)	1.385(9)
C(37)-C(42)	1.387(10)	C(56)-H(56)	0.9500
C(37)-C(38)	1.397(9)	C(57)-C(58)	1.385(10)
C(38)-C(39)	1.364(9)	C(57)-H(57)	0.9500
C(38)-H(38)	0.9500	C(58)-C(59)	1.365(10)
C(39)-C(40)	1.380(10)	C(58)-H(58)	0.9500
C(39)-H(39)	0.9500	C(59)-C(60)	1.402(10)
C(40)-C(41)	1.386(10)	C(59)-H(59)	0.9500
C(40)-H(40)	0.9500	C(60)-H(60)	0.9500
C(41)-C(42)	1.401(10)	P(2)-Fe(1)-P(4)	177.37(8)
C(41)-H(41)	0.9500	P(2)-Fe(1)-P(1)	83.26(7)
C(42)-H(42)	0.9500	P(4)-Fe(1)-P(1)	96.66(8)
C(43)-C(48)	1.384(9)	P(2)-Fe(1)-P(3)	97.16(8)
C(43)-C(44)	1.387(10)	P(4)-Fe(1)-P(3)	82.48(7)
C(44)-C(45)	1.373(10)	P(1)-Fe(1)-P(3)	170.57(7)
C(44)-H(44)	0.9500	P(2)-Fe(1)-Br(1)	90.70(6)
C(45)-C(46)	1.380(12)	P(4)-Fe(1)-Br(1)	91.93(6)
C(45)-H(45)	0.9500	P(1)-Fe(1)-Br(1)	95.37(6)
C(46)-C(47)	1.369(11)	P(3)-Fe(1)-Br(1)	94.05(6)
C(46)-H(46)	0.9500	P(2)-Fe(1)-H(1)	86(3)
C(47)-C(48)	1.392(10)	P(4)-Fe(1)-H(1)	91(3)
C(47)-H(47)	0.9500	P(1)-Fe(1)-H(1)	87(3)
C(48)-H(48)	0.9500	P(3)-Fe(1)-H(1)	84(3)

Br(1)-Fe(1)-H(1)	176(3)	C(3)-C(4)-H(4)	119.5
C(1)-P(1)-C(7)	102.5(3)	C(4)-C(5)-C(6)	121.0(7)
C(1)-P(1)-C(13)	98.3(3)	C(4)-C(5)-H(5)	119.5
C(7)-P(1)-C(13)	100.4(3)	C(6)-C(5)-H(5)	119.5
C(1)-P(1)-Fe(1)	109.2(2)	C(1)-C(6)-C(5)	118.4(6)
C(7)-P(1)-Fe(1)	114.4(2)	C(1)-C(6)-P(2)	115.0(5)
C(13)-P(1)-Fe(1)	128.3(2)	C(5)-C(6)-P(2)	126.4(5)
C(6)-P(2)-C(25)	102.0(3)	C(12)-C(7)-C(8)	117.6(6)
C(6)-P(2)-C(19)	97.4(3)	C(12)-C(7)-P(1)	124.6(5)
C(25)-P(2)-C(19)	100.1(3)	C(8)-C(7)-P(1)	117.8(5)
C(6)-P(2)-Fe(1)	108.7(2)	C(7)-C(8)-C(9)	121.4(7)
C(25)-P(2)-Fe(1)	124.2(2)	C(7)-C(8)-H(8)	119.3
C(19)-P(2)-Fe(1)	120.0(2)	C(9)-C(8)-H(8)	119.3
C(37)-P(3)-C(43)	101.5(3)	C(10)-C(9)-C(8)	118.9(7)
C(37)-P(3)-C(31)	99.5(3)	C(10)-C(9)-H(9)	120.6
C(43)-P(3)-C(31)	101.7(3)	C(8)-C(9)-H(9)	120.6
C(37)-P(3)-Fe(1)	127.3(2)	C(11)-C(10)-C(9)	120.9(7)
C(43)-P(3)-Fe(1)	115.2(2)	C(11)-C(10)-H(10)	119.5
C(31)-P(3)-Fe(1)	107.9(2)	C(9)-C(10)-H(10)	119.5
C(55)-P(4)-C(36)	101.1(3)	C(10)-C(11)-C(12)	120.5(7)
C(55)-P(4)-C(49)	102.6(3)	C(10)-C(11)-H(11)	119.8
C(36)-P(4)-C(49)	100.1(3)	C(12)-C(11)-H(11)	119.8
C(55)-P(4)-Fe(1)	121.9(2)	C(7)-C(12)-C(11)	120.8(7)
C(36)-P(4)-Fe(1)	108.2(2)	C(7)-C(12)-H(12)	119.6
C(49)-P(4)-Fe(1)	119.4(2)	C(11)-C(12)-H(12)	119.6
C(2)-C(1)-C(6)	119.6(6)	C(18)-C(13)-C(14)	118.2(7)
C(2)-C(1)-P(1)	125.0(5)	C(18)-C(13)-P(1)	117.1(5)
C(6)-C(1)-P(1)	115.4(5)	C(14)-C(13)-P(1)	124.3(5)
C(3)-C(2)-C(1)	121.7(7)	C(15)-C(14)-C(13)	121.5(7)
C(3)-C(2)-H(2)	119.1	C(15)-C(14)-H(14)	119.3
C(1)-C(2)-H(2)	119.1	C(13)-C(14)-H(14)	119.3
C(2)-C(3)-C(4)	118.2(7)	C(14)-C(15)-C(16)	120.2(7)
C(2)-C(3)-H(3)	120.9	C(14)-C(15)-H(15)	119.9
C(4)-C(3)-H(3)	120.9	C(16)-C(15)-H(15)	119.9
C(5)-C(4)-C(3)	121.0(6)	C(17)-C(16)-C(15)	119.2(7)
C(5)-C(4)-H(4)	119.5	C(17)-C(16)-H(16)	120.4

C(15)-C(16)-H(16)	120.4	C(29)-C(28)-H(28)	120.4
C(16)-C(17)-C(18)	121.2(7)	C(28)-C(29)-C(30)	120.8(7)
C(16)-C(17)-H(17)	119.4	C(28)-C(29)-H(29)	119.6
C(18)-C(17)-H(17)	119.4	C(30)-C(29)-H(29)	119.6
C(13)-C(18)-C(17)	119.7(7)	C(25)-C(30)-C(29)	119.7(7)
C(13)-C(18)-H(18)	120.1	C(25)-C(30)-H(30)	120.2
C(17)-C(18)-H(18)	120.1	C(29)-C(30)-H(30)	120.2
C(24)-C(19)-C(20)	118.7(6)	C(36)-C(31)-C(32)	121.2(6)
C(24)-C(19)-P(2)	121.8(5)	C(36)-C(31)-P(3)	114.4(5)
C(20)-C(19)-P(2)	119.5(5)	C(32)-C(31)-P(3)	124.3(5)
C(21)-C(20)-C(19)	120.5(7)	C(33)-C(32)-C(31)	119.2(6)
C(21)-C(20)-H(20)	119.7	C(33)-C(32)-H(32)	120.4
C(19)-C(20)-H(20)	119.7	C(31)-C(32)-H(32)	120.4
C(20)-C(21)-C(22)	120.6(7)	C(32)-C(33)-C(34)	120.1(7)
C(20)-C(21)-H(21)	119.7	C(32)-C(33)-H(33)	120.0
C(22)-C(21)-H(21)	119.7	C(34)-C(33)-H(33)	120.0
C(21)-C(22)-C(23)	119.5(7)	C(33)-C(34)-C(35)	120.3(6)
C(21)-C(22)-H(22)	120.3	C(33)-C(34)-H(34)	119.8
C(23)-C(22)-H(22)	120.3	C(35)-C(34)-H(34)	119.8
C(22)-C(23)-C(24)	120.1(7)	C(36)-C(35)-C(34)	119.9(7)
C(22)-C(23)-H(23)	119.9	C(36)-C(35)-H(35)	120.1
C(24)-C(23)-H(23)	119.9	C(34)-C(35)-H(35)	120.1
C(19)-C(24)-C(23)	120.5(7)	C(31)-C(36)-C(35)	119.3(6)
C(19)-C(24)-H(24)	119.7	C(31)-C(36)-P(4)	115.3(5)
C(23)-C(24)-H(24)	119.7	C(35)-C(36)-P(4)	125.4(5)
C(30)-C(25)-C(26)	119.1(7)	C(42)-C(37)-C(38)	119.1(6)
C(30)-C(25)-P(2)	119.9(5)	C(42)-C(37)-P(3)	122.3(5)
C(26)-C(25)-P(2)	120.9(5)	C(38)-C(37)-P(3)	118.5(5)
C(27)-C(26)-C(25)	119.7(7)	C(39)-C(38)-C(37)	120.1(7)
C(27)-C(26)-H(26)	120.2	C(39)-C(38)-H(38)	119.9
C(25)-C(26)-H(26)	120.2	C(37)-C(38)-H(38)	119.9
C(28)-C(27)-C(26)	121.3(7)	C(38)-C(39)-C(40)	121.6(7)
C(28)-C(27)-H(27)	119.3	C(38)-C(39)-H(39)	119.2
C(26)-C(27)-H(27)	119.3	C(40)-C(39)-H(39)	119.2
C(27)-C(28)-C(29)	119.3(7)	C(39)-C(40)-C(41)	119.1(7)
C(27)-C(28)-H(28)	120.4	C(39)-C(40)-H(40)	120.5

C(41)-C(40)-H(40)	120.5	C(52)-C(51)-C(50)	118.7(8)
C(40)-C(41)-C(42)	119.9(7)	C(52)-C(51)-H(51)	120.6
C(40)-C(41)-H(41)	120.0	C(50)-C(51)-H(51)	120.6
C(42)-C(41)-H(41)	120.0	C(53)-C(52)-C(51)	120.6(8)
C(37)-C(42)-C(41)	120.1(7)	C(53)-C(52)-H(52)	119.7
C(37)-C(42)-H(42)	119.9	C(51)-C(52)-H(52)	119.7
C(41)-C(42)-H(42)	119.9	C(52)-C(53)-C(54)	120.8(7)
C(48)-C(43)-C(44)	117.9(7)	C(52)-C(53)-H(53)	119.6
C(48)-C(43)-P(3)	123.3(5)	C(54)-C(53)-H(53)	119.6
C(44)-C(43)-P(3)	118.8(5)	C(53)-C(54)-C(49)	119.5(7)
C(45)-C(44)-C(43)	121.6(7)	C(53)-C(54)-H(54)	120.2
C(45)-C(44)-H(44)	119.2	C(49)-C(54)-H(54)	120.2
C(43)-C(44)-H(44)	119.2	C(56)-C(55)-C(60)	118.8(6)
C(44)-C(45)-C(46)	119.7(8)	C(56)-C(55)-P(4)	120.2(5)
C(44)-C(45)-H(45)	120.2	C(60)-C(55)-P(4)	120.9(5)
C(46)-C(45)-H(45)	120.2	C(57)-C(56)-C(55)	120.6(7)
C(47)-C(46)-C(45)	120.0(7)	C(57)-C(56)-H(56)	119.7
C(47)-C(46)-H(46)	120.0	C(55)-C(56)-H(56)	119.7
C(45)-C(46)-H(46)	120.0	C(56)-C(57)-C(58)	120.5(7)
C(46)-C(47)-C(48)	120.0(8)	C(56)-C(57)-H(57)	119.8
C(46)-C(47)-H(47)	120.0	C(58)-C(57)-H(57)	119.8
C(48)-C(47)-H(47)	120.0	C(59)-C(58)-C(57)	120.0(7)
C(43)-C(48)-C(47)	120.8(7)	C(59)-C(58)-H(58)	120.0
C(43)-C(48)-H(48)	119.6	C(57)-C(58)-H(58)	120.0
C(47)-C(48)-H(48)	119.6	C(58)-C(59)-C(60)	121.1(7)
C(50)-C(49)-C(54)	118.0(7)	C(58)-C(59)-H(59)	119.5
C(50)-C(49)-P(4)	120.8(6)	C(60)-C(59)-H(59)	119.5
C(54)-C(49)-P(4)	121.1(6)	C(59)-C(60)-C(55)	119.0(7)
C(49)-C(50)-C(51)	122.2(7)	C(59)-C(60)-H(60)	120.5
C(49)-C(50)-H(50)	118.9	C(55)-C(60)-H(60)	120.5
C(51)-C(50)-H(50)	118.9		

Table S75. Anisotropic displacement parameters ($Å^2$x 10^3) for neisc20. The anisotropic displacement factor exponent takes the form: $-2\pi^2[h^2 a^{*2}U_{11} + ... + 2 h k a^* b^* U_{12}]$

	U_{11}	U_{22}	U_{33}	U_{23}	U_{13}	U_{12}
Fe1	17(1)	14(1)	15(1)	1(1)	-3(1)	-6(1)
Br1	17(1)	22(1)	18(1)	3(1)	-4(1)	-7(1)
P1	17(1)	14(1)	15(1)	0(1)	-4(1)	-5(1)
P2	17(1)	17(1)	15(1)	0(1)	-1(1)	-7(1)
P3	18(1)	17(1)	16(1)	1(1)	-3(1)	-8(1)
P4	20(1)	16(1)	16(1)	2(1)	-3(1)	-7(1)
C1	23(4)	16(4)	16(4)	7(3)	-6(3)	-11(3)
C2	22(4)	26(4)	21(4)	-1(3)	-2(3)	-10(3)
C3	23(4)	20(4)	20(4)	-8(3)	0(3)	-6(3)
C4	27(4)	14(4)	28(4)	-2(3)	0(3)	-4(3)
C5	28(4)	21(4)	16(4)	0(3)	1(3)	-10(3)
C6	18(4)	23(4)	17(4)	0(3)	-5(3)	-7(3)
C7	15(4)	16(3)	14(4)	2(3)	-3(3)	-4(3)
C8	27(4)	25(4)	17(4)	-2(3)	-4(3)	-12(3)
C9	21(4)	31(4)	30(5)	3(4)	-7(4)	-4(3)
C10	25(4)	40(5)	14(4)	-3(3)	-5(3)	-15(4)
C11	17(4)	36(5)	31(5)	-9(4)	-4(3)	-15(4)
C12	20(4)	24(4)	18(4)	-4(3)	0(3)	-7(3)
C13	21(4)	16(4)	19(4)	-2(3)	-5(3)	-7(3)
C14	31(5)	23(4)	17(4)	4(3)	-5(3)	-14(4)
C15	30(4)	33(4)	16(4)	-3(3)	4(3)	-19(4)
C16	25(4)	38(5)	25(4)	-7(4)	3(3)	-19(4)
C17	24(4)	36(4)	27(5)	-5(4)	-6(3)	-16(4)
C18	22(4)	27(4)	26(4)	1(3)	-5(3)	-11(3)
C19	13(4)	20(4)	17(4)	-2(3)	0(3)	-4(3)
C20	26(4)	31(4)	26(4)	-8(3)	2(3)	-16(4)
C21	46(5)	32(4)	32(5)	-2(4)	-7(4)	-25(4)
C22	26(5)	37(5)	33(5)	-10(4)	-6(4)	-14(4)
C23	21(4)	25(4)	30(5)	-8(3)	-9(3)	-4(3)
C24	30(4)	26(4)	24(4)	-13(3)	-2(3)	-12(4)
C25	29(4)	13(3)	17(4)	-3(3)	3(3)	-12(3)

C26	25(4)	8(3)	33(4)	-3(3)	-6(3)	-6(3)
C27	30(4)	17(4)	24(4)	-3(3)	-6(3)	-8(3)
C28	43(5)	25(4)	13(4)	-1(3)	-1(4)	-21(4)
C29	28(4)	25(4)	22(4)	-1(3)	4(3)	-17(3)
C30	28(4)	20(4)	26(4)	1(3)	-5(3)	-12(3)
C31	18(4)	20(4)	16(4)	3(3)	-6(3)	-10(3)
C32	21(4)	18(4)	19(4)	0(3)	-2(3)	-11(3)
C33	26(4)	24(4)	14(4)	-7(3)	2(3)	-3(3)
C34	23(4)	17(4)	30(5)	-1(3)	-10(3)	-8(3)
C35	26(4)	19(4)	25(4)	5(3)	-6(3)	-13(3)
C36	15(4)	14(3)	19(4)	0(3)	-3(3)	-2(3)
C37	22(4)	15(4)	21(4)	-5(3)	-2(3)	-4(3)
C38	16(4)	21(4)	14(4)	-4(3)	2(3)	-9(3)
C39	31(4)	23(4)	13(4)	4(3)	-5(3)	-8(3)
C40	36(5)	32(4)	14(4)	3(3)	-1(3)	-18(4)
C41	25(4)	38(5)	25(5)	1(4)	-2(4)	-12(4)
C42	17(4)	22(4)	21(4)	0(3)	-3(3)	-1(3)
C43	19(4)	27(4)	6(4)	-4(3)	-3(3)	-6(3)
C44	27(4)	42(5)	13(4)	-5(4)	-5(3)	-13(4)
C45	24(5)	64(6)	24(5)	-4(4)	-3(4)	-19(5)
C46	10(4)	56(6)	38(5)	-2(5)	-6(4)	5(4)
C47	19(4)	37(5)	31(5)	3(4)	0(4)	6(4)
C48	23(4)	28(4)	27(5)	-4(3)	-3(3)	-8(3)
C49	24(4)	25(4)	16(4)	0(3)	-4(3)	-8(3)
C50	24(4)	30(4)	15(4)	-1(3)	-5(3)	-6(3)
C51	33(5)	41(5)	23(4)	2(4)	-8(4)	-10(4)
C52	43(5)	28(5)	34(5)	-4(4)	-8(4)	-3(4)
C53	42(5)	21(4)	25(5)	9(3)	-5(4)	-6(4)
C54	33(5)	22(4)	27(4)	-4(3)	-3(4)	-10(4)
C55	22(4)	16(4)	18(4)	4(3)	-2(3)	-10(3)
C56	24(4)	15(3)	26(4)	-1(3)	-3(3)	-13(3)
C57	20(4)	16(4)	35(5)	0(3)	-3(3)	-9(3)
C58	26(5)	33(5)	35(5)	-11(4)	8(4)	-15(4)
C59	39(5)	23(4)	16(4)	-4(3)	2(4)	-11(4)
C60	28(4)	22(4)	22(4)	-1(3)	0(3)	-11(3)

Table S76. Hydrogen coordinates (x 10^4) and isotropic displacement parameters ($Å^2$ x 10^3) for neisc20.

	x	y	z	U(eq)
H1	8060(60)	2410(60)	7560(30)	29(18)
H2	4002	5551	9257	28
H3	3322	7674	9119	26
H4	4059	8585	8067	30
H5	5474	7400	7202	27
H8	7589	1264	9005	27
H9	9284	599	9678	35
H10	9802	2046	10065	30
H11	8716	4089	9771	31
H12	7061	4767	9079	26
H14	5244	2782	9875	28
H15	3294	2717	10603	31
H16	1262	3275	10195	33
H17	1224	3864	9052	32
H18	3213	3844	8296	30
H20	7909	6638	6942	32
H21	9794	6895	7131	40
H22	11515	5271	7604	37
H23	11358	3358	7871	31
H24	9442	3101	7700	30
H26	4689	6264	6446	26
H27	4483	7480	5424	29
H28	6404	7384	4618	30
H29	8574	6211	4873	30
H30	8829	5014	5911	28
H32	7974	859	5217	22
H33	7220	-669	5139	29
H34	6263	-1562	6121	27
H35	6023	-912	7183	27
H38	6020	3741	5632	21

H39	6038	4683	4546	28
H40	8088	4402	3792	32
H41	10168	3065	4126	36
H42	10173	2114	5236	27
H44	10303	2523	6523	32
H45	12619	1424	6537	44
H46	13565	-696	6472	49
H47	12210	-1683	6342	44
H48	9895	-557	6277	32
H50	9358	647	7866	29
H51	11221	-968	8250	41
H52	11084	-2873	8596	46
H53	9108	-3138	8600	40
H54	7247	-1539	8191	34
H56	4007	1424	7407	24
H57	2064	1171	8061	29
H58	2015	459	9204	38
H59	3917	-46	9690	33
H60	5909	184	9051	29

Table S77. Torsion angles [°] for neisc20.

C7-P1-C1-C2	-73.5(6)	C8-C7-C12-C11	0.0(10)
C13-P1-C1-C2	29.2(7)	P1-C7-C12-C11	-178.3(5)
Fe1-P1-C1-C2	164.8(5)	C10-C11-C12-C7	0.4(11)
C7-P1-C1-C6	105.8(5)	C1-P1-C13-C18	71.3(6)
C13-P1-C1-C6	-151.5(5)	C7-P1-C13-C18	175.8(5)
Fe1-P1-C1-C6	-15.9(6)	Fe1-P1-C13-C18	-51.3(6)
C6-C1-C2-C3	-1.7(10)	C1-P1-C13-C14	-100.9(6)
P1-C1-C2-C3	177.6(5)	C7-P1-C13-C14	3.6(7)
C1-C2-C3-C4	2.6(10)	Fe1-P1-C13-C14	136.5(5)
C2-C3-C4-C5	-1.0(11)	C18-C13-C14-C15	1.5(10)
C3-C4-C5-C6	-1.5(11)	P1-C13-C14-C15	173.7(6)
C2-C1-C6-C5	-0.8(10)	C13-C14-C15-C16	-1.9(11)
P1-C1-C6-C5	179.8(5)	C14-C15-C16-C17	0.5(11)
C2-C1-C6-P2	174.1(5)	C15-C16-C17-C18	1.2(11)
P1-C1-C6-P2	-5.2(7)	C14-C13-C18-C17	0.1(10)
C4-C5-C6-C1	2.4(10)	P1-C13-C18-C17	-172.6(6)
C4-C5-C6-P2	-171.9(6)	C16-C17-C18-C13	-1.5(11)
C25-P2-C6-C1	157.1(5)	C6-P2-C19-C24	115.0(6)
C19-P2-C6-C1	-100.9(5)	C25-P2-C19-C24	-141.3(6)
Fe1-P2-C6-C1	24.3(6)	Fe1-P2-C19-C24	-1.6(7)
C25-P2-C6-C5	-28.4(7)	C6-P2-C19-C20	-63.4(6)
C19-P2-C6-C5	73.6(7)	C25-P2-C19-C20	40.3(6)
Fe1-P2-C6-C5	-161.2(6)	Fe1-P2-C19-C20	180.0(5)
C1-P1-C7-C12	-4.8(7)	C24-C19-C20-C21	1.7(11)
C13-P1-C7-C12	-105.8(6)	P2-C19-C20-C21	-179.8(6)
Fe1-P1-C7-C12	113.3(6)	C19-C20-C21-C22	-0.7(11)
C1-P1-C7-C8	176.9(6)	C20-C21-C22-C23	0.3(12)
C13-P1-C7-C8	75.8(6)	C21-C22-C23-C24	-0.9(11)
Fe1-P1-C7-C8	-65.1(6)	C20-C19-C24-C23	-2.3(10)
C12-C7-C8-C9	-0.9(10)	P2-C19-C24-C23	179.3(6)
P1-C7-C8-C9	177.6(5)	C22-C23-C24-C19	2.0(11)
C7-C8-C9-C10	1.3(11)	C6-P2-C25-C30	138.3(6)
C8-C9-C10-C11	-0.8(11)	C19-P2-C25-C30	38.5(6)
C9-C10-C11-C12	0.0(11)	Fe1-P2-C25-C30	-98.8(6)

C6-P2-C25-C26	-44.1(6)	Fe1-P3-C37-C42	135.6(5)
C19-P2-C25-C26	-144.0(5)	C43-P3-C37-C38	176.5(5)
Fe1-P2-C25-C26	78.7(6)	C31-P3-C37-C38	72.4(6)
C30-C25-C26-C27	-4.0(10)	Fe1-P3-C37-C38	-48.9(6)
P2-C25-C26-C27	178.4(5)	C42-C37-C38-C39	-0.7(10)
C25-C26-C27-C28	3.9(10)	P3-C37-C38-C39	-176.3(5)
C26-C27-C28-C29	-2.8(10)	C37-C38-C39-C40	-0.2(11)
C27-C28-C29-C30	1.7(10)	C38-C39-C40-C41	1.5(11)
C26-C25-C30-C29	3.0(10)	C39-C40-C41-C42	-1.8(11)
P2-C25-C30-C29	-179.4(5)	C38-C37-C42-C41	0.3(10)
C28-C29-C30-C25	-1.9(10)	P3-C37-C42-C41	175.8(5)
C37-P3-C31-C36	-160.1(5)	C40-C41-C42-C37	1.0(11)
C43-P3-C31-C36	96.0(5)	C37-P3-C43-C48	-104.6(6)
Fe1-P3-C31-C36	-25.6(5)	C31-P3-C43-C48	-2.2(7)
C37-P3-C31-C32	22.7(6)	Fe1-P3-C43-C48	114.2(6)
C43-P3-C31-C32	-81.2(6)	C37-P3-C43-C44	74.9(6)
Fe1-P3-C31-C32	157.2(5)	C31-P3-C43-C44	177.3(5)
C36-C31-C32-C33	-0.2(10)	Fe1-P3-C43-C44	-66.3(6)
P3-C31-C32-C33	176.8(5)	C48-C43-C44-C45	-0.5(11)
C31-C32-C33-C34	-0.9(10)	P3-C43-C44-C45	179.9(6)
C32-C33-C34-C35	0.6(11)	C43-C44-C45-C46	-1.4(11)
C33-C34-C35-C36	0.7(11)	C44-C45-C46-C47	1.7(12)
C32-C31-C36-C35	1.5(10)	C45-C46-C47-C48	-0.1(12)
P3-C31-C36-C35	-175.8(5)	C44-C43-C48-C47	2.1(11)
C32-C31-C36-P4	179.7(5)	P3-C43-C48-C47	-178.4(6)
P3-C31-C36-P4	2.5(7)	C46-C47-C48-C43	-1.8(12)
C34-C35-C36-C31	-1.7(10)	C55-P4-C49-C50	-145.9(6)
C34-C35-C36-P4	-179.8(5)	C36-P4-C49-C50	110.1(6)
C55-P4-C36-C31	151.0(5)	Fe1-P4-C49-C50	-7.5(7)
C49-P4-C36-C31	-103.8(5)	C55-P4-C49-C54	36.2(7)
Fe1-P4-C36-C31	21.9(6)	C36-P4-C49-C54	-67.8(6)
C55-P4-C36-C35	-30.8(7)	Fe1-P4-C49-C54	174.6(5)
C49-P4-C36-C35	74.3(6)	C54-C49-C50-C51	1.2(11)
Fe1-P4-C36-C35	-160.0(5)	P4-C49-C50-C51	-176.8(6)
C43-P3-C37-C42	1.0(7)	C49-C50-C51-C52	-0.4(12)
C31-P3-C37-C42	-103.1(6)	C50-C51-C52-C53	-1.2(12)

C51-C52-C53-C54	2.1(12)
C52-C53-C54-C49	-1.2(12)
C50-C49-C54-C53	-0.4(11)
P4-C49-C54-C53	177.6(6)
C36-P4-C55-C56	-44.5(6)
C49-P4-C55-C56	-147.7(6)
Fe1-P4-C55-C56	75.3(6)
C36-P4-C55-C60	138.6(6)
C49-P4-C55-C60	35.5(6)
Fe1-P4-C55-C60	-101.5(5)
C60-C55-C56-C57	-2.3(10)
P4-C55-C56-C57	-179.2(5)
C55-C56-C57-C58	1.5(10)
C56-C57-C58-C59	-0.5(11)
C57-C58-C59-C60	0.4(11)
C58-C59-C60-C55	-1.2(11)
C56-C55-C60-C59	2.2(10)
P4-C55-C60-C59	179.0(5)

4. DFT Calculations Data

4.1 Computational Details

All geometry optimizations of intermediates and transition states were achieved using spin-unrestricted UB3LYP[8]/def2-SVP[9] method, in gas phase with “opt=noeigen” and “guess=mix” keywords as implemented in Gaussian09[10] and Gaussian16[11]. Frequency calculations were also conducted at the same level of theory to obtain vibrational frequencies to determine the identity of stationary points as intermediates (no imaginary frequencies) or transition states (only one imaginary frequency), as well as obtaining the thermal corrections to enthalpy ($H_{correction}$) and free energy ($G_{correction}$) at the temperature of 298 K. Energies were refined by computing single point energies with UB3LYP-D3/def2-SVP and UPBEPBE[12]-D3/def2-SVP method in THF solvent using the SMD solvent model[13] . All structural figures were generated with CYLview.[14] Distances in structural figures are shown in Å and energies are in kcal/mol.

4.2 Complete Energetic Diagrams

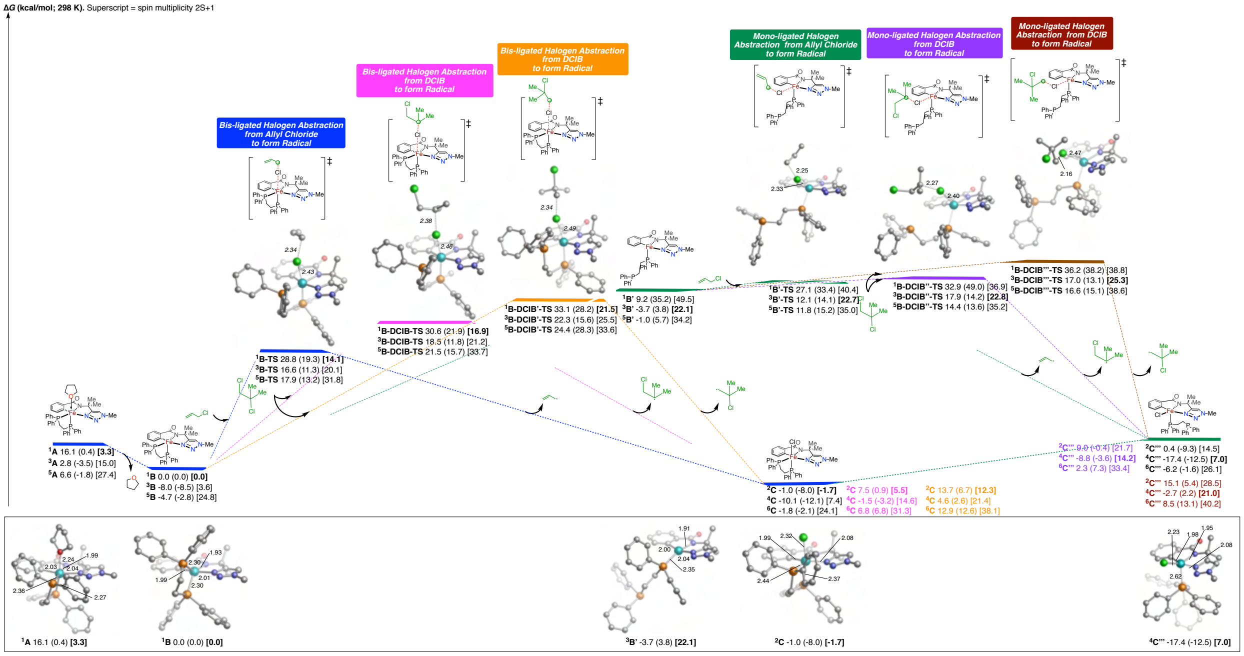

Figure S30. Calculated energetics of the halogen abstraction step of the iron-catalyzed triazole-directed C-H allylation. Free energies (kcal/mol) were computed at the UB3LYP/def2-SVP(gas), UB3LYP-D3/def2-SVP-SMD(THF)// UB3LYP/def2-SVP(gas) (in parenthesis), and UPBEPBE-D3/def2-SVP-SMD(THF)// UB3LYP/def2-SVP(gas) (in bracket) levels of theory.

To model the system, we truncated the benzyl group on the triazole directing group to a methyl group and also use the unsubstituted aryl ring on the substrate. The use of DCIB is the calculations is also consistent with experiment which show slower kinetics for this reaction. However, for simplicity we only discussed the lowest energy pathway (blue pathway) that uses the allyl chloride as the substrate (Figure S30). In particular, the low spin ^{1}**A** could equilibrate to the desolvated ^{1}**B** easily (downhill in only ~3 kcal/mol). In turn, ^{1}**B** then could undergo facile halogen atom abstraction from allyl chloride (barrier is only ~14 kcal/mol) to form the corresponding allyl radical and ^{2}**C**, which is only 1.7 kcal/mol lower in energy. Specifically, the six coordinated ^{2}**C** could decomplex the equatorial phosphine to form the ^{4}**C'''** which can then undergo the future C-C bond formation step.

We also considered the bis-ligated halogen atom abstraction from DCIB pathways (pink and orange). In these two pathways, their barriers are ~17 and 21 kcal/mol, which are ~3 and 7 kcal/mol higher than the blue pathway respectively.

Also, the five coordinated ^{1}**B** could decomplex the equatorial phosphine to form the ^{3}**B'** (uphill ~22 kcal/mol in energy), followed by mono-ligated halogen atom abstraction to generate the ^{4}**C'''** intermediate. Specifically, ^{4}**C'''** could undergo halogen atom abstraction with allyl chloride (via ^{1}**B'-TS**) to form the ^{4}**C'''** and allyl radical (barrier is only ~1 kcal/mol). However, with DCIB, the energy barrier is higher than that of allyl chloride (via ^{3}**B-DCIB''-TS** and ^{3}**B-DCIB'''-TS**), which is consistent with the experiments.

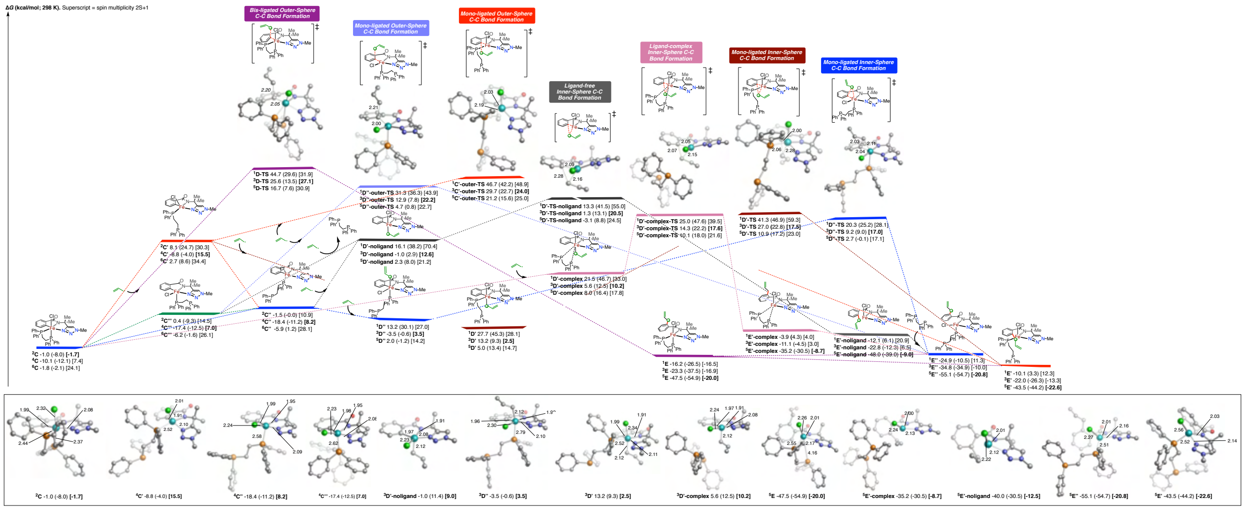

Figure S31. Calculated energetics of the C-C bond formation step of the iron-catalyzed triazole-directed C-H allylation. Free energies (kcal/mol) were computed at the UB3LYP/def2-SVP(gas), UB3LYP-D3/def2-SVP-SMD(THF)//UB3LYP/def2-SVP(gas) (in parenthesis), and UPBEPBE-D3/def2-SVP-SMD(THF)//UB3LYP/def2-SVP(gas) (in bracket) levels of theory.

The lowest energy pathway is the blue pathway as discussed in the main text. Starting from the six coordinated ^{2}**C,** ^{2}**C** could decomplex the equatorial phosphine to form a distorted five coordinated square pyramidal species ^{4}**C''**. This Fe(III) species could then trap the allyl radical at the axial position to form the more energetically stable six coordinated ^{3}**D''** species. Finally, this structure is poised to undergo reductive elimination (via ^{3}**D''-TS**) to form the experimentally detected high-spin iron(II) product bound structure ^{5}**E''**.
From the six coordinated ^{2}**C,** we also considered the bis-ligated outer-sphere C-C bond formation pathway (via ^{3}**D-TS**). The barrier is ~29 kcal/mol, which is a much higher barrier than the mono-ligated inner-sphere pathway.
Also, the ^{2}**C** could decomplex the equatorial phosphine to form a distorted five coordinated square pyramidal species ^{4}**C'''**, which could undergo mono-ligated outer-sphere reductive elimination via ^{3}**D''-outer-TS** to form the final product bond structure ^{5}**E''**. The barrier is ~15 kcal/mol in this step. Furthermore, the ^{2}**C** could decomplex the axial phosphine to form a tetrahedral species ^{4}**C'**(uphill in energy by ~17 kcal/mol), which could undergo mono-ligated outer-sphere reductive elimination via ^{3}**C'-outer-TS** to form the final product ^{5}**E'**. Also, the iron(III) species ^{4}**C'** could undergo radical addition with the allyl radical to form the six coordinated octahedral iron species ^{3}**D'** (downhill in energy by ~13 kcal/mol), followed by mono-ligated inner-sphere reductive elimination (via ^{3}**D'-TS**) to form the quintet iron(II) product bound structure ^{5}**E'**.
However, from the six coordinated ^{2}**C,** ^{2}**C** could decomplex the phosphine partially or completely followed by radical addition to form the square pyramidal iron species ^{3}**D-complex** and ^{3}**D-noligand**, respectively. Then, these square pyramidal iron species could undergo inner-sphere C-C bond formation to form the quintet product bound structure ^{5}**E'-noligand** and ^{5}**E'-complex**.

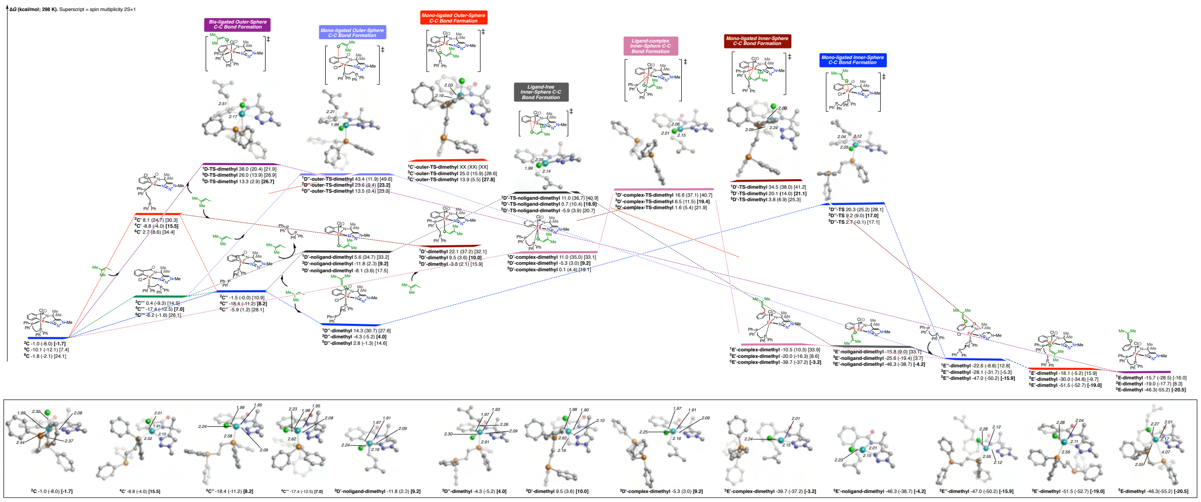

Figure S32. Calculated energetics of the C-C bond formation step of the iron-catalyzed triazole-directed C-H allylation with the steric allyl radical. Free energies (kcal/mol) were computed at the UB3LYP/def2-SVP(gas), UB3LYP-D3/def2-SVP-SMD(THF)//UB3LYP/def2-SVP(gas) (in parenthesis), and UPBEPBE-D3/def2-SVP-SMD(THF)//UB3LYP/def2-SVP(gas) (in bracket) levels of theory.

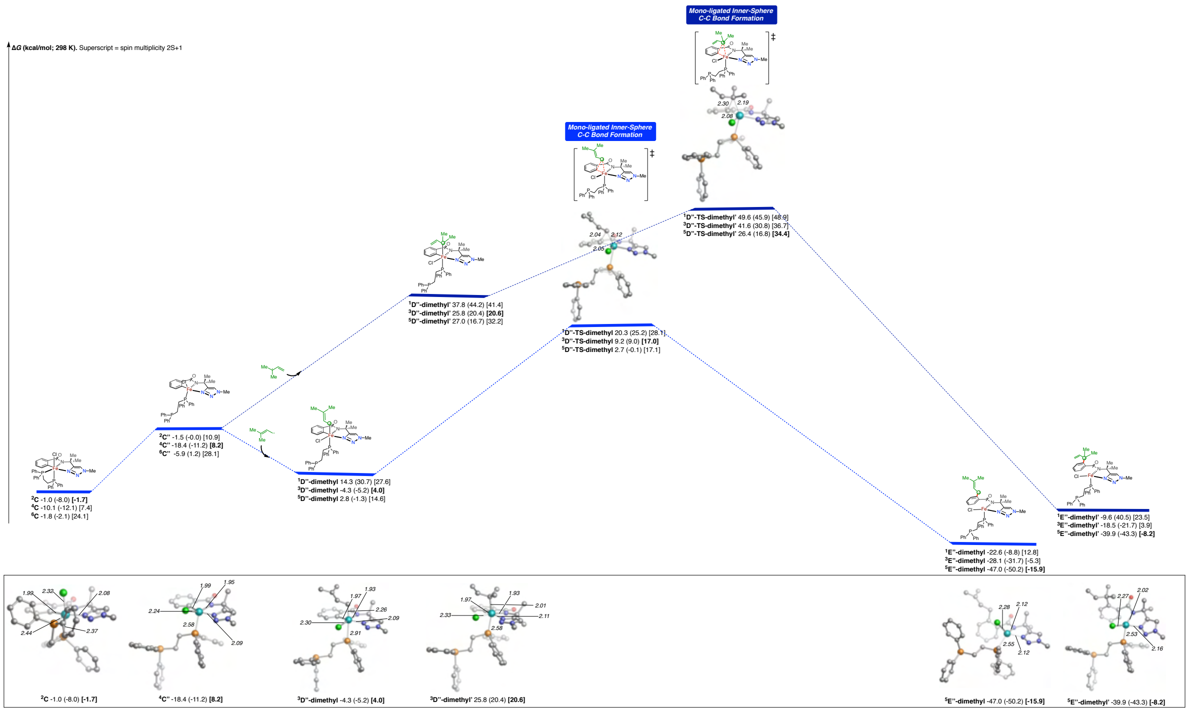

Figure S33. Calculated energetics of the C-C bond formation step of the iron-catalyzed triazole-directed C-H allylation with the allyl radical and its relative resonance radical. Free energies (kcal/mol) were computed at the UB3LYP/def2-SVP(gas), UB3LYP-D3/def2-SVP-SMD(THF)//UB3LYP/def2-SVP(gas) (in parenthesis), and UPBEPBE-D3/def2-SVP-SMD(THF)//UB3LYP/def2-SVP(gas) (in bracket) levels of theory.

We performed computations to investigate the effect of the steric effect in the lowest pathway (via monoligated inner-sphere C-C bond formation) by using the resonance structure of the dimethyl alkyl radical (Figure S33). Presumably, the less sterically primary terminus does not pay a penalty to form the Fe-alkyl species and can quickly undergo inner- sphere C-C bond formation to form the product. Thus, from these results, we hypothesize that for these monoligated inner-sphere C-C bond formation pathways, sterics make a large difference in the nature of the C-C bond formation step.

4.3 Coordinates and Energies

Figure S30

allylchloride

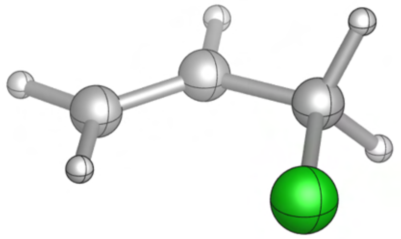

Zero-point correction= 0.070969 (Hartree/Particle)
Thermal correction to Energy= 0.075701
Thermal correction to Enthalpy= 0.076645
Thermal correction to Gibbs Free Energy= 0.043106
Sum of electronic and zero-point Energies= -577.214043
Sum of electronic and thermal Energies= -577.209312
Sum of electronic and thermal Enthalpies= -577.208367
Sum of electronic and thermal Free Energies= -577.241907

C	-3.18704600	-0.51449700	0.44949800
H	-2.80750100	-1.40460100	0.95735800
H	-4.26489400	-0.33520400	0.46992400
C	-2.36516000	0.32852600	-0.17608600
H	-2.77578200	1.21526100	-0.67796900
C	-0.87309500	0.22881000	-0.29646400
H	-0.57282100	0.18433300	-1.35526800
H	-0.38996700	1.11618400	0.14213300
Cl	-0.13924100	-1.20832200	0.50825200

UB3LYP-D3/def2-SVP-SMD(THF)//UB3LYP/def2-SVP(gas)
HF= -577.2952299
UPBEPBE-D3/def2-SVP-SMD(THF)//UB3LYP/def2-SVP(gas)
HF= -576.9231999

Allyl radical

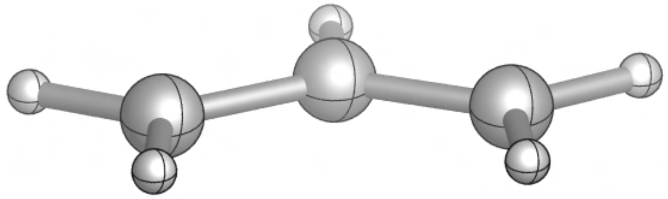

Zero-point correction= 0.065771 (Hartree/Particle)
Thermal correction to Energy= 0.069599
Thermal correction to Enthalpy= 0.070543
Thermal correction to Gibbs Free Energy= 0.040614
Sum of electronic and zero-point Energies= -117.111382
Sum of electronic and thermal Energies= -117.107554
Sum of electronic and thermal Enthalpies= -117.106610
Sum of electronic and thermal Free Energies= -117.136539

C	-2.95546800	-1.09114100	0.05291300
H	-2.45481600	-2.04249900	0.25760800
H	-4.03316900	-1.11556600	-0.12302500
C	-2.24375700	0.09974800	0.01903000
H	-2.80739900	1.01777700	-0.19117900
C	-0.87727400	0.22518400	0.22889500
H	-0.38094200	1.19725600	0.18718600
H	-0.25611800	-0.65019200	0.44221400

UB3LYP-D3/def2-SVP-SMD(THF)//UB3LYP/def2-SVP(gas)
HF= -117.1809493
UPBEPBE-D3/def2-SVP-SMD(THF)//UB3LYP/def2-SVP(gas)
HF= -117.0077079

DCIB

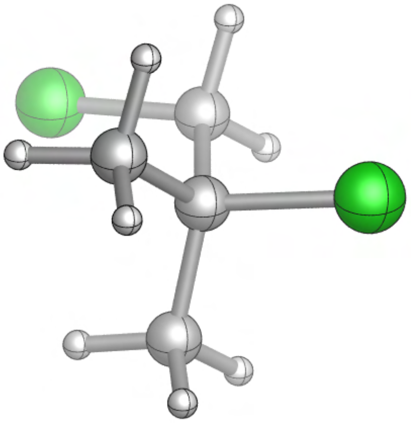

Zero-point correction= 0.113248 (Hartree/Particle)
Thermal correction to Energy= 0.120730
Thermal correction to Enthalpy= 0.121674
Thermal correction to Gibbs Free Energy= 0.081296
Sum of electronic and zero-point Energies= -1077.164846
Sum of electronic and thermal Energies= -1077.157364
Sum of electronic and thermal Enthalpies= -1077.156420
Sum of electronic and thermal Free Energies= -1077.196798

Cl	-17.09024500	-0.33228900	-0.85865200
C	-17.30688600	-1.03057400	0.84513400
C	-17.43711500	-2.54423500	0.70801300
H	-18.28131000	-2.81127500	0.05532800
H	-16.51895900	-2.97242300	0.28093300
H	-17.60806200	-2.99019400	1.70005000
C	-16.08920000	-0.62109200	1.66773900
H	-15.17270300	-1.05274000	1.24052400
H	-15.97855400	0.47301000	1.69360900
H	-16.20404500	-0.98458600	2.70063900
C	-18.60109600	-0.36583700	1.33127000
H	-18.49118000	0.72556600	1.35367600
H	-19.44203300	-0.63117300	0.67838700
Cl	-19.05553700	-0.88304100	3.01004700

UB3LYP-D3/def2-SVP-SMD(THF)//UB3LYP/def2-SVP(gas)
HF= -1077.3004351
UPBEPBE-D3/def2-SVP-SMD(THF)//UB3LYP/def2-SVP(gas)
HF= -1076.6643967

DCIB radical 1

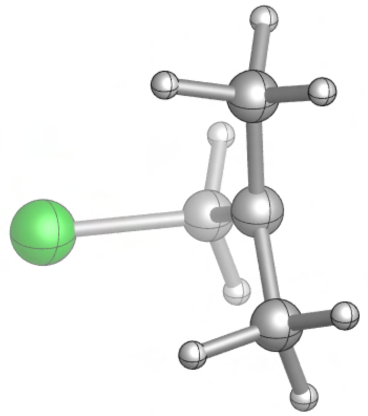

Zero-point correction= 0.107957 (Hartree/Particle)
Thermal correction to Energy= 0.115128
Thermal correction to Enthalpy= 0.116073
Thermal correction to Gibbs Free Energy= 0.075758
Sum of electronic and zero-point Energies= -617.045599
Sum of electronic and thermal Energies= -617.038427
Sum of electronic and thermal Enthalpies= -617.037483
Sum of electronic and thermal Free Energies= -617.077798

C	-17.35607300	-1.11063600	1.09381700
C	-17.44247500	-2.54519500	0.68001500
H	-18.30029500	-2.73285100	0.01407300
H	-16.52358300	-2.88076100	0.17206500
H	-17.57775200	-3.20136700	1.56499100
C	-16.07519800	-0.59713500	1.67040500
H	-15.19769200	-0.98377100	1.12667300
H	-16.03238800	0.50405300	1.66832400
H	-15.96397600	-0.91779100	2.72723800
C	-18.59411400	-0.35813500	1.32228000
H	-18.45312700	0.72985200	1.33137100
H	-19.40985600	-0.63505400	0.64289600
Cl	-19.30149600	-0.72878000	3.04111200

UB3LYP-D3/def2-SVP-SMD(THF)//UB3LYP/def2-SVP(gas)
HF= -617.1689322
UPBEPBE-D3/def2-SVP-SMD(THF)//UB3LYP/def2-SVP(gas)
HF= -616.7343124

DCIB radical 2

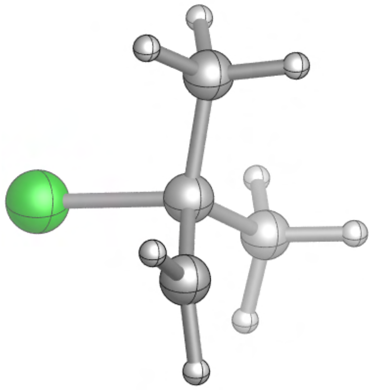

Zero-point correction= 0.107253 (Hartree/Particle)
Thermal correction to Energy= 0.114058
Thermal correction to Enthalpy= 0.115002
Thermal correction to Gibbs Free Energy= 0.076891
Sum of electronic and zero-point Energies= -617.037624
Sum of electronic and thermal Energies= -617.030819
Sum of electronic and thermal Enthalpies= -617.029875
Sum of electronic and thermal Free Energies= -617.067986

C	-17.44497100	-2.51741200	0.67481200
H	-18.28067200	-2.77528100	0.00835100
H	-16.52649500	-2.96573500	0.26939700
H	-17.64173000	-2.95676600	1.66753600
C	-16.09346500	-0.59574100	1.64906100
H	-15.16825300	-1.03274900	1.24654900
H	-15.98026300	0.49779000	1.67009500
H	-16.22890600	-0.95251600	2.68424300
C	-18.54293700	-0.32346200	1.18961600
H	-18.51133200	0.69160700	1.59346400
H	-19.50829300	-0.72633300	0.87300300
C	-17.29861900	-1.00545400	0.80925600
Cl	-16.92609700	-0.36433800	-0.97194500

UB3LYP-D3/def2-SVP-SMD(THF)//UB3LYP/def2-SVP(gas)
HF= -617.1608167
UPBEPBE-D3/def2-SVP-SMD(THF)//UB3LYP/def2-SVP(gas)
HF= -616.7246324

THF

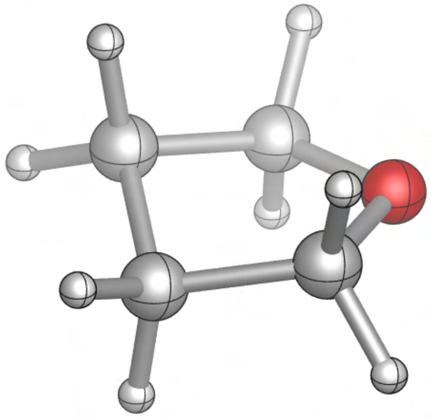

Zero-point correction= 0.116229 (Hartree/Particle)
Thermal correction to Energy= 0.121115
Thermal correction to Enthalpy= 0.122059
Thermal correction to Gibbs Free Energy= 0.088129
Sum of electronic and zero-point Energies= -232.162413
Sum of electronic and thermal Energies= -232.157527
Sum of electronic and thermal Enthalpies= -232.156583
Sum of electronic and thermal Free Energies= -232.190513

C	-0.37642300	2.34148900	0.79139900
O	-0.10847800	2.42721500	-0.60388000
C	-1.31962700	2.56610000	-1.33848300
C	-2.41066300	2.87775100	-0.30805000
C	-1.88838500	2.12716500	0.92369600
H	0.21642400	1.51852300	1.22595900
H	-0.06376700	3.27916600	1.29339500
H	-1.19797000	3.36156900	-2.09344800
H	-1.54278800	1.62427400	-1.87904100
H	-2.45212100	3.96134900	-0.10619900
H	-3.41249400	2.55632600	-0.63082900
H	-2.29476000	2.50114000	1.87562300
H	-2.13367600	1.05430600	0.84985100

UB3LYP-D3/def2-SVP-SMD(THF)//UB3LYP/def2-SVP(gas)
HF= -232.2916192
UPBEPBE-D3/def2-SVP-SMD(THF)//UB3LYP/def2-SVP(gas)
HF= -231.9875088

dppe

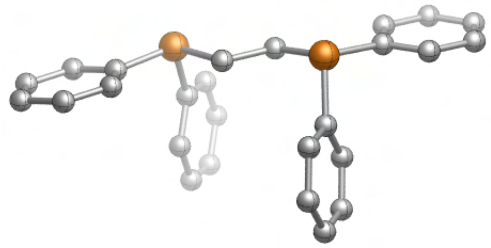

Zero-point correction= 0.421999 (Hartree/Particle)
Thermal correction to Energy= 0.447471
Thermal correction to Enthalpy= 0.448415
Thermal correction to Gibbs Free Energy= 0.359480
Sum of electronic and zero-point Energies= -1686.607291
Sum of electronic and thermal Energies= -1686.581818
Sum of electronic and thermal Enthalpies= -1686.580874
Sum of electronic and thermal Free Energies= -1686.669809

C	1.65331700	0.74572500	0.11416100
H	1.51859000	0.38478700	1.14728000
H	2.63420600	1.24875300	0.06690800
C	1.62300500	-0.42570500	-0.87360600
H	0.65169000	-0.94566200	-0.82869800
H	1.74696500	-0.04904300	-1.90324300
P	3.03189900	-1.62965500	-0.57307200
P	0.36652100	2.04682700	-0.30506100
C	2.48691000	-2.40638100	1.02745100
C	3.25907600	-2.15491800	2.17392000
C	1.34201100	-3.21396800	1.15065900
C	2.89299000	-2.68600600	3.41595500
H	4.15984900	-1.54014400	2.08946300
C	0.97729400	-3.74760000	2.38865600
H	0.73365800	-3.43391200	0.26937600
C	1.75145900	-3.48366700	3.52477400
H	3.50541300	-2.48040400	4.29800000
H	0.08508700	-4.37458000	2.46811800
H	1.46564300	-3.90423400	4.49254600
C	2.65492600	-2.98621300	-1.77932200
C	3.46975500	-4.13445600	-1.72788900
C	1.67631400	-2.91222000	-2.78489900
C	3.29885200	-5.18022400	-2.63528400
H	4.24893800	-4.20926800	-0.96332300
C	1.51235200	-3.95654300	-3.70331700
H	1.02682600	-2.03869800	-2.86462400
C	2.31834100	-5.09400300	-3.63057900
H	3.93882400	-6.06432200	-2.57088700
H	0.74468300	-3.87745300	-4.47790800
H	2.18752300	-5.90901000	-4.34705300
C	-1.19630300	1.14777200	0.15449700
C	-2.08351600	0.80595000	-0.87985000
C	-1.52559200	0.78234200	1.47214700
C	-3.26513500	0.10642600	-0.60941800

H	-1.84661100	1.09664700	-1.90739100
C	-2.70604900	0.08737100	1.74412600
H	-0.85683200	1.04975900	2.29458200
C	-3.57815700	-0.25350000	0.70346800
H	-3.94437800	-0.15151500	-1.42638500
H	-2.94868200	-0.18868000	2.77387100
H	-4.50287200	-0.79548200	0.91864900
C	0.53042400	3.23350400	1.11004100
C	-0.39150000	4.29756600	1.16327900
C	1.55052000	3.19119000	2.07541400
C	-0.30975800	5.27243900	2.15785600
H	-1.18594800	4.36098200	0.41351900
C	1.64011300	4.17620500	3.06672800
H	2.28910900	2.38765600	2.06823800
C	0.71054200	5.21656900	3.11455900
H	-1.04106900	6.08478700	2.18264900
H	2.44256000	4.12377900	3.80760200
H	0.78091200	5.98339100	3.89026500

UB3LYP-D3/def2-SVP-SMD(THF)//UB3LYP/def2-SVP(gas)
HF= -1687.10642241
UPBEPBE-D3/def2-SVP-SMD(THF)//UB3LYP/def2-SVP(gas)
HF= -1685.43181199

^{1}A

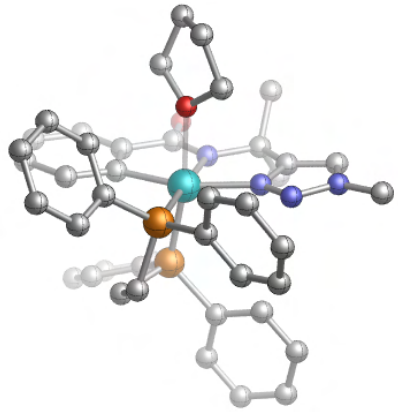

UB3LYP/def2-SVP(gas)
Zero-point correction= 0.801247 (Hartree/Particle)
Thermal correction to Energy= 0.850424
Thermal correction to Enthalpy= 0.851368
Thermal correction to Gibbs Free Energy= 0.716868
Sum of electronic and zero-point Energies= -3979.584082
Sum of electronic and thermal Energies= -3979.534905
Sum of electronic and thermal Enthalpies= -3979.533961
Sum of electronic and thermal Free Energies= -3979.668462

C	-18.65711500	1.40104500	-3.73508500
C	-19.13673900	2.65951000	-3.28067400
C	-20.49527900	2.98804100	-3.23620000
C	-21.45340100	2.06061200	-3.65403200
C	-21.02594500	0.81215500	-4.11497700
C	-19.65715800	0.49895400	-4.15388600
H	-20.77069100	3.98014500	-2.86821800
H	-22.51891100	2.30674500	-3.62363100
H	-21.76009800	0.07002100	-4.44665000
H	-19.38929800	-0.49536300	-4.52315800
C	-18.12299100	3.65947900	-2.80022000
O	-18.44317000	4.78209200	-2.37179400
C	-15.74743000	3.90214900	-2.30950800
C	-15.94956500	4.24021400	-0.80871900
H	-15.08077300	4.78543300	-0.40271900
H	-16.84847100	4.86156800	-0.70654700
H	-16.08284600	3.31972500	-0.22053600
C	-15.45702800	5.21196700	-3.08396100
H	-16.36379000	5.83125100	-3.07324800
H	-14.62979500	5.77544400	-2.61982500
H	-15.17325900	4.99350000	-4.12430500
C	-14.56919800	2.97002200	-2.44048300
N	-14.72871100	1.74139300	-3.01853400
N	-13.59915400	1.08283100	-3.00761600
C	-13.24411800	3.05883900	-2.05869900
H	-12.67414200	3.84248300	-1.56776700

N	-12.69299700	1.87146700	-2.43132400
N	-16.87451800	3.14828400	-2.86560000
C	-15.83186500	-0.00540600	-0.68143800
C	-17.99087500	0.95926300	-0.61740000
C	-16.53311600	-0.53729700	0.56376500
H	-15.07811500	0.75814800	-0.41261400
H	-15.33564000	-0.77292900	-1.29048900
C	-17.58614800	0.55255400	0.80400400
H	-18.88000200	0.41890500	-0.97730300
H	-18.18458800	2.03296100	-0.72531100
H	-17.01346400	-1.50609200	0.34776300
H	-15.84509300	-0.67988000	1.41088900
H	-18.44547700	0.20874800	1.39853900
H	-17.13347100	1.40522800	1.33627700
O	-16.87323900	0.59111100	-1.46408400
Fe	-16.63998200	1.30453000	-3.57591900
P	-16.29095800	-0.88226500	-4.40151100
P	-16.73723300	1.92893000	-5.75632900
C	-16.43806800	-0.78915900	-6.27705800
H	-16.84484100	-1.72848400	-6.67971800
H	-15.42211900	-0.68461600	-6.68420600
C	-17.29200900	0.40865300	-6.69408200
H	-18.34319700	0.23772900	-6.42586700
H	-17.23783500	0.57944500	-7.78065100
C	-17.41219800	-2.29924300	-3.94908800
C	-18.21866300	-2.20028400	-2.80567000
C	-17.44479500	-3.49245800	-4.69328600
C	-19.04085200	-3.26378700	-2.41578600
H	-18.20636900	-1.27475500	-2.23224100
C	-18.27149900	-4.55110100	-4.31097000
H	-16.80807900	-3.61044700	-5.57376300
C	-19.07260800	-4.43926000	-3.16897400
H	-19.66745600	-3.16539100	-1.52549100
H	-18.28694600	-5.46919700	-4.90432900
H	-19.71997600	-5.26771800	-2.86963200
C	-14.65102900	-1.76165300	-4.19933700
C	-13.54004600	-1.45884300	-5.00449200
C	-14.47687600	-2.70553800	-3.17080300
C	-12.30827700	-2.08801800	-4.80378900
H	-13.61748000	-0.71559800	-5.79954300
C	-13.24187700	-3.32468800	-2.95677700
H	-15.32018900	-2.97896800	-2.53330800
C	-12.15102900	-3.02261200	-3.77629500
H	-11.46654000	-1.84410100	-5.45794300
H	-13.13833600	-4.05819300	-2.15256600
H	-11.18884500	-3.51778400	-3.62087200
C	-15.25516100	2.41434700	-6.79400800
C	-15.40735900	2.96473000	-8.08111700
C	-13.95387900	2.18136600	-6.32135000
C	-14.29427700	3.26937200	-8.86765000
H	-16.40750600	3.16589900	-8.47165300

C	-12.83697100	2.48661500	-7.10954500
H	-13.80958600	1.75313600	-5.33045100
C	-13.00298300	3.03233500	-8.38367600
H	-14.43620800	3.69791900	-9.86345600
H	-11.83233300	2.29788500	-6.72092800
H	-12.13199200	3.27486600	-8.99824800
C	-17.94414700	3.22607500	-6.29206500
C	-19.17244400	2.91761900	-6.89848800
C	-17.63210500	4.57688200	-6.06435500
C	-20.06004200	3.93239100	-7.26886200
H	-19.46396300	1.88203100	-7.07199500
C	-18.52031200	5.58975600	-6.42921700
H	-16.68904200	4.84500300	-5.58874500
C	-19.73863300	5.27101300	-7.03512900
H	-21.01375700	3.66875500	-7.73325200
H	-18.26103900	6.63230900	-6.22906100
H	-20.43774400	6.06272500	-7.31667800
C	-11.32589900	1.41088800	-2.27455800
H	-11.03745300	1.41708200	-1.21284700
H	-11.27800400	0.38564300	-2.66128500
H	-10.63400600	2.05214800	-2.84126000

UB3LYP-D3/def2-SVP-SMD(THF)//UB3LYP/def2-SVP(gas)
HF= -3980.59991619
UPBEPBE-D3/def2-SVP-SMD(THF)//UB3LYP/def2-SVP(gas)
HF= -3977.432823840

[3]A

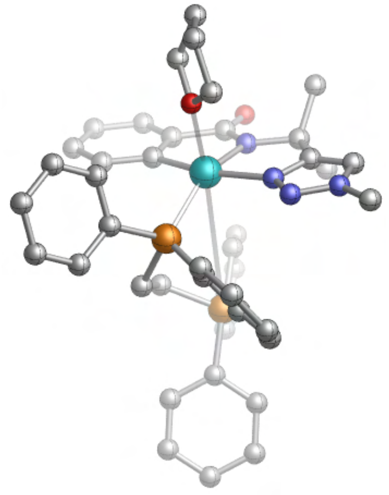

Zero-point correction= 0.798602 (Hartree/Particle)
Thermal correction to Energy= 0.849409
Thermal correction to Enthalpy= 0.850353
Thermal correction to Gibbs Free Energy= 0.705237
Sum of electronic and zero-point Energies= -3979.596288
Sum of electronic and thermal Energies= -3979.545482
Sum of electronic and thermal Enthalpies= -3979.544537
Sum of electronic and thermal Free Energies= -3979.689653

C	-18.88231300	1.24447600	-3.31120600
C	-19.39241600	2.54761600	-3.08324700
C	-20.71877800	2.90015200	-3.35423900
C	-21.59848800	1.94085700	-3.86286200
C	-21.13370100	0.64157900	-4.09467000
C	-19.79774000	0.30538500	-3.82582100
H	-21.03767900	3.92628400	-3.15171800
H	-22.63899100	2.20105900	-4.07769600
H	-21.81545100	-0.11804000	-4.49123500
H	-19.48290800	-0.72407700	-4.02640900
C	-18.44188300	3.55675800	-2.50280800
O	-18.75691600	4.73709300	-2.30049400
C	-16.15418900	3.77991600	-1.63409200
C	-16.53472400	4.26789800	-0.21550400
H	-15.70922300	4.83063500	0.25200000
H	-17.41442700	4.92072900	-0.29061000
H	-16.78147500	3.41148400	0.43153400
C	-15.73996100	4.98731200	-2.51134300
H	-16.59951000	5.65919800	-2.62699100
H	-14.90720600	5.54251200	-2.04767700
H	-15.41705100	4.64594800	-3.50623100
C	-14.98507300	2.82655000	-1.54458000
N	-15.09661500	1.56004300	-2.04787600
N	-13.98481300	0.89562700	-1.87859500
C	-13.70156700	2.93637300	-1.04496200
H	-13.16635000	3.75170600	-0.56684400
N	-13.13247900	1.72055600	-1.27957000

N	-17.23248900	2.98457500	-2.23847600
C	-16.40274500	-0.64727100	0.13483200
C	-18.51997300	0.40661600	-0.00229800
C	-17.20493400	-1.03964600	1.37352200
H	-15.59461900	0.06284100	0.38836400
H	-15.95182600	-1.49675700	-0.40059000
C	-18.20781000	0.11848800	1.46829000
H	-19.38189200	-0.17401200	-0.37072700
H	-18.70586600	1.46699400	-0.21953300
H	-17.73064100	-1.99421200	1.20383600
H	-16.57650700	-1.15405000	2.26971500
H	-19.10904400	-0.12715700	2.04925500
H	-17.73293700	0.99429800	1.94093800
O	-17.34613100	-0.01209300	-0.73696500
Fe	-16.96246600	1.12619300	-2.77158800
P	-16.32246500	-0.60537900	-4.26277000
P	-15.83663900	2.40164600	-6.65300300
C	-16.83102100	-0.27649300	-6.05684500
H	-17.72225400	-0.90828900	-6.19162700
H	-16.05285500	-0.68502100	-6.72123700
C	-17.22135200	1.15733900	-6.44883700
H	-17.88221600	1.58154500	-5.67756700
H	-17.82755200	1.12449900	-7.36929400
C	-17.04050800	-2.29970600	-4.01316400
C	-17.77011500	-2.57348300	-2.84620500
C	-16.87664100	-3.32294900	-4.96541400
C	-18.32501300	-3.84183900	-2.63527300
H	-17.90689100	-1.78254700	-2.10598000
C	-17.43394300	-4.58584700	-4.75610200
H	-16.30079600	-3.13937200	-5.87596900
C	-18.16039500	-4.84850400	-3.58871700
H	-18.89493600	-4.03886400	-1.72338200
H	-17.29959400	-5.36966300	-5.50627000
H	-18.59747000	-5.83720900	-3.42607100
C	-14.51461000	-1.01516300	-4.38659700
C	-13.64317300	-0.18010200	-5.10508200
C	-13.96989600	-2.10655100	-3.68874900
C	-12.27077300	-0.44074200	-5.14323100
H	-14.03185400	0.69657000	-5.62892600
C	-12.59681300	-2.36491500	-3.72278300
H	-14.62044700	-2.76912600	-3.11451700
C	-11.74079000	-1.53514500	-4.45338500
H	-11.61481900	0.21682700	-5.72007800
H	-12.19620500	-3.22599700	-3.18060700
H	-10.66824100	-1.74460600	-4.49038300
C	-15.10791200	1.98765800	-8.30866800
C	-15.71354900	1.16965900	-9.27796000
C	-13.83399100	2.51734100	-8.58921900
C	-15.06848900	0.89337500	-10.48822300
H	-16.70295000	0.74347800	-9.09930100
C	-13.19406100	2.25534800	-9.80317600

H	-13.33958200	3.14547400	-7.84188900
C	-13.80925900	1.43726100	-10.75611400
H	-15.55651700	0.25241800	-11.22751400
H	-12.20835100	2.68431900	-10.00252300
H	-13.30744900	1.22198900	-11.70309500
C	-16.84923000	3.91299100	-7.00434500
C	-17.53284800	4.47637800	-5.91114300
C	-16.94190200	4.54418200	-8.25521500
C	-18.30986500	5.62583200	-6.06430800
H	-17.46107800	4.01228600	-4.92566400
C	-17.70848700	5.70566100	-8.40507900
H	-16.41785000	4.13112600	-9.11984200
C	-18.39654800	6.24672600	-7.31501200
H	-18.83703800	6.02554900	-5.19444700
H	-17.77131100	6.18658400	-9.38532300
H	-18.99758100	7.15143300	-7.43958600
C	-11.79095500	1.26770100	-0.95947200
H	-11.65599300	1.18853900	0.13007000
H	-11.65794400	0.28246100	-1.42222200
H	-11.04789600	1.96763300	-1.36803200

UB3LYP-D3/def2-SVP-SMD(THF)//UB3LYP/def2-SVP(gas)
HF= -3980.5943565
UPBEPBE-D3/def2-SVP-SMD(THF)//UB3LYP/def2-SVP(gas)
HF= -3977.402565540

^{5}A

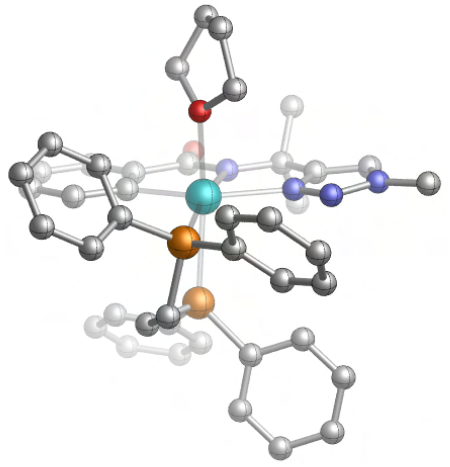

Zero-point correction= 0.797261 (Hartree/Particle)
Thermal correction to Energy= 0.848466
Thermal correction to Enthalpy= 0.849411
Thermal correction to Gibbs Free Energy= 0.704076
Sum of electronic and zero-point Energies= -3979.590437
Sum of electronic and thermal Energies= -3979.539231
Sum of electronic and thermal Enthalpies= -3979.538287
Sum of electronic and thermal Free Energies= -3979.683621

C	-18.93071100	1.52596400	-3.42169400
C	-19.33503400	2.81376300	-2.97054300
C	-20.67965000	3.21186000	-2.95216600
C	-21.68064500	2.33396400	-3.37543000
C	-21.32261300	1.05636700	-3.81854200
C	-19.97232900	0.67277400	-3.84069400
H	-20.91235900	4.22000100	-2.59979300
H	-22.73084800	2.64076600	-3.35821100
H	-22.09632400	0.35428300	-4.14856600
H	-19.74441000	-0.33962200	-4.19628800
C	-18.30059900	3.81352700	-2.48671300
O	-18.63427100	4.95214700	-2.12694600
C	-15.92218800	4.15802800	-2.04994800
C	-16.07589100	4.62062800	-0.57894200
H	-15.21573700	5.23160100	-0.25599400
H	-16.99411500	5.21529800	-0.49292100
H	-16.15210400	3.74912100	0.09063500
C	-15.73854800	5.39431400	-2.96757600
H	-16.64977100	6.00451900	-2.91796500
H	-14.87331900	6.00425600	-2.65660800
H	-15.58489300	5.07396700	-4.00953000
C	-14.68315000	3.28573500	-2.15624700
N	-14.73270100	2.03217100	-2.69287200
N	-13.54951800	1.47871000	-2.68746300
C	-13.36364200	3.50469800	-1.79819200
H	-12.85919700	4.35044100	-1.33968800
N	-12.71049300	2.36335700	-2.14730000
N	-17.03246100	3.31770900	-2.48482700

C	-15.82017800	-0.46817700	-0.58221900
C	-17.85540500	0.70707800	-0.25592900
C	-16.34372000	-0.79751000	0.81468400
H	-14.92120600	0.17155900	-0.53942700
H	-15.57468200	-1.35455500	-1.18743500
C	-17.22831400	0.42128300	1.10881500
H	-18.79378700	0.14810300	-0.40908500
H	-18.05300400	1.76952000	-0.44598300
H	-16.95075800	-1.71797400	0.79070800
H	-15.53517200	-0.94487200	1.54663600
H	-17.98257200	0.23963300	1.88879400
H	-16.60754500	1.27314900	1.43307700
O	-16.88546200	0.24644600	-1.22695500
Fe	-16.83708600	1.43954200	-3.27161100
P	-16.17836000	-1.10856900	-4.47539100
P	-16.67151200	1.99373200	-5.90728200
C	-16.36356800	-0.78205500	-6.31791400
H	-16.71090000	-1.69851100	-6.81960800
H	-15.35738500	-0.57379200	-6.71304700
C	-17.30773600	0.38723900	-6.63152900
H	-18.30287900	0.21419300	-6.19369800
H	-17.43934300	0.48759800	-7.72118300
C	-17.40498000	-2.45652100	-4.15517800
C	-18.22088900	-2.35572400	-3.01652200
C	-17.54537700	-3.57875600	-4.99289100
C	-19.15754200	-3.35390400	-2.72279500
H	-18.12456100	-1.48263000	-2.36802900
C	-18.48211100	-4.57180300	-4.70075800
H	-16.91112000	-3.68959200	-5.87632200
C	-19.29138200	-4.46039400	-3.56374000
H	-19.79181100	-3.25693400	-1.83803900
H	-18.58094700	-5.43694200	-5.36173800
H	-20.02699500	-5.23675500	-3.33753900
C	-14.55802200	-2.00621900	-4.39746900
C	-13.40778200	-1.40611200	-4.94450300
C	-14.40790000	-3.21970600	-3.70215100
C	-12.15749000	-2.01739500	-4.83020800
H	-13.48031300	-0.44743700	-5.46289300
C	-13.15257200	-3.82390500	-3.57766800
H	-15.27883200	-3.70729000	-3.25986100
C	-12.02310900	-3.22986300	-4.14582600
H	-11.28174200	-1.53994400	-5.27825400
H	-13.06192500	-4.77069000	-3.03838800
H	-11.04408900	-3.70757100	-4.05595000
C	-15.15679300	2.25657600	-6.95639600
C	-15.20973700	2.36182700	-8.35902400
C	-13.90404800	2.32600300	-6.32579900
C	-14.04270300	2.52743100	-9.10749600
H	-16.17447400	2.32723600	-8.87193100
C	-12.73277400	2.49408900	-7.07565800
H	-13.84688700	2.24203100	-5.23885500

C	-12.79963700	2.59435900	-8.46663500
H	-14.10229600	2.60849900	-10.19617000
H	-11.76578000	2.54884200	-6.56828300
H	-11.88690000	2.72734200	-9.05342200
C	-17.83309400	3.26045800	-6.57669100
C	-19.21893400	3.02680400	-6.61513700
C	-17.35467600	4.52977900	-6.95170800
C	-20.09592700	4.02738400	-7.04291300
H	-19.63065200	2.07298100	-6.28320900
C	-18.23464800	5.52807800	-7.37567700
H	-16.28423200	4.74470400	-6.91651000
C	-19.60906700	5.27890700	-7.42737200
H	-21.16968700	3.82541500	-7.05853900
H	-17.84220600	6.50722300	-7.66277700
H	-20.29829100	6.06132400	-7.75524300
C	-11.30664000	2.03146600	-1.98848200
H	-11.02774200	2.02848600	-0.92406500
H	-11.15724300	1.02938100	-2.40833900
H	-10.67608600	2.75530500	-2.52583800

UB3LYP-D3/def2-SVP-SMD(THF)//UB3LYP/def2-SVP(gas)
HF= -3980.5905459
UPBEPBE-D3/def2-SVP-SMD(THF)//UB3LYP/def2-SVP(gas)
HF= -3977.381586230

^{1}B

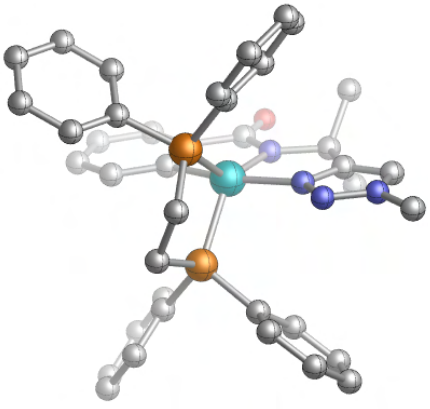

Zero-point correction= 0.681585 (Hartree/Particle)
Thermal correction to Energy= 0.725054
Thermal correction to Enthalpy= 0.725998
Thermal correction to Gibbs Free Energy= 0.601459
Sum of electronic and zero-point Energies= -3747.423459
Sum of electronic and thermal Energies= -3747.379991
Sum of electronic and thermal Enthalpies= -3747.379047
Sum of electronic and thermal Free Energies= -3747.503586

C	-18.55589400	1.52238700	-3.61988400
C	-18.92619200	2.35271200	-2.53405900
C	-20.25305900	2.52972300	-2.12645000
C	-21.27725800	1.87143400	-2.81171000
C	-20.95140600	1.04816100	-3.89621600
C	-19.61555900	0.88037000	-4.28731800
H	-20.45583000	3.18855500	-1.27745600
H	-22.32042300	1.99841800	-2.50818900
H	-21.74519900	0.53042700	-4.44438900
H	-19.41692500	0.22363100	-5.14120500
C	-17.81195100	3.07118800	-1.83685800
O	-17.98714000	3.83845700	-0.88813800
C	-15.37345300	3.38730700	-1.90127400
C	-15.10964100	3.04252600	-0.41527500
H	-14.15379400	3.47275400	-0.07200300
H	-15.92702600	3.44472900	0.19619400
H	-15.06937900	1.95115200	-0.27499100
C	-15.39234100	4.92345100	-2.09119000
H	-16.21428500	5.34639600	-1.49928800
H	-14.44173600	5.37787300	-1.76507400
H	-15.55286000	5.17356700	-3.15119800
C	-14.27854000	2.78915000	-2.75291600
N	-14.61272700	1.89006100	-3.72495100
N	-13.54817100	1.46091300	-4.34918300
C	-12.90332400	2.91499600	-2.79771800
H	-12.19996100	3.50865700	-2.22108100
N	-12.50891200	2.07321500	-3.79443100
N	-16.60232000	2.75975800	-2.41049400
Fe	-16.58778500	1.53809500	-3.89831900

P	-16.31618100	-0.69823000	-4.36950400
P	-16.66138600	1.80857500	-6.18587600
C	-15.79148800	-0.84418000	-6.15940200
H	-15.84538900	-1.87170500	-6.55032900
H	-14.73022900	-0.54787100	-6.16245500
C	-16.62728500	0.12478200	-7.01014800
H	-17.67827200	-0.20464300	-7.05266200
H	-16.26434300	0.16846300	-8.04879800
C	-17.69901800	-1.91438700	-4.17027900
C	-18.36048100	-1.93660000	-2.92954700
C	-18.11583400	-2.80584000	-5.17057000
C	-19.39495600	-2.84140200	-2.68957600
H	-18.07452800	-1.22720900	-2.14940800
C	-19.16200800	-3.70501900	-4.93378200
H	-17.63302400	-2.81294700	-6.14983600
C	-19.80050100	-3.72863200	-3.69225500
H	-19.89731800	-2.84239100	-1.71906600
H	-19.47588200	-4.38965500	-5.72626400
H	-20.61713300	-4.43129400	-3.50752100
C	-14.97152400	-1.57715100	-3.44240800
C	-14.28977100	-2.69191600	-3.95808900
C	-14.65422100	-1.12932700	-2.15004400
C	-13.30768600	-3.33730800	-3.20177300
H	-14.52757100	-3.07242400	-4.95441100
C	-13.68005800	-1.78191000	-1.38825800
H	-15.17487300	-0.25881600	-1.74256200
C	-13.00168500	-2.88499500	-1.91391000
H	-12.78471900	-4.20298400	-3.61716100
H	-13.44983300	-1.42413300	-0.38117800
H	-12.23828800	-3.39509000	-1.32049400
C	-15.20607400	2.70406700	-6.91842200
C	-15.06115800	4.06676300	-6.59881700
C	-14.22867400	2.10142400	-7.72435800
C	-13.97960400	4.80610400	-7.08055700
H	-15.80916300	4.55848000	-5.97070600
C	-13.14189800	2.84069000	-8.20650600
H	-14.29895200	1.04439600	-7.98745500
C	-13.01332600	4.19458400	-7.88784000
H	-13.89281300	5.86607000	-6.82741900
H	-12.39571400	2.35261000	-8.83958700
H	-12.16869200	4.77340500	-8.27080700
C	-18.04321200	2.69625100	-7.03493100
C	-18.28035900	2.55019900	-8.41325900
C	-18.85084700	3.57314900	-6.29432200
C	-19.30974800	3.26009100	-9.03503100
H	-17.65442900	1.88628700	-9.01488400
C	-19.87411400	4.29189100	-6.92129400
H	-18.68891900	3.67906900	-5.22035900
C	-20.10817500	4.13433700	-8.28922300
H	-19.48611700	3.13423700	-10.10662300
H	-20.49712400	4.96782200	-6.33037400

H	-20.91328800	4.69076200	-8.77637100
C	-11.17448300	1.85362300	-4.32181700
H	-10.43772800	1.99394300	-3.51996000
H	-11.11645000	0.82463700	-4.69773900
H	-10.96407000	2.55377700	-5.14483200

UB3LYP-D3/def2-SVP-SMD(THF)//UB3LYP/def2-SVP(gas)
HF= -3748.2815869
UPBEPBE-D3/def2-SVP-SMD(THF)//UB3LYP/def2-SVP(gas)
HF= -3745.423329330

^{3}**B**

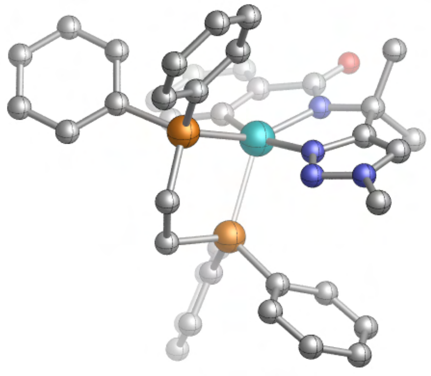

Zero-point correction= 0.680864 (Hartree/Particle)
Thermal correction to Energy= 0.724829
Thermal correction to Enthalpy= 0.725773
Thermal correction to Gibbs Free Energy= 0.598025
Sum of electronic and zero-point Energies= -3747.433476
Sum of electronic and thermal Energies= -3747.389511
Sum of electronic and thermal Enthalpies= -3747.388567
Sum of electronic and thermal Free Energies= -3747.516315

C	-18.88622100	1.51418300	-3.37461100
C	-19.38513200	2.43379900	-2.42274200
C	-20.75423200	2.63832100	-2.21963100
C	-21.67771100	1.92014300	-2.98407900
C	-21.21725200	1.01009900	-3.94362200
C	-19.84138400	0.81242100	-4.13108400
H	-21.06714300	3.36599500	-1.46573600
H	-22.75170600	2.06847600	-2.83780100
H	-21.93457500	0.44793100	-4.55017300
H	-19.52510600	0.09130800	-4.89148200
C	-18.36844700	3.21702600	-1.64417100
O	-18.67499000	4.04886100	-0.78314900
C	-15.95076100	3.54040000	-1.40655900
C	-15.89096400	3.27480900	0.11857100
H	-14.99245300	3.72997800	0.56831800
H	-16.78687400	3.70120500	0.58730500
H	-15.87105000	2.19188900	0.31712900
C	-15.93453900	5.06425700	-1.67813600
H	-16.83280700	5.51082100	-1.23261000
H	-15.03978000	5.54088400	-1.24299600
H	-15.94193200	5.25780500	-2.76183900
C	-14.74899200	2.90179800	-2.06230800
N	-14.93211900	1.90064900	-2.97124500
N	-13.78635700	1.46181600	-3.42248900
C	-13.38214600	3.07987900	-1.95563900
H	-12.77242800	3.75496400	-1.36230900
N	-12.84396500	2.16544200	-2.81110300
N	-17.10446000	2.88488700	-2.03607700
Fe	-16.89324200	1.52608800	-3.40873700
P	-16.46438900	-0.73519100	-4.00202300

P	-16.51865400	1.89536500	-5.89717400
C	-15.58191300	-0.75330100	-5.65153800
H	-15.49298300	-1.76735900	-6.07207300
H	-14.56208500	-0.40688400	-5.41986000
C	-16.26745100	0.19705900	-6.64747500
H	-17.28147500	-0.16136600	-6.88906200
H	-15.71526400	0.24339800	-7.60017300
C	-17.80863600	-1.99710800	-4.17742400
C	-18.64508200	-2.20268400	-3.06512300
C	-18.04956600	-2.73910700	-5.34432700
C	-19.67838900	-3.13859600	-3.11256400
H	-18.49332100	-1.61468700	-2.15661800
C	-19.09412100	-3.66972800	-5.39553900
H	-17.42740300	-2.60379200	-6.23119400
C	-19.90793500	-3.87516000	-4.27986000
H	-20.31645800	-3.28384500	-2.23726000
H	-19.26819000	-4.23608300	-6.31433900
H	-20.72298600	-4.60228800	-4.32018700
C	-15.29744700	-1.65312700	-2.88865000
C	-14.58711900	-2.79161200	-3.30606800
C	-15.15174300	-1.21380900	-1.56354300
C	-13.74242400	-3.46666300	-2.42145800
H	-14.69827500	-3.16809500	-4.32600100
C	-14.31533100	-1.89618700	-0.67407900
H	-15.69998000	-0.32849600	-1.23111400
C	-13.60556000	-3.02101000	-1.10218000
H	-13.19553900	-4.35048400	-2.76086300
H	-14.21865900	-1.54515100	0.35676900
H	-12.95020200	-3.55451300	-0.40860600
C	-14.97275200	2.82105900	-6.32934500
C	-14.94246500	4.19545900	-6.02360200
C	-13.82119300	2.23558600	-6.87854000
C	-13.80385900	4.96211500	-6.27593300
H	-15.82569500	4.67211500	-5.58882500
C	-12.67695500	3.00308200	-7.12841100
H	-13.80107200	1.17151600	-7.12149900
C	-12.66379300	4.36788600	-6.83024900
H	-13.80716200	6.02954800	-6.04009100
H	-11.79456800	2.52870100	-7.56696000
H	-11.77299500	4.96817800	-7.03313000
C	-17.73081100	2.68380000	-7.04957200
C	-17.52446800	2.71173200	-8.44136300
C	-18.88331600	3.28338800	-6.51833800
C	-18.45800900	3.31737200	-9.28383500
H	-16.62243300	2.26906500	-8.87247500
C	-19.81489500	3.89569300	-7.36520800
H	-19.05683900	3.26140600	-5.44087000
C	-19.60632400	3.91110600	-8.74567800
H	-18.28730800	3.33216900	-10.36364800
H	-20.70866000	4.35675000	-6.93759600
H	-20.33600700	4.38750600	-9.40586400

C	-11.44738600	1.95672300	-3.15044300
H	-11.33081200	0.92271300	-3.49691600
H	-11.13835300	2.64691800	-3.95017500
H	-10.82547100	2.11926500	-2.26013100

UB3LYP-D3/def2-SVP-SMD(THF)//UB3LYP/def2-SVP(gas)
HF= -3748.2916728
UPBEPBE-D3/def2-SVP-SMD(THF)//UB3LYP/def2-SVP(gas)
HF= -3745.414197640

^{5}B

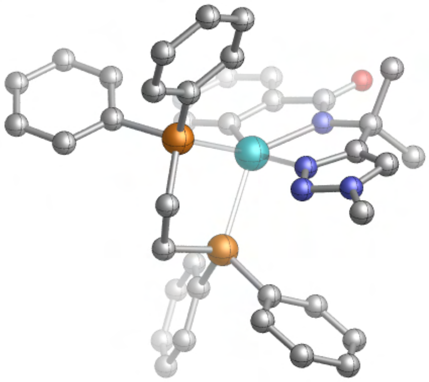

Zero-point correction= 0.679786 (Hartree/Particle)
Thermal correction to Energy= 0.724344
Thermal correction to Enthalpy= 0.725288
Thermal correction to Gibbs Free Energy= 0.594892
Sum of electronic and zero-point Energies= -3747.426246
Sum of electronic and thermal Energies= -3747.381688
Sum of electronic and thermal Enthalpies= -3747.380744
Sum of electronic and thermal Free Energies= -3747.511140

C	-19.21064300	1.71884100	-3.17863700
C	-19.41572600	2.61675200	-2.09972700
C	-20.70049800	2.91756700	-1.62344900
C	-21.82107700	2.32727100	-2.21135600
C	-21.65189400	1.43543400	-3.27667600
C	-20.36361600	1.14191600	-3.74558700
H	-20.79173200	3.61847300	-0.79015300
H	-22.82429800	2.55972300	-1.84161100
H	-22.52560400	0.96700900	-3.74224400
H	-20.27042700	0.43764200	-4.58122900
C	-18.23445200	3.28437500	-1.41810500
O	-18.42003000	4.08487700	-0.49193000
C	-15.80951000	3.48701300	-1.33285100
C	-15.66843700	3.14464800	0.17286400
H	-14.72502800	3.54006600	0.58573200
H	-16.51417200	3.58182800	0.71777500
H	-15.68043000	2.05296000	0.31850500
C	-15.74361900	5.02279400	-1.52955500
H	-16.59495000	5.48260600	-1.01187600
H	-14.80572400	5.44033800	-1.12555200
H	-15.80152200	5.27061800	-2.60088700
C	-14.64822100	2.85341900	-2.07359800
N	-14.84497700	1.92463000	-3.04759500
N	-13.70428000	1.50944200	-3.52770900
C	-13.27706400	3.01421200	-1.95743200
H	-12.66138400	3.64038300	-1.31826500
N	-12.74676200	2.15996800	-2.87392600
N	-17.02054100	2.91396700	-1.91871700
Fe	-17.16455800	1.63030100	-3.48549900
P	-16.43399400	-0.80998200	-4.01683800

P	-16.49595500	1.91183600	-5.98967200
C	-15.39461300	-0.68043400	-5.56220100
H	-15.14547900	-1.66947600	-5.97980800
H	-14.45165800	-0.22231600	-5.22413200
C	-16.08600100	0.19574900	-6.62186300
H	-17.05820600	-0.23924500	-6.90633900
H	-15.48994700	0.24859400	-7.54746400
C	-17.74586500	-2.04335300	-4.43319300
C	-18.85649500	-2.10577400	-3.57215800
C	-17.69558000	-2.91450000	-5.53468000
C	-19.87822100	-3.03160400	-3.79549500
H	-18.92998400	-1.41267000	-2.73065600
C	-18.72468800	-3.83358900	-5.76277700
H	-16.85209400	-2.88791100	-6.22799400
C	-19.81548600	-3.89751700	-4.89150800
H	-20.73365200	-3.06462000	-3.11624700
H	-18.67153000	-4.50253700	-6.62582000
H	-20.61930400	-4.61615500	-5.07084600
C	-15.36414100	-1.74065200	-2.83193400
C	-14.71018100	-2.93771800	-3.17036500
C	-15.22719900	-1.23401500	-1.52967900
C	-13.92611800	-3.60704600	-2.22816300
H	-14.82125300	-3.36236900	-4.17156900
C	-14.44793300	-1.90916100	-0.58436400
H	-15.73667700	-0.30593700	-1.25808200
C	-13.79395400	-3.09357500	-0.93281400
H	-13.42283900	-4.53796200	-2.50264500
H	-14.35469100	-1.50679200	0.42767700
H	-13.18533800	-3.62214800	-0.19437600
C	-15.00844400	2.93976500	-6.36841200
C	-15.08491900	4.30952700	-6.05235800
C	-13.81111500	2.44051900	-6.90318500
C	-14.00202100	5.15845100	-6.28584800
H	-16.00422500	4.71617900	-5.62122100
C	-12.72332100	3.29130600	-7.13322200
H	-13.71036200	1.38127000	-7.14675200
C	-12.81548400	4.65158400	-6.82850800
H	-14.08455000	6.22045400	-6.04091900
H	-11.80168300	2.88565800	-7.55955800
H	-11.96763800	5.31615100	-7.01396600
C	-17.71258100	2.51276000	-7.24073800
C	-17.36097200	2.71689000	-8.58777500
C	-19.03238600	2.76036400	-6.83105000
C	-18.31658700	3.15024000	-9.50834500
H	-16.33270700	2.54812300	-8.91867800
C	-19.98731100	3.19547200	-7.75734600
H	-19.31432700	2.61343000	-5.78543300
C	-19.63258500	3.38941000	-9.09402900
H	-18.03332100	3.30730600	-10.55252700
H	-21.01080200	3.38546400	-7.42463500
H	-20.37875400	3.73232600	-9.81568800

C	-11.35361000	1.95896800	-3.22751700
H	-10.73199300	2.00202200	-2.32294600
H	-11.25931900	0.96897600	-3.69007000
H	-11.01968000	2.72767600	-3.94128500

UB3LYP-D3/def2-SVP-SMD(THF)//UB3LYP/def2-SVP(gas)
HF= -3748.2794955
UPBEPBE-D3/def2-SVP-SMD(THF)//UB3LYP/def2-SVP(gas)
HF= -3745.3772034

^{1}B'

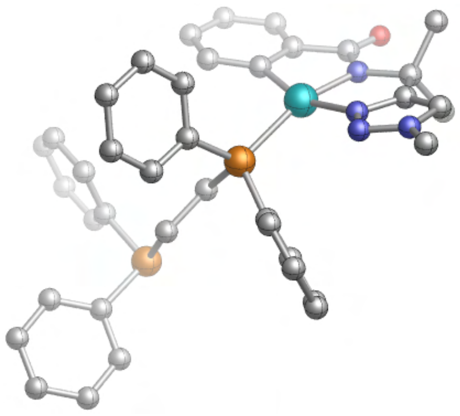

Zero-point correction= 0.681248 (Hartree/Particle)
Thermal correction to Energy= 0.725416
Thermal correction to Enthalpy= 0.726360
Thermal correction to Gibbs Free Energy= 0.596836
Sum of electronic and zero-point Energies= -3747.404446
Sum of electronic and thermal Energies= -3747.360277
Sum of electronic and thermal Enthalpies= -3747.359333
Sum of electronic and thermal Free Energies= -3747.488858

C	-16.85673400	0.62388900	-3.45228800
C	-17.72503400	0.55752800	-2.33483900
C	-18.28391300	-0.64026600	-1.88001400
C	-17.96493500	-1.83763800	-2.52659400
C	-17.08372800	-1.81737100	-3.61281800
C	-16.54443900	-0.60367500	-4.06602700
H	-18.95064400	-0.61109500	-1.01387700
H	-18.39179300	-2.78396500	-2.18209400
H	-16.81854900	-2.75188100	-4.11725700
H	-15.85629900	-0.64300900	-4.91803100
C	-17.97686200	1.84230300	-1.59858000
O	-18.71629000	1.92621800	-0.61577200
C	-17.23415900	4.18805400	-1.52130100
C	-16.71794900	4.13244100	-0.06336300
H	-16.65383500	5.14284400	0.37430700
H	-17.40650300	3.52430800	0.53657400
H	-15.71933300	3.67020800	-0.03078000
C	-18.62666800	4.86251300	-1.56996700
H	-19.33373300	4.24975200	-0.99569600
H	-18.59628600	5.87863600	-1.14194900
H	-18.98075400	4.92967100	-2.61026900
C	-16.25691100	4.98266000	-2.35617300
N	-15.70818600	4.41480500	-3.47187800
N	-14.86776300	5.23235800	-4.04867400
C	-15.71071400	6.24766200	-2.25912400
H	-15.84492900	7.05470300	-1.54473900
N	-14.86507300	6.34287100	-3.32504100
N	-17.24976600	2.86203900	-2.15212100

Fe	-16.35665300	2.51760100	-3.79731100
P	-15.99787400	2.35321200	-6.10019300
C	-17.25489800	1.13824600	-8.45321800
H	-17.23003900	2.11783700	-8.95779500
H	-16.34914400	0.59920900	-8.77096700
C	-17.30482400	1.30712600	-6.93394500
H	-17.25858200	0.33813800	-6.41575800
H	-18.26545800	1.74693100	-6.61679700
C	-14.37752600	1.63703700	-6.62762700
C	-13.89778200	1.66871800	-7.95078900
C	-13.59078400	1.01981200	-5.63954900
C	-12.66914100	1.08769300	-8.27437000
H	-14.47735900	2.16230500	-8.73310800
C	-12.36175800	0.43687200	-5.96517400
H	-13.94855300	0.98810300	-4.60701500
C	-11.89971200	0.46869300	-7.28297200
H	-12.31027100	1.11996000	-9.30644800
H	-11.76546500	-0.04070400	-5.18370700
H	-10.93856700	0.01539500	-7.53916600
C	-16.10896800	3.96267100	-7.00926500
C	-17.37153700	4.50378600	-7.31344200
C	-14.96703800	4.72308000	-7.31281400
C	-17.48747900	5.75584800	-7.92389200
H	-18.28224500	3.94949500	-7.07464800
C	-15.08324000	5.97315700	-7.92731300
H	-13.97548300	4.34018500	-7.06606500
C	-16.34281000	6.49380600	-8.23762700
H	-18.47886900	6.15290100	-8.15694000
H	-14.18077000	6.54228300	-8.16707200
H	-16.43260600	7.46949000	-8.72220600
C	-14.00030700	7.44983100	-3.69413200
H	-14.57324900	8.38772000	-3.69892000
H	-13.16041500	7.53764800	-2.98842800
H	-13.61555400	7.24914000	-4.70077100
P	-18.79821400	0.30676700	-9.13843400
C	-18.36028500	0.32746300	-10.94649200
C	-18.87037000	1.39088600	-11.71280600
C	-17.56378300	-0.63787500	-11.58564500
C	-18.57656100	1.50011000	-13.07504300
H	-19.51248400	2.13767500	-11.23581100
C	-17.27772300	-0.53498200	-12.95032600
H	-17.16696400	-1.48110100	-11.01559600
C	-17.77980900	0.53489500	-13.69793800
H	-18.98107300	2.33499200	-13.65360700
H	-16.65956400	-1.29706400	-13.43280800
H	-17.55649100	0.61200800	-14.76528400
C	-18.56341600	-1.47670400	-8.69125400
C	-19.70151000	-2.29943700	-8.78493500
C	-17.36104500	-2.05254600	-8.24619600
C	-19.63644300	-3.65761300	-8.46661900
H	-20.65244000	-1.86523000	-9.10767600

C	-17.29741000	-3.41004500	-7.91319300
H	-16.45679200	-1.44726400	-8.15353000
C	-18.43258900	-4.21682400	-8.02634100
H	-20.53199300	-4.27890200	-8.55025800
H	-16.35373600	-3.83731100	-7.56370100
H	-18.38171000	-5.27677000	-7.76433900

UB3LYP-D3/def2-SVP-SMD(THF)//UB3LYP/def2-SVP(gas)
HF= -3748.2209057
UPBEPBE-D3/def2-SVP-SMD(THF)//UB3LYP/def2-SVP(gas)
HF= -3745.3398643

^{3}B'

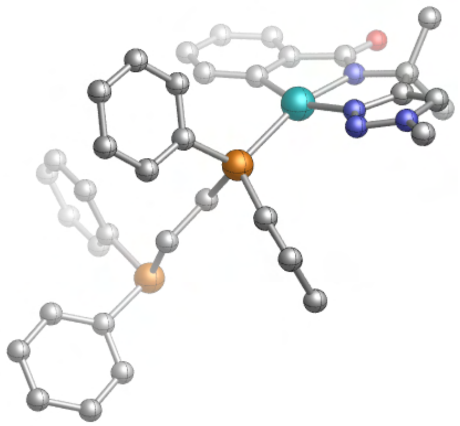

Zero-point correction= 0.681351 (Hartree/Particle)
Thermal correction to Energy= 0.725488
Thermal correction to Enthalpy= 0.726432
Thermal correction to Gibbs Free Energy= 0.596143
Sum of electronic and zero-point Energies= -3747.424221
Sum of electronic and thermal Energies= -3747.380085
Sum of electronic and thermal Enthalpies= -3747.379140
Sum of electronic and thermal Free Energies= -3747.509429

C	-16.79660700	0.63883600	-3.44025100
C	-17.58713900	0.55823900	-2.26875800
C	-18.10266700	-0.64769700	-1.78518700
C	-17.81806200	-1.83813500	-2.45986100
C	-17.01281400	-1.80273200	-3.60274100
C	-16.51444800	-0.58105200	-4.08166200
H	-18.70986600	-0.62895400	-0.87606500
H	-18.21269600	-2.79039700	-2.09393200
H	-16.77373700	-2.73125000	-4.13071500
H	-15.88173800	-0.61040200	-4.97571800
C	-17.80667800	1.83605200	-1.51014300
O	-18.48262000	1.90179000	-0.48083400
C	-17.12175100	4.19677500	-1.47399300
C	-16.50942000	4.16075400	-0.05308200
H	-16.45368400	5.17249700	0.38270900
H	-17.13533400	3.52748400	0.58817300
H	-15.49462300	3.73526100	-0.08702300
C	-18.53598100	4.82571400	-1.43076700
H	-19.18385100	4.19207100	-0.81121900
H	-18.50998900	5.84328400	-1.00576500
H	-18.95975600	4.87911300	-2.44549400
C	-16.23087600	5.02408500	-2.37050200
N	-15.73514300	4.47562000	-3.51956000
N	-14.96777600	5.32422800	-4.15065700
C	-15.72990300	6.31015800	-2.30967800
H	-15.84892000	7.11351400	-1.58850000
N	-14.96188300	6.43593600	-3.42976100
N	-17.13414000	2.86931000	-2.10197300
Fe	-16.32107200	2.54542900	-3.80130200

P	-16.01936200	2.36825800	-6.12863600
C	-17.29315200	1.12389800	-8.45440200
H	-17.26492700	2.09562100	-8.97382200
H	-16.39081600	0.57564400	-8.76617700
C	-17.33766300	1.31662500	-6.93770200
H	-17.29601600	0.35594200	-6.40440200
H	-18.29406600	1.76854300	-6.62490200
C	-14.40349800	1.64298100	-6.65479400
C	-13.93282400	1.65491900	-7.98157800
C	-13.61006200	1.04032100	-5.66281700
C	-12.70601300	1.06979300	-8.30463300
H	-14.51784300	2.13620100	-8.76741600
C	-12.38259000	0.45401800	-5.98804300
H	-13.96149700	1.02112600	-4.62778000
C	-11.92929700	0.46666700	-7.30918400
H	-12.35436000	1.08651000	-9.33953900
H	-11.78102100	-0.01164800	-5.20348000
H	-10.96945900	0.01037400	-7.56494000
C	-16.13579300	3.96506500	-7.05928000
C	-17.40003800	4.50590200	-7.35666800
C	-14.99521800	4.71652000	-7.38882600
C	-17.51913000	5.74842400	-7.98562900
H	-18.30984200	3.95920700	-7.09786200
C	-15.11442200	5.95666100	-8.02243700
H	-14.00189300	4.33488100	-7.14727300
C	-16.37592800	6.47694500	-8.32565400
H	-18.51197700	6.14525600	-8.21277100
H	-14.21280100	6.51805800	-8.28276500
H	-16.46835900	7.44470400	-8.82539900
C	-14.16883400	7.57537200	-3.85700700
H	-14.77394600	8.49149600	-3.80864000
H	-13.27956700	7.68991800	-3.21886200
H	-13.85839900	7.39415700	-4.89264600
P	-18.84151200	0.28963300	-9.12466800
C	-18.40361000	0.28057100	-10.93286000
C	-18.91321800	1.33122400	-11.71685300
C	-17.60609600	-0.69472500	-11.55547300
C	-18.61807800	1.41832400	-13.08043400
H	-19.55581200	2.08565000	-11.25270800
C	-17.31854700	-0.61389300	-12.92129700
H	-17.20963300	-1.52860600	-10.97159500
C	-17.82026600	0.44350300	-13.68673100
H	-19.02224200	2.24357500	-13.67288500
H	-16.69955100	-1.38347100	-13.39062800
H	-17.59575200	0.50340700	-14.75492900
C	-18.61505700	-1.48809500	-8.65093200
C	-19.75694700	-2.30675500	-8.73339700
C	-17.41554900	-2.06314600	-8.19722200
C	-19.69840900	-3.66051100	-8.39564000
H	-20.70564500	-1.87281000	-9.06299600
C	-17.35842900	-3.41606000	-7.84487000

H	-16.50840300	-1.46098400	-8.11289700
C	-18.49735400	-4.21900900	-7.94690900
H	-20.59682100	-4.27873400	-8.47084100
H	-16.41689800	-3.84275100	-7.48894900
H	-18.45152600	-5.27530800	-7.66972000

UB3LYP-D3/def2-SVP-SMD(THF)//UB3LYP/def2-SVP(gas)
HF= -3748.2702559
UPBEPBE-D3/def2-SVP-SMD(THF)//UB3LYP/def2-SVP(gas)
HF= -3745.3828484

[5]B'

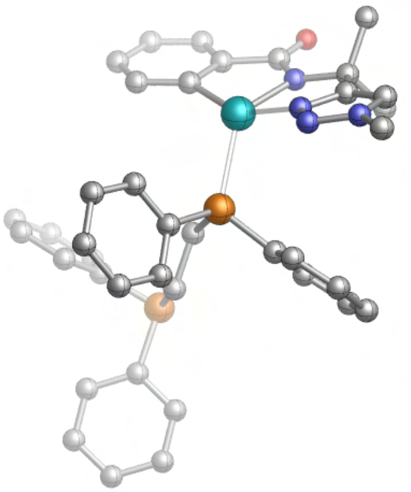

Zero-point correction= 0.679287 (Hartree/Particle)
Thermal correction to Energy= 0.724288
Thermal correction to Enthalpy= 0.725232
Thermal correction to Gibbs Free Energy= 0.589713
Sum of electronic and zero-point Energies= -3747.415619
Sum of electronic and thermal Energies= -3747.370618
Sum of electronic and thermal Enthalpies= -3747.369674
Sum of electronic and thermal Free Energies= -3747.505193

C	-16.74656600	0.22585400	-3.17327600
C	-17.90060700	0.46447300	-2.37408900
C	-18.72844800	-0.58642100	-1.95631400
C	-18.42884300	-1.90158800	-2.31951100
C	-17.29950900	-2.16417100	-3.10270800
C	-16.47765300	-1.10915400	-3.52226200
H	-19.59836600	-0.34563700	-1.34009500
H	-19.07293200	-2.72282000	-1.99147600
H	-17.06000100	-3.19325000	-3.38926800
H	-15.60325900	-1.35092800	-4.13934300
C	-18.24766600	1.87435700	-1.91167500
O	-19.21955900	2.08744900	-1.18037600
C	-17.38004900	4.18801600	-1.89627000
C	-17.35469000	4.27843500	-0.34975900
H	-17.30348400	5.32827500	-0.01613900
H	-18.26279100	3.81170700	0.05136000
H	-16.47975800	3.74081200	0.04717100
C	-18.59067100	4.97415200	-2.45187700
H	-19.51014600	4.49968000	-2.08199700
H	-18.57747300	6.02956700	-2.13049100
H	-18.59430800	4.94268100	-3.55246600
C	-16.08262300	4.78544400	-2.42465100
N	-15.19490600	4.03289700	-3.14338300
N	-14.14564400	4.73558100	-3.47411400
C	-15.51555300	6.04213100	-2.31178700
H	-15.84788200	6.95585100	-1.82753800
N	-14.32768300	5.95264200	-2.97090000

N	-17.37199500	2.81136100	-2.37238400
Fe	-16.05144900	2.07659500	-3.65197900
P	-15.87645000	2.36088800	-6.15592200
C	-17.22093800	1.19627200	-8.47050500
H	-17.28861600	2.19707000	-8.92704500
H	-16.29945400	0.73817900	-8.86209000
C	-17.18520400	1.29178900	-6.94466900
H	-17.03735500	0.30119200	-6.48395200
H	-18.14661700	1.64884500	-6.53747700
C	-14.29474200	1.77665100	-6.90279500
C	-13.85557500	2.13074300	-8.19142500
C	-13.50782100	0.89983500	-6.13565300
C	-12.65933800	1.61555700	-8.69736700
H	-14.44474700	2.81849500	-8.80187900
C	-12.31502800	0.37909900	-6.64636300
H	-13.83182700	0.62155900	-5.12826500
C	-11.88829700	0.73762800	-7.92768400
H	-12.32891700	1.89969200	-9.69985900
H	-11.71610500	-0.30196200	-6.03664200
H	-10.95309600	0.33647100	-8.32675600
C	-16.14152500	4.05144000	-6.83474900
C	-17.42574100	4.49933600	-7.19488700
C	-15.07595600	4.97129000	-6.86785800
C	-17.63421000	5.82228300	-7.59685600
H	-18.27932900	3.81911800	-7.16263300
C	-15.28718900	6.29070800	-7.27489300
H	-14.07391100	4.65528400	-6.56970900
C	-16.56599200	6.72160200	-7.64319000
H	-18.63927800	6.14782500	-7.87693500
H	-14.44454200	6.98689000	-7.30740300
H	-16.72907200	7.75371200	-7.96345400
C	-13.32997600	6.98584100	-3.18195900
H	-13.71947000	7.76859300	-3.85021900
H	-13.04011200	7.43621400	-2.22179200
H	-12.45562100	6.51150600	-3.64322300
P	-18.75280800	0.28764400	-9.08917300
C	-18.40745700	0.33394000	-10.91461300
C	-18.68086100	1.54345600	-11.58213200
C	-17.95172900	-0.75728400	-11.67244000
C	-18.47567300	1.66842500	-12.95774900
H	-19.07021800	2.39748800	-11.01865000
C	-17.75983800	-0.63656400	-13.05325200
H	-17.74577000	-1.71227000	-11.18522200
C	-18.01494600	0.57550100	-13.69957300
H	-18.68945400	2.61853100	-13.45477600
H	-17.40698700	-1.49861000	-13.62597800
H	-17.86369900	0.66711300	-14.77825300
C	-18.40056800	-1.47197600	-8.62901700
C	-19.51246300	-2.28937100	-8.35911800
C	-17.11546800	-2.03069300	-8.50884300
C	-19.34889600	-3.62930600	-7.99626300

H	-20.51819600	-1.86509600	-8.42749400
C	-16.94940200	-3.36779000	-8.13668300
H	-16.22904900	-1.42383900	-8.70774300
C	-18.06578900	-4.17092100	-7.88263200
H	-20.22622100	-4.24780700	-7.79010300
H	-15.94269500	-3.78379600	-8.04354800
H	-17.93439400	-5.21549100	-7.58870100

UB3LYP-D3/def2-SVP-SMD(THF)//UB3LYP/def2-SVP(gas)
HF= -3748.2608253
UPBEPBE-D3/def2-SVP-SMD(THF)//UB3LYP/def2-SVP(gas)
HF= -3745.3570435

^{1}B-TS

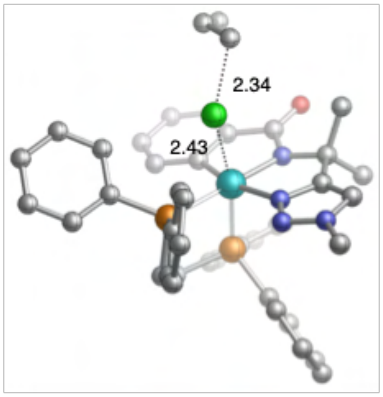

Zero-point correction= 0.753150 (Hartree/Particle)
Thermal correction to Energy= 0.802457
Thermal correction to Enthalpy= 0.803401
Thermal correction to Gibbs Free Energy= 0.668062
Sum of electronic and zero-point Energies= -4324.614500
Sum of electronic and thermal Energies= -4324.565193
Sum of electronic and thermal Enthalpies= -4324.564249
Sum of electronic and thermal Free Energies= -4324.699588

C	-18.68031300	1.07333600	-3.91915300
C	-19.12508800	2.11684600	-3.07071100
C	-20.47440900	2.46007100	-2.92902700
C	-21.44861000	1.76870000	-3.65361500
C	-21.04729400	0.74102000	-4.51166500
C	-19.68985200	0.40530400	-4.63692200
H	-20.73217500	3.28003600	-2.25322600
H	-22.50611700	2.03135300	-3.55826900
H	-21.79492700	0.19019900	-5.09199200
H	-19.44234300	-0.41334600	-5.31865600
C	-18.07974700	2.87388500	-2.31665400
O	-18.35680500	3.73943300	-1.46903200
C	-15.65238200	3.03475300	-1.98568500
C	-15.71583600	2.86515700	-0.44518500
H	-14.80660600	3.27561700	0.02460900
H	-16.59565800	3.39207000	-0.05674900
H	-15.78185100	1.79869800	-0.18931900
C	-15.43822500	4.52751700	-2.32934100
H	-16.32750400	5.09203700	-2.01777100
H	-14.55860600	4.93565000	-1.80401200
H	-15.27924500	4.66198200	-3.40987800
C	-14.49151400	2.21187600	-2.48647700
N	-14.72113700	1.14286400	-3.30139200
N	-13.60477300	0.52698800	-3.57588100
C	-13.13052200	2.24300200	-2.25002700
H	-12.50210900	2.90309100	-1.65919500
N	-12.63503000	1.18134100	-2.94279800
N	-16.82900700	2.48059900	-2.67711200
Fe	-16.68671200	0.88663000	-3.78374700
P	-16.39029900	-1.12180200	-5.07406900

P	-16.70386800	1.98132100	-5.83088500
C	-16.29585700	-0.57695700	-6.86847400
H	-16.60051600	-1.38335500	-7.55167800
H	-15.24198500	-0.35154600	-7.08620600
C	-17.13903000	0.67919900	-7.09865300
H	-18.20852900	0.46045300	-6.96445000
H	-16.99203200	1.06823700	-8.11822100
C	-17.62372300	-2.51919800	-5.11525500
C	-18.58859900	-2.65325800	-4.10492500
C	-17.56516500	-3.49481900	-6.12746300
C	-19.48215500	-3.73036300	-4.11876800
H	-18.63555400	-1.91687600	-3.30312200
C	-18.46146200	-4.56508200	-6.14271400
H	-16.80222500	-3.43804800	-6.90801000
C	-19.42634600	-4.68422300	-5.13674100
H	-20.22962300	-3.81701000	-3.32588900
H	-18.40106600	-5.31196800	-6.93879900
H	-20.12832200	-5.52230100	-5.14595800
C	-14.84279700	-2.13201900	-4.85445700
C	-14.58908500	-2.66761800	-3.57870300
C	-13.93272800	-2.40599800	-5.88753500
C	-13.45805400	-3.45125900	-3.34634300
H	-15.27772600	-2.45375400	-2.75834600
C	-12.79572600	-3.18841500	-5.65231500
H	-14.09418600	-2.01879000	-6.89490800
C	-12.55355100	-3.71366400	-4.38147800
H	-13.28112200	-3.85885700	-2.34727600
H	-12.10210500	-3.39183200	-6.47285400
H	-11.66886500	-4.32975700	-4.19936300
C	-15.16285000	2.71586000	-6.58980400
C	-15.17807500	3.91565100	-7.32443400
C	-13.93958000	2.03043300	-6.46414600
C	-14.01129700	4.41040600	-7.91534100
H	-16.10831400	4.47303100	-7.44107700
C	-12.77623200	2.52107200	-7.06515900
H	-13.88183700	1.11155800	-5.87992000
C	-12.80611500	3.71465300	-7.79115600
H	-14.04922200	5.34612800	-8.47945200
H	-11.83991200	1.96588900	-6.95988200
H	-11.89556300	4.10148700	-8.25617900
C	-17.93478300	3.32404300	-6.13025100
C	-18.86007200	3.27936400	-7.18582800
C	-17.93270200	4.44800500	-5.28641800
C	-19.75573800	4.33300500	-7.39412800
H	-18.89870200	2.42335300	-7.86010900
C	-18.82500900	5.50074700	-5.49748400
H	-17.23730300	4.49644500	-4.44955600
C	-19.74037900	5.44719000	-6.55256600
H	-20.47100400	4.27659200	-8.21882200
H	-18.81165700	6.35972200	-4.82221600
H	-20.44383600	6.26811800	-6.71375700

C	-20.04619800	0.86224400	0.04851700
H	-21.13354400	0.83952900	-0.05454600
H	-19.56668800	1.84601000	0.06827400
C	-19.32341000	-0.28686600	0.10907500
H	-19.84420900	-1.24880900	0.03912900
C	-17.90305900	-0.31977400	0.24231900
H	-17.38234700	0.61188600	0.46657100
H	-17.41679700	-1.23523700	0.58160300
Cl	-16.91253400	-0.59110100	-1.86428000
C	-11.25810600	0.74358600	-3.07743700
H	-10.69039800	1.43443800	-3.71972800
H	-10.78087300	0.69078300	-2.08854200
H	-11.27118600	-0.25450800	-3.53195200

UB3LYP-D3/def2-SVP-SMD(THF)//UB3LYP/def2-SVP(gas)
HF= -4325.5696287
UPBEPBE-D3/def2-SVP-SMD(THF)//UB3LYP/def2-SVP(gas)
HF= -4322.3476259

^{3}B-TS

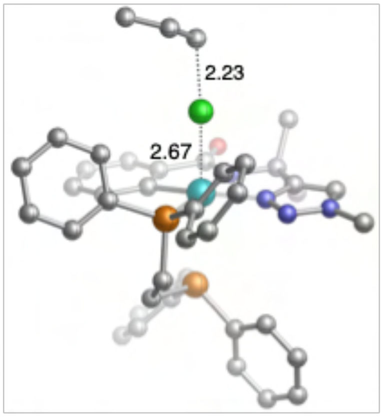

Zero-point correction= 0.751036 (Hartree/Particle)
Thermal correction to Energy= 0.801682
Thermal correction to Enthalpy= 0.802626
Thermal correction to Gibbs Free Energy= 0.658418
Sum of electronic and zero-point Energies= -4324.626370
Sum of electronic and thermal Energies= -4324.575724
Sum of electronic and thermal Enthalpies= -4324.574780
Sum of electronic and thermal Free Energies= -4324.718988

C	-18.81062600	1.42740200	-3.76710700
C	-19.28750400	2.57149000	-3.08964500
C	-20.63649200	2.93747100	-3.08996900
C	-21.56076300	2.15881000	-3.79025000
C	-21.11845500	1.02403400	-4.47774500
C	-19.76253100	0.66287200	-4.46114500
H	-20.93281300	3.83336500	-2.53767600
H	-22.61981500	2.43193900	-3.80183300
H	-21.83421300	0.40573700	-5.02858000
H	-19.47468800	-0.24528600	-4.99381400
C	-18.27347600	3.40300900	-2.36902600
O	-18.56333800	4.41132600	-1.71928400
C	-15.86768500	3.53941800	-1.89372600
C	-15.99534800	3.49346200	-0.35075100
H	-15.10231400	3.92503800	0.13183000
H	-16.87981800	4.06887000	-0.04881100
H	-16.11498400	2.45206100	-0.01949800
C	-15.66477500	4.99732800	-2.37327900
H	-16.55670900	5.58282700	-2.11731900
H	-14.78462000	5.45400200	-1.89026700
H	-15.51503900	5.02689300	-3.46384300
C	-14.67339900	2.71802500	-2.31279300
N	-14.84769400	1.59758100	-3.06837200
N	-13.70089500	1.03795900	-3.34616900
C	-13.31184800	2.83927000	-2.10650600
H	-12.71178200	3.56575700	-1.56645300
N	-12.76627700	1.77783800	-2.76285400
N	-17.01639000	2.89986400	-2.55558100
Fe	-16.84063800	1.21803600	-3.47998900

P	-16.49855300	-0.95064800	-4.67167700
P	-16.52207800	2.17417500	-6.20735600
C	-16.12261100	-0.59687600	-6.47114900
H	-16.25321000	-1.50704100	-7.07523000
H	-15.04632500	-0.36562200	-6.50595300
C	-16.93618900	0.56021400	-7.06665600
H	-18.01694800	0.39996500	-6.92503000
H	-16.74649200	0.63110900	-8.14971300
C	-17.87655600	-2.19096200	-4.71940200
C	-18.39939100	-2.61975600	-3.48558100
C	-18.41486500	-2.72101000	-5.90309800
C	-19.42186100	-3.56899800	-3.44255300
H	-18.01775700	-2.19490900	-2.55438500
C	-19.44596400	-3.66578000	-5.85548000
H	-18.04703400	-2.40254900	-6.88006500
C	-19.94930600	-4.09578300	-4.62587400
H	-19.81234800	-3.88854100	-2.47311700
H	-19.85546400	-4.06469800	-6.78733400
H	-20.75429200	-4.83440300	-4.58995500
C	-15.07548900	-2.05745100	-4.21278600
C	-14.53115100	-2.96078500	-5.14361100
C	-14.55195500	-2.03386700	-2.91066600
C	-13.47998800	-3.80868400	-4.78646800
H	-14.93013900	-3.02235100	-6.15809800
C	-13.50629900	-2.89148900	-2.55274300
H	-14.97689100	-1.35478000	-2.17000000
C	-12.96314900	-3.77605400	-3.48763100
H	-13.07012000	-4.50324000	-5.52457400
H	-13.11631100	-2.86631200	-1.53144900
H	-12.14536400	-4.44449300	-3.20512000
C	-14.99522200	2.69605000	-7.13219000
C	-15.00450500	3.60573500	-8.20448300
C	-13.75800100	2.16702200	-6.71494000
C	-13.81693700	3.95811500	-8.85293000
H	-15.94620500	4.04620700	-8.53754100
C	-12.57390800	2.50838800	-7.37557200
H	-13.71479700	1.49225900	-5.85618500
C	-12.59862200	3.40643000	-8.44642400
H	-13.84635000	4.66860200	-9.68346700
H	-11.62513000	2.07652700	-7.04481000
H	-11.67213400	3.68085100	-8.95750000
C	-17.80979100	3.33785200	-6.82096200
C	-18.50890000	3.17282400	-8.02974100
C	-18.11136500	4.44672100	-6.01156900
C	-19.47703600	4.10121100	-8.42301500
H	-18.30245500	2.31766500	-8.67722600
C	-19.07403900	5.37808400	-6.40902100
H	-17.60223200	4.57027600	-5.05316100
C	-19.75874300	5.20726100	-7.61530100
H	-20.01498000	3.95778000	-9.36391300
H	-19.30060000	6.22910300	-5.76219300

H	-20.51916500	5.93011800	-7.92209700
C	-18.87874700	-3.00008500	0.64068200
H	-18.93985000	-4.09011400	0.58744700
H	-19.82053700	-2.44295900	0.60872300
C	-17.68759700	-2.36452200	0.75417800
H	-16.77206300	-2.96854500	0.77540400
C	-17.51954500	-0.93546900	0.82249000
H	-18.41498100	-0.32516100	0.96395200
H	-16.62233100	-0.54994300	1.30989800
Cl	-16.99208800	-0.15837800	-1.19976000
C	-11.36460000	1.42660700	-2.90978100
H	-10.85946900	1.49272500	-1.93614600
H	-11.31636700	0.39545500	-3.27979500
H	-10.86893200	2.10002000	-3.62556000

UB3LYP-D3/def2-SVP-SMD(THF)//UB3LYP/def2-SVP(gas)
HF= -4325.5726358
UPBEPBE-D3/def2-SVP-SMD(THF)//UB3LYP/def2-SVP(gas)
HF= -4322.3283668

^{5}B-TS

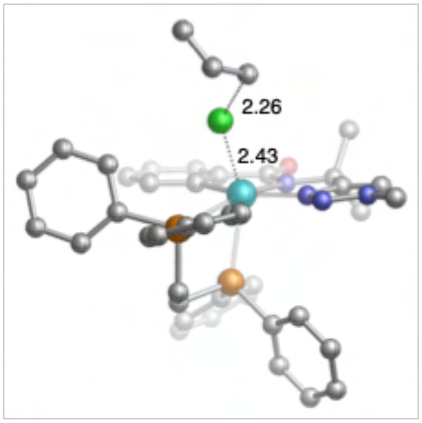

Zero-point correction=	0.749273 (Hartree/Particle)		
Thermal correction to Energy=	0.800565		
Thermal correction to Enthalpy=	0.801509		
Thermal correction to Gibbs Free Energy=	0.653538		
Sum of electronic and zero-point Energies=	-4324.621196		
Sum of electronic and thermal Energies=	-4324.569905		
Sum of electronic and thermal Enthalpies=	-4324.568960		
Sum of electronic and thermal Free Energies=	-4324.716932		
C	-19.07715500	1.64973700	-3.61640500
C	-19.44880200	2.96321200	-3.25698000
C	-20.77281800	3.40030000	-3.39237200
C	-21.74974800	2.53275900	-3.88566600
C	-21.39529700	1.22648200	-4.23963700
C	-20.06943700	0.78807800	-4.10155400
H	-21.00686200	4.42679700	-3.09815100
H	-22.78522400	2.86941600	-3.98932100
H	-22.15605000	0.53600400	-4.61798200
H	-19.82955800	-0.24522600	-4.36663700
C	-18.40761900	3.90843700	-2.70989400
O	-18.70114000	5.04624800	-2.32422000
C	-16.05149000	4.05221100	-2.05168600
C	-16.35676700	4.32793200	-0.55664200
H	-15.49274500	4.78984900	-0.05002300
H	-17.21888800	5.00332500	-0.48714000
H	-16.60491800	3.38488500	-0.04622100
C	-15.67672400	5.37295700	-2.76744100
H	-16.55385600	6.03303200	-2.76390100
H	-14.84146100	5.88325700	-2.25858900
H	-15.37418800	5.17714300	-3.80793000
C	-14.87624500	3.09555600	-2.12351600
N	-14.99748000	1.85598800	-2.67699800
N	-13.87040600	1.20185600	-2.59924000
C	-13.56875100	3.19784400	-1.68015700
H	-13.02481700	3.99377700	-1.17969200
N	-12.99561500	2.00696300	-1.99769200
N	-17.15982400	3.35802300	-2.70518200
Fe	-17.04793900	1.45017500	-3.37165300

P	-16.51008200	-1.32910800	-4.78514800
P	-16.68880500	1.93897400	-5.96105300
C	-16.14884000	-0.79417900	-6.54041200
H	-16.25619800	-1.63128800	-7.24944000
H	-15.08085300	-0.52223700	-6.55782000
C	-17.01801100	0.39605700	-6.97074200
H	-18.08771800	0.16302500	-6.83980100
H	-16.85374300	0.62091800	-8.03693100
C	-18.01090000	-2.40083300	-4.94529400
C	-18.64764200	-2.78174000	-3.74820100
C	-18.55986500	-2.83400200	-6.16430200
C	-19.78305100	-3.59425800	-3.77292200
H	-18.26575800	-2.41600400	-2.79214400
C	-19.70485300	-3.63806500	-6.18718900
H	-18.10147000	-2.55033200	-7.11382900
C	-20.31602300	-4.02512700	-4.99214300
H	-20.26255900	-3.87811800	-2.83267300
H	-20.11886300	-3.96217300	-7.14576000
H	-21.21092200	-4.65252100	-5.01090700
C	-15.16482800	-2.57655100	-4.50710400
C	-15.19708000	-3.87796300	-5.03792900
C	-14.06237100	-2.18930200	-3.72507900
C	-14.14433500	-4.76666600	-4.80489500
H	-16.05447600	-4.20433500	-5.63138200
C	-13.00829900	-3.08096700	-3.49625600
H	-14.03036700	-1.18585600	-3.29200000
C	-13.04603700	-4.37001400	-4.03454700
H	-14.18372300	-5.77574000	-5.22394000
H	-12.15651100	-2.76900700	-2.88530200
H	-12.22517300	-5.06792600	-3.84951300
C	-15.05952100	2.47864400	-6.67362800
C	-14.96072500	3.30175200	-7.80983900
C	-13.87429200	2.02679500	-6.06548200
C	-13.71156100	3.65331900	-8.32834100
H	-15.86654400	3.67495000	-8.29222200
C	-12.62536600	2.37089300	-6.59306200
H	-13.92370000	1.40798100	-5.16736100
C	-12.53992100	3.18652100	-7.72427200
H	-13.65439100	4.29617900	-9.21078200
H	-11.71470000	2.00390600	-6.11181200
H	-11.56393100	3.46184800	-8.13240400
C	-17.86516400	3.16777300	-6.66810700
C	-18.72567200	2.90060600	-7.74593500
C	-17.91203200	4.43842500	-6.06676200
C	-19.60157900	3.88454100	-8.21644600
H	-18.72804800	1.92382000	-8.23166300
C	-18.78295400	5.42031300	-6.54084500
H	-17.27467700	4.65671500	-5.20855500
C	-19.63083500	5.14627600	-7.61830700
H	-20.26718900	3.65795600	-9.05349100
H	-18.81132400	6.39546800	-6.04907000

H	-20.32067500	5.91132600	-7.98371500
C	-17.28559400	-3.19529900	0.70349100
H	-17.33677000	-4.27742700	0.55998500
H	-17.95694600	-2.75693700	1.44882400
C	-16.42471800	-2.42772100	-0.00969100
H	-15.78137600	-2.91049600	-0.75497500
C	-16.30738800	-1.00098900	0.11485500
H	-16.81345300	-0.51268300	0.95133800
H	-15.37907000	-0.52807200	-0.20803300
Cl	-17.54834500	-0.01865300	-1.49526500
C	-11.64066300	1.55589700	-1.73641800
H	-11.48311900	1.40280300	-0.65800800
H	-11.49636700	0.60571000	-2.26424800
H	-10.91745200	2.29566900	-2.10828100

UB3LYP-D3/def2-SVP-SMD(THF)//UB3LYP/def2-SVP(gas)
HF= -4325.5648004
UPBEPBE-D3/def2-SVP-SMD(THF)//UB3LYP/def2-SVP(gas)
HF= -4322.3048615

¹B'-TS

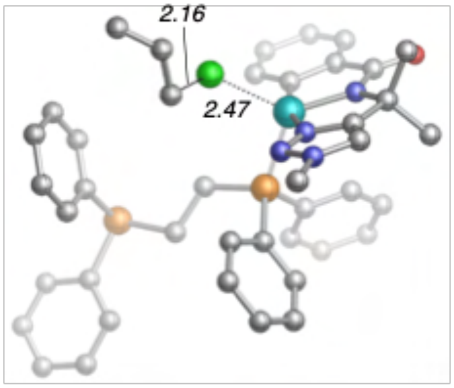

Zero-point correction= 0.751627 (Hartree/Particle)
Thermal correction to Energy= 0.801298
Thermal correction to Enthalpy= 0.802242
Thermal correction to Gibbs Free Energy= 0.661914
Sum of electronic and zero-point Energies= -4324.612532
Sum of electronic and thermal Energies= -4324.562862
Sum of electronic and thermal Enthalpies= -4324.561917
Sum of electronic and thermal Free Energies= -4324.702245

C	-18.47667600	1.19107300	-3.35043500
C	-19.18836500	2.26700600	-2.76753400
C	-20.58070400	2.22173600	-2.61819200
C	-21.29253600	1.09557100	-3.03786100
C	-20.60436400	0.01433900	-3.60016000
C	-19.21070200	0.06479200	-3.75467400
H	-21.07762100	3.08259300	-2.16301400
H	-22.37914400	1.05438200	-2.91967000
H	-21.15355300	-0.87799400	-3.91735300
H	-18.69653200	-0.80083400	-4.18385400
C	-18.43359700	3.47775300	-2.26780700
O	-19.00850700	4.42776400	-1.73015700
C	-16.16413900	4.36870000	-1.93052100
C	-16.31023400	4.52756200	-0.39637000
H	-15.57068300	5.24325400	-0.00001700
H	-17.32049000	4.88964500	-0.16837800
H	-16.16176100	3.55718700	0.10211300
C	-16.34363800	5.74021000	-2.62428400
H	-17.35942600	6.10443600	-2.42174800
H	-15.61310100	6.47829900	-2.25217000
H	-16.21947300	5.63779200	-3.71278200
C	-14.77716800	3.82475400	-2.22217900
N	-14.61409300	2.64132800	-2.88101900
N	-13.35021100	2.34020800	-2.99558100
C	-13.50543300	4.27579500	-1.91684700
H	-13.14418200	5.15877000	-1.39749700
N	-12.66828800	3.32437800	-2.41394500
N	-17.08562700	3.36595700	-2.46510400
Fe	-16.51645700	1.75079100	-3.46781100
P	-16.47159700	2.24240600	-6.00075000

C	-17.41140100	0.74826700	-8.33365800
H	-18.10585400	1.55310300	-8.62470600
H	-16.46986900	0.93947200	-8.87122100
C	-17.20857800	0.73239700	-6.81920200
H	-16.53740200	-0.08537600	-6.51028500
H	-18.15539800	0.54092100	-6.29024800
C	-14.92687600	2.51559600	-6.98355400
C	-14.89294700	3.13549800	-8.24653500
C	-13.72807300	2.01461400	-6.44591300
C	-13.69110000	3.25099700	-8.95059300
H	-15.80979900	3.53608300	-8.68369400
C	-12.52793900	2.12274900	-7.15657300
H	-13.73266000	1.54507000	-5.46002700
C	-12.50579900	2.74255900	-8.40891100
H	-13.68228500	3.73651700	-9.93002100
H	-11.60533200	1.72650900	-6.72376800
H	-11.56725400	2.83254200	-8.96224800
C	-17.59032900	3.63421800	-6.44827200
C	-18.98245000	3.43484900	-6.48282700
C	-17.09545700	4.94197900	-6.60598500
C	-19.85040200	4.50954100	-6.69447700
H	-19.40336500	2.44026700	-6.32319900
C	-17.96583700	6.01370100	-6.82022200
H	-16.02026800	5.13003900	-6.56125000
C	-19.34649200	5.80081800	-6.86888100
H	-20.92868000	4.33318400	-6.71224400
H	-17.56124000	7.02173200	-6.94355100
H	-20.02731000	6.64009000	-7.03128200
C	-11.21734500	3.29195500	-2.38517800
H	-10.79934700	4.09407700	-3.01188700
H	-10.85430200	3.40820000	-1.35381000
H	-10.89987000	2.31865400	-2.77759700
P	-18.21798400	-0.83658900	-8.95295800
C	-18.24765900	-0.46430200	-10.77431300
C	-19.35322800	0.26439700	-11.25179700
C	-17.26916400	-0.87502200	-11.69474600
C	-19.46429500	0.59707200	-12.60396800
H	-20.14133100	0.56826700	-10.55572600
C	-17.38737800	-0.55432800	-13.05121800
H	-16.40748600	-1.45249500	-11.35341300
C	-18.48075200	0.18594600	-13.50935000
H	-20.32830000	1.16846700	-12.95372300
H	-16.61766100	-0.88545800	-13.75380900
H	-18.57060900	0.43584300	-14.56973500
C	-16.84431100	-2.07704200	-8.80934100
C	-17.22125700	-3.43094700	-8.74689400
C	-15.47566700	-1.76348000	-8.72881800
C	-16.26386800	-4.44201500	-8.62817100
H	-18.28260900	-3.69293300	-8.78648400
C	-14.51575600	-2.77293600	-8.59904200
H	-15.14254600	-0.72402000	-8.77026700

C	-14.90633900	-4.11505400	-8.55173700
H	-16.57991500	-5.48771300	-8.58684100
H	-13.45642400	-2.50790800	-8.54107600
H	-14.15504700	-4.90330700	-8.45481600
C	-14.44160100	-4.13851800	-3.93394000
H	-14.57749700	-4.13491900	-5.02027700
H	-14.60227900	-5.08585000	-3.41320900
C	-14.09388800	-3.01813600	-3.26342000
H	-13.98670200	-3.05911400	-2.17343200
C	-13.87817400	-1.73281100	-3.89074300
H	-13.20779900	-1.02712000	-3.39596000
H	-13.82794800	-1.71631000	-4.98295900
Cl	-15.66778300	-0.55774800	-3.63340900

UB3LYP-D3/def2-SVP-SMD(THF)//UB3LYP/def2-SVP(gas)
HF= -4325.5409762
UPBEPBE-D3/def2-SVP-SMD(THF)//UB3LYP/def2-SVP(gas)
HF= -4322.2994625

^{3}B’-TS

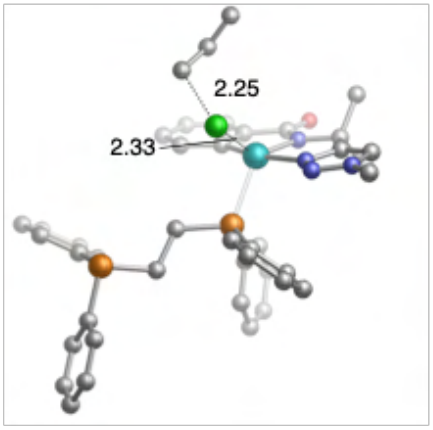

Zero-point correction= 0.750801 (Hartree/Particle)
Thermal correction to Energy= 0.801627
Thermal correction to Enthalpy= 0.802571
Thermal correction to Gibbs Free Energy= 0.655296
Sum of electronic and zero-point Energies= -4324.630671
Sum of electronic and thermal Energies= -4324.579846
Sum of electronic and thermal Enthalpies= -4324.578902
Sum of electronic and thermal Free Energies= -4324.726176

C	-20.08611600	-2.73054200	-4.16505800
C	-20.65929000	-2.24211700	-2.97068400
C	-21.97763900	-2.52665600	-2.60235600
C	-22.77369500	-3.31082300	-3.44273500
C	-22.23953300	-3.78933100	-4.64513200
C	-20.91246400	-3.50086900	-5.00091000
H	-22.35743000	-2.11420100	-1.66345600
H	-23.80699200	-3.54221200	-3.16933100
H	-22.85922800	-4.39395100	-5.31484900
H	-20.53519600	-3.88120400	-5.95511900
C	-19.78699600	-1.35553700	-2.13060000
O	-20.16098500	-0.85481700	-1.06745700
C	-17.56547100	-0.30003900	-2.10228400
C	-17.13255400	-0.81229500	-0.70732400
H	-16.34475000	-0.17192500	-0.27654100
H	-18.00310000	-0.81144300	-0.03897000
H	-16.74407500	-1.83969300	-0.78350000
C	-18.07482700	1.15773700	-2.00581400
H	-18.95306000	1.18901600	-1.34837400
H	-17.29645500	1.82493500	-1.59913300
H	-18.36462000	1.52305500	-3.00311700
C	-16.38217400	-0.35045600	-3.03996900
N	-16.42917500	-1.15907900	-4.13883700
N	-15.33033700	-1.06705600	-4.83458500
C	-15.15066700	0.27496300	-3.07077700
H	-14.67384300	0.99518800	-2.41226600
N	-14.54734500	-0.20670000	-4.19459600
N	-18.56554800	-1.18442500	-2.72245800
Fe	-18.22384500	-2.12180700	-4.39599600

C	-13.24641500	0.13215900	-4.74255900
H	-12.48575700	0.09922700	-3.94992800
H	-13.00837500	-0.61208600	-5.51174300
H	-13.26270000	1.13675700	-5.19231300
P	-17.17644400	-4.36270300	-4.69254500
C	-18.00738400	-5.39553700	-6.00932800
H	-18.03141900	-4.75152300	-6.90431400
H	-19.05252100	-5.46638600	-5.67231700
C	-17.44452700	-6.78006000	-6.33148400
H	-17.56736600	-7.47175500	-5.48417800
H	-16.36351000	-6.72642800	-6.53966700
P	-18.18398200	-7.50747400	-7.90325100
C	-19.91364100	-7.94351000	-7.40014100
C	-20.84842100	-8.10676400	-8.43926300
C	-20.35858100	-8.09106500	-6.07460100
C	-22.18092200	-8.42537400	-8.16580300
H	-20.52485100	-7.97707600	-9.47629600
C	-21.69570500	-8.39476400	-5.79791800
H	-19.66362200	-7.97108900	-5.24059600
C	-22.60907900	-8.56651300	-6.84206200
H	-22.88955500	-8.55265000	-8.98847900
H	-22.02336700	-8.49689700	-4.76008900
H	-23.65358700	-8.80394300	-6.62414400
C	-17.32405900	-9.15557000	-7.90134100
C	-17.86444400	-10.34186400	-7.37754400
C	-16.04860200	-9.20513800	-8.49470900
C	-17.14378400	-11.53939600	-7.43550700
H	-18.85737000	-10.33513400	-6.92284000
C	-15.32299300	-10.39798300	-8.54049500
H	-15.62166200	-8.29787700	-8.93373200
C	-15.87087200	-11.57119800	-8.01194300
H	-17.58186400	-12.45363200	-7.02571000
H	-14.33183600	-10.41401700	-9.00175600
H	-15.31007700	-12.50849000	-8.05575200
C	-17.20624100	-5.42717000	-3.18230700
C	-18.03372800	-5.03482600	-2.11650700
C	-16.46562900	-6.61934900	-3.06085900
C	-18.13058000	-5.82030300	-0.96275500
H	-18.60533900	-4.10813400	-2.18928500
C	-16.56164800	-7.40023500	-1.90670000
H	-15.79759400	-6.93548300	-3.86426700
C	-17.39724600	-7.00392300	-0.85626500
H	-18.78170600	-5.49816500	-0.14643300
H	-15.98005000	-8.32251200	-1.82729600
H	-17.47114500	-7.61684800	0.04593100
C	-15.40163900	-4.33602600	-5.20111800
C	-15.06686200	-4.13673800	-6.55251300
C	-14.36358100	-4.37550100	-4.25405100
C	-13.73323400	-4.01086200	-6.94819300
H	-15.85092800	-4.06151100	-7.30903300
C	-13.02933800	-4.24493900	-4.65016700

H	-14.59459100	-4.51253600	-3.19554300
C	-12.70770600	-4.06793200	-5.99916900
H	-13.49601000	-3.86537100	-8.00537700
H	-12.23665500	-4.28953900	-3.89818600
H	-11.66344300	-3.97752200	-6.30999800
C	-20.56159400	1.42519100	-6.56369100
H	-19.98432600	1.89426100	-7.36736400
H	-20.98673100	2.08491900	-5.80322900
C	-20.75037100	0.08553800	-6.51822300
H	-21.31918300	-0.34723400	-5.68837100
C	-20.21518000	-0.83623700	-7.48387300
H	-19.77705900	-0.43108000	-8.39983700
H	-20.69383300	-1.81136200	-7.58277000
Cl	-18.28482300	-1.65929100	-6.67592000

UB3LYP-D3/def2-SVP-SMD(THF)//UB3LYP/def2-SVP(gas)
HF= -4325.5650786
UPBEPBE-D3/def2-SVP-SMD(THF)//UB3LYP/def2-SVP(gas)
HF= -4322.3211285

[5]B'-TS

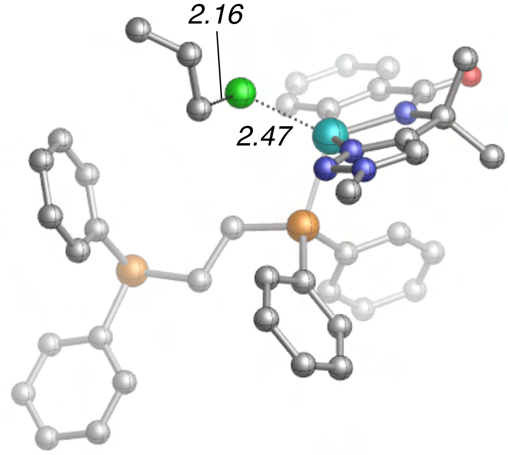

Zero-point correction= 0.749600 (Hartree/Particle)
Thermal correction to Energy= 0.801079
Thermal correction to Enthalpy= 0.802023
Thermal correction to Gibbs Free Energy= 0.651071
Sum of electronic and zero-point Energies= -4324.628103
Sum of electronic and thermal Energies= -4324.576625
Sum of electronic and thermal Enthalpies= -4324.575680
Sum of electronic and thermal Free Energies= -4324.726633

C	-18.47667600	1.19107300	-3.35043500
C	-19.18836500	2.26700600	-2.76753400
C	-20.58070400	2.22173600	-2.61819200
C	-21.29253600	1.09557100	-3.03786100
C	-20.60436400	0.01433900	-3.60016000
C	-19.21070200	0.06479200	-3.75467400
H	-21.07762100	3.08259300	-2.16301400
H	-22.37914400	1.05438200	-2.91967000
H	-21.15355300	-0.87799400	-3.91735300
H	-18.69653200	-0.80083400	-4.18385400
C	-18.43359700	3.47775300	-2.26780700
O	-19.00850700	4.42776400	-1.73015700
C	-16.16413900	4.36870000	-1.93052100
C	-16.31023400	4.52756200	-0.39637000
H	-15.57068300	5.24325400	-0.00001700
H	-17.32049000	4.88964500	-0.16837800
H	-16.16176100	3.55718700	0.10211300
C	-16.34363800	5.74021000	-2.62428400
H	-17.35942600	6.10443600	-2.42174800
H	-15.61310100	6.47829900	-2.25217000
H	-16.21947300	5.63779200	-3.71278200
C	-14.77716800	3.82475400	-2.22217900
N	-14.61409300	2.64132800	-2.88101900
N	-13.35021100	2.34020800	-2.99558100
C	-13.50543300	4.27579500	-1.91684700
H	-13.14418200	5.15877000	-1.39749700
N	-12.66828800	3.32437800	-2.41394500
N	-17.08562700	3.36595700	-2.46510400

Fe	-16.51645700	1.75079100	-3.46781100
P	-16.47159700	2.24240600	-6.00075000
C	-17.41140100	0.74826700	-8.33365800
H	-18.10585400	1.55310300	-8.62470600
H	-16.46986900	0.93947200	-8.87122100
C	-17.20857800	0.73239700	-6.81920200
H	-16.53740200	-0.08537600	-6.51028500
H	-18.15539800	0.54092100	-6.29024800
C	-14.92687600	2.51559600	-6.98355400
C	-14.89294700	3.13549800	-8.24653500
C	-13.72807300	2.01461400	-6.44591300
C	-13.69110000	3.25099700	-8.95059300
H	-15.80979900	3.53608300	-8.68369400
C	-12.52793900	2.12274900	-7.15657300
H	-13.73266000	1.54507000	-5.46002700
C	-12.50579900	2.74255900	-8.40891100
H	-13.68228500	3.73651700	-9.93002100
H	-11.60533200	1.72650900	-6.72376800
H	-11.56725400	2.83254200	-8.96224800
C	-17.59032900	3.63421800	-6.44827200
C	-18.98245000	3.43484900	-6.48282700
C	-17.09545700	4.94197900	-6.60598500
C	-19.85040200	4.50954100	-6.69447700
H	-19.40336500	2.44026700	-6.32319900
C	-17.96583700	6.01370100	-6.82022200
H	-16.02026800	5.13003900	-6.56125000
C	-19.34649200	5.80081800	-6.86888100
H	-20.92868000	4.33318400	-6.71224400
H	-17.56124000	7.02173200	-6.94355100
H	-20.02731000	6.64009000	-7.03128200
C	-11.21734500	3.29195500	-2.38517800
H	-10.79934700	4.09407700	-3.01188700
H	-10.85430200	3.40820000	-1.35381000
H	-10.89987000	2.31865400	-2.77759700
P	-18.21798400	-0.83658900	-8.95295800
C	-18.24765900	-0.46430200	-10.77431300
C	-19.35322800	0.26439700	-11.25179700
C	-17.26916400	-0.87502200	-11.69474600
C	-19.46429500	0.59707200	-12.60396800
H	-20.14133100	0.56826700	-10.55572600
C	-17.38737800	-0.55432800	-13.05121800
H	-16.40748600	-1.45249500	-11.35341300
C	-18.48075200	0.18594600	-13.50935000
H	-20.32830000	1.16846700	-12.95372300
H	-16.61766100	-0.88545800	-13.75380900
H	-18.57060900	0.43584300	-14.56973500
C	-16.84431100	-2.07704200	-8.80934100
C	-17.22125700	-3.43094700	-8.74689400
C	-15.47566700	-1.76348000	-8.72881800
C	-16.26386800	-4.44201500	-8.62817100
H	-18.28260900	-3.69293300	-8.78648400

C	-14.51575600	-2.77293600	-8.59904200
H	-15.14254600	-0.72402000	-8.77026700
C	-14.90633900	-4.11505400	-8.55173700
H	-16.57991500	-5.48771300	-8.58684100
H	-13.45642400	-2.50790800	-8.54107600
H	-14.15504700	-4.90330700	-8.45481600
C	-14.44160100	-4.13851800	-3.93394000
H	-14.57749700	-4.13491900	-5.02027700
H	-14.60227900	-5.08585000	-3.41320900
C	-14.09388800	-3.01813600	-3.26342000
H	-13.98670200	-3.05911400	-2.17343200
C	-13.87817400	-1.73281100	-3.89074300
H	-13.20779900	-1.02712000	-3.39596000
H	-13.82794800	-1.71631000	-4.98295900
Cl	-15.66778300	-0.55774800	-3.63340900

UB3LYP-D3/def2-SVP-SMD(THF)//UB3LYP/def2-SVP(gas)
HF= -4325.5590406
UPBEPBE-D3/def2-SVP-SMD(THF)//UB3LYP/def2-SVP(gas)
HF= -4322.2972773

[1]B-DCIB-TS

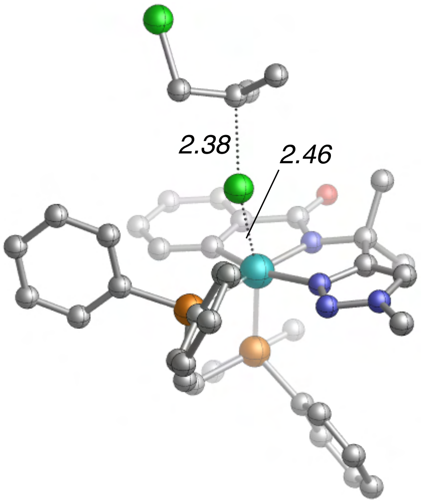

Zero-point correction= 0.794616 (Hartree/Particle)
Thermal correction to Energy= 0.847431
Thermal correction to Enthalpy= 0.848375
Thermal correction to Gibbs Free Energy= 0.703809
Sum of electronic and zero-point Energies= -4824.560788
Sum of electronic and thermal Energies= -4824.507973
Sum of electronic and thermal Enthalpies= -4824.507028
Sum of electronic and thermal Free Energies= -4824.651594

C	-18.53710000	1.23459100	-3.72660000
C	-18.93951700	2.39771800	-3.02667700
C	-20.28117100	2.73212500	-2.81226900
C	-21.28921200	1.90088400	-3.30549900
C	-20.92904300	0.74392600	-4.00405300
C	-19.57818200	0.42041800	-4.20701800
H	-20.50552300	3.64543300	-2.25453800
H	-22.34280900	2.14823400	-3.14760700
H	-21.70609400	0.07820100	-4.39396600
H	-19.35997300	-0.50516300	-4.74289800
C	-17.85359300	3.26827500	-2.48606000
O	-18.06832000	4.29178200	-1.82354400
C	-15.41644900	3.39245000	-2.23333700
C	-15.45444900	3.41087400	-0.68409800
H	-14.51410200	3.81609900	-0.27476400
H	-16.29287700	4.03409200	-0.34935400
H	-15.59101700	2.38776000	-0.30539700
C	-15.18270600	4.82389000	-2.77213800
H	-16.04778900	5.44651600	-2.50904300
H	-14.27529400	5.27058400	-2.33204600
H	-15.05865200	4.81202800	-3.86589300
C	-14.28444100	2.49707900	-2.66770900
N	-14.55196300	1.36311100	-3.37535800
N	-13.45006400	0.71513400	-3.63836400
C	-12.91532400	2.53417300	-2.48383500
H	-12.26072200	3.23778900	-1.97773500
N	-12.45325200	1.41201000	-3.09945800

N	-16.62211400	2.77440200	-2.80938500
Fe	-16.54452600	1.07656900	-3.72741400
P	-16.30642100	-1.07491900	-4.87032900
P	-16.66895400	1.96763300	-5.89612300
C	-16.17204500	-0.65880900	-6.68897900
H	-16.40327200	-1.52233400	-7.32974400
H	-15.11538100	-0.40946500	-6.87074400
C	-17.06665000	0.53628100	-7.03079700
H	-18.12574400	0.28867400	-6.85955600
H	-16.94861800	0.83565300	-8.08394800
C	-17.64459000	-2.36690800	-4.81906500
C	-17.88651400	-3.02026600	-3.59734300
C	-18.42631300	-2.71857900	-5.93152200
C	-18.87427200	-4.00150300	-3.49645500
H	-17.30436400	-2.74721200	-2.71627600
C	-19.41919200	-3.69902100	-5.82765400
H	-18.28313600	-2.22870400	-6.89603900
C	-19.64584000	-4.34507300	-4.61105400
H	-19.04561900	-4.49801800	-2.53776900
H	-20.01755800	-3.95494100	-6.70602000
H	-20.42169800	-5.11056100	-4.53002800
C	-14.83175100	-2.17375500	-4.59608900
C	-14.27881100	-2.93380200	-5.64153100
C	-14.27908600	-2.29446800	-3.31082500
C	-13.19607300	-3.78699400	-5.41016500
H	-14.69347800	-2.87873100	-6.64998900
C	-13.20531800	-3.15906500	-3.07828600
H	-14.69551900	-1.70838700	-2.48987300
C	-12.65640000	-3.90381300	-4.12607100
H	-12.77994100	-4.36917100	-6.23669800
H	-12.79399400	-3.24781500	-2.06897400
H	-11.81531000	-4.57781200	-3.94275600
C	-15.18298700	2.69423100	-6.76166800
C	-15.27629400	3.81242600	-7.61046200
C	-13.92516700	2.08126400	-6.60639200
C	-14.15113300	4.29740600	-8.28410400
H	-16.23516000	4.31258300	-7.75161600
C	-12.80396600	2.56044700	-7.29092600
H	-13.80751300	1.22907300	-5.93644900
C	-12.91121300	3.67205500	-8.13064500
H	-14.24907600	5.16949400	-8.93600800
H	-11.83937500	2.06177300	-7.16145300
H	-12.03341500	4.05057900	-8.66080200
C	-17.97027900	3.21893800	-6.26674800
C	-18.97662300	3.00731200	-7.22290600
C	-17.95219800	4.43868000	-5.56733900
C	-19.93712600	3.99188600	-7.47557900
H	-19.02983600	2.07255700	-7.78177000
C	-18.90940100	5.42152000	-5.82343600
H	-17.19102400	4.62184300	-4.80931500
C	-19.90577300	5.20167900	-6.77906900

H	-20.71574100	3.80592900	-8.21986100
H	-18.88224600	6.35674700	-5.25926100
H	-20.66014800	5.96813000	-6.97429300
Cl	-16.68816800	-0.26295400	-1.67465700
C	-11.08417600	0.95164600	-3.24312500
H	-10.57161600	0.98695100	-2.27134400
H	-11.11758400	-0.08396900	-3.60236900
H	-10.53657600	1.57545400	-3.96623500
C	-17.69209300	-0.32387000	0.48422700
C	-16.49348100	-0.32455300	1.38533900
H	-15.93876400	0.62222900	1.31912700
H	-16.82883900	-0.44653700	2.43409800
H	-15.80919900	-1.15549700	1.15705200
C	-18.56137900	0.89546700	0.48052700
H	-19.24797200	0.90644700	-0.37816700
H	-19.17453700	0.89762500	1.40308800
H	-17.97302600	1.82304400	0.46447100
C	-18.37482200	-1.63243800	0.27292500
H	-19.11493600	-1.59065600	-0.53394800
H	-17.66918200	-2.45340500	0.10127500
Cl	-19.32237700	-2.12409000	1.79569000

UB3LYP-D3/def2-SVP-SMD(THF)//UB3LYP/def2-SVP(gas)
HF= -4825.5680988
UPBEPBE-D3/def2-SVP-SMD(THF)//UB3LYP/def2-SVP(gas)
HF= -4822.0818106

^{3}B-DCIB-TS

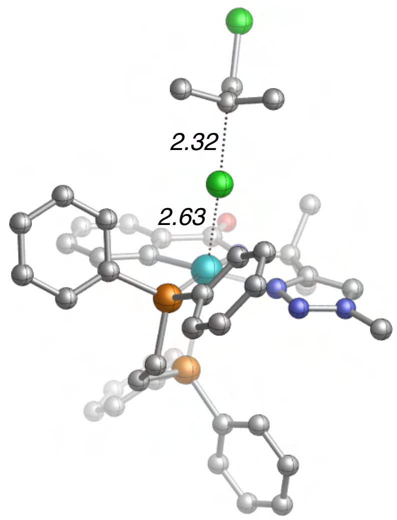

Zero-point correction= 0.792877 (Hartree/Particle)
Thermal correction to Energy= 0.846779
Thermal correction to Enthalpy= 0.847723
Thermal correction to Gibbs Free Energy= 0.695248
Sum of electronic and zero-point Energies= -4824.573327
Sum of electronic and thermal Energies= -4824.519426
Sum of electronic and thermal Enthalpies= -4824.518482
Sum of electronic and thermal Free Energies= -4824.670957

C	-18.62310700	1.60070900	-3.57164600
C	-18.90631300	2.78281400	-2.85328800
C	-20.20186000	3.29149200	-2.72467100
C	-21.26679900	2.62378100	-3.33359300
C	-21.01660500	1.45545100	-4.06049800
C	-19.71232000	0.94969300	-4.17298800
H	-20.34553700	4.20875400	-2.14730600
H	-22.28644300	3.00915500	-3.24442000
H	-21.84379500	0.92404400	-4.54148700
H	-19.57576400	0.02211100	-4.73161800
C	-17.74394400	3.49615900	-2.23816100
O	-17.85942500	4.52808400	-1.57166800
C	-15.30198600	3.37198300	-2.00097500
C	-15.28011200	3.33593500	-0.45299700
H	-14.30243300	3.66502500	-0.06299100
H	-16.06257900	4.00318800	-0.06933400
H	-15.47808400	2.31227800	-0.10389300
C	-14.99435700	4.80175500	-2.50934600
H	-15.78961800	5.47817400	-2.17199100
H	-14.02781300	5.16105500	-2.11807300
H	-14.94922400	4.81828100	-3.60936600
C	-14.24888500	2.43092000	-2.53189600
N	-14.61412000	1.34762700	-3.27284700
N	-13.56667000	0.67036700	-3.65945800
C	-12.86897800	2.39991200	-2.45345400
H	-12.14516900	3.04836700	-1.96849000

N	-12.50664800	1.29531000	-3.16302300
N	-16.57281700	2.85926800	-2.53918600
Fe	-16.66943600	1.17377300	-3.46785700
P	-16.65524100	-0.94957100	-4.79171200
P	-16.52706000	2.24527000	-6.21321000
C	-16.41307500	-0.54298200	-6.60092700
H	-16.68198800	-1.40897900	-7.22370200
H	-15.32778500	-0.40703900	-6.72955800
C	-17.16738500	0.70704100	-7.07465800
H	-18.24012100	0.63783600	-6.83304400
H	-17.07622700	0.80244700	-8.16854800
C	-18.14372700	-2.05458400	-4.75148500
C	-18.61195600	-2.44992200	-3.48510900
C	-18.80991400	-2.52217000	-5.89537700
C	-19.70806200	-3.30658500	-3.37144500
H	-18.12489700	-2.06601000	-2.58577700
C	-19.91417600	-3.37346900	-5.77698200
H	-18.48542500	-2.22798100	-6.89506900
C	-20.36335500	-3.77177700	-4.51621800
H	-20.05881100	-3.60315600	-2.37965400
H	-20.42344900	-3.72487900	-6.67820000
H	-21.22573000	-4.43713200	-4.42526100
C	-15.30957000	-2.20017600	-4.50419700
C	-14.89358500	-3.07415300	-5.52454300
C	-14.71852200	-2.31385700	-3.23583800
C	-13.90280400	-4.03007000	-5.28629700
H	-15.34458500	-3.02719300	-6.51769100
C	-13.73411100	-3.27847600	-2.99755300
H	-15.03794900	-1.65191100	-2.42915400
C	-13.31923200	-4.13530800	-4.02014900
H	-13.59251800	-4.70007500	-6.09249900
H	-13.28940000	-3.35790700	-2.00175200
H	-12.54893500	-4.88782300	-3.83125800
C	-15.04893900	2.66093100	-7.26251700
C	-15.06716400	3.61476600	-8.29567700
C	-13.83565100	2.00143000	-6.98296900
C	-13.91425000	3.88476900	-9.03928800
H	-15.98854100	4.15438900	-8.52314800
C	-12.68853700	2.26098800	-7.73900100
H	-13.78036500	1.28714100	-6.15733200
C	-12.72305200	3.20507100	-8.76926000
H	-13.94969700	4.63149900	-9.83712600
H	-11.75967200	1.72912600	-7.51457100
H	-11.82414800	3.41556500	-9.35450500
C	-17.75322500	3.53937600	-6.67574700
C	-18.58006200	3.47087900	-7.81116700
C	-17.86950300	4.65052700	-5.82263500
C	-19.49064300	4.49390100	-8.09074300
H	-18.51976500	2.61817200	-8.49105800
C	-18.77530800	5.67579600	-6.10628100
H	-17.25921200	4.70540900	-4.91873300

C	-19.58784500	5.59973300	-7.24066000
H	-20.12832200	4.42453200	-8.97605900
H	-18.85626300	6.52720800	-5.42620100
H	-20.30340100	6.39692800	-7.45817800
Cl	-16.76208300	-0.20857800	-1.23361000
C	-11.17190900	0.78939600	-3.43280900
H	-10.59712900	0.73215700	-2.49778700
H	-11.27804500	-0.21460700	-3.86082900
H	-10.64671600	1.44306000	-4.14555100
C	-17.11947600	-1.07959200	0.88505200
C	-18.12987300	-2.16359800	0.63670700
H	-17.71628500	-2.96075500	0.00075900
H	-18.42889400	-2.61877400	1.60091100
H	-19.03914900	-1.76671600	0.16043000
C	-15.74454200	-1.52643500	1.29503900
H	-15.02209200	-0.69673800	1.26464100
H	-15.77237100	-1.90813100	2.33428800
H	-15.37661300	-2.33671100	0.64765600
C	-17.63223400	0.16469100	1.52569900
H	-16.88977300	0.97034000	1.54147500
H	-18.56136800	0.52287400	1.06820100
Cl	-18.05000900	-0.13835900	3.31708400

UB3LYP-D3/def2-SVP-SMD(THF)//UB3LYP/def2-SVP(gas)
HF= -4825.5756637
UPBEPBE-D3/def2-SVP-SMD(THF)//UB3LYP/def2-SVP(gas)
HF= -4822.0664294

^{5}B-DCIB-TS

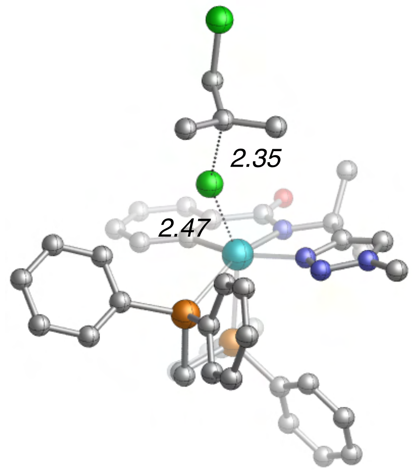

Zero-point correction= 0.791425 (Hartree/Particle)
Thermal correction to Energy= 0.845765
Thermal correction to Enthalpy= 0.846709
Thermal correction to Gibbs Free Energy= 0.692686
Sum of electronic and zero-point Energies= -4824.567379
Sum of electronic and thermal Energies= -4824.513038
Sum of electronic and thermal Enthalpies= -4824.512094
Sum of electronic and thermal Free Energies= -4824.666117

C	-19.14069700	1.76326100	-3.59858100
C	-19.44410000	3.07952500	-3.18984100
C	-20.75151700	3.57504700	-3.27055300
C	-21.77908600	2.76579000	-3.76050300
C	-21.49045200	1.46027400	-4.17126200
C	-20.18070700	0.96300300	-4.08783100
H	-20.93200300	4.60110000	-2.93923800
H	-22.80184900	3.14830900	-3.82112300
H	-22.28968700	0.81699700	-4.55332500
H	-19.99397000	-0.06803600	-4.40277600
C	-18.34297800	3.96456200	-2.65857800
O	-18.56435600	5.11525500	-2.26475800
C	-15.95705800	3.97111400	-2.07498500
C	-16.19357500	4.30908800	-0.58146400
H	-15.28175900	4.71950100	-0.11632800
H	-17.00209200	5.04687300	-0.50443600
H	-16.49151600	3.40202000	-0.03359700
C	-15.51795600	5.24253800	-2.84113700
H	-16.34770000	5.96168900	-2.82838700
H	-14.63251200	5.70839500	-2.37670900
H	-15.26729800	4.99553100	-3.88445800
C	-14.85859400	2.92766000	-2.16272300
N	-15.08685100	1.71236700	-2.73790900
N	-14.01845200	0.96598700	-2.69293500
C	-13.54222700	2.91222300	-1.73620400
H	-12.92774600	3.64832500	-1.22583400
N	-13.07480500	1.68339500	-2.08720500

N	-17.13039800	3.34258500	-2.68220300
Fe	-17.13568000	1.44551700	-3.37719700
P	-16.46266400	-1.27770400	-4.79238200
P	-16.83853900	1.95662100	-5.94564800
C	-16.30914900	-0.76049600	-6.58477700
H	-16.51069100	-1.60368000	-7.26427500
H	-15.26158400	-0.46304400	-6.74964700
C	-17.24350200	0.41057100	-6.92044300
H	-18.29033400	0.15714700	-6.68299900
H	-17.19043200	0.63475100	-7.99785600
C	-17.76807600	-2.59928100	-4.85628000
C	-18.79083800	-2.57313700	-3.89373100
C	-17.75659800	-3.63677300	-5.80611900
C	-19.78781400	-3.55673600	-3.89202900
H	-18.80071300	-1.78506700	-3.13715400
C	-18.75227400	-4.61527900	-5.80385700
H	-16.95769700	-3.69525200	-6.55025100
C	-19.77299900	-4.57549800	-4.84651700
H	-20.57967200	-3.52038900	-3.13937700
H	-18.72966300	-5.41531500	-6.54868200
H	-20.55267600	-5.34176700	-4.84542400
C	-14.93658700	-2.28814400	-4.51079700
C	-14.09706800	-2.77951900	-5.52441200
C	-14.60613200	-2.56324200	-3.17143900
C	-12.96284400	-3.53488500	-5.20652900
H	-14.31877600	-2.58147200	-6.57541700
C	-13.48210500	-3.32904500	-2.85484500
H	-15.23720900	-2.15967500	-2.37542100
C	-12.65469000	-3.81592300	-3.87288400
H	-12.32098800	-3.91024300	-6.00815100
H	-13.24683000	-3.54152100	-1.80833700
H	-11.77186000	-4.41191400	-3.62702500
C	-15.21379600	2.44304500	-6.70245000
C	-15.11921600	3.26082200	-7.84279300
C	-14.02788000	1.95729800	-6.12165500
C	-13.87317500	3.57503300	-8.39201000
H	-16.02535600	3.65980300	-8.30332200
C	-12.78285900	2.26400200	-6.68041400
H	-14.07403200	1.33829200	-5.22332500
C	-12.70142600	3.07517300	-7.81522400
H	-13.81860200	4.21458300	-9.27700200
H	-11.87205500	1.87125000	-6.22036600
H	-11.72801700	3.32172800	-8.24720200
C	-17.99764800	3.21580500	-6.62443700
C	-18.92811200	2.95464600	-7.64382700
C	-17.95964200	4.50323000	-6.05871000
C	-19.78885800	3.96115200	-8.09437700
H	-18.99874400	1.96499800	-8.09762000
C	-18.81516100	5.50727100	-6.51377300
H	-17.26546600	4.71984900	-5.24494900
C	-19.73296500	5.23913700	-7.53407000

H	-20.50953100	3.73932900	-8.88580900
H	-18.77578900	6.49668500	-6.05213700
H	-20.41038900	6.02228200	-7.88395700
Cl	-17.72085700	0.02216600	-1.45138300
C	-11.75235000	1.12022900	-1.88038900
H	-11.76699800	0.09484600	-2.26886000
H	-10.99460700	1.70874800	-2.41868800
H	-11.50640900	1.10437100	-0.80845300
C	-17.17410100	-0.95132800	0.61425300
C	-17.28401600	-2.41973600	0.32697500
H	-18.27433500	-2.68520900	-0.07162900
H	-16.51752800	-2.75499900	-0.38677000
H	-17.14075400	-2.98622800	1.26771900
C	-15.80007000	-0.40837300	0.87702100
H	-15.08552600	-0.73404100	0.10658300
H	-15.79899700	0.69105200	0.91642800
H	-15.44089200	-0.77853900	1.85695700
C	-18.29749000	-0.33329800	1.37585800
H	-18.24335400	0.76098400	1.39228200
H	-19.28004000	-0.65234700	1.01013500
Cl	-18.23416200	-0.86163500	3.15700000

UB3LYP-D3/def2-SVP-SMD(THF)//UB3LYP/def2-SVP(gas)
HF= -4825.5669119
UPBEPBE-D3/def2-SVP-SMD(THF)//UB3LYP/def2-SVP(gas)
HF= -4822.0439618

^{1}B-DCIB'-TS

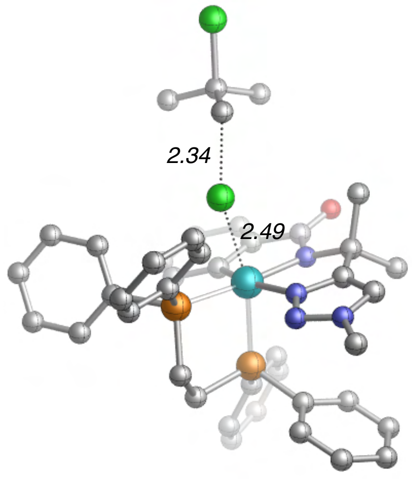

Zero-point correction= 0.793450 (Hartree/Particle)
Thermal correction to Energy= 0.846336
Thermal correction to Enthalpy= 0.847280
Thermal correction to Gibbs Free Energy= 0.701520
Sum of electronic and zero-point Energies= -4824.555757
Sum of electronic and thermal Energies= -4824.502871
Sum of electronic and thermal Enthalpies= -4824.501927
Sum of electronic and thermal Free Energies= -4824.647687

C	-18.73326000	1.33444500	-3.75083300
C	-19.17296100	2.44321000	-2.99129500
C	-20.52345000	2.78795000	-2.86523200
C	-21.49202300	2.03347200	-3.53068800
C	-21.08719100	0.94499100	-4.31209500
C	-19.73092300	0.60217000	-4.41722200
H	-20.78569800	3.65093200	-2.24678600
H	-22.55190100	2.28985900	-3.44682800
H	-21.83545500	0.34955600	-4.84513600
H	-19.47344100	-0.26430800	-5.03087300
C	-18.11136300	3.25856200	-2.33035700
O	-18.35251100	4.19122200	-1.55479200
C	-15.67585900	3.39603400	-2.04970600
C	-15.69563800	3.18320500	-0.51297000
H	-14.76764000	3.56554000	-0.05657300
H	-16.55431100	3.71864900	-0.08848700
H	-15.79307800	2.11333300	-0.28286600
C	-15.50329500	4.89968300	-2.36142300
H	-16.37321200	5.44201200	-1.97020800
H	-14.59030700	5.29911300	-1.88832000
H	-15.43284900	5.06743900	-3.44567500
C	-14.52131700	2.61181800	-2.62222400
N	-14.77145400	1.46296100	-3.31343400
N	-13.65969500	0.91904300	-3.73080500
C	-13.14836400	2.76771900	-2.61110200
H	-12.49835700	3.52666600	-2.18589100
N	-12.67137600	1.69676400	-3.30406600

N	-16.86850200	2.82639100	-2.70405800
Fe	-16.76595300	1.12840300	-3.62083800
P	-16.42068600	-1.08854700	-4.47909800
P	-16.59914200	1.82267600	-5.89466200
C	-15.72755200	-0.83647100	-6.19427800
H	-15.71586600	-1.76772300	-6.78101400
H	-14.67727100	-0.55879100	-6.01679100
C	-16.47949600	0.27587900	-6.93492500
H	-17.52035100	-0.02700300	-7.13108000
H	-16.01370700	0.49392900	-7.90880200
C	-17.83549700	-2.25953000	-4.72721000
C	-18.53984100	-2.68816400	-3.58684400
C	-18.23761800	-2.73651300	-5.98550200
C	-19.60865200	-3.57732900	-3.70619200
H	-18.25468300	-2.30592400	-2.60490400
C	-19.31456000	-3.62289400	-6.10263200
H	-17.72071300	-2.42556500	-6.89515800
C	-20.00149900	-4.04740700	-4.96385900
H	-20.14438600	-3.89849900	-2.80931600
H	-19.61392800	-3.98104900	-7.09115800
H	-20.84261000	-4.73937000	-5.05528500
C	-15.15986400	-2.23998400	-3.74815400
C	-14.82533300	-3.42763200	-4.42393100
C	-14.52602100	-1.95397500	-2.53001200
C	-13.86595200	-4.29918600	-3.90406000
H	-15.32800100	-3.68984800	-5.35826400
C	-13.57162100	-2.83275600	-2.00622700
H	-14.80362100	-1.05697700	-1.97830400
C	-13.23444200	-4.00209200	-2.69189900
H	-13.61916100	-5.21805900	-4.44243900
H	-13.09429900	-2.60174700	-1.05007200
H	-12.48961900	-4.68752300	-2.27875200
C	-15.07359500	2.77892700	-6.36571100
C	-15.05260300	4.17062900	-6.16234400
C	-13.92514900	2.16895800	-6.89844500
C	-13.92419700	4.92784000	-6.48514900
H	-15.93474600	4.67620900	-5.76512000
C	-12.79522500	2.92760000	-7.22455700
H	-13.89621600	1.09323400	-7.07505300
C	-12.78899800	4.30946700	-7.01806400
H	-13.93738800	6.00893800	-6.32497200
H	-11.92004500	2.43207500	-7.65391800
H	-11.90906100	4.90278900	-7.27961600
C	-17.89664400	2.82668300	-6.73868300
C	-18.19914500	2.65129900	-8.10042100
C	-18.56905000	3.82688500	-6.01804600
C	-19.15871200	3.45363800	-8.72299100
H	-17.68702700	1.89107900	-8.69339400
C	-19.52289400	4.63303700	-6.64538800
H	-18.36221000	3.96505800	-4.95744000
C	-19.82263400	4.44684200	-7.99701800

H	-19.38625900	3.30146700	-9.78129800
H	-20.04214900	5.39953800	-6.06529600
H	-20.57522400	5.07193200	-8.48447500
Cl	-17.21638000	-0.09687800	-1.49675300
C	-11.29876400	1.38922700	-3.66292300
H	-11.23706600	0.31582900	-3.87947100
H	-10.99595200	1.96213100	-4.55287200
H	-10.63380900	1.63401900	-2.82366700
C	-17.50255200	-0.55125700	0.78580700
C	-18.86053500	-0.10380700	1.18188600
Cl	-18.96244800	-0.68113200	3.01832500
H	-16.65634700	0.02433500	1.16734700
H	-17.33706600	-1.62643700	0.69059300
C	-19.03665800	1.40742200	1.20689300
H	-18.99446400	1.80338600	0.18047200
H	-20.00793000	1.67662500	1.64594900
H	-18.24359400	1.88874700	1.79720300
C	-19.98804500	-0.82418600	0.45542300
H	-20.96167700	-0.56006900	0.89241800
H	-19.97860700	-0.52286800	-0.60379000
H	-19.86353700	-1.91567900	0.51466800

UB3LYP-D3/def2-SVP-SMD(THF)//UB3LYP/def2-SVP(gas)
HF= -4825.5558606
UPBEPBE-D3/def2-SVP-SMD(THF)//UB3LYP/def2-SVP(gas)
HF= -4822.0721820

[3]B-DCIB'-TS

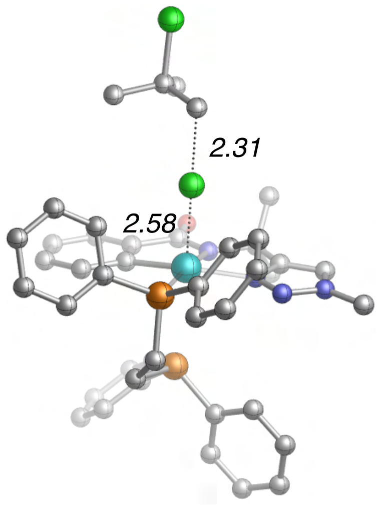

Zero-point correction=		0.793100 (Hartree/Particle)	
Thermal correction to Energy=		0.846885	
Thermal correction to Enthalpy=		0.847830	
Thermal correction to Gibbs Free Energy=		0.696789	
Sum of electronic and zero-point Energies=		-4824.573320	
Sum of electronic and thermal Energies=		-4824.519535	
Sum of electronic and thermal Enthalpies=		-4824.518590	
Sum of electronic and thermal Free Energies=		-4824.669631	
C	-18.84319800	1.43293500	-3.83808800
C	-19.32498600	2.59462400	-3.19732200
C	-20.66837900	2.97698600	-3.25010900
C	-21.57750300	2.19800400	-3.96959300
C	-21.12661000	1.04737700	-4.62415700
C	-19.77708800	0.66824900	-4.55449000
H	-20.97231100	3.88542200	-2.72309700
H	-22.63180000	2.48384000	-4.02209600
H	-21.83063000	0.42997700	-5.19059100
H	-19.48248900	-0.25044200	-5.06415000
C	-18.32334600	3.42576600	-2.45890000
O	-18.62086800	4.44306800	-1.82909500
C	-15.92854500	3.54405800	-1.91615200
C	-16.09135700	3.50061100	-0.37675800
H	-15.20782300	3.92922800	0.12508800
H	-16.97918900	4.08080600	-0.09422000
H	-16.22227700	2.46043800	-0.04580600
C	-15.70699000	4.99940700	-2.39468000
H	-16.59999400	5.59208100	-2.16066000
H	-14.83505000	5.45068400	-1.89244800
H	-15.53190200	5.02542600	-3.48144100
C	-14.72964500	2.71612100	-2.30723500
N	-14.89475300	1.59227100	-3.05868700
N	-13.74628500	1.02437700	-3.31150600
C	-13.37155100	2.83181300	-2.07622900
H	-12.77756700	3.55834900	-1.52952400
N	-12.81958100	1.76377700	-2.71635500

N	-17.06605800	2.90740700	-2.60214500
Fe	-16.88709600	1.20086200	-3.47155400
P	-16.51145100	-0.94912100	-4.70962200
P	-16.51298500	2.20229600	-6.24957600
C	-16.09150700	-0.57147200	-6.49243700
H	-16.19540500	-1.47972800	-7.10484400
H	-15.01727400	-0.32754000	-6.49667600
C	-16.90418900	0.57863500	-7.10321100
H	-17.98566600	0.41022700	-6.97816300
H	-16.69842600	0.64523100	-8.18356200
C	-17.88615800	-2.18929400	-4.80188400
C	-18.43762300	-2.63135000	-3.58523800
C	-18.39453000	-2.70787900	-6.00345300
C	-19.45906300	-3.58268200	-3.57596000
H	-18.07683800	-2.21090200	-2.64354300
C	-19.42397100	-3.65567000	-5.98996400
H	-18.00350300	-2.37840600	-6.96762700
C	-19.95584700	-4.09879500	-4.77730400
H	-19.87674200	-3.91398500	-2.62187500
H	-19.80983100	-4.04649800	-6.93515500
H	-20.75979100	-4.83918100	-4.76785500
C	-15.09973100	-2.05253200	-4.21683800
C	-14.51023800	-2.93263700	-5.14216000
C	-14.63262400	-2.05144200	-2.89311700
C	-13.46936800	-3.78085700	-4.75674100
H	-14.86406600	-2.97518800	-6.17419300
C	-13.59756000	-2.90972700	-2.50756900
H	-15.09156700	-1.38767300	-2.15868700
C	-13.00926300	-3.77160400	-3.43633000
H	-13.02301000	-4.45759400	-5.49016400
H	-13.25221900	-2.90313000	-1.47020000
H	-12.20010100	-4.44104900	-3.13244300
C	-14.97544700	2.72077600	-7.15892200
C	-14.97877800	3.59122600	-8.26326800
C	-13.73872400	2.22755100	-6.69938900
C	-13.78463600	3.94095100	-8.90074100
H	-15.92110100	4.00279500	-8.63011300
C	-12.54739700	2.56563200	-7.34886700
H	-13.70240700	1.58119700	-5.81858200
C	-12.56573100	3.42524900	-8.45073300
H	-13.80905500	4.62083500	-9.75663100
H	-11.59823100	2.16195100	-6.98534300
H	-11.63383300	3.69756400	-8.95295500
C	-17.79534100	3.35293900	-6.89993700
C	-18.50544600	3.14909600	-8.09616600
C	-18.07941200	4.49597400	-6.13236800
C	-19.46686300	4.07204600	-8.51812200
H	-18.31317300	2.26828500	-8.71272400
C	-19.03522700	5.42148000	-6.55845700
H	-17.56018200	4.65323900	-5.18410200
C	-19.73120000	5.21134500	-7.75220000

H	-20.01306100	3.89817800	-9.44904900
H	-19.24699800	6.30023500	-5.94448500
H	-20.48611200	5.93007100	-8.08136300
Cl	-17.13384200	-0.13005600	-1.27051400
C	-11.41651600	1.40333700	-2.82954200
H	-10.94410000	1.42311100	-1.83750700
H	-11.36589400	0.38820000	-3.24066700
H	-10.89147100	2.10115500	-3.49915900
C	-17.46904200	-1.08240900	0.80838700
C	-18.84619500	-0.74897600	1.24884600
Cl	-18.99577600	-1.60900100	2.96871800
H	-16.65090800	-0.58635300	1.33589200
H	-17.27768900	-2.12312800	0.53571000
C	-19.06859500	0.73523400	1.50645100
H	-19.00537800	1.27876700	0.55080000
H	-20.05863200	0.91074300	1.95126200
H	-18.30467400	1.13691600	2.18818200
C	-19.93616900	-1.36933400	0.38410500
H	-20.92875100	-1.19940700	0.82551900
H	-19.90645300	-0.90829100	-0.61576000
H	-19.78452600	-2.45392700	0.27766900

UB3LYP-D3/def2-SVP-SMD(THF)//UB3LYP/def2-SVP(gas)
HF= -4825.5708796
UPBEPBE-D3/def2-SVP-SMD(THF)//UB3LYP/def2-SVP(gas)
HF= -4822.0607950

^{5}B-DCIB'-TS

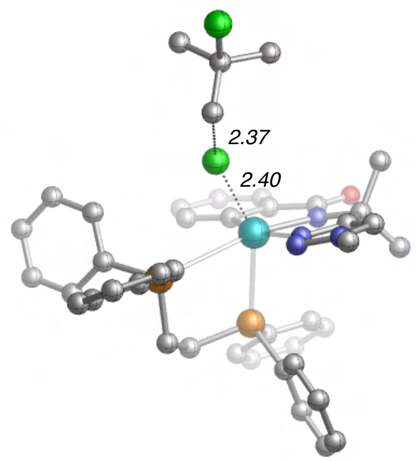

Zero-point correction= 0.790639 (Hartree/Particle)
Thermal correction to Energy= 0.844968
Thermal correction to Enthalpy= 0.845912
Thermal correction to Gibbs Free Energy= 0.690339
Sum of electronic and zero-point Energies= -4824.562330
Sum of electronic and thermal Energies= -4824.508002
Sum of electronic and thermal Enthalpies= -4824.507057
Sum of electronic and thermal Free Energies= -4824.662630

C	-19.18666100	1.70444300	-3.88160000
C	-19.58504900	3.02282500	-3.58542400
C	-20.89867000	3.44349400	-3.82724200
C	-21.83099700	2.55148100	-4.36165500
C	-21.44398400	1.23823200	-4.65083900
C	-20.12830600	0.81396100	-4.40868200
H	-21.15999100	4.47624200	-3.58182200
H	-22.85895300	2.87471400	-4.54799000
H	-22.17177500	0.53018100	-5.05959400
H	-19.85953500	-0.22340800	-4.62331900
C	-18.58135700	3.97999500	-2.99568700
O	-18.88697400	5.13241400	-2.67304600
C	-16.27143600	4.11320600	-2.17347900
C	-16.67661300	4.46444200	-0.71954100
H	-15.84063000	4.93582500	-0.17630900
H	-17.52680000	5.15778900	-0.74156000
H	-16.97693400	3.55258800	-0.18151600
C	-15.81664300	5.39240900	-2.91792400
H	-16.67584700	6.06983100	-3.00856800
H	-15.00988900	5.90838300	-2.37086400
H	-15.44186100	5.14394600	-3.92289000
C	-15.11942000	3.12847300	-2.12180700
N	-15.21848600	1.89327500	-2.68880100
N	-14.12380200	1.20741800	-2.50280100
C	-13.86063500	3.19347900	-1.54995800
H	-13.34758400	3.97174200	-0.99239900
N	-13.29246400	1.98708100	-1.81513300

N	-17.34619700	3.41209700	-2.87861600
Fe	-17.20042700	1.49813500	-3.49240400
P	-16.55190100	-1.33730200	-4.90540700
P	-16.70289500	1.94264500	-6.05611000
C	-16.10007200	-0.77909500	-6.63181300
H	-16.15745600	-1.60598200	-7.35832200
H	-15.03684600	-0.49226700	-6.58796000
C	-16.96345500	0.40426600	-7.09130300
H	-18.03435800	0.15345600	-7.01475200
H	-16.75292500	0.64671400	-8.14551400
C	-18.03327000	-2.41826400	-5.16504200
C	-18.75428000	-2.78605100	-4.01255000
C	-18.48955400	-2.87285100	-6.41416000
C	-19.88017000	-3.60680500	-4.10710500
H	-18.44366600	-2.40011200	-3.03854000
C	-19.62486100	-3.68538900	-6.50834600
H	-17.96446700	-2.59996200	-7.33191600
C	-20.31940800	-4.05958800	-5.35543600
H	-20.42606800	-3.88144200	-3.20086500
H	-19.96546200	-4.02711200	-7.48943400
H	-21.20607300	-4.69442300	-5.42991700
C	-15.21550400	-2.57540500	-4.55914100
C	-15.17176100	-3.85105900	-5.14855300
C	-14.20128700	-2.20751100	-3.65820500
C	-14.12873300	-4.73301000	-4.85545900
H	-15.96292000	-4.16389100	-5.83434100
C	-13.15681300	-3.09251200	-3.36848500
H	-14.23300000	-1.22518900	-3.17947600
C	-13.11740300	-4.35508800	-3.96553500
H	-14.10803300	-5.72248200	-5.32005900
H	-12.37529800	-2.79758100	-2.66267000
H	-12.30501200	-5.04854400	-3.73264500
C	-15.03234500	2.48720900	-6.65718400
C	-14.86390100	3.29129200	-7.79897500
C	-13.88789500	2.05210500	-5.96507600
C	-13.58534100	3.64302900	-8.23942800
H	-15.73784800	3.64969000	-8.34691600
C	-12.60887000	2.39735100	-6.41416500
H	-13.99286900	1.44383500	-5.06483900
C	-12.45374500	3.19523700	-7.55046300
H	-13.47351000	4.27091100	-9.12733700
H	-11.73000700	2.04432500	-5.86796300
H	-11.45452400	3.47097700	-7.89741100
C	-17.83395900	3.17322700	-6.83058200
C	-18.69657900	2.87748500	-7.89899100
C	-17.85129500	4.47188400	-6.29004800
C	-19.54585400	3.85987400	-8.41921200
H	-18.72244500	1.87952300	-8.33835900
C	-18.69574900	5.45152700	-6.81335100
H	-17.21008700	4.71706700	-5.44220700
C	-19.54643300	5.14833700	-7.88092300

H	-20.21315400	3.61065600	-9.24835600
H	-18.70098500	6.44946200	-6.36882900
H	-20.21518900	5.91238500	-8.28538100
Cl	-17.79258200	0.01366400	-1.70408600
C	-11.98913000	1.49138000	-1.40851500
H	-11.21557700	2.23758200	-1.63786500
H	-11.97659300	1.27132900	-0.33026000
H	-11.78959800	0.57096800	-1.96999600
C	-16.69842300	-1.06478300	0.09692500
C	-17.52284800	-0.83626900	1.30394400
Cl	-16.52036300	-1.72383300	2.69705500
H	-15.76183400	-0.51097400	0.01006900
H	-16.69459700	-2.06828100	-0.33369600
C	-17.61208400	0.62402100	1.72611100
H	-18.17941400	1.18199000	0.96444600
H	-18.12209500	0.71971100	2.69535100
H	-16.61138200	1.07196700	1.81395500
C	-18.87867500	-1.52855000	1.26245200
H	-19.39700800	-1.43116800	2.22716700
H	-19.49370200	-1.06142700	0.47661900
H	-18.76902000	-2.59839700	1.03234000

UB3LYP-D3/def2-SVP-SMD(THF)//UB3LYP/def2-SVP(gas)
HF= -4825.5586127
UPBEPBE-D3/def2-SVP-SMD(THF)//UB3LYP/def2-SVP(gas)
HF= -4822.0366622

^{1}B-DCIB''-TS

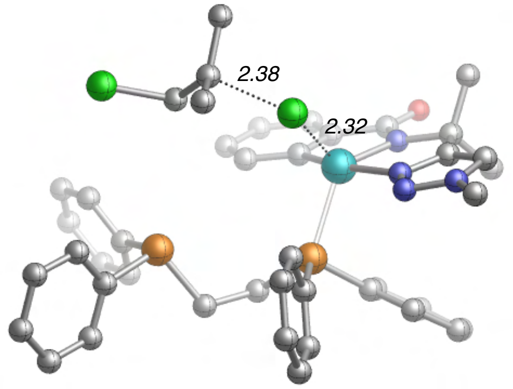

Zero-point correction= 0.792547 (Hartree/Particle)
Thermal correction to Energy= 0.846507
Thermal correction to Enthalpy= 0.847451
Thermal correction to Gibbs Free Energy= 0.696755
Sum of electronic and zero-point Energies= -4824.552131
Sum of electronic and thermal Energies= -4824.498171
Sum of electronic and thermal Enthalpies= -4824.497226
Sum of electronic and thermal Free Energies= -4824.647923

C	-18.87345800	1.68589100	-2.98391500
C	-19.89336200	2.63363000	-2.75235100
C	-21.25085400	2.29857200	-2.76806100
C	-21.62702200	0.97445800	-3.01088500
C	-20.63769700	0.00590400	-3.22203600
C	-19.27948600	0.36002700	-3.20395300
H	-21.98838600	3.08338700	-2.57861100
H	-22.68378100	0.69331600	-3.02733100
H	-20.92083400	-1.03571800	-3.40128000
H	-18.53338900	-0.42497900	-3.35557200
C	-19.43177100	4.02196900	-2.44713000
O	-20.19430100	4.95911300	-2.21523200
C	-17.38956800	5.36029600	-2.12028300
C	-17.70297800	5.80413400	-0.67105200
H	-17.14968800	6.72184700	-0.41057000
H	-18.77915800	5.99772200	-0.57804800
H	-17.42129300	5.01061500	0.03788500
C	-17.75344500	6.48419200	-3.12044800
H	-18.82841300	6.69249500	-3.05363500
H	-17.19235100	7.40596000	-2.89252300
H	-17.51352500	6.17395100	-4.14824700
C	-15.91711700	5.05458200	-2.23689300
N	-15.51754500	3.80366200	-2.60399200
N	-14.21640500	3.72483000	-2.64816100
C	-14.76503600	5.79188000	-2.04228300
H	-14.59200800	6.82173900	-1.74369500
N	-13.75077300	4.92325600	-2.31189400
N	-18.05427300	4.08790000	-2.44893500
Fe	-17.09248700	2.48695300	-2.82444400
P	-16.78637400	-2.10084700	-4.84315900

P	-16.31921900	1.72462800	-5.00389500
C	-16.59557600	-0.92226500	-6.28108900
H	-17.00915200	-1.36454100	-7.20196600
H	-15.51108100	-0.84398700	-6.45095500
C	-17.21661300	0.47609300	-6.10545800
H	-18.24552400	0.41188600	-5.71955300
H	-17.27640900	0.95415800	-7.09569100
C	-18.46831600	-2.85196700	-5.10714300
C	-18.94901300	-3.68882700	-4.08156200
C	-19.29155000	-2.62063900	-6.22067500
C	-20.20320900	-4.29497400	-4.18166600
H	-18.34005600	-3.86431800	-3.18969000
C	-20.55325200	-3.21830300	-6.31482700
H	-18.96241000	-1.96838400	-7.03208400
C	-21.01129900	-4.06079700	-5.29938000
H	-20.55458800	-4.94541000	-3.37638900
H	-21.18018700	-3.02150700	-7.18867600
H	-21.99688700	-4.52742600	-5.37426100
C	-15.68805500	-3.50726800	-5.36661500
C	-15.46047800	-3.87991900	-6.70286000
C	-15.05723300	-4.24168900	-4.34727900
C	-14.61924300	-4.95204000	-7.01110300
H	-15.94688200	-3.33919500	-7.51823200
C	-14.22417400	-5.32234200	-4.65524300
H	-15.22852700	-3.97275200	-3.30104600
C	-13.99932100	-5.67687600	-5.98771900
H	-14.45203200	-5.22738700	-8.05601200
H	-13.74661400	-5.88529400	-3.84892700
H	-13.34398700	-6.51757600	-6.23030100
C	-14.54212800	1.32943200	-5.29578400
C	-13.95610500	1.37819300	-6.57498300
C	-13.76733400	0.89971300	-4.20722000
C	-12.62269200	1.00657400	-6.75865300
H	-14.54242300	1.71167800	-7.43521600
C	-12.43307900	0.52142400	-4.39566600
H	-14.20939600	0.87021300	-3.20942300
C	-11.85891900	0.57493200	-5.66759500
H	-12.17893000	1.04937900	-7.75678500
H	-11.84176500	0.18456100	-3.54028200
H	-10.81676800	0.27840000	-5.81310200
C	-16.61665000	3.28985700	-5.94476400
C	-17.93075300	3.60478100	-6.33858000
C	-15.59740100	4.22707800	-6.18487200
C	-18.20980100	4.81112300	-6.98483300
H	-18.75109500	2.91573300	-6.12457300
C	-15.88081200	5.43408200	-6.83143200
H	-14.57476400	4.01479800	-5.86738200
C	-17.18516000	5.72811100	-7.23834500
H	-19.23658900	5.03812300	-7.28209000
H	-15.07453600	6.14850800	-7.01839000
H	-17.40471300	6.67199300	-7.74359500

Cl	-16.13087200	1.16721200	-1.17491600
C	-12.31884100	5.15300200	-2.24631600
H	-12.04237500	6.01724300	-2.86751100
H	-12.00374600	5.33460200	-1.20774500
H	-11.82307200	4.25218000	-2.62691000
C	-15.88341500	-0.96341500	-0.13420500
C	-14.43415100	-1.23782000	-0.40438400
H	-14.22781700	-1.31566500	-1.48208800
H	-13.79190400	-0.45556200	0.02730500
H	-14.14941000	-2.20251800	0.05914600
C	-16.26859500	-0.64325000	1.27835300
H	-15.57445900	0.07846800	1.73412500
H	-17.28930400	-0.23814200	1.33918100
H	-16.23799000	-1.57053100	1.88470500
C	-16.88017100	-1.67775900	-0.97175400
H	-17.90983900	-1.35952700	-0.77696600
H	-16.65895000	-1.61975400	-2.04322200
Cl	-16.85544500	-3.51320100	-0.59538300

UB3LYP-D3/def2-SVP-SMD(THF)//UB3LYP/def2-SVP(gas)
HF= -4825.5314415
UPBEPBE-D3/def2-SVP-SMD(THF)//UB3LYP/def2-SVP(gas)
HF= -4822.0372633

^{3}B-DCIB''-TS

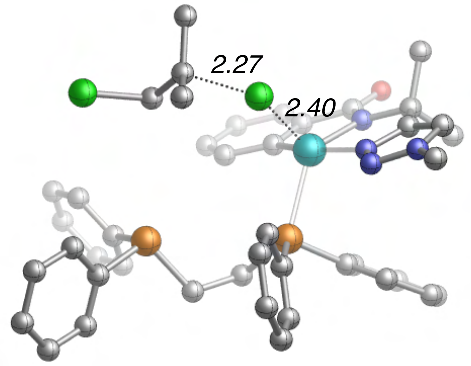

Zero-point correction= 0.792464 (Hartree/Particle)
Thermal correction to Energy= 0.846524
Thermal correction to Enthalpy= 0.847468
Thermal correction to Gibbs Free Energy= 0.694796
Sum of electronic and zero-point Energies= -4824.574204
Sum of electronic and thermal Energies= -4824.520144
Sum of electronic and thermal Enthalpies= -4824.519200
Sum of electronic and thermal Free Energies= -4824.671871

C	-18.93222800	1.73905900	-3.07392100
C	-19.94133300	2.68079700	-2.79063300
C	-21.29806500	2.34304200	-2.81126900
C	-21.67348600	1.02839000	-3.10220800
C	-20.68744300	0.06582200	-3.35384900
C	-19.32955800	0.42024900	-3.33602000
H	-22.03406600	3.11904900	-2.58344200
H	-22.73035000	0.74746200	-3.12093900
H	-20.97222900	-0.96927300	-3.56451700
H	-18.58074300	-0.35633600	-3.51456600
C	-19.47574300	4.05424400	-2.41760000
O	-20.24318000	4.97992500	-2.14709400
C	-17.42861800	5.36038100	-2.04145000
C	-17.72786400	5.75741800	-0.57582000
H	-17.17492700	6.66744100	-0.28822500
H	-18.80418000	5.94521400	-0.47091200
H	-17.44154100	4.94049200	0.10400200
C	-17.79242800	6.52081800	-3.00066800
H	-18.86753400	6.72500000	-2.92275400
H	-17.23074000	7.43456900	-2.74350900
H	-17.55691600	6.24466500	-4.03958900
C	-15.95667300	5.05749200	-2.18269900
N	-15.55159100	3.82924300	-2.61520500
N	-14.24949000	3.75954500	-2.67529200
C	-14.80533900	5.78936700	-1.96145200
H	-14.63418500	6.80392500	-1.61363700
N	-13.78911900	4.94016400	-2.28189300
N	-18.10735200	4.10867100	-2.40875500
Fe	-17.12607000	2.50940100	-2.86970100
P	-16.78781400	-2.10069500	-4.87615000
P	-16.33138100	1.67721900	-5.05840700

C	-16.61761600	-0.95925200	-6.34792200
H	-17.05451200	-1.42004500	-7.24896100
H	-15.53559100	-0.89889600	-6.53973700
C	-17.21380000	0.45187800	-6.19788100
H	-18.25722200	0.40973800	-5.84945600
H	-17.22886400	0.92871500	-7.19063000
C	-18.47197200	-2.85934700	-5.09358700
C	-18.91494800	-3.70648600	-4.05916700
C	-19.33319200	-2.62142700	-6.17654200
C	-20.16946800	-4.31631300	-4.12183900
H	-18.27420000	-3.88784100	-3.19089900
C	-20.59540800	-3.22331800	-6.23301300
H	-19.03394300	-1.96064900	-6.99262800
C	-21.01577400	-4.07594000	-5.20986900
H	-20.49122800	-4.97503000	-3.31093700
H	-21.25219900	-3.02167100	-7.08350100
H	-22.00152600	-4.54597100	-5.25540100
C	-15.69885600	-3.51800100	-5.39010000
C	-15.56610700	-3.96743900	-6.71573000
C	-14.97777300	-4.17827000	-4.38059300
C	-14.72661800	-5.04040900	-7.02432600
H	-16.12781600	-3.48684600	-7.52050400
C	-14.14493900	-5.25970600	-4.68800100
H	-15.08046300	-3.85357300	-3.34149900
C	-14.01341700	-5.68954800	-6.01050200
H	-14.63389100	-5.37652600	-8.06067300
H	-13.59591200	-5.76496200	-3.88900100
H	-13.35941800	-6.53137100	-6.25286400
C	-14.55053800	1.26705700	-5.30958500
C	-13.93574100	1.28413500	-6.57585100
C	-13.79864700	0.86847400	-4.19381000
C	-12.59819500	0.90972600	-6.71996300
H	-14.50276700	1.59601900	-7.45694200
C	-12.45983700	0.48841600	-4.34105600
H	-14.26352200	0.86495400	-3.20587500
C	-11.85814600	0.50856400	-5.60119800
H	-12.13222100	0.92690300	-7.70869500
H	-11.88720800	0.17603700	-3.46393700
H	-10.81271800	0.21009800	-5.71581700
C	-16.58147900	3.25456000	-5.99603200
C	-17.88474600	3.60221000	-6.39889000
C	-15.54031900	4.17188800	-6.21788900
C	-18.13209900	4.82131600	-7.03391600
H	-18.72162800	2.92881400	-6.19926500
C	-15.79214300	5.39251300	-6.85216700
H	-14.52544300	3.93451400	-5.89341500
C	-17.08609100	5.71964800	-7.26662000
H	-19.15098800	5.07332800	-7.33824500
H	-14.96900700	6.09148500	-7.02376600
H	-17.28098800	6.67408700	-7.76209900
Cl	-16.29318500	1.15268300	-1.07495200

C	-12.35769400	5.17766600	-2.22087800
H	-12.09037600	6.05391100	-2.82912400
H	-12.03963900	5.34420800	-1.18094300
H	-11.85882200	4.28627300	-2.61906300
C	-15.83681400	-0.87501400	-0.16026000
C	-14.37789900	-1.06539000	-0.46736600
H	-14.19055300	-1.09675000	-1.55093500
H	-13.76959000	-0.26338600	-0.02303000
H	-14.03441000	-2.02792000	-0.04254200
C	-16.19242300	-0.66190600	1.28398400
H	-15.53763600	0.08680500	1.75417900
H	-17.23690300	-0.33721300	1.40014400
H	-16.07096300	-1.61461800	1.83546900
C	-16.80111900	-1.66533200	-0.97886100
H	-17.84492600	-1.42035500	-0.75361900
H	-16.61605600	-1.57586500	-2.05532700
Cl	-16.63749300	-3.49072500	-0.62708300

UB3LYP-D3/def2-SVP-SMD(THF)//UB3LYP/def2-SVP(gas)
HF= -4825.5715044
UPBEPBE-D3/def2-SVP-SMD(THF)//UB3LYP/def2-SVP(gas)
HF= -4822.0634912

[5]B-DCIB''-TS

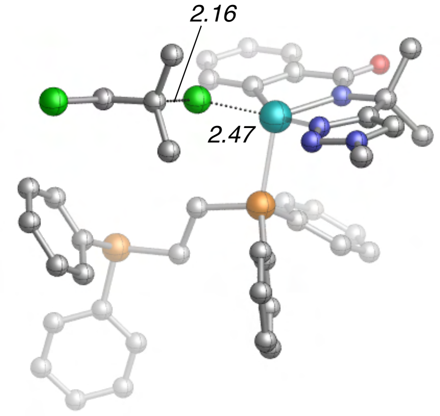

Zero-point correction= 0.791700 (Hartree/Particle)
Thermal correction to Energy= 0.845272
Thermal correction to Enthalpy= 0.846216
Thermal correction to Gibbs Free Energy= 0.692537
Sum of electronic and zero-point Energies= -4824.578348
Sum of electronic and thermal Energies= -4824.524776
Sum of electronic and thermal Enthalpies= -4824.523832
Sum of electronic and thermal Free Energies= -4824.677511

C	-18.48043900	1.20442500	-3.43169000
C	-19.15246900	2.27246300	-2.78555800
C	-20.53642900	2.23804100	-2.56765100
C	-21.28341800	1.13233300	-2.98064600
C	-20.63838000	0.05956000	-3.60660900
C	-19.25367300	0.10069600	-3.82784600
H	-20.99958900	3.09162200	-2.06585500
H	-22.36321200	1.10073500	-2.80880300
H	-21.21463900	-0.81672700	-3.92091300
H	-18.77484600	-0.76032200	-4.30660000
C	-18.36837100	3.47035400	-2.29244000
O	-18.91876200	4.40748700	-1.70680800
C	-16.08807300	4.35607900	-2.04117300
C	-16.16254100	4.48590600	-0.49899600
H	-15.40657700	5.19553800	-0.12299600
H	-17.16202900	4.84065200	-0.21808000
H	-15.98995700	3.50632300	-0.02686400
C	-16.29670500	5.74080900	-2.69985100
H	-17.30256000	6.10074100	-2.44615300
H	-15.55057100	6.47279400	-2.34693300
H	-16.22012900	5.65807600	-3.79453700
C	-14.71481400	3.81965300	-2.40692700
N	-14.57426300	2.64628600	-3.08671500
N	-13.31257900	2.36133100	-3.26936600
C	-13.43301600	4.28121800	-2.16197100
H	-13.05553300	5.16360600	-1.65366400
N	-12.61253700	3.34591200	-2.71337200
N	-17.03168800	3.36481700	-2.55221200
Fe	-16.52137700	1.77043400	-3.62683700

P	-16.59143700	2.32131300	-6.14648600
C	-17.59513300	0.85821600	-8.46932900
H	-18.32807000	1.65402300	-8.67953400
H	-16.68345100	1.08855300	-9.04244500
C	-17.30455700	0.80116000	-6.97012300
H	-16.57857400	0.00645500	-6.73412100
H	-18.20823400	0.55167100	-6.39152000
C	-15.12291900	2.71037700	-7.20426500
C	-15.19554400	3.34551100	-8.45819900
C	-13.86614100	2.29184800	-6.73421300
C	-14.04221500	3.55162100	-9.21991700
H	-16.15818100	3.68889300	-8.84211300
C	-12.71381300	2.49012600	-7.50221800
H	-13.78820100	1.81483200	-5.75533700
C	-12.79833500	3.12173300	-8.74552900
H	-14.11646700	4.04762700	-10.19130700
H	-11.74535600	2.15307500	-7.12264400
H	-11.89791000	3.28210700	-9.34433500
C	-17.80177500	3.66697100	-6.48631300
C	-19.18242900	3.40166500	-6.43715900
C	-17.37939500	4.99978400	-6.64503600
C	-20.11127000	4.43719400	-6.57176500
H	-19.54614500	2.38606500	-6.27031400
C	-18.31076900	6.03246900	-6.77996000
H	-16.31357800	5.23873100	-6.66364700
C	-19.68024500	5.75438100	-6.74796500
H	-21.17890600	4.20912300	-6.52608200
H	-17.96220700	7.06107600	-6.90439100
H	-20.40849500	6.56294700	-6.84862300
C	-11.16068900	3.31685600	-2.74898200
H	-10.78693000	2.43384600	-2.21110800
H	-10.81305200	3.27963700	-3.79099700
H	-10.77536900	4.22492800	-2.26924200
P	-18.35880200	-0.74643800	-9.09218900
C	-18.29915000	-0.49801400	-10.92906700
C	-18.33142300	0.77060700	-11.53411400
C	-18.34797300	-1.63220500	-11.76322700
C	-18.39834300	0.90053200	-12.92613000
H	-18.30669000	1.67614300	-10.92479700
C	-18.40841800	-1.50230000	-13.15140200
H	-18.33369800	-2.63196700	-11.32016500
C	-18.43425500	-0.23312400	-13.74035800
H	-18.42060900	1.89793900	-13.37364300
H	-18.43848900	-2.39802000	-13.77767900
H	-18.48479700	-0.13048300	-14.82731900
C	-16.93771800	-1.92024300	-8.83895100
C	-17.14684900	-3.03316300	-8.00793200
C	-15.66470300	-1.73649900	-9.40948700
C	-16.10907300	-3.93509900	-7.74192300
H	-18.13360200	-3.19201300	-7.56395700
C	-14.62723800	-2.63278400	-9.14203100

H	-15.48256600	-0.88685200	-10.07314500
C	-14.84588100	-3.73349200	-8.30406600
H	-16.28583700	-4.79618100	-7.09205200
H	-13.64288000	-2.47412300	-9.59084200
H	-14.03253400	-4.42869400	-8.08238700
C	-13.99952600	-1.86358000	-3.82258500
Cl	-15.71152500	-0.55509500	-3.74120200
C	-13.04311000	-1.17036100	-4.75604300
H	-13.47381200	-1.04116900	-5.75935500
H	-12.75179900	-0.18799300	-4.35751600
H	-12.13579200	-1.79310600	-4.85665200
C	-13.55805200	-1.94995700	-2.38497400
H	-12.70981700	-2.65525000	-2.31667000
H	-13.22518000	-0.96961600	-2.01582900
H	-14.36228800	-2.32123000	-1.73370600
C	-14.69422100	-3.08222600	-4.37222500
H	-15.45397000	-3.46585600	-3.68128400
H	-15.14149400	-2.89298700	-5.35423600
Cl	-13.49605300	-4.45358500	-4.62612200

UB3LYP-D3/def2-SVP-SMD(THF)//UB3LYP/def2-SVP(gas)
HF= -4825.5700971
UPBEPBE-D3/def2-SVP-SMD(THF)//UB3LYP/def2-SVP(gas)
HF= -4822.0414913

[1]B-DCIB'''-TS

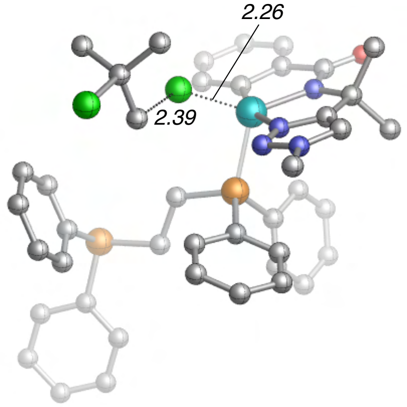

Zero-point correction= 0.792478 (Hartree/Particle)
Thermal correction to Energy= 0.845157
Thermal correction to Enthalpy= 0.846101
Thermal correction to Gibbs Free Energy= 0.698418
Sum of electronic and zero-point Energies= -4824.548563
Sum of electronic and thermal Energies= -4824.495884
Sum of electronic and thermal Enthalpies= -4824.494940
Sum of electronic and thermal Free Energies= -4824.642623

C	-18.46915100	1.18827400	-3.28949300
C	-19.10850800	2.09969300	-2.41581800
C	-20.44724900	1.97060200	-2.03198700
C	-21.20120200	0.89543000	-2.51103900
C	-20.59445900	-0.03775600	-3.35885300
C	-19.25018400	0.10671900	-3.73627100
H	-20.87152200	2.71817400	-1.35584800
H	-22.24911200	0.77863200	-2.22065000
H	-21.17118500	-0.89208700	-3.72834600
H	-18.81145200	-0.66031700	-4.38340800
C	-18.26704800	3.22971100	-1.90263000
O	-18.70070200	4.11979400	-1.16683000
C	-15.97472700	4.12421200	-1.98342100
C	-15.77472500	4.18665400	-0.44989800
H	-14.97474000	4.89788000	-0.18347200
H	-16.71267000	4.50552100	0.02133800
H	-15.50422500	3.19285600	-0.06084800
C	-16.32420500	5.52816100	-2.53333700
H	-17.27070700	5.85888400	-2.08604500
H	-15.53559700	6.26066500	-2.29230200
H	-16.44589300	5.48950700	-3.62681900
C	-14.69612400	3.63127500	-2.62204500
N	-14.73179300	2.51476300	-3.40731300
N	-13.53793100	2.20937400	-3.83953400
C	-13.37654100	4.03971200	-2.58778800
H	-12.87646800	4.86824800	-2.09444300
N	-12.70869600	3.12900100	-3.34963000
N	-16.98244400	3.12365100	-2.36869600
Fe	-16.56491600	1.69414200	-3.58408700

P	-16.77077200	2.12156700	-5.93517800
C	-17.58062200	0.70336600	-8.37053000
H	-18.36550300	1.43241800	-8.62674800
H	-16.65153500	1.02968000	-8.86486100
C	-17.41405900	0.62925900	-6.85176600
H	-16.74906000	-0.19753200	-6.55502700
H	-18.37757300	0.41634400	-6.36311100
C	-15.16726500	2.52591100	-6.76297300
C	-14.66463800	3.83952800	-6.71412000
C	-14.35331300	1.52391200	-7.31922100
C	-13.39901800	4.14338600	-7.22168900
H	-15.27124000	4.63857800	-6.28092000
C	-13.08555000	1.82741200	-7.82615600
H	-14.70099500	0.49004300	-7.36659700
C	-12.60350300	3.13810700	-7.78225500
H	-13.03619000	5.17437400	-7.18538200
H	-12.47625100	1.03152700	-8.26262300
H	-11.61680400	3.37676500	-8.18794300
C	-17.89119000	3.48777200	-6.47883300
C	-17.78926600	4.09540100	-7.74514400
C	-18.91078700	3.90505900	-5.60718800
C	-18.68842100	5.09349600	-8.12789000
H	-16.99668900	3.79747800	-8.43471500
C	-19.81202300	4.90204500	-5.99560500
H	-19.00607700	3.44354100	-4.62408000
C	-19.70320100	5.49840100	-7.25368600
H	-18.59558800	5.55741500	-9.11354200
H	-20.59829300	5.21205800	-5.30306300
H	-20.40561100	6.28026900	-7.55429100
C	-11.29752700	3.09528500	-3.68698000
H	-11.09855800	2.14686300	-4.19949800
H	-11.04171800	3.92808500	-4.35901700
H	-10.68699600	3.15849600	-2.77473900
P	-18.13365300	-0.95395700	-9.07089500
C	-18.01748800	-0.66528900	-10.89745000
C	-17.90321000	0.60256600	-11.49248700
C	-18.15266900	-1.78599300	-11.74082200
C	-17.91260600	0.74372400	-12.88560200
H	-17.80714000	1.49828400	-10.87631000
C	-18.15049900	-1.64659000	-13.12890200
H	-18.25995900	-2.78268100	-11.30240900
C	-18.03181900	-0.37778700	-13.70818300
H	-17.82328300	1.74008000	-13.32693000
H	-18.24969600	-2.53182700	-13.76276900
H	-18.03715700	-0.26597400	-14.79540700
C	-16.59559900	-1.95855500	-8.76027100
C	-16.65864000	-2.96190800	-7.77877000
C	-15.37405500	-1.74570400	-9.42578700
C	-15.52759200	-3.72132400	-7.45467500
H	-17.60484600	-3.15051900	-7.26392100
C	-14.24502500	-2.50407100	-9.10509800

H	-15.30682000	-0.98346900	-10.20658900
C	-14.31716000	-3.49155400	-8.11447800
H	-15.59781200	-4.50102700	-6.69133800
H	-13.30395400	-2.32817700	-9.63346700
H	-13.43228800	-4.08116900	-7.86127600
C	-13.87102400	-1.28379100	-4.50755600
Cl	-15.96209100	-0.48408900	-3.66390700
H	-13.42732500	-0.29126000	-4.61685300
H	-14.27030400	-1.75172900	-5.41049900
C	-13.33450200	-2.18157200	-3.44866300
C	-14.15359200	-3.44493600	-3.22825700
H	-14.34583300	-3.96428900	-4.17785700
H	-13.62842700	-4.13297000	-2.55053800
H	-15.12019800	-3.16904700	-2.77827800
C	-12.96293700	-1.46999900	-2.15615200
H	-12.42212700	-2.15018400	-1.48287000
H	-12.33408100	-0.59057200	-2.35363700
H	-13.88554200	-1.13219500	-1.65939600
Cl	-11.71417000	-2.70379200	-4.28854000

UB3LYP-D3/def2-SVP-SMD(THF)//UB3LYP/def2-SVP(gas)
HF= -4825.5367419
UPBEPBE-D3/def2-SVP-SMD(THF)//UB3LYP/def2-SVP(gas)
HF= -4822.0415871

^{3}**B-DCIB'''-TS**

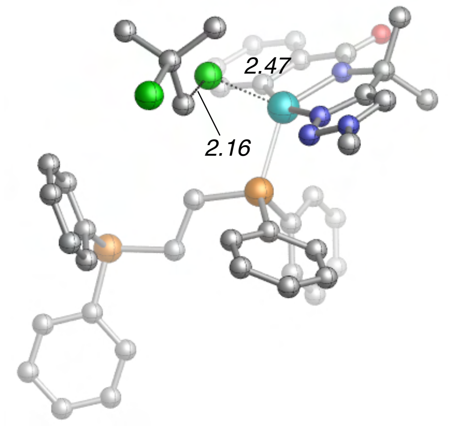

Zero-point correction= 0.792693 (Hartree/Particle)
Thermal correction to Energy= 0.845502
Thermal correction to Enthalpy= 0.846447
Thermal correction to Gibbs Free Energy= 0.696891
Sum of electronic and zero-point Energies= -4824.577520
Sum of electronic and thermal Energies= -4824.524710
Sum of electronic and thermal Enthalpies= -4824.523766
Sum of electronic and thermal Free Energies= -4824.673322

C	-18.81549700	1.51616200	-3.33419200
C	-19.36933700	2.35441800	-2.33773200
C	-20.71186000	2.28115600	-1.95361600
C	-21.54995200	1.33485600	-2.55091700
C	-21.02487800	0.46719200	-3.51506300
C	-19.67778100	0.55920300	-3.89775700
H	-21.07071300	2.96466000	-1.17905600
H	-22.60182800	1.26350000	-2.25983200
H	-21.66813600	-0.29082900	-3.97371100
H	-19.30485600	-0.15439400	-4.64044400
C	-18.42924400	3.31705800	-1.66874700
O	-18.79250800	4.12522100	-0.80910900
C	-16.06801200	3.99789700	-1.62273800
C	-15.85451300	3.81171600	-0.10213200
H	-15.00269400	4.41269600	0.25920100
H	-16.76459900	4.12549200	0.42458100
H	-15.66100000	2.75268200	0.12839700
C	-16.30196600	5.49380500	-1.95041900
H	-17.21614100	5.82870300	-1.44328500
H	-15.45337500	6.11506900	-1.61799700
H	-16.43099000	5.62710700	-3.03579900
C	-14.84137300	3.51378000	-2.36130700
N	-14.98012800	2.59949100	-3.36704300
N	-13.81648200	2.27745500	-3.86589800
C	-13.48841800	3.77478900	-2.25681600
H	-12.91474200	4.42740500	-1.60499000
N	-12.90563700	2.98583000	-3.20256000
N	-17.15549300	3.15380500	-2.13525500
Fe	-16.89531400	1.93660200	-3.61802400

P	-16.91955900	2.18578100	-6.04610400
C	-17.65669300	0.71404600	-8.48218100
H	-18.40652500	1.45412200	-8.80337800
H	-16.69440400	0.99799100	-8.93709400
C	-17.57122300	0.68987900	-6.95469500
H	-16.95603100	-0.15172100	-6.59681200
H	-18.56753700	0.52883600	-6.51503700
C	-15.27889500	2.52835000	-6.82612900
C	-14.74936100	3.83157800	-6.79535100
C	-14.47063600	1.49389000	-7.32821900
C	-13.46078200	4.09341300	-7.26642100
H	-15.35174400	4.65443100	-6.40279800
C	-13.17962500	1.75540300	-7.79826000
H	-14.83921700	0.46662000	-7.36018000
C	-12.66967300	3.05603000	-7.77179800
H	-13.07514400	5.11642300	-7.24347700
H	-12.57417200	0.93452300	-8.19165800
H	-11.66417000	3.26194400	-8.14818900
C	-17.97872000	3.57509700	-6.65285100
C	-17.85077200	4.12065900	-7.94477400
C	-18.96937600	4.08569000	-5.79715200
C	-18.69872600	5.14600300	-8.36957600
H	-17.07496100	3.75419300	-8.62036700
C	-19.81877000	5.11093900	-6.22676300
H	-19.08052700	3.67694600	-4.79114300
C	-19.68659200	5.64211800	-7.51150800
H	-18.58657500	5.56067300	-9.37494800
H	-20.58335800	5.49408600	-5.54659800
H	-20.34890700	6.44527000	-7.84516200
C	-11.50053700	2.87768700	-3.55123900
H	-10.90326700	2.63961300	-2.65912600
H	-11.40143400	2.07285100	-4.28902800
H	-11.14011800	3.81893100	-3.99245400
P	-18.21948300	-0.95254800	-9.15335000
C	-18.00169700	-0.73339600	-10.98012000
C	-17.80625300	0.50646500	-11.61177600
C	-18.13900600	-1.87826700	-11.78998600
C	-17.73925300	0.59681000	-13.00752300
H	-17.70505000	1.41973900	-11.02281100
C	-18.06056200	-1.78999000	-13.18004900
H	-18.30873600	-2.85328500	-11.32344400
C	-17.86160700	-0.54873000	-13.79581500
H	-17.58749200	1.57211400	-13.47783200
H	-18.16302500	-2.69333500	-13.78726400
H	-17.80721400	-0.47669100	-14.88504500
C	-16.72733800	-1.98254500	-8.72567200
C	-16.86614000	-2.94103200	-7.70761400
C	-15.46843300	-1.82756000	-9.33511200
C	-15.77226600	-3.71037800	-7.29205900
H	-17.84221400	-3.08620100	-7.23614700
C	-14.37659600	-2.59606800	-8.92282600

H	-15.34187200	-1.10203800	-10.14304100
C	-14.52375400	-3.53626000	-7.89544200
H	-15.90117500	-4.45434300	-6.50118300
H	-13.40521300	-2.46413300	-9.40724300
H	-13.66703500	-4.13066900	-7.56816900
C	-14.41990800	-1.11026900	-4.36017900
Cl	-16.28905500	-0.45046400	-3.51127000
H	-13.96383100	-0.13108500	-4.53105200
H	-14.78386100	-1.61616900	-5.25874000
C	-13.73205200	-1.99602900	-3.35697200
C	-14.46747400	-3.30187100	-3.08667800
H	-14.69614300	-3.82903100	-4.02394000
H	-13.86074400	-3.96240500	-2.45096000
H	-15.41437000	-3.08168000	-2.56950800
C	-13.31611000	-1.27202700	-2.08331100
H	-12.69685700	-1.92648200	-1.45344600
H	-12.74677800	-0.36071500	-2.31459600
H	-14.21773200	-0.98457300	-1.52089100
Cl	-12.16252500	-2.41883500	-4.29976300

UB3LYP-D3/def2-SVP-SMD(THF)//UB3LYP/def2-SVP(gas)
HF= -4825.5753145
UPBEPBE-D3/def2-SVP-SMD(THF)//UB3LYP/def2-SVP(gas)
HF= -4822.0616158

^{5}B-DCIB'''-TS

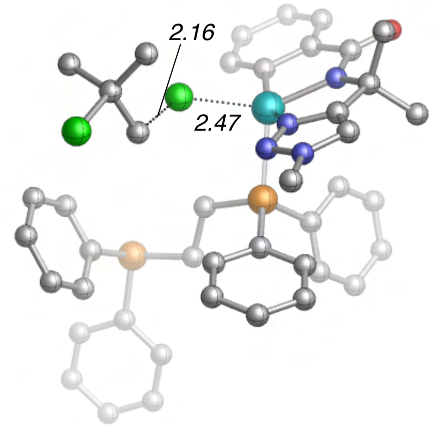

Zero-point correction= 0.791404 (Hartree/Particle)
Thermal correction to Energy= 0.844876
Thermal correction to Enthalpy= 0.845820
Thermal correction to Gibbs Free Energy= 0.692528
Sum of electronic and zero-point Energies= -4824.575065
Sum of electronic and thermal Energies= -4824.521593
Sum of electronic and thermal Enthalpies= -4824.520649
Sum of electronic and thermal Free Energies= -4824.673942

C	-18.90473000	1.23950400	-3.10456900
C	-19.36725400	2.28314000	-2.25881500
C	-20.70004400	2.33168700	-1.82498900
C	-21.60089800	1.33906400	-2.21721000
C	-21.16643200	0.29627300	-3.04372900
C	-19.83469200	0.25376700	-3.47813600
H	-21.00134200	3.15741700	-1.17535100
H	-22.64007600	1.37387300	-1.87706200
H	-21.86752200	-0.48724800	-3.34914100
H	-19.52223200	-0.57936600	-4.11836500
C	-18.42056700	3.37750800	-1.78974600
O	-18.81336600	4.27960200	-1.04092500
C	-16.08559700	4.13565000	-1.86035500
C	-15.89746800	4.14465100	-0.32182600
H	-15.04899400	4.78562800	-0.02914200
H	-16.81534500	4.51909800	0.14841600
H	-15.70628500	3.12315700	0.04186400
C	-16.31681400	5.57702000	-2.37264500
H	-17.24367900	5.96205600	-1.92694600
H	-15.48223800	6.24495500	-2.09945100
H	-16.42525700	5.58019800	-3.46843600
C	-14.82276100	3.56493900	-2.48607200
N	-14.85111000	2.38942600	-3.17405400
N	-13.65817400	2.06746300	-3.59708100
C	-13.50424300	3.98883800	-2.49807300
H	-13.00862800	4.86047000	-2.08075000
N	-12.83419100	3.02891800	-3.19305900
N	-17.15634700	3.23498400	-2.27758100
Fe	-16.95608500	1.69884100	-3.54846200

P	-16.83929900	2.21379300	-6.04774400
C	-17.63845500	0.75816000	-8.46007000
H	-18.38763600	1.51064700	-8.75307500
H	-16.68422100	1.03798100	-8.93488900
C	-17.51127200	0.71664800	-6.93630100
H	-16.88125800	-0.12326100	-6.60177200
H	-18.49300600	0.54651800	-6.46409700
C	-15.18738600	2.53528700	-6.80186100
C	-14.63558500	3.82920100	-6.74352300
C	-14.38804500	1.49170200	-7.30182900
C	-13.33429700	4.07384600	-7.18883300
H	-15.23093300	4.65797100	-6.35234300
C	-13.08487700	1.73721400	-7.74544900
H	-14.77496300	0.47198000	-7.35339800
C	-12.55280000	3.02871100	-7.69410400
H	-12.93165600	5.08958800	-7.14645000
H	-12.48672000	0.91106200	-8.13869900
H	-11.53803800	3.22157800	-8.05206300
C	-17.89595900	3.60576900	-6.63776500
C	-17.75846400	4.20030100	-7.90627800
C	-18.90859700	4.06362200	-5.77715800
C	-18.61991000	5.22541800	-8.30408500
H	-16.96754800	3.87055500	-8.58373300
C	-19.77306700	5.08620400	-6.18151000
H	-19.02239200	3.61814200	-4.78667300
C	-19.63052400	5.66836900	-7.44320400
H	-18.50175600	5.68081600	-9.29095100
H	-20.55492700	5.42912000	-5.49963300
H	-20.30329700	6.47095100	-7.75657400
C	-11.42109600	2.96106100	-3.52526500
H	-10.96000400	2.08592400	-3.04511300
H	-11.30396500	2.88156600	-4.61490700
H	-10.92826500	3.87368800	-3.16802900
P	-18.23508300	-0.89402400	-9.13617300
C	-18.08551900	-0.64375100	-10.96588200
C	-17.89836600	0.60434700	-11.58365700
C	-18.26832600	-1.77270900	-11.78897300
C	-17.88357000	0.71791500	-12.97925200
H	-17.76318600	1.50616500	-10.98395800
C	-18.24188400	-1.66144300	-13.17933600
H	-18.43272500	-2.75346300	-11.33265500
C	-18.05066100	-0.41226200	-13.78136700
H	-17.73748000	1.69927000	-13.43865100
H	-18.37914300	-2.55284600	-13.79721700
H	-18.03707100	-0.32208300	-14.87051400
C	-16.73423100	-1.93962500	-8.78425400
C	-16.84065700	-2.91727900	-7.78075300
C	-15.50025400	-1.78363200	-9.44253700
C	-15.73956900	-3.70786000	-7.42948600
H	-17.79704800	-3.06154300	-7.27041500
C	-14.40129500	-2.57347100	-9.09472500

H	-15.39975400	-1.04235200	-10.23977900
C	-14.51663300	-3.53557100	-8.08357200
H	-15.84321100	-4.46696400	-6.64946700
H	-13.45022200	-2.44237000	-9.61811900
H	-13.65567500	-4.15114300	-7.81054100
C	-14.26613500	-1.32772400	-4.34495800
Cl	-16.21032000	-0.64530200	-3.70923500
H	-13.77332000	-0.35475200	-4.42455900
H	-14.52723800	-1.79897600	-5.29657700
C	-13.72657300	-2.25384000	-3.29078900
C	-14.51720900	-3.54727100	-3.14417700
H	-14.64298300	-4.04727500	-4.11533300
H	-14.00474500	-4.23713000	-2.45858300
H	-15.51407100	-3.31678400	-2.73713700
C	-13.44879900	-1.56984200	-1.95877900
H	-12.91677900	-2.25158300	-1.28001900
H	-12.84508000	-0.66222300	-2.09862000
H	-14.40472700	-1.28027500	-1.49585000
Cl	-12.06221600	-2.70112900	-4.04859000

UB3LYP-D3/def2-SVP-SMD(THF)//UB3LYP/def2-SVP(gas)
HF= -4825.5677959
UPBEPBE-D3/def2-SVP-SMD(THF)//UB3LYP/def2-SVP(gas)
HF= -4822.0359138

^{2}C

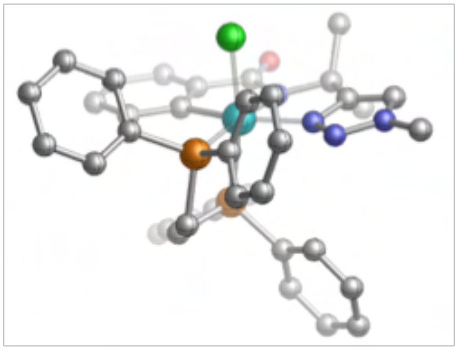

Zero-point correction= 0.684867 (Hartree/Particle)
Thermal correction to Energy= 0.729617
Thermal correction to Enthalpy= 0.730561
Thermal correction to Gibbs Free Energy= 0.605430
Sum of electronic and zero-point Energies= -4207.531154
Sum of electronic and thermal Energies= -4207.486404
Sum of electronic and thermal Enthalpies= -4207.485460
Sum of electronic and thermal Free Energies= -4207.610591

C	-18.71124500	1.41103400	-3.76394800
C	-19.22859700	2.63561400	-3.29237800
C	-20.59826700	2.91857500	-3.28652300
C	-21.50184000	1.97151000	-3.77046000
C	-21.01648800	0.75231500	-4.25602700
C	-19.64077800	0.47825600	-4.25408800
H	-20.92410500	3.88616500	-2.89602900
H	-22.57587700	2.17719600	-3.77054500
H	-21.71384400	-0.00130400	-4.63551500
H	-19.31938700	-0.49545600	-4.62859700
C	-18.24077900	3.62921000	-2.78539100
O	-18.55888000	4.73008600	-2.32281500
C	-15.86899100	3.84904800	-2.21497400
C	-16.13276800	3.98800600	-0.69406800
H	-15.27164000	4.46111300	-0.19286500
H	-17.02262800	4.61071500	-0.53845800
H	-16.30647900	2.99593000	-0.25444800
C	-15.60268800	5.24058400	-2.83991100
H	-16.51299900	5.84686600	-2.75119600
H	-14.77614500	5.75365100	-2.32042800
H	-15.32911800	5.14440500	-3.90250900
C	-14.65621400	2.97832300	-2.41699700
N	-14.76214600	1.80698100	-3.10155100
N	-13.60965500	1.20023100	-3.16503700
C	-13.33402900	3.08614700	-2.02815900
H	-12.79441700	3.83676400	-1.45821100
N	-12.73861500	1.96294300	-2.51519700
N	-16.96826300	3.14574000	-2.89940500
Fe	-16.73818700	1.33516500	-3.54942400
P	-16.25443400	-0.89487500	-4.42685600
P	-16.81619500	1.95951100	-5.83123900

C	-16.14987900	-0.70802600	-6.28632200
H	-16.33682400	-1.65610500	-6.81059300
H	-15.11602000	-0.41293400	-6.52140500
C	-17.12229700	0.37546500	-6.76896100
H	-18.16269200	0.07934200	-6.56852200
H	-17.01622000	0.55024500	-7.85105600
C	-17.43873100	-2.30521400	-4.16627200
C	-18.25613700	-2.33189400	-3.02389700
C	-17.47789900	-3.39421300	-5.05543000
C	-19.10262400	-3.42042400	-2.78992300
H	-18.22317100	-1.49956100	-2.31754600
C	-18.33013600	-4.47571500	-4.82086200
H	-16.83225900	-3.42154500	-5.93604800
C	-19.14741800	-4.49049200	-3.68634800
H	-19.73451100	-3.42361200	-1.89817000
H	-18.34966900	-5.31175800	-5.52494000
H	-19.81398900	-5.33703600	-3.50157000
C	-14.67355800	-1.72783000	-3.94921300
C	-13.77021000	-2.26174200	-4.88090400
C	-14.39560800	-1.85702000	-2.57644300
C	-12.60818500	-2.91249200	-4.45069500
H	-13.95894000	-2.18435800	-5.95351100
C	-13.24006600	-2.51500000	-2.15194400
H	-15.08135100	-1.41809900	-1.84601200
C	-12.34112600	-3.04319000	-3.08581800
H	-11.91524700	-3.32522200	-5.18888400
H	-13.03924800	-2.61319400	-1.08166100
H	-11.43811100	-3.56034600	-2.75030600
C	-15.29744900	2.59205800	-6.70624500
C	-15.38925100	3.49013600	-7.78624900
C	-14.02500800	2.11702400	-6.33969900
C	-14.24439200	3.89905800	-8.47568600
H	-16.36109100	3.87707500	-8.09534400
C	-12.88230600	2.52189400	-7.03722100
H	-13.91081800	1.43389700	-5.49859000
C	-12.98640500	3.41624500	-8.10507300
H	-14.33999900	4.59965700	-9.30932000
H	-11.90429400	2.13653900	-6.73670400
H	-12.09216300	3.73726700	-8.64551200
C	-18.11046000	3.13398900	-6.39599400
C	-19.16067600	2.75325800	-7.24595800
C	-18.05028900	4.46463000	-5.94451000
C	-20.12514000	3.68547500	-7.64106600
H	-19.24585200	1.72689000	-7.60457600
C	-19.01497600	5.39195600	-6.33975400
H	-17.25288500	4.77771600	-5.26998100
C	-20.05521400	5.00531900	-7.19016000
H	-20.93869200	3.37190700	-8.30003400
H	-18.95938700	6.41716100	-5.96656400
H	-20.81442300	5.73029600	-7.49436500
Cl	-16.93357600	0.41685900	-1.42799700

C	-11.35081800	1.54982900	-2.40679000
H	-10.70018600	2.21862500	-2.99033300
H	-11.03518100	1.56180700	-1.35372900
H	-11.27913000	0.52806500	-2.79839500

UB3LYP-D3/def2-SVP-SMD(THF)//UB3LYP/def2-SVP(gas)
HF= -4208.4101327
UPBEPBE-D3/def2-SVP-SMD(THF)//UB3LYP/def2-SVP(gas)
HF= -4205.3430888

^{4}C

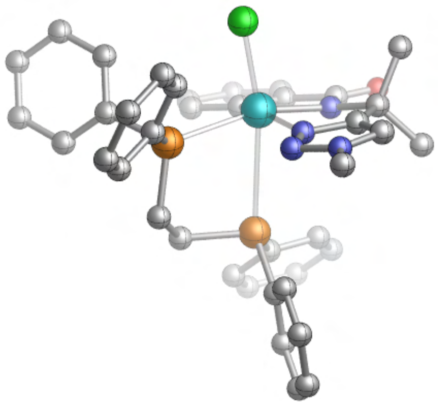

Zero-point correction= 0.682816 (Hartree/Particle)
Thermal correction to Energy= 0.728788
Thermal correction to Enthalpy= 0.729732
Thermal correction to Gibbs Free Energy= 0.597594
Sum of electronic and zero-point Energies= -4207.539832
Sum of electronic and thermal Energies= -4207.493860
Sum of electronic and thermal Enthalpies= -4207.492916
Sum of electronic and thermal Free Energies= -4207.625055

C	-18.70428500	1.41609800	-3.37594700
C	-19.20573600	2.66571700	-2.96953400
C	-20.56285800	2.98356000	-3.06997100
C	-21.45205400	2.04344100	-3.59467800
C	-20.97170700	0.79553200	-4.00524000
C	-19.60884400	0.47944100	-3.89196400
H	-20.89152900	3.96859900	-2.72811000
H	-22.51667300	2.27741800	-3.68116700
H	-21.66255800	0.05036600	-4.41108000
H	-19.28420600	-0.51529900	-4.19727300
C	-18.21929900	3.64472400	-2.41948000
O	-18.53541300	4.75303900	-1.98511300
C	-15.82113100	3.89415600	-1.92049700
C	-15.95490500	4.03394800	-0.38451500
H	-15.07980100	4.55085700	0.04361800
H	-16.85765100	4.61601600	-0.15629800
H	-16.04485900	3.03828700	0.07381000
C	-15.66208900	5.28404400	-2.58338500
H	-16.56033400	5.88231100	-2.39007000
H	-14.78279400	5.81011400	-2.17589500
H	-15.52765600	5.17847700	-3.67116500
C	-14.59931300	3.06944700	-2.24366600
N	-14.73571100	1.86010700	-2.85253000
N	-13.57304400	1.31056500	-3.07199100
C	-13.24188000	3.26494800	-2.06977600
H	-12.66519200	4.07337900	-1.63003000
N	-12.66224800	2.15120600	-2.59802600
N	-16.95254300	3.13363000	-2.48067700
Fe	-16.74576800	1.26997700	-2.96936600
P	-16.25626900	-0.90282900	-4.24005500

P	-16.64746000	2.13116600	-6.00439500
C	-16.00438000	-0.61024300	-6.06559300
H	-16.05779700	-1.56341700	-6.61281900
H	-14.96490900	-0.26052800	-6.17104300
C	-16.97585800	0.41086800	-6.67640300
H	-18.01969100	0.16478200	-6.42356500
H	-16.88589400	0.39648700	-7.77455800
C	-17.51567100	-2.25830400	-4.13529500
C	-17.97683000	-2.61255700	-2.85320900
C	-18.01670000	-2.94637900	-5.25211000
C	-18.90362000	-3.64528200	-2.69896000
H	-17.62790800	-2.05724900	-1.97811900
C	-18.95148100	-3.97494100	-5.09186300
H	-17.69172800	-2.69096400	-6.26226600
C	-19.39349200	-4.33039100	-3.81558500
H	-19.25303800	-3.90727400	-1.69709100
H	-19.33351400	-4.49877400	-5.97204700
H	-20.12323000	-5.13469600	-3.69166900
C	-14.71666300	-1.82421500	-3.76323300
C	-14.00308800	-2.59741200	-4.69585300
C	-14.27448900	-1.78925800	-2.43042400
C	-12.86783300	-3.31370300	-4.30680700
H	-14.32679000	-2.65921200	-5.73655100
C	-13.14496100	-2.51676100	-2.04346500
H	-14.81897200	-1.19063300	-1.69570400
C	-12.43605200	-3.27686400	-2.97773700
H	-12.32510200	-3.90946200	-5.04558000
H	-12.81878200	-2.48574300	-1.00034800
H	-11.55293300	-3.84422300	-2.67171400
C	-15.20571800	2.64346100	-7.06377500
C	-15.34018700	3.30163700	-8.29922500
C	-13.90932800	2.36272600	-6.59191500
C	-14.21285600	3.65343500	-9.04701100
H	-16.33387700	3.54605200	-8.68063500
C	-12.78298400	2.70250800	-7.34831300
H	-13.77740800	1.87884000	-5.62068300
C	-12.93097400	3.35103900	-8.57724800
H	-14.33804200	4.16748500	-10.00383500
H	-11.78421300	2.46739400	-6.97016100
H	-12.05099500	3.62592600	-9.16458200
C	-18.03476300	3.13377700	-6.68835600
C	-18.87200000	2.72071100	-7.73933300
C	-18.27155800	4.38514700	-6.09117800
C	-19.90927000	3.54391300	-8.18880100
H	-18.72299200	1.75170500	-8.22005400
C	-19.30196300	5.21056300	-6.54653700
H	-17.65337900	4.71124300	-5.25120800
C	-20.12391500	4.79163100	-7.59692600
H	-20.55315900	3.20604300	-9.00520000
H	-19.47252100	6.17649700	-6.06489900
H	-20.93714800	5.43243100	-7.94739100

Cl	-16.75909900	0.17276400	-0.90197200
C	-11.25023400	1.82257500	-2.69409400
H	-10.74153000	2.50430500	-3.39198200
H	-10.77896700	1.89480400	-1.70357500
H	-11.17584800	0.79220400	-3.06151100

UB3LYP-D3/def2-SVP-SMD(THF)//UB3LYP/def2-SVP(gas)
HF= -4208.4087957
UPBEPBE-D3/def2-SVP-SMD(THF)//UB3LYP/def2-SVP(gas)
HF= -4205.3207036

^{6}C

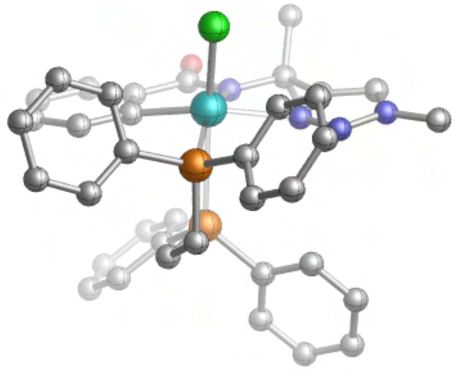

Zero-point correction= 0.681734 (Hartree/Particle)
Thermal correction to Energy= 0.728050
Thermal correction to Enthalpy= 0.728994
Thermal correction to Gibbs Free Energy= 0.595234
Sum of electronic and zero-point Energies= -4207.525316
Sum of electronic and thermal Energies= -4207.479000
Sum of electronic and thermal Enthalpies= -4207.478056
Sum of electronic and thermal Free Energies= -4207.611816

C	-18.81187400	1.43158400	-3.44192700
C	-19.27666200	2.69171000	-3.00635900
C	-20.63929200	3.01552500	-3.03442900
C	-21.56456700	2.08327600	-3.50744200
C	-21.12339700	0.82869000	-3.94421300
C	-19.75980700	0.50629100	-3.90584200
H	-20.94591800	4.00238100	-2.67916500
H	-22.62975100	2.32996000	-3.53369200
H	-21.84584800	0.09204200	-4.30938600
H	-19.45139100	-0.49102800	-4.23260600
C	-18.28930200	3.70993700	-2.49352500
O	-18.65254200	4.81924200	-2.09342700
C	-15.90792400	4.09395400	-2.01576600
C	-16.05730600	4.30868300	-0.48956400
H	-15.20712100	4.88195100	-0.08359800
H	-16.98484500	4.86308700	-0.29567600
H	-16.10781100	3.33558900	0.02100400
C	-15.80238300	5.45570600	-2.74626900
H	-16.73291200	6.01724600	-2.59919700
H	-14.95761800	6.04467700	-2.35241000
H	-15.63842500	5.30274200	-3.82440600
C	-14.63739000	3.31135000	-2.28255000
N	-14.67194900	2.10827400	-2.91544100
N	-13.47437200	1.60712100	-3.03434400
C	-13.30664100	3.56103200	-1.99573600
H	-12.80345000	4.38562200	-1.49920800
N	-12.63811800	2.47949900	-2.48085900
N	-16.99697800	3.26201900	-2.53583300
Fe	-16.77382900	1.31808200	-3.02683200
P	-16.04844200	-1.03592900	-4.35691300

P	-16.65098700	1.97901000	-5.91927300
C	-15.86212300	-0.72151600	-6.18506500
H	-15.92200700	-1.65653100	-6.76353400
H	-14.83626200	-0.34437100	-6.32492100
C	-16.88744000	0.29580300	-6.70929100
H	-17.91391800	-0.02474400	-6.46664500
H	-16.81228800	0.37615300	-7.80558300
C	-17.35595400	-2.34066200	-4.22859800
C	-17.86180600	-2.61733600	-2.94436900
C	-17.86330100	-3.05539600	-5.32625500
C	-18.83681100	-3.60133700	-2.76681000
H	-17.50496200	-2.04313100	-2.08511300
C	-18.84676100	-4.03321900	-5.14431100
H	-17.50113900	-2.86120300	-6.33767200
C	-19.33255100	-4.31192200	-3.86439600
H	-19.21981400	-3.80314200	-1.76331000
H	-19.23272600	-4.57870000	-6.00955900
H	-20.10114800	-5.07626900	-3.72364100
C	-14.50846800	-1.96446300	-3.92159400
C	-13.89439800	-2.85974100	-4.81514700
C	-13.95018900	-1.78141100	-2.64563300
C	-12.74006600	-3.55289900	-4.44324500
H	-14.31726400	-3.03157500	-5.80779500
C	-12.80066200	-2.48644400	-2.27361200
H	-14.42479200	-1.09276300	-1.94266500
C	-12.19129200	-3.36845100	-3.16979400
H	-12.27243200	-4.24554900	-5.14804700
H	-12.38126500	-2.34350400	-1.27410700
H	-11.29209300	-3.91693000	-2.87664200
C	-15.19752800	2.61684900	-6.89043000
C	-15.33784000	3.37458200	-8.06740700
C	-13.89940500	2.32179800	-6.43318100
C	-14.21321300	3.81129400	-8.77299000
H	-16.33331100	3.62879400	-8.43674900
C	-12.77606400	2.74943200	-7.14855800
H	-13.75858100	1.76369700	-5.50519600
C	-12.92865100	3.49689300	-8.31915400
H	-14.34332100	4.40080900	-9.68450300
H	-11.77577900	2.50275400	-6.78212400
H	-12.05060200	3.83826500	-8.87359700
C	-18.04471900	2.98117200	-6.58505500
C	-18.91060900	2.54867400	-7.60348700
C	-18.25064200	4.25204800	-6.01876700
C	-19.94792900	3.37284400	-8.05180500
H	-18.78698900	1.56520300	-8.06026000
C	-19.28155300	5.07656000	-6.47258600
H	-17.60749300	4.59595600	-5.20601700
C	-20.13368000	4.63886200	-7.49105000
H	-20.61473100	3.01965600	-8.84282000
H	-19.42883500	6.05682800	-6.01303100
H	-20.94739300	5.27972900	-7.84010700

Cl	-16.55352900	0.20883900	-1.01583000
C	-11.21095700	2.21044600	-2.46312900
H	-10.82089000	2.31006400	-1.44017900
H	-11.06516300	1.18137500	-2.81262900
H	-10.67687700	2.90646600	-3.12735200

UB3LYP-D3/def2-SVP-SMD(THF)//UB3LYP/def2-SVP(gas)
HF= -4208.3905090
UPBEPBE-D3/def2-SVP-SMD(THF)//UB3LYP/def2-SVP(gas)
HF= -4205.2917538

^{2}C'''

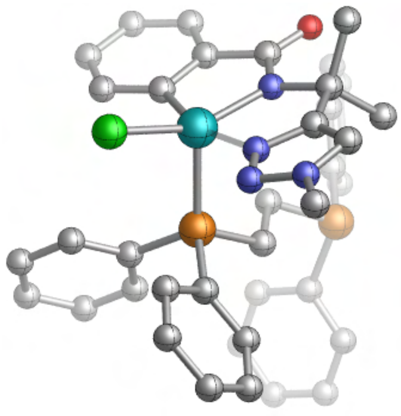

Zero-point correction= 0.683272 (Hartree/Particle)
Thermal correction to Energy= 0.729120
Thermal correction to Enthalpy= 0.730065
Thermal correction to Gibbs Free Energy= 0.598267
Sum of electronic and zero-point Energies= -4207.523308
Sum of electronic and thermal Energies= -4207.477459
Sum of electronic and thermal Enthalpies= -4207.476515
Sum of electronic and thermal Free Energies= -4207.608313

C	-19.09746200	0.70692500	-2.88171800
C	-19.51634800	2.02720700	-3.13529600
C	-20.77377500	2.31543200	-3.67418400
C	-21.64736100	1.26596600	-3.97023500
C	-21.25510700	-0.05178300	-3.70862100
C	-19.99278900	-0.33184800	-3.16438800
H	-21.05007900	3.35978700	-3.83970300
H	-22.63457800	1.47220100	-4.39231700
H	-21.94208300	-0.87595200	-3.92374500
H	-19.72382000	-1.36642700	-2.94612600
C	-18.56178500	3.10827700	-2.74101300
O	-18.77882600	4.30714000	-2.92913400
C	-16.41753200	3.48895800	-1.57611600
C	-17.02898200	4.36935500	-0.45797000
H	-16.25064700	4.98492800	0.02186700
H	-17.79142600	5.03112000	-0.88732500
H	-17.49838700	3.73680400	0.31067500
C	-15.73688300	4.38310900	-2.63795300
H	-15.27962700	3.77607700	-3.43349100
H	-16.48359200	5.04818600	-3.08922200
H	-14.94493700	4.99728400	-2.17887300
C	-15.40167500	2.56109900	-0.95485300
N	-15.57977000	1.21329400	-1.05191600
N	-14.64074800	0.57068100	-0.41863000
C	-14.25436100	2.75009900	-0.20891900
H	-13.73034300	3.64062200	0.12587000
N	-13.83072600	1.48952700	0.09054400
N	-17.43371200	2.58532700	-2.15351200
Fe	-17.32362400	0.66631400	-2.05212200

P	-16.13992400	-0.17990400	-3.92476400
P	-16.28852100	3.25714700	-6.92874600
C	-15.73713400	1.14326600	-5.16399000
H	-15.15467600	0.67992000	-5.97578300
H	-15.04807500	1.82343500	-4.63762500
C	-16.91591400	1.93520200	-5.73809200
H	-17.44308700	2.45927500	-4.93174900
H	-17.65267900	1.27932500	-6.22611700
C	-16.92204200	-1.54623300	-4.89418200
C	-17.52821600	-2.61090200	-4.20482200
C	-16.87095900	-1.58608200	-6.29788500
C	-18.06351100	-3.69305800	-4.90753900
H	-17.58779200	-2.58838700	-3.11449600
C	-17.41656300	-2.66747800	-6.99603800
H	-16.40598200	-0.78213300	-6.86972800
C	-18.01223700	-3.72453400	-6.30400100
H	-18.53025000	-4.51337900	-4.35632800
H	-17.37011800	-2.67936400	-8.08797900
H	-18.43690900	-4.56986100	-6.85160100
C	-14.47994200	-0.90333300	-3.52387300
C	-13.31911700	-0.60049300	-4.25682200
C	-14.39257300	-1.82551200	-2.46584500
C	-12.10250700	-1.21704600	-3.94761000
H	-13.34397400	0.11204900	-5.08201200
C	-13.17798800	-2.44833800	-2.16997300
H	-15.27280500	-2.03831100	-1.85493500
C	-12.02954400	-2.14803100	-2.90857100
H	-11.21094600	-0.97149900	-4.53062700
H	-13.13098400	-3.16824000	-1.34873300
H	-11.07993800	-2.63679800	-2.67485600
C	-15.85322900	2.20261800	-8.39717000
C	-16.74695400	1.30399100	-9.01081700
C	-14.54441200	2.29115100	-8.89977800
C	-16.33928900	0.51711900	-10.09070900
H	-17.77625900	1.22719500	-8.65052100
C	-14.13287300	1.50251100	-9.98104000
H	-13.84151300	2.98987700	-8.43718500
C	-15.02959900	0.61343800	-10.57720900
H	-17.04776400	-0.17145000	-10.55928000
H	-13.11025400	1.58641200	-10.35835600
H	-14.71257400	-0.00247600	-11.42288400
C	-17.86892400	4.07173300	-7.45009200
C	-18.74916300	4.56666000	-6.46790900
C	-18.14653900	4.35393000	-8.80013600
C	-19.88509400	5.29422000	-6.83281000
H	-18.56596800	4.40074900	-5.40323600
C	-19.28135600	5.08715500	-9.15996700
H	-17.47368800	3.99905100	-9.58380700
C	-20.15825000	5.55645200	-8.17854000
H	-20.55526100	5.66074100	-6.05096100
H	-19.47923000	5.29041100	-10.21605900

H	-21.04674800	6.12747100	-8.46038800
Cl	-17.58500300	-1.24864600	-0.90562100
C	-12.68131100	1.08389600	0.88114700
H	-11.77230700	1.57329600	0.50371200
H	-12.82819500	1.34711800	1.93934100
H	-12.58219700	-0.00339000	0.78314000

UB3LYP-D3/def2-SVP-SMD(THF)//UB3LYP/def2-SVP(gas)
HF= -4208.4049294
UPBEPBE-D3/def2-SVP-SMD(THF)//UB3LYP/def2-SVP(gas)
HF= -4205.3100932

^{4}C'''

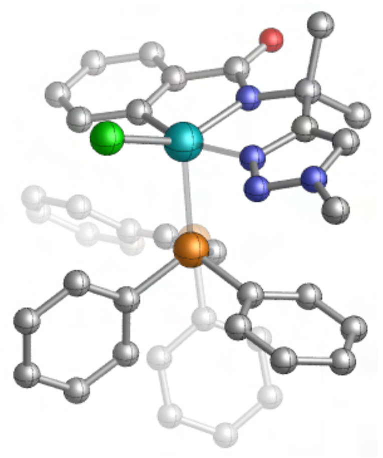

Zero-point correction= 0.682085 (Hartree/Particle)
Thermal correction to Energy= 0.728502
Thermal correction to Enthalpy= 0.729446
Thermal correction to Gibbs Free Energy= 0.593051
Sum of electronic and zero-point Energies= -4207.547646
Sum of electronic and thermal Energies= -4207.501229
Sum of electronic and thermal Enthalpies= -4207.500285
Sum of electronic and thermal Free Energies= -4207.636680

C	-18.81203600	1.67376100	-2.64023000
C	-18.71694400	3.07300300	-2.59035200
C	-19.71401600	3.88707400	-3.13933600
C	-20.83211100	3.29524200	-3.73150000
C	-20.94555900	1.89830700	-3.76097300
C	-19.94084100	1.08345500	-3.21810600
H	-19.59439500	4.97204800	-3.07879600
H	-21.62116200	3.91733000	-4.16175300
H	-21.83200100	1.43409300	-4.20393100
H	-20.05523200	-0.00247500	-3.22922500
C	-17.51881500	3.63915900	-1.89335900
O	-17.32672000	4.84315700	-1.74460000
C	-15.50970600	2.94994500	-0.63485100
C	-15.90065000	3.65469200	0.68646700
H	-15.01194300	3.83432800	1.31351200
H	-16.37642400	4.61703600	0.45840700
H	-16.60963700	3.03279300	1.25386300
C	-14.49416500	3.81116500	-1.42239900
H	-14.95922800	4.76856900	-1.68813300
H	-13.59225300	4.00308900	-0.81817600
H	-14.18747800	3.29352200	-2.34396400
C	-14.88641900	1.61095500	-0.31534800
N	-15.48227200	0.47165700	-0.76422100
N	-14.82444500	-0.58777700	-0.38921200
C	-13.76902400	1.20774300	0.39076500
H	-12.99785700	1.75396500	0.92596100
N	-13.78602600	-0.15307200	0.31174700
N	-16.70190800	2.62804900	-1.44359800

Fe	-17.26282000	0.79111200	-1.78569000
P	-16.15293200	0.13291000	-4.06995600
P	-17.22969500	3.20329200	-7.27941900
C	-16.09872900	1.51060000	-5.33381400
H	-15.31352700	1.26842000	-6.06802500
H	-15.76247500	2.40601100	-4.78582400
C	-17.42759900	1.80425600	-6.03717200
H	-18.17972300	2.13065500	-5.30384500
H	-17.81788000	0.90348300	-6.53663100
C	-16.80685600	-1.32099100	-5.00076000
C	-17.74484700	-2.16419300	-4.38402000
C	-16.37618800	-1.61250500	-6.30884000
C	-18.24443800	-3.27991200	-5.06733100
H	-18.07641600	-1.95668900	-3.36312600
C	-16.88231300	-2.72200500	-6.98755500
H	-15.64045000	-0.97519700	-6.80542100
C	-17.81869200	-3.55826600	-6.36740700
H	-18.97186400	-3.93055200	-4.57528800
H	-16.54289200	-2.93583300	-8.00438100
H	-18.21318200	-4.42734600	-6.90052800
C	-14.35991600	-0.29496200	-3.89386600
C	-13.34667600	0.66887800	-4.04031800
C	-13.99402600	-1.60202800	-3.52491200
C	-12.00395700	0.33044900	-3.83988800
H	-13.59210800	1.69503900	-4.32050600
C	-12.65301000	-1.93944200	-3.32992800
H	-14.76355900	-2.36585900	-3.39330200
C	-11.65145500	-0.97499400	-3.48798000
H	-11.23112100	1.09274200	-3.96930200
H	-12.38947000	-2.96529500	-3.05874100
H	-10.60104900	-1.24199200	-3.34475700
C	-16.16110000	2.36513400	-8.55451400
C	-16.58591800	1.27057400	-9.33011000
C	-14.85372800	2.84714500	-8.73414700
C	-15.72311200	0.67269100	-10.25173100
H	-17.60426600	0.88860600	-9.21947900
C	-13.98633800	2.24705900	-9.65470600
H	-14.51538200	3.70710800	-8.14886900
C	-14.41996700	1.15861400	-10.41491600
H	-16.06979700	-0.17405200	-10.85056800
H	-12.97267300	2.63650600	-9.78170000
H	-13.74730300	0.69132900	-11.13900200
C	-18.87240000	3.19638800	-8.13542800
C	-20.02801300	2.59106800	-7.61350400
C	-18.98585300	3.92890000	-9.33367100
C	-21.25522700	2.70395700	-8.27842400
H	-19.98986000	2.03167000	-6.67753800
C	-20.20728000	4.03337300	-9.99964200
H	-18.10365500	4.42186500	-9.75277100
C	-21.34995500	3.41988000	-9.47307300
H	-22.14185800	2.22469400	-7.85456900

H	-20.26948900	4.60131500	-10.93184300
H	-22.30887600	3.50474100	-9.99078600
Cl	-18.36638200	-0.97478200	-0.98749700
C	-12.81604700	-1.10151300	0.83143300
H	-13.29256200	-2.08874100	0.85224500
H	-11.92851700	-1.13723800	0.18185500
H	-12.52004000	-0.81125700	1.84872600

UB3LYP-D3/def2-SVP-SMD(THF)//UB3LYP/def2-SVP(gas)
HF= -4208.4049294
UPBEPBE-D3/def2-SVP-SMD(THF)//UB3LYP/def2-SVP(gas)
HF= -4205.3167641

^{6}C'''

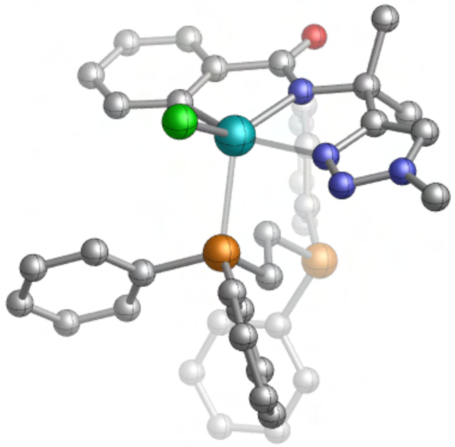

Zero-point correction= 0.681461 (Hartree/Particle)
Thermal correction to Energy= 0.728165
Thermal correction to Enthalpy= 0.729109
Thermal correction to Gibbs Free Energy= 0.591631
Sum of electronic and zero-point Energies= -4207.529027
Sum of electronic and thermal Energies= -4207.482323
Sum of electronic and thermal Enthalpies= -4207.481379
Sum of electronic and thermal Free Energies= -4207.618857

C	-18.86788000	0.42204300	-2.95069800
C	-19.32159200	1.75479600	-2.93470300
C	-20.60744500	2.09664200	-3.37441100
C	-21.46428700	1.09594100	-3.83657300
C	-21.03590800	-0.23797700	-3.84654500
C	-19.74996000	-0.57132700	-3.40231300
H	-20.91647000	3.14380400	-3.33580600
H	-22.47027800	1.35058500	-4.18158600
H	-21.71141300	-1.02421600	-4.19759200
H	-19.44519400	-1.62118100	-3.40785200
C	-18.40644600	2.81808000	-2.39445800
O	-18.72366600	4.01159800	-2.41522500
C	-16.27066000	3.24454000	-1.27442200
C	-16.87860900	3.89454500	-0.00771500
H	-16.13882800	4.53615200	0.49857700
H	-17.74299300	4.50792400	-0.29130800
H	-17.20825500	3.11710200	0.69828400
C	-15.77052600	4.34091000	-2.24923400
H	-16.61185500	4.97436900	-2.55393800
H	-15.00526800	4.96823400	-1.76365900
H	-15.32068200	3.88806400	-3.14645900
C	-15.08669300	2.39653300	-0.86735900
N	-15.02840000	1.08106600	-1.20305100
N	-13.93654400	0.53144800	-0.74964200
C	-13.93200800	2.67154500	-0.15656700
H	-13.54919100	3.57491800	0.30931000
N	-13.26322600	1.48560900	-0.11920500
N	-17.22260100	2.31818600	-1.90872700

Fe	-16.97543200	0.30340800	-2.08743200
P	-15.69385800	-0.51445000	-4.22241800
P	-15.96808700	3.21068800	-6.82146200
C	-15.32991300	0.86386200	-5.41098700
H	-14.92636900	0.44390700	-6.34631900
H	-14.50541100	1.42787100	-4.94345800
C	-16.51145800	1.79842000	-5.69688600
H	-16.86300600	2.24516500	-4.75566200
H	-17.36593100	1.25510600	-6.12961100
C	-16.62247800	-1.79964100	-5.16274400
C	-17.16011900	-2.87424300	-4.43116400
C	-16.78243300	-1.77229900	-6.55685500
C	-17.82756200	-3.90992300	-5.08814100
H	-17.06897000	-2.89282900	-3.34140300
C	-17.46196500	-2.80683800	-7.20835200
H	-16.38347200	-0.94952500	-7.15303400
C	-17.98095000	-3.87862100	-6.47808600
H	-18.23821200	-4.74039900	-4.50836600
H	-17.58237300	-2.77120500	-8.29411700
H	-18.50927000	-4.68676300	-6.99037200
C	-14.05373700	-1.32601200	-3.95641900
C	-13.03926000	-1.30823200	-4.93014000
C	-13.83824100	-2.02543300	-2.75714900
C	-11.83366000	-1.97805300	-4.70784500
H	-13.18087300	-0.77981900	-5.87503700
C	-12.63450500	-2.70440800	-2.54518200
H	-14.60766900	-2.03307700	-1.98186100
C	-11.63019900	-2.68127100	-3.51612500
H	-11.05361600	-1.95610600	-5.47336500
H	-12.48306600	-3.24923100	-1.60979300
H	-10.68892600	-3.21094800	-3.34690900
C	-15.80262800	2.29537000	-8.43193800
C	-16.84495400	1.55172600	-9.01765500
C	-14.55659700	2.32653800	-9.07959100
C	-16.64155600	0.85723100	-10.21247800
H	-17.82940800	1.52595300	-8.54273200
C	-14.35004300	1.63020300	-10.27672300
H	-13.74046700	2.90682500	-8.63973100
C	-15.39202900	0.89369100	-10.84406000
H	-17.46296200	0.28871300	-10.65707400
H	-13.37363500	1.66754400	-10.76705800
H	-15.23524800	0.35111700	-11.78006200
C	-17.54687600	4.15208800	-7.04713900
C	-18.34264300	4.48071100	-5.93390000
C	-17.89376800	4.69451000	-8.29877000
C	-19.46119900	5.30690100	-6.07353100
H	-18.11016600	4.10527800	-4.93602100
C	-19.01199400	5.52179400	-8.43516600
H	-17.28826600	4.46919000	-9.17990200
C	-19.80242000	5.82963200	-7.32382700
H	-20.05951500	5.53800000	-5.18876500

H	-19.26562900	5.92739600	-9.41851200
H	-20.67727700	6.47604200	-7.43199600
Cl	-17.02234900	-1.51461100	-0.75194100
C	-11.99296300	1.18022900	0.51627500
H	-11.73613900	0.14686700	0.25545200
H	-11.21101700	1.85995300	0.14840500
H	-12.07689800	1.27479600	1.60909200

UB3LYP-D3/def2-SVP-SMD(THF)//UB3LYP/def2-SVP(gas)
HF= -4208.3860408
UPBEPBE-D3/def2-SVP-SMD(THF)//UB3LYP/def2-SVP(gas)
HF= -4205.2848441

Figure S31

^{2}C'

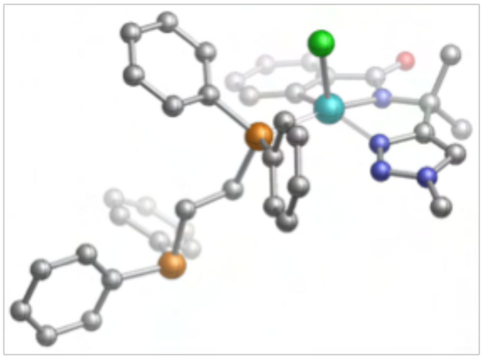

Zero-point correction= 0.683089 (Hartree/Particle)
Thermal correction to Energy= 0.729024
Thermal correction to Enthalpy= 0.729968
Thermal correction to Gibbs Free Energy= 0.596800
Sum of electronic and zero-point Energies= -4207.509702
Sum of electronic and thermal Energies= -4207.463768
Sum of electronic and thermal Enthalpies= -4207.462824
Sum of electronic and thermal Free Energies= -4207.595992

C	-19.81423200	-2.92673200	-3.31809900
C	-20.63708000	-1.83471500	-2.97039100
C	-22.02234500	-1.94990500	-2.83376100
C	-22.63290500	-3.18338200	-3.07411000
C	-21.84671000	-4.27806700	-3.44694500
C	-20.45345000	-4.15228100	-3.56087300
H	-22.59653300	-1.06339700	-2.55179600
H	-23.71676300	-3.29195400	-2.97894600
H	-22.31599300	-5.24527300	-3.64908100
H	-19.89002100	-5.04522700	-3.83704600
C	-19.95067100	-0.51843800	-2.80043000
O	-20.51558000	0.50873100	-2.43919400
C	-17.73647600	0.54804200	-3.09742300
C	-17.51469500	1.02069400	-1.64047600
H	-16.79994200	1.85922700	-1.60171400
H	-18.47436900	1.35224800	-1.22187800
H	-17.12668900	0.19000000	-1.03271100
C	-18.26631500	1.70439200	-3.97709700
H	-19.22268700	2.06288000	-3.57902000
H	-17.54546500	2.53831700	-3.98998900
H	-18.42000600	1.35983000	-5.01140700
C	-16.43288300	0.05705400	-3.67713900
N	-16.28995100	-1.26480900	-3.97946300
N	-15.11073800	-1.50510200	-4.47983800
C	-15.23973300	0.66414500	-4.01622900
H	-14.89054600	1.69053000	-3.95265300
N	-14.46717500	-0.34777400	-4.50426900
N	-18.61949700	-0.63230900	-3.13767800
Fe	-17.90318400	-2.36630200	-3.27800200
Cl	-16.92184900	-2.98507800	-1.26077200

C	-13.11352600	-0.28293100	-5.02941400
H	-12.45780900	0.22945400	-4.31141700
H	-12.76739000	-1.31323900	-5.17473000
H	-13.09712400	0.25456400	-5.98942400
P	-16.93812500	-4.59968000	-3.94079800
C	-17.56196200	-5.09422700	-5.63814500
H	-17.17334000	-4.31428600	-6.31564200
H	-18.64738500	-4.91733800	-5.59493300
C	-17.27947600	-6.49482800	-6.18856200
H	-17.76818200	-7.25690900	-5.56245200
H	-16.19886800	-6.70952100	-6.18814700
P	-17.84067200	-6.64765300	-7.97771000
C	-19.68592100	-6.66458400	-7.75195600
C	-20.40339800	-5.52365300	-8.14955300
C	-20.39202400	-7.74302200	-7.18935900
C	-21.78964000	-5.45206100	-7.97475600
H	-19.86981600	-4.68322600	-8.60264300
C	-21.77717800	-7.67571300	-7.02202300
H	-19.85638500	-8.64636600	-6.88605300
C	-22.47877300	-6.52889400	-7.41189900
H	-22.33125600	-4.55409700	-8.28291900
H	-22.31293800	-8.52312000	-6.58576000
H	-23.56259600	-6.47762200	-7.27948600
C	-17.49129800	-8.42967700	-8.34837800
C	-17.79917300	-8.87764900	-9.64800600
C	-16.88750700	-9.33546300	-7.45977600
C	-17.53290600	-10.19020600	-10.03933500
H	-18.25472500	-8.18544600	-10.36263000
C	-16.60790600	-10.64897500	-7.85620800
H	-16.63030900	-9.02951800	-6.44416300
C	-16.93215600	-11.08193600	-9.14332800
H	-17.78726500	-10.51712400	-11.05108700
H	-16.13703300	-11.33721000	-7.14903600
H	-16.71526000	-12.10832000	-9.45016500
C	-17.25557400	-6.06271300	-2.85031900
C	-18.21585800	-6.00823000	-1.82730100
C	-16.52786600	-7.25407200	-3.03295200
C	-18.46435200	-7.12958500	-1.02955500
H	-18.75040900	-5.07997900	-1.63272700
C	-16.77701000	-8.37167600	-2.23289200
H	-15.74429700	-7.30741700	-3.79087100
C	-17.75184500	-8.31360700	-1.23200100
H	-19.21357700	-7.06739300	-0.23640000
H	-16.20060700	-9.28761200	-2.38737100
H	-17.94501600	-9.18647900	-0.60280900
C	-15.09765000	-4.63626900	-4.10595100
C	-14.44984200	-4.63494400	-5.35181100
C	-14.31341500	-4.60779100	-2.93791100
C	-13.05339000	-4.62406100	-5.43087900
H	-15.02305400	-4.63406700	-6.27997100
C	-12.91981000	-4.58982700	-3.01943300

H	-14.80122800	-4.57766400	-1.96214000
C	-12.28307900	-4.60223400	-4.26522600
H	-12.56898400	-4.63762500	-6.41106200
H	-12.32759600	-4.56853700	-2.10075800
H	-11.19138300	-4.60076600	-4.32632600

UB3LYP-D3/def2-SVP-SMD(THF)//UB3LYP/def2-SVP(gas)
HF= -4208.3493296
UPBEPBE-D3/def2-SVP-SMD(THF)//UB3LYP/def2-SVP(gas)
HF= -4205.2833791

^{4}C'

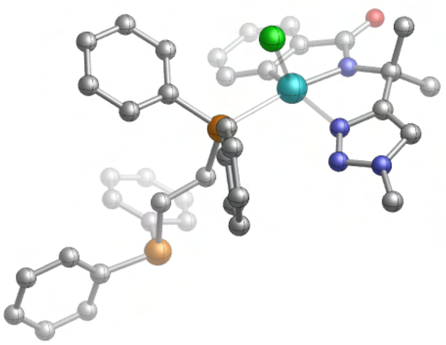

Zero-point correction= 0.682801 (Hartree/Particle)
Thermal correction to Energy= 0.728870
Thermal correction to Enthalpy= 0.729814
Thermal correction to Gibbs Free Energy= 0.594710
Sum of electronic and zero-point Energies= -4207.534955
Sum of electronic and thermal Energies= -4207.488885
Sum of electronic and thermal Enthalpies= -4207.487941
Sum of electronic and thermal Free Energies= -4207.623045

C	-19.84165600	-2.86736900	-3.35491800
C	-20.63487400	-1.78347200	-2.93200900
C	-22.02024000	-1.88545100	-2.78787900
C	-22.65352400	-3.09273400	-3.09424800
C	-21.89070600	-4.17862400	-3.53516200
C	-20.49633800	-4.06998700	-3.65730200
H	-22.57506100	-1.00812600	-2.44469700
H	-23.73821700	-3.18849800	-2.99472300
H	-22.37810200	-5.12530500	-3.78563300
H	-19.94796900	-4.95504000	-3.98426500
C	-19.91941800	-0.49314000	-2.68427800
O	-20.46474800	0.51834700	-2.25097600
C	-17.69725600	0.55167000	-2.98958300
C	-17.39428700	0.94888200	-1.52537400
H	-16.66975300	1.77899900	-1.48104400
H	-18.32883700	1.26766900	-1.04390300
H	-16.98692100	0.08548000	-0.97938500
C	-18.25206600	1.75958000	-3.78152000
H	-19.18149100	2.10961200	-3.31778000
H	-17.51877400	2.58273500	-3.78879300
H	-18.46351500	1.47104300	-4.82283400
C	-16.43356100	0.07881600	-3.66825100
N	-16.32565800	-1.22198500	-4.05876000
N	-15.18221600	-1.44400600	-4.64345200
C	-15.25392900	0.69347700	-4.04075600
H	-14.88733000	1.71036100	-3.93596100
N	-14.52498900	-0.29387000	-4.63528600
N	-18.60119600	-0.61189900	-3.04179400
Fe	-17.89338800	-2.37425100	-3.27493600
Cl	-16.82487900	-2.97203800	-1.29603200
C	-13.20542100	-0.20841100	-5.23801800
H	-12.50859300	0.28072800	-4.54308900

H	-12.86748200	-1.23212200	-5.43834400
H	-13.24560200	0.36121100	-6.17870900
P	-16.95440000	-4.61295700	-3.95692500
C	-17.56142400	-5.11688800	-5.65778200
H	-17.17897400	-4.33422800	-6.33548400
H	-18.64953600	-4.95573300	-5.62434700
C	-17.25428000	-6.51514100	-6.20071800
H	-17.73368300	-7.28238000	-5.57371700
H	-16.17002600	-6.71084700	-6.19433600
P	-17.80768100	-6.68383900	-7.99108400
C	-19.65323500	-6.71732400	-7.76880800
C	-20.38195800	-5.58889400	-8.18133100
C	-20.34878900	-7.79591000	-7.19336400
C	-21.76913400	-5.52945800	-8.00886300
H	-19.85655000	-4.74871500	-8.64432900
C	-21.73469800	-7.74076100	-7.02800700
H	-19.80415900	-8.68983100	-6.87830700
C	-22.44767700	-6.60618200	-7.43301500
H	-22.31970300	-4.64123000	-8.32912700
H	-22.26217500	-8.58802600	-6.58153500
H	-23.53216200	-6.56462800	-7.30256900
C	-17.44064500	-8.46455100	-8.34994900
C	-17.76009200	-8.92931400	-9.64089800
C	-16.81113000	-9.35283400	-7.46162200
C	-17.48001100	-10.24149100	-10.02358800
H	-18.23603900	-8.25097400	-10.35551000
C	-16.51787700	-10.66590000	-7.84962700
H	-16.54377000	-9.03341500	-6.45282800
C	-16.85375600	-11.11583800	-9.12786400
H	-17.74378800	-10.58174200	-11.02850000
H	-16.02701600	-11.34034400	-7.14277300
H	-16.62621900	-12.14185500	-9.42810500
C	-17.30751600	-6.05512100	-2.85569100
C	-18.28785200	-5.96824100	-1.85433400
C	-16.59523600	-7.25989800	-3.00622600
C	-18.57138300	-7.07278500	-1.04525200
H	-18.81204300	-5.02864000	-1.68612000
C	-16.87910600	-8.36037200	-2.19403600
H	-15.79790600	-7.33701100	-3.74766700
C	-17.87351600	-8.27073300	-1.21491000
H	-19.33584100	-6.98666500	-0.26913600
H	-16.31497800	-9.28791500	-2.32208600
H	-18.09369400	-9.13046800	-0.57661400
C	-15.11304100	-4.65414900	-4.10864700
C	-14.46326100	-4.61256900	-5.35340000
C	-14.32984300	-4.67728800	-2.93983100
C	-13.06689300	-4.61539900	-5.43087600
H	-15.03502500	-4.57020700	-6.28134800
C	-12.93581100	-4.67290900	-3.02001700
H	-14.81743200	-4.67771800	-1.96388800
C	-12.29792900	-4.64662000	-4.26455900

H	-12.58165500	-4.59769400	-6.41055400
H	-12.34483500	-4.69190300	-2.10059900
H	-11.20626200	-4.65442800	-4.32459300

UB3LYP-D3/def2-SVP-SMD(THF)//UB3LYP/def2-SVP(gas)
HF= -4208.39294102
UPBEPBE-D3/def2-SVP-SMD(THF)//UB3LYP/def2-SVP(gas)
HF= -4205.3048230

^{6}C'

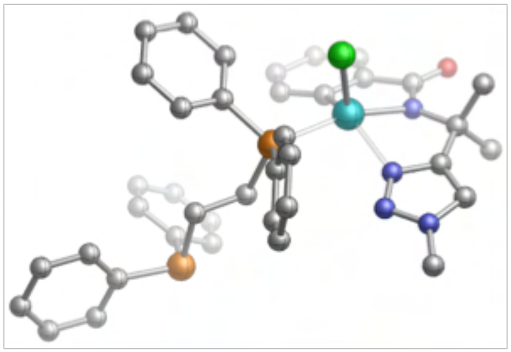

Zero-point correction= 0.681523 (Hartree/Particle)
Thermal correction to Energy= 0.728021
Thermal correction to Enthalpy= 0.728965
Thermal correction to Gibbs Free Energy= 0.591596
Sum of electronic and zero-point Energies= -4207.514675
Sum of electronic and thermal Energies= -4207.468177
Sum of electronic and thermal Enthalpies= -4207.467233
Sum of electronic and thermal Free Energies= -4207.604601

C	-19.99063800	-2.92218800	-3.22777300
C	-20.68571600	-1.76175300	-2.81452800
C	-22.07650700	-1.76174200	-2.65638800
C	-22.80711800	-2.91868000	-2.93813800
C	-22.14248600	-4.06947500	-3.37529500
C	-20.74657400	-4.06995200	-3.50996400
H	-22.56142100	-0.83982900	-2.32573400
H	-23.89487700	-2.92293400	-2.82528200
H	-22.71015100	-4.97475600	-3.61049700
H	-20.26311200	-4.99554600	-3.83748400
C	-19.91714800	-0.47903000	-2.59897800
O	-20.45860900	0.54800900	-2.19207000
C	-17.70056900	0.53562100	-3.03550800
C	-17.25256200	0.99406600	-1.62756500
H	-16.51642500	1.81312700	-1.68595500
H	-18.13504200	1.34888400	-1.07678400
H	-16.80884900	0.15069500	-1.07803000
C	-18.30736900	1.72133300	-3.82345800
H	-19.18413300	2.10792000	-3.29137700
H	-17.56421500	2.52767300	-3.93665500
H	-18.62250300	1.39404000	-4.82641100
C	-16.51399900	0.00845500	-3.82195500
N	-16.45572300	-1.30557200	-4.18319300
N	-15.38178000	-1.55327200	-4.87946100
C	-15.37662600	0.59976800	-4.33904100
H	-14.99707500	1.61686400	-4.30731600
N	-14.72012000	-0.40916800	-4.97802600
N	-18.60032700	-0.61296900	-2.94489200
Fe	-17.95508100	-2.50525300	-3.09196400
Cl	-16.88362900	-3.11527500	-1.11839300
C	-13.47782500	-0.34978600	-5.73021900

H	-12.73267600	0.23091300	-5.16940900
H	-13.11941100	-1.37763400	-5.86223000
H	-13.64136400	0.11589400	-6.71391700
P	-16.86950700	-4.65383200	-3.84279900
C	-17.46427700	-5.09186300	-5.56510500
H	-17.07496200	-4.28870900	-6.21484600
H	-18.55309900	-4.92478200	-5.52307400
C	-17.16598400	-6.47461400	-6.15072700
H	-17.65198300	-7.25690300	-5.54781900
H	-16.08313700	-6.67833700	-6.14632800
P	-17.71352100	-6.58563600	-7.94736200
C	-19.55976700	-6.62375000	-7.73330800
C	-20.28361300	-5.47636200	-8.09971800
C	-20.26070700	-7.72389200	-7.20744300
C	-21.67115500	-5.41986300	-7.92959300
H	-19.75405100	-4.61873600	-8.52443900
C	-21.64708400	-7.67149200	-7.04516400
H	-19.71988800	-8.63207900	-6.92880000
C	-22.35507700	-6.51829700	-7.40328900
H	-22.21774900	-4.51666400	-8.21256400
H	-22.17875700	-8.53542600	-6.63728100
H	-23.43983200	-6.47871700	-7.27461400
C	-17.34558100	-8.35409800	-8.36181300
C	-17.64288400	-8.77141800	-9.67405400
C	-16.73699200	-9.27648200	-7.49380600
C	-17.36170900	-10.07076800	-10.09768100
H	-18.10200500	-8.06559500	-10.37289800
C	-16.44239400	-10.57650600	-7.92253000
H	-16.48767900	-8.99430600	-6.46938200
C	-16.75625100	-10.97931100	-9.22195500
H	-17.60810400	-10.37402300	-11.11872600
H	-15.96798600	-11.27807300	-7.23099600
H	-16.52767400	-11.99525000	-9.55402400
C	-17.27800100	-6.13949400	-2.81899400
C	-18.34307400	-6.10077200	-1.90471900
C	-16.54139600	-7.33147600	-2.95211000
C	-18.67975200	-7.23619700	-1.16115000
H	-18.89989200	-5.17686900	-1.75159100
C	-16.87710600	-8.46237800	-2.20397900
H	-15.68775100	-7.37524900	-3.63117600
C	-17.95160700	-8.41867800	-1.30988500
H	-19.51050700	-7.18649000	-0.45296900
H	-16.29227000	-9.37909300	-2.31640700
H	-18.21261700	-9.30275400	-0.72230900
C	-15.02627100	-4.71917400	-3.94489400
C	-14.33783300	-4.66742600	-5.16883400
C	-14.27925900	-4.76202500	-2.75273200
C	-12.93982000	-4.68224100	-5.20236300
H	-14.88254900	-4.60741200	-6.11184600
C	-12.88306900	-4.76984000	-2.78959000
H	-14.79569700	-4.77498600	-1.79147700

C	-12.20710400	-4.73455900	-4.01326400
H	-12.42363200	-4.65868200	-6.16610200
H	-12.32103400	-4.80466600	-1.85271700
H	-11.11433600	-4.74988400	-4.03985100

UB3LYP-D3/def2-SVP-SMD(THF)//UB3LYP/def2-SVP(gas)
HF= -4208.3698350
UPBEPBE-D3/def2-SVP-SMD(THF)//UB3LYP/def2-SVP(gas)
HF= -4205.2715992

[2]C''

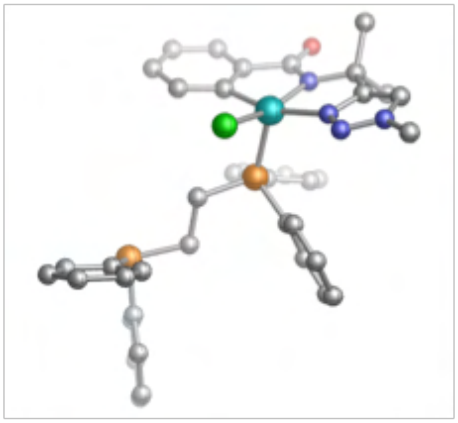

Zero-point correction= 0.683372 (Hartree/Particle)
Thermal correction to Energy= 0.729090
Thermal correction to Enthalpy= 0.730035
Thermal correction to Gibbs Free Energy= 0.597220
Sum of electronic and zero-point Energies= -4207.525225
Sum of electronic and thermal Energies= -4207.479507
Sum of electronic and thermal Enthalpies= -4207.478563
Sum of electronic and thermal Free Energies= -4207.611377

C	-17.99026800	1.10726800	-3.57522300
C	-18.82811900	2.11081300	-3.04835500
C	-20.19057800	1.89639000	-2.81796300
C	-20.74441100	0.64623900	-3.10486200
C	-19.92168600	-0.37697100	-3.59381900
C	-18.55611100	-0.15257400	-3.82569900
H	-20.78516500	2.71030700	-2.39449700
H	-21.80733700	0.45898300	-2.92917900
H	-20.34514600	-1.36659800	-3.79159300
H	-17.92713200	-0.97025200	-4.18607700
C	-18.14852000	3.37378000	-2.63274500
O	-18.72753300	4.33117100	-2.12273900
C	-15.88574100	4.26080100	-2.21299100
C	-16.06253200	4.29961000	-0.67384700
H	-15.34024800	4.99355100	-0.21373400
H	-17.07990000	4.63285300	-0.43296100
H	-15.90642300	3.29682500	-0.24792400
C	-16.07754400	5.67911100	-2.79910400
H	-17.11116900	6.00255600	-2.62397000
H	-15.38804800	6.39431700	-2.32085500
H	-15.87938300	5.68308700	-3.88057900
C	-14.49456300	3.76877800	-2.52610700
N	-14.33682600	2.61239300	-3.23146300
N	-13.07327200	2.32559900	-3.38502200
C	-13.22300600	4.22086600	-2.23000500
H	-12.85536700	5.08682300	-1.68720500
N	-12.39312300	3.29243900	-2.78450600
N	-16.78691000	3.26167700	-2.82048000
Fe	-16.11683400	1.70357600	-3.69747000

P	-16.59338200	2.00618200	-5.90278900
C	-17.21888900	0.60102100	-8.34014300
H	-17.87946200	1.43642600	-8.62407600
H	-16.24052300	0.81209500	-8.79850400
C	-17.11711600	0.47402200	-6.81836800
H	-16.38029100	-0.28270900	-6.51007900
H	-18.07150600	0.15603500	-6.37482300
C	-15.11521300	2.55784100	-6.87506100
C	-15.19351400	3.47066600	-7.94237300
C	-13.87594700	1.95927700	-6.58126300
C	-14.05519600	3.78877700	-8.68903200
H	-16.14607300	3.93363400	-8.20380600
C	-12.74313700	2.27624900	-7.33677700
H	-13.79991100	1.23684800	-5.76553800
C	-12.82671900	3.19410700	-8.38770400
H	-14.13392900	4.50005800	-9.51531000
H	-11.78881200	1.79975700	-7.09744300
H	-11.93843600	3.44228200	-8.97470000
C	-17.90236600	3.25580900	-6.22866100
C	-19.22442500	2.86062800	-6.49349700
C	-17.61943200	4.62820500	-6.11612400
C	-20.23039000	3.81564200	-6.66562700
H	-19.48779500	1.80370400	-6.55154400
C	-18.62538500	5.58064100	-6.28778800
H	-16.60421700	4.96002000	-5.89201700
C	-19.93443300	5.17670200	-6.56646000
H	-21.25309100	3.48823100	-6.86746500
H	-18.38619300	6.64279800	-6.19400500
H	-20.72338600	5.92181500	-6.69527100
Cl	-15.17058600	-0.31459900	-4.08559100
C	-10.94050800	3.26500600	-2.78491700
H	-10.54032700	4.11531800	-3.35671600
H	-10.55991600	3.30429700	-1.75417100
H	-10.63001100	2.32505800	-3.25589200
P	-18.02380200	-0.90494300	-9.13191700
C	-17.97039700	-0.35688100	-10.90855600
C	-19.11118000	0.29742200	-11.40771100
C	-16.87954100	-0.55578500	-11.77162100
C	-19.15371400	0.76112600	-12.72551100
H	-19.97955200	0.43784600	-10.75666700
C	-16.92590800	-0.10228000	-13.09345600
H	-15.98721700	-1.07289000	-11.41143100
C	-18.06011700	0.56015300	-13.57309400
H	-20.04829300	1.27003300	-13.09445300
H	-16.06951800	-0.26822600	-13.75280200
H	-18.09433600	0.91271600	-14.60729300
C	-16.69188900	-2.19123000	-9.06955200
C	-17.08944200	-3.51684800	-9.32681200
C	-15.34362100	-1.94927400	-8.75522200
C	-16.16657900	-4.56428100	-9.29398200
H	-18.13916200	-3.72849600	-9.55135100

C	-14.42096300	-3.00001500	-8.70658100
H	-14.99605600	-0.93632500	-8.54194600
C	-14.82781400	-4.30827100	-8.98004200
H	-16.49608400	-5.58576200	-9.50167000
H	-13.37870300	-2.79188000	-8.45097000
H	-14.10580000	-5.12797500	-8.94026900

UB3LYP-D3/def2-SVP-SMD(THF)//UB3LYP/def2-SVP(gas)
HF= -4208.3891593
UPBEPBE-D3/def2-SVP-SMD(THF)//UB3LYP/def2-SVP(gas)
HF= -4205.3147076

^{4}C''

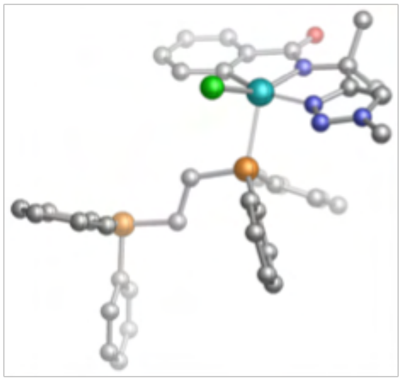

Zero-point correction=	0.682088 (Hartree/Particle)		
Thermal correction to Energy=	0.728464		
Thermal correction to Enthalpy=	0.729408		
Thermal correction to Gibbs Free Energy=	0.592135		
Sum of electronic and zero-point Energies=	-4207.548350		
Sum of electronic and thermal Energies=	-4207.501974		
Sum of electronic and thermal Enthalpies=	-4207.501030		
Sum of electronic and thermal Free Energies=	-4207.638303		
C	-18.25518400	0.81537700	-3.59067400
C	-19.19031700	1.64702500	-2.95731000
C	-20.53858700	1.28550200	-2.86692900
C	-20.95898000	0.06607000	-3.40239300
C	-20.02437900	-0.78551400	-4.00531200
C	-18.67279300	-0.41972700	-4.09820800
H	-21.22751300	1.96837500	-2.36244600
H	-22.00875500	-0.23227400	-3.33728200
H	-20.34548400	-1.75331300	-4.40199000
H	-17.95154300	-1.10920100	-4.54139600
C	-18.65845300	2.89950200	-2.33806100
O	-19.35711400	3.70295000	-1.72525300
C	-16.55436500	4.11225500	-1.92347300
C	-16.63887200	4.07043000	-0.37887200
H	-16.03104500	4.87315300	0.07033500
H	-17.68381100	4.20071300	-0.06999900
H	-16.27458700	3.10218400	-0.00291100
C	-17.03699000	5.48427700	-2.45282400
H	-18.08187200	5.63936700	-2.15739000
H	-16.41929700	6.29967900	-2.04128200
H	-16.97376500	5.51390700	-3.55063400
C	-15.12096800	3.89731400	-2.34494500
N	-14.80960600	2.85354700	-3.16332500
N	-13.53150400	2.81644300	-3.41454000
C	-13.93174200	4.55004900	-2.08097500
H	-13.68723400	5.42293400	-1.48271000
N	-12.99139700	3.84027400	-2.76645600
N	-17.30014500	2.99301800	-2.53038500
Fe	-16.43026000	1.59805400	-3.58160700
P	-16.48955600	2.08797500	-6.11748100
C	-17.30008000	0.79685600	-8.59284900

H	-17.86863100	1.69487900	-8.88487400
H	-16.29666000	0.91091200	-9.03160300
C	-17.23887800	0.67065400	-7.06961400
H	-16.63410200	-0.19650200	-6.75723800
H	-18.23764200	0.49835000	-6.63934300
C	-14.87443200	2.33415200	-6.98188100
C	-14.71548100	3.13326700	-8.12900000
C	-13.76703700	1.61732700	-6.49278100
C	-13.47532700	3.21893900	-8.76780500
H	-15.56273400	3.69174000	-8.53166600
C	-12.53140600	1.69717400	-7.14338000
H	-13.87325800	0.99292500	-5.60242300
C	-12.38049300	2.49980700	-8.27751100
H	-13.36789700	3.84405500	-9.65819700
H	-11.68185000	1.12978300	-6.75421300
H	-11.41243300	2.56422700	-8.78154300
C	-17.51292100	3.56119900	-6.52845800
C	-18.89258000	3.43379300	-6.76968000
C	-16.96218600	4.85476900	-6.45641200
C	-19.69176200	4.56548200	-6.95641700
H	-19.36017200	2.44785500	-6.80295600
C	-17.76216000	5.98368800	-6.64798100
H	-15.89686600	4.98395100	-6.25105600
C	-19.13016200	5.84322800	-6.90102200
H	-20.76181400	4.44348400	-7.14183500
H	-17.31389000	6.97927000	-6.59434100
H	-19.75645700	6.72668600	-7.04726600
Cl	-15.30084200	-0.33656700	-3.68491000
C	-11.55664500	4.06110700	-2.82746300
H	-11.34599500	5.09661500	-3.13037900
H	-11.09448500	3.86339900	-1.84881700
H	-11.14863700	3.36840300	-3.57269500
P	-18.23034800	-0.62555600	-9.40089800
C	-18.09125700	-0.09069700	-11.17675100
C	-19.15715000	0.66388600	-11.69948900
C	-17.00586700	-0.39022200	-12.01742100
C	-19.13037700	1.12658500	-13.01806100
H	-20.02217400	0.88438100	-11.06643900
C	-16.98378400	0.06262200	-13.34011300
H	-16.17182900	-0.98580600	-11.63924300
C	-18.04276400	0.82475100	-13.84311100
H	-19.96710300	1.71418200	-13.40535500
H	-16.13295900	-0.18243400	-13.98177400
H	-18.02365300	1.17662600	-14.87792900
C	-17.02593900	-2.03145800	-9.30933600
C	-17.54455000	-3.31721000	-9.55291500
C	-15.66301600	-1.91379400	-8.98600000
C	-16.72509700	-4.44665600	-9.49765800
H	-18.60768300	-3.43171100	-9.78472100
C	-14.84408200	-3.04614300	-8.91510100
H	-15.22217600	-0.93505300	-8.78512500

C	-15.37071600	-4.31397900	-9.17471600
H	-17.14795600	-5.43523400	-9.69522600
H	-13.78840600	-2.93360000	-8.65444500
H	-14.72967100	-5.19750400	-9.11791600

UB3LYP-D3/def2-SVP-SMD(THF)//UB3LYP/def2-SVP(gas)
HF= -4208.4018406
UPBEPBE-D3/def2-SVP-SMD(THF)//UB3LYP/def2-SVP(gas)
HF= -4205.3139406

^{6}C''

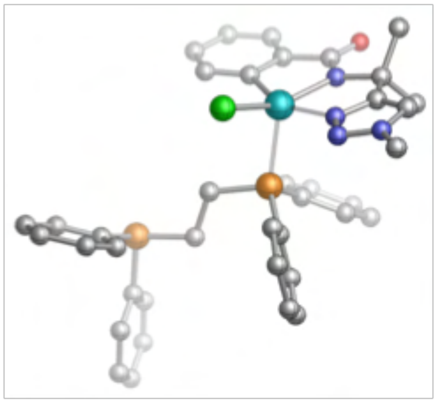

Zero-point correction= 0.681195 (Hartree/Particle)
Thermal correction to Energy= 0.727966
Thermal correction to Enthalpy= 0.728910
Thermal correction to Gibbs Free Energy= 0.589923
Sum of electronic and zero-point Energies= -4207.527025
Sum of electronic and thermal Energies= -4207.480253
Sum of electronic and thermal Enthalpies= -4207.479309
Sum of electronic and thermal Free Energies= -4207.618296

C	-18.44299600	0.84728000	-3.62149100
C	-19.24416100	1.81853300	-2.99256000
C	-20.62874000	1.65141900	-2.86147500
C	-21.22905900	0.49071500	-3.35614300
C	-20.44433100	-0.49996800	-3.96306400
C	-19.05962800	-0.32395900	-4.09016100
H	-21.20791100	2.43196700	-2.36174600
H	-22.30884400	0.34698700	-3.25759400
H	-20.91460000	-1.41703800	-4.33140000
H	-18.45641200	-1.11542500	-4.54689000
C	-18.56768000	3.02815300	-2.40444800
O	-19.20817500	3.90576300	-1.82399700
C	-16.40307800	4.06498700	-1.93368800
C	-16.57119400	4.05696600	-0.39379200
H	-15.91330400	4.80638000	0.07637900
H	-17.61292500	4.28731600	-0.13917600
H	-16.31581100	3.06539900	0.01045800
C	-16.73390900	5.46748800	-2.50181500
H	-17.77687900	5.71494100	-2.26944200
H	-16.07206600	6.23342900	-2.06466500
H	-16.60466600	5.47468500	-3.59453300
C	-14.96294500	3.73547100	-2.25941600
N	-14.66270900	2.67003000	-3.04727500
N	-13.37346800	2.54509600	-3.19736600
C	-13.75595100	4.30906100	-1.90125800
H	-13.49927100	5.16523000	-1.28412100
N	-12.81485000	3.53378000	-2.50937800
N	-17.20221400	3.00986300	-2.57123200
Fe	-16.46784500	1.48586200	-3.70293700
P	-16.52913400	2.12198000	-6.20349400

C	-17.29631700	0.75422500	-8.63834500
H	-17.92201300	1.61478000	-8.92632300
H	-16.30902300	0.92154400	-9.09663600
C	-17.19935200	0.64439400	-7.11592300
H	-16.53468500	-0.17647200	-6.80084400
H	-18.17688200	0.41943100	-6.66085600
C	-14.93142500	2.47531100	-7.05306400
C	-14.83538000	3.26758800	-8.21205300
C	-13.77261800	1.85967300	-6.54595700
C	-13.60286200	3.44640400	-8.84558900
H	-15.72421300	3.74776700	-8.62578900
C	-12.54401500	2.03632900	-7.19034800
H	-13.83088000	1.23541800	-5.65113900
C	-12.45465800	2.83100500	-8.33639900
H	-13.54224400	4.06430700	-9.74523200
H	-11.65224400	1.54965100	-6.78714400
H	-11.49196200	2.97043000	-8.83532100
C	-17.65167600	3.52577500	-6.57532100
C	-19.02666500	3.30566100	-6.77096900
C	-17.18033300	4.85102200	-6.52563700
C	-19.90145800	4.38296400	-6.93725500
H	-19.43063000	2.29167700	-6.78033600
C	-18.05729700	5.92449000	-6.69564100
H	-16.11994200	5.04947200	-6.35366500
C	-19.42044700	5.69396300	-6.90459600
H	-20.96700000	4.19192400	-7.08573200
H	-17.67312300	6.94702600	-6.65786100
H	-20.10665300	6.53467800	-7.03260500
Cl	-15.25855700	-0.42124100	-3.95799600
C	-11.36907000	3.66795600	-2.47148800
H	-11.06225700	4.63533700	-2.89551700
H	-11.00458900	3.58946700	-1.43680400
H	-10.94849200	2.85242700	-3.07134500
P	-18.15894900	-0.72681600	-9.41756400
C	-18.07541300	-0.20004000	-11.19922800
C	-19.18676400	0.49469200	-11.71014100
C	-16.98999900	-0.45208700	-12.05523600
C	-19.20487300	0.94615000	-13.03273600
H	-20.05168300	0.67652700	-11.06477600
C	-17.01245500	-0.01070600	-13.38180400
H	-16.12108400	-1.00166200	-11.68604600
C	-18.11679300	0.69230600	-13.87321700
H	-20.07658400	1.48690100	-13.41095000
H	-16.16099000	-0.21842500	-14.03564700
H	-18.13243600	1.03519600	-14.91109100
C	-16.87762900	-2.06252100	-9.33067700
C	-17.32959600	-3.37766400	-9.54849400
C	-15.51778600	-1.86622400	-9.03414200
C	-16.44856500	-4.45978800	-9.49435400
H	-18.38889300	-3.55323300	-9.75851400
C	-14.63693000	-2.95101700	-8.96421700

H	-15.12781600	-0.86240300	-8.85287700
C	-15.09799900	-4.24897200	-9.19830600
H	-16.82014600	-5.47256500	-9.67118300
H	-13.58476300	-2.77779600	-8.72358600
H	-14.40872500	-5.09540100	-9.14184300

UB3LYP-D3/def2-SVP-SMD(THF)//UB3LYP/def2-SVP(gas)
HF= -4208.3799396
UPBEPBE-D3/def2-SVP-SMD(THF)//UB3LYP/def2-SVP(gas)
HF= -4205.2800206

[1]C’-outer-TS

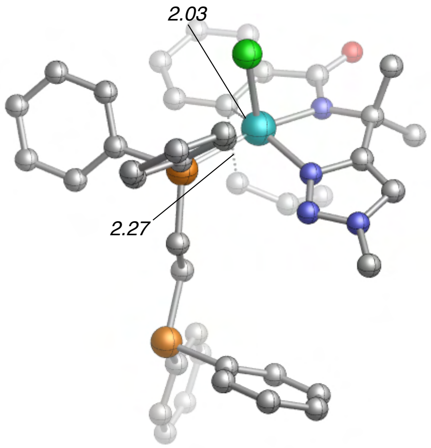

Zero-point correction= 0.751221 (Hartree/Particle)
Thermal correction to Energy= 0.801579
Thermal correction to Enthalpy= 0.802523
Thermal correction to Gibbs Free Energy= 0.661584
Sum of electronic and zero-point Energies= -4324.595752
Sum of electronic and thermal Energies= -4324.545394
Sum of electronic and thermal Enthalpies= -4324.544450
Sum of electronic and thermal Free Energies= -4324.685389

C	-20.65873900	-2.87630800	-4.53902200
C	-21.23370400	-1.71392600	-3.95716100
C	-22.54198900	-1.70798700	-3.46716900
C	-23.30897500	-2.87316400	-3.52596100
C	-22.75514700	-4.04561300	-4.06854100
C	-21.45437900	-4.04716300	-4.56794000
H	-22.92477300	-0.78217800	-3.02992300
H	-24.33341600	-2.87876500	-3.14377300
H	-23.34921500	-4.96333700	-4.10524800
H	-21.06749600	-4.96452300	-5.01540000
C	-20.36521200	-0.50359800	-3.82273600
O	-20.78632400	0.59282000	-3.44908700
C	-18.00227400	0.17812500	-4.01462900
C	-17.75123400	0.52586700	-2.52576200
H	-16.89756900	1.21587500	-2.41996200
H	-18.65195800	1.00827900	-2.12224600
H	-17.55455300	-0.39030400	-1.95123000
C	-18.27201300	1.46800600	-4.82242700
H	-19.18152400	1.94619400	-4.44001800
H	-17.42537900	2.16797500	-4.72440500
H	-18.41822200	1.23723700	-5.88740700
C	-16.79091800	-0.51983900	-4.58502700
N	-16.86938900	-1.84947700	-4.87509200
N	-15.74375900	-2.29054300	-5.37464300
C	-15.51190700	-0.12694400	-4.93070000
H	-14.99777100	0.82826200	-4.88181500
N	-14.91855300	-1.25664000	-5.41234500
N	-19.07820700	-0.81722800	-4.14840000

Fe	-18.64931100	-2.67710800	-4.30558800
P	-17.78314200	-4.99016300	-4.80273800
C	-17.03800600	-5.29606600	-6.49680100
H	-16.75315800	-6.35915600	-6.55171100
H	-16.09984100	-4.72098100	-6.46888500
C	-18.92394100	-6.41480200	-4.51566700
C	-19.50120600	-6.52636500	-3.23577000
C	-19.24583300	-7.37027300	-5.49262000
C	-20.37172800	-7.57729700	-2.94468900
H	-19.27231100	-5.77622900	-2.47370800
C	-20.12795000	-8.41746800	-5.19830000
H	-18.81147900	-7.31757100	-6.49254000
C	-20.69052400	-8.52447400	-3.92487400
H	-20.81007000	-7.65207500	-1.94628400
H	-20.36994600	-9.15250200	-5.97039800
H	-21.37705100	-9.34353700	-3.69540800
C	-16.31099800	-5.52423500	-3.79221500
C	-15.59833300	-4.62660500	-2.98319800
C	-15.86763400	-6.85692700	-3.87496400
C	-14.46210500	-5.05094400	-2.28608800
H	-15.95665800	-3.60651800	-2.86052400
C	-14.73133600	-7.27783600	-3.18034400
H	-16.41954400	-7.58419000	-4.47534200
C	-14.02223000	-6.37288500	-2.38466400
H	-13.92731300	-4.34171800	-1.64861400
H	-14.40566200	-8.31871300	-3.25502100
H	-13.13717600	-6.70187800	-1.83354300
Cl	-18.42837600	-3.13458500	-2.08071000
C	-20.53240200	-0.16728200	-7.44845200
H	-21.60642800	-0.05591300	-7.27194100
H	-19.99016200	0.71496200	-7.79600000
C	-19.90054300	-1.35505000	-7.23215500
H	-18.81714800	-1.40003400	-7.39851800
C	-20.54807800	-2.54256400	-6.78417300
H	-21.63873600	-2.56323700	-6.80618400
H	-20.06367900	-3.50408000	-6.94353300
P	-17.05673500	-5.51848600	-9.30498300
C	-18.18216500	-4.86802700	-10.62417000
C	-17.95333800	-5.34351000	-11.92996000
C	-19.25731700	-3.98745000	-10.41402600
C	-18.75783100	-4.93574300	-12.99524900
H	-17.13428900	-6.04605700	-12.11138700
C	-20.07236000	-3.58884100	-11.48004300
H	-19.47431800	-3.59592700	-9.41891200
C	-19.82368600	-4.05710000	-12.77211400
H	-18.55890600	-5.31321700	-14.00174800
H	-20.90373600	-2.90398600	-11.29380000
H	-20.46087200	-3.74320700	-13.60290000
C	-17.85715100	-4.88500300	-7.72347800
H	-17.97766400	-3.79161600	-7.75287800
H	-18.86539900	-5.33039700	-7.70481400

C	-15.59922700	-4.36739200	-9.44543300
C	-14.33470200	-4.87477400	-9.09938600
C	-15.69035200	-3.03675200	-9.89116800
C	-13.19096100	-4.07336600	-9.18520200
H	-14.24409900	-5.91403700	-8.77085000
C	-14.54821900	-2.23691600	-9.98551600
H	-16.66044300	-2.62503700	-10.18069800
C	-13.29508300	-2.75304100	-9.63342900
H	-12.21523100	-4.48822000	-8.91823200
H	-14.63511600	-1.20823300	-10.34612400
H	-12.40025700	-2.13098900	-9.72358200
C	-13.55917100	-1.43351300	-5.89971600
H	-13.10087000	-0.44583300	-6.03334100
H	-12.97243200	-2.01703100	-5.17538500
H	-13.58310200	-1.96294100	-6.86168600

UB3LYP-D3/def2-SVP-SMD(THF)//UB3LYP/def2-SVP(gas)
HF= -4325.5237476
UPBEPBE-D3/def2-SVP-SMD(THF)//UB3LYP/def2-SVP(gas)
HF= -4322.2815579

^{3}C'-outer-TS

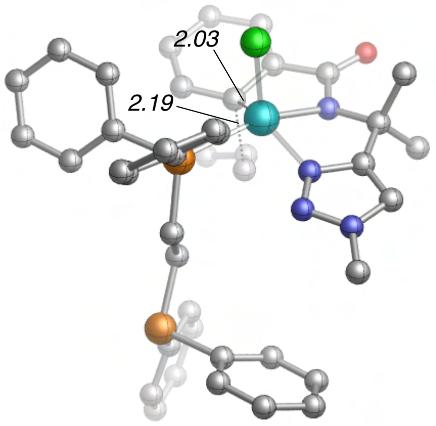

Zero-point correction= 0.750567 (Hartree/Particle)
Thermal correction to Energy= 0.801086
Thermal correction to Enthalpy= 0.802030
Thermal correction to Gibbs Free Energy= 0.658872
Sum of electronic and zero-point Energies= -4324.619169
Sum of electronic and thermal Energies= -4324.568650
Sum of electronic and thermal Enthalpies= -4324.567706
Sum of electronic and thermal Free Energies= -4324.710864

C	-20.36900800	-2.58830500	-4.76690600
C	-20.88390800	-1.39975900	-4.15746300
C	-22.20331500	-1.29737900	-3.72020900
C	-23.05149200	-2.40244600	-3.82243900
C	-22.55720500	-3.61208900	-4.34833800
C	-21.24222500	-3.71366000	-4.78633100
H	-22.53348300	-0.35214800	-3.28119700
H	-24.08732000	-2.33816000	-3.47919300
H	-23.21302900	-4.48569700	-4.40786200
H	-20.90527100	-4.66486800	-5.20167600
C	-19.92787500	-0.27067300	-4.00548700
O	-20.24680400	0.87217000	-3.68099400
C	-17.50116100	0.19667000	-4.12628000
C	-17.31278100	0.63896200	-2.65409100
H	-16.40916300	1.26079100	-2.54314000
H	-18.18983400	1.22174900	-2.34280400
H	-17.22689800	-0.24473000	-2.00509600
C	-17.62096600	1.42640600	-5.05480100
H	-18.50471400	2.01025000	-4.77043000
H	-16.72602900	2.06501700	-4.96969300
H	-17.72643800	1.11073500	-6.10440600
C	-16.31916800	-0.63680200	-4.55466400
N	-16.48376100	-1.97791900	-4.72111100
N	-15.36770100	-2.54545600	-5.09476900
C	-14.99395500	-0.37143800	-4.84287100
H	-14.40965300	0.54390900	-4.84426000
N	-14.46060100	-1.58403300	-5.16584800
N	-18.65428400	-0.71033300	-4.27046200
Fe	-18.40958900	-2.60899700	-4.22042600

P	-17.74309000	-4.99803100	-4.76048200
C	-17.01568500	-5.27339100	-6.46177900
H	-16.79693900	-6.34806000	-6.56834700
H	-16.04268700	-4.76088300	-6.40604800
C	-19.01315400	-6.31454900	-4.52922100
C	-19.65814100	-6.36123300	-3.27830700
C	-19.35774500	-7.25533000	-5.51217000
C	-20.61728000	-7.34075000	-3.01873100
H	-19.41209000	-5.61723300	-2.51500000
C	-20.32841500	-8.22980200	-5.24939500
H	-18.87426700	-7.24591800	-6.49091800
C	-20.95677100	-8.27634600	-4.00315300
H	-21.10885300	-7.36779900	-2.04301600
H	-20.58862700	-8.95552700	-6.02427400
H	-21.71249800	-9.03907700	-3.79847600
C	-16.34700500	-5.68504600	-3.73988400
C	-15.63907700	-4.89874000	-2.81931500
C	-15.97182000	-7.03001500	-3.91409400
C	-14.56798200	-5.44398200	-2.10297300
H	-15.95351600	-3.87422100	-2.62783500
C	-14.89941600	-7.56980800	-3.20117000
H	-16.52840000	-7.67368100	-4.59959400
C	-14.19107200	-6.77500300	-2.29427600
H	-14.03489000	-4.82169900	-1.37925200
H	-14.62386300	-8.61738800	-3.34818100
H	-13.35643800	-7.19806900	-1.72889200
Cl	-18.30957400	-3.04131900	-1.95341400
C	-22.64510400	-2.27039800	-7.80301500
H	-22.54487900	-3.33737800	-8.02663900
H	-23.60631600	-1.80207700	-8.02886100
C	-21.61954100	-1.56089600	-7.26444400
H	-21.78108100	-0.49807200	-7.04872500
C	-20.34257200	-2.10319400	-6.90400700
H	-20.10669100	-3.10932900	-7.25415900
H	-19.49338600	-1.42195800	-6.82485100
P	-17.00875900	-5.32257400	-9.27119700
C	-18.11474100	-4.59986000	-10.56985700
C	-17.89394000	-5.03740000	-11.89013300
C	-19.16935500	-3.70195700	-10.33297800
C	-18.68736300	-4.57629900	-12.94207000
H	-17.09149600	-5.75286800	-12.09390700
C	-19.97519400	-3.25113800	-11.38502700
H	-19.37869200	-3.33667500	-9.32690700
C	-19.73456700	-3.68266700	-12.69135900
H	-18.49518100	-4.92489800	-13.96026700
H	-20.79301400	-2.55762900	-11.17299400
H	-20.36383200	-3.32893000	-13.51212900
C	-17.81033100	-4.75535700	-7.66481400
H	-17.89991600	-3.65899100	-7.62352600
H	-18.83243800	-5.16826000	-7.67830500
C	-15.53750800	-4.18126800	-9.35654000

C	-14.26074900	-4.74703600	-9.20101800
C	-15.63589100	-2.79749800	-9.58786100
C	-13.10995400	-3.95354000	-9.27069700
H	-14.16784300	-5.82431700	-9.03684100
C	-14.48876900	-2.00320900	-9.66070300
H	-16.61689000	-2.33695900	-9.73050500
C	-13.22184000	-2.57994000	-9.50504100
H	-12.12452600	-4.41354300	-9.15734300
H	-14.58286700	-0.93116600	-9.85461400
H	-12.32377000	-1.96073600	-9.58299200
C	-13.10882200	-1.89503600	-5.60378200
H	-13.05840800	-1.91944100	-6.70245200
H	-12.42168300	-1.13349300	-5.21349200
H	-12.83331500	-2.87989200	-5.20641200

UB3LYP-D3/def2-SVP-SMD(THF)//UB3LYP/def2-SVP(gas)
HF= -4325.5684905
UPBEPBE-D3/def2-SVP-SMD(THF)//UB3LYP/def2-SVP(gas)
HF= -4322.3169181

[5]C'-outer-TS

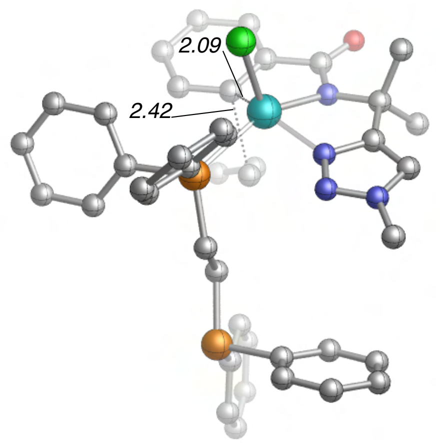

Zero-point correction= 0.749388 (Hartree/Particle)
Thermal correction to Energy= 0.800479
Thermal correction to Enthalpy= 0.801424
Thermal correction to Gibbs Free Energy= 0.655575
Sum of electronic and zero-point Energies= -4324.630698
Sum of electronic and thermal Energies= -4324.579607
Sum of electronic and thermal Enthalpies= -4324.578663
Sum of electronic and thermal Free Energies= -4324.724511

C	-20.14205400	-2.56849900	-4.51081200
C	-20.54556400	-1.26115300	-4.17814900
C	-21.85554400	-1.00156200	-3.77507000
C	-22.77670000	-2.05167700	-3.69335500
C	-22.37711600	-3.35701300	-4.01063500
C	-21.06645400	-3.61836600	-4.42702900
H	-22.12235900	0.02780000	-3.52090100
H	-23.80538600	-1.85694400	-3.37853300
H	-23.09318700	-4.18103900	-3.93979500
H	-20.78433600	-4.63926100	-4.68971700
C	-19.51695600	-0.16990600	-4.24184500
O	-19.80624500	1.01892400	-4.09258000
C	-17.10200300	0.20091100	-4.46771100
C	-16.88103300	0.84152800	-3.07414500
H	-15.96375700	1.45320800	-3.05741600
H	-17.74161000	1.48002100	-2.83435900
H	-16.80037600	0.05346500	-2.31048900
C	-17.19822100	1.29287300	-5.55829800
H	-18.06182100	1.93591200	-5.34989200
H	-16.28530100	1.91143100	-5.57685900
H	-17.32795200	0.83593300	-6.55202700
C	-15.93043900	-0.70268400	-4.78000100
N	-16.09544700	-2.05326500	-4.78922900
N	-14.97322700	-2.66024700	-5.07160900
C	-14.59813800	-0.47348800	-5.07383800
H	-14.01112400	0.43492600	-5.17138900
N	-14.06124300	-1.71479400	-5.23931500
N	-18.27372800	-0.68743700	-4.45658800

Fe	-18.07439100	-2.65506000	-4.19302200
P	-17.35589700	-5.15246000	-4.84819500
C	-16.52631200	-5.46036600	-6.50135200
H	-16.34246100	-6.54310300	-6.59608700
H	-15.54087200	-4.98305100	-6.38391100
C	-18.67093600	-6.44087400	-4.70996700
C	-19.34615400	-6.52112500	-3.47580700
C	-19.03268500	-7.32292100	-5.74074900
C	-20.34832900	-7.47221900	-3.27949500
H	-19.08727100	-5.82483800	-2.67253400
C	-20.04726100	-8.26760800	-5.54284000
H	-18.52443400	-7.29369100	-6.70650300
C	-20.70411400	-8.34674400	-4.31307600
H	-20.85926500	-7.52536400	-2.31478300
H	-20.31793200	-8.94702400	-6.35532000
H	-21.49299600	-9.08745200	-4.15905300
C	-16.04599800	-5.88269900	-3.74694600
C	-15.35185000	-5.09690500	-2.81520100
C	-15.71513800	-7.24501800	-3.86795700
C	-14.33805700	-5.66137000	-2.03308600
H	-15.62986500	-4.05427700	-2.66975100
C	-14.70040100	-7.80439300	-3.08881700
H	-16.26182200	-7.88435800	-4.56565200
C	-14.00601100	-7.01093600	-2.16963500
H	-13.81482800	-5.03911800	-1.30220100
H	-14.45815200	-8.86520100	-3.19429500
H	-13.21636400	-7.44886800	-1.55318300
Cl	-17.99517100	-3.13367700	-1.96424300
C	-21.95500000	-2.36683000	-7.89660600
H	-22.07851700	-3.45417600	-7.90930200
H	-22.78436800	-1.76405900	-8.27399400
C	-20.81563800	-1.78835200	-7.41730100
H	-20.75202800	-0.69405700	-7.40952100
C	-19.70824200	-2.49990400	-6.88736500
H	-19.68541900	-3.58716500	-6.96465500
H	-18.76284200	-1.98080500	-6.73248700
P	-16.39673200	-5.52422300	-9.32144100
C	-17.42221300	-4.73580900	-10.64787400
C	-17.12945500	-5.11100700	-11.97360200
C	-18.49043100	-3.84958600	-10.42707700
C	-17.86476300	-4.60056600	-13.04443600
H	-16.31513500	-5.81585800	-12.16722700
C	-19.23752300	-3.34887300	-11.49998400
H	-18.75732900	-3.53317400	-9.41753300
C	-18.92540800	-3.71817800	-12.81004500
H	-17.61639600	-4.90113200	-14.06578300
H	-20.06640300	-2.66442100	-11.30163000
H	-19.50889000	-3.32510600	-13.64656300
C	-17.22700000	-4.91318600	-7.74853300
H	-17.26252900	-3.81348300	-7.72086800
H	-18.26701200	-5.27236800	-7.81041100

C	-14.86467000	-4.46484500	-9.35816800
C	-13.63309200	-5.08658200	-9.08978400
C	-14.87201200	-3.09236400	-9.66520900
C	-12.43873400	-4.35811800	-9.12158400
H	-13.60924600	-6.15728200	-8.86787900
C	-13.68017300	-2.36390300	-9.70366700
H	-15.81592800	-2.59117200	-9.89440800
C	-12.45952600	-2.99565600	-9.43417200
H	-11.48939600	-4.86113200	-8.91877000
H	-13.70241000	-1.30071600	-9.95848700
H	-11.52555700	-2.42894800	-9.48342100
C	-12.70257700	-2.07495900	-5.61291500
H	-12.47114200	-3.04957200	-5.16595000
H	-12.61061000	-2.14621700	-6.70694300
H	-12.00876800	-1.31685500	-5.22695200

UB3LYP-D3/def2-SVP-SMD(THF)//UB3LYP/def2-SVP(gas)
HF= -4325.5765320
UPBEPBE-D3/def2-SVP-SMD(THF)//UB3LYP/def2-SVP(gas)
HF= -4322.3119478

[1]D-TS

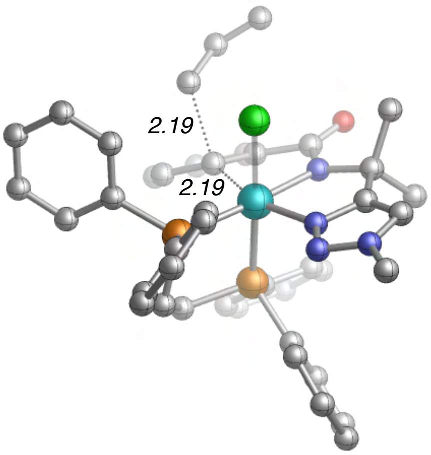

Zero-point correction= 0.754050 (Hartree/Particle)
Thermal correction to Energy= 0.801988
Thermal correction to Enthalpy= 0.802932
Thermal correction to Gibbs Free Energy= 0.673546
Sum of electronic and zero-point Energies= -4324.593767
Sum of electronic and thermal Energies= -4324.545829
Sum of electronic and thermal Enthalpies= -4324.544884
Sum of electronic and thermal Free Energies= -4324.674271

C	-18.95696700	1.10575300	-3.60949300
C	-19.40305600	2.46467500	-3.46186100
C	-20.57714300	2.94039000	-4.04286100
C	-21.35313700	2.09403200	-4.84087200
C	-20.95781000	0.76485300	-5.04679400
C	-19.80960600	0.28693000	-4.41461300
H	-20.85598100	3.98131100	-3.86527500
H	-22.27220400	2.46753100	-5.30199200
H	-21.56961000	0.09664300	-5.65952900
H	-19.57369100	-0.77633800	-4.51297900
C	-18.53001600	3.42657300	-2.70650800
O	-19.00256800	4.45781900	-2.19456600
C	-16.20352300	3.85382600	-2.13439100
C	-16.41201600	3.97420300	-0.60458000
H	-15.60177100	4.56580300	-0.14514000
H	-17.37023000	4.47609500	-0.41655200
H	-16.42509500	2.97163000	-0.15544400
C	-16.11454600	5.26635500	-2.76384700
H	-17.08404200	5.76963300	-2.65237900
H	-15.33804300	5.86870300	-2.26405900
H	-15.85319600	5.20296500	-3.83163300
C	-14.92077700	3.10981000	-2.41125800
N	-14.94494900	1.95035300	-3.13192300
N	-13.74765700	1.44876000	-3.23609400
C	-13.60410900	3.32251100	-2.05637300
H	-13.11697100	4.10183900	-1.47771800
N	-12.92597100	2.26741400	-2.58701300
N	-17.24869800	3.03182200	-2.76221600

Fe	-16.77478500	1.25130800	-3.52879500
P	-16.23468400	-0.90122800	-4.32757500
P	-16.78291400	1.88424000	-5.77911900
C	-16.13271500	-0.80447500	-6.19790700
H	-16.34352800	-1.76824500	-6.68445700
H	-15.08342500	-0.56851000	-6.43024000
C	-17.04569700	0.29755600	-6.74259100
H	-18.10501800	0.02582300	-6.61213700
H	-16.86586600	0.46223100	-7.81658200
C	-17.44332300	-2.28118500	-4.00603600
C	-17.66454600	-2.65359400	-2.66649000
C	-18.14379500	-2.96273000	-5.01481800
C	-18.55154000	-3.68616500	-2.35324500
H	-17.14869900	-2.11478200	-1.86765900
C	-19.03701700	-3.99295000	-4.69762000
H	-18.00861100	-2.69936000	-6.06542400
C	-19.24207700	-4.36036700	-3.36617500
H	-18.70550700	-3.96335400	-1.30690500
H	-19.57109700	-4.51019200	-5.49933600
H	-19.93591500	-5.16792400	-3.11842200
C	-14.65803100	-1.80099600	-3.90889300
C	-14.03665300	-2.66339200	-4.82964500
C	-14.09130700	-1.65621000	-2.63172200
C	-12.87128200	-3.35575700	-4.48806400
H	-14.46024400	-2.81571600	-5.82431000
C	-12.93217900	-2.35982300	-2.28916300
H	-14.57191600	-0.99460300	-1.90704300
C	-12.31496700	-3.20623400	-3.21458900
H	-12.40283400	-4.02070600	-5.21876800
H	-12.51016600	-2.24359400	-1.28688300
H	-11.40775000	-3.75330500	-2.94375000
C	-15.23912200	2.52918800	-6.61592700
C	-15.26963400	3.55469900	-7.57924800
C	-13.99624100	1.93641600	-6.32232000
C	-14.10134600	3.96928300	-8.22637200
H	-16.21182500	4.03938400	-7.83601400
C	-12.83108600	2.34439200	-6.97876100
H	-13.91943300	1.16562100	-5.55660700
C	-12.87725100	3.36389800	-7.93297300
H	-14.15401000	4.77011000	-8.96880700
H	-11.88067500	1.86168400	-6.73490100
H	-11.96553500	3.68618600	-8.44284200
C	-18.05392800	3.02521000	-6.48738600
C	-18.89792000	2.67163400	-7.55291600
C	-18.15819000	4.32248900	-5.95326400
C	-19.81755200	3.58958900	-8.07131600
H	-18.84800000	1.67689600	-7.99679800
C	-19.06922700	5.24165700	-6.47687200
H	-17.52588500	4.61754600	-5.11576700
C	-19.90434100	4.87751400	-7.53759000
H	-20.46573000	3.29399800	-8.90064200

H	-19.13368400	6.24221100	-6.04232500
H	-20.62152700	5.59472600	-7.94533600
Cl	-16.51362400	0.37081200	-1.22479100
C	-20.05745000	1.83705400	-0.01880900
C	-20.53172600	1.11386200	-1.05368900
H	-21.58025200	1.21399900	-1.35463200
C	-19.69547800	0.17692800	-1.77333600
H	-18.68674800	0.00283700	-1.38793800
H	-20.18931700	-0.67553800	-2.23933800
C	-11.50251800	1.99186200	-2.55889100
H	-11.10174700	2.20975900	-1.55915200
H	-11.36231400	0.92771100	-2.78530300
H	-10.96883200	2.59988400	-3.30619600
H	-20.69996000	2.52108300	0.54137900
H	-19.00747700	1.76013200	0.27753700

UB3LYP-D3/def2-SVP-SMD(THF)//UB3LYP/def2-SVP(gas)
HF= -4325.5586621
UPBEPBE-D3/def2-SVP-SMD(THF)//UB3LYP/def2-SVP(gas)
HF= -4322.3246608

^{3}D-TS

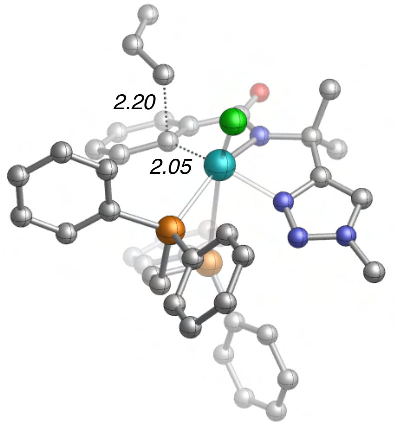

Zero-point correction= 0.750676 (Hartree/Particle)
Thermal correction to Energy= 0.801083
Thermal correction to Enthalpy= 0.802027
Thermal correction to Gibbs Free Energy= 0.659694
Sum of electronic and zero-point Energies= -4324.613703
Sum of electronic and thermal Energies= -4324.563296
Sum of electronic and thermal Enthalpies= -4324.562352
Sum of electronic and thermal Free Energies= -4324.704685

C	-18.83113400	1.20991500	-3.10888800
C	-19.25057000	2.56968300	-3.03880900
C	-20.40645700	3.02895000	-3.66142400
C	-21.16647600	2.15473100	-4.44834500
C	-20.75071200	0.82097400	-4.59237900
C	-19.60557100	0.35571500	-3.94642200
H	-20.68433100	4.07823600	-3.53274300
H	-22.07027200	2.50711400	-4.95165100
H	-21.33795300	0.13185600	-5.20737200
H	-19.35322600	-0.70182100	-4.03316300
C	-18.38672200	3.48080400	-2.23498600
O	-18.76091700	4.57840500	-1.81951300
C	-16.04005900	3.70495100	-1.48126400
C	-16.14250700	3.72642500	0.06030200
H	-15.29511300	4.27123000	0.50959600
H	-17.07439100	4.23562700	0.34503500
H	-16.15573400	2.69938200	0.45265000
C	-15.94771000	5.14851100	-2.03683500
H	-16.83460000	5.72191700	-1.74742600
H	-15.04414800	5.64393600	-1.64520300
H	-15.88436900	5.13074700	-3.13589900
C	-14.79443000	2.97714200	-1.92915200
N	-14.90946000	1.85098900	-2.68466700
N	-13.73853800	1.40066800	-3.03733100
C	-13.44079700	3.22188800	-1.80079200
H	-12.87775600	3.99783000	-1.29054400
N	-12.84074300	2.22033700	-2.50296300
N	-17.14962900	2.92409300	-2.06405000

Fe	-16.84811100	1.09785100	-2.60816100
P	-16.24158800	-0.87542300	-4.11353700
P	-16.63686000	2.33069400	-6.02925900
C	-16.09962700	-0.46223700	-5.93304000
H	-16.17215200	-1.39865200	-6.50623400
H	-15.06178500	-0.11681700	-6.06454200
C	-17.08481200	0.56857900	-6.50580900
H	-18.10855000	0.39279800	-6.14017200
H	-17.10080800	0.46946700	-7.60362000
C	-17.42346800	-2.29974100	-4.02265700
C	-17.67268000	-2.83768500	-2.74551700
C	-18.06829900	-2.86343300	-5.13458800
C	-18.53800700	-3.92308300	-2.59339800
H	-17.19273700	-2.39503300	-1.86751000
C	-18.93936700	-3.94719200	-4.97610000
H	-17.90930200	-2.46181500	-6.13718700
C	-19.17425000	-4.48147300	-3.70725600
H	-18.71944100	-4.33095400	-1.59571000
H	-19.43583900	-4.37311200	-5.85198400
H	-19.85410700	-5.32856800	-3.58541700
C	-14.63957600	-1.78786200	-3.84009000
C	-14.08811700	-2.58378100	-4.86114900
C	-13.98781200	-1.73727600	-2.59818200
C	-12.90590100	-3.29779200	-4.65122500
H	-14.57897000	-2.66773700	-5.83232300
C	-12.80953300	-2.46204300	-2.38873000
H	-14.41416900	-1.14260500	-1.78801500
C	-12.26097100	-3.23835100	-3.41225000
H	-12.49371200	-3.90943900	-5.45816400
H	-12.32142900	-2.41863500	-1.41121000
H	-11.33977600	-3.80297800	-3.24478700
C	-15.20147200	2.61495200	-7.18321500
C	-15.33619500	3.04545900	-8.51512000
C	-13.90652500	2.38728300	-6.68100500
C	-14.21084800	3.23116900	-9.32300300
H	-16.32931200	3.24332400	-8.92497400
C	-12.78157400	2.55969100	-7.49453000
H	-13.77815300	2.07357800	-5.64117700
C	-12.93026100	2.98517500	-8.81720600
H	-14.33556800	3.57051700	-10.35495100
H	-11.78367900	2.36907100	-7.08987000
H	-12.05157400	3.13022400	-9.45114300
C	-17.97842000	3.31923800	-6.83060500
C	-18.92274300	2.81324700	-7.73991400
C	-18.06595500	4.67454700	-6.46158900
C	-19.91654800	3.64136500	-8.27293300
H	-18.89570900	1.76484000	-8.04244400
C	-19.05057000	5.50435000	-7.00107300
H	-17.35911300	5.08181300	-5.73316100
C	-19.98105200	4.98867200	-7.90915700
H	-20.64426900	3.22833700	-8.97670600

H	-19.10003000	6.55387700	-6.69987200
H	-20.75900100	5.63391000	-8.32548600
Cl	-16.27131900	-0.09479700	-0.62382600
C	-21.44426200	1.67163600	-0.52590200
H	-22.53583600	1.71426900	-0.48507200
H	-20.89462300	2.54396600	-0.15856200
C	-20.79632600	0.59106900	-1.02270900
H	-21.39095100	-0.24473900	-1.40993100
C	-19.36504500	0.47311600	-1.10915300
H	-18.75707100	1.16417400	-0.52477500
H	-18.93517300	-0.52246500	-1.20999900
C	-11.42397800	1.99711100	-2.73251000
H	-11.02375400	2.73602600	-3.44335900
H	-10.87650100	2.07029900	-1.78242600
H	-11.30909600	0.98767300	-3.14513700

UB3LYP-D3/def2-SVP-SMD(THF)//UB3LYP/def2-SVP(gas)
HF= -4325.5704508
UPBEPBE-D3/def2-SVP-SMD(THF)//UB3LYP/def2-SVP(gas)
HF= -4322.3185069

^{5}D-TS

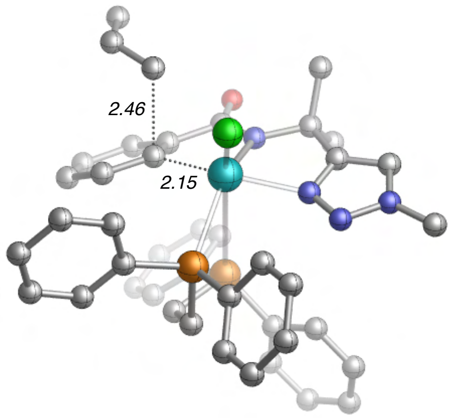

Zero-point correction= 0.749229 (Hartree/Particle)
Thermal correction to Energy= 0.799380
Thermal correction to Enthalpy= 0.800324
Thermal correction to Gibbs Free Energy= 0.658525
Sum of electronic and zero-point Energies= -4324.628150
Sum of electronic and thermal Energies= -4324.578000
Sum of electronic and thermal Enthalpies= -4324.577055
Sum of electronic and thermal Free Energies= -4324.718854

C	-18.89850400	0.99010600	-3.01159200
C	-19.24189000	2.34059300	-3.20063100
C	-20.39284500	2.69020100	-3.91093300
C	-21.20109100	1.69073800	-4.46147900
C	-20.86236900	0.34208700	-4.28354900
C	-19.72685100	-0.01052800	-3.54412900
H	-20.62940700	3.75133300	-4.02130700
H	-22.10060000	1.95962500	-5.02161900
H	-21.50162200	-0.44335600	-4.69769700
H	-19.51687700	-1.06519300	-3.35886100
C	-18.35412600	3.39519400	-2.60135700
O	-18.75662500	4.55010900	-2.42160100
C	-16.06861100	3.79061100	-1.79990900
C	-16.32603400	4.13188500	-0.31428000
H	-15.52544400	4.76838800	0.09867100
H	-17.27904000	4.67333900	-0.23321800
H	-16.38307500	3.20997800	0.28419500
C	-15.90502600	5.09222400	-2.62689000
H	-16.82215600	5.68872100	-2.56711700
H	-15.05664200	5.68279600	-2.24390100
H	-15.70586500	4.84857800	-3.68169800
C	-14.77958500	3.00933900	-1.93942300
N	-14.77451300	1.77718900	-2.51694600
N	-13.56258600	1.30278500	-2.58989500
C	-13.46397700	3.30236100	-1.62974300
H	-12.99029400	4.16142900	-1.16355900
N	-12.76011500	2.21702000	-2.05446200
N	-17.11948600	2.90262800	-2.32063500
Fe	-16.78504800	0.93704600	-2.59925900

P	-16.18583200	-1.00778000	-4.33496800
P	-16.73254300	2.22772100	-5.97274100
C	-16.08374600	-0.52853300	-6.13737600
H	-16.15358900	-1.43745500	-6.75433400
H	-15.05957700	-0.14882000	-6.28196100
C	-17.10984900	0.50661400	-6.62122800
H	-18.12723400	0.25512900	-6.27870800
H	-17.12071500	0.50641900	-7.72369700
C	-17.39762700	-2.41037800	-4.29959500
C	-17.61779000	-3.03782300	-3.05819700
C	-18.11087900	-2.86166300	-5.42088800
C	-18.51441800	-4.10285300	-2.95278900
H	-17.10280100	-2.67090700	-2.16616400
C	-19.01471200	-3.92459600	-5.30929200
H	-17.97868300	-2.38923100	-6.39586000
C	-19.21575300	-4.55121900	-4.07769000
H	-18.67101500	-4.58058400	-1.98214600
H	-19.56270700	-4.26179200	-6.19317300
H	-19.92007900	-5.38273100	-3.99237500
C	-14.58983300	-1.93971700	-4.12163800
C	-14.03825000	-2.69617500	-5.17257400
C	-13.93322600	-1.92734500	-2.88113500
C	-12.85329700	-3.41247400	-4.99079600
H	-14.53329300	-2.74496600	-6.14438700
C	-12.75206200	-2.65543100	-2.69973700
H	-14.35559100	-1.35919400	-2.05119600
C	-12.20570000	-3.39419800	-3.75125500
H	-12.43976700	-3.99365400	-5.81933600
H	-12.25924100	-2.64258100	-1.72377600
H	-11.28166300	-3.96051600	-3.60659400
C	-15.23143800	2.64165000	-6.99463600
C	-15.28538500	3.14143700	-8.30800300
C	-13.96916200	2.44379300	-6.40422700
C	-14.11230200	3.42274100	-9.01356900
H	-16.25306500	3.31913900	-8.78300600
C	-12.79514300	2.71347000	-7.11569200
H	-13.90436400	2.07698900	-5.37600700
C	-12.86362000	3.20610900	-8.42142800
H	-14.17399800	3.81445900	-10.03258300
H	-11.82338200	2.54550600	-6.64342600
H	-11.94727600	3.42640600	-8.97536700
C	-18.04488700	3.25553000	-6.77712800
C	-18.90487500	2.81093600	-7.79617500
C	-18.20460500	4.56789600	-6.29404200
C	-19.88464200	3.65784100	-8.32574200
H	-18.82236800	1.79592000	-8.18912700
C	-19.17475600	5.41723600	-6.82995400
H	-17.57592500	4.92413200	-5.47411200
C	-20.01910700	4.96413500	-7.84871200
H	-20.54557500	3.29204100	-9.11627500
H	-19.28154800	6.43145400	-6.43697800

H	-20.78550800	5.62452300	-8.26280300
Cl	-16.33751700	-0.44366400	-0.77032100
C	-21.36627400	2.20396600	-0.40119500
C	-20.76009400	0.98526300	-0.50483600
H	-21.39271900	0.09013500	-0.53814800
C	-19.35906100	0.79513200	-0.60441600
H	-18.68277300	1.63009600	-0.41837900
H	-18.91925300	-0.19781600	-0.53569100
C	-11.33003300	1.97599500	-1.98203300
H	-11.14322300	0.98269100	-2.40717400
H	-10.78141100	2.73511200	-2.55905600
H	-10.99183100	1.99761000	-0.93567100
H	-22.45300500	2.29558100	-0.32930000
H	-20.78242300	3.12967500	-0.40857800

UB3LYP-D3/def2-SVP-SMD(THF)//UB3LYP/def2-SVP(gas)
HF= -4325.5787213
UPBEPBE-D3/def2-SVP-SMD(THF)//UB3LYP/def2-SVP(gas)
HF= -4322.3113130

[1]D'

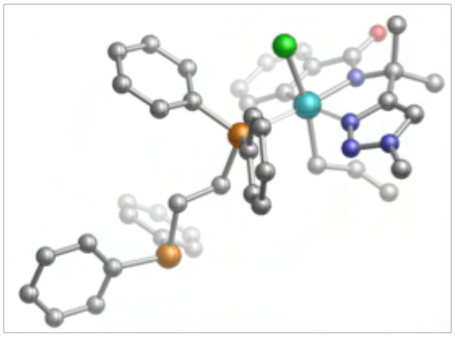

Zero-point correction= 0.753944 (Hartree/Particle)
Thermal correction to Energy= 0.803886
Thermal correction to Enthalpy= 0.804830
Thermal correction to Gibbs Free Energy= 0.665218
Sum of electronic and zero-point Energies= -4324.625393
Sum of electronic and thermal Energies= -4324.575451
Sum of electronic and thermal Enthalpies= -4324.574506
Sum of electronic and thermal Free Energies= -4324.714118

C	-19.90198300	-2.68479600	-3.50442800
C	-20.60060200	-1.66078100	-2.84088600
C	-21.96255600	-1.76379600	-2.53810900
C	-22.66521300	-2.90587800	-2.92524400
C	-21.99662600	-3.92559100	-3.61387800
C	-20.62954300	-3.81479800	-3.90352700
H	-22.44360500	-0.93811100	-2.00754700
H	-23.73107200	-3.00191300	-2.70195200
H	-22.54170600	-4.81850200	-3.93358600
H	-20.15490500	-4.63491500	-4.44648600
C	-19.81327900	-0.44612000	-2.48732500
O	-20.27138600	0.50578700	-1.86077900
C	-17.55401600	0.53286400	-2.69536500
C	-17.26396300	0.65738700	-1.17844700
H	-16.48930600	1.42173700	-1.00208900
H	-18.18324300	0.95744800	-0.65904500
H	-16.92085700	-0.30527900	-0.77623300
C	-18.02145200	1.89566400	-3.26036100
H	-18.94363500	2.20421100	-2.75413100
H	-17.24679800	2.66081400	-3.08704600
H	-18.20850900	1.82301400	-4.34052300
C	-16.30135400	0.10397700	-3.41572000
N	-16.24162400	-1.12904000	-3.98672900
N	-15.10227400	-1.31356900	-4.58781000
C	-15.08976700	0.71325800	-3.67834200
H	-14.68472700	1.68818500	-3.42384300
N	-14.39304400	-0.20768800	-4.40209400
N	-18.52804900	-0.54568900	-2.96959900
Fe	-17.98644200	-2.22456300	-3.63653700
Cl	-17.20529200	-2.93429400	-1.56260200
C	-18.26128800	0.28128200	-6.73304900
H	-17.24082100	0.11534100	-7.09550500

H	-18.72952700	1.23601400	-6.98825100
C	-18.91272100	-0.65168600	-6.01843100
H	-19.93542500	-0.43950000	-5.68465300
C	-18.37626500	-1.97948100	-5.66828700
H	-19.08139700	-2.77352000	-5.93425100
H	-17.39589800	-2.15595300	-6.13329400
C	-13.05917400	-0.09757000	-4.96650100
H	-12.34875000	0.22298600	-4.19139500
H	-12.77558400	-1.08999000	-5.33655000
H	-13.04793400	0.62629900	-5.79531800
P	-17.09102400	-4.49451200	-4.27228100
C	-17.66838100	-5.16252800	-5.92639800
H	-17.32306600	-4.44128700	-6.68383200
H	-18.76374800	-5.05863000	-5.91178500
C	-17.29597600	-6.59395300	-6.32195500
H	-17.75515700	-7.31449200	-5.62873700
H	-16.20546700	-6.74481200	-6.28027700
P	-17.80907800	-6.95648600	-8.09506100
C	-19.65956100	-6.97129800	-7.90982600
C	-20.38454500	-5.92010900	-8.49628100
C	-20.36203300	-7.96040300	-7.19813600
C	-21.77578500	-5.84810700	-8.36390600
H	-19.85292400	-5.15173200	-9.06490000
C	-21.75131600	-7.89251300	-7.07016400
H	-19.81981300	-8.79502100	-6.74582600
C	-22.46114400	-6.83484400	-7.65107700
H	-22.32401200	-5.02142800	-8.82315600
H	-22.28405300	-8.66973300	-6.51563400
H	-23.54845600	-6.78393200	-7.55070700
C	-17.43437800	-8.76479500	-8.25006300
C	-17.74585800	-9.37376000	-9.48164500
C	-16.80367900	-9.54335100	-7.26487600
C	-17.45739200	-10.71907900	-9.71289200
H	-18.22247400	-8.78320400	-10.26995600
C	-16.50219400	-10.89016600	-7.50151700
H	-16.54236800	-9.11101400	-6.29740500
C	-16.83046200	-11.48332200	-8.72194800
H	-17.71533500	-11.17197000	-10.67391400
H	-16.01093200	-11.47783800	-6.72128600
H	-16.59657500	-12.53542500	-8.90364500
C	-17.38245100	-5.91505400	-3.11796900
C	-18.47711000	-5.91289900	-2.23847800
C	-16.52688800	-7.03219500	-3.12860100
C	-18.72511500	-7.01342400	-1.41294700
H	-19.12471000	-5.04019800	-2.17560400
C	-16.77339000	-8.12795900	-2.29700000
H	-15.64980600	-7.04603700	-3.77791100
C	-17.87793100	-8.12379800	-1.44037200
H	-19.58047600	-6.99080200	-0.73313600
H	-16.09438700	-8.98453200	-2.31586900
H	-18.06998900	-8.97921700	-0.78735200

C	-15.25020500	-4.46850500	-4.42906900
C	-14.60053500	-4.48455300	-5.67376700
C	-14.47138000	-4.35970100	-3.26149900
C	-13.20559200	-4.41402800	-5.75204600
H	-15.17056100	-4.54826500	-6.60193600
C	-13.07992700	-4.28654800	-3.34296900
H	-14.96408300	-4.31229900	-2.28848300
C	-12.44031400	-4.31749400	-4.58736000
H	-12.71886300	-4.44106400	-6.73067300
H	-12.49104200	-4.20744200	-2.42522800
H	-11.34943300	-4.27267000	-4.64760700

UB3LYP-D3/def2-SVP-SMD(THF)//UB3LYP/def2-SVP(gas)
HF= -4325.5388659
UPBEPBE-D3/def2-SVP-SMD(THF)//UB3LYP/def2-SVP(gas)
HF= -4322.3167620

^{3}D'

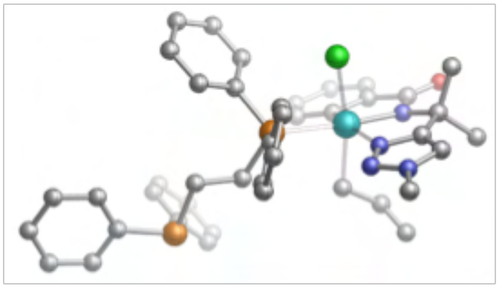

Zero-point correction= 0.753465 (Hartree/Particle)
Thermal correction to Energy= 0.803578
Thermal correction to Enthalpy= 0.804523
Thermal correction to Gibbs Free Energy= 0.663482
Sum of electronic and zero-point Energies= -4324.647146
Sum of electronic and thermal Energies= -4324.597032
Sum of electronic and thermal Enthalpies= -4324.596088
Sum of electronic and thermal Free Energies= -4324.737128

C	-19.90958700	-2.70062300	-3.50306800
C	-20.60465500	-1.66106900	-2.86376000
C	-21.96970800	-1.75789800	-2.57258700
C	-22.67213500	-2.90487600	-2.94582700
C	-22.00160500	-3.93947500	-3.60913200
C	-20.63086600	-3.83815700	-3.88713900
H	-22.45169400	-0.92121700	-2.06027600
H	-23.74017900	-2.99371800	-2.72987200
H	-22.54618100	-4.83669600	-3.91737100
H	-20.15186500	-4.66974000	-4.40836700
C	-19.82113100	-0.43712600	-2.51851900
O	-20.29480600	0.51570600	-1.90241000
C	-17.56405600	0.54317300	-2.70870100
C	-17.28749900	0.66831900	-1.18935400
H	-16.51806500	1.43568100	-1.00251600
H	-18.21384000	0.96358200	-0.67950000
H	-16.94546400	-0.29380300	-0.78482900
C	-18.02124500	1.90828400	-3.27665200
H	-18.95308700	2.21156800	-2.78479000
H	-17.25203300	2.67532500	-3.08708200
H	-18.19095900	1.83956100	-4.35992600
C	-16.30230000	0.11347700	-3.41458000
N	-16.22334100	-1.12312900	-3.97698000
N	-15.07209700	-1.30287100	-4.55745400
C	-15.09075100	0.72989800	-3.66197300
H	-14.69743600	1.70941400	-3.40675400
N	-14.37486400	-0.19005000	-4.36789600
N	-18.53973900	-0.53028100	-2.99247600
Fe	-17.97784100	-2.23708000	-3.64207200
Cl	-17.19784100	-2.93606200	-1.54889800
C	-18.24033600	0.29561800	-6.73776600
H	-17.21362000	0.13236300	-7.08299500
H	-18.70354100	1.25298700	-6.99156000

C	-18.90580500	-0.64471600	-6.04368300
H	-19.93379400	-0.43611500	-5.72536100
C	-18.37558400	-1.97134200	-5.70296300
H	-19.08241500	-2.77055500	-5.94521700
H	-17.38768400	-2.15494400	-6.14757300
C	-13.03196700	-0.07364900	-4.90883000
H	-12.33394300	0.23854700	-4.11905500
H	-12.74229000	-1.06197400	-5.28504700
H	-13.00733900	0.65920400	-5.72938000
P	-17.08294300	-4.51104900	-4.27254400
C	-17.66864600	-5.17283000	-5.92526400
H	-17.32643000	-4.44961100	-6.68228800
H	-18.76404400	-5.06880500	-5.90412100
C	-17.29791200	-6.60307800	-6.32692100
H	-17.75227900	-7.32564300	-5.63265400
H	-16.20702100	-6.75297300	-6.29258600
P	-17.82273100	-6.96031300	-8.09793700
C	-19.67174000	-6.97720000	-7.89962800
C	-20.40181000	-5.92575800	-8.47929700
C	-20.36817100	-7.96813800	-7.18455200
C	-21.79212700	-5.85520800	-8.33680700
H	-19.87497700	-5.15613700	-9.05070200
C	-21.75654100	-7.90163200	-7.04645500
H	-19.82203600	-8.80314200	-6.73769000
C	-22.47142300	-6.84363400	-7.62054400
H	-22.34443700	-5.02837700	-8.79083900
H	-22.28462600	-8.68019400	-6.48938600
H	-23.55800400	-6.79378600	-7.51218600
C	-17.44704500	-8.76758800	-8.26112000
C	-17.76616800	-9.37265800	-9.49265600
C	-16.80912700	-9.54879000	-7.28272400
C	-17.47805300	-10.71694000	-9.73025900
H	-18.24853200	-8.77995500	-10.27583700
C	-16.50804400	-10.89453300	-7.52583300
H	-16.54165700	-9.11949900	-6.31557900
C	-16.84391100	-11.48389100	-8.74603300
H	-17.74195600	-11.16686300	-10.69104500
H	-16.01107700	-11.48435800	-6.75087100
H	-16.61031900	-12.53516700	-8.93277800
C	-17.37311200	-5.92680700	-3.11441500
C	-18.45756800	-5.91736100	-2.22258000
C	-16.52278000	-7.04791000	-3.13430400
C	-18.70184600	-7.01645200	-1.39401200
H	-19.09813100	-5.04018100	-2.15152400
C	-16.76590800	-8.14193700	-2.29959900
H	-15.65264200	-7.06593400	-3.79282300
C	-17.86097700	-8.13131400	-1.43085000
H	-19.54893800	-6.98886000	-0.70416600
H	-16.09159900	-9.00199800	-2.32530200
H	-18.05019000	-8.98539600	-0.77527300
C	-15.24419800	-4.47690000	-4.43208700

C	-14.59634500	-4.48948900	-5.67779700
C	-14.46562000	-4.36539500	-3.26461700
C	-13.20176000	-4.41438600	-5.75698500
H	-15.16721700	-4.55505900	-6.60539600
C	-13.07457200	-4.28840400	-3.34751000
H	-14.95833900	-4.31754800	-2.29151500
C	-12.43616500	-4.31685700	-4.59260100
H	-12.71556900	-4.43869300	-6.73590800
H	-12.48505700	-4.20745300	-2.43037400
H	-11.34548500	-4.26864100	-4.65373900

UB3LYP-D3/def2-SVP-SMD(THF)//UB3LYP/def2-SVP(gas)
HF= -4325.5944946
UPBEPBE-D3/def2-SVP-SMD(THF)//UB3LYP/def2-SVP(gas)
HF= -4322.3558103

^{5}D'

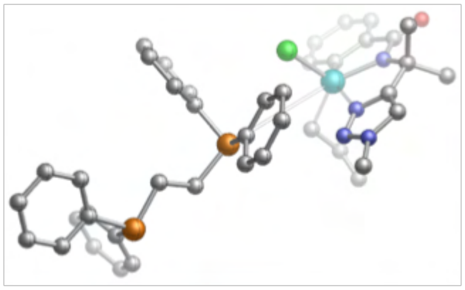

Zero-point correction=	0.750064 (Hartree/Particle)		
Thermal correction to Energy=	0.801850		
Thermal correction to Enthalpy=	0.802794		
Thermal correction to Gibbs Free Energy=	0.648646		
Sum of electronic and zero-point Energies=	-4324.648762		
Sum of electronic and thermal Energies=	-4324.596976		
Sum of electronic and thermal Enthalpies=	-4324.596032		
Sum of electronic and thermal Free Energies=	-4324.750180		
C	-20.68269300	-0.56641000	-1.85842200
C	-20.49626000	0.49159000	-0.95401000
C	-21.53456300	0.88000300	-0.10051400
C	-22.75905100	0.21031800	-0.14582400
C	-22.93949500	-0.85313800	-1.03756000
C	-21.90151800	-1.24557700	-1.89479700
H	-21.34704000	1.70510800	0.59155100
H	-23.57428500	0.51108200	0.51738100
H	-23.89395400	-1.38629800	-1.06905200
H	-22.06628800	-2.08646500	-2.57363400
C	-19.15858900	1.18051200	-0.90119000
O	-18.92597700	2.10514500	-0.12553800
C	-16.92744600	1.16061700	-1.95711700
C	-16.07016800	0.84312500	-0.70979100
H	-15.02911800	1.18002300	-0.84460900
H	-16.50007000	1.35858700	0.15976700
H	-16.07140200	-0.24041000	-0.51769300
C	-16.91760600	2.67827200	-2.25915300
H	-17.33335300	3.22595600	-1.40510200
H	-15.88932400	3.02738800	-2.44839600
H	-17.52894000	2.89314300	-3.14902500
C	-16.37349900	0.42966000	-3.16126900
N	-17.13501400	-0.50584700	-3.78997800
N	-16.51108000	-1.02257100	-4.81062500
C	-15.18160000	0.48146400	-3.86049000
H	-14.27777300	1.07003100	-3.73345700
N	-15.32692900	-0.43291600	-4.86089700
N	-18.29007600	0.63287500	-1.79987900
Fe	-18.91244100	-1.01587200	-2.73803900
Cl	-18.24058100	-2.96744000	-1.87419600
C	-19.69891200	0.47344500	-6.12102000
H	-18.75941000	0.06987900	-6.51124900
H	-20.07111000	1.40069500	-6.56445900

C	-20.37398900	-0.15074600	-5.13616100
H	-21.30750300	0.29619600	-4.77415100
C	-19.95703800	-1.39170400	-4.48246400
H	-20.76818700	-2.06067500	-4.17592400
H	-19.14613500	-1.94471900	-4.98294800
C	-14.38758600	-0.77765200	-5.91697000
H	-13.37285700	-0.82503300	-5.50161300
H	-14.66390800	-1.76518100	-6.30453200
H	-14.42574100	-0.03102600	-6.72452800
P	-17.43632800	-4.96368900	-5.49264300
C	-17.53372300	-5.98895100	-7.06883600
H	-17.09984100	-5.35564600	-7.86267700
H	-18.61192100	-6.03345300	-7.29583900
C	-16.93666900	-7.39904500	-7.09495000
H	-17.47649800	-8.05121200	-6.39108000
H	-15.88069300	-7.38264500	-6.77954300
P	-16.94920500	-8.12987000	-8.82573300
C	-18.77051600	-8.42467300	-9.06423500
C	-19.44568800	-7.63008200	-10.00593200
C	-19.50395500	-9.38468900	-8.34409000
C	-20.82118600	-7.77939100	-10.21574200
H	-18.88503600	-6.88961500	-10.58370200
C	-20.87595200	-9.53808600	-8.55583800
H	-18.99702400	-10.02352400	-7.61607300
C	-21.53829900	-8.73445900	-9.49119900
H	-21.33091900	-7.15173700	-10.95162600
H	-21.43204600	-10.28982600	-7.98918000
H	-22.61191800	-8.85711600	-9.65665600
C	-16.33348700	-9.85421600	-8.53325600
C	-16.24858300	-10.69968200	-9.65692200
C	-15.88285100	-10.34673000	-7.29668500
C	-15.75123900	-11.99848900	-9.54533900
H	-16.57839800	-10.33253300	-10.63356500
C	-15.37162400	-11.64574700	-7.18675100
H	-15.92830400	-9.72429800	-6.40132100
C	-15.30726200	-12.47650000	-8.30695800
H	-15.70177300	-12.63871200	-10.43022100
H	-15.02616000	-12.00866100	-6.21489800
H	-14.91004900	-13.49096200	-8.21850300
C	-17.87372100	-6.21215300	-4.19125500
C	-19.19354500	-6.19198500	-3.70964600
C	-16.98288100	-7.16633000	-3.66768900
C	-19.62219000	-7.11590500	-2.75209100
H	-19.88856400	-5.43337200	-4.07901700
C	-17.40701900	-8.08269200	-2.70163500
H	-15.94507000	-7.18771500	-4.00780400
C	-18.72929100	-8.06346800	-2.24541500
H	-20.65235900	-7.08273100	-2.38812400
H	-16.70028000	-8.81462600	-2.30105800
H	-19.05886500	-8.77906700	-1.48759500
C	-15.61694400	-4.73418000	-5.23010700

C	-14.65356000	-4.88276900	-6.24456000
C	-15.18960200	-4.25697300	-3.97317400
C	-13.30353400	-4.59528900	-6.00441800
H	-14.94299000	-5.23292200	-7.23754300
C	-13.84233000	-3.97937700	-3.73295700
H	-15.92262300	-4.09247600	-3.17810800
C	-12.89046100	-4.15082600	-4.74616700
H	-12.57255400	-4.73331800	-6.80601800
H	-13.53436700	-3.62614100	-2.74486800
H	-11.83458600	-3.94206400	-4.55388000

UB3LYP-D3/def2-SVP-SMD(THF)//UB3LYP/def2-SVP(gas)
HF= -4325.5731117
UPBEPBE-D3/def2-SVP-SMD(THF)//UB3LYP/def2-SVP(gas)
HF= -4322.3214474

^{1}D''

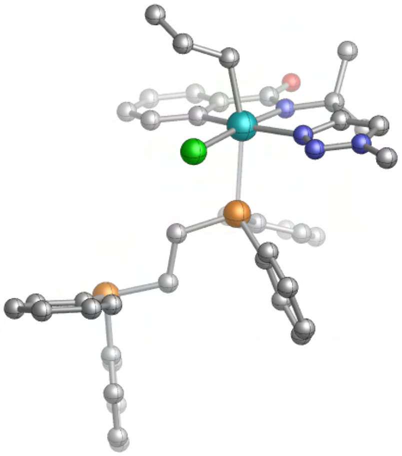

Zero-point correction= 0.752992 (Hartree/Particle)
Thermal correction to Energy= 0.803409
Thermal correction to Enthalpy= 0.804353
Thermal correction to Gibbs Free Energy= 0.661995
Sum of electronic and zero-point Energies= -4324.633418
Sum of electronic and thermal Energies= -4324.583000
Sum of electronic and thermal Enthalpies= -4324.582056
Sum of electronic and thermal Free Energies= -4324.724415

C	-18.32302300	0.89983200	-3.54767900
C	-19.18668100	1.94529400	-3.17567400
C	-20.57761300	1.81098200	-3.26319600
C	-21.11626800	0.61291600	-3.73649700
C	-20.26391900	-0.44082300	-4.10153100
C	-18.87302000	-0.30644400	-4.00122600
H	-21.20805900	2.64893100	-2.95476800
H	-22.19996000	0.48921400	-3.81352400
H	-20.68972400	-1.38285500	-4.45990700
H	-18.21755400	-1.13958600	-4.26421900
C	-18.53692900	3.18147900	-2.65007300
O	-19.15566000	4.14894000	-2.21275500
C	-16.30830100	4.13120600	-2.14203200
C	-16.51696900	4.26627600	-0.61248600
H	-15.85326100	5.04197800	-0.19716300
H	-17.55905000	4.54961700	-0.41518700
H	-16.30119500	3.31702900	-0.10053000
C	-16.54598900	5.49880000	-2.82284700
H	-17.57923400	5.82055600	-2.64675700
H	-15.85578400	6.25444700	-2.41301700
H	-16.38106000	5.42443900	-3.90584800
C	-14.89336700	3.68006700	-2.41486600
N	-14.67524800	2.46401700	-2.98923600
N	-13.39999100	2.23007700	-3.12351900
C	-13.64734200	4.23250600	-2.18800900
H	-13.32464800	5.17526300	-1.75586000
N	-12.77017700	3.29339000	-2.64393600
N	-17.16277900	3.06428200	-2.70570300

Fe	-16.45723700	1.41782000	-3.31274800
P	-16.60436900	2.03873600	-5.94618300
C	-17.30300600	0.73005900	-8.44412200
H	-17.94188700	1.58661100	-8.71507500
H	-16.31490700	0.92818600	-8.88792200
C	-17.22286300	0.57413000	-6.92455700
H	-16.54684700	-0.24140200	-6.62415100
H	-18.20330500	0.31057600	-6.49974700
C	-15.02470400	2.44551100	-6.83214300
C	-14.95712200	3.27897600	-7.96455000
C	-13.84634300	1.81898200	-6.38501600
C	-13.74097000	3.49695000	-8.61856200
H	-15.85821900	3.75922600	-8.34895000
C	-12.63371300	2.03372600	-7.04844700
H	-13.88277600	1.14519000	-5.52667700
C	-12.57424400	2.87713500	-8.16122300
H	-13.70990000	4.14752800	-9.49664400
H	-11.73047000	1.53261000	-6.68978200
H	-11.62476500	3.04529100	-8.67661200
C	-17.77033800	3.40211100	-6.37003800
C	-19.13911700	3.12765400	-6.54762400
C	-17.35528700	4.74707900	-6.39039200
C	-20.05500500	4.16120200	-6.76320000
H	-19.50725800	2.10111000	-6.50756200
C	-18.27119700	5.77875000	-6.60909100
H	-16.30261000	4.99614700	-6.24133700
C	-19.62550500	5.48968300	-6.79903200
H	-21.11282400	3.92162000	-6.89693900
H	-17.92359800	6.81504600	-6.62573200
H	-20.34290200	6.29682700	-6.96664800
Cl	-15.42250300	-0.47473900	-4.11077800
C	-11.31882800	3.35656900	-2.68448300
H	-10.98657300	4.08150100	-3.44248800
H	-10.92730200	3.64789200	-1.69977400
H	-10.95209100	2.35747500	-2.94747200
P	-18.12846100	-0.73906700	-9.28062800
C	-18.06264200	-0.14307900	-11.04179500
C	-19.19531400	0.53394100	-11.52892200
C	-16.96771900	-0.32418200	-11.90381700
C	-19.22642900	1.03572600	-12.83307800
H	-20.06625500	0.66247900	-10.87888300
C	-17.00248000	0.16761200	-13.21223500
H	-16.08109500	-0.85737600	-11.55299200
C	-18.12909200	0.85152000	-13.67961600
H	-20.11501000	1.56181700	-13.19240000
H	-16.14288700	0.01505700	-13.87072500
H	-18.15431400	1.23416100	-14.70336400
C	-16.81620300	-2.04757700	-9.25884800
C	-17.22550400	-3.35191400	-9.59526400
C	-15.47266400	-1.84336600	-8.90087300
C	-16.31844800	-4.41342000	-9.59739300

H	-18.27233400	-3.53550000	-9.85545700
C	-14.56599000	-2.90921900	-8.88732000
H	-15.11616500	-0.84873700	-8.62572100
C	-14.98389600	-4.19484000	-9.23957100
H	-16.65703900	-5.41727900	-9.86737400
H	-13.52724700	-2.73009500	-8.59751100
H	-14.27430800	-5.02624900	-9.22760900
C	-17.99504000	-1.41697100	-0.77415000
H	-18.82314700	-0.78819000	-0.43096600
H	-18.11880100	-2.49904000	-0.67516200
C	-16.87837500	-0.88699800	-1.30207900
H	-16.08645600	-1.55282000	-1.65659500
C	-16.63300000	0.55338500	-1.44051100
H	-15.59937800	0.82950800	-1.17823600
H	-17.36163600	1.16819600	-0.90235100

UB3LYP-D3/def2-SVP-SMD(THF)//UB3LYP/def2-SVP(gas)
HF= -4325.5462049
UPBEPBE-D3/def2-SVP-SMD(THF)//UB3LYP/def2-SVP(gas)
HF= -4322.3209152

^{3}D''

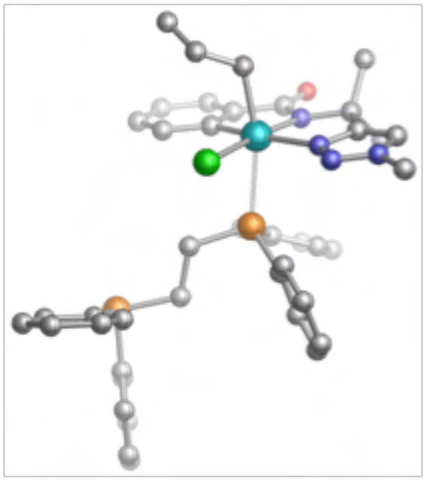

Zero-point correction= 0.752041 (Hartree/Particle)
Thermal correction to Energy= 0.802877
Thermal correction to Enthalpy= 0.803821
Thermal correction to Gibbs Free Energy= 0.658611
Sum of electronic and zero-point Energies= -4324.657643
Sum of electronic and thermal Energies= -4324.606808
Sum of electronic and thermal Enthalpies= -4324.605864
Sum of electronic and thermal Free Energies= -4324.751073

C	-18.32327200	0.82633100	-3.53275600
C	-19.20564100	1.84691500	-3.14226400
C	-20.59263900	1.68162200	-3.23574200
C	-21.10352900	0.47892700	-3.72887700
C	-20.22871000	-0.54913200	-4.11115200
C	-18.84055000	-0.38358300	-4.01006000
H	-21.24152200	2.50085000	-2.91512600
H	-22.18419500	0.33266400	-3.80930000
H	-20.63281100	-1.49396400	-4.48675700
H	-18.16510300	-1.19496700	-4.28983400
C	-18.58762000	3.09625200	-2.59922600
O	-19.23978000	4.03851300	-2.15395000
C	-16.39003800	4.11559100	-2.10580200
C	-16.58770400	4.26196900	-0.57602800
H	-15.95248800	5.06896800	-0.17569100
H	-17.63875300	4.50539100	-0.37252000
H	-16.32866900	3.32915200	-0.05368600
C	-16.67642700	5.46811600	-2.79866400
H	-17.71531700	5.76246200	-2.60755200
H	-16.00137300	6.24913600	-2.41140900
H	-16.53139100	5.38327300	-3.88390900
C	-14.96312900	3.70714900	-2.38817100
N	-14.70661500	2.49548700	-2.95646300
N	-13.42517900	2.30594800	-3.10238000
C	-13.73478000	4.30463200	-2.17823100
H	-13.44185700	5.26175200	-1.75659000
N	-12.82885800	3.39430000	-2.63614400

N	-17.21621100	3.02142900	-2.65613600
Fe	-16.45447300	1.37640000	-3.27609400
P	-16.55199400	2.06719300	-5.97831500
C	-17.29727000	0.75767100	-8.46808700
H	-17.92306600	1.62390600	-8.73890800
H	-16.30930800	0.93565200	-8.92077400
C	-17.20761100	0.61107000	-6.94827600
H	-16.54600000	-0.21671500	-6.64847200
H	-18.18948500	0.36869100	-6.51366000
C	-14.96324100	2.42966500	-6.86645800
C	-14.87346500	3.23337400	-8.01868500
C	-13.79755000	1.80001400	-6.39125700
C	-13.64851300	3.41741200	-8.66660800
H	-15.76429900	3.71775500	-8.42187600
C	-12.57591600	1.97998000	-7.04870300
H	-13.85081500	1.15107700	-5.51469100
C	-12.49493200	2.79283400	-8.18264600
H	-13.59976400	4.04508500	-9.56045400
H	-11.68276600	1.47663700	-6.66850500
H	-11.53855800	2.93431200	-8.69330700
C	-17.68462100	3.45591400	-6.41272800
C	-19.06286600	3.21585400	-6.56785500
C	-17.23469700	4.78923300	-6.45716000
C	-19.95430900	4.27022400	-6.78453700
H	-19.45688300	2.19964200	-6.50920100
C	-18.12655500	5.84171400	-6.67793800
H	-16.17404000	5.01268300	-6.32365600
C	-19.49054200	5.58644500	-6.84520400
H	-21.01990500	4.05716100	-6.90058000
H	-17.75203800	6.86815400	-6.71429900
H	-20.18872300	6.40991600	-7.01454200
Cl	-15.40636800	-0.50127500	-4.08754500
C	-11.38104200	3.50809600	-2.68961300
H	-11.08167500	4.25267600	-3.44217100
H	-10.98942000	3.80135200	-1.70542200
H	-10.98300800	2.52539900	-2.96830700
P	-18.15491100	-0.70168600	-9.28891400
C	-18.09307200	-0.11870800	-11.05459500
C	-19.21968600	0.57031900	-11.53888600
C	-17.00631800	-0.32010800	-11.92246000
C	-19.25264400	1.06380900	-12.84617800
H	-20.08444800	0.71497300	-10.88401800
C	-17.04303100	0.16344600	-13.23387500
H	-16.12462600	-0.86282000	-11.57377300
C	-18.16347100	0.85927500	-13.69854900
H	-20.13641700	1.59945900	-13.20323600
H	-16.18987800	-0.00495100	-13.89686800
H	-18.19028200	1.23544400	-14.72465500
C	-16.86542600	-2.03288000	-9.26945800
C	-17.29971200	-3.33174300	-9.59562100
C	-15.51595900	-1.85022500	-8.92225500

C	-16.41132100	-4.40891700	-9.59826700
H	-18.35142000	-3.49842700	-9.84741400
C	-14.62785800	-2.93164100	-8.90922300
H	-15.14021400	-0.86041200	-8.65544800
C	-15.07059200	-4.21170800	-9.25122600
H	-16.76926400	-5.40817100	-9.86029600
H	-13.58403400	-2.76900200	-8.62805300
H	-14.37548500	-5.05527500	-9.23978000
C	-17.95683900	-1.40658100	-0.69068800
H	-18.76152800	-0.74936800	-0.34558000
H	-18.12125900	-2.48386900	-0.60194100
C	-16.81622200	-0.91269000	-1.21124000
H	-16.05044800	-1.60477100	-1.57349000
C	-16.51918100	0.50953300	-1.33402400
H	-15.46489700	0.76176000	-1.15609400
H	-17.21039600	1.16663000	-0.79813300

UB3LYP-D3/def2-SVP-SMD(THF)//UB3LYP/def2-SVP(gas)
HF= -4325.5917637
UPBEPBE-D3/def2-SVP-SMD(THF)//UB3LYP/def2-SVP(gas)
HF= -4322.3550667

^{5}D''

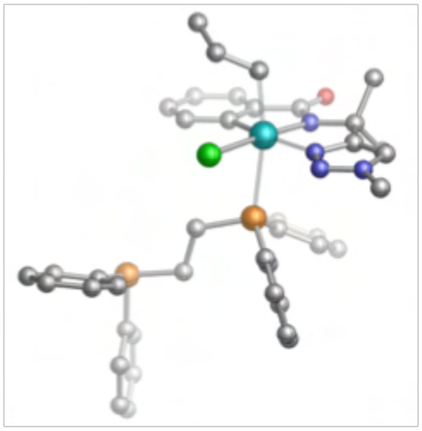

Zero-point correction= 0.751103 (Hartree/Particle)
Thermal correction to Energy= 0.802263
Thermal correction to Enthalpy= 0.803207
Thermal correction to Gibbs Free Energy= 0.656059
Sum of electronic and zero-point Energies= -4324.647270
Sum of electronic and thermal Energies= -4324.596111
Sum of electronic and thermal Enthalpies= -4324.595166
Sum of electronic and thermal Free Energies= -4324.742315

C	-18.46989300	1.18168800	-3.37481600
C	-19.05569100	2.40318900	-3.01761600
C	-20.44931400	2.54332900	-3.06030000
C	-21.24507800	1.47255600	-3.47365000
C	-20.64972400	0.25821700	-3.83936000
C	-19.25681700	0.10386600	-3.77921200
H	-20.87677400	3.50437800	-2.76404800
H	-22.33250000	1.57948300	-3.50972500
H	-21.27176900	-0.58196100	-4.16236600
H	-18.80020800	-0.85699200	-4.03241600
C	-18.17755100	3.54612200	-2.58049900
O	-18.66234400	4.60242100	-2.16396600
C	-15.85793600	4.20991400	-2.20229600
C	-16.03058000	4.45864600	-0.68162000
H	-15.23863500	5.12334600	-0.29927500
H	-17.00782100	4.92324200	-0.49916000
H	-15.98052200	3.50715100	-0.13026200
C	-15.88691500	5.55959300	-2.95950100
H	-16.88323900	6.00790900	-2.85850700
H	-15.13760800	6.25527100	-2.54690900
H	-15.65765000	5.41045900	-4.02549400
C	-14.51835100	3.54802700	-2.43324400
N	-14.45630700	2.28863400	-2.94195000
N	-13.21939200	1.88894900	-3.05503100
C	-13.21210700	3.94898800	-2.22066800
H	-12.77430700	4.86465000	-1.83375100
N	-12.45935700	2.88607800	-2.62109600
N	-16.85099400	3.25156300	-2.70440600
Fe	-16.42383200	1.34686100	-3.43123800

P	-16.89331500	1.98288600	-5.99467900
C	-17.24045700	0.60515000	-8.52284400
H	-17.85598700	1.45479400	-8.85999200
H	-16.21803900	0.81171600	-8.87630600
C	-17.28149400	0.46215000	-6.99846500
H	-16.56139100	-0.28731000	-6.63708600
H	-18.26664500	0.11338500	-6.64756400
C	-15.42128100	2.70357800	-6.85617200
C	-15.50666000	3.74431100	-7.79729700
C	-14.15868300	2.15145200	-6.56499600
C	-14.35614500	4.22506300	-8.43034300
H	-16.47490200	4.18362000	-8.04337200
C	-13.01398400	2.63006000	-7.20874600
H	-14.07481400	1.33916300	-5.83817700
C	-13.10718200	3.66962300	-8.13940400
H	-14.44076400	5.03428200	-9.16050900
H	-12.04219100	2.18554400	-6.97732100
H	-12.20983900	4.04442900	-8.63892000
C	-18.27363600	3.15200500	-6.33179200
C	-19.49163800	2.73582400	-6.89749100
C	-18.15781700	4.48722300	-5.90249400
C	-20.55265400	3.63416600	-7.04569400
H	-19.63285600	1.70375000	-7.22012100
C	-19.21757700	5.38281800	-6.05138800
H	-17.23496000	4.83008500	-5.43366800
C	-20.41960500	4.95946800	-6.62621100
H	-21.49093900	3.28906900	-7.48741500
H	-19.10692500	6.41192600	-5.70148000
H	-21.25226800	5.65853300	-6.73789700
Cl	-15.28192800	-0.50149200	-4.31575900
C	-11.01273000	2.75323000	-2.62997100
H	-10.56473700	3.48170700	-3.32182400
H	-10.60929000	2.91178600	-1.61941600
H	-10.77914900	1.73561500	-2.96454000
P	-17.98210700	-0.87506300	-9.41473200
C	-17.76038500	-0.30015700	-11.16962000
C	-18.85143400	0.35203300	-11.77161100
C	-16.58501500	-0.47349900	-11.92013600
C	-18.76503300	0.83759000	-13.07950200
H	-19.78255700	0.47376100	-11.20970500
C	-16.50162600	0.00215200	-13.23216100
H	-15.72816000	-0.98788400	-11.47882500
C	-17.58879000	0.66160000	-13.81428600
H	-19.62295500	1.34452000	-13.52939500
H	-15.58054900	-0.14355100	-13.80318800
H	-17.52144900	1.03175900	-14.84069300
C	-16.67869100	-2.18234900	-9.25954900
C	-17.05246800	-3.48782700	-9.63146800
C	-15.37800400	-1.97639600	-8.76932400
C	-16.14971100	-4.54885200	-9.53937600
H	-18.06777100	-3.67274300	-9.99513400

C	-14.47749300	-3.04205500	-8.66089300
H	-15.05080900	-0.98127600	-8.46185600
C	-14.85791900	-4.32868900	-9.04959300
H	-16.45932600	-5.55358600	-9.83921500
H	-13.47393800	-2.86185900	-8.26683900
H	-14.15327600	-5.15990200	-8.96342300
C	-18.23245200	-1.64445900	-1.08348400
H	-19.04821600	-1.03929800	-0.67578700
H	-18.37179400	-2.72903000	-1.09057400
C	-17.10222000	-1.08268800	-1.55428500
H	-16.32151100	-1.72421200	-1.97468900
C	-16.82911100	0.34983300	-1.56299100
H	-15.77461000	0.58995000	-1.35301700
H	-17.51564300	0.96807200	-0.97810900

UB3LYP-D3/def2-SVP-SMD(THF)//UB3LYP/def2-SVP(gas)
HF= -4325.5902992
UPBEPBE-D3/def2-SVP-SMD(THF)//UB3LYP/def2-SVP(gas)
HF= -4322.3353194

^{1}D'-complex

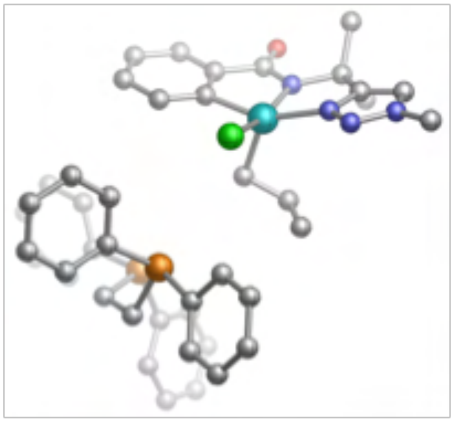

Zero-point correction= 0.751447 (Hartree/Particle)
Thermal correction to Energy= 0.802740
Thermal correction to Enthalpy= 0.803684
Thermal correction to Gibbs Free Energy= 0.652371
Sum of electronic and zero-point Energies= -4324.624896
Sum of electronic and thermal Energies= -4324.573603
Sum of electronic and thermal Enthalpies= -4324.572659
Sum of electronic and thermal Free Energies= -4324.723972

C	-18.68023900	-1.32356900	-4.59182700
C	-19.06956200	-0.01002800	-4.90198500
C	-20.34335900	0.27901500	-5.39917900
C	-21.25080700	-0.76670700	-5.59039300
C	-20.87314200	-2.08030300	-5.28261000
C	-19.59031900	-2.36583800	-4.78957100
H	-20.60274900	1.31875400	-5.61494100
H	-22.25407000	-0.56102300	-5.97313900
H	-21.58479600	-2.89845400	-5.42847900
H	-19.30797300	-3.39492900	-4.56483200
C	-18.06593300	1.05193100	-4.60414200
O	-18.23189800	2.24358000	-4.84131200
C	-15.98060800	1.39441200	-3.31890600
C	-16.66957500	2.35325100	-2.31520800
H	-15.91431600	2.94059100	-1.76863100
H	-17.33684000	3.03912400	-2.85035600
H	-17.26014700	1.77935700	-1.58493300
C	-15.15525400	2.20270400	-4.34527900
H	-15.83216600	2.84589300	-4.92254100
H	-14.40823400	2.83487900	-3.83778200
H	-14.62622900	1.53066600	-5.03704200
C	-15.09049500	0.45989700	-2.53781500
N	-15.35206900	-0.87585400	-2.57384200
N	-14.52239900	-1.54140200	-1.82207700
C	-14.00938500	0.62125800	-1.69354800
H	-13.45776400	1.49421400	-1.35670300
N	-13.70606400	-0.64469500	-1.28572200
N	-16.95974000	0.50243500	-3.97941800
Fe	-16.83895600	-1.37839700	-3.90430100

P	-16.49933000	-5.98033200	-6.54583700
P	-17.43894000	-3.95094300	-9.41739500
C	-16.44407100	-6.43784500	-8.35936700
H	-16.45096500	-7.53110900	-8.50269900
H	-15.46751500	-6.08634400	-8.73271500
C	-17.59346300	-5.80591300	-9.16643700
H	-18.55410000	-5.94770200	-8.64409500
H	-17.70796200	-6.31204700	-10.13979600
C	-17.88729100	-7.04410100	-5.89939400
C	-18.54653600	-6.58957700	-4.74286900
C	-18.30684700	-8.25328200	-6.47972500
C	-19.58920000	-7.33125600	-4.17748900
H	-18.23284000	-5.65163100	-4.27522800
C	-19.35771900	-8.98703000	-5.92207100
H	-17.81195200	-8.64082500	-7.37339000
C	-20.00097100	-8.52893900	-4.76775500
H	-20.08415700	-6.96435500	-3.27435100
H	-19.67206300	-9.92439700	-6.38936900
H	-20.82135800	-9.10500000	-4.33139000
C	-15.02465000	-6.88036200	-5.85748900
C	-14.68430000	-6.59771000	-4.51979200
C	-14.21642100	-7.77613900	-6.57726800
C	-13.58398500	-7.21166400	-3.91691500
H	-15.28589700	-5.88558600	-3.94591800
C	-13.10611300	-8.37963900	-5.97606500
H	-14.44479500	-8.01831200	-7.61713000
C	-12.78884600	-8.10406900	-4.64382300
H	-13.34290100	-6.98460200	-2.87488400
H	-12.48929200	-9.07349300	-6.55405700
H	-11.92351000	-8.57977900	-4.17470300
C	-16.24045700	-3.81594400	-10.82855400
C	-15.82808300	-2.51471600	-11.17628100
C	-15.68159400	-4.89649400	-11.53179400
C	-14.91093200	-2.29898200	-12.20596200
H	-16.23492100	-1.65910600	-10.62877900
C	-14.74935000	-4.68327300	-12.55441100
H	-15.97000700	-5.92187600	-11.29174500
C	-14.36447000	-3.38559800	-12.89807400
H	-14.61343000	-1.27890700	-12.46341900
H	-14.32576100	-5.53945700	-13.08655500
H	-13.63818600	-3.22023300	-13.69807200
C	-19.06341700	-3.62747800	-10.26060000
C	-19.31349800	-3.87977500	-11.62061400
C	-20.10115700	-3.10264500	-9.46984300
C	-20.57184200	-3.62256800	-12.17206200
H	-18.51766400	-4.27448800	-12.25711400
C	-21.36220700	-2.85342000	-10.02003000
H	-19.91908300	-2.88030200	-8.41418700
C	-21.59994500	-3.11148600	-11.37300300
H	-20.75010900	-3.82146500	-13.23245500
H	-22.15671600	-2.44526400	-9.38976800

H	-22.58284000	-2.90888600	-11.80666600
Cl	-16.96740000	-3.47927400	-3.12379200
C	-14.13046300	-2.54909400	-5.35578300
H	-14.46686300	-3.54235400	-5.04742500
H	-13.05584700	-2.34790100	-5.32680100
C	-15.00694200	-1.60496000	-5.75581600
H	-14.62809500	-0.61287400	-6.02782700
C	-16.45874400	-1.80287600	-5.83883400
H	-16.97942800	-1.10801800	-6.50154200
H	-16.78366300	-2.84101200	-5.96542900
C	-12.66008400	-1.07329600	-0.37195300
H	-11.67528600	-0.75801000	-0.74586100
H	-12.82718300	-0.64661200	0.62789100
H	-12.70204400	-2.16733600	-0.31648700

UB3LYP-D3/def2-SVP-SMD(THF)//UB3LYP/def2-SVP(gas)
HF= -4325.5236855
UPBEPBE-D3/def2-SVP-SMD(THF)//UB3LYP/def2-SVP(gas)
HF= -4322.2960107

^{3}D'-complex

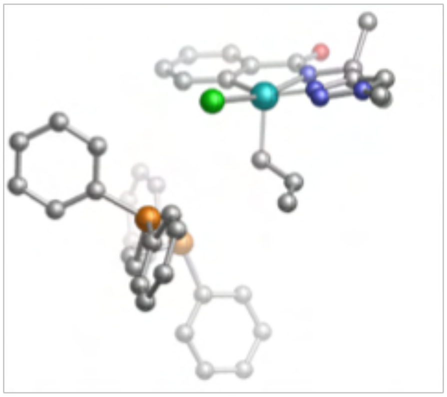

Zero-point correction=	0.750258 (Hartree/Particle)		
Thermal correction to Energy=	0.801897		
Thermal correction to Enthalpy=	0.802841		
Thermal correction to Gibbs Free Energy=	0.651210		
Sum of electronic and zero-point Energies=	-4324.650284		
Sum of electronic and thermal Energies=	-4324.598645		
Sum of electronic and thermal Enthalpies=	-4324.597701		
Sum of electronic and thermal Free Energies=	-4324.749332		
C	-18.75483800	-1.44733100	-4.51867900
C	-19.17749200	-0.15238100	-4.85954800
C	-20.44547100	0.08472600	-5.39677600
C	-21.30957400	-0.99574200	-5.59878400
C	-20.89808700	-2.29157100	-5.26079400
C	-19.62322200	-2.52421900	-4.72084100
H	-20.73380200	1.11129000	-5.63762700
H	-22.30771800	-0.83023200	-6.01348600
H	-21.57663800	-3.13554100	-5.41649300
H	-19.31467800	-3.53901000	-4.46672400
C	-18.21376900	0.95002000	-4.56326600
O	-18.43091800	2.13253100	-4.80755400
C	-16.07185600	1.38349600	-3.40858000
C	-16.70286700	2.35302900	-2.37748000
H	-15.92597600	2.98654800	-1.91963000
H	-17.44025300	2.99442300	-2.87479100
H	-17.20522700	1.78525700	-1.57957600
C	-15.35826900	2.18230700	-4.52306100
H	-16.09619500	2.79879200	-5.05227400
H	-14.58080800	2.83931900	-4.09988000
H	-14.88154200	1.50245800	-5.24491900
C	-15.08577300	0.50043700	-2.68321300
N	-15.29892800	-0.84390100	-2.65731000
N	-14.38113000	-1.45630000	-1.96495500
C	-13.94008800	0.72552600	-1.94466700
H	-13.39305800	1.62701300	-1.68415100
N	-13.55384300	-0.51569500	-1.53063800
N	-17.07927200	0.44931800	-3.95527300
Fe	-16.90821900	-1.44730900	-3.83159800
P	-16.55626500	-6.01620900	-6.49879400

P	-17.39306200	-3.90341800	-9.33751800
C	-16.50361000	-6.44611500	-8.31854400
H	-16.55122800	-7.53551200	-8.48194100
H	-15.51038600	-6.12683600	-8.67649100
C	-17.62054400	-5.75554500	-9.12340600
H	-18.59055400	-5.86939600	-8.61175300
H	-17.74575700	-6.23825900	-10.10729500
C	-17.96969700	-7.05524500	-5.86850700
C	-18.56323200	-6.64508900	-4.66036300
C	-18.47383400	-8.20007000	-6.50831500
C	-19.62071600	-7.37018300	-4.10209100
H	-18.18913200	-5.75307700	-4.14910200
C	-19.54056600	-8.91620500	-5.95685900
H	-18.03541100	-8.55128800	-7.44523600
C	-20.11555300	-8.50511200	-4.75053200
H	-20.06330200	-7.03868900	-3.15896800
H	-19.92087600	-9.80306400	-6.47132200
H	-20.94818500	-9.06729300	-4.31936400
C	-15.10303400	-6.95788000	-5.81913900
C	-14.71114000	-6.64428600	-4.50289900
C	-14.36369700	-7.92153900	-6.52538100
C	-13.62661700	-7.29277600	-3.90644200
H	-15.26001000	-5.88192700	-3.94079400
C	-13.26934900	-8.55975900	-5.93163300
H	-14.63548300	-8.19032300	-7.54825300
C	-12.90004400	-8.25163000	-4.61971200
H	-13.34443800	-7.04063400	-2.88064200
H	-12.70608200	-9.30638200	-6.49840500
H	-12.04722100	-8.75458200	-4.15610400
C	-16.18916900	-3.78721500	-10.74556200
C	-15.73969400	-2.49461900	-11.07954200
C	-15.65823300	-4.87600000	-11.45759100
C	-14.81372300	-2.29460400	-12.10439100
H	-16.12465900	-1.63314800	-10.52542400
C	-14.71710200	-4.67893400	-12.47536900
H	-15.97564500	-5.89527000	-11.22844800
C	-14.29527600	-3.38929500	-12.80539700
H	-14.48749800	-1.28072300	-12.35134100
H	-14.31581100	-5.54145700	-13.01450200
H	-13.56224800	-3.23647100	-13.60172300
C	-19.00269800	-3.49923500	-10.17368900
C	-19.26138400	-3.71413700	-11.53853500
C	-20.01966100	-2.95090800	-9.37196700
C	-20.50842100	-3.39697200	-12.08439200
H	-18.48119200	-4.12685600	-12.18294600
C	-21.26959700	-2.64156100	-9.91696300
H	-19.83104400	-2.75820600	-8.31170300
C	-21.51628800	-2.86244600	-11.27489200
H	-20.69371800	-3.56733100	-13.14854400
H	-22.04755900	-2.21579100	-9.27783400
H	-22.49023000	-2.61286000	-11.70424900

Cl	-16.91357600	-3.54819800	-3.04252200
C	-13.91637300	-2.70045100	-5.40114400
H	-14.25751500	-3.70683100	-5.14456000
H	-12.83885200	-2.51493000	-5.39372000
C	-14.79441500	-1.71956000	-5.71669800
H	-14.40679000	-0.71798400	-5.93798900
C	-16.23099400	-1.89894600	-5.78674300
H	-16.78194300	-1.17173000	-6.38750300
H	-16.59365400	-2.92831800	-5.87659500
C	-12.41362800	-0.87854800	-0.70537800
H	-12.50389100	-0.42466200	0.29234700
H	-12.41045600	-1.97094400	-0.61353100
H	-11.47932200	-0.54299700	-1.17815900

UB3LYP-D3/def2-SVP-SMD(THF)//UB3LYP/def2-SVP(gas)
HF= -4325.5770566
UPBEPBE-D3/def2-SVP-SMD(THF)//UB3LYP/def2-SVP(gas)
HF= -4322.3311663

^{5}D'-complex

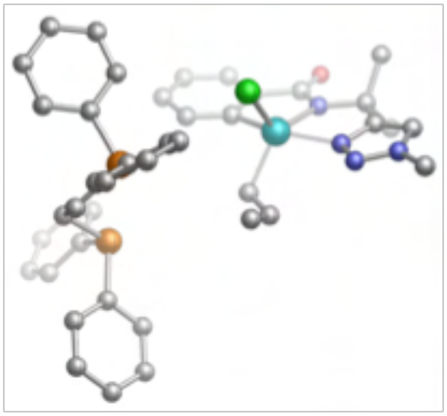

Zero-point correction= 0.750037 (Hartree/Particle)
Thermal correction to Energy= 0.801863
Thermal correction to Enthalpy= 0.802808
Thermal correction to Gibbs Free Energy= 0.649223
Sum of electronic and zero-point Energies= -4324.644671
Sum of electronic and thermal Energies= -4324.592845
Sum of electronic and thermal Enthalpies= -4324.591901
Sum of electronic and thermal Free Energies= -4324.745485

C	-18.43406700	-1.36777400	-4.90926100
C	-19.19255100	-0.30898700	-4.40431100
C	-20.57641000	-0.27937600	-4.62133100
C	-21.19188200	-1.31767100	-5.32182300
C	-20.42509800	-2.38864100	-5.79897200
C	-19.03689000	-2.41673600	-5.60156800
H	-21.13980300	0.56248900	-4.21057700
H	-22.27217900	-1.30267400	-5.48830500
H	-20.90595800	-3.21536400	-6.32945300
H	-18.45225500	-3.25724700	-5.98233900
C	-18.51517900	0.74283300	-3.57315000
O	-19.12093500	1.72466700	-3.14525600
C	-16.39414700	1.30449100	-2.48820300
C	-16.94009600	1.34033700	-1.04140300
H	-16.28986600	1.94698400	-0.38955500
H	-17.94614400	1.77855400	-1.04463700
H	-16.99658800	0.32064100	-0.63128200
C	-16.28445700	2.73917700	-3.06274300
H	-17.28100200	3.19680900	-3.09044200
H	-15.61755000	3.35945200	-2.44172800
H	-15.87902100	2.70864000	-4.08597400
C	-15.01813300	0.68165700	-2.48574300
N	-14.75380300	-0.37800200	-3.29530700
N	-13.51949800	-0.77684900	-3.16400300
C	-13.84509400	0.95148300	-1.80518400
H	-13.57915400	1.69494600	-1.05937500
N	-12.96078600	0.02224000	-2.26545700
N	-17.20423200	0.43120500	-3.35007400
Fe	-16.51477500	-1.26160000	-4.21987100

P	-16.65992800	-5.97114900	-6.55388600
P	-17.40370500	-4.09281100	-9.54374200
C	-16.71600500	-6.61097400	-8.31169500
H	-16.89326100	-7.69876400	-8.34261800
H	-15.70164600	-6.45655000	-8.71597800
C	-17.76255300	-5.90303700	-9.19028700
H	-18.74817000	-5.90815700	-8.69543100
H	-17.90331400	-6.45251700	-10.13657100
C	-18.15811900	-6.75547800	-5.77237100
C	-18.63992400	-6.15777000	-4.59271200
C	-18.82936300	-7.88294300	-6.27550900
C	-19.75292600	-6.68373000	-3.92963100
H	-18.13904700	-5.27450800	-4.18547600
C	-19.94990500	-8.40029900	-5.61860600
H	-18.48165800	-8.37592700	-7.18624800
C	-20.41340700	-7.80383800	-4.44207300
H	-20.10802100	-6.20707700	-3.01223000
H	-20.46046100	-9.27681600	-6.02712100
H	-21.28886300	-8.21008000	-3.92850000
C	-15.29152100	-6.98788300	-5.80954700
C	-14.73623500	-6.51047600	-4.60726100
C	-14.77201700	-8.16925400	-6.36581800
C	-13.70363000	-7.20648500	-3.97258800
H	-15.11518000	-5.58424700	-4.16427500
C	-13.72950700	-8.85700700	-5.73657400
H	-15.17828000	-8.57056100	-7.29669100
C	-13.19474300	-8.38030100	-4.53635800
H	-13.29099000	-6.82304600	-3.03554300
H	-13.33706400	-9.77362500	-6.18569500
H	-12.38218100	-8.92117300	-4.04392600
C	-16.05760600	-4.17383900	-10.81991600
C	-15.40112700	-2.96315100	-11.11234100
C	-15.62743300	-5.33723600	-11.48099000
C	-14.36825000	-2.91090000	-12.05036700
H	-15.70541200	-2.04983100	-10.59254200
C	-14.58247700	-5.28980600	-12.41091000
H	-16.10668800	-6.29758100	-11.27943100
C	-13.95299800	-4.07713400	-12.70185200
H	-13.87817100	-1.95778200	-12.26634100
H	-14.26204400	-6.20742200	-12.91176900
H	-13.13783100	-4.04110500	-13.42920200
C	-18.89616000	-3.69272800	-10.57912800
C	-19.04769500	-4.06642900	-11.92565900
C	-19.93430500	-2.97585600	-9.95836500
C	-20.21159700	-3.74153900	-12.62790500
H	-18.24746500	-4.60953300	-12.43443500
C	-21.10235600	-2.65760400	-10.65851200
H	-19.82388200	-2.65670800	-8.91792500
C	-21.24300000	-3.03918300	-11.99559300
H	-20.31287400	-4.03693800	-13.67583900
H	-21.89936400	-2.10035600	-10.15908900

H	-22.15179200	-2.78420600	-12.54713000
Cl	-16.42393600	-3.23190400	-3.12766200
C	-14.16759000	-2.59692800	-6.12616800
H	-14.74376700	-3.52457200	-6.19839100
H	-13.08467400	-2.67873600	-6.00722300
C	-14.77627500	-1.38937900	-6.17127300
H	-14.16069000	-0.48949900	-6.06329800
C	-16.22140400	-1.20528600	-6.29645700
H	-16.56040500	-0.21721400	-6.62665000
H	-16.75054400	-2.02446600	-6.79124400
C	-11.57160600	-0.17066100	-1.88445400
H	-11.49929900	-0.39676900	-0.81071800
H	-11.18655400	-1.01747400	-2.46441800
H	-10.98434900	0.73136000	-2.11007800

UB3LYP-D3/def2-SVP-SMD(THF)//UB3LYP/def2-SVP(gas)
HF= -4325.5689308
UPBEPBE-D3/def2-SVP-SMD(THF)//UB3LYP/def2-SVP(gas)
HF= -4322.3170791

^{1}D'-noligand

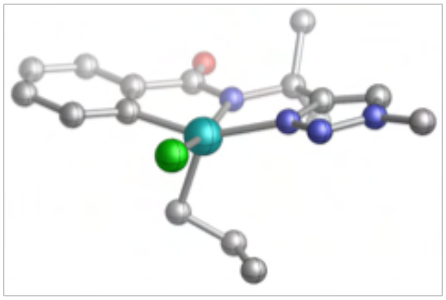

Zero-point correction=		0.329128 (Hartree/Particle)	
Thermal correction to Energy=		0.352520	
Thermal correction to Enthalpy=		0.353464	
Thermal correction to Gibbs Free Energy=		0.276532	
Sum of electronic and zero-point Energies=		-2638.016339	
Sum of electronic and thermal Energies=		-2637.992947	
Sum of electronic and thermal Enthalpies=		-2637.992003	
Sum of electronic and thermal Free Energies=		-2638.068935	
C	-18.52365300	-1.40400900	-4.58791500
C	-19.13226600	-0.13847900	-4.56007400
C	-20.48709800	0.02711500	-4.86048400
C	-21.25010000	-1.09598300	-5.19308300
C	-20.65244100	-2.36262000	-5.21781400
C	-19.29033200	-2.52412800	-4.92188000
H	-20.92047200	1.02966700	-4.81515000
H	-22.31219800	-0.98836100	-5.42873300
H	-21.25292700	-3.24030300	-5.47495000
H	-18.83023100	-3.51362500	-4.95129900
C	-18.26048200	0.98948400	-4.12212600
O	-18.61929300	2.16053300	-4.06993900
C	-16.09839500	1.39012800	-2.98429100
C	-16.78375400	2.00037200	-1.73599900
H	-16.05403600	2.57434400	-1.14257700
H	-17.59734700	2.66775900	-2.04372900
H	-17.19860800	1.20275600	-1.10109100
C	-15.52145800	2.51176000	-3.87728500
H	-16.34429100	3.14518900	-4.23363200
H	-14.80890000	3.13570700	-3.31332500
H	-14.99686000	2.08678900	-4.74591300
C	-15.00060800	0.46767700	-2.51446000
N	-15.07182300	-0.85270100	-2.84010000
N	-14.07428000	-1.52010500	-2.33373600
C	-13.86292000	0.61692800	-1.74570800
H	-13.40557500	1.47394700	-1.25973400
N	-13.33753600	-0.63988500	-1.67044900
N	-17.01902400	0.50984100	-3.73738900
Fe	-16.62252900	-1.29262500	-4.11794600
Cl	-16.31810300	-3.49577100	-3.95170500
C	-13.97400100	-1.71925300	-6.04590200
H	-14.16566000	-2.78178500	-5.87015300
H	-12.93174800	-1.39264300	-6.09916300

C	-14.98819300	-0.83874100	-6.16040400
H	-14.75232300	0.22615600	-6.27420400
C	-16.41542600	-1.19198800	-6.11895600
H	-17.08441900	-0.43275200	-6.53181200
H	-16.65795500	-2.20206700	-6.46066900
C	-12.14305800	-1.07921300	-0.96789800
H	-12.25456900	-0.91700600	0.11416900
H	-12.02063400	-2.14998900	-1.16846900
H	-11.26335700	-0.52980200	-1.33291400

UB3LYP-D3/def2-SVP-SMD(THF)//UB3LYP/def2-SVP(gas)
HF= -2638.4047957
UPBEPBE-D3/def2-SVP-SMD(THF)//UB3LYP/def2-SVP(gas)
HF= -2636.7975212

^{3}D'-noligand

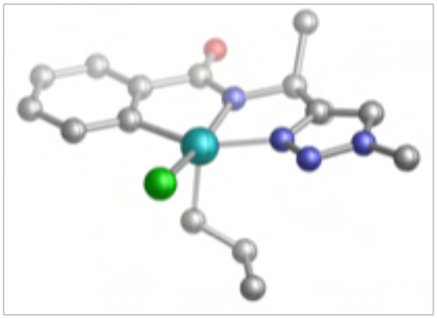

Zero-point correction= 0.327682 (Hartree/Particle)
Thermal correction to Energy= 0.351548
Thermal correction to Enthalpy= 0.352492
Thermal correction to Gibbs Free Energy= 0.273283
Sum of electronic and zero-point Energies= -2638.041801
Sum of electronic and thermal Energies= -2638.017935
Sum of electronic and thermal Enthalpies= -2638.016991
Sum of electronic and thermal Free Energies= -2638.096200

C	-18.62406800	-1.39983800	-4.54224400
C	-19.21534400	-0.12752100	-4.51667200
C	-20.54860100	0.06403700	-4.88863500
C	-21.30497900	-1.03960600	-5.29442800
C	-20.72413300	-2.31371200	-5.31803400
C	-19.38467700	-2.50231400	-4.94325200
H	-20.97028600	1.07166900	-4.84439700
H	-22.34992100	-0.91055000	-5.58891400
H	-21.31985600	-3.17577800	-5.63270000
H	-18.93797500	-3.49825300	-4.96832500
C	-18.35037000	0.98195600	-4.01600300
O	-18.70868300	2.15282700	-3.94548100
C	-16.16319000	1.38941500	-2.93708400
C	-16.78669400	2.01492000	-1.66442200
H	-16.03587400	2.61243600	-1.12236300
H	-17.62629400	2.66300800	-1.94247600
H	-17.15446000	1.22420800	-0.99294900
C	-15.63963500	2.49964200	-3.87708100
H	-16.48102500	3.12891300	-4.19470200
H	-14.89475100	3.13021300	-3.36465400
H	-15.16716900	2.06152400	-4.76921600
C	-15.02939300	0.48706400	-2.51518600
N	-15.08964300	-0.83540200	-2.83121800
N	-14.05564200	-1.48086800	-2.37151300
C	-13.85844000	0.66247000	-1.80322100
H	-13.39501900	1.53081700	-1.34385500
N	-13.30431100	-0.58299600	-1.74905900
N	-17.11856300	0.49304400	-3.62302300
Fe	-16.72882200	-1.33939300	-4.00855800
Cl	-16.40496900	-3.54015900	-3.91688400
C	-13.84165500	-1.95776600	-6.04462900
H	-14.14541600	-3.00508200	-5.95943900

H	-12.77102100	-1.74013900	-6.08214500
C	-14.76166300	-0.96542300	-6.06468400
H	-14.41325400	0.07452600	-6.08142700
C	-16.19798900	-1.17234600	-6.05363200
H	-16.81305400	-0.32995700	-6.37939500
H	-16.55575100	-2.14676000	-6.39593300
C	-12.06726100	-0.99448600	-1.10595200
H	-12.12541800	-0.82386600	-0.02102400
H	-11.93606300	-2.06483800	-1.30326900
H	-11.21755100	-0.43331800	-1.52080900

UB3LYP-D3/def2-SVP-SMD(THF)//UB3LYP/def2-SVP(gas)
HF= -2638.4577780
UPBEPBE-D3/def2-SVP-SMD(THF)//UB3LYP/def2-SVP(gas)
HF= -2636.8864254

[5]D'-noligand

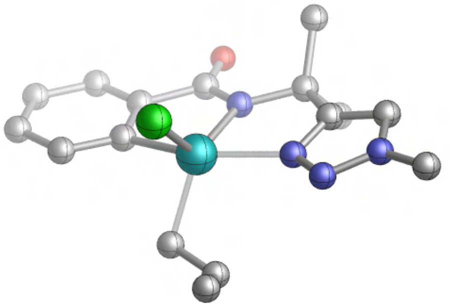

Zero-point correction= 0.327141 (Hartree/Particle)
Thermal correction to Energy= 0.351271
Thermal correction to Enthalpy= 0.352215
Thermal correction to Gibbs Free Energy= 0.270872
Sum of electronic and zero-point Energies= -2638.034717
Sum of electronic and thermal Energies= -2638.010588
Sum of electronic and thermal Enthalpies= -2638.009643
Sum of electronic and thermal Free Energies= -2638.090987

C	-18.52953400	-1.22816900	-4.87460800
C	-19.14197700	-0.00304800	-4.60740800
C	-20.48735900	0.18882700	-4.94751700
C	-21.21003600	-0.85165500	-5.53321600
C	-20.59319800	-2.08822600	-5.76355100
C	-19.24489300	-2.28457500	-5.43475200
H	-20.93854300	1.15859800	-4.72173400
H	-22.26045500	-0.70840000	-5.79932300
H	-21.16334700	-2.91208100	-6.20219200
H	-18.77794500	-3.25787400	-5.60177000
C	-18.35902600	1.05379700	-3.88325700
O	-18.82459100	2.16867900	-3.65323800
C	-16.24705600	1.45230600	-2.71011300
C	-16.87107000	1.79070400	-1.33611600
H	-16.17489100	2.38692300	-0.72337100
H	-17.79175600	2.36808200	-1.48808200
H	-17.11581000	0.86649200	-0.79100200
C	-15.87266300	2.74961400	-3.47017600
H	-16.77879300	3.34191200	-3.64736000
H	-15.15468600	3.35037400	-2.88826700
H	-15.41438100	2.50275000	-4.44039700
C	-14.99164500	0.63924300	-2.50122900
N	-14.84583400	-0.55247700	-3.13916700
N	-13.70362300	-1.10874300	-2.84767700
C	-13.83947400	0.82491200	-1.75980500
H	-13.50879000	1.61875100	-1.09635200
N	-13.08658700	-0.28233800	-2.01479000
N	-17.13057300	0.58729800	-3.50754000
Fe	-16.63952300	-1.28143900	-4.10911000
Cl	-16.83038600	-3.20155500	-2.98646100
C	-14.26345600	-2.99592600	-5.66363400
H	-14.88096900	-3.88958000	-5.53419800

H	-13.18757600	-3.10180700	-5.50828200
C	-14.81449400	-1.79851000	-5.96561900
H	-14.15578800	-0.92422900	-6.02865700
C	-16.24532600	-1.56510900	-6.15400300
H	-16.52162600	-0.65018100	-6.68842900
H	-16.81950700	-2.43499800	-6.48539200
C	-11.77592500	-0.62512400	-1.48812100
H	-11.81495800	-0.70288800	-0.39194000
H	-11.49380800	-1.59468100	-1.91477200
H	-11.03700200	0.13666900	-1.77626000

UB3LYP-D3/def2-SVP-SMD(THF)//UB3LYP/def2-SVP(gas)
HF= -2638.4471480
UPBEPBE-D3/def2-SVP-SMD(THF)//UB3LYP/def2-SVP(gas)
HF= -2636.8702054

^{1}D'-TS

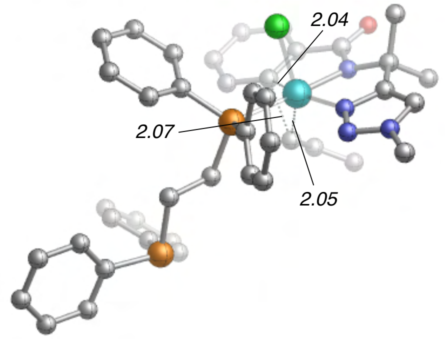

Zero-point correction= 0.751916 (Hartree/Particle)
Thermal correction to Energy= 0.801631
Thermal correction to Enthalpy= 0.802576
Thermal correction to Gibbs Free Energy= 0.663055
Sum of electronic and zero-point Energies= -4324.603474
Sum of electronic and thermal Energies= -4324.553758
Sum of electronic and thermal Enthalpies= -4324.552814
Sum of electronic and thermal Free Energies= -4324.692334

C	-19.99135700	-2.59530900	-3.49262900
C	-20.55769200	-1.50176100	-2.80890600
C	-21.81941200	-1.59537300	-2.21786700
C	-22.55410700	-2.77960100	-2.32129300
C	-22.01692200	-3.86266200	-3.02688000
C	-20.75279300	-3.76706200	-3.62168900
H	-22.19543100	-0.72301800	-1.67757100
H	-23.54387600	-2.85812800	-1.86424800
H	-22.58782900	-4.79025300	-3.12697800
H	-20.37817200	-4.61968600	-4.19406500
C	-19.74177900	-0.25589600	-2.68669100
O	-20.17543100	0.76364800	-2.14517900
C	-17.47584200	0.61666900	-2.97596000
C	-17.21838700	0.83520700	-1.46281100
H	-16.40759600	1.56716600	-1.31236800
H	-18.13420000	1.21499000	-0.99222600
H	-16.94128800	-0.11723100	-0.99004600
C	-17.85735700	1.95539900	-3.65075500
H	-18.79505000	2.32127800	-3.21549800
H	-17.06858400	2.70932500	-3.49060700
H	-17.99738200	1.82153100	-4.73391900
C	-16.21657600	0.08496300	-3.60818900
N	-16.16021100	-1.20719000	-4.03721100
N	-14.99685200	-1.47075300	-4.56489400
C	-14.98354800	0.64343700	-3.88550500
H	-14.56934300	1.63332800	-3.71854300
N	-14.27547700	-0.36234100	-4.47200100
N	-18.49551200	-0.42468600	-3.21418300
Fe	-17.99919600	-2.21150800	-3.73388300
Cl	-17.49130900	-2.83251000	-1.53460800
C	-19.94423300	-0.02150500	-6.40775800

H	-18.91197200	0.32755200	-6.50826400
H	-20.73110800	0.64197100	-6.77580000
C	-20.23531900	-1.20412300	-5.85137400
H	-21.28262100	-1.51250600	-5.76524200
C	-19.23401300	-2.18584800	-5.37153000
H	-19.45003200	-3.18432900	-5.76152300
H	-18.21242600	-1.88131900	-5.71719900
C	-12.91686300	-0.33564800	-4.98526000
H	-12.23283900	0.05131000	-4.21668500
H	-12.64042800	-1.36632500	-5.23755400
H	-12.85408000	0.29800200	-5.88287300
P	-17.09890200	-4.51974000	-4.25435800
C	-17.59102100	-5.16868800	-5.94499300
H	-17.21357700	-4.42435600	-6.66784000
H	-18.68767000	-5.06903700	-5.97217200
C	-17.21457100	-6.59062700	-6.37130900
H	-17.71594800	-7.32592900	-5.72433000
H	-16.13003800	-6.75915400	-6.28238200
P	-17.64627300	-6.88937200	-8.17759600
C	-19.50378600	-6.86731100	-8.08535000
C	-20.17920300	-5.80601700	-8.71127400
C	-20.25918400	-7.84046400	-7.40650300
C	-21.57416200	-5.70910400	-8.65041200
H	-19.60541200	-5.05053700	-9.25577100
C	-21.65155800	-7.74678000	-7.34824700
H	-19.75575700	-8.68327700	-6.92531700
C	-22.31205300	-6.67957200	-7.96849100
H	-22.08394500	-4.87617300	-9.14170800
H	-22.22551300	-8.51132000	-6.81782000
H	-23.40186000	-6.60854900	-7.92247100
C	-17.30507400	-8.70083600	-8.36870200
C	-17.59643300	-9.27407600	-9.62217900
C	-16.71294800	-9.51421100	-7.38779100
C	-17.32586800	-10.61838600	-9.87916700
H	-18.04322200	-8.65654500	-10.40726700
C	-16.42954600	-10.86024700	-7.64988800
H	-16.46757500	-9.11020000	-6.40407100
C	-16.73755400	-11.41769100	-8.89217300
H	-17.56776000	-11.04322300	-10.85701700
H	-15.96839000	-11.47531300	-6.87243400
H	-16.51790400	-12.46920700	-9.09381400
C	-17.47062800	-5.93969600	-3.11913900
C	-18.53617400	-5.88404000	-2.20743200
C	-16.68096500	-7.10484400	-3.16442700
C	-18.82786400	-6.98155900	-1.39076900
H	-19.11564500	-4.96934500	-2.10376600
C	-16.97205900	-8.19758000	-2.34448000
H	-15.81673600	-7.15689700	-3.82880100
C	-18.05322900	-8.14124000	-1.45936600
H	-19.65914400	-6.91604600	-0.68429200
H	-16.34512200	-9.09193600	-2.39181400

H	-18.27964000	-8.99419500	-0.81406100
C	-15.25279500	-4.54151400	-4.27275200
C	-14.49605700	-4.64235200	-5.44985800
C	-14.58268300	-4.38639800	-3.04488800
C	-13.09786000	-4.61585700	-5.40175800
H	-14.98260300	-4.73483400	-6.42235900
C	-13.18818100	-4.35609000	-3.00067600
H	-15.16377900	-4.26122300	-2.12794800
C	-12.43957500	-4.47697100	-4.17726700
H	-12.52426700	-4.71015700	-6.32790100
H	-12.68264300	-4.23799300	-2.03851700
H	-11.34682400	-4.46649000	-4.13829900

UB3LYP-D3/def2-SVP-SMD(THF)//UB3LYP/def2-SVP(gas)
HF= -4325.5341468
UPBEPBE-D3/def2-SVP-SMD(THF)//UB3LYP/def2-SVP(gas)
HF= -4322.2648794

^{3}D'-TS

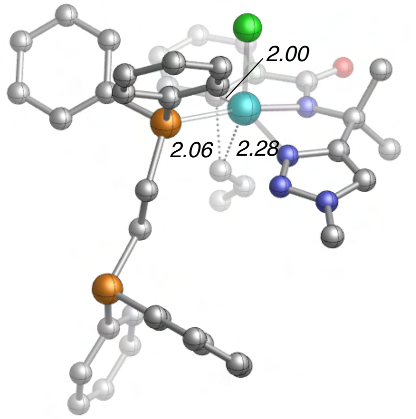

Zero-point correction= 0.752646 (Hartree/Particle)
Thermal correction to Energy= 0.802519
Thermal correction to Enthalpy= 0.803463
Thermal correction to Gibbs Free Energy= 0.663007
Sum of electronic and zero-point Energies= -4324.625517
Sum of electronic and thermal Energies= -4324.575644
Sum of electronic and thermal Enthalpies= -4324.574700
Sum of electronic and thermal Free Energies= -4324.715156

C	-20.17848800	-2.65499100	-4.72541200
C	-20.67581200	-1.49926200	-4.07586900
C	-22.00008000	-1.43358900	-3.63353300
C	-22.86042000	-2.51157500	-3.84105200
C	-22.38255100	-3.65876500	-4.49098900
C	-21.06189700	-3.72873000	-4.93798200
H	-22.32212400	-0.52448800	-3.11965000
H	-23.89836400	-2.46448900	-3.50198700
H	-23.04851400	-4.51015200	-4.65797900
H	-20.72968200	-4.62750500	-5.46067400
C	-19.72866900	-0.37400800	-3.81863200
O	-20.07045000	0.71081800	-3.34891000
C	-17.31329100	0.16184500	-3.96723400
C	-17.05172400	0.41377100	-2.46317300
H	-16.15566900	1.04012100	-2.31987800
H	-17.92083200	0.93067300	-2.03425800
H	-16.91712500	-0.54258100	-1.93837200
C	-17.50776200	1.50295700	-4.71278700
H	-18.38825200	2.01687400	-4.30893200
H	-16.62295200	2.14780200	-4.58295300
H	-17.65801600	1.33230100	-5.79019900
C	-16.14278400	-0.57291100	-4.57960400
N	-16.29688900	-1.86756800	-4.97344200
N	-15.19874700	-2.32915100	-5.50875200
C	-14.84209600	-0.22536800	-4.89436300
H	-14.27559500	0.69314300	-4.77241600
N	-14.31245500	-1.34355200	-5.46645400
N	-18.46078700	-0.74058600	-4.17218400
Fe	-18.20088600	-2.60550000	-4.41552100

P	-17.43433300	-4.98373100	-4.92608200
C	-16.61404300	-5.35240600	-6.57604500
H	-16.36402400	-6.42629700	-6.56994000
H	-15.65782700	-4.81125400	-6.50786700
C	-18.70292900	-6.30833000	-4.71218400
C	-19.39445200	-6.34616400	-3.48592100
C	-19.00885600	-7.26279700	-5.69630000
C	-20.36206600	-7.32430700	-3.25330700
H	-19.17284800	-5.59897500	-2.71905600
C	-19.98876100	-8.23511900	-5.46131000
H	-18.48560600	-7.26925400	-6.65398100
C	-20.66532400	-8.26925300	-4.24029800
H	-20.88814100	-7.34294800	-2.29552100
H	-20.21675600	-8.96989800	-6.23785600
H	-21.42804500	-9.03050400	-4.05692700
C	-16.05544900	-5.61628000	-3.84785500
C	-15.24625300	-4.74888800	-3.09864300
C	-15.76967500	-6.99304800	-3.82932000
C	-14.16799400	-5.24779700	-2.36141200
H	-15.48477300	-3.68853000	-3.05618400
C	-14.69225200	-7.48937000	-3.09116700
H	-16.39910100	-7.69279300	-4.38412400
C	-13.88530100	-6.61590500	-2.35661300
H	-13.55519200	-4.55986600	-1.77281300
H	-14.48950800	-8.56353600	-3.08524900
H	-13.04596700	-7.00287000	-1.77269100
Cl	-17.98878800	-3.19903500	-2.17796300
C	-20.96046600	-1.40395000	-7.88042800
H	-21.34733100	-2.38691000	-8.16944700
H	-21.47497800	-0.52866700	-8.28566000
C	-19.91357700	-1.27561500	-7.05247500
H	-19.57403600	-0.27255500	-6.77410200
C	-19.13763200	-2.40284000	-6.48838900
H	-19.44519800	-3.36835100	-6.89749400
H	-18.07178100	-2.24123300	-6.69096900
P	-16.51501000	-5.75942300	-9.36677900
C	-17.49172600	-5.05148600	-10.77338300
C	-17.23912600	-5.59588400	-12.04747300
C	-18.48496300	-4.06345800	-10.65655400
C	-17.93899600	-5.15306600	-13.17110000
H	-16.48478900	-6.38089100	-12.15742200
C	-19.19625700	-3.62858900	-11.78106900
H	-18.71778000	-3.61425100	-9.68943800
C	-18.92377200	-4.16772800	-13.04031000
H	-17.72267600	-5.58621500	-14.15125600
H	-19.96518900	-2.86010100	-11.66625100
H	-19.47981400	-3.82591900	-13.91705500
C	-17.33399800	-4.96517000	-7.87177100
H	-17.35967400	-3.87293600	-7.98480700
H	-18.37680500	-5.32154300	-7.88164800
C	-14.93962100	-4.76982100	-9.45726600

C	-13.75825000	-5.38537900	-9.00859900
C	-14.86699000	-3.45442200	-9.94898500
C	-12.53748400	-4.70227800	-9.03551300
H	-13.79388700	-6.41558900	-8.64301800
C	-13.64712000	-2.77363800	-9.98580100
H	-15.76942100	-2.96082900	-10.31819500
C	-12.47885700	-3.39519600	-9.52826600
H	-11.62863300	-5.19946600	-8.68604000
H	-13.60647100	-1.75561700	-10.38311600
H	-11.52261500	-2.86646000	-9.57088600
C	-12.95893200	-1.56180500	-5.95123300
H	-12.99602500	-2.28702400	-6.77389100
H	-12.54873900	-0.61145300	-6.31684200
H	-12.31962100	-1.95237300	-5.14504400

UB3LYP-D3/def2-SVP-SMD(THF)//UB3LYP/def2-SVP(gas)
HF= -4325.5723948
UPBEPBE-D3/def2-SVP-SMD(THF)//UB3LYP/def2-SVP(gas)
HF= -4322.3313352

^{5}D'-TS

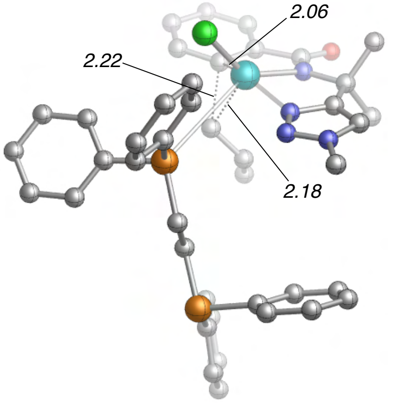

Zero-point correction= 0.750095 (Hartree/Particle)
Thermal correction to Energy= 0.801153
Thermal correction to Enthalpy= 0.802097
Thermal correction to Gibbs Free Energy= 0.653119
Sum of electronic and zero-point Energies= -4324.643845
Sum of electronic and thermal Energies= -4324.592787
Sum of electronic and thermal Enthalpies= -4324.591843
Sum of electronic and thermal Free Energies= -4324.740821

C	-20.75104500	-1.79507700	-3.99647800
C	-20.97862300	-0.40768000	-3.98187500
C	-22.23145500	0.07795100	-3.58069700
C	-23.23090000	-0.80055600	-3.16298800
C	-22.97548000	-2.17748400	-3.12669700
C	-21.74027900	-2.67750200	-3.54991200
H	-22.37402500	1.16156300	-3.58196000
H	-24.20456300	-0.41577700	-2.84925300
H	-23.74552800	-2.87124100	-2.77844000
H	-21.55703200	-3.75495400	-3.53554900
C	-19.85960200	0.56954000	-4.25980400
O	-20.07874700	1.77065700	-4.41093600
C	-17.41533500	0.68826000	-4.54289100
C	-17.04869600	1.62012300	-3.36559800
H	-16.09688500	2.14517500	-3.55034700
H	-17.84489600	2.36721800	-3.24298800
H	-16.95707000	1.04352000	-2.43268600
C	-17.49732400	1.50120700	-5.85912800
H	-18.27272300	2.27106400	-5.76852300
H	-16.52910300	1.98153200	-6.07497300
H	-17.75486500	0.84356300	-6.70310100
C	-16.35353800	-0.38170700	-4.71464400
N	-16.69278300	-1.70101900	-4.67187400
N	-15.64748800	-2.46100100	-4.85731600
C	-14.99040100	-0.34219700	-4.94725600
H	-14.28527400	0.47656300	-5.05647100
N	-14.61449700	-1.64942500	-5.02449400
N	-18.64920100	-0.05324100	-4.25747400
Fe	-18.70860300	-2.02008900	-3.88358400

P	-17.08831600	-5.84186400	-5.20731200
C	-16.22744600	-6.09121800	-6.85296300
H	-15.93096900	-7.14145100	-7.01304400
H	-15.29123700	-5.51647200	-6.75795800
C	-18.30908600	-7.23613200	-5.11931100
C	-19.22526600	-7.18052600	-4.05019100
C	-18.41199000	-8.29570300	-6.03537800
C	-20.19796500	-8.16940800	-3.89054500
H	-19.16999800	-6.34965800	-3.33912300
C	-19.39692500	-9.27881100	-5.88274200
H	-17.72232000	-8.36900100	-6.87967400
C	-20.28866800	-9.22145300	-4.80905700
H	-20.89368800	-8.11465800	-3.04889500
H	-19.46323600	-10.09510200	-6.60727500
H	-21.05541500	-9.99139700	-4.68982800
C	-15.76703400	-6.43872100	-4.04287600
C	-15.37973100	-5.58749000	-2.99438100
C	-15.14143600	-7.69206300	-4.16603100
C	-14.37816000	-5.97842300	-2.09732100
H	-15.88306900	-4.62615300	-2.86956500
C	-14.13925100	-8.07878600	-3.27337800
H	-15.44657300	-8.38186100	-4.95758200
C	-13.75338500	-7.22009100	-2.23744100
H	-14.09430100	-5.31094300	-1.27903500
H	-13.66285100	-9.05717300	-3.38069900
H	-12.97331800	-7.52566600	-1.53481500
Cl	-18.38076900	-3.37624100	-2.12956200
C	-20.35923300	-1.60172800	-7.64708600
H	-21.41714100	-1.85154100	-7.51787000
H	-20.09622800	-0.97575900	-8.50375200
C	-19.42398900	-2.03249100	-6.78303800
H	-18.38155100	-1.73801100	-6.94301500
C	-19.70016900	-2.88547900	-5.61657600
H	-20.66469900	-3.39057800	-5.66745400
H	-18.91142400	-3.63911200	-5.41645000
P	-16.15692500	-5.78542800	-9.67142100
C	-17.33047700	-5.00807700	-10.87730300
C	-17.00949800	-5.14010000	-12.24227500
C	-18.52649600	-4.35358200	-10.53510500
C	-17.84293900	-4.61669600	-13.23203800
H	-16.09359400	-5.66450900	-12.53128900
C	-19.36925500	-3.84029700	-11.52820700
H	-18.81669500	-4.22997700	-9.49018100
C	-19.02973900	-3.96590300	-12.87708000
H	-17.57097300	-4.72637900	-14.28523000
H	-20.29562800	-3.33641900	-11.23975700
H	-19.68911300	-3.56350000	-13.65042600
C	-17.04928300	-5.56235500	-8.03472000
H	-17.30889800	-4.50368500	-7.86964100
H	-17.99754300	-6.11869800	-8.12561600
C	-14.84595100	-4.46832500	-9.52433500

C	-13.52914300	-4.88515300	-9.26279000
C	-15.09798300	-3.09190100	-9.66529600
C	-12.49221100	-3.95433500	-9.13381600
H	-13.31266700	-5.95337300	-9.17121300
C	-14.06310700	-2.16046400	-9.54449100
H	-16.11073800	-2.74491400	-9.88533500
C	-12.75663700	-2.58902300	-9.27844400
H	-11.47339000	-4.29907500	-8.93712300
H	-14.27519200	-1.09499600	-9.66993500
H	-11.94462800	-1.86063200	-9.20103500
C	-13.29815200	-2.20258400	-5.30582500
H	-13.13584400	-2.27580600	-6.39185700
H	-12.53035800	-1.55944300	-4.85592200
H	-13.24957000	-3.20394700	-4.86092400

UB3LYP-D3/def2-SVP-SMD(THF)//UB3LYP/def2-SVP(gas)
HF= -4325.5714470
UPBEPBE-D3/def2-SVP-SMD(THF)//UB3LYP/def2-SVP(gas)
HF= -4322.3126639

[1]D’-complex-TS

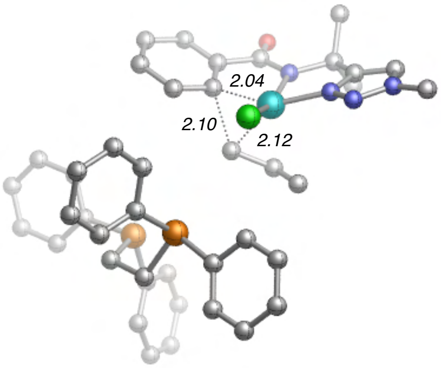

Zero-point correction= 0.752072 (Hartree/Particle)
Thermal correction to Energy= 0.802332
Thermal correction to Enthalpy= 0.803276
Thermal correction to Gibbs Free Energy= 0.655655
Sum of electronic and zero-point Energies= -4324.621971
Sum of electronic and thermal Energies= -4324.571711
Sum of electronic and thermal Enthalpies= -4324.570767
Sum of electronic and thermal Free Energies= -4324.718388

C	-18.36766400	-1.28886700	-4.89033000
C	-18.76150900	0.05451900	-5.04070300
C	-20.07875200	0.38664400	-5.36697600
C	-21.02811900	-0.62602500	-5.53060000
C	-20.64874900	-1.96241900	-5.35485100
C	-19.32309000	-2.29874500	-5.04924700
H	-20.33732400	1.44447500	-5.46154500
H	-22.06250400	-0.37636700	-5.78069400
H	-21.39040900	-2.75944700	-5.46056900
H	-19.03781400	-3.34516400	-4.93698400
C	-17.75767600	1.10744900	-4.69380600
O	-17.91736700	2.30312800	-4.92807100
C	-15.79602000	1.41075500	-3.23159000
C	-16.55906600	2.39011500	-2.30591200
H	-15.85066700	2.94948200	-1.67352900
H	-17.13951300	3.09895200	-2.90793400
H	-17.24787900	1.83513400	-1.65083600
C	-14.83390900	2.19258800	-4.15445400
H	-15.42523700	2.84720400	-4.80899400
H	-14.13501800	2.81201200	-3.56837200
H	-14.24465400	1.50336400	-4.77817400
C	-15.03463500	0.44052500	-2.36133200
N	-15.29309300	-0.89275900	-2.48626000
N	-14.58430200	-1.59431100	-1.64770900
C	-14.08994200	0.55776200	-1.36133700
H	-13.59375800	1.41307900	-0.91183100
N	-13.85343100	-0.72515500	-0.96284500
N	-16.70937100	0.54663000	-4.00733200
Fe	-16.55160200	-1.36354600	-3.96274300
P	-16.52003100	-5.94781800	-6.61807800

P	-17.58964500	-4.02207400	-9.51423600
C	-16.48509200	-6.45063700	-8.41995900
H	-16.46130200	-7.54717900	-8.53367500
H	-15.52548500	-6.08120300	-8.81891200
C	-17.66409400	-5.87678600	-9.22717400
H	-18.61460100	-6.04769000	-8.69514600
H	-17.76525300	-6.40374300	-10.19099400
C	-17.87599100	-7.01876400	-5.91870900
C	-18.49866900	-6.56388100	-4.74177200
C	-18.30879100	-8.23139700	-6.48182600
C	-19.51800800	-7.30955800	-4.14047000
H	-18.17493800	-5.62354600	-4.28512600
C	-19.33672000	-8.96895600	-5.88737000
H	-17.84280400	-8.61876600	-7.39089000
C	-19.94317700	-8.51107500	-4.71336500
H	-19.98418300	-6.94241800	-3.22227000
H	-19.66178900	-9.90902100	-6.34174100
H	-20.74558400	-9.09001400	-4.24831300
C	-15.01342500	-6.79661300	-5.93432400
C	-14.66484500	-6.48657000	-4.60476400
C	-14.18876000	-7.67751000	-6.65378300
C	-13.53896600	-7.06065500	-4.00982500
H	-15.27905500	-5.78534000	-4.03061000
C	-13.05319700	-8.24033500	-6.06018800
H	-14.42292700	-7.94006300	-7.68733800
C	-12.72692200	-7.93838500	-4.73599500
H	-13.29152100	-6.81319100	-2.97393300
H	-12.42388000	-8.92313100	-6.63788900
H	-11.84180700	-8.38232500	-4.27261500
C	-16.38793100	-3.86052800	-10.91987000
C	-16.01924000	-2.54972700	-11.27999300
C	-15.79045400	-4.92880300	-11.61008800
C	-15.10632300	-2.31348400	-12.30904600
H	-16.45725700	-1.70269700	-10.74326600
C	-14.86268400	-4.69450400	-12.63206700
H	-16.04477700	-5.96083300	-11.36019000
C	-14.52098900	-3.38800500	-12.98796700
H	-14.84286100	-1.28662200	-12.57631000
H	-14.40838500	-5.54113900	-13.15400900
H	-13.79799500	-3.20631000	-13.78737900
C	-19.22074500	-3.78942500	-10.37485800
C	-19.45132600	-4.09674000	-11.72698900
C	-20.28409200	-3.28093500	-9.60802300
C	-20.71521800	-3.90951800	-12.29353600
H	-18.63588500	-4.47942900	-12.34577500
C	-21.55058300	-3.10155100	-10.17294000
H	-20.11739000	-3.01592300	-8.55968800
C	-21.76852100	-3.41432700	-11.51760800
H	-20.87811200	-4.15059300	-13.34760500
H	-22.36581300	-2.70555600	-9.56162900
H	-22.75591700	-3.26662400	-11.96300000

Cl	-16.85685400	-3.48338500	-3.16813100
C	-14.37771400	-2.21407700	-5.17816800
H	-14.60626500	-3.21288700	-4.79992800
H	-13.36655700	-1.82968000	-5.01816500
C	-15.29656500	-1.48142600	-5.86004600
H	-15.01384900	-0.48971000	-6.22744500
C	-16.69673700	-1.87535700	-6.01758000
H	-17.21090300	-1.35462900	-6.82551700
H	-16.88394300	-2.95373400	-6.02242600
C	-12.96147600	-1.19717400	0.08297000
H	-11.93357500	-0.85910700	-0.11238100
H	-13.29039200	-0.82435500	1.06415900
H	-12.99892400	-2.29276800	0.07577500

UB3LYP-D3/def2-SVP-SMD(THF)//UB3LYP/def2-SVP(gas)
HF= -4325.5256437
UPBEPBE-D3/def2-SVP-SMD(THF)//UB3LYP/def2-SVP(gas)
HF= -4322.2890126

[3]D'-complex-TS

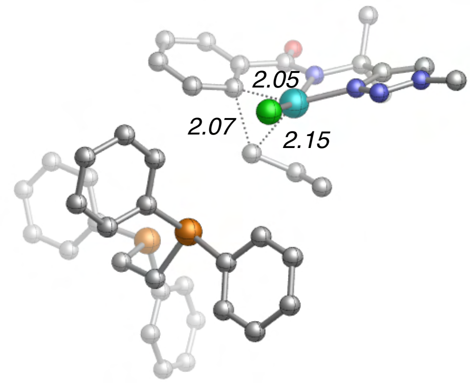

Zero-point correction= 0.751998 (Hartree/Particle)
Thermal correction to Energy= 0.802279
Thermal correction to Enthalpy= 0.803223
Thermal correction to Gibbs Free Energy= 0.654735
Sum of electronic and zero-point Energies= -4324.638097
Sum of electronic and thermal Energies= -4324.587816
Sum of electronic and thermal Enthalpies= -4324.586871
Sum of electronic and thermal Free Energies= -4324.735360

C	-18.32833800	-1.30678700	-4.90432400
C	-18.71476100	0.03778000	-5.07465000
C	-20.02834000	0.37072500	-5.41260800
C	-20.98282300	-0.63964600	-5.56319200
C	-20.61340700	-1.97547200	-5.36146100
C	-19.29131800	-2.31401900	-5.04592500
H	-20.28109400	1.42817800	-5.52527900
H	-22.01461800	-0.38801400	-5.82194000
H	-21.36030100	-2.76914000	-5.45428800
H	-19.01189000	-3.35997500	-4.91483100
C	-17.71081900	1.09399200	-4.73116500
O	-17.86333300	2.28519600	-4.99330600
C	-15.76792800	1.41627000	-3.24689100
C	-16.53396100	2.42623600	-2.35712500
H	-15.82910500	2.99621900	-1.73023800
H	-17.10034500	3.12334300	-2.98571600
H	-17.23637100	1.89470700	-1.69702800
C	-14.78407500	2.16534600	-4.17389500
H	-15.35935700	2.80799000	-4.85422700
H	-14.08857000	2.79396200	-3.59355000
H	-14.19193800	1.45485700	-4.77033100
C	-15.02859600	0.46135100	-2.34033900
N	-15.29852700	-0.87314100	-2.42902700
N	-14.60691900	-1.55631500	-1.56139100
C	-14.09448000	0.59840600	-1.33300700
H	-13.59436600	1.46166800	-0.90349500
N	-13.87557400	-0.67441400	-0.89382700
N	-16.67998100	0.54410400	-4.01483000
Fe	-16.53646900	-1.37218100	-3.91825900
P	-16.52803100	-5.96570400	-6.62597000

P	-17.61377000	-4.02079200	-9.50541300
C	-16.51498500	-6.46159100	-8.43003500
H	-16.50100300	-7.55784600	-8.54803900
H	-15.55600900	-6.09854000	-8.83629400
C	-17.69657100	-5.87626800	-9.22507600
H	-18.64402200	-6.04365600	-8.68648200
H	-17.80853300	-6.39827600	-10.19040800
C	-17.88394000	-7.03003000	-5.91624500
C	-18.48346700	-6.58008400	-4.72539500
C	-18.33879600	-8.23226300	-6.48411800
C	-19.50115500	-7.32063200	-4.11517900
H	-18.14317800	-5.64732600	-4.26526200
C	-19.36526100	-8.96461400	-5.88061600
H	-17.89156200	-8.61558300	-7.40423100
C	-19.94819400	-8.51196200	-4.69281400
H	-19.94900100	-6.95751600	-3.18631500
H	-19.70764200	-9.89657600	-6.33894700
H	-20.74941500	-9.08684200	-4.22074900
C	-15.01995800	-6.82740200	-5.96186900
C	-14.64563300	-6.51196600	-4.64076000
C	-14.21886900	-7.72439200	-6.68800400
C	-13.51769900	-7.09630800	-4.05978000
H	-15.24153600	-5.79870800	-4.06226000
C	-13.08105800	-8.29747200	-6.10882800
H	-14.47356500	-7.99158000	-7.71552300
C	-12.72918300	-7.98985200	-4.79247000
H	-13.24991600	-6.84434400	-3.03005500
H	-12.47010900	-8.99263200	-6.69147200
H	-11.84226300	-8.44169200	-4.34031200
C	-16.41492200	-3.85975800	-10.91349900
C	-16.03435800	-2.54978900	-11.26399100
C	-15.83118300	-4.92821400	-11.61514300
C	-15.12306400	-2.31411600	-12.29465800
H	-16.46154600	-1.70296500	-10.71830300
C	-14.90509800	-4.69471200	-12.63878500
H	-16.09512700	-5.95967400	-11.37294600
C	-14.55143900	-3.38874000	-12.98492400
H	-14.85006600	-1.28776800	-12.55419300
H	-14.46146300	-5.54143900	-13.16967000
H	-13.82966800	-3.20765000	-13.78557900
C	-19.24633000	-3.77793500	-10.36060400
C	-19.48054200	-4.07297000	-11.71482200
C	-20.30692500	-3.27367600	-9.58714900
C	-20.74525000	-3.87802000	-12.27699700
H	-18.66734200	-4.45204500	-12.33872600
C	-21.57423200	-3.08674500	-10.14770000
H	-20.13729700	-3.01774700	-8.53702700
C	-21.79581000	-3.38734800	-11.49456700
H	-20.91087000	-4.10949800	-13.33279000
H	-22.38728500	-2.69421900	-9.53126000
H	-22.78382300	-3.23358300	-11.93652100

Cl	-16.80461500	-3.50334100	-3.14395400
C	-14.33565400	-2.16880500	-5.17660500
H	-14.53178500	-3.15988500	-4.76091200
H	-13.33103400	-1.75560500	-5.05071100
C	-15.28263900	-1.48312100	-5.86392900
H	-15.03207900	-0.49543700	-6.26407700
C	-16.67747900	-1.91622400	-5.99833000
H	-17.19637800	-1.44490100	-6.83307100
H	-16.83687200	-2.99855000	-5.96456200
C	-12.99909300	-1.12390900	0.17475300
H	-11.96479200	-0.80726800	-0.02282300
H	-13.33057100	-0.71381600	1.14006300
H	-13.05223200	-2.21850300	0.20351300

UB3LYP-D3/def2-SVP-SMD(THF)//UB3LYP/def2-SVP(gas)
HF= -4325.5651717
UPBEPBE-D3/def2-SVP-SMD(THF)//UB3LYP/def2-SVP(gas)
HF= -4322.3230129

^{5}D'-complex-TS

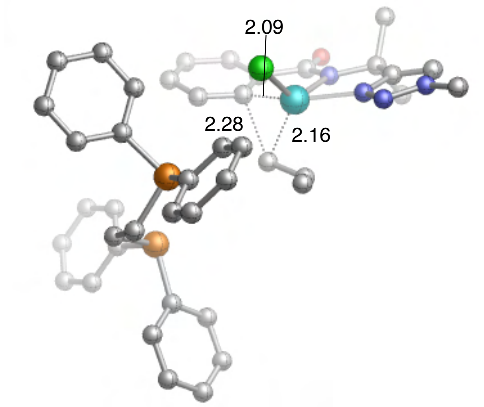

Zero-point correction= 0.750124 (Hartree/Particle)
Thermal correction to Energy= 0.801071
Thermal correction to Enthalpy= 0.802015
Thermal correction to Gibbs Free Energy= 0.651502
Sum of electronic and zero-point Energies= -4324.643482
Sum of electronic and thermal Energies= -4324.592535
Sum of electronic and thermal Enthalpies= -4324.591591
Sum of electronic and thermal Free Energies= -4324.742104

C	-18.48060500	-1.30761100	-4.98698500
C	-19.06397900	-0.07517100	-4.67330800
C	-20.44116200	0.10558600	-4.85795800
C	-21.23014300	-0.94628100	-5.32650500
C	-20.64137600	-2.18655400	-5.60256900
C	-19.26063400	-2.36925900	-5.44646100
H	-20.86363800	1.07985400	-4.59915200
H	-22.30546500	-0.80716800	-5.46485600
H	-21.25525700	-3.02321700	-5.94814600
H	-18.81104100	-3.33623300	-5.68128300
C	-18.23636700	1.00326700	-4.02956700
O	-18.67514000	2.14423800	-3.87643300
C	-16.15583800	1.40756000	-2.81937900
C	-16.83862800	1.85604700	-1.50471800
H	-16.14891700	2.45857800	-0.89088000
H	-17.72465600	2.45877700	-1.73950600
H	-17.14986400	0.97693900	-0.92009300
C	-15.69312100	2.64186200	-3.63237900
H	-16.56859200	3.24648200	-3.90061800
H	-14.99243100	3.25988000	-3.04707900
H	-15.18526600	2.32309500	-4.55614300
C	-14.94744400	0.56593000	-2.47618300
N	-14.83258700	-0.69033800	-2.97843700
N	-13.72778600	-1.25712600	-2.58537000
C	-13.81749100	0.78306700	-1.70778900
H	-13.47779300	1.62822100	-1.11605000
N	-13.10457500	-0.37329900	-1.81356600
N	-17.02756600	0.52652000	-3.61215600
Fe	-16.57471500	-1.38859100	-4.13735600

P	-16.59830400	-5.99013400	-6.55461600
P	-17.47809800	-4.08054700	-9.49488400
C	-16.58075700	-6.56615400	-8.33504600
H	-16.65968900	-7.66363000	-8.40560300
H	-15.58209900	-6.30655400	-8.72449600
C	-17.68312700	-5.92310800	-9.19536300
H	-18.66401000	-6.02544600	-8.70198300
H	-17.77825300	-6.45402300	-10.15768700
C	-18.05714200	-6.90202100	-5.83918800
C	-18.64291100	-6.35391100	-4.68317700
C	-18.60103100	-8.08360800	-6.37149600
C	-19.73564400	-6.97802100	-4.07268600
H	-18.23665700	-5.43504600	-4.25023900
C	-19.70092700	-8.69968600	-5.76761600
H	-18.16768600	-8.54182700	-7.26356700
C	-20.27042200	-8.14960800	-4.61492600
H	-20.17226400	-6.53880600	-3.17188100
H	-20.11191900	-9.61703100	-6.19813100
H	-21.12960800	-8.63333400	-4.14267800
C	-15.18079300	-6.94020500	-5.81484300
C	-14.74885100	-6.53118600	-4.53821500
C	-14.50521300	-8.00023300	-6.44292100
C	-13.68636200	-7.17913800	-3.90293100
H	-15.24653700	-5.69471400	-4.03733900
C	-13.43278900	-8.63858800	-5.81095400
H	-14.80973700	-8.34483000	-7.43327800
C	-13.02284800	-8.23375000	-4.53790700
H	-13.37182000	-6.85095700	-2.90861000
H	-12.91872300	-9.46068400	-6.31672300
H	-12.18699400	-8.73639900	-4.04402700
C	-16.16712600	-4.00888900	-10.80706500
C	-15.65900800	-2.73191100	-11.11393500
C	-15.62340100	-5.11688200	-11.47916200
C	-14.66104200	-2.56437700	-12.07557200
H	-16.05395400	-1.85727000	-10.58838600
C	-14.61200300	-4.95269100	-12.43292900
H	-15.98617900	-6.12501500	-11.26841500
C	-14.13088700	-3.67727000	-12.73737500
H	-14.28830700	-1.56204500	-12.30284800
H	-14.20168300	-5.82899000	-12.94211800
H	-13.34232300	-3.55019000	-13.48356000
C	-19.02486200	-3.76364400	-10.47618700
C	-19.18107000	-4.09730100	-11.83271500
C	-20.10048600	-3.15854900	-9.80248800
C	-20.38567300	-3.84219200	-12.49384200
H	-18.35322800	-4.55368800	-12.38104800
C	-21.30842100	-2.91045400	-10.46202200
H	-19.98906700	-2.87129400	-8.75279300
C	-21.45312100	-3.25102500	-11.80972300
H	-20.49079000	-4.10494100	-13.55004600
H	-22.13422200	-2.43946700	-9.92228500

H	-22.39384600	-3.05025000	-12.32915700
Cl	-16.75264300	-3.38692600	-3.10158800
C	-14.43642600	-2.48329400	-5.77620900
H	-14.88451100	-3.48145500	-5.78869500
H	-13.37461600	-2.40557700	-5.53220500
C	-15.17853400	-1.37981500	-6.05108500
H	-14.69437300	-0.39660400	-6.02148500
C	-16.61925700	-1.41533600	-6.30133100
H	-17.03546400	-0.53572200	-6.79682600
H	-16.99287300	-2.36124700	-6.70379600
C	-11.83558400	-0.71486900	-1.19444000
H	-11.93065300	-0.71196900	-0.09864800
H	-11.56457400	-1.72100400	-1.53532700
H	-11.05729700	0.00107900	-1.49632800

UB3LYP-D3/def2-SVP-SMD(THF)//UB3LYP/def2-SVP(gas)
HF= -4325.5686541
UPBEPBE-D3/def2-SVP-SMD(THF)//UB3LYP/def2-SVP(gas)
HF= -4322.3133719

[1]D’-TS-noligand

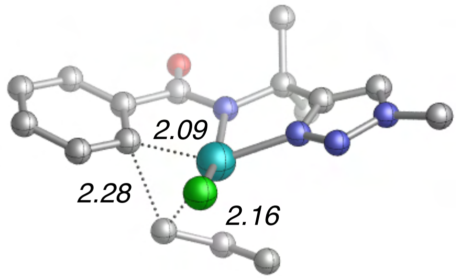

Zero-point correction= 0.329201 (Hartree/Particle)
Thermal correction to Energy= 0.350839
Thermal correction to Enthalpy= 0.351783
Thermal correction to Gibbs Free Energy= 0.279844
Sum of electronic and zero-point Energies= -2638.011240
Sum of electronic and thermal Energies= -2637.989601
Sum of electronic and thermal Enthalpies= -2637.988657
Sum of electronic and thermal Free Energies= -2638.060596

C	-18.39537900	-1.40026000	-4.80122900
C	-19.00865400	-0.15053800	-4.63683800
C	-20.39728200	-0.02053800	-4.73856400
C	-21.17722500	-1.15291900	-4.99138400
C	-20.56304800	-2.40407400	-5.12869100
C	-19.16867900	-2.53438800	-5.04634700
H	-20.84026000	0.96842500	-4.59420200
H	-22.26402000	-1.06536400	-5.06927200
H	-21.17411400	-3.29383500	-5.30673100
H	-18.69120100	-3.50992800	-5.15815700
C	-18.13357400	0.98940500	-4.22410800
O	-18.51909800	2.15388900	-4.16201300
C	-16.01572700	1.39085000	-3.02630800
C	-16.75831900	1.99628500	-1.80983800
H	-16.05209000	2.54633800	-1.16697800
H	-17.54132400	2.68344200	-2.15061000
H	-17.22386000	1.19768000	-1.21230400
C	-15.37258600	2.51563700	-3.86937000
H	-16.16976100	3.15337600	-4.27482800
H	-14.69637400	3.13532500	-3.25758300
H	-14.79343000	2.09452400	-4.70520700
C	-14.96203500	0.44321200	-2.50542700
N	-14.98822300	-0.85539200	-2.91751700
N	-14.04087900	-1.55376600	-2.35591100
C	-13.90969500	0.54216400	-1.61647300
H	-13.51442000	1.36657100	-1.03040900
N	-13.38500500	-0.71625700	-1.56721900
N	-16.89055700	0.52927800	-3.85049900
Fe	-16.37450700	-1.26097200	-4.29376500
Cl	-16.13565100	-3.50369500	-4.08689500
C	-14.35312600	-1.30391900	-5.91456300
H	-14.30366200	-2.37625600	-5.71084100
H	-13.44007800	-0.71688900	-5.78436400

C	-15.49693100	-0.71198800	-6.36196400
H	-15.48772800	0.36713500	-6.54855900
C	-16.78450200	-1.37369400	-6.41619000
H	-17.57636300	-0.82792800	-6.92794200
H	-16.79178600	-2.45398000	-6.57847200
C	-12.26123100	-1.20253600	-0.78181800
H	-11.87668200	-0.38018000	-0.16623900
H	-12.59219100	-2.02565700	-0.13385800
H	-11.46762700	-1.56626500	-1.44940300

UB3LYP-D3/def2-SVP-SMD(THF)//UB3LYP/def2-SVP(gas)
HF= -2638.4028789
UPBEPBE-D3/def2-SVP-SMD(THF)//UB3LYP/def2-SVP(gas)
HF= -2636.8253172

[3]D'-TS-noligand

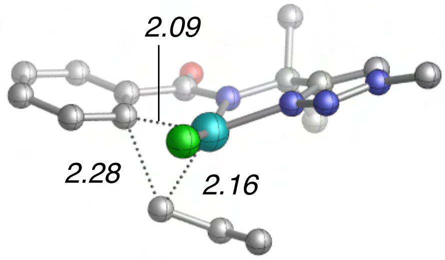

Zero-point correction= 0.328856 (Hartree/Particle)
Thermal correction to Energy= 0.350635
Thermal correction to Enthalpy= 0.351579
Thermal correction to Gibbs Free Energy= 0.278292
Sum of electronic and zero-point Energies= -2638.029216
Sum of electronic and thermal Energies= -2638.007437
Sum of electronic and thermal Enthalpies= -2638.006493
Sum of electronic and thermal Free Energies= -2638.079780

C	-18.42939300	-1.40918900	-4.77771900
C	-19.03109200	-0.15242100	-4.62447000
C	-20.41333700	-0.00463800	-4.77277000
C	-21.19730500	-1.12656400	-5.06003700
C	-20.59514600	-2.38499400	-5.18492000
C	-19.20637200	-2.53370600	-5.05328800
H	-20.84952600	0.98881300	-4.63895500
H	-22.27957200	-1.02489200	-5.17519200
H	-21.21147200	-3.26527500	-5.38988300
H	-18.73636800	-3.51442000	-5.15319300
C	-18.15203600	0.97922500	-4.19132400
O	-18.53438100	2.14484700	-4.12857100
C	-16.02543000	1.38349100	-3.00671600
C	-16.75246500	1.99950200	-1.78575200
H	-16.03953300	2.55872800	-1.15837200
H	-17.54249100	2.68010300	-2.12362400
H	-17.20706500	1.20559600	-1.17366600
C	-15.39405300	2.50045900	-3.86842600
H	-16.19648400	3.13467000	-4.26887100
H	-14.70973300	3.12588500	-3.27165300
H	-14.82637700	2.07117600	-4.70787900
C	-14.96171200	0.44396400	-2.48956000
N	-14.99885100	-0.86540500	-2.86569400
N	-14.03844300	-1.54981900	-2.30877100
C	-13.88704000	0.56504700	-1.63022200
H	-13.47550700	1.40451300	-1.07769000
N	-13.36238700	-0.69246700	-1.55993900
N	-16.91440400	0.51504200	-3.80860200
Fe	-16.42070900	-1.29246600	-4.21878500
Cl	-16.15683700	-3.53227400	-4.05731300
C	-14.31597600	-1.37555800	-5.96620800
H	-14.29913500	-2.44383400	-5.73499200
H	-13.37405400	-0.82409700	-5.90102700

C	-15.46046600	-0.74637000	-6.33951000
H	-15.42500500	0.33136900	-6.53284300
C	-16.77926700	-1.36645400	-6.35219800
H	-17.55560600	-0.78978800	-6.85355800
H	-16.81943400	-2.44199100	-6.53858100
C	-12.21910300	-1.15859900	-0.79040300
H	-11.81398300	-0.31852800	-0.21318500
H	-12.53502700	-1.95843700	-0.10673000
H	-11.44630600	-1.54792500	-1.46779600

UB3LYP-D3/def2-SVP-SMD(THF)//UB3LYP/def2-SVP(gas)
HF= -2638.4465703
UPBEPBE-D3/def2-SVP-SMD(THF)//UB3LYP/def2-SVP(gas)
HF= -2636.8787259

^{5}D'-TS-noligand

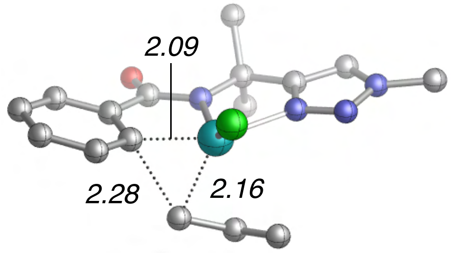

Zero-point correction= 0.327470 (Hartree/Particle)
Thermal correction to Energy= 0.350659
Thermal correction to Enthalpy= 0.351604
Thermal correction to Gibbs Free Energy= 0.273282
Sum of electronic and zero-point Energies= -2638.032652
Sum of electronic and thermal Energies= -2638.009463
Sum of electronic and thermal Enthalpies= -2638.008519
Sum of electronic and thermal Free Energies= -2638.086841

C	-18.49214200	-1.33392300	-4.95185100
C	-19.05605300	-0.08483900	-4.67273800
C	-20.43512600	0.10060900	-4.83270100
C	-21.24212200	-0.96235000	-5.24359200
C	-20.67076500	-2.21861100	-5.47943200
C	-19.28914400	-2.40874600	-5.34414900
H	-20.84506700	1.08718000	-4.60153000
H	-22.31872900	-0.81912900	-5.36597400
H	-21.30067700	-3.06151900	-5.77738300
H	-18.84869100	-3.39142200	-5.52708900
C	-18.20131100	1.00077600	-4.07969900
O	-18.61376300	2.15520300	-3.96415100
C	-16.12040400	1.38837900	-2.86220100
C	-16.81829600	1.89352200	-1.57607400
H	-16.12220100	2.48859800	-0.96241800
H	-17.67858700	2.51858300	-1.84492200
H	-17.17074900	1.04062000	-0.97617000
C	-15.60492700	2.58600600	-3.69749500
H	-16.45767300	3.20406800	-4.00604500
H	-14.90456400	3.20407100	-3.11189900
H	-15.08048800	2.22886600	-4.59765600
C	-14.94743300	0.52082400	-2.46344900
N	-14.85627600	-0.75219300	-2.92676800
N	-13.78396100	-1.34165000	-2.48031500
C	-13.83776900	0.72461200	-1.66237100
H	-13.49268500	1.57446100	-1.08054800
N	-13.15983800	-0.45618600	-1.71093400
N	-17.00012900	0.51087300	-3.65123500
Fe	-16.55033400	-1.40241100	-4.18664900
Cl	-16.59193300	-3.52595600	-3.46181400
C	-14.35099400	-2.13803400	-5.82568700
H	-14.63596000	-3.18400800	-5.68188500
H	-13.30620900	-1.87393800	-5.64767800
C	-15.26276300	-1.19808600	-6.18471900

H	-14.93389200	-0.15691200	-6.28241100
C	-16.69088400	-1.45940700	-6.34592800
H	-17.24656000	-0.70776500	-6.90872300
H	-16.95379700	-2.48724600	-6.61148200
C	-11.91241900	-0.81270400	-1.05696400
H	-11.98002600	-0.61310500	0.02193800
H	-11.74890800	-1.88405600	-1.22288700
H	-11.07463400	-0.24073700	-1.48321000

UB3LYP-D3/def2-SVP-SMD(THF)//UB3LYP/def2-SVP(gas)
HF= -2638.4484247
UPBEPBE-D3/def2-SVP-SMD(THF)//UB3LYP/def2-SVP(gas)
HF= -2636.8673726

¹D''-TS

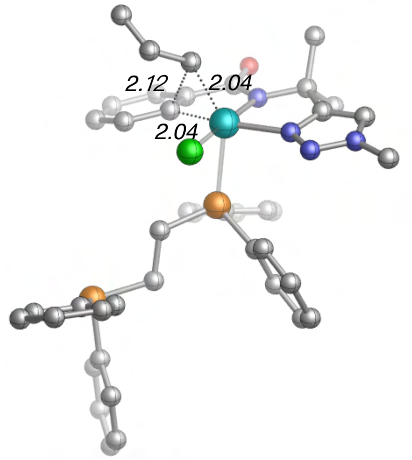

Zero-point correction= 0.752588 (Hartree/Particle)
Thermal correction to Energy= 0.802122
Thermal correction to Enthalpy= 0.803066
Thermal correction to Gibbs Free Energy= 0.663244
Sum of electronic and zero-point Energies= -4324.623819
Sum of electronic and thermal Energies= -4324.574286
Sum of electronic and thermal Enthalpies= -4324.573341
Sum of electronic and thermal Free Energies= -4324.713163

C	-18.33023700	0.94578700	-3.45490100
C	-19.13244900	2.05482400	-3.12507500
C	-20.51079900	2.03611200	-3.35425300
C	-21.10238900	0.90682800	-3.92788600
C	-20.30827500	-0.19907700	-4.26131500
C	-18.93032900	-0.19070600	-4.00999300
H	-21.09231700	2.91941400	-3.07845700
H	-22.18011300	0.88145100	-4.10947000
H	-20.76745100	-1.08663100	-4.70636900
H	-18.32345400	-1.06984400	-4.23708800
C	-18.44290800	3.24548700	-2.52877600
O	-19.05761300	4.18146100	-2.01216600
C	-16.20053500	4.15631000	-2.07213500
C	-16.27912600	4.14011200	-0.52439600
H	-15.58933100	4.87763700	-0.08229300
H	-17.30511300	4.38704700	-0.21908200
H	-16.01981900	3.14514700	-0.13098500
C	-16.49467600	5.58419700	-2.58667000
H	-17.51506800	5.86986200	-2.30542500
H	-15.78080400	6.30263200	-2.15079900
H	-16.40725900	5.62858500	-3.68035400
C	-14.81510600	3.74426500	-2.50934800
N	-14.64479800	2.56767500	-3.18150800
N	-13.38475000	2.35596200	-3.44137900
C	-13.55604500	4.29123000	-2.35924300
H	-13.20060000	5.20708800	-1.89613000
N	-12.71928900	3.39194400	-2.95119800
N	-17.08770900	3.12620700	-2.64468000

Fe	-16.36122200	1.48021900	-3.36715700
P	-16.63639600	2.01570600	-5.86988600
C	-17.22382900	0.68207800	-8.37912800
H	-17.82792100	1.55331300	-8.68060100
H	-16.21578200	0.85222400	-8.78809700
C	-17.19880400	0.53440600	-6.85582800
H	-16.52123600	-0.26875500	-6.52796800
H	-18.18998100	0.25949500	-6.46564500
C	-15.10015100	2.50056400	-6.79581800
C	-15.07409100	3.47614000	-7.80884500
C	-13.91907300	1.78710700	-6.51596300
C	-13.89535600	3.74375500	-8.51208500
H	-15.97892000	4.02835800	-8.06571200
C	-12.74582100	2.05218600	-7.22926000
H	-13.92331700	1.01594700	-5.74245600
C	-12.72646100	3.03382000	-8.22436900
H	-13.89752500	4.50479500	-9.29701000
H	-11.84042500	1.48278500	-7.00166400
H	-11.80725400	3.23986100	-8.77943800
C	-17.86425500	3.33260500	-6.26907000
C	-19.18287900	3.01757400	-6.64003600
C	-17.53059800	4.68798100	-6.09101300
C	-20.12865000	4.02638300	-6.84757700
H	-19.49303300	1.97848000	-6.75849200
C	-18.47393400	5.69539700	-6.30076200
H	-16.51760600	4.96272800	-5.79024200
C	-19.77837300	5.36779100	-6.68291800
H	-21.14812300	3.75562600	-7.13353900
H	-18.18992800	6.74108000	-6.15718300
H	-20.51923100	6.15504500	-6.84316700
Cl	-15.28269500	-0.44253900	-4.10827600
C	-11.27900000	3.47403500	-3.12358600
H	-11.02436200	4.22106800	-3.89035600
H	-10.80217200	3.74610600	-2.17160800
H	-10.92717700	2.48669900	-3.44486200
P	-18.06178600	-0.76933200	-9.23221000
C	-17.92958100	-0.18882100	-10.99456500
C	-19.03238600	0.50678300	-11.52212500
C	-16.81161600	-0.39806900	-11.81983300
C	-19.01210700	0.99913800	-12.83011000
H	-19.92060700	0.65764800	-10.90092000
C	-16.79495400	0.08441300	-13.13200200
H	-15.94735500	-0.94576400	-11.43697100
C	-17.89234200	0.78682100	-13.63982600
H	-19.87805800	1.54007100	-13.22124200
H	-15.91797300	-0.08970000	-13.76155500
H	-17.87729200	1.16225900	-14.66643500
C	-16.78831900	-2.11376000	-9.16417700
C	-17.22050800	-3.40635600	-9.51717000
C	-15.45412300	-1.94641300	-8.75591000
C	-16.34353000	-4.49233900	-9.48659800

H	-18.26140400	-3.56108500	-9.81701500
C	-14.57831700	-3.03694000	-8.70949600
H	-15.08106000	-0.96202200	-8.46644300
C	-15.01779200	-4.31051100	-9.07867700
H	-16.69910200	-5.48643100	-9.77059300
H	-13.54679300	-2.88648600	-8.38036300
H	-14.33219600	-5.16107800	-9.04120000
C	-18.18799100	-1.57049800	-0.78902700
H	-18.91529600	-0.95773100	-0.24588400
H	-18.24312000	-2.65272500	-0.64217600
C	-17.27109700	-1.02501600	-1.60215900
H	-16.56455000	-1.65544700	-2.14765500
C	-17.12536400	0.43017900	-1.79109700
H	-16.05898500	0.72543300	-1.59028100
H	-17.77817600	1.01099400	-1.13368700

UB3LYP-D3/def2-SVP-SMD(THF)//UB3LYP/def2-SVP(gas)
HF= -4325.5552781
UPBEPBE-D3/def2-SVP-SMD(THF)//UB3LYP/def2-SVP(gas)
HF= -4322.3204338

^{3}D''-TS

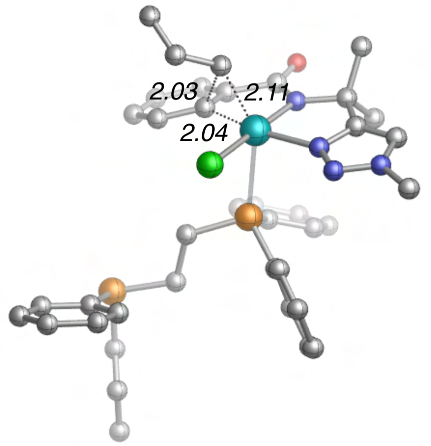

Zero-point correction= 0.752078 (Hartree/Particle)
Thermal correction to Energy= 0.801988
Thermal correction to Enthalpy= 0.802932
Thermal correction to Gibbs Free Energy= 0.660470
Sum of electronic and zero-point Energies= -4324.639186
Sum of electronic and thermal Energies= -4324.589277
Sum of electronic and thermal Enthalpies= -4324.588333
Sum of electronic and thermal Free Energies= -4324.730795

C	-18.24027900	0.77030400	-3.33253400
C	-19.10326600	1.85474900	-3.06644000
C	-20.45187700	1.80362500	-3.42544200
C	-20.95498600	0.66967200	-4.07103100
C	-20.10019800	-0.40525400	-4.35343400
C	-18.75408600	-0.36598400	-3.97410700
H	-21.08005900	2.66871000	-3.19871700
H	-22.00979600	0.61907400	-4.35341300
H	-20.48770700	-1.29072100	-4.86520300
H	-18.10103400	-1.21735300	-4.17428100
C	-18.50021200	3.07606000	-2.43938800
O	-19.17883200	3.98643500	-1.96409500
C	-16.32506700	4.15001300	-1.99042000
C	-16.44577700	4.25204700	-0.45073600
H	-15.81235500	5.06324000	-0.05565800
H	-17.49170900	4.45974800	-0.18792700
H	-16.13884900	3.30837000	0.02619900
C	-16.69740700	5.50257600	-2.64042000
H	-17.73116500	5.76162200	-2.38235600
H	-16.02312900	6.29898100	-2.28456500
H	-16.61776000	5.43639700	-3.73423900
C	-14.90582300	3.79573600	-2.36631200
N	-14.65598600	2.62226200	-3.01796600
N	-13.38022600	2.47317200	-3.24193400
C	-13.68119600	4.40899000	-2.18863800
H	-13.38641300	5.34732400	-1.72783600
N	-12.78284700	3.54846100	-2.74692300
N	-17.13452400	3.02679400	-2.50355700

Fe	-16.31554200	1.45251500	-3.24299400
P	-16.52107200	2.11306500	-5.95258100
C	-17.28253500	0.74960500	-8.41398100
H	-17.90469300	1.61282000	-8.70202700
H	-16.29626200	0.91282800	-8.87581800
C	-17.18377700	0.63974700	-6.89137200
H	-16.51947400	-0.18117400	-6.57762600
H	-18.16300600	0.40537400	-6.44643100
C	-14.96122000	2.48016000	-6.89164400
C	-14.90836900	3.30308400	-8.03236700
C	-13.78219000	1.83799700	-6.46902300
C	-13.70706900	3.49168700	-8.72195200
H	-15.81036900	3.79938300	-8.39425100
C	-12.58467100	2.02208000	-7.16855300
H	-13.80636700	1.17921800	-5.59830700
C	-12.54026300	2.85265200	-8.29161700
H	-13.68758100	4.13430000	-9.60631100
H	-11.68078100	1.50901800	-6.82870200
H	-11.60239000	2.99739500	-8.83474100
C	-17.66609900	3.49374900	-6.38877100
C	-19.03532200	3.25212400	-6.60256000
C	-17.22450200	4.83108200	-6.37245500
C	-19.92583300	4.30857000	-6.81662800
H	-19.42515000	2.23268200	-6.59252100
C	-18.11422800	5.88555800	-6.59011700
H	-16.17010000	5.05481100	-6.19437800
C	-19.46984700	5.62860500	-6.81587000
H	-20.98487300	4.09348700	-6.98042200
H	-17.74557500	6.91472000	-6.57841400
H	-20.16692800	6.45342100	-6.98331700
Cl	-15.18134900	-0.39687700	-4.01593800
C	-11.34308400	3.69910000	-2.87103300
H	-11.09995200	4.49806100	-3.58730500
H	-10.90258100	3.93590800	-1.89219700
H	-10.94233400	2.74627200	-3.23605100
P	-18.15284400	-0.72322600	-9.19494700
C	-18.10651600	-0.18096800	-10.97396400
C	-19.24358500	0.48168900	-11.47009200
C	-17.01884600	-0.38595800	-11.83992600
C	-19.28647600	0.94584700	-12.78789800
H	-20.10840800	0.62901300	-10.81594400
C	-17.06513300	0.06837100	-13.16134400
H	-16.12886200	-0.90850200	-11.48151000
C	-18.19643500	0.73803300	-13.63824100
H	-20.17837900	1.46131600	-13.15434500
H	-16.21104700	-0.10210600	-13.82259000
H	-18.23070700	1.09146000	-14.67218600
C	-16.86867500	-2.05930100	-9.15973900
C	-17.30710100	-3.36012200	-9.47272700
C	-15.51938300	-1.87792400	-8.81127800
C	-16.42285800	-4.44054900	-9.46153100

H	-18.35871500	-3.52564300	-9.72583700
C	-14.63549500	-2.96265200	-8.78414800
H	-15.14016400	-0.88683600	-8.55447600
C	-15.08215700	-4.24465500	-9.11325900
H	-16.78388000	-5.44118300	-9.71398600
H	-13.59182400	-2.80067600	-8.50210300
H	-14.39024000	-5.09063600	-9.09109300
C	-18.14915500	-1.78988800	-0.76509900
H	-18.90552700	-1.22784300	-0.20688200
H	-18.15632200	-2.87755500	-0.65233900
C	-17.25312300	-1.17985100	-1.55218800
H	-16.51376600	-1.76150900	-2.10929300
C	-17.15487000	0.29201700	-1.69021800
H	-16.11646600	0.59586700	-1.41204600
H	-17.84282100	0.82417700	-1.02751800

UB3LYP-D3/def2-SVP-SMD(THF)//UB3LYP/def2-SVP(gas)
HF= -4325.5784534
UPBEPBE-D3/def2-SVP-SMD(THF)//UB3LYP/def2-SVP(gas)
HF= -4322.3353695

^{5}D''-TS

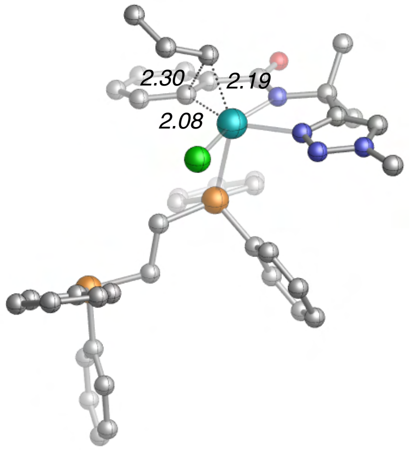

Zero-point correction= 0.750841 (Hartree/Particle)
Thermal correction to Energy= 0.801366
Thermal correction to Enthalpy= 0.802310
Thermal correction to Gibbs Free Energy= 0.656192
Sum of electronic and zero-point Energies= -4324.646617
Sum of electronic and thermal Energies= -4324.596092
Sum of electronic and thermal Enthalpies= -4324.595148
Sum of electronic and thermal Free Energies= -4324.741265

C	-18.46128400	1.14257800	-3.25719300
C	-19.05274900	2.37095200	-2.92646200
C	-20.43651600	2.53155200	-3.06908300
C	-21.21763100	1.48345600	-3.56184400
C	-20.61373900	0.26893600	-3.91070300
C	-19.23363000	0.08753500	-3.74341100
H	-20.86767500	3.49656700	-2.79176300
H	-22.29737600	1.60986900	-3.67575300
H	-21.22026400	-0.55374600	-4.30075300
H	-18.77430400	-0.87565100	-3.97868900
C	-18.19148800	3.50879600	-2.44298700
O	-18.69544700	4.53539700	-1.97674700
C	-15.88159600	4.21092100	-2.08540000
C	-16.02069000	4.39301900	-0.55246300
H	-15.23847200	5.06452600	-0.16194800
H	-17.00557800	4.82132200	-0.32669600
H	-15.92948800	3.42183900	-0.04195900
C	-15.96780400	5.58874400	-2.78599500
H	-16.97290700	6.00444900	-2.64178500
H	-15.22634900	6.28821900	-2.36576100
H	-15.76369400	5.48820800	-3.86283100
C	-14.52966800	3.59947400	-2.38053500
N	-14.44211900	2.37471700	-2.96489000
N	-13.19690000	2.02088000	-3.12702000
C	-13.23118800	4.02714300	-2.17136900
H	-12.81183400	4.93109700	-1.73913400
N	-12.45645600	3.01389300	-2.65075400
N	-16.86360200	3.24776300	-2.60155800

Fe	-16.40122800	1.39579900	-3.43410900
P	-16.91471900	2.02432000	-5.98102100
C	-17.22028100	0.60201100	-8.49411600
H	-17.77475600	1.47638500	-8.87171700
H	-16.17626900	0.74457200	-8.81468600
C	-17.32050600	0.49680400	-6.96918300
H	-16.64025100	-0.26905200	-6.56747600
H	-18.32925300	0.19134200	-6.64580900
C	-15.44986100	2.73624100	-6.86030300
C	-15.53785500	3.79167100	-7.78410600
C	-14.18939900	2.16689600	-6.59449200
C	-14.39158600	4.26848200	-8.42787200
H	-16.50486900	4.24614300	-8.00702600
C	-13.04920000	2.64157800	-7.24877500
H	-14.10280100	1.34709200	-5.87635500
C	-13.14493500	3.69458000	-8.16411300
H	-14.47762400	5.08946700	-9.14464600
H	-12.07891500	2.18432400	-7.03677400
H	-12.25099800	4.06625600	-8.67203800
C	-18.29162600	3.19932400	-6.31776000
C	-19.48010700	2.81820400	-6.96448600
C	-18.19491100	4.51025700	-5.81475500
C	-20.53118400	3.72776700	-7.11830600
H	-19.60597100	1.80567400	-7.34953800
C	-19.24331900	5.41778800	-5.97135800
H	-17.29591000	4.82301100	-5.28215600
C	-20.41637900	5.02941700	-6.62588800
H	-21.44671500	3.41035700	-7.62403400
H	-19.14791600	6.42752300	-5.56491000
H	-21.24089300	5.73718000	-6.74339900
Cl	-15.28875600	-0.48205100	-4.28252500
C	-11.00726900	2.92716900	-2.69905700
H	-10.59779300	3.70529900	-3.36009400
H	-10.58458200	3.04360900	-1.69063700
H	-10.75123800	1.93682400	-3.09365100
P	-18.01145000	-0.85493300	-9.38095600
C	-17.68727100	-0.34025900	-11.13866800
C	-18.72659400	0.32355700	-11.81447000
C	-16.47991700	-0.56699900	-11.82128900
C	-18.55908400	0.76777200	-13.12934800
H	-19.68116400	0.48729800	-11.30511500
C	-16.31516200	-0.13220300	-13.13959900
H	-15.66228700	-1.09158300	-11.32128800
C	-17.35196800	0.53875200	-13.79606600
H	-19.37780200	1.28423500	-13.63766600
H	-15.37030500	-0.31902100	-13.65729900
H	-17.22118000	0.87674200	-14.82744400
C	-16.79420300	-2.23126100	-9.14367200
C	-17.22206100	-3.51872200	-9.52052000
C	-15.51181700	-2.09256600	-8.58625300
C	-16.38802100	-4.62779400	-9.36787700

H	-18.22538400	-3.65104700	-9.93667600
C	-14.68134400	-3.20618400	-8.41677200
H	-15.14439600	-1.11394100	-8.27174900
C	-15.11373000	-4.47447800	-8.81103900
H	-16.73838000	-5.61746500	-9.67298800
H	-13.69213600	-3.07730500	-7.96993800
H	-14.46377700	-5.34312500	-8.67728200
C	-18.30572900	-1.69349600	-0.99957000
H	-19.09805400	-1.11951900	-0.50818300
H	-18.39910900	-2.78294300	-0.98573500
C	-17.26100600	-1.09120800	-1.59308700
H	-16.49941400	-1.69651500	-2.09392400
C	-17.05145300	0.35939500	-1.62288000
H	-15.99485700	0.61950300	-1.42469900
H	-17.71041200	0.93506600	-0.96842000

UB3LYP-D3/def2-SVP-SMD(THF)//UB3LYP/def2-SVP(gas)
HF= -4325.5885697
UPBEPBE-D3/def2-SVP-SMD(THF)//UB3LYP/def2-SVP(gas)
HF= -4322.3309822

[1]D''-outer-TS

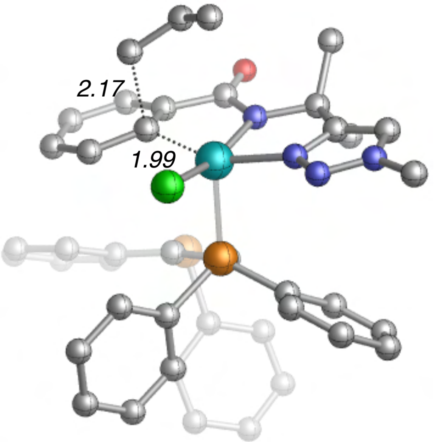

Zero-point correction= 0.750638 (Hartree/Particle)
Thermal correction to Energy= 0.801223
Thermal correction to Enthalpy= 0.802168
Thermal correction to Gibbs Free Energy= 0.658913
Sum of electronic and zero-point Energies= -4324.603944
Sum of electronic and thermal Energies= -4324.553359
Sum of electronic and thermal Enthalpies= -4324.552414
Sum of electronic and thermal Free Energies= -4324.695669

C	-19.09387700	1.57089100	-2.21761900
C	-18.96109300	2.92199500	-2.66613300
C	-19.90333100	3.52783900	-3.49721500
C	-20.96843300	2.76881800	-3.99563500
C	-21.05390100	1.39787800	-3.68358400
C	-20.13148600	0.80409200	-2.82591900
H	-19.76556600	4.57795200	-3.76865700
H	-21.71216300	3.22826900	-4.65171500
H	-21.86189000	0.79568400	-4.11017200
H	-20.23948500	-0.24920100	-2.55587700
C	-17.73132400	3.63381900	-2.22085900
O	-17.55320200	4.84623900	-2.35165600
C	-15.59162000	3.22268600	-1.07146100
C	-15.85296400	4.27188500	0.03731400
H	-14.90385200	4.57546100	0.50870200
H	-16.33534800	5.15515600	-0.39838400
H	-16.50864600	3.85015400	0.81272300
C	-14.63494600	3.80864900	-2.13623000
H	-14.37444500	3.04365200	-2.88258200
H	-15.12946400	4.65192200	-2.63638000
H	-13.70025200	4.16496400	-1.67300300
C	-14.97188500	2.00092800	-0.43945500
N	-15.60006200	0.79856900	-0.56756100
N	-14.95420000	-0.14050000	0.06572100
C	-13.84776800	1.77203900	0.32918600
H	-13.05708900	2.42158800	0.69306200
N	-13.89237000	0.43811300	0.60923900
N	-16.84608200	2.73335100	-1.67965200

Fe	-17.31066600	0.85688700	-1.68072100
P	-16.38614100	0.19164300	-3.91341400
P	-17.37016800	3.23681000	-7.15834500
C	-16.26663300	1.54449100	-5.19221000
H	-15.47642000	1.27299100	-5.90970800
H	-15.92894200	2.44643700	-4.65790400
C	-17.57604400	1.82230900	-5.93733100
H	-18.36265600	2.12703900	-5.23086200
H	-17.92287300	0.91596000	-6.45714700
C	-17.05471300	-1.23917200	-4.88100200
C	-18.11218100	-2.01562300	-4.38564000
C	-16.49360900	-1.56351500	-6.13123200
C	-18.60643000	-3.09085700	-5.13456400
H	-18.52305500	-1.80318100	-3.39739000
C	-16.99203500	-2.63379100	-6.87491500
H	-15.65732000	-0.98532900	-6.53168400
C	-18.05358100	-3.39959100	-6.37831600
H	-19.42730600	-3.69120400	-4.73392100
H	-16.54679500	-2.87304800	-7.84412600
H	-18.44241600	-4.23928700	-6.96047200
C	-14.63289000	-0.34083000	-3.65924500
C	-13.51925000	0.35131200	-4.16263200
C	-14.42087900	-1.49652100	-2.88382200
C	-12.22227000	-0.11389700	-3.91458800
H	-13.64650300	1.25600200	-4.75983100
C	-13.12692300	-1.96241500	-2.64700100
H	-15.27558600	-2.02456300	-2.45284400
C	-12.02240700	-1.27434600	-3.16367100
H	-11.36690500	0.43288400	-4.32016300
H	-12.98159200	-2.86912900	-2.05381300
H	-11.00939500	-1.64320700	-2.98191500
C	-16.21818700	2.44311000	-8.38998100
C	-16.57669500	1.35348400	-9.20424100
C	-14.91460300	2.95743100	-8.49268500
C	-15.65324600	0.79085200	-10.08872900
H	-17.59010900	0.94681000	-9.15248300
C	-13.98666900	2.39249500	-9.37547500
H	-14.62774100	3.81536900	-7.87755700
C	-14.35491600	1.30790500	-10.17503300
H	-15.94853000	-0.05313100	-10.71821300
H	-12.97712500	2.80682600	-9.44329700
H	-13.63475500	0.86807600	-10.87008200
C	-18.97143200	3.18367000	-8.09014100
C	-20.07972200	2.39842400	-7.73073900
C	-19.09950600	4.05519700	-9.18952300
C	-21.27443800	2.47427000	-8.45769200
H	-20.02813300	1.72200100	-6.87618000
C	-20.28621800	4.12239900	-9.91995500
H	-18.25540800	4.68947200	-9.47670600
C	-21.38148800	3.33094000	-9.55483000
H	-22.12501700	1.85438000	-8.16129600

H	-20.36016900	4.80121100	-10.77383300
H	-22.31452800	3.38702900	-10.12146800
Cl	-18.01377900	-1.19607800	-1.01201300
C	-18.53346500	1.62352300	1.71862100
H	-17.92866800	2.13901000	2.46958400
H	-18.57580000	0.53119200	1.76556900
C	-19.19821000	2.31380300	0.75398900
H	-19.10599200	3.40674400	0.73951100
C	-20.02894600	1.72396100	-0.26009900
H	-20.25358300	0.65954400	-0.16098300
H	-20.82677800	2.33968600	-0.67814200
C	-12.93217100	-0.36216100	1.34871900
H	-12.03211200	-0.54082200	0.74121900
H	-12.65349800	0.15206200	2.27894600
H	-13.40995400	-1.32036100	1.58468200

UB3LYP-D3/def2-SVP-SMD(THF)//UB3LYP/def2-SVP(gas)
HF= -4325.5332985
UPBEPBE-D3/def2-SVP-SMD(THF)//UB3LYP/def2-SVP(gas)
HF= -4322.2909020

^{3}D''-outer-TS

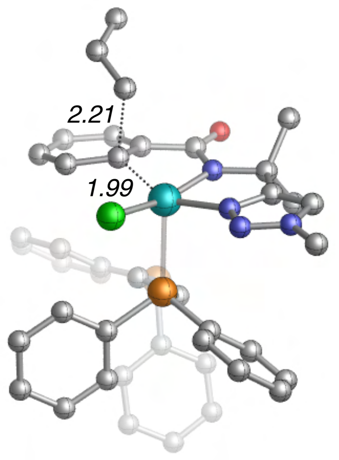

Zero-point correction= 0.749867 (Hartree/Particle)
Thermal correction to Energy= 0.800810
Thermal correction to Enthalpy= 0.801754
Thermal correction to Gibbs Free Energy= 0.654628
Sum of electronic and zero-point Energies= -4324.629687
Sum of electronic and thermal Energies= -4324.578744
Sum of electronic and thermal Enthalpies= -4324.577800
Sum of electronic and thermal Free Energies= -4324.724927

C	-19.05667900	1.42271100	-2.02094500
C	-18.92589500	2.76098600	-2.49151800
C	-19.89885100	3.36465500	-3.28623700
C	-21.00013400	2.61222800	-3.71113100
C	-21.09851200	1.25349200	-3.35677500
C	-20.13915700	0.65796100	-2.54075700
H	-19.76116000	4.40663900	-3.58762000
H	-21.76838600	3.07083500	-4.33909800
H	-21.94264300	0.65959400	-3.72046200
H	-20.23797300	-0.39010800	-2.24884200
C	-17.66906100	3.46288200	-2.10169800
O	-17.48727800	4.67362400	-2.22321800
C	-15.49532600	3.01734300	-1.01680800
C	-15.73868500	4.01690500	0.14029200
H	-14.78367500	4.30609500	0.60852400
H	-16.23278800	4.91593300	-0.24803200
H	-16.37853800	3.56280300	0.91237000
C	-14.56429900	3.65125000	-2.07641600
H	-15.06881400	4.51557900	-2.52778000
H	-13.61813800	3.98511500	-1.61976400
H	-14.32341400	2.92038200	-2.86250400
C	-14.85856600	1.77016400	-0.45395900
N	-15.49834200	0.57679100	-0.60606000
N	-14.83443700	-0.39042900	-0.03725700
C	-13.70837200	1.50537700	0.26242700
H	-12.89996900	2.13564000	0.62129700
N	-13.75041200	0.16110600	0.48994200
N	-16.76508800	2.55002500	-1.60967700

Fe	-17.23162000	0.67974400	-1.71306200
P	-16.13186100	0.05726400	-4.07942700
P	-17.12901700	3.31080700	-7.10001700
C	-15.97917500	1.45903100	-5.30156100
H	-15.25456100	1.17409400	-6.08098800
H	-15.55430000	2.31601400	-4.75464200
C	-17.31846300	1.84581900	-5.93800300
H	-18.03645500	2.15354100	-5.16097300
H	-17.75334500	0.98560000	-6.47126000
C	-16.81249500	-1.30706100	-5.12855400
C	-17.98703500	-1.96984300	-4.74167200
C	-16.17782600	-1.67889800	-6.32850700
C	-18.52312000	-2.98007400	-5.54946600
H	-18.46401800	-1.71704700	-3.79334800
C	-16.71688400	-2.68510400	-7.13137000
H	-15.25361900	-1.18593800	-6.64093100
C	-17.89408100	-3.33637500	-6.74373000
H	-19.43607800	-3.49250300	-5.23539600
H	-16.21484300	-2.96426200	-8.06142300
H	-18.31500400	-4.12570700	-7.37224500
C	-14.39094100	-0.50149500	-3.81489900
C	-13.26050600	0.17906200	-4.29675300
C	-14.20557500	-1.66476900	-3.04298700
C	-11.97349800	-0.30363000	-4.03051400
H	-13.36815700	1.08869800	-4.89052700
C	-12.92138700	-2.14891800	-2.78934000
H	-15.07464900	-2.18366600	-2.62920400
C	-11.80017300	-1.47134500	-3.28407100
H	-11.10469900	0.23447200	-4.41892600
H	-12.79646600	-3.06156900	-2.20047500
H	-10.79485400	-1.85418800	-3.08885600
C	-16.08394500	2.54777400	-8.43983100
C	-16.51489200	1.48944700	-9.26051100
C	-14.78616700	3.05387200	-8.62311800
C	-15.66722500	0.95026500	-10.23100700
H	-17.52535600	1.08810800	-9.14612700
C	-13.93415300	2.51286000	-9.59308100
H	-14.44397900	3.88629500	-8.00136400
C	-14.37385700	1.45985400	-10.39865700
H	-16.01811200	0.13013100	-10.86337600
H	-12.92804200	2.92065400	-9.72308900
H	-13.71325600	1.03846500	-11.16106100
C	-18.78600400	3.36176400	-7.92842700
C	-19.91974000	2.66620500	-7.47524900
C	-18.93049300	4.22632000	-9.03138200
C	-21.15569300	2.82143200	-8.11520800
H	-19.85706700	1.99886700	-6.61415100
C	-20.15973800	4.37239100	-9.67469200
H	-18.06573900	4.79108000	-9.39245100
C	-21.28039100	3.66940100	-9.21712000
H	-22.02492000	2.27019300	-7.74625700

H	-20.24597700	5.04336800	-10.53361300
H	-22.24581800	3.78708000	-9.71600200
Cl	-17.90123100	-1.43315800	-1.22454900
C	-21.14038200	3.58246900	0.26979800
H	-22.14536300	4.01183300	0.26827600
H	-20.30071400	4.27503400	0.38597800
C	-20.93930100	2.24684500	0.12654900
H	-21.81550200	1.60142100	-0.00807800
C	-19.65727900	1.61000600	0.09817000
H	-18.79071800	2.19522300	0.41787000
H	-19.59165500	0.53701200	0.29040700
C	-12.77145500	-0.67066000	1.16816900
H	-13.23158100	-1.65117300	1.33788500
H	-11.87212800	-0.78952300	0.54554400
H	-12.49711800	-0.21835600	2.13144800

UB3LYP-D3/def2-SVP-SMD(THF)//UB3LYP/def2-SVP(gas)
HF= -4325.5744132
UPBEPBE-D3/def2-SVP-SMD(THF)//UB3LYP/def2-SVP(gas)
HF= -4322.3212100

[5]D''-outer-TS

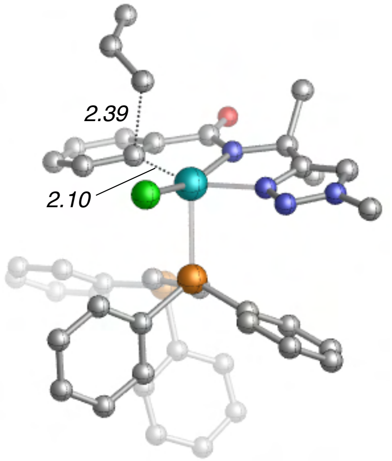

Zero-point correction= 0.749065 (Hartree/Particle)
Thermal correction to Energy= 0.800334
Thermal correction to Enthalpy= 0.801278
Thermal correction to Gibbs Free Energy= 0.652988
Sum of electronic and zero-point Energies= -4324.641874
Sum of electronic and thermal Energies= -4324.590605
Sum of electronic and thermal Enthalpies= -4324.589660
Sum of electronic and thermal Free Energies= -4324.737951

C	-18.86881300	1.37537600	-2.13411500
C	-18.67430300	2.73747600	-2.42943900
C	-19.61408300	3.44111900	-3.18676600
C	-20.74199200	2.77813000	-3.68355800
C	-20.91818300	1.41001000	-3.42590500
C	-19.98908900	0.70634300	-2.65121100
H	-19.43142400	4.50101400	-3.38342100
H	-21.48071400	3.32258100	-4.27698700
H	-21.79853600	0.89060000	-3.81642800
H	-20.15091000	-0.34858100	-2.41584700
C	-17.42603200	3.39830200	-1.91882300
O	-17.27703200	4.62135200	-1.93573500
C	-15.24718800	2.92753700	-0.88791900
C	-15.46523300	3.81607700	0.36067100
H	-14.50070000	4.10684600	0.80847200
H	-16.01052400	4.72338200	0.07161000
H	-16.04780000	3.27478900	1.12230300
C	-14.38294500	3.68090100	-1.92830200
H	-14.92299100	4.57223800	-2.27216800
H	-13.42033100	3.98974300	-1.48874300
H	-14.17135100	3.03155100	-2.79136100
C	-14.52620800	1.66427000	-0.47049600
N	-15.08134000	0.44561500	-0.71728700
N	-14.32383700	-0.51471000	-0.26966200
C	-13.32763900	1.42199900	0.17430600
H	-12.55271000	2.07707100	0.56154600
N	-13.25864000	0.06469400	0.27078900

N	-16.52430700	2.47350600	-1.46807500
Fe	-17.01745300	0.52811100	-1.62983700
P	-16.06121100	-0.05983700	-4.05019800
P	-17.21444200	3.18169000	-7.03789600
C	-15.99033000	1.35595200	-5.26782300
H	-15.26086500	1.11051700	-6.05608500
H	-15.59558100	2.22432100	-4.71526700
C	-17.34762100	1.69814400	-5.89152100
H	-18.07210400	1.96956500	-5.10774300
H	-17.75144200	0.82929600	-6.43477700
C	-16.77611300	-1.43035300	-5.06986400
C	-17.88936700	-2.14165300	-4.59755000
C	-16.22924700	-1.76684500	-6.32236800
C	-18.45149200	-3.16547700	-5.37012100
H	-18.29616300	-1.91496300	-3.61043200
C	-16.79456800	-2.78526200	-7.09103400
H	-15.35288400	-1.23549500	-6.70226700
C	-17.91005600	-3.48606500	-6.61623700
H	-19.31506400	-3.71617100	-4.98826400
H	-16.36114100	-3.03563700	-8.06289700
H	-18.35083200	-4.28538300	-7.21797300
C	-14.29421800	-0.59169900	-3.91705300
C	-13.20755900	0.18918600	-4.34427400
C	-14.03459700	-1.82878700	-3.29775100
C	-11.89396800	-0.26545400	-4.17586800
H	-13.37043100	1.15840600	-4.81916400
C	-12.72525600	-2.28486000	-3.14109300
H	-14.86607800	-2.43342300	-2.92655300
C	-11.64876500	-1.50552100	-3.58174800
H	-11.06110700	0.35263400	-4.52181700
H	-12.54511000	-3.25460000	-2.66958700
H	-10.62317700	-1.86583100	-3.46436200
C	-16.15676500	2.46374100	-8.39323400
C	-16.57027300	1.41045900	-9.22912800
C	-14.87023100	2.99858500	-8.57380500
C	-15.71632400	0.90318300	-10.21124100
H	-17.57243300	0.98837200	-9.11679600
C	-14.01189300	2.48954900	-9.55551700
H	-14.54158900	3.82763800	-7.94034600
C	-14.43397100	1.44072500	-10.37578500
H	-16.05367200	0.08645500	-10.85531200
H	-13.01450700	2.91900400	-9.68286600
H	-13.76826600	1.04443500	-11.14713400
C	-18.87576300	3.19090600	-7.85975200
C	-19.97911700	2.43336100	-7.43211200
C	-19.05668000	4.08436800	-8.93398400
C	-21.22044100	2.55705400	-8.06881800
H	-19.88763300	1.74166200	-6.59341100
C	-20.29078100	4.19982600	-9.57423400
H	-18.21641400	4.69682800	-9.27465200
C	-21.38075300	3.43512600	-9.14218500

H	-22.06572400	1.95728400	-7.72039300
H	-20.40510100	4.89486200	-10.41044900
H	-22.35015200	3.52813900	-9.63858300
Cl	-17.73069200	-1.56093100	-1.06447600
C	-20.61641900	3.33467000	0.60314100
H	-21.59661600	3.80483200	0.71396400
H	-19.73889900	3.98638400	0.65769600
C	-20.49041800	1.99456300	0.39060700
H	-21.40289900	1.39147200	0.31487500
C	-19.25237600	1.31618400	0.22364600
H	-18.32471900	1.84995700	0.44340500
H	-19.21227300	0.22697100	0.25629800
C	-12.18880200	-0.75217600	0.81578300
H	-12.58948000	-1.75983100	0.97796400
H	-11.34600300	-0.80376400	0.10998700
H	-11.84607900	-0.33134800	1.77114300

UB3LYP-D3/def2-SVP-SMD(THF)//UB3LYP/def2-SVP(gas)
HF= -4325.5838961
UPBEPBE-D3/def2-SVP-SMD(THF)//UB3LYP/def2-SVP(gas)
HF= -4322.3188493

[1]E

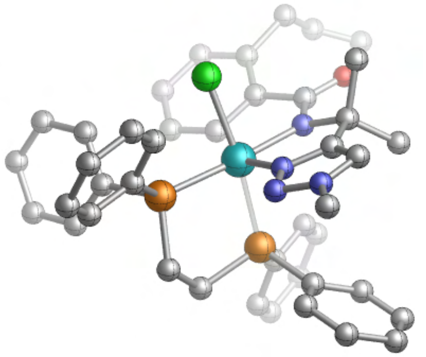

Zero-point correction= 0.756365 (Hartree/Particle)
Thermal correction to Energy= 0.805588
Thermal correction to Enthalpy= 0.806532
Thermal correction to Gibbs Free Energy= 0.670463
Sum of electronic and zero-point Energies= -4324.685401
Sum of electronic and thermal Energies= -4324.636177
Sum of electronic and thermal Enthalpies= -4324.635233
Sum of electronic and thermal Free Energies= -4324.771303

C	-20.71503800	2.22061300	-1.99744400
C	-19.49693300	2.21395600	-2.72701600
C	-19.31366700	1.19732800	-3.67861400
C	-20.26569300	0.21666700	-3.94596000
C	-21.45376500	0.21908000	-3.21603300
C	-21.65680200	1.20788800	-2.25524200
H	-18.43848100	1.22401400	-4.35056000
H	-20.07924900	-0.53931300	-4.71067900
H	-22.21565600	-0.54467800	-3.39217900
H	-22.58388600	1.20250400	-1.67381400
C	-18.39501100	3.23535600	-2.47615500
O	-18.68184700	4.31946000	-1.93448200
C	-16.03053700	3.70270400	-2.33410000
C	-16.03990700	3.77103000	-0.78338100
H	-15.14515600	4.30106100	-0.41610300
H	-16.93404800	4.30647900	-0.44383000
H	-16.05014300	2.75143900	-0.37215600
C	-16.00540300	5.12823700	-2.93413700
H	-16.93052300	5.64655300	-2.65599700
H	-15.14237200	5.70042000	-2.55325400
H	-15.92805300	5.08650300	-4.03064900
C	-14.76742000	2.97782900	-2.73381300
N	-14.83446900	1.76285300	-3.34215700
N	-13.64515900	1.28277100	-3.58121700
C	-13.42139400	3.25565200	-2.58507400
H	-12.89016200	4.09751800	-2.15105300
N	-12.78002800	2.17905400	-3.11713200
N	-17.14808900	2.87743300	-2.85348600
Fe	-16.64783700	1.12193900	-3.67532100
P	-16.12070300	-0.98106800	-4.52950300
P	-16.50496500	1.88486000	-5.95327200

C	-15.50027600	-0.75104300	-6.27712100
H	-15.51582300	-1.68773400	-6.85526500
H	-14.44040200	-0.48049700	-6.15569100
C	-16.27568900	0.35068000	-7.00965200
H	-17.29520000	0.00161200	-7.23890300
H	-15.79462800	0.60881900	-7.96619800
C	-17.53497700	-2.16540200	-4.68305900
C	-18.16251200	-2.57780700	-3.49189800
C	-18.01411600	-2.65830000	-5.90787200
C	-19.22947200	-3.47679700	-3.53227400
H	-17.82249700	-2.16792400	-2.53688400
C	-19.09184500	-3.55111900	-5.94381600
H	-17.55352600	-2.35954800	-6.85179900
C	-19.69849000	-3.96632100	-4.75627900
H	-19.70461200	-3.78843200	-2.59864000
H	-19.45260000	-3.92463800	-6.90587800
H	-20.53715100	-4.66689100	-4.78439400
C	-14.80788200	-2.07342600	-3.80983300
C	-14.37051600	-3.20695700	-4.51883800
C	-14.23519900	-1.78717800	-2.56137800
C	-13.36595000	-4.02713400	-4.00065400
H	-14.82389800	-3.46832500	-5.47844600
C	-13.23464500	-2.61620700	-2.04215800
H	-14.60787500	-0.93809500	-1.98627500
C	-12.79300500	-3.73073100	-2.75940100
H	-13.03713600	-4.90484000	-4.56344800
H	-12.80446200	-2.38984400	-1.06276400
H	-12.01171700	-4.37597900	-2.34856100
C	-15.08383600	2.96561800	-6.47020700
C	-15.26555600	4.34128200	-6.69716100
C	-13.79140800	2.43107700	-6.62353600
C	-14.19081200	5.15532500	-7.06676900
H	-16.25737700	4.78524800	-6.59982300
C	-12.71949500	3.24472500	-7.00269300
H	-13.60627700	1.37144700	-6.44739800
C	-12.91371200	4.61128200	-7.22326100
H	-14.35925400	6.22111000	-7.24078700
H	-11.72778600	2.80275800	-7.13338100
H	-12.07603600	5.24681500	-7.52155700
C	-17.93266800	2.74053200	-6.75470200
C	-18.29857600	2.51419200	-8.09404500
C	-18.66894400	3.66986800	-5.99950900
C	-19.38099200	3.19392900	-8.65800300
H	-17.73922800	1.81096300	-8.71409300
C	-19.74901000	4.35209800	-6.56803000
H	-18.40078700	3.85957600	-4.95958200
C	-20.10953600	4.11318200	-7.89616500
H	-19.65511200	3.00446600	-9.69912900
H	-20.31385100	5.06146300	-5.95856100
H	-20.95878600	4.64028500	-8.33848400
Cl	-16.85918800	0.12186700	-1.56940000

C	-21.63566100	5.60912000	-1.74371500
H	-22.34256900	6.33293500	-2.16182900
H	-20.62004300	5.94950400	-1.52651300
C	-21.99327500	4.33996700	-1.52956500
H	-23.00991400	4.02013000	-1.79777000
C	-21.09807200	3.26280100	-0.95443100
H	-21.65424300	2.74205400	-0.15472400
H	-20.20517300	3.71968800	-0.52000000
C	-11.35437800	1.95791700	-3.27023300
H	-10.96734300	2.50791100	-4.14202600
H	-10.82843600	2.29043700	-2.36483000
H	-11.19283900	0.88256100	-3.41324200

UB3LYP-D3/def2-SVP-SMD(THF)//UB3LYP/def2-SVP(gas)
HF= -4325.6450020
UPBEPBE-D3/def2-SVP-SMD(THF)//UB3LYP/def2-SVP(gas)
HF= -4322.3987643

^{3}E

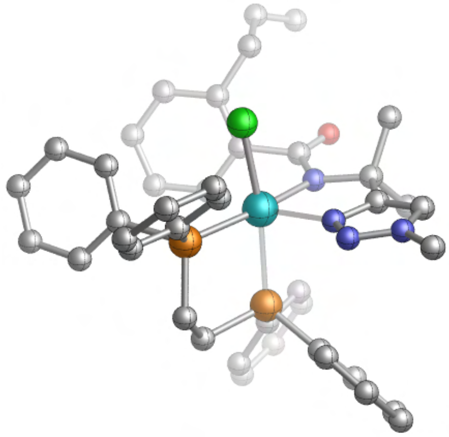

Zero-point correction= 0.755619 (Hartree/Particle)
Thermal correction to Energy= 0.805605
Thermal correction to Enthalpy= 0.806549
Thermal correction to Gibbs Free Energy= 0.665361
Sum of electronic and zero-point Energies= -4324.692438
Sum of electronic and thermal Energies= -4324.642453
Sum of electronic and thermal Enthalpies= -4324.641509
Sum of electronic and thermal Free Energies= -4324.782697

C	-20.33301700	1.62279400	-1.96944600
C	-19.57691300	2.14005100	-3.04811500
C	-19.79059000	1.62399800	-4.33616200
C	-20.73410000	0.62106000	-4.57890800
C	-21.47238100	0.10460800	-3.51541600
C	-21.26111800	0.60402300	-2.22821900
H	-19.24263900	2.04607500	-5.17617600
H	-20.88967200	0.25071700	-5.59548400
H	-22.20850000	-0.68572500	-3.68361600
H	-21.83467600	0.19019400	-1.39313300
C	-18.60870000	3.29191000	-2.81575800
O	-19.07349700	4.36409700	-2.39455100
C	-16.31396700	4.03304500	-2.57804300
C	-16.47647700	4.27089700	-1.05232100
H	-15.67266700	4.92402900	-0.67448200
H	-17.44320600	4.74643300	-0.85184600
H	-16.43041800	3.30683100	-0.52502500
C	-16.38067200	5.38099100	-3.33493700
H	-17.39072200	5.79693500	-3.23157000
H	-15.65516400	6.10104500	-2.92057700
H	-16.14461200	5.24635800	-4.40168000
C	-14.94084600	3.42508700	-2.77250200
N	-14.77209900	2.17215300	-3.26979500
N	-13.51209700	1.83591200	-3.26966100
C	-13.67713500	3.89218900	-2.45335800
H	-13.32411700	4.82569900	-2.02500900
N	-12.83772100	2.87109600	-2.77478600
N	-17.30471500	3.04710600	-3.07699400
Fe	-16.67916300	1.25622400	-3.71055100

P	-16.30954300	-0.91128300	-4.52699600
P	-16.39560900	1.89965200	-6.14361100
C	-15.68893700	-0.83080400	-6.28791500
H	-15.78491100	-1.80352100	-6.79471400
H	-14.60882000	-0.63701200	-6.20812400
C	-16.38580800	0.27703600	-7.08385300
H	-17.44636100	0.02184400	-7.24149000
H	-15.92100800	0.40480300	-8.07401600
C	-17.80512500	-1.99671600	-4.61063400
C	-18.47426500	-2.27693400	-3.40483500
C	-18.29424800	-2.55506500	-5.80355500
C	-19.58894000	-3.11705800	-3.39707700
H	-18.12496800	-1.81681600	-2.47692000
C	-19.41833000	-3.38819900	-5.79138500
H	-17.80607600	-2.35544900	-6.75934900
C	-20.06405400	-3.67700000	-4.58725000
H	-20.09556100	-3.32660300	-2.45172900
H	-19.78486800	-3.81478400	-6.72887900
H	-20.93912600	-4.33209700	-4.57725200
C	-15.05381700	-2.01880000	-3.74599500
C	-14.84501700	-3.31839400	-4.24200100
C	-14.27763400	-1.56261200	-2.67035100
C	-13.86463100	-4.14081500	-3.68382900
H	-15.46104800	-3.70070500	-5.06025600
C	-13.29930900	-2.39209900	-2.11117100
H	-14.46238200	-0.57254800	-2.25414200
C	-13.08754500	-3.67680000	-2.61653500
H	-13.71293900	-5.14952700	-4.07695700
H	-12.70598600	-2.03067900	-1.26716400
H	-12.32441300	-4.32270100	-2.17403400
C	-14.80841100	2.69697300	-6.69381500
C	-14.78893700	3.91289800	-7.40020800
C	-13.57933100	2.07702900	-6.39954400
C	-13.57989000	4.48234800	-7.81210700
H	-15.72290200	4.42240100	-7.64034400
C	-12.37373500	2.64037900	-6.82582200
H	-13.54714200	1.16076000	-5.81032900
C	-12.36822000	3.84579000	-7.53393800
H	-13.59078600	5.42772300	-8.36073400
H	-11.43283500	2.13293500	-6.59630600
H	-11.42466500	4.28728100	-7.86498100
C	-17.65390500	2.89981800	-7.05285700
C	-18.04647300	2.60834200	-8.37170300
C	-18.22519400	4.01149300	-6.40831300
C	-18.98799400	3.40779700	-9.02582000
H	-17.61723200	1.75973500	-8.90720600
C	-19.16131700	4.81344100	-7.06719300
H	-17.95051300	4.24018200	-5.37802000
C	-19.54657000	4.51264900	-8.37620500
H	-19.28452900	3.16509600	-10.04955000
H	-19.59969300	5.66640700	-6.54383600

H	-20.28519000	5.13439700	-8.88850100
Cl	-16.97085200	0.35354400	-1.59862500
C	-21.55747300	4.21066000	-0.14323200
H	-22.47083300	4.69774800	0.21257300
H	-20.78723400	4.83526700	-0.60543200
C	-21.40137900	2.88676900	-0.05715800
H	-22.21219600	2.28075600	0.36864500
C	-20.18024700	2.13101100	-0.54397800
H	-20.00131300	1.26129600	0.11033100
H	-19.29609400	2.77226200	-0.46638100
C	-11.39176100	2.81830200	-2.67068100
H	-10.92360100	3.45964300	-3.43301100
H	-11.07191100	3.14536000	-1.67109500
H	-11.08542900	1.77757600	-2.83082200

UB3LYP-D3/def2-SVP-SMD(THF)//UB3LYP/def2-SVP(gas)
HF= -4325.6574305
UPBEPBE-D3/def2-SVP-SMD(THF)//UB3LYP/def2-SVP(gas)
HF= -4322.3942079

^{5}E

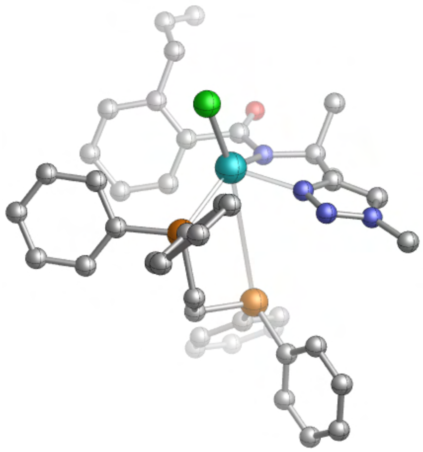

Zero-point correction= 0.754024 (Hartree/Particle)
Thermal correction to Energy= 0.805093
Thermal correction to Enthalpy= 0.806037
Thermal correction to Gibbs Free Energy= 0.658021
Sum of electronic and zero-point Energies= -4324.725262
Sum of electronic and thermal Energies= -4324.674193
Sum of electronic and thermal Enthalpies= -4324.673248
Sum of electronic and thermal Free Energies= -4324.821265

C	-20.58992500	1.09520300	-1.28047400
C	-19.74177500	1.72708700	-2.21678900
C	-19.75374100	1.30910800	-3.56009100
C	-20.60648900	0.29107900	-3.99238600
C	-21.44009800	-0.34203500	-3.06784100
C	-21.41851000	0.05478900	-1.72929600
H	-19.10582800	1.82264400	-4.27517500
H	-20.62105800	-0.00565100	-5.04432500
H	-22.10315200	-1.14999900	-3.38721200
H	-22.06483900	-0.45539900	-1.00873500
C	-18.87210300	2.91585900	-1.83952900
O	-19.40742500	3.96731100	-1.47312100
C	-16.60001100	3.78445000	-1.72267500
C	-16.68350400	4.28542900	-0.25703200
H	-15.92465700	5.06138600	-0.06331400
H	-17.68187400	4.70083900	-0.07360000
H	-16.51312300	3.44838700	0.43753000
C	-16.81591900	4.95333500	-2.70837500
H	-17.81886800	5.37166900	-2.55687600
H	-16.07246300	5.75223700	-2.54874200
H	-16.73199700	4.59259400	-3.74453400
C	-15.21468000	3.20609800	-1.92823100
N	-15.01138200	1.86119000	-1.97827300
N	-13.74341200	1.58328700	-2.11630800
C	-13.96609300	3.79156500	-2.03849500
H	-13.63743800	4.82664700	-2.04623900
N	-13.10020700	2.74728900	-2.14807900
N	-17.54506500	2.68088900	-1.97859100

Fe	-16.88840200	0.78174500	-1.88155000
P	-16.38507800	-1.20183500	-3.40586200
P	-16.03893400	1.81550500	-5.82417600
C	-15.72406300	-0.93659900	-5.13072100
H	-15.62508300	-1.92378900	-5.60870300
H	-14.69134200	-0.57863600	-4.99353100
C	-16.53523400	0.01385100	-6.03101400
H	-17.61157100	-0.05380500	-5.80356700
H	-16.41014700	-0.28404200	-7.08496700
C	-17.80809200	-2.35948800	-3.63142300
C	-18.54248500	-2.69982500	-2.48019100
C	-18.17681200	-2.92380500	-4.86388900
C	-19.60331700	-3.60350100	-2.56218900
H	-18.28501300	-2.24849500	-1.51765400
C	-19.24922500	-3.81897600	-4.94289400
H	-17.63431400	-2.67602100	-5.77824200
C	-19.96048000	-4.16600600	-3.79193100
H	-20.16012100	-3.86146300	-1.65790500
H	-19.52477000	-4.24829700	-5.90983200
H	-20.79484000	-4.86951900	-3.85385600
C	-15.07535100	-2.31552700	-2.71803500
C	-14.82497600	-3.57458900	-3.29279500
C	-14.30867800	-1.90299100	-1.61786400
C	-13.81463300	-4.39574300	-2.78947500
H	-15.43227000	-3.92764600	-4.13021700
C	-13.29869900	-2.72977600	-1.11406300
H	-14.51052600	-0.94595700	-1.13702500
C	-13.04705300	-3.97275200	-1.69851700
H	-13.63182500	-5.37275700	-3.24448900
H	-12.71367600	-2.39947500	-0.25195700
H	-12.26031500	-4.61843700	-1.29939500
C	-14.41716100	1.80310700	-6.73657900
C	-14.29477400	1.93351600	-8.13101300
C	-13.24598800	1.65144700	-5.97149800
C	-13.03793900	1.90559100	-8.74199700
H	-15.18862600	2.06309400	-8.74580500
C	-11.98939100	1.60737700	-6.58474800
H	-13.32253500	1.56522800	-4.88361400
C	-11.88212700	1.73898800	-7.97191600
H	-12.96095500	2.01274400	-9.82736800
H	-11.09040600	1.47507900	-5.97635400
H	-10.90066500	1.71484000	-8.45250000
C	-17.15316900	2.67489800	-7.02555800
C	-18.10748000	2.03994400	-7.83883500
C	-17.06683400	4.08024300	-7.07307500
C	-18.94328600	2.78682200	-8.67755800
H	-18.21152900	0.95353900	-7.82989900
C	-17.89032800	4.82409000	-7.91902100
H	-16.34505600	4.59757600	-6.43409700
C	-18.83526300	4.17813100	-8.72385500
H	-19.68225100	2.27381700	-9.29876400

H	-17.80371600	5.91347900	-7.94071300
H	-19.48955700	4.75924400	-9.37855100
Cl	-17.14875800	-0.33771700	0.06862600
C	-22.10102800	3.53992600	0.55442700
H	-23.06705300	3.98548300	0.81115900
H	-21.28543200	4.21293700	0.27330000
C	-21.93153800	2.21528400	0.54290200
H	-22.78225800	1.56442300	0.78411100
C	-20.63558600	1.51878700	0.17888900
H	-20.51025900	0.61858900	0.80377000
H	-19.78801100	2.17733300	0.40377600
C	-11.65682200	2.78084600	-2.30501300
H	-11.38529200	3.21571200	-3.27862600
H	-11.20135700	3.37382300	-1.49895600
H	-11.29507600	1.74722100	-2.24956700

UB3LYP-D3/def2-SVP-SMD(THF)//UB3LYP/def2-SVP(gas)
HF= -4325.6778229
UPBEPBE-D3/def2-SVP-SMD(THF)//UB3LYP/def2-SVP(gas)
HF= -4322.3919245

^{1}E’

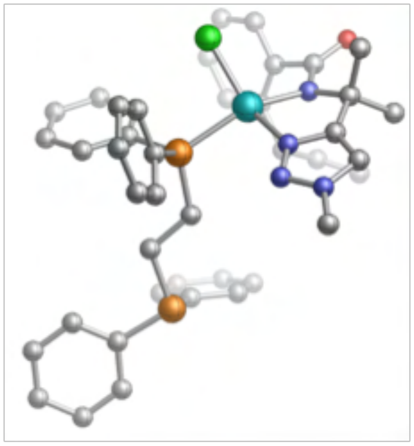

Zero-point correction= 0.754218 (Hartree/Particle)
Thermal correction to Energy= 0.805104
Thermal correction to Enthalpy= 0.806049
Thermal correction to Gibbs Free Energy= 0.660037
Sum of electronic and zero-point Energies= -4324.680100
Sum of electronic and thermal Energies= -4324.629214
Sum of electronic and thermal Enthalpies= -4324.628270
Sum of electronic and thermal Free Energies= -4324.774281

C	-20.91909700	-2.61483800	-3.36471500
C	-20.37271500	-1.78927500	-2.35673500
C	-20.71565900	-2.02292800	-1.01327500
C	-21.55945700	-3.06908700	-0.65089300
C	-22.09453900	-3.89801800	-1.64309800
C	-21.77429300	-3.66496400	-2.97990000
H	-20.29633200	-1.36605400	-0.24938200
H	-21.80038500	-3.23866900	0.40162300
H	-22.76399600	-4.72056300	-1.37625600
H	-22.20040400	-4.30941700	-3.75530800
C	-19.52391000	-0.55892300	-2.63120200
O	-19.96373000	0.53325800	-2.26848800
C	-17.56211400	0.42834200	-3.68101300
C	-17.00247100	1.18972100	-2.45248200
H	-16.35265500	2.02436100	-2.76385400
H	-17.84393000	1.58375800	-1.86863800
H	-16.42296200	0.50523600	-1.81476200
C	-18.36413700	1.38743900	-4.58841900
H	-19.22447100	1.78301000	-4.03672900
H	-17.73220200	2.23040900	-4.91427700
H	-18.72734900	0.86091100	-5.48429100
C	-16.40568200	-0.13009900	-4.47435000
N	-16.12448100	-1.46051100	-4.38038700
N	-15.09552900	-1.78233700	-5.11960400
C	-15.46306400	0.40042600	-5.33350300
H	-15.28842200	1.40631100	-5.70386200
N	-14.68578800	-0.66050000	-5.69535600
N	-18.33511500	-0.76903700	-3.26725500
Fe	-17.28828400	-2.42520200	-3.10677500

Cl	-17.25474700	-2.90614500	-0.91098000
C	-21.75729100	-0.55768200	-6.26476000
H	-20.79167700	-0.08071700	-6.46121600
H	-22.64008700	-0.08823400	-6.70817900
C	-21.85646300	-1.65207900	-5.50477300
H	-22.84962200	-2.08316600	-5.32338600
C	-20.70190000	-2.37668600	-4.85314800
H	-20.59476100	-3.36055100	-5.34709800
H	-19.76987000	-1.82319900	-5.02544500
C	-13.55872800	-0.68318900	-6.61247200
H	-12.85321700	0.11983900	-6.35747000
H	-13.06326500	-1.65530900	-6.50632500
H	-13.90133200	-0.55478800	-7.65031400
P	-16.66428400	-4.62525200	-3.74222600
C	-16.95436600	-4.88967900	-5.56668600
H	-16.28832100	-4.17254200	-6.07509700
H	-17.97750700	-4.51117100	-5.72822600
C	-16.82155500	-6.29780400	-6.15252700
H	-17.53115700	-6.98296300	-5.66411900
H	-15.80890000	-6.69996300	-5.98882900
P	-17.09369900	-6.28236700	-8.01536300
C	-18.93459200	-6.02475900	-8.07133200
C	-19.40251800	-4.73589800	-8.38169500
C	-19.87462900	-7.03338200	-7.79491600
C	-20.77270600	-4.45250100	-8.39228100
H	-18.68467100	-3.94530100	-8.61909600
C	-21.24356900	-6.75533000	-7.81910800
H	-19.53513100	-8.04585600	-7.56258400
C	-21.69572300	-5.46398200	-8.11307500
H	-21.11872300	-3.44066300	-8.61785300
H	-21.96212200	-7.55103000	-7.60477900
H	-22.76705100	-5.24758800	-8.12767000
C	-16.94257700	-8.07660000	-8.45096400
C	-17.01527800	-8.40147000	-9.81957900
C	-16.70546200	-9.11428400	-7.53329200
C	-16.87736500	-9.72014900	-10.25502600
H	-17.18174900	-7.60698300	-10.55337900
C	-16.55317000	-10.43545100	-7.97083400
H	-16.64070200	-8.90615700	-6.46379900
C	-16.64224800	-10.74350500	-9.32991500
H	-16.94429900	-9.94960000	-11.32181100
H	-16.36807800	-11.22774800	-7.24049600
H	-16.52527900	-11.77593300	-9.66912300
C	-17.50944100	-6.03546700	-2.91397000
C	-18.75847700	-5.82089800	-2.31122500
C	-16.94595500	-7.32423900	-2.88364600
C	-19.44304300	-6.88280300	-1.71205500
H	-19.19139700	-4.82035900	-2.28380300
C	-17.63015600	-8.38094800	-2.27878700
H	-15.96053200	-7.50200100	-3.32019000
C	-18.88319800	-8.16235300	-1.69574200

H	-20.41279400	-6.69653200	-1.24483600
H	-17.18010100	-9.37698100	-2.25665500
H	-19.41640800	-8.98895400	-1.21868400
C	-14.87555900	-4.94653900	-3.43985200
C	-13.92833300	-5.10178900	-4.46472000
C	-14.43946000	-4.96095400	-2.10105700
C	-12.57697900	-5.29526800	-4.15839200
H	-14.23177000	-5.06783000	-5.51218600
C	-13.08956600	-5.15319000	-1.80065300
H	-15.16073400	-4.79570200	-1.29639400
C	-12.15476400	-5.32590300	-2.82705700
H	-11.85286400	-5.42444600	-4.96733300
H	-12.76638300	-5.16149500	-0.75655100
H	-11.09883100	-5.47866400	-2.58914200

UB3LYP-D3/def2-SVP-SMD(THF)//UB3LYP/def2-SVP(gas)
HF= -4325.6005993
UPBEPBE-D3/def2-SVP-SMD(THF)//UB3LYP/def2-SVP(gas)
HF= -4322.3366750

^{3}E'

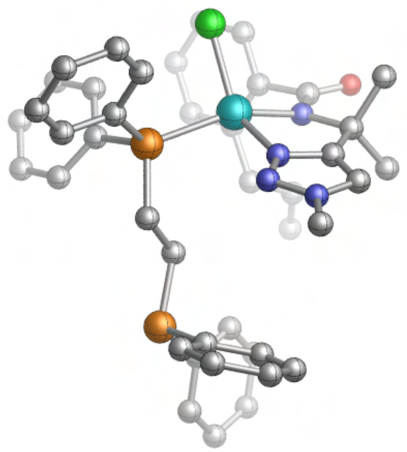

Zero-point correction= 0.754524 (Hartree/Particle)
Thermal correction to Energy= 0.805302
Thermal correction to Enthalpy= 0.806246
Thermal correction to Gibbs Free Energy= 0.659804
Sum of electronic and zero-point Energies= -4324.698540
Sum of electronic and thermal Energies= -4324.647762
Sum of electronic and thermal Enthalpies= -4324.646818
Sum of electronic and thermal Free Energies= -4324.793260

C	-21.07854100	-2.60436200	-5.39439700
C	-20.57903800	-1.88122800	-4.28981500
C	-21.15221200	-2.07097600	-3.02230200
C	-22.18390200	-2.98821700	-2.83161000
C	-22.66831200	-3.72271600	-3.91893500
C	-22.12069200	-3.52277400	-5.18578800
H	-20.76666900	-1.49843600	-2.17738200
H	-22.60938300	-3.13032200	-1.83478700
H	-23.47840600	-4.44414000	-3.78201900
H	-22.51468500	-4.07990300	-6.04081700
C	-19.55795700	-0.76965700	-4.46356700
O	-19.97681300	0.39164400	-4.46805100
C	-17.27803500	-0.06728000	-4.92853900
C	-17.07925800	0.83968900	-3.68922900
H	-16.28709000	1.58628200	-3.86545100
H	-18.02124600	1.35998000	-3.47446800
H	-16.80512400	0.22982000	-2.81505900
C	-17.64040500	0.78193300	-6.17035600
H	-18.57361800	1.32538700	-5.98627900
H	-16.83976900	1.50855900	-6.38686200
H	-17.77038700	0.13852900	-7.05451000
C	-15.98145700	-0.78470700	-5.21460500
N	-15.90686000	-2.13113400	-5.03059300
N	-14.70952900	-2.58455500	-5.30508000
C	-14.72582500	-0.38757400	-5.63517900
H	-14.31061000	0.57958500	-5.90252600
N	-13.99183000	-1.53598500	-5.67243400
N	-18.26182000	-1.14529900	-4.65153400

Fe	-17.53544900	-2.91345300	-4.20140600
P	-17.04775000	-5.20488900	-4.60395700
C	-16.23547900	-5.46939000	-6.26033300
H	-16.06870200	-6.54707400	-6.41741200
H	-15.24168200	-5.00637500	-6.16072700
C	-18.50650700	-6.33707200	-4.60918400
C	-19.46921500	-6.17130000	-3.59697100
C	-18.68458900	-7.35888600	-5.55911600
C	-20.57395000	-7.02380800	-3.53052400
H	-19.35189700	-5.36842300	-2.86414200
C	-19.79663200	-8.20366100	-5.49148000
H	-17.96217000	-7.51055400	-6.36354800
C	-20.74134600	-8.04081100	-4.47471500
H	-21.31296500	-6.88151100	-2.73844500
H	-19.92232600	-8.99168500	-6.23860700
H	-21.60996000	-8.70250100	-4.42283200
C	-15.85001200	-6.02840800	-3.46201200
C	-15.30309700	-5.30918800	-2.38814600
C	-15.46359900	-7.36612600	-3.65773700
C	-14.37123700	-5.91408500	-1.53783600
H	-15.63825600	-4.28899400	-2.19324300
C	-14.53182400	-7.96592300	-2.80853700
H	-15.90100900	-7.95419700	-4.46862400
C	-13.98026700	-7.23831100	-1.74806400
H	-13.95811600	-5.34611200	-0.70026500
H	-14.24105500	-9.00719300	-2.97042000
H	-13.25420100	-7.70950100	-1.08025900
Cl	-18.15600700	-3.21278600	-2.03973100
C	-22.04060900	-1.26194400	-8.58787400
H	-22.13474000	-2.20029700	-9.14597200
H	-22.56122900	-0.39005200	-8.99436100
C	-21.33414500	-1.18708300	-7.45522100
H	-21.27911600	-0.23674300	-6.91019300
C	-20.59626600	-2.33775200	-6.81212800
H	-20.72104700	-3.24462600	-7.42594200
H	-19.51938300	-2.10183900	-6.78473500
P	-16.34207800	-5.43956400	-9.09165500
C	-17.38625800	-4.49992100	-10.29723800
C	-17.25269300	-4.85139100	-11.65445200
C	-18.32131600	-3.50832200	-9.95463500
C	-18.01095200	-4.21575700	-12.63915900
H	-16.54601100	-5.63612400	-11.94114200
C	-19.09119600	-2.87873300	-10.94009700
H	-18.46481200	-3.21220100	-8.91445100
C	-18.93520300	-3.22698400	-12.28376300
H	-17.88770700	-4.50062400	-13.68741900
H	-19.81333200	-2.11282400	-10.64592700
H	-19.53613900	-2.73428900	-13.05242000
C	-17.00148000	-4.84774700	-7.43460500
H	-16.98363600	-3.74899600	-7.35922800
H	-18.05968100	-5.15827500	-7.41842600

C	-14.72211300	-4.52664100	-9.20355400
C	-13.55230200	-5.23976900	-8.88615400
C	-14.59806200	-3.19140200	-9.62742500
C	-12.29367000	-4.63569800	-8.97778800
H	-13.62745400	-6.28740400	-8.58071200
C	-13.34043600	-2.58993900	-9.73234700
H	-15.49080000	-2.62120500	-9.89538900
C	-12.18446700	-3.30999400	-9.40878300
H	-11.39597400	-5.21016200	-8.73369300
H	-13.26218000	-1.55673700	-10.08253300
H	-11.20036000	-2.84348600	-9.50695100
C	-12.58275000	-1.69681800	-5.99493100
H	-12.43657600	-2.69726100	-6.41870300
H	-12.29490800	-0.94294000	-6.73828400
H	-11.96738200	-1.57988000	-5.09009900

UB3LYP-D3/def2-SVP-SMD(THF)//UB3LYP/def2-SVP(gas)
HF= -4325.6474694
UPBEPBE-D3/def2-SVP-SMD(THF)//UB3LYP/def2-SVP(gas)
HF= -4322.3772520

^{5}E'

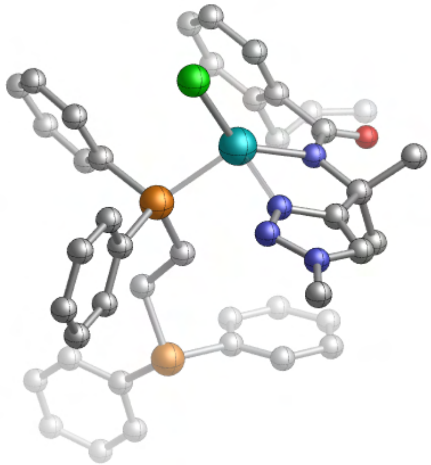

Zero-point correction= 0.753774 (Hartree/Particle)
Thermal correction to Energy= 0.804916
Thermal correction to Enthalpy= 0.805860
Thermal correction to Gibbs Free Energy= 0.656868
Sum of electronic and zero-point Energies= -4324.730699
Sum of electronic and thermal Energies= -4324.679556
Sum of electronic and thermal Enthalpies= -4324.678612
Sum of electronic and thermal Free Energies= -4324.827605

C	-21.67446500	-1.80436500	-2.11402200
C	-20.67995900	-0.83054300	-1.85314000
C	-20.19643900	-0.67609800	-0.54068700
C	-20.65000500	-1.48408600	0.50491700
C	-21.60701000	-2.46431800	0.24425800
C	-22.10729000	-2.61337900	-1.05171000
H	-19.46800200	0.10773200	-0.32486400
H	-20.24346400	-1.34716500	1.50925800
H	-21.96397200	-3.11632800	1.04547500
H	-22.85692500	-3.38588200	-1.24918900
C	-20.13243000	0.07204400	-2.94573100
O	-20.91174900	0.68004500	-3.68936900
C	-18.14171200	1.00879500	-3.98992300
C	-18.48355100	2.49016000	-3.70402400
H	-17.96895100	3.15958200	-4.41304600
H	-19.56640400	2.63859100	-3.80001000
H	-18.17429100	2.76075900	-2.68277800
C	-18.53205300	0.63745500	-5.44303900
H	-19.61159600	0.77030200	-5.57908700
H	-17.99376100	1.27371400	-6.16465100
H	-18.27519500	-0.41293000	-5.65067800
C	-16.64198600	0.82037100	-3.85545200
N	-16.11567800	-0.12828900	-3.03215300
N	-14.81358100	-0.12910200	-3.08576000
C	-15.56641200	1.44556400	-4.46100000
H	-15.49505600	2.25405800	-5.18273500
N	-14.46975000	0.82033200	-3.94965500

N	-18.77463400	0.11358600	-2.99601900
Fe	-17.60764000	-1.03024800	-1.78719700
Cl	-16.77283500	-0.95504300	0.32820300
C	-24.05722300	-0.28313400	-4.09458500
H	-23.29668200	0.38237600	-4.51214100
H	-25.09719600	0.05743400	-4.09850900
C	-23.71752900	-1.45672700	-3.55563900
H	-24.49490700	-2.08186800	-3.09652900
C	-22.30316600	-1.99772600	-3.48712500
H	-22.32837700	-3.07951700	-3.70904100
H	-21.69492500	-1.51314600	-4.25950000
C	-13.06718100	1.08027600	-4.22177100
H	-12.79399200	2.09534500	-3.89742700
H	-12.48279100	0.34476100	-3.65650000
H	-12.85905200	0.97060500	-5.29606500
P	-17.47220900	-3.46072800	-2.41017900
C	-18.22866500	-3.78985900	-4.07969200
H	-17.56801700	-3.20577700	-4.74478200
H	-19.18293300	-3.24087300	-4.08216800
C	-18.43601400	-5.21925700	-4.62582000
H	-19.46486600	-5.55967600	-4.43755500
H	-17.76845300	-5.94393300	-4.13691100
P	-18.07948500	-5.30959300	-6.47089000
C	-19.45980800	-4.25676100	-7.13031000
C	-19.12162700	-3.04943400	-7.76322800
C	-20.81977600	-4.59284800	-6.99971400
C	-20.11658100	-2.18806700	-8.24036900
H	-18.06782300	-2.78284200	-7.88422900
C	-21.81295500	-3.73511000	-7.47670100
H	-21.10528500	-5.53672800	-6.52722400
C	-21.46308000	-2.52867300	-8.09532000
H	-19.83726000	-1.24951500	-8.72583400
H	-22.86602200	-4.00592200	-7.36461400
H	-22.24234300	-1.85656700	-8.46316000
C	-18.66869600	-7.01317500	-6.89693000
C	-18.83715000	-7.32514200	-8.26053800
C	-18.85542200	-8.03811600	-5.95349000
C	-19.19779000	-8.61082500	-8.66459700
H	-18.68834400	-6.54823200	-9.01615600
C	-19.20878600	-9.33044200	-6.36012100
H	-18.72783200	-7.84363200	-4.88694100
C	-19.38418600	-9.62127600	-7.71409200
H	-19.33032400	-8.82711500	-9.72801200
H	-19.35013500	-10.11171800	-5.60849600
H	-19.66256100	-10.62992500	-8.02967400
C	-18.22906900	-4.59571800	-1.16209100
C	-17.75958800	-4.50650400	0.16259600
C	-19.29269600	-5.47035600	-1.43813100
C	-18.30680000	-5.30781200	1.16602200
H	-16.99043200	-3.77843100	0.42563400
C	-19.84467600	-6.26495000	-0.42785700

H	-19.71033700	-5.53768400	-2.44078100
C	-19.34655000	-6.19610000	0.87438100
H	-17.92536100	-5.22396400	2.18682200
H	-20.67060300	-6.94037100	-0.66605900
H	-19.77526800	-6.82095000	1.66212100
C	-15.70274100	-3.98980800	-2.54286700
C	-15.19208700	-4.76880600	-3.59636500
C	-14.81376200	-3.53016900	-1.55242600
C	-13.83669000	-5.11086900	-3.63606400
H	-15.83286700	-5.10663100	-4.41032000
C	-13.46355300	-3.88661100	-1.58917800
H	-15.16805200	-2.85907000	-0.76553100
C	-12.97100500	-4.68304300	-2.62657200
H	-13.46050900	-5.71689600	-4.46442200
H	-12.79160300	-3.52201800	-0.80803700
H	-11.91327900	-4.95769500	-2.65645000

UB3LYP-D3/def2-SVP-SMD(THF)//UB3LYP/def2-SVP(gas)
HF= -4325.6731347
UPBEPBE-D3/def2-SVP-SMD(THF)//UB3LYP/def2-SVP(gas)
HF= -4322.3890755

^{1}E'-complex

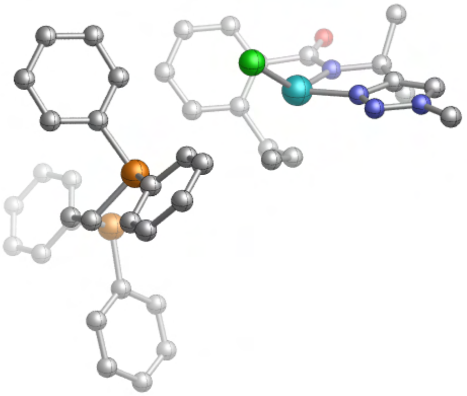

Zero-point correction= 0.753917 (Hartree/Particle)
Thermal correction to Energy= 0.804573
Thermal correction to Enthalpy= 0.805517
Thermal correction to Gibbs Free Energy= 0.656231
Sum of electronic and zero-point Energies= -4324.666754
Sum of electronic and thermal Energies= -4324.616098
Sum of electronic and thermal Enthalpies= -4324.615154
Sum of electronic and thermal Free Energies= -4324.764440

C	-18.12133600	-0.89239700	-5.75831500
C	-18.70625900	-0.28558600	-4.61967400
C	-19.96378200	-0.72148300	-4.16884200
C	-20.61984100	-1.77979100	-4.79278900
C	-20.03025800	-2.40526500	-5.89704100
C	-18.80144000	-1.95496000	-6.37968500
H	-20.41721600	-0.21098100	-3.31609700
H	-21.58996000	-2.11859700	-4.42036300
H	-20.53083300	-3.24464500	-6.38618000
H	-18.35850100	-2.43062200	-7.25950600
C	-18.04468400	0.85534800	-3.86335100
O	-18.62605300	1.92653600	-3.69081500
C	-16.00835600	1.42969400	-2.59301800
C	-16.74294000	1.87861500	-1.30650100
H	-16.09680000	2.52844800	-0.69385200
H	-17.65132500	2.42826300	-1.58349900
H	-17.02865800	1.00177300	-0.70570900
C	-15.58268500	2.66103000	-3.42291200
H	-16.48219700	3.21058900	-3.73131000
H	-14.93961200	3.33501100	-2.83342400
H	-15.02889800	2.35276300	-4.32296000
C	-14.79798700	0.60914000	-2.20583600
N	-14.75246000	-0.70706700	-2.57154000
N	-13.66137600	-1.28272000	-2.15175800
C	-13.63476200	0.84666600	-1.50113900
H	-13.23659200	1.73265900	-1.01494200
N	-12.97818700	-0.34951600	-1.50167800
N	-16.82957900	0.49480100	-3.38422500
Fe	-16.27369400	-1.33345800	-3.60880200
P	-16.65019800	-5.83472600	-6.69462700

P	-17.69437700	-4.21740300	-9.76769300
C	-16.68027600	-6.57138100	-8.41633800
H	-16.74731800	-7.67141000	-8.37547000
H	-15.69585000	-6.34099800	-8.85675300
C	-17.80866500	-6.03672300	-9.31439300
H	-18.78333300	-6.14199800	-8.80935900
H	-17.88255200	-6.64470300	-10.23208500
C	-18.13587400	-6.62120000	-5.89154600
C	-18.75084500	-5.90423200	-4.84965100
C	-18.66581400	-7.87239000	-6.25224000
C	-19.86164900	-6.43161200	-4.18188300
H	-18.34923400	-4.93528500	-4.54039300
C	-19.78392200	-8.39142200	-5.59412700
H	-18.20339600	-8.46100400	-7.04840400
C	-20.38457100	-7.67207200	-4.55537800
H	-20.31766300	-5.86350100	-3.36683100
H	-20.18378400	-9.36547300	-5.88946100
H	-21.25681800	-8.08082900	-4.03811300
C	-15.26612200	-6.78337700	-5.89217400
C	-14.92123100	-6.40264800	-4.58008500
C	-14.52066500	-7.80071000	-6.51132800
C	-13.87890600	-7.04011700	-3.90390100
H	-15.46790000	-5.59702400	-4.07932100
C	-13.46746800	-8.42838100	-5.83602500
H	-14.75168600	-8.12101800	-7.52904400
C	-13.14601700	-8.05461600	-4.52938500
H	-13.63479800	-6.73348100	-2.88323800
H	-12.89936800	-9.21762500	-6.33650600
H	-12.32565000	-8.54861600	-4.00181000
C	-16.32595600	-4.17703300	-11.02105200
C	-15.83268900	-2.90533100	-11.36923700
C	-15.73376000	-5.30361500	-11.61761900
C	-14.79981800	-2.76131400	-12.29774600
H	-16.26694200	-2.01607100	-10.90258500
C	-14.68877400	-5.16247300	-12.53784600
H	-16.08491900	-6.30815600	-11.37246800
C	-14.22143600	-3.89218900	-12.88370100
H	-14.43807600	-1.76295600	-12.55782200
H	-14.24051100	-6.05239600	-12.98796000
H	-13.40585200	-3.78324900	-13.60325000
C	-19.20792500	-4.09250300	-10.84305000
C	-19.27180000	-4.54415900	-12.17261000
C	-20.35664100	-3.51528200	-10.27374900
C	-20.45553900	-4.43137200	-12.90711700
H	-18.38803400	-4.98179500	-12.64318500
C	-21.54386200	-3.41002600	-11.00508200
H	-20.31904100	-3.13537900	-9.24872600
C	-21.59536800	-3.86733600	-12.32468100
H	-20.48738000	-4.78489800	-13.94135700
H	-22.42720800	-2.95968000	-10.54465800
H	-22.51988300	-3.77865600	-12.90125200

Cl	-16.69724000	-3.37685100	-2.80041100
C	-15.28408000	-2.17340400	-5.34111700
H	-15.93363600	-2.98903400	-5.67287700
H	-14.29280200	-2.46594100	-4.98620900
C	-15.57652600	-0.85479000	-5.60074800
H	-14.79180000	-0.10512700	-5.45272000
C	-16.81345900	-0.38208400	-6.34693700
H	-16.82311100	0.71788300	-6.35497200
H	-16.74795100	-0.70640900	-7.39939300
C	-11.70071500	-0.67945800	-0.89191700
H	-11.75359600	-0.55042700	0.19902000
H	-11.48573600	-1.72832000	-1.12724300
H	-10.90650500	-0.03763200	-1.29995800

UB3LYP-D3/def2-SVP-SMD(THF)//UB3LYP/def2-SVP(gas)
HF= -4325.5951978
UPBEPBE-D3/def2-SVP-SMD(THF)//UB3LYP/def2-SVP(gas)
HF= -4322.3461246

[3]E'-complex

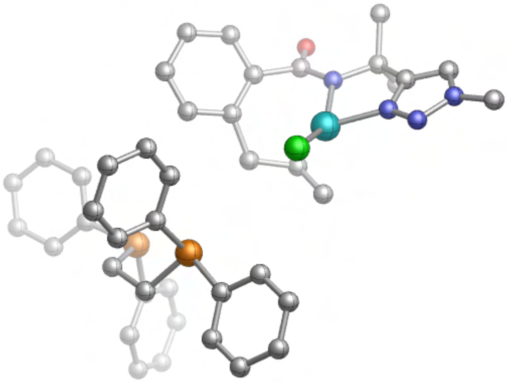

Zero-point correction= 0.753643 (Hartree/Particle)
Thermal correction to Energy= 0.804473
Thermal correction to Enthalpy= 0.805417
Thermal correction to Gibbs Free Energy= 0.653177
Sum of electronic and zero-point Energies= -4324.675474
Sum of electronic and thermal Energies= -4324.624645
Sum of electronic and thermal Enthalpies= -4324.623700
Sum of electronic and thermal Free Energies= -4324.775940

C	-18.19826000	-1.37116400	-5.68416300
C	-18.48597300	-0.22165700	-4.91401600
C	-19.82938100	0.03568200	-4.56016400
C	-20.86357500	-0.82782300	-4.90064000
C	-20.57527400	-1.98262200	-5.63541300
C	-19.26343400	-2.23188900	-6.02334200
H	-20.04364500	0.94957900	-4.00297000
H	-21.88864100	-0.60231700	-4.59557700
H	-21.36866900	-2.68299500	-5.90898100
H	-19.03643700	-3.12364600	-6.61338100
C	-17.53828900	0.85379200	-4.37817800
O	-17.80463200	2.03197900	-4.62071900
C	-15.85398200	1.44640000	-2.70520300
C	-16.83215000	2.29286100	-1.85793100
H	-16.27707800	2.97280300	-1.19111600
H	-17.47330000	2.88974200	-2.51677200
H	-17.46488900	1.63713400	-1.24065700
C	-14.95907500	2.36600800	-3.57218700
H	-15.59210100	2.91458400	-4.28134900
H	-14.40914700	3.08756500	-2.94606100
H	-14.22278600	1.77042500	-4.13453900
C	-14.97582700	0.64943400	-1.76637200
N	-14.80957800	-0.68741100	-1.97582100
N	-14.01503000	-1.21521300	-1.08692100
C	-14.21581800	0.95918600	-0.65494200
H	-14.04278800	1.88519400	-0.11441600
N	-13.64957600	-0.22501700	-0.28390100
N	-16.56216200	0.43179600	-3.52379100
Fe	-15.86295500	-1.37599800	-3.53344100
P	-16.44821300	-5.92860500	-6.88857400

P	-17.70967800	-4.19036700	-9.82251000
C	-16.64236900	-6.59877100	-8.62708700
H	-16.72162900	-7.69864100	-8.61995600
H	-15.69901300	-6.36277700	-9.14722200
C	-17.84026000	-6.01515800	-9.39594000
H	-18.76197400	-6.10431300	-8.79690500
H	-18.02442600	-6.60189600	-10.31198500
C	-17.85581600	-6.74982200	-5.98425900
C	-18.42472500	-6.04498700	-4.90881300
C	-18.36850300	-8.01967300	-6.30266900
C	-19.47696400	-6.59817900	-4.17049400
H	-18.03046100	-5.06400900	-4.63001100
C	-19.42662200	-8.56655200	-5.57261700
H	-17.93771500	-8.60018500	-7.12231400
C	-19.98396500	-7.85631200	-4.50350900
H	-19.89977000	-6.03721500	-3.33299300
H	-19.81408500	-9.55475400	-5.83563000
H	-20.81027800	-8.28634200	-3.93107300
C	-14.99858700	-6.90512000	-6.25584600
C	-14.53567400	-6.57639000	-4.96624200
C	-14.31172400	-7.89314200	-6.98101800
C	-13.43545400	-7.23504500	-4.41354400
H	-15.03771500	-5.79411200	-4.38811100
C	-13.20084700	-8.54251200	-6.42998700
H	-14.63401800	-8.17261700	-7.98585800
C	-12.76182000	-8.22007500	-5.14405400
H	-13.09866300	-6.96879300	-3.40803700
H	-12.67961700	-9.30804900	-7.01164100
H	-11.89588100	-8.73049800	-4.71410900
C	-16.48560500	-4.17207500	-11.21905100
C	-15.99830300	-2.90934300	-11.60651700
C	-15.99231900	-5.30586700	-11.88749500
C	-15.06908000	-2.78052000	-12.64063600
H	-16.35440100	-2.01495400	-11.08650300
C	-15.05006300	-5.18033000	-12.91482700
H	-16.34113900	-6.30423100	-11.61519000
C	-14.58873000	-3.91849200	-13.29732500
H	-14.71070100	-1.78861900	-12.92860100
H	-14.67768200	-6.07594500	-13.41958900
H	-13.85348900	-3.82163200	-14.10034800
C	-19.32238500	-3.98351900	-10.72758200
C	-19.56599400	-4.46985400	-12.02391200
C	-20.35555000	-3.30438300	-10.05877400
C	-20.81297700	-4.29171300	-12.62881000
H	-18.77270700	-4.98505200	-12.57132000
C	-21.60617100	-3.13184000	-10.66090500
H	-20.17636800	-2.89866300	-9.05903900
C	-21.83718600	-3.62492200	-11.94777400
H	-20.98544800	-4.67344200	-13.63886500
H	-22.39783800	-2.60129200	-10.12526700
H	-22.81147100	-3.48445900	-12.42334200

Cl	-16.14224100	-3.52980400	-3.02973300
C	-14.50781700	-1.76431000	-5.30972900
H	-14.46742100	-2.85589700	-5.28265400
H	-13.57827300	-1.22682400	-5.10076500
C	-15.61388600	-1.09970000	-5.75484400
H	-15.54332200	-0.01658800	-5.89129200
C	-16.85042400	-1.76348500	-6.28544200
H	-16.90139700	-1.54318200	-7.36963700
H	-16.73708400	-2.85651300	-6.22153800
C	-12.76224700	-0.49070500	0.83622900
H	-13.26807100	-0.26356900	1.78599700
H	-12.50285000	-1.55532200	0.80359700
H	-11.84923800	0.11634900	0.75188200

UB3LYP-D3/def2-SVP-SMD(THF)//UB3LYP/def2-SVP(gas)
HF= -4325.6061767
UPBEPBE-D3/def2-SVP-SMD(THF)//UB3LYP/def2-SVP(gas)
HF= -4322.3446017

[5]E'-complex

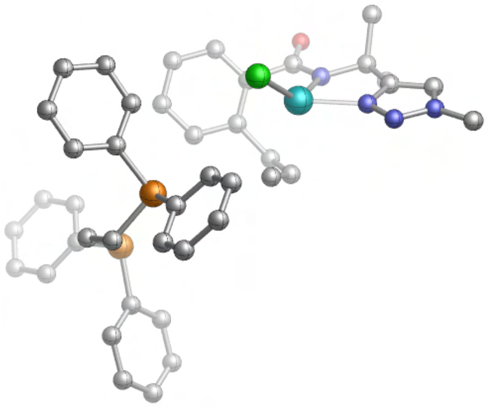

Zero-point correction= 0.753044 (Hartree/Particle)
Thermal correction to Energy= 0.804325
Thermal correction to Enthalpy= 0.805270
Thermal correction to Gibbs Free Energy= 0.652744
Sum of electronic and zero-point Energies= -4324.714058
Sum of electronic and thermal Energies= -4324.662777
Sum of electronic and thermal Enthalpies= -4324.661832
Sum of electronic and thermal Free Energies= -4324.814358

C	-17.99522400	-0.78102400	-5.78818500
C	-18.61881200	-0.36974000	-4.58612400
C	-19.87259200	-0.90124600	-4.24132100
C	-20.48712900	-1.86842000	-5.03379700
C	-19.85966300	-2.29963500	-6.20662500
C	-18.63438700	-1.74797600	-6.58344500
H	-20.35911800	-0.53721800	-3.33361200
H	-21.45402200	-2.28477100	-4.73999100
H	-20.32654000	-3.06411900	-6.83261200
H	-18.16449000	-2.07632600	-7.51527200
C	-18.03140100	0.68025500	-3.65437500
O	-18.69369800	1.68192200	-3.37950700
C	-16.09666700	1.29744000	-2.28803700
C	-16.86313000	1.48352500	-0.95490000
H	-16.30892900	2.14498900	-0.26898600
H	-17.84665600	1.92340400	-1.16378400
H	-17.00582000	0.51021600	-0.46054300
C	-15.86178300	2.66730300	-2.96543800
H	-16.83009600	3.12480300	-3.20359400
H	-15.30224300	3.34566600	-2.30058700
H	-15.28605700	2.54372500	-3.89604200
C	-14.75010800	0.66301400	-1.98364600
N	-14.46747700	-0.61632200	-2.36353800
N	-13.26935500	-0.96597800	-1.98651200
C	-13.62809100	1.12272700	-1.31823500
H	-13.39178000	2.06956000	-0.84137300
N	-12.75297400	0.07912900	-1.34922600
N	-16.79809800	0.35747500	-3.18381300
Fe	-16.12358000	-1.52090600	-3.35865300

P	-16.70120000	-5.83839600	-6.74016900
P	-17.70798200	-4.24794700	-9.87521900
C	-16.75571000	-6.58617700	-8.45637900
H	-16.83883000	-7.68447900	-8.40284900
H	-15.77140100	-6.37565000	-8.90665700
C	-17.88275200	-6.04322000	-9.35152300
H	-18.85093000	-6.09549100	-8.82603900
H	-17.99563100	-6.68090700	-10.24472900
C	-18.18441900	-6.60749000	-5.91709200
C	-18.81709600	-5.85785200	-4.90992200
C	-18.69418500	-7.87998500	-6.22954900
C	-19.92617500	-6.37178100	-4.22896900
H	-18.43075200	-4.87261100	-4.63679100
C	-19.81018100	-8.38677900	-5.55908200
H	-18.21615300	-8.49432800	-6.99664500
C	-20.42902400	-7.63332900	-4.55547100
H	-20.39535800	-5.77766600	-3.44052300
H	-20.19425000	-9.37780700	-5.81632300
H	-21.29956300	-8.03262600	-4.02812400
C	-15.31526700	-6.79393200	-5.94959500
C	-14.96725000	-6.42554300	-4.63480100
C	-14.56794400	-7.80251400	-6.58082500
C	-13.92029600	-7.06489300	-3.96784100
H	-15.51669700	-5.62969800	-4.12188100
C	-13.51018700	-8.43261000	-5.91476800
H	-14.80077200	-8.11400700	-7.60080800
C	-13.18545200	-8.07024100	-4.60579500
H	-13.67504400	-6.76742500	-2.94479100
H	-12.94091300	-9.21489600	-6.42470600
H	-12.36138100	-8.56598600	-4.08580300
C	-16.35658700	-4.30985200	-11.14740200
C	-15.82402300	-3.07201900	-11.55473700
C	-15.81310400	-5.48030000	-11.70460700
C	-14.80100900	-3.00307500	-12.50252400
H	-16.21954500	-2.14948200	-11.11928000
C	-14.77792900	-5.41437000	-12.64422600
H	-16.19484200	-6.46095500	-11.41289100
C	-14.27163800	-4.17690200	-13.04904100
H	-14.40847300	-2.02975900	-12.80882500
H	-14.36805700	-6.33745400	-13.06306900
H	-13.46386400	-4.12672900	-13.78372300
C	-19.22741000	-4.10656200	-10.94129400
C	-19.34390900	-4.66984700	-12.22403800
C	-20.32317100	-3.39956900	-10.41660300
C	-20.52768200	-4.53901200	-12.95499800
H	-18.50026700	-5.20914400	-12.66182900
C	-21.51110200	-3.27421800	-11.14419800
H	-20.24308500	-2.93394600	-9.43032400
C	-21.61539200	-3.84364500	-12.41592300
H	-20.60065000	-4.98080900	-13.95254400
H	-22.35296300	-2.72179000	-10.71856200

H	-22.54011500	-3.74091300	-12.98981700
Cl	-16.69168500	-3.54202600	-2.58781000
C	-15.30993500	-2.23301300	-5.59892500
H	-16.13322400	-2.93278300	-5.77280100
H	-14.34424600	-2.67253800	-5.33260500
C	-15.42817900	-0.90491300	-5.80086800
H	-14.53634000	-0.27731100	-5.68203300
C	-16.67986300	-0.18128300	-6.25756100
H	-16.60989100	0.86977300	-5.94415300
H	-16.66771300	-0.17066700	-7.36302900
C	-11.41334400	0.00245500	-0.79232100
H	-11.44363000	0.14223900	0.29817800
H	-11.01861000	-0.99388500	-1.02357800
H	-10.76660000	0.76989800	-1.24216500

UB3LYP-D3/def2-SVP-SMD(THF)//UB3LYP/def2-SVP(gas)
HF= -4325.6470436
UPBEPBE-D3/def2-SVP-SMD(THF)//UB3LYP/def2-SVP(gas)
HF= -4322.3629501

[1]E’-noligand

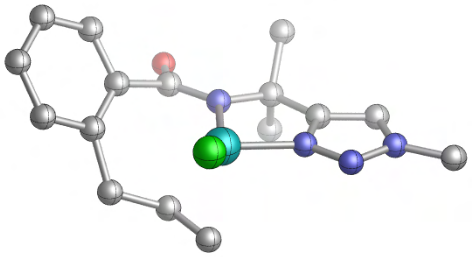

Zero-point correction= 0.331151 (Hartree/Particle)
Thermal correction to Energy= 0.354077
Thermal correction to Enthalpy= 0.355021
Thermal correction to Gibbs Free Energy= 0.278258
Sum of electronic and zero-point Energies= -2638.048298
Sum of electronic and thermal Energies= -2638.025372
Sum of electronic and thermal Enthalpies= -2638.024428
Sum of electronic and thermal Free Energies= -2638.101190

C	-18.54506100	-1.33514700	-5.81629500
C	-18.84469700	-0.32785500	-4.86866700
C	-20.16342400	-0.24328800	-4.36821600
C	-21.15723600	-1.13975500	-4.73935900
C	-20.85524900	-2.15233400	-5.65574100
C	-19.57196500	-2.23064600	-6.18242300
H	-20.38870200	0.56547500	-3.67095500
H	-22.16213500	-1.04982700	-4.31922000
H	-21.61765100	-2.87288000	-5.96248000
H	-19.34302200	-3.01145300	-6.91346200
C	-17.93731400	0.75248400	-4.27613800
O	-18.34835600	1.91487800	-4.27271200
C	-15.99631600	1.28947400	-2.88665600
C	-16.80536500	1.90020900	-1.71974500
H	-16.16739500	2.55578100	-1.10445300
H	-17.63801900	2.49049300	-2.12079100
H	-17.20905900	1.10224300	-1.07818900
C	-15.41174400	2.41046600	-3.78129800
H	-16.23561100	2.97801100	-4.23178200
H	-14.78216500	3.09509500	-3.18989800
H	-14.78944600	1.98281200	-4.58302100
C	-14.85587500	0.47414600	-2.32170800
N	-14.68171300	-0.80810200	-2.75245000
N	-13.64658500	-1.36589200	-2.18922100
C	-13.83902200	0.71403100	-1.41799900
H	-13.57429200	1.58094000	-0.81955100
N	-13.12989700	-0.45046000	-1.37907800
N	-16.80468500	0.31738700	-3.66157300
Fe	-16.08083100	-1.44296200	-3.99193100
Cl	-16.24504200	-3.64032600	-3.76529800
C	-14.81790400	-1.55709000	-5.80356100
H	-14.73846300	-2.64079500	-5.91676100
H	-13.89028200	-1.01086900	-5.61409400
C	-15.97989500	-0.88430400	-6.08090000

H	-15.95230700	0.20828300	-6.11297400
C	-17.23766000	-1.53331200	-6.59213300
H	-17.40931500	-1.15712300	-7.61983200
H	-17.06634600	-2.61675200	-6.68270900
C	-11.95957300	-0.76984300	-0.57854100
H	-11.14387700	-0.06595900	-0.79834800
H	-12.20515200	-0.72472200	0.49258900
H	-11.65023500	-1.78819100	-0.84131700

UB3LYP-D3/def2-SVP-SMD(THF)//UB3LYP/def2-SVP(gas)
HF= -2638.4576064
UPBEPBE-D3/def2-SVP-SMD(THF)//UB3LYP/def2-SVP(gas)
HF= -2636.8781503

^{3}E'-noligand

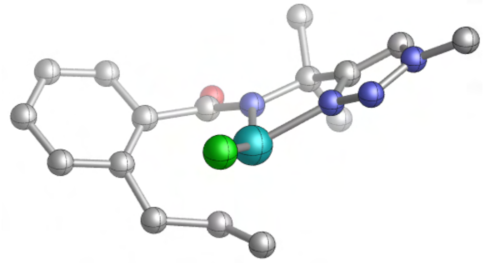

Zero-point correction= 0.331218 (Hartree/Particle)
Thermal correction to Energy= 0.354060
Thermal correction to Enthalpy= 0.355005
Thermal correction to Gibbs Free Energy= 0.277782
Sum of electronic and zero-point Energies= -2638.064772
Sum of electronic and thermal Energies= -2638.041930
Sum of electronic and thermal Enthalpies= -2638.040986
Sum of electronic and thermal Free Energies= -2638.118208

C	-18.62725300	-1.41149900	-5.77713600
C	-18.81052700	-0.34714600	-4.86528000
C	-20.09212300	-0.16668000	-4.29619500
C	-21.15244300	-1.02333100	-4.56395700
C	-20.96298800	-2.09052600	-5.44774900
C	-19.72004900	-2.26165900	-6.04464300
H	-20.23585900	0.68471100	-3.62910700
H	-22.12356100	-0.85708900	-4.09087100
H	-21.78104300	-2.77825200	-5.67668400
H	-19.57603700	-3.08230100	-6.75390700
C	-17.82371000	0.71911400	-4.37422700
O	-18.19881600	1.89246400	-4.40761300
C	-15.91065500	1.26792700	-2.95814100
C	-16.77912200	1.90680100	-1.84990700
H	-16.16278800	2.54894600	-1.19970400
H	-17.56955100	2.51791300	-2.30012300
H	-17.24098500	1.12306900	-1.23040000
C	-15.25894800	2.36717400	-3.83348000
H	-16.04819400	2.93405600	-4.34326600
H	-14.65764900	3.05681500	-3.21877400
H	-14.59632900	1.91568700	-4.58861500
C	-14.81589500	0.46065000	-2.29742800
N	-14.56037200	-0.80439600	-2.73478100
N	-13.57882400	-1.35063900	-2.07271000
C	-13.91006900	0.70425500	-1.28305500
H	-13.73657400	1.56104000	-0.63858700
N	-13.17788500	-0.44328200	-1.19280800
N	-16.67500700	0.28888700	-3.77398000
Fe	-15.81119800	-1.39857900	-4.17696800
Cl	-15.73568600	-3.60695500	-3.99279500
C	-14.87231200	-1.41327200	-6.20825800
H	-14.66577400	-2.48332300	-6.28273900
H	-14.01005300	-0.74052300	-6.19027800
C	-16.14137500	-0.91244500	-6.31129600

H	-16.25979500	0.17310200	-6.38007300
C	-17.37018200	-1.72938300	-6.58382700
H	-17.62703600	-1.59031800	-7.65265400
H	-17.13327500	-2.79815400	-6.45948300
C	-12.09154400	-0.75234800	-0.27782300
H	-12.44838000	-0.72243600	0.76201900
H	-11.73941700	-1.76269700	-0.51649700
H	-11.26960000	-0.03306200	-0.40508800

UB3LYP-D3/def2-SVP-SMD(THF)//UB3LYP/def2-SVP(gas)
HF= -2638.4864615
UPBEPBE-D3/def2-SVP-SMD(THF)//UB3LYP/def2-SVP(gas)
HF= -2636.9006439

^{5}E'-noligand

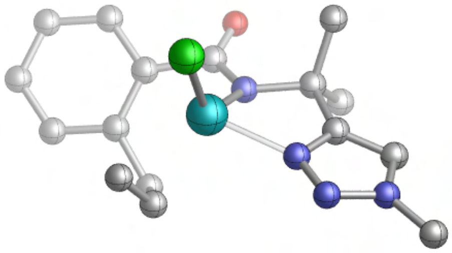

Zero-point correction= 0.330903 (Hartree/Particle)
Thermal correction to Energy= 0.353991
Thermal correction to Enthalpy= 0.354935
Thermal correction to Gibbs Free Energy= 0.275920
Sum of electronic and zero-point Energies= -2638.103388
Sum of electronic and thermal Energies= -2638.080301
Sum of electronic and thermal Enthalpies= -2638.079356
Sum of electronic and thermal Free Energies= -2638.158371

C	-18.17471900	-1.03636400	-5.99258700
C	-18.79574500	-0.29924000	-4.95517500
C	-20.19348000	-0.37185000	-4.81040600
C	-20.97258000	-1.18271200	-5.63132000
C	-20.36179300	-1.91612300	-6.65290400
C	-18.98140000	-1.82929400	-6.82931600
H	-20.65568100	0.24431900	-4.03709100
H	-22.05460100	-1.23232700	-5.48544600
H	-20.95923200	-2.54501100	-7.31803800
H	-18.50824600	-2.38623900	-7.64376000
C	-18.09718200	0.69413400	-4.03273600
O	-18.67069900	1.76342500	-3.81159300
C	-16.12769400	1.28588400	-2.71770200
C	-16.81703600	1.55560800	-1.35649200
H	-16.21723100	2.23983200	-0.73428500
H	-17.80190600	2.00515800	-1.53908200
H	-16.95215000	0.61012700	-0.80836000
C	-15.89010600	2.61052600	-3.47739100
H	-16.84949500	3.09991900	-3.67821600
H	-15.26198700	3.29116700	-2.87966500
H	-15.37945500	2.42090400	-4.43461500
C	-14.77360200	0.64604900	-2.45370100
N	-14.57839800	-0.69209900	-2.62480000
N	-13.35916100	-1.03706800	-2.31865600
C	-13.56843100	1.15269900	-2.00228400
H	-13.24584600	2.15374000	-1.73179100
N	-12.73919300	0.07333300	-1.93686100
N	-16.90194300	0.30265800	-3.51420100
Fe	-16.41272000	-1.61067100	-3.15169100
Cl	-17.02518300	-2.94667000	-1.48512900
C	-16.46036300	-3.15871200	-4.86117600
H	-17.53609800	-3.35285000	-4.89049200
H	-15.83640200	-3.92902400	-4.39919400
C	-15.90597900	-2.08759900	-5.48541100

H	-14.81345700	-2.00409900	-5.52725100
C	-16.67899700	-1.02877000	-6.24750500
H	-16.24384000	-0.04594100	-6.01763800
H	-16.49424100	-1.20386300	-7.32392800
C	-11.34744300	0.02459600	-1.52255400
H	-11.24589900	0.37492500	-0.48510300
H	-11.02095100	-1.01975900	-1.59065800
H	-10.72832000	0.64960000	-2.18268700

UB3LYP-D3/def2-SVP-SMD(THF)//UB3LYP/def2-SVP(gas)
HF= -2638.5271763
UPBEPBE-D3/def2-SVP-SMD(THF)//UB3LYP/def2-SVP(gas)
HF= -2636.9233447

¹E''

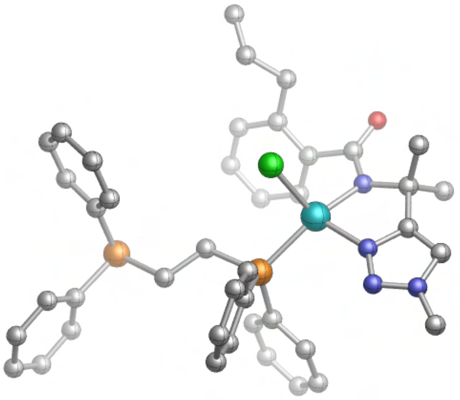

Zero-point correction= 0.754280 (Hartree/Particle)
Thermal correction to Energy= 0.805173
Thermal correction to Enthalpy= 0.806117
Thermal correction to Gibbs Free Energy= 0.658898
Sum of electronic and zero-point Energies= -4324.689798
Sum of electronic and thermal Energies= -4324.638904
Sum of electronic and thermal Enthalpies= -4324.637960
Sum of electronic and thermal Free Energies= -4324.785179

C	-18.79321300	-0.14358100	-2.65471600
C	-18.65463000	1.26383300	-2.66017000
C	-19.36212100	2.03260900	-3.59895600
C	-20.22402100	1.43055800	-4.52214400
C	-20.36277900	0.04225000	-4.52145800
C	-19.64676800	-0.72912700	-3.59932600
H	-19.25596600	3.11999100	-3.57924500
H	-20.78738200	2.04600500	-5.22876100
H	-21.02619500	-0.44664400	-5.23985500
H	-19.75206500	-1.81646600	-3.61170100
C	-17.82570400	1.98150200	-1.61164000
O	-18.07470800	1.81623000	-0.41593900
C	-16.09583100	3.67231200	-1.23805100
C	-15.20405700	2.84159000	-0.28135000
H	-14.56155600	3.49571500	0.33103800
H	-15.84851700	2.24647800	0.37818200
H	-14.56597700	2.15756900	-0.86129000
C	-16.99268200	4.64378800	-0.44037600
H	-17.65653600	4.06695900	0.21543200
H	-16.38442800	5.32366000	0.17916500
H	-17.60730100	5.24913600	-1.12453300
C	-15.20222900	4.46074800	-2.16824800
N	-15.10345900	4.08243500	-3.47617700
N	-14.27300900	4.85033700	-4.12987000
C	-14.35783400	5.54350600	-2.01831200
H	-14.10708400	6.16803900	-1.16578800
N	-13.81421000	5.73517000	-3.25419100
N	-16.87812200	2.80331700	-2.13832800
Fe	-16.17454400	2.46788100	-3.92087200
P	-16.30950100	2.57605500	-6.29137800

C	-17.17514700	0.87578200	-8.48041500
H	-17.56672600	1.76766300	-8.99654900
H	-16.15466400	0.72471900	-8.86636000
C	-17.17184900	1.07237000	-6.96265400
H	-16.67066800	0.24266500	-6.43926100
H	-18.19304200	1.09481000	-6.54642400
C	-14.70780600	2.63789000	-7.20996400
C	-14.56317300	3.28662800	-8.44852400
C	-13.60105500	1.97206800	-6.65311500
C	-13.33472600	3.27309200	-9.11526600
H	-15.41016300	3.80906200	-8.89794800
C	-12.37672400	1.95598600	-7.32797800
H	-13.70830700	1.45358700	-5.69645600
C	-12.23890300	2.60792000	-8.55686700
H	-13.23649300	3.78135500	-10.07815700
H	-11.52540700	1.43167900	-6.88594700
H	-11.27934100	2.59690800	-9.08065300
C	-17.25880000	4.03428600	-6.90059300
C	-18.58406600	3.93257600	-7.35711500
C	-16.67943200	5.31374500	-6.79502100
C	-19.30504300	5.07739900	-7.71418100
H	-19.07017500	2.95854700	-7.43420800
C	-17.39986900	6.45351400	-7.15672600
H	-15.65733700	5.41523400	-6.42156900
C	-18.71554600	6.33971000	-7.61905800
H	-20.33376500	4.97723800	-8.06968600
H	-16.93263000	7.43846400	-7.07374500
H	-19.27935300	7.23270800	-7.90040000
Cl	-15.37678000	0.32551900	-3.95198900
C	-12.86631900	6.75271200	-3.67539300
H	-13.33254500	7.74901400	-3.64957500
H	-11.98633400	6.74359600	-3.01667200
H	-12.55952400	6.51584700	-4.70093300
P	-18.32135300	-0.50900900	-9.03446200
C	-18.03841300	-0.40089300	-10.87003900
C	-18.96652400	0.34535300	-11.61802800
C	-16.96627300	-1.00877300	-11.54530300
C	-18.81767300	0.49902100	-12.99950800
H	-19.82081300	0.80449300	-11.11111600
C	-16.82224200	-0.86414000	-12.92830700
H	-16.23973100	-1.60473100	-10.98795900
C	-17.74456800	-0.10782100	-13.65859600
H	-19.54855800	1.08448400	-13.56387100
H	-15.98398700	-1.34632700	-13.43867200
H	-17.63076300	0.00233600	-14.74025800
C	-17.39706400	-2.04880800	-8.58075500
C	-18.11894400	-3.25557600	-8.64767100
C	-16.06203900	-2.09450900	-8.14452800
C	-17.52048200	-4.47155300	-8.31219700
H	-19.16640100	-3.23830400	-8.96354700
C	-15.46600900	-3.31058500	-7.79231200

H	-15.46943800	-1.18062100	-8.06968600
C	-16.19055600	-4.50170900	-7.87948700
H	-18.09809100	-5.39754600	-8.37605900
H	-14.42963800	-3.32260000	-7.44504300
H	-15.72367800	-5.45042600	-7.60247900
C	-18.15052400	-3.51991600	-1.51770600
H	-18.77125700	-3.49224200	-0.61523000
H	-17.87440300	-4.50770600	-1.89844500
C	-17.74162400	-2.39845100	-2.11897400
H	-17.12024500	-2.46489400	-3.02116700
C	-18.03577300	-1.00069400	-1.65144400
H	-17.07702600	-0.50403700	-1.43352100
H	-18.59155700	-1.01549700	-0.69849900

UB3LYP-D3/def2-SVP-SMD(THF)//UB3LYP/def2-SVP(gas)
HF= -4325.6078871
UPBEPBE-D3/def2-SVP-SMD(THF)//UB3LYP/def2-SVP(gas)
HF= -4322.3429146

^{3}E''

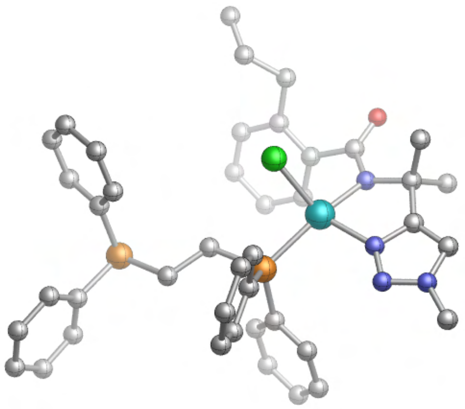

Zero-point correction= 0.754349 (Hartree/Particle)
Thermal correction to Energy= 0.805185
Thermal correction to Enthalpy= 0.806129
Thermal correction to Gibbs Free Energy= 0.657465
Sum of electronic and zero-point Energies= -4324.704087
Sum of electronic and thermal Energies= -4324.653251
Sum of electronic and thermal Enthalpies= -4324.652306
Sum of electronic and thermal Free Energies= -4324.800971

C	-18.79800600	-0.19425500	-2.60999900
C	-18.59813300	1.20588300	-2.60316200
C	-19.30675400	2.01573800	-3.50663900
C	-20.21903700	1.46373800	-4.41242800
C	-20.41307000	0.08238300	-4.42772900
C	-19.70311200	-0.72958200	-3.53660500
H	-19.15981200	3.09764400	-3.47170800
H	-20.77889600	2.11263000	-5.09154200
H	-21.11656700	-0.36932200	-5.13230200
H	-19.85232900	-1.81167200	-3.56089900
C	-17.69841000	1.87573000	-1.58365200
O	-17.85397600	1.64230100	-0.38269400
C	-16.02548300	3.62299300	-1.22656200
C	-15.01238400	2.80443700	-0.38812200
H	-14.36950700	3.46407900	0.21817300
H	-15.56875900	2.13087800	0.27638400
H	-14.37749400	2.19700300	-1.05076500
C	-16.92074300	4.48352700	-0.30676800
H	-17.49322600	3.82765100	0.36003100
H	-16.31036600	5.16860200	0.30490000
H	-17.62419300	5.08197700	-0.90616700
C	-15.26439800	4.53251400	-2.16310000
N	-15.24425000	4.25034500	-3.49814200
N	-14.51501800	5.11483400	-4.15344500
C	-14.48327600	5.65974000	-1.99817300
H	-14.20840600	6.24310800	-1.12425100
N	-14.04996700	5.96948400	-3.25380200
N	-16.81132000	2.75596700	-2.12449900
Fe	-16.17882600	2.53832300	-3.95227100

P	-16.27014800	2.59966200	-6.31109700
C	-17.15294400	0.89475300	-8.48451700
H	-17.52076200	1.79126600	-9.01004400
H	-16.13386000	0.71678200	-8.86277900
C	-17.15424000	1.10189600	-6.96822100
H	-16.67052900	0.26917700	-6.43409000
H	-18.17677800	1.14447900	-6.55678400
C	-14.65155400	2.60579700	-7.20129400
C	-14.46011500	3.23483400	-8.44346100
C	-13.57779900	1.91278800	-6.61286300
C	-13.21764900	3.17760800	-9.08149900
H	-15.28131500	3.77542000	-8.91840000
C	-12.33925300	1.85363100	-7.25867900
H	-13.72326400	1.40408200	-5.65562600
C	-12.15456700	2.48781800	-8.49076800
H	-13.08281500	3.67110700	-10.04762400
H	-11.51422600	1.30893500	-6.79218300
H	-11.18406100	2.44318600	-8.99205500
C	-17.19069400	4.05645300	-6.96966200
C	-18.52434600	3.95763500	-7.40355800
C	-16.58937600	5.32911700	-6.93069100
C	-19.23083500	5.09656800	-7.80454700
H	-19.02881300	2.99029800	-7.42881500
C	-17.29569000	6.46313800	-7.33660100
H	-15.56412000	5.43230100	-6.56882000
C	-18.61878100	6.35147800	-7.77691900
H	-20.26594600	4.99753000	-8.14129500
H	-16.81067100	7.44252300	-7.30516500
H	-19.17094300	7.24006800	-8.09323600
Cl	-15.52806200	0.34595400	-3.96726600
C	-13.21072100	7.08120200	-3.66823800
H	-13.75673700	8.03204100	-3.57616200
H	-12.30435500	7.11813200	-3.04774200
H	-12.93214500	6.91644600	-4.71566800
P	-18.32807700	-0.46660600	-9.03602600
C	-18.02890100	-0.37944200	-10.87023400
C	-18.92659700	0.39272200	-11.62905900
C	-16.97381700	-1.02761500	-11.53475000
C	-18.76397900	0.53226500	-13.01044400
H	-19.76813800	0.88370000	-11.13070300
C	-16.81642000	-0.89711400	-12.91775200
H	-16.27124400	-1.64397500	-10.96896300
C	-17.70796200	-0.11487000	-13.65879600
H	-19.47111200	1.13839200	-13.58320000
H	-15.99182500	-1.41079300	-13.41958900
H	-17.58379600	-0.01577600	-14.74039000
C	-17.44534700	-2.02494200	-8.56360000
C	-18.19761700	-3.21341100	-8.62185100
C	-16.11351400	-2.10049600	-8.12185400
C	-17.63167500	-4.44101900	-8.27253300
H	-19.24322300	-3.17230500	-8.94160000

C	-15.54999700	-3.32795000	-7.75600300
H	-15.49840900	-1.20110300	-8.05330000
C	-16.30449700	-4.50097400	-7.83461600
H	-18.23261800	-5.35249100	-8.32953200
H	-14.51548800	-3.36327900	-7.40476300
H	-15.86304500	-5.45859500	-7.54677000
C	-18.18344600	-3.63010600	-1.64301900
H	-18.79640900	-3.64545200	-0.73490700
H	-17.91709100	-4.59859600	-2.07669500
C	-17.77209800	-2.48140000	-2.18886800
H	-17.15965900	-2.50489800	-3.09925500
C	-18.05248400	-1.10732500	-1.64760400
H	-17.09258800	-0.63263800	-1.39392200
H	-18.61489000	-1.17022000	-0.70022100

UB3LYP-D3/def2-SVP-SMD(THF)//UB3LYP/def2-SVP(gas)
HF= -4325.6452571
UPBEPBE-D3/def2-SVP-SMD(THF)//UB3LYP/def2-SVP(gas)
HF= -4322.3753752

^{5}E''

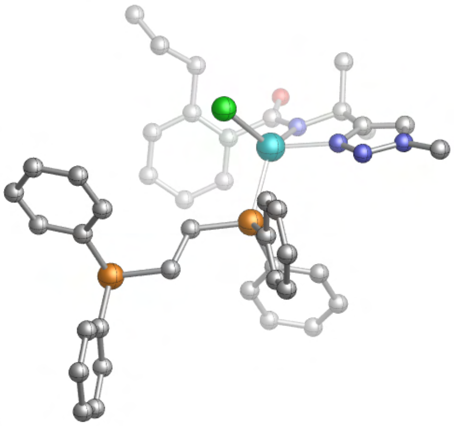

Zero-point correction= 0.753568 (Hartree/Particle)
Thermal correction to Energy= 0.804827
Thermal correction to Enthalpy= 0.805771
Thermal correction to Gibbs Free Energy= 0.654931
Sum of electronic and zero-point Energies= -4324.734624
Sum of electronic and thermal Energies= -4324.683365
Sum of electronic and thermal Enthalpies= -4324.682421
Sum of electronic and thermal Free Energies= -4324.833261

C	-18.75067800	-0.03188000	-2.59972200
C	-18.77771800	1.37133100	-2.76888900
C	-19.51843000	1.93657700	-3.81907100
C	-20.25571400	1.12984500	-4.69152400
C	-20.23278000	-0.25693900	-4.52848000
C	-19.48005000	-0.82555500	-3.49615600
H	-19.53346700	3.02375300	-3.93447100
H	-20.84947800	1.58392800	-5.48942800
H	-20.79961000	-0.90047000	-5.20642500
H	-19.46153700	-1.91128400	-3.37646000
C	-18.10827700	2.29664000	-1.76363300
O	-18.62469900	2.44487200	-0.65426800
C	-16.27523300	3.86240900	-1.38211300
C	-15.76476000	3.21334800	-0.07064400
H	-15.19860500	3.94020800	0.53476100
H	-16.62232200	2.84936300	0.50888500
H	-15.10367700	2.36364700	-0.30167000
C	-17.16719500	5.08446200	-1.06819300
H	-18.04403200	4.75391000	-0.49721500
H	-16.61944500	5.83463100	-0.47396200
H	-17.50734400	5.55822700	-2.00203900
C	-15.06872600	4.31954400	-2.17968700
N	-14.69750100	3.67998100	-3.32440200
N	-13.62281500	4.21554000	-3.83463200
C	-14.13800400	5.32309500	-1.97850900
H	-14.02472600	6.07722400	-1.20510400
N	-13.27338200	5.20980900	-3.02481200
N	-16.98135400	2.88605200	-2.23422800
Fe	-16.04929500	2.07604500	-3.81852300

P	-16.37838500	2.43510200	-6.27921200
C	-17.22144500	0.82292200	-8.54938400
H	-17.65998600	1.72390300	-9.00857200
H	-16.20315200	0.73812300	-8.96011700
C	-17.19805000	0.93950200	-7.02357800
H	-16.65993700	0.09781600	-6.55789500
H	-18.21204500	0.90344400	-6.59151700
C	-14.81567400	2.62287300	-7.24597800
C	-14.73698100	3.36961200	-8.43445400
C	-13.66941000	1.95340100	-6.78139700
C	-13.53515800	3.44547900	-9.14381100
H	-15.61543000	3.89841800	-8.81022600
C	-12.47245900	2.02494100	-7.50031200
H	-13.71729600	1.36733900	-5.85960400
C	-12.40090500	2.77216000	-8.67942500
H	-13.48777700	4.02971600	-10.06659400
H	-11.59003900	1.49708500	-7.12951000
H	-11.46232500	2.83084300	-9.23693400
C	-17.39507300	3.89519400	-6.74902600
C	-18.71188000	3.78953800	-7.22697900
C	-16.86683800	5.17854400	-6.50788100
C	-19.47509400	4.93703000	-7.47050100
H	-19.15799400	2.81044600	-7.40918700
C	-17.62842100	6.32133500	-6.75638700
H	-15.84847300	5.28211300	-6.12346300
C	-18.93665100	6.20448600	-7.23926700
H	-20.49717200	4.83493500	-7.84409400
H	-17.20041100	7.30930100	-6.56747300
H	-19.53426800	7.09921200	-7.43060100
Cl	-15.08419600	0.02408900	-3.92452900
C	-12.09121900	6.00222300	-3.31495200
H	-12.35697500	7.06493000	-3.41271400
H	-11.34435800	5.88439600	-2.51597900
H	-11.67611300	5.63714500	-4.26182000
P	-18.31795200	-0.58036600	-9.15652100
C	-18.06975700	-0.37032100	-10.98790200
C	-19.02628700	0.39555600	-11.67831800
C	-17.00041400	-0.92155000	-11.71414400
C	-18.90732500	0.62436900	-13.05212900
H	-19.87938200	0.81038000	-11.13260500
C	-16.88660100	-0.70215900	-13.09021000
H	-16.25190000	-1.53154400	-11.20312900
C	-17.83646300	0.07357900	-13.76226700
H	-19.65979100	1.22431500	-13.57105200
H	-16.05000300	-1.14099100	-13.64079300
H	-17.74596000	0.24240000	-14.83852700
C	-17.32189400	-2.09799000	-8.78812800
C	-18.00012700	-3.32864500	-8.87204700
C	-15.97076500	-2.10697100	-8.40152300
C	-17.34469200	-4.53134100	-8.60069000
H	-19.05826800	-3.34090200	-9.15019000

C	-15.31716400	-3.31014200	-8.11342200
H	-15.41000100	-1.17379800	-8.31774300
C	-15.99973500	-4.52469800	-8.21650900
H	-17.88916400	-5.47627600	-8.67674000
H	-14.26888600	-3.29417100	-7.80414800
H	-15.48790600	-5.46348300	-7.98968600
C	-17.88552300	-3.11720200	-0.92045400
H	-18.56666900	-3.00926900	-0.06901600
H	-17.50369100	-4.12188100	-1.12448500
C	-17.53026700	-2.06894300	-1.66942800
H	-16.84460600	-2.21189800	-2.51393300
C	-17.97547100	-0.65081900	-1.44583800
H	-17.07801600	-0.03442800	-1.27003900
H	-18.58267000	-0.57224800	-0.52752100

UB3LYP-D3/def2-SVP-SMD(THF)//UB3LYP/def2-SVP(gas)
HF= -4325.6743694
UPBEPBE-D3/def2-SVP-SMD(THF)//UB3LYP/def2-SVP(gas)
HF= -4322.3899661

Figure S32
Dimethyl allyl radical

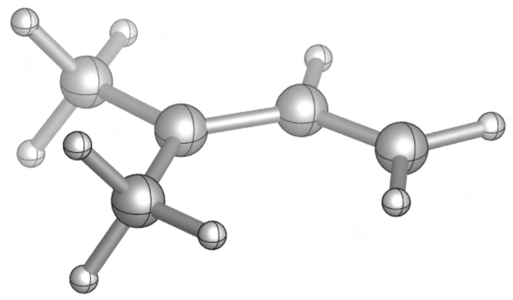

Zero-point correction= 0.121656 (Hartree/Particle)
Thermal correction to Energy= 0.128437
Thermal correction to Enthalpy= 0.129381
Thermal correction to Gibbs Free Energy= 0.091107
Sum of electronic and zero-point Energies= -195.636829
Sum of electronic and thermal Energies= -195.630048
Sum of electronic and thermal Enthalpies= -195.629104
Sum of electronic and thermal Free Energies= -195.667378

C	-7.27192600	-0.59393800	0.22416200
C	-6.90329100	-0.13420300	-1.04718100
H	-7.72961100	0.01147400	-1.75470200
C	-8.71231400	-0.85862800	0.55437300
H	-8.86918500	-1.91224300	0.85502600
H	-9.04719600	-0.24695700	1.41383600
H	-9.38018300	-0.64568800	-0.29375500
C	-6.28669100	-0.85411400	1.32623400
H	-6.52349300	-0.24449500	2.21820500
H	-6.33490100	-1.90860600	1.65677300
H	-5.24814300	-0.64016700	1.04063300
C	-5.63321000	0.15676600	-1.51720500
H	-5.48775200	0.51064500	-2.54005300
H	-4.73866100	0.04795200	-0.89962700

UB3LYP-D3/def2-SVP-SMD(THF)//UB3LYP/def2-SVP(gas)
HF= -195.7693236
UPBEPBE-D3/def2-SVP-SMD(THF)//UB3LYP/def2-SVP(gas)
HF= -195.4821463

^{1}C'-outer-TS-dimethyl

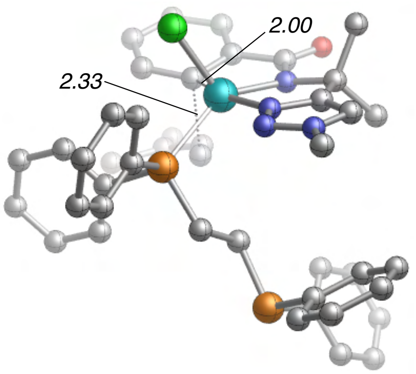

Zero-point correction= 0.806590 (Hartree/Particle)
Thermal correction to Energy= 0.859206
Thermal correction to Enthalpy= 0.860150
Thermal correction to Gibbs Free Energy= 0.714006
Sum of electronic and zero-point Energies= -4403.107720
Sum of electronic and thermal Energies= -4403.055104
Sum of electronic and thermal Enthalpies= -4403.054159
Sum of electronic and thermal Free Energies= -4403.200303

C	-20.39583600	-2.55229200	-4.32561400
C	-20.84782000	-1.21281300	-4.56598800
C	-22.11168800	-0.76046900	-4.18772800
C	-22.98657400	-1.63597800	-3.54048000
C	-22.56928300	-2.94851300	-3.24136500
C	-21.30651600	-3.38921100	-3.61536400
H	-22.38392800	0.27610400	-4.40240900
H	-23.98312000	-1.29831800	-3.24159400
H	-23.24240300	-3.61855900	-2.69767400
H	-21.01007300	-4.40888000	-3.36504300
C	-19.87091500	-0.28479700	-5.20057900
O	-20.17569200	0.84041200	-5.60599100
C	-17.46223500	0.02649800	-5.52654200
C	-17.39304100	1.23857000	-4.56288900
H	-16.48374400	1.83221300	-4.75242200
H	-18.27414600	1.87604700	-4.70690200
H	-17.37183800	0.88736500	-3.52016000
C	-17.47854200	0.50686400	-6.99500200
H	-18.37408900	1.11874500	-7.16125600
H	-16.58388600	1.11015900	-7.22263400
H	-17.50161700	-0.35189400	-7.68388200
C	-16.25600100	-0.84294300	-5.27526600
N	-16.42290600	-2.07813700	-4.72301100
N	-15.27071700	-2.67353200	-4.56800800
C	-14.89683600	-0.67409200	-5.45800900
H	-14.30351200	0.14251700	-5.85787700
N	-14.34252900	-1.83287000	-5.00683800
N	-18.61495400	-0.84424300	-5.22250200
Fe	-18.39221300	-2.56789500	-4.40635000

P	-17.80216400	-4.95758100	-4.88972300
C	-16.97752700	-5.11742700	-6.56583000
H	-16.91189500	-6.18281300	-6.83961100
H	-15.94592800	-4.77701500	-6.38502900
C	-19.12432900	-6.25300700	-4.89708200
C	-19.69876900	-6.58081700	-3.65333400
C	-19.57940100	-6.92016600	-6.04553200
C	-20.69257900	-7.55621600	-3.56297900
H	-19.35634100	-6.06487100	-2.75176600
C	-20.58248200	-7.89400000	-5.95351700
H	-19.15136200	-6.70014300	-7.02568800
C	-21.13937500	-8.21618000	-4.71416800
H	-21.12072700	-7.80269300	-2.58791400
H	-20.92067800	-8.40692600	-6.85777700
H	-21.91662200	-8.98129600	-4.64281700
C	-16.54214600	-5.82887600	-3.83505400
C	-15.98620700	-5.21117800	-2.70546100
C	-16.13113400	-7.13202000	-4.17413900
C	-15.03020700	-5.88637000	-1.93710300
H	-16.32577400	-4.21636400	-2.41191800
C	-15.17085400	-7.79734600	-3.41009300
H	-16.57187700	-7.64473700	-5.03293100
C	-14.61599800	-7.17294700	-2.28727600
H	-14.61281800	-5.39767800	-1.05258500
H	-14.86256800	-8.80923200	-3.68661100
H	-13.86951200	-7.69548200	-1.68272500
Cl	-18.30165600	-2.78530700	-2.11828300
C	-23.43958400	-3.58004400	-6.63888700
C	-22.27430900	-2.86524200	-6.76396200
H	-22.35983300	-1.83585600	-7.12547300
C	-20.96476500	-3.31242400	-6.45254600
H	-20.77572200	-4.36065500	-6.22551400
H	-20.11696200	-2.74924600	-6.83782000
P	-16.80378800	-4.67403500	-9.35169100
C	-17.64659000	-3.48396600	-10.49295700
C	-17.29861100	-3.56262800	-11.85575600
C	-18.63946500	-2.56530200	-10.11229300
C	-17.90406000	-2.73456700	-12.80186400
H	-16.54235600	-4.28472900	-12.17834500
C	-19.25687600	-1.74358900	-11.06315800
H	-18.94533200	-2.47590600	-9.06887100
C	-18.88900100	-1.82167500	-12.40781800
H	-17.61326400	-2.80837100	-13.85312600
H	-20.02620600	-1.03559300	-10.74420700
H	-19.37014400	-1.17744800	-13.14813600
C	-17.60779400	-4.29522700	-7.69522500
H	-17.55888100	-3.22086300	-7.45480500
H	-18.67220500	-4.55017300	-7.82346700
C	-15.13524000	-3.87679100	-9.13812400
C	-14.04348300	-4.71986300	-8.86464800
C	-14.90300700	-2.49488400	-9.25611900

C	-12.75595400	-4.19801600	-8.69926600
H	-14.20277100	-5.79965000	-8.79376700
C	-13.61535300	-1.97310200	-9.10256000
H	-15.73255800	-1.82084400	-9.48275000
C	-12.53848600	-2.82251500	-8.82292800
H	-11.91910800	-4.87064800	-8.49269600
H	-13.45074500	-0.89762600	-9.21282700
H	-11.52958600	-2.41434100	-8.71694600
C	-12.94023700	-2.20628000	-4.93769100
H	-12.34938500	-1.46951900	-5.49522200
H	-12.60594100	-2.23332600	-3.89027900
H	-12.80452300	-3.19722400	-5.39026900
C	-23.51233100	-4.99755200	-6.15306300
H	-24.21877000	-5.07626100	-5.30927300
H	-23.91131900	-5.65149300	-6.95053700
H	-22.54883500	-5.40520300	-5.82544300
C	-24.74954300	-2.96828100	-7.04426800
H	-25.22694800	-3.55894900	-7.84771900
H	-25.45972200	-2.97275000	-6.19823100
H	-24.63591100	-1.93242900	-7.39364100

UB3LYP-D3/def2-SVP-SMD(THF)//UB3LYP/def2-SVP(gas)
HF= -4404.1156206
UPBEPBE-D3/def2-SVP-SMD(THF)//UB3LYP/def2-SVP(gas)
HF= -4400.7597924

^{3}C'-outer-TS-dimethyl

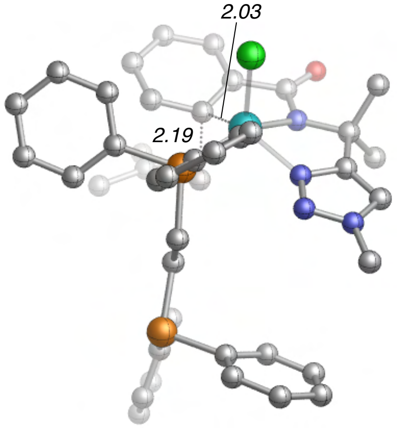

Zero-point correction= 0.805955 (Hartree/Particle)
Thermal correction to Energy= 0.858869
Thermal correction to Enthalpy= 0.859814
Thermal correction to Gibbs Free Energy= 0.711362
Sum of electronic and zero-point Energies= -4403.140319
Sum of electronic and thermal Energies= -4403.087404
Sum of electronic and thermal Enthalpies= -4403.086460
Sum of electronic and thermal Free Energies= -4403.234912

C	-20.40848000	-2.62003700	-4.73206500
C	-20.90967400	-1.39762400	-4.18421500
C	-22.21710000	-1.26775200	-3.71952600
C	-23.06454600	-2.37827000	-3.72331400
C	-22.57780500	-3.62003000	-4.17558600
C	-21.27542400	-3.74721600	-4.64553300
H	-22.53666900	-0.29604600	-3.33355100
H	-24.08942800	-2.29395600	-3.35224900
H	-23.22878100	-4.49917000	-4.15040300
H	-20.94418100	-4.72459900	-4.99922300
C	-19.95398400	-0.26094700	-4.12393800
O	-20.27002700	0.89970300	-3.86326700
C	-17.53368100	0.20420200	-4.29123300
C	-17.34917200	0.72962000	-2.84588000
H	-16.44651600	1.35788200	-2.76796700
H	-18.22764800	1.32708500	-2.56888000
H	-17.26223400	-0.11726700	-2.14975000
C	-17.65614500	1.37842000	-5.28871600
H	-18.54320500	1.97293600	-5.03874700
H	-16.76431100	2.02529000	-5.23985300
H	-17.75868000	1.00246200	-6.31856900
C	-16.34627700	-0.64687000	-4.66433400
N	-16.50749200	-1.99400400	-4.77519000
N	-15.38237500	-2.57590000	-5.09676500
C	-15.01476000	-0.39270000	-4.93278500
H	-14.43059200	0.52236700	-4.95871900
N	-14.47354200	-1.61751900	-5.18882300
N	-18.68389400	-0.71327200	-4.38433600

Fe	-18.43244800	-2.61006200	-4.25084100
P	-17.73885900	-5.00968000	-4.76044600
C	-16.99914000	-5.28159800	-6.45780700
H	-16.81763200	-6.36093500	-6.58564900
H	-16.01049900	-4.80341500	-6.38242100
C	-18.99593400	-6.34058000	-4.53882100
C	-19.60202900	-6.43449600	-3.27111500
C	-19.37280400	-7.24340200	-5.54488000
C	-20.55418000	-7.42224000	-3.01806900
H	-19.33313100	-5.71871200	-2.48899800
C	-20.33626900	-8.22691600	-5.28845300
H	-18.92016400	-7.19736300	-6.53728200
C	-20.92564200	-8.32018900	-4.02600600
H	-21.01513000	-7.48563200	-2.02917200
H	-20.62112500	-8.92342700	-6.08129200
H	-21.67545500	-9.09007200	-3.82647300
C	-16.34687700	-5.68796000	-3.72901300
C	-15.65546900	-4.89829600	-2.79883900
C	-15.95601000	-7.02804700	-3.90720500
C	-14.58498100	-5.43634600	-2.07585300
H	-15.98308500	-3.87778900	-2.60566900
C	-14.88398000	-7.56000500	-3.18795100
H	-16.49957300	-7.67347200	-4.60153700
C	-14.19214300	-6.76223600	-2.27079400
H	-14.06479500	-4.81221500	-1.34434300
H	-14.59552300	-8.60368500	-3.33814900
H	-13.35778300	-7.17930300	-1.70051300
Cl	-18.29192500	-2.96972600	-1.97310200
C	-22.81702400	-2.40813200	-7.74584500
C	-21.73446100	-1.73238400	-7.24718800
H	-21.87442800	-0.66117800	-7.05697400
C	-20.45375300	-2.25851500	-6.89319600
H	-20.19784700	-3.27182000	-7.20157100
H	-19.61515500	-1.56088300	-6.85598600
P	-16.97988100	-5.26394800	-9.27157900
C	-18.03623500	-4.44172200	-10.55246300
C	-17.84475500	-4.86414600	-11.88217400
C	-19.02362600	-3.47530900	-10.29576500
C	-18.59916400	-4.32208400	-12.92430800
H	-17.09610200	-5.63149000	-12.10126700
C	-19.79027300	-2.94211700	-11.33854800
H	-19.21226300	-3.12168500	-9.28111300
C	-19.57788200	-3.35928500	-12.65448100
H	-18.43026200	-4.66059900	-13.95001000
H	-20.55514100	-2.19472900	-11.11232500
H	-20.17632400	-2.94075800	-13.46780800
C	-17.76404700	-4.70631000	-7.65375500
H	-17.81421000	-3.60871900	-7.58195100
H	-18.80056200	-5.08012700	-7.68374300
C	-15.44867800	-4.20188800	-9.31910500
C	-14.20673800	-4.83930000	-9.15772900

C	-15.46803700	-2.81234000	-9.53522100
C	-13.01318800	-4.11030000	-9.20741600
H	-14.17546700	-5.92187300	-9.00553800
C	-14.27777000	-2.08258100	-9.59006500
H	-16.42070400	-2.29683300	-9.68120000
C	-13.04639300	-2.73045900	-9.42913700
H	-12.05596500	-4.62519800	-9.08902700
H	-14.31020600	-1.00515700	-9.77423600
H	-12.11448400	-2.16167900	-9.49234000
C	-13.11072700	-1.94639400	-5.57601800
H	-13.02758400	-2.00920700	-6.67116400
H	-12.43270000	-1.17328800	-5.19238900
H	-12.85041100	-2.91718700	-5.13612100
C	-24.10529000	-1.66118600	-8.01792900
H	-24.89656100	-2.33049400	-8.38877800
H	-24.48441000	-1.17143800	-7.10480400
H	-23.96638300	-0.86656800	-8.77205700
C	-22.82083000	-3.87593100	-8.07118900
H	-23.05988600	-4.03946000	-9.13873500
H	-21.86515400	-4.37268300	-7.85851400
H	-23.60499700	-4.40052200	-7.49668800

UB3LYP-D3/def2-SVP-SMD(THF)//UB3LYP/def2-SVP(gas)
HF= -4404.1549390
UPBEPBE-D3/def2-SVP-SMD(THF)//UB3LYP/def2-SVP(gas)
HF= -4400.7894737

^{5}C'-outer-TS-dimethyl

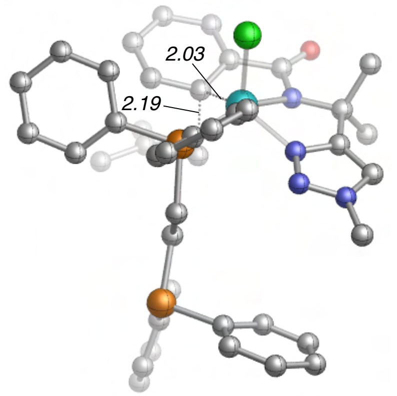

Zero-point correction= 0.805296 (Hartree/Particle)
Thermal correction to Energy= 0.859324
Thermal correction to Enthalpy= 0.860269
Thermal correction to Gibbs Free Energy= 0.708137
Sum of electronic and zero-point Energies= -4403.155497
Sum of electronic and thermal Energies= -4403.101469
Sum of electronic and thermal Enthalpies= -4403.100525
Sum of electronic and thermal Free Energies= -4403.252657

C	-20.74538600	-2.73590100	-4.23860600
C	-21.23888900	-1.46008200	-3.91204600
C	-22.52617100	-1.30777800	-3.38941400
C	-23.33061200	-2.43301400	-3.18348600
C	-22.83835400	-3.70866300	-3.49290300
C	-21.55403600	-3.86219400	-4.02822600
H	-22.86175100	-0.29950600	-3.13228400
H	-24.33715900	-2.32104600	-2.77143800
H	-23.46110000	-4.59198100	-3.32325700
H	-21.20089500	-4.86169900	-4.29011600
C	-20.30870000	-0.28519500	-4.03269000
O	-20.70390600	0.87697600	-3.88437200
C	-17.92726700	0.27440800	-4.23380700
C	-17.71891600	0.82263000	-2.79931200
H	-16.84809200	1.49737200	-2.74987300
H	-18.61724500	1.37930200	-2.49862900
H	-17.56823500	-0.01195700	-2.09855200
C	-18.11120800	1.44155300	-5.23045000
H	-19.01407400	2.00426600	-4.96568100
H	-17.24051000	2.11802800	-5.20091300
H	-18.21853700	1.05996300	-6.25604400
C	-16.70483500	-0.51914900	-4.64369500
N	-16.76957700	-1.87485400	-4.73953900
N	-15.61991600	-2.37439800	-5.10703300
C	-15.40723100	-0.16990000	-4.97339100
H	-14.89373900	0.78518000	-5.03206600
N	-14.78777200	-1.35290100	-5.24529900
N	-19.03206400	-0.69503600	-4.24888900
Fe	-18.65940700	-2.65934000	-4.06579500
P	-17.71095900	-5.11924500	-4.81348100

C	-16.93874200	-5.34835200	-6.50740700
H	-16.65869400	-6.40860200	-6.61981100
H	-15.99805600	-4.78028200	-6.43622800
C	-18.89093900	-6.52911400	-4.62650000
C	-19.49765100	-6.67926100	-3.36346400
C	-19.21710600	-7.43669500	-5.64716800
C	-20.39646600	-7.72122900	-3.13008900
H	-19.26558300	-5.96758300	-2.56545000
C	-20.12872900	-8.47322600	-5.41186100
H	-18.75952300	-7.35589600	-6.63510500
C	-20.71741400	-8.62000900	-4.15415600
H	-20.85361200	-7.82743700	-2.14295100
H	-20.37241900	-9.17073200	-6.21758100
H	-21.42563300	-9.43216800	-3.97091600
C	-16.28414000	-5.73370100	-3.78802800
C	-15.62085100	-4.88966000	-2.88499900
C	-15.83265200	-7.05797700	-3.93693600
C	-14.51934400	-5.35819100	-2.16016200
H	-15.99011000	-3.87936400	-2.71581000
C	-14.73113000	-7.52180100	-3.21481400
H	-16.35180000	-7.74360100	-4.61138800
C	-14.06845600	-6.66961100	-2.32526600
H	-14.02084800	-4.69223100	-1.45075000
H	-14.39560500	-8.55458400	-3.34129800
H	-13.21040300	-7.03308500	-1.75339000
Cl	-18.40371700	-3.21598600	-1.86368600
C	-20.91741900	-0.47108800	-7.55850600
C	-20.18920900	-1.61074100	-7.27409600
H	-19.12696500	-1.58830100	-7.54395700
C	-20.66690500	-2.78591900	-6.65856100
H	-21.73658900	-2.95182000	-6.54837400
H	-20.04947600	-3.68251000	-6.65685400
P	-16.93242400	-5.41017800	-9.32452300
C	-18.06337300	-4.71369000	-10.61516200
C	-17.75259300	-5.02128200	-11.95438700
C	-19.22535500	-3.96723800	-10.35578500
C	-18.56268700	-4.57867200	-13.00083500
H	-16.86355200	-5.61870900	-12.17828800
C	-20.04524200	-3.53450400	-11.40514600
H	-19.50932200	-3.70832900	-9.33476300
C	-19.71603300	-3.83404800	-12.72855500
H	-18.29899900	-4.82453100	-14.03291200
H	-20.94475800	-2.95581900	-11.17938000
H	-20.35705000	-3.49403200	-13.54598600
C	-17.74638300	-4.86754200	-7.71761100
H	-17.87354700	-3.77406300	-7.68820600
H	-18.75246800	-5.31719600	-7.73023300
C	-15.49526500	-4.22877400	-9.41356200
C	-14.21200000	-4.74016000	-9.15412700
C	-15.62205600	-2.86759100	-9.74385700
C	-13.08399700	-3.91450400	-9.21496600

H	-14.09487500	-5.80088200	-8.91472300
C	-14.49634000	-2.04250200	-9.81223300
H	-16.60811900	-2.45192700	-9.96648500
C	-13.22354200	-2.56427800	-9.54933400
H	-12.09315100	-4.33230100	-9.01737900
H	-14.61121800	-0.98952200	-10.08404000
H	-12.34146000	-1.92186300	-9.62068300
C	-13.42518000	-1.58390100	-5.69750900
H	-12.77995900	-0.77798100	-5.32462200
H	-13.08597700	-2.54569300	-5.29321900
H	-13.38459900	-1.61901500	-6.79650200
C	-22.34555100	-0.26918900	-7.14687600
H	-22.93336700	0.18789400	-7.96150200
H	-22.84557300	-1.19257200	-6.82747800
H	-22.37941100	0.43152800	-6.29204200
C	-20.28304100	0.68507700	-8.27511300
H	-20.82660500	0.91185400	-9.21080400
H	-20.33313900	1.60203300	-7.66062700
H	-19.22954100	0.49531400	-8.5278050

UB3LYP-D3/def2-SVP-SMD(THF)//UB3LYP/def2-SVP(gas)
HF= -4404.1682022
UPBEPBE-D3/def2-SVP-SMD(THF)//UB3LYP/def2-SVP(gas)
HF= -4400.7875190

[1]D-TS-dimethyl

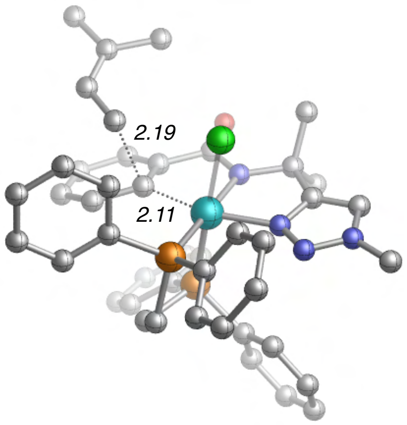

Zero-point correction= 0.810226 (Hartree/Particle)
Thermal correction to Energy= 0.861634
Thermal correction to Enthalpy= 0.862578
Thermal correction to Gibbs Free Energy= 0.725319
Sum of electronic and zero-point Energies= -4403.129327
Sum of electronic and thermal Energies= -4403.077919
Sum of electronic and thermal Enthalpies= -4403.076975
Sum of electronic and thermal Free Energies= -4403.214233

C	-18.81576900	1.25568300	-3.62758400
C	-19.29382200	2.58342900	-3.33380900
C	-20.55925900	3.02751000	-3.69084600
C	-21.42662800	2.18580200	-4.40602200
C	-21.01228400	0.90219700	-4.76396600
C	-19.74968600	0.45126500	-4.36050600
H	-20.84231800	4.04843300	-3.42269400
H	-22.42039400	2.54157000	-4.69407000
H	-21.67886300	0.24084800	-5.32470800
H	-19.47635700	-0.57873100	-4.60495300
C	-18.35183100	3.53957700	-2.65968100
O	-18.77356500	4.58510300	-2.12047900
C	-15.99695800	3.90364300	-2.17819800
C	-16.16082200	4.05754700	-0.64574900
H	-15.30459400	4.60615300	-0.21746600
H	-17.08201700	4.61774800	-0.44047300
H	-16.22259500	3.06434700	-0.18006800
C	-15.87142800	5.30023900	-2.83516500
H	-16.81627300	5.84335100	-2.70024100
H	-15.05593000	5.88012900	-2.37194500
H	-15.65049300	5.20888300	-3.91003200
C	-14.75109100	3.10430300	-2.46851700
N	-14.83605000	1.92678300	-3.15349400
N	-13.65844900	1.38427900	-3.27997500
C	-13.41800900	3.28342600	-2.15826800
H	-12.88847900	4.06239600	-1.61761300
N	-12.79058200	2.19266900	-2.67840500
N	-17.08801400	3.11633100	-2.77259600

Fe	-16.71388000	1.29696900	-3.51768700
P	-16.22704000	-0.86755400	-4.28665500
P	-16.74963200	1.90559200	-5.75725500
C	-16.12852200	-0.79273900	-6.15880300
H	-16.35002900	-1.75983200	-6.63434000
H	-15.07783800	-0.56798200	-6.39594500
C	-17.03281200	0.31397900	-6.70963500
H	-18.09359400	0.05075700	-6.57662000
H	-16.85583800	0.47092600	-7.78526900
C	-17.43305900	-2.24844000	-3.95098500
C	-17.61030300	-2.64663200	-2.61154700
C	-18.16636600	-2.91196900	-4.94819400
C	-18.48073200	-3.69053400	-2.28931300
H	-17.08075500	-2.11288600	-1.81804300
C	-19.04457900	-3.95239200	-4.62156000
H	-18.06581900	-2.62792300	-5.99745200
C	-19.20078400	-4.34922600	-3.29209500
H	-18.59999700	-3.98753900	-1.24389800
H	-19.60418500	-4.45545500	-5.41485400
H	-19.88099400	-5.16616300	-3.03746700
C	-14.65890100	-1.77716200	-3.85534200
C	-14.02581300	-2.63545900	-4.77124600
C	-14.11246700	-1.64291100	-2.56805100
C	-12.86897700	-3.33519600	-4.41484400
H	-14.43283700	-2.77887300	-5.77412100
C	-12.96317600	-2.35451800	-2.21021600
H	-14.59852900	-0.97904300	-1.84867800
C	-12.33401600	-3.19761200	-3.13104200
H	-12.39085200	-3.99683500	-5.14233700
H	-12.55711400	-2.24603900	-1.20049400
H	-11.43401200	-3.75077700	-2.84863200
C	-15.23284300	2.54822700	-6.64438000
C	-15.29219600	3.56290200	-7.61747700
C	-13.98117700	1.96133400	-6.37733600
C	-14.14280200	3.97274500	-8.30058600
H	-16.24296900	4.04180500	-7.85317000
C	-12.83522400	2.36418700	-7.06981600
H	-13.88395300	1.19768500	-5.60626200
C	-12.90980200	3.37305700	-8.03364600
H	-14.21712700	4.76515600	-9.05022100
H	-11.87714500	1.88625900	-6.84721400
H	-12.01296100	3.69158400	-8.57153500
C	-18.04708200	3.03959200	-6.43040800
C	-18.93593300	2.67329900	-7.45468200
C	-18.13168700	4.34247600	-5.90608900
C	-19.87782100	3.58449600	-7.94414100
H	-18.90292700	1.67400700	-7.89018300
C	-19.06699900	5.25385900	-6.39942100
H	-17.46303800	4.64776900	-5.10116000
C	-19.94505400	4.87754500	-7.42044200
H	-20.56002000	3.27872900	-8.74189200

H	-19.11554600	6.25850600	-5.97229500
H	-20.68076200	5.58907300	-7.80452200
Cl	-16.51279100	0.46222500	-1.20006900
C	-21.25998700	1.51942000	-0.63303700
C	-20.94461000	0.52357600	-1.51801700
H	-21.77028600	-0.04997000	-1.95094300
C	-19.61596200	0.16918900	-1.90746500
H	-18.74470100	0.53562000	-1.36093300
H	-19.46662400	-0.83004400	-2.31303400
C	-11.37659200	1.87153600	-2.68453300
H	-10.93719000	2.11105300	-1.70618700
H	-11.27681100	0.79615200	-2.87671800
H	-10.84763500	2.43539900	-3.46902100
C	-22.69122400	1.77034200	-0.25596100
H	-22.84018100	1.59686500	0.82487300
H	-22.95211500	2.82866600	-0.43309200
H	-23.39369400	1.13521200	-0.81423600
C	-20.24756100	2.42192500	0.00216600
H	-19.24294900	1.98108100	0.04853300
H	-20.14802700	3.35529100	-0.58603800
H	-20.56098700	2.70766000	1.01912800

UB3LYP-D3/def2-SVP-SMD(THF)//UB3LYP/def2-SVP(gas)
HF= -4404.1615751
UPBEPBE-D3/def2-SVP-SMD(THF)//UB3LYP/def2-SVP(gas)
HF= -4400.8141516

[3]D-TS-dimethyl

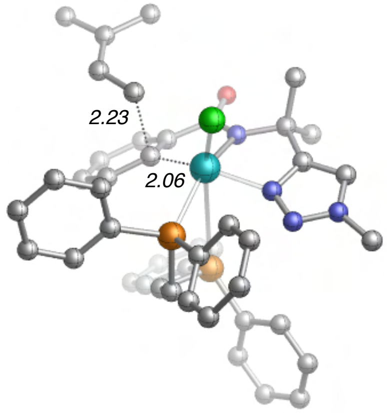

Zero-point correction= 0.807026 (Hartree/Particle)
Thermal correction to Energy= 0.860266
Thermal correction to Enthalpy= 0.861210
Thermal correction to Gibbs Free Energy= 0.713823
Sum of electronic and zero-point Energies= -4403.140153
Sum of electronic and thermal Energies= -4403.086912
Sum of electronic and thermal Enthalpies= -4403.085968
Sum of electronic and thermal Free Energies= -4403.233355

C	-18.87748700	1.24156100	-3.12739900
C	-19.30801700	2.59253700	-3.00719600
C	-20.45153800	3.07315600	-3.64011700
C	-21.19554200	2.22470400	-4.46827700
C	-20.77421000	0.89900000	-4.65127000
C	-19.63917000	0.41620000	-3.99701700
H	-20.73334100	4.11782000	-3.48414100
H	-22.09111600	2.59267500	-4.97570600
H	-21.34808700	0.22954200	-5.29965700
H	-19.38369500	-0.63791700	-4.11459200
C	-18.45672600	3.48626100	-2.16802700
O	-18.85494800	4.56263500	-1.71210400
C	-16.11237500	3.72104700	-1.42185800
C	-16.19863800	3.69252400	0.12068400
H	-15.35413700	4.23516900	0.57793500
H	-17.13437000	4.18030500	0.42975900
H	-16.19324700	2.65343600	0.47991100
C	-16.04717900	5.18254900	-1.93116700
H	-16.93845500	5.73330100	-1.61237900
H	-15.14599400	5.67874100	-1.53487300
H	-15.99958000	5.20168500	-3.03085200
C	-14.86302400	3.02189300	-1.90596400
N	-14.97293200	1.90025700	-2.67040300
N	-13.80136200	1.47790900	-3.05340500
C	-13.51217100	3.29189500	-1.80412600
H	-12.95339100	4.07373900	-1.29823300
N	-12.90760100	2.30950400	-2.52915100
N	-17.21601800	2.94405700	-2.01944300
Fe	-16.89516000	1.12692000	-2.60000600

P	-16.26099500	-0.82712200	-4.09120100
P	-16.54597900	2.38938500	-6.11985500
C	-16.06859500	-0.40917500	-5.90635300
H	-16.12076600	-1.34643100	-6.48042200
H	-15.02706700	-0.06346200	-6.00474500
C	-17.04025600	0.61848500	-6.51110900
H	-18.06347300	0.48024200	-6.12847900
H	-17.07318900	0.47540200	-7.60388300
C	-17.44808800	-2.24911500	-4.04131200
C	-17.70529100	-2.81483300	-2.77754200
C	-18.08733100	-2.78748800	-5.16878700
C	-18.57254800	-3.90238800	-2.65423500
H	-17.22787000	-2.39248600	-1.88786000
C	-18.96064900	-3.87353900	-5.03922200
H	-17.92071000	-2.36413100	-6.16122400
C	-19.20282000	-4.43564600	-3.78393200
H	-18.75959800	-4.33257300	-1.66697700
H	-19.45226100	-4.27986400	-5.92712600
H	-19.88331100	-5.28521600	-3.68477800
C	-14.67187200	-1.75631900	-3.79258000
C	-14.16289600	-2.62047800	-4.77993800
C	-13.98806200	-1.64970700	-2.57214900
C	-12.98853100	-3.34404500	-4.56088400
H	-14.68455000	-2.75136200	-5.72997100
C	-12.81725900	-2.38423700	-2.35254800
H	-14.38878900	-1.01125500	-1.78292500
C	-12.30959200	-3.22658500	-3.34416100
H	-12.60959600	-4.00904500	-5.34151900
H	-12.30402400	-2.29762200	-1.39086300
H	-11.39463800	-3.79881800	-3.16831300
C	-15.11187400	2.57778800	-7.29561000
C	-15.25144300	2.87415200	-8.66318100
C	-13.81510900	2.41390000	-6.77511600
C	-14.12846400	2.99406100	-9.48630500
H	-16.24703000	3.01988400	-9.08915900
C	-12.69149800	2.52208700	-7.60183100
H	-13.68558700	2.19912000	-5.71064000
C	-12.84499500	2.81583400	-8.95917400
H	-14.25653800	3.22973600	-10.54635600
H	-11.69142700	2.38418900	-7.18144200
H	-11.96777300	2.91072000	-9.60455500
C	-17.86317700	3.37355100	-6.96795700
C	-18.85873000	2.84259200	-7.80530000
C	-17.87845600	4.75676100	-6.70690800
C	-19.83201600	3.67219100	-8.37337300
H	-18.88917000	1.77333800	-8.02204200
C	-18.84170400	5.58694800	-7.28246600
H	-17.12916900	5.18635200	-6.03561600
C	-19.82418100	5.04547600	-8.11821200
H	-20.60072200	3.23941100	-9.01935500
H	-18.83380200	6.65845600	-7.06646800

H	-20.58590600	5.69165000	-8.56216000
Cl	-16.29544500	-0.07747600	-0.60280800
C	-21.41778400	1.54246500	-0.30362700
C	-20.80692400	0.54271700	-1.01184600
H	-21.45773300	-0.15610500	-1.54917400
C	-19.39858400	0.34102500	-1.14822800
H	-18.70733700	0.88578100	-0.50884100
H	-19.04831700	-0.64956200	-1.43437400
C	-11.49224800	2.11400600	-2.78863200
H	-11.36841600	1.11168300	-3.21589300
H	-11.11768700	2.86795700	-3.49779900
H	-10.92742600	2.18479400	-1.84847600
C	-22.91649800	1.64797300	-0.27113600
H	-23.40417300	0.87767000	-0.88622100
H	-23.29663700	1.56308000	0.76342300
H	-23.24203000	2.63934000	-0.63588500
C	-20.67514500	2.60185900	0.46090800
H	-19.65559100	2.29641400	0.73266200
H	-20.57894400	3.52232100	-0.14303500
H	-21.21519800	2.86912200	1.38456300

UB3LYP-D3/def2-SVP-SMD(THF)//UB3LYP/def2-SVP(gas)
HF= -4404.1605724
UPBEPBE-D3/def2-SVP-SMD(THF)//UB3LYP/def2-SVP(gas)
HF= -4400.7947406

^{5}D-TS-dimethyl

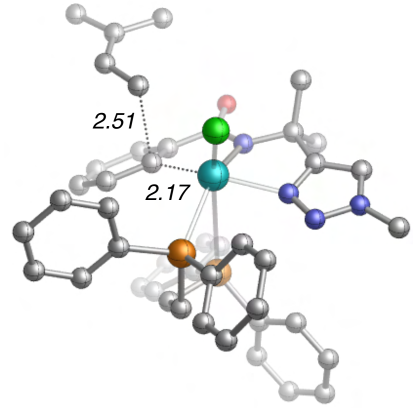

Zero-point correction= 0.805139 (Hartree/Particle)
Thermal correction to Energy= 0.859127
Thermal correction to Enthalpy= 0.860071
Thermal correction to Gibbs Free Energy= 0.708195
Sum of electronic and zero-point Energies= -4403.156624
Sum of electronic and thermal Energies= -4403.102636
Sum of electronic and thermal Enthalpies= -4403.101692
Sum of electronic and thermal Free Energies= -4403.253568

C	-18.91942400	1.08868500	-3.12297600
C	-19.27339800	2.45082600	-3.13564200
C	-20.41309900	2.89156300	-3.81558100
C	-21.20811600	1.97530000	-4.51014700
C	-20.86268600	0.61730300	-4.51320200
C	-19.73617900	0.17450200	-3.80874600
H	-20.65123800	3.95797500	-3.79189500
H	-22.09683900	2.31633000	-5.04808400
H	-21.48755300	-0.10652000	-5.04543100
H	-19.52398400	-0.89592700	-3.77369900
C	-18.39079200	3.43297800	-2.41578800
O	-18.81998000	4.53823200	-2.05339200
C	-16.08351500	3.78735300	-1.66870200
C	-16.28111700	3.92977700	-0.14138200
H	-15.47143200	4.52264200	0.31639700
H	-17.23714000	4.43818300	0.04712100
H	-16.29884800	2.93611400	0.33113100
C	-15.98620200	5.18495900	-2.33084300
H	-16.91456700	5.74174900	-2.16082700
H	-15.13717200	5.74991400	-1.91194400
H	-15.82891900	5.07913900	-3.41544500
C	-14.78604300	3.05916600	-1.95578100
N	-14.78689900	1.86229000	-2.60212200
N	-13.57058300	1.43226800	-2.78583200
C	-13.46049900	3.37663800	-1.72021000
H	-12.97969700	4.22307900	-1.23847800
N	-12.75715100	2.34014400	-2.25335200
N	-17.13633600	2.94900400	-2.25908000
Fe	-16.80422000	0.98655800	-2.62917000

P	-16.18725500	-0.98570900	-4.29808600
P	-16.66449800	2.21815500	-6.02219600
C	-16.06621200	-0.55340800	-6.11246800
H	-16.14558900	-1.47560600	-6.70832900
H	-15.03414800	-0.19549900	-6.25719600
C	-17.07032700	0.48813800	-6.62963500
H	-18.09286400	0.26309000	-6.28493300
H	-17.07647100	0.46262600	-7.73195200
C	-17.40484700	-2.38182500	-4.23024400
C	-17.65473000	-2.94815100	-2.96518000
C	-18.09408400	-2.88511300	-5.34453700
C	-18.55854600	-4.00363100	-2.82849700
H	-17.15040900	-2.54530100	-2.08200900
C	-19.00531300	-3.93800400	-5.20185200
H	-17.93724900	-2.46068300	-6.33793000
C	-19.23736700	-4.50262500	-3.94585500
H	-18.73789900	-4.43436300	-1.83995300
H	-19.53479700	-4.31625200	-6.08040500
H	-19.94784200	-5.32601600	-3.83620600
C	-14.59816100	-1.91784300	-4.04602600
C	-14.07139800	-2.74773700	-5.05317100
C	-13.92102900	-1.82773900	-2.82006300
C	-12.88827900	-3.45947000	-4.84431000
H	-14.58594500	-2.85731800	-6.01011600
C	-12.74146000	-2.55115800	-2.61077000
H	-14.32824300	-1.20553000	-2.02161500
C	-12.21871600	-3.36248700	-3.61998700
H	-12.49327800	-4.09860900	-5.63851400
H	-12.23167300	-2.47794100	-1.64626600
H	-11.29602300	-3.92501900	-3.45349900
C	-15.17703900	2.59060400	-7.07939400
C	-15.25118900	3.06214900	-8.40222300
C	-13.90621400	2.38594300	-6.51039400
C	-14.08915100	3.31068800	-9.13769000
H	-16.22606600	3.24299400	-8.86123500
C	-12.74363700	2.62250800	-7.25202200
H	-13.82502100	2.03977400	-5.47615100
C	-12.83177300	3.08817400	-8.56645900
H	-14.16627400	3.68078900	-10.16372900
H	-11.76469900	2.44948800	-6.79658200
H	-11.92395300	3.28245500	-9.14372200
C	-17.97405000	3.24789400	-6.82520900
C	-18.90326800	2.77987300	-7.76964600
C	-18.05808900	4.59199500	-6.41540200
C	-19.87811000	3.63383700	-8.29758700
H	-18.87951400	1.74129100	-8.10446200
C	-19.02295100	5.44749500	-6.94996000
H	-17.36552800	4.96988100	-5.65825700
C	-19.93827300	4.96974100	-7.89385400
H	-20.59371500	3.24972300	-9.02966300
H	-19.06910000	6.48747400	-6.61679800

H	-20.70067300	5.63539000	-8.30687600
Cl	-16.31808100	-0.29054500	-0.72678500
C	-21.45342200	1.85464600	-0.19414400
C	-20.86590900	0.69588400	-0.66666600
H	-21.54142300	-0.08708600	-1.02950900
C	-19.48713600	0.43994500	-0.76366900
H	-18.74562100	1.12212200	-0.35180600
H	-19.12534100	-0.56275800	-0.98081700
C	-11.31924600	2.14632800	-2.30081600
H	-11.13546600	1.15986800	-2.74303700
H	-10.84353500	2.92305000	-2.91823400
H	-10.89726100	2.17810500	-1.28575700
C	-22.94641700	2.00376600	-0.19846900
H	-23.33086900	2.15886800	0.82663000
H	-23.24284100	2.90065200	-0.77283600
H	-23.45496900	1.13128600	-0.63372800
C	-20.67419800	3.03009800	0.31384000
H	-19.69022000	2.74656200	0.71292600
H	-20.48176300	3.75589200	-0.50001100
H	-21.23072100	3.56089200	1.10349000

UB3LYP-D3/def2-SVP-SMD(THF)//UB3LYP/def2-SVP(gas)
HF= -4404.1724762
UPBEPBE-D3/def2-SVP-SMD(THF)//UB3LYP/def2-SVP(gas)
HF= -4400.7893847

[1]D'-dimethyl

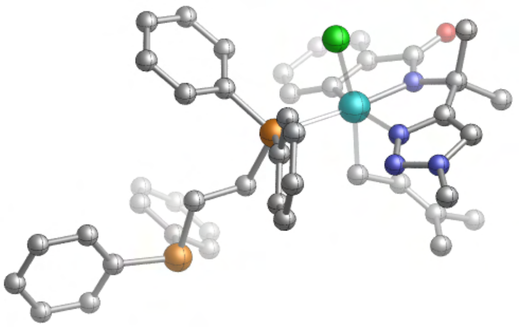

Zero-point correction= 0.809741 (Hartree/Particle)
Thermal correction to Energy= 0.862694
Thermal correction to Enthalpy= 0.863638
Thermal correction to Gibbs Free Energy= 0.718053
Sum of electronic and zero-point Energies= -4403.147781
Sum of electronic and thermal Energies= -4403.094828
Sum of electronic and thermal Enthalpies= -4403.093884
Sum of electronic and thermal Free Energies= -4403.239469

C	-20.05628800	-2.79714700	-3.41826300
C	-20.77326200	-1.80283400	-2.72914200
C	-22.12388300	-1.95395700	-2.39722900
C	-22.79718500	-3.11508700	-2.78034700
C	-22.11158500	-4.10512400	-3.49497700
C	-20.75575400	-3.94614100	-3.81373600
H	-22.62016000	-1.14943900	-1.84840600
H	-23.85393800	-3.24852600	-2.53397800
H	-22.63432200	-5.01220700	-3.81223400
H	-20.26732400	-4.74452800	-4.37703100
C	-20.01987200	-0.56337400	-2.38535900
O	-20.49862500	0.37090700	-1.74671600
C	-17.79967700	0.48993500	-2.63114000
C	-17.48976700	0.62345600	-1.11891500
H	-16.73730900	1.41216800	-0.95343400
H	-18.41022500	0.89362100	-0.58541300
H	-17.10997200	-0.32815600	-0.72341500
C	-18.32030200	1.83689000	-3.18677300
H	-19.24397400	2.11305400	-2.66470200
H	-17.56942200	2.62846000	-3.02836000
H	-18.52753100	1.75650600	-4.26268200
C	-16.54391000	0.10432100	-3.37105300
N	-16.45675200	-1.11751200	-3.96222800
N	-15.31658800	-1.26348900	-4.57286400
C	-15.35031800	0.74917800	-3.63326800
H	-14.96757200	1.72907800	-3.36361100
N	-14.63488400	-0.14193400	-4.37558800
N	-18.74292500	-0.61730300	-2.89270300
Fe	-18.16169800	-2.26869100	-3.59234600
Cl	-17.29507700	-2.96728500	-1.53902100
C	-18.81448500	0.28895600	-6.68168400
C	-19.29001800	-0.74708800	-5.95075000

H	-20.32215200	-0.66393200	-5.59073500
C	-18.61823100	-2.01398000	-5.61954100
H	-19.26204100	-2.86502300	-5.86148300
H	-17.63766400	-2.12792400	-6.09779700
C	-13.30757100	0.01101300	-4.94559900
H	-13.31984400	0.74833600	-5.76235000
H	-12.60067500	0.33639700	-4.16928000
H	-13.00135500	-0.96769700	-5.33361600
P	-17.20079800	-4.50459700	-4.26589000
C	-17.78174300	-5.17752100	-5.91732500
H	-17.47976900	-4.43314000	-6.67107000
H	-18.87989700	-5.11697800	-5.88115500
C	-17.36149900	-6.58746400	-6.34008700
H	-17.78054300	-7.33559600	-5.65052300
H	-16.26540800	-6.69580700	-6.31889600
P	-17.89338900	-6.94238900	-8.10920400
C	-19.73845500	-7.02368300	-7.88997200
C	-20.51030100	-5.98924600	-8.44542000
C	-20.39216600	-8.04543900	-7.17808100
C	-21.89993600	-5.96544700	-8.28227400
H	-20.01662700	-5.19553300	-9.01344000
C	-21.77975400	-8.02552600	-7.01911400
H	-19.81289700	-8.86719800	-6.74880900
C	-22.53656900	-6.98402400	-7.56902800
H	-22.48507300	-5.15109100	-8.71736900
H	-22.27423800	-8.82739300	-6.46424500
H	-23.62243200	-6.97029500	-7.44402600
C	-17.46060600	-8.73447800	-8.29733200
C	-17.78872500	-9.34155100	-9.52559800
C	-16.77237700	-9.50031800	-7.34116700
C	-17.46087900	-10.67336500	-9.78097800
H	-18.31066700	-8.76059200	-10.29206000
C	-16.43162200	-10.83318900	-7.60241100
H	-16.49636000	-9.06924100	-6.37723700
C	-16.77710700	-11.42521500	-8.81858300
H	-17.73299200	-11.12507100	-10.73864200
H	-15.89602000	-11.41113400	-6.84439300
H	-16.51254200	-12.46657700	-9.01912200
C	-17.42969900	-5.94249000	-3.11923500
C	-18.50376600	-5.97576400	-2.21545700
C	-16.54587000	-7.03681200	-3.15903600
C	-18.70465500	-7.08946600	-1.39476200
H	-19.17168600	-5.12039300	-2.13073800
C	-16.74499600	-8.14556600	-2.33196700
H	-15.68317900	-7.02127900	-3.82736200
C	-17.82972600	-8.17699700	-1.45085600
H	-19.54490800	-7.09504800	-0.69600600
H	-16.04475200	-8.98406000	-2.37356300
H	-17.98478300	-9.04249400	-0.80125800
C	-15.36376900	-4.41944000	-4.45249400
C	-14.73576700	-4.39350900	-5.70818400

C	-14.56811500	-4.30428500	-3.29692200
C	-13.34537200	-4.27670800	-5.80933300
H	-15.31986500	-4.46009300	-6.62737000
C	-13.18136000	-4.18475800	-3.40102000
H	-15.04463000	-4.28721100	-2.31492600
C	-12.56291200	-4.17501600	-4.65644800
H	-12.87549600	-4.27178400	-6.79654900
H	-12.57933400	-4.10152700	-2.49220300
H	-11.47520600	-4.09442000	-4.73459800
C	-19.68851200	1.47851500	-6.99060300
H	-19.23439900	2.41737000	-6.62247700
H	-19.81409900	1.60724500	-8.08155600
H	-20.68739700	1.38559700	-6.53976100
C	-17.42776500	0.36225200	-7.26824600
H	-16.80022100	-0.50330800	-7.01796000
H	-17.47230900	0.44400900	-8.36980900
H	-16.90691300	1.27172100	-6.91615200

UB3LYP-D3/def2-SVP-SMD(THF)//UB3LYP/def2-SVP(gas)
HF= -4404.1275348
UPBEPBE-D3/def2-SVP-SMD(THF)//UB3LYP/def2-SVP(gas)
HF= -4400.7906228

^{3}D'-dimethyl

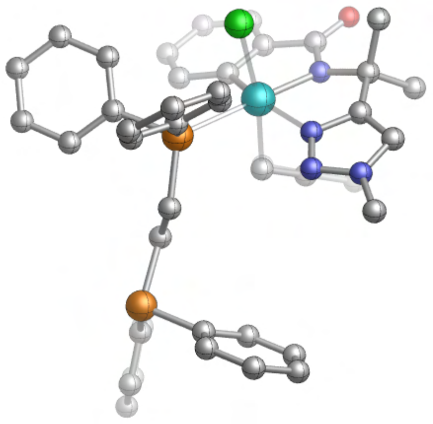

Zero-point correction= 0.808933 (Hartree/Particle)
Thermal correction to Energy= 0.862307
Thermal correction to Enthalpy= 0.863252
Thermal correction to Gibbs Free Energy= 0.714202
Sum of electronic and zero-point Energies= -4403.164845
Sum of electronic and thermal Energies= -4403.111471
Sum of electronic and thermal Enthalpies= -4403.110527
Sum of electronic and thermal Free Energies= -4403.259576

C	-20.25887700	-2.81654600	-4.70234100
C	-20.88034900	-1.64157200	-4.25247400
C	-22.27323000	-1.54223000	-4.16031600
C	-23.06488500	-2.62631200	-4.54419200
C	-22.45539100	-3.79607200	-5.01648300
C	-21.05871600	-3.89348500	-5.09743900
H	-22.70557800	-0.61072800	-3.78558800
H	-24.15458700	-2.56486800	-4.47906200
H	-23.07189800	-4.64610300	-5.32407500
H	-20.61772300	-4.82040300	-5.46936700
C	-19.98739000	-0.50796700	-3.86938000
O	-20.39618300	0.55048300	-3.39451500
C	-17.59323600	0.11224700	-3.72597000
C	-17.50856200	0.24975200	-2.18514800
H	-16.66396600	0.89840400	-1.89933500
H	-18.44133100	0.69818900	-1.81763400
H	-17.38415600	-0.73831300	-1.72164500
C	-17.75227500	1.51012400	-4.36940500
H	-18.68537700	1.96422500	-4.01670100
H	-16.90629300	2.15850900	-4.08673100
H	-17.78773900	1.43867000	-5.46597700
C	-16.32802300	-0.52684500	-4.24945100
N	-16.36184600	-1.80841700	-4.70426400
N	-15.19933000	-2.17945900	-5.16341100
C	-15.02964100	-0.08778600	-4.43481400
H	-14.53573100	0.85739300	-4.22975100
N	-14.38387900	-1.14578600	-5.00285700
N	-18.67547900	-0.81508800	-4.12071700
Fe	-18.29112300	-2.62391700	-4.57699000

P	-17.42653600	-5.07186400	-5.00625600
C	-16.64085600	-5.44304200	-6.67484900
H	-16.16652600	-6.43429800	-6.59341100
H	-15.80950600	-4.72088400	-6.73171200
C	-18.60538600	-6.47097200	-4.71503200
C	-19.38646100	-6.44130800	-3.54386800
C	-18.72777200	-7.57144900	-5.58164600
C	-20.26523400	-7.48690300	-3.25443900
H	-19.30000000	-5.59296200	-2.86066600
C	-19.61611000	-8.61292600	-5.29038800
H	-18.12621300	-7.64127000	-6.48865200
C	-20.38724400	-8.57329300	-4.12695900
H	-20.86257700	-7.44719900	-2.34005100
H	-19.69836200	-9.45914600	-5.97752700
H	-21.08027200	-9.38748600	-3.89952500
C	-15.98923200	-5.60713200	-3.93885800
C	-15.32082000	-4.72457800	-3.07785700
C	-15.54049100	-6.93875300	-4.01532500
C	-14.21935700	-5.15822000	-2.33234000
H	-15.68902700	-3.70961500	-2.95326300
C	-14.43992000	-7.36998600	-3.27172700
H	-16.06108600	-7.66132100	-4.64779900
C	-13.77116700	-6.47701600	-2.42929400
H	-13.71897000	-4.45783400	-1.65818300
H	-14.11188700	-8.41016100	-3.34518900
H	-12.91333900	-6.81335500	-1.84085500
Cl	-18.19992500	-3.31923100	-2.33115100
C	-19.89332200	-0.51395000	-7.72063400
C	-18.74756500	-0.97921400	-7.15850500
H	-17.93769000	-0.25185400	-7.03218200
C	-18.44532800	-2.33295100	-6.70782100
H	-19.14556100	-3.09840500	-7.04802700
H	-17.40325400	-2.61216000	-6.90150200
P	-16.56119800	-6.09574400	-9.40820900
C	-17.68535800	-5.71318500	-10.83083700
C	-17.23036100	-6.07339100	-12.11454100
C	-18.97350600	-5.16488400	-10.71169000
C	-18.02583400	-5.87147400	-13.24274500
H	-16.23710900	-6.51775200	-12.23030300
C	-19.77685200	-4.97356400	-11.84255100
H	-19.36838200	-4.87700300	-9.73586900
C	-19.30606000	-5.32139700	-13.10996400
H	-17.64878200	-6.15190700	-14.22985600
H	-20.77640200	-4.54600700	-11.72707300
H	-19.93400800	-5.16915900	-13.99149500
C	-17.49583300	-5.36979700	-7.94710300
H	-17.79646900	-4.33810400	-8.16554800
H	-18.41827000	-5.96264700	-7.84478200
C	-15.23983300	-4.80159600	-9.62926100
C	-13.91100300	-5.16383700	-9.35399500
C	-15.50782300	-3.48304700	-10.03972700

C	-12.87378000	-4.23155300	-9.47563800
H	-13.68655400	-6.18905800	-9.04618900
C	-14.47394600	-2.55237000	-10.16502800
H	-16.53277800	-3.18215700	-10.27178300
C	-13.15382200	-2.92448200	-9.88283300
H	-11.84443300	-4.53252400	-9.26257600
H	-14.69839000	-1.53293100	-10.49049300
H	-12.34413800	-2.19766300	-9.99179800
C	-12.98589900	-1.25792500	-5.38464200
H	-12.35959300	-1.46500300	-4.50376400
H	-12.89797300	-2.08488700	-6.09947500
H	-12.65630800	-0.32313000	-5.85796600
C	-20.01242800	0.92876000	-8.13678000
H	-20.26479800	1.01578700	-9.20938100
H	-20.82950400	1.42827300	-7.58511600
H	-19.08609000	1.49363600	-7.95476400
C	-21.11911000	-1.34735700	-7.97490100
H	-21.41599400	-1.28566800	-9.03771800
H	-20.99288300	-2.40391300	-7.70832400
H	-21.97233300	-0.96481300	-7.38795500

UB3LYP-D3/def2-SVP-SMD(THF)//UB3LYP/def2-SVP(gas)
HF= -4404.1773179
UPBEPBE-D3/def2-SVP-SMD(THF)//UB3LYP/def2-SVP(gas)
HF= -4400.8219974

^{5}D'-dimethyl

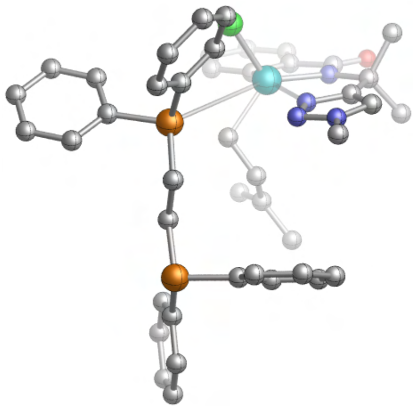

Zero-point correction= 0.805466 (Hartree/Particle)
Thermal correction to Energy= 0.860428
Thermal correction to Enthalpy= 0.861372
Thermal correction to Gibbs Free Energy= 0.700898
Sum of electronic and zero-point Energies= -4403.176318
Sum of electronic and thermal Energies= -4403.121356
Sum of electronic and thermal Enthalpies= -4403.120412
Sum of electronic and thermal Free Energies= -4403.280886

C	-20.94361700	-1.36729200	-3.92979300
C	-21.06173800	-0.00573900	-3.61568700
C	-22.31226400	0.53291100	-3.28820900
C	-23.43798000	-0.29265200	-3.25899900
C	-23.31172100	-1.65813200	-3.54466900
C	-22.06298500	-2.19978200	-3.87977600
H	-22.36573100	1.59822200	-3.04902800
H	-24.41623700	0.12259500	-3.00271000
H	-24.18975900	-2.30929300	-3.50504500
H	-21.97946400	-3.27067500	-4.08643300
C	-19.82244100	0.85142600	-3.59533200
O	-19.86793800	2.05767200	-3.35701800
C	-17.38202300	0.74982800	-3.89765700
C	-16.95554500	1.25263300	-2.49886900
H	-15.94103600	1.68393700	-2.52214000
H	-17.66154700	2.02545100	-2.16669500
H	-16.97015300	0.42310800	-1.77587500
C	-17.32789800	1.90405300	-4.92905300
H	-18.01757700	2.69999900	-4.62335600
H	-16.30729200	2.31456900	-4.99945900
H	-17.62717500	1.53904500	-5.92361000
C	-16.44106500	-0.34342300	-4.35788800
N	-16.92590100	-1.57868700	-4.65750000
N	-15.97324400	-2.38097600	-5.04765600
C	-15.07738500	-0.40295300	-4.58465400
H	-14.28130100	0.32831100	-4.48075600
N	-14.85173200	-1.67910800	-5.00555300
N	-18.70396600	0.11258100	-3.84967500
Fe	-18.98599700	-1.86284400	-4.09120800
P	-17.08841100	-5.98855200	-5.35584600

C	-16.18298400	-6.23842200	-6.97759000
H	-15.74067900	-7.24504300	-7.06407800
H	-15.33715200	-5.53263700	-6.92615900
C	-18.14135800	-7.50756100	-5.19029500
C	-19.12799400	-7.46055400	-4.18632100
C	-18.05271100	-8.65907600	-5.98981300
C	-19.98249300	-8.54423500	-3.97270800
H	-19.22379100	-6.56018500	-3.57085600
C	-18.91891900	-9.73910100	-5.78495300
H	-17.30392400	-8.72805800	-6.78266100
C	-19.88188700	-9.68693700	-4.77388600
H	-20.73673100	-8.49250500	-3.18293900
H	-18.83717600	-10.62645800	-6.41869500
H	-20.55631100	-10.53219700	-4.61364300
C	-15.72149700	-6.35349100	-4.14620700
C	-15.49894100	-5.42622500	-3.11372800
C	-14.90865600	-7.49892200	-4.21447400
C	-14.47751300	-5.63614100	-2.17887200
H	-16.14613300	-4.54947200	-3.03066600
C	-13.88676300	-7.70476500	-3.28471100
H	-15.08024300	-8.24800500	-4.99210500
C	-13.66717200	-6.77098100	-2.26524000
H	-14.32408400	-4.91244800	-1.37357300
H	-13.26424800	-8.60139700	-3.35006500
H	-12.87150600	-6.93582400	-1.53360400
Cl	-18.67017600	-3.34351200	-2.43068400
C	-20.02368600	-1.31033500	-7.82925300
C	-19.16771400	-1.97954300	-7.00889300
H	-18.09781600	-1.86651200	-7.21683600
C	-19.49786200	-2.82895800	-5.87357100
H	-20.51943800	-3.21134400	-5.81898400
H	-18.75358300	-3.61325700	-5.64942900
P	-16.11042700	-6.07353900	-9.80485800
C	-17.35439000	-5.51379500	-11.06011400
C	-16.93898600	-5.50413500	-12.40617700
C	-18.68680800	-5.17140200	-10.77333200
C	-17.81730800	-5.14268700	-13.42837300
H	-15.91135000	-5.78594400	-12.65519900
C	-19.57231600	-4.82218000	-11.80039000
H	-19.05166200	-5.17046600	-9.74479200
C	-19.14127900	-4.80213600	-13.12832700
H	-17.47063500	-5.13634500	-14.46520400
H	-20.60587200	-4.56278700	-11.55562600
H	-19.83394000	-4.52806900	-13.92820700
C	-17.06006700	-5.91717600	-8.19386700
H	-17.49539600	-4.90904200	-8.09304000
H	-17.89996000	-6.62933100	-8.25800900
C	-15.00901900	-4.57420900	-9.69090900
C	-13.63608600	-4.78440100	-9.47792400
C	-15.47657800	-3.25221900	-9.80215700
C	-12.75167400	-3.70480600	-9.36940300

H	-13.25508100	-5.80715500	-9.40658800
C	-14.59545500	-2.17270200	-9.69939000
H	-16.53837000	-3.06479700	-9.98218000
C	-13.22973300	-2.39607600	-9.48274400
H	-11.68545600	-3.88883000	-9.21157600
H	-14.97376600	-1.15154200	-9.79934400
H	-12.53891400	-1.55081900	-9.41838100
C	-13.59878300	-2.29523900	-5.41811600
H	-13.51712600	-2.30149600	-6.51538800
H	-12.76444700	-1.72831900	-4.98616300
H	-13.57711300	-3.32651700	-5.04334300
C	-21.51805500	-1.31545500	-7.67509500
H	-21.87952000	-0.29900900	-7.43661300
H	-22.00669600	-1.60372900	-8.62293700
H	-21.87378900	-1.98246400	-6.88051500
C	-19.49978800	-0.48624000	-8.97409900
H	-19.89497100	-0.85185800	-9.93929500
H	-19.82951900	0.56505400	-8.88556900
H	-18.40103600	-0.49824100	-9.02665400

UB3LYP-D3/def2-SVP-SMD(THF)//UB3LYP/def2-SVP(gas)
HF= -4404.1664393
UPBEPBE-D3/def2-SVP-SMD(THF)//UB3LYP/def2-SVP(gas)
HF= -4400.7992476

^{1}D''-dimethyl

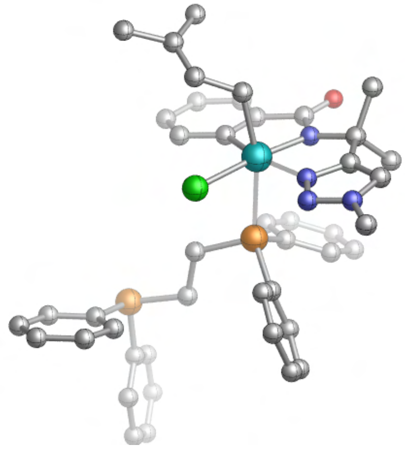

Zero-point correction= 0.808798 (Hartree/Particle)
Thermal correction to Energy= 0.862228
Thermal correction to Enthalpy= 0.863172
Thermal correction to Gibbs Free Energy= 0.714954
Sum of electronic and zero-point Energies= -4403.158106
Sum of electronic and thermal Energies= -4403.104676
Sum of electronic and thermal Enthalpies= -4403.103732
Sum of electronic and thermal Free Energies= -4403.251951

C	-18.32080800	0.89794300	-3.50742700
C	-19.17872300	1.95054600	-3.14261900
C	-20.57052000	1.82727300	-3.23501500
C	-21.11756300	0.63159400	-3.70497600
C	-20.27177400	-0.43068100	-4.06257600
C	-18.87974600	-0.30561400	-3.95877100
H	-21.19516000	2.67163500	-2.93215100
H	-22.20192200	0.51718900	-3.78791000
H	-20.70395200	-1.36945500	-4.42229500
H	-18.22923700	-1.14335100	-4.22048600
C	-18.52184200	3.18469600	-2.61688700
O	-19.13848500	4.15518900	-2.18165200
C	-16.28970800	4.13062600	-2.12544900
C	-16.48705000	4.28631900	-0.59667800
H	-15.82180600	5.06836400	-0.19575800
H	-17.52829000	4.57086100	-0.39628400
H	-16.26554800	3.34416300	-0.07376200
C	-16.52836400	5.48911400	-2.82388800
H	-17.55895600	5.81710800	-2.64371500
H	-15.83164000	6.24861000	-2.43248700
H	-16.37401200	5.39618600	-3.90714200
C	-14.87785900	3.67287800	-2.40383200
N	-14.66736900	2.45201700	-2.97093200
N	-13.39386200	2.21470600	-3.11577400
C	-13.62867500	4.22476600	-2.19339500
H	-13.30032000	5.17050100	-1.77210800
N	-12.75751000	3.28054300	-2.65031100
N	-17.15020200	3.05962200	-2.67031000
Fe	-16.45008000	1.40548500	-3.27639800

P	-16.60243900	2.03886300	-5.94184500
C	-17.32611200	0.72250600	-8.43005300
H	-17.98306600	1.56692700	-8.69526300
H	-16.34645500	0.93796900	-8.88435100
C	-17.22641000	0.56972300	-6.91157300
H	-16.54056500	-0.24034400	-6.61863800
H	-18.19877800	0.29852100	-6.47272000
C	-15.02506900	2.43480900	-6.83822900
C	-14.95190000	3.26489800	-7.97265400
C	-13.85000100	1.80410900	-6.38779500
C	-13.73381400	3.47431600	-8.62617900
H	-15.84972500	3.75015200	-8.35866900
C	-12.63557000	2.01017800	-7.05053100
H	-13.89131700	1.13400800	-5.52664400
C	-12.57069600	2.84993400	-8.16580300
H	-13.69823100	4.12236600	-9.50600200
H	-11.73520200	1.50576200	-6.68920200
H	-11.61979200	3.01193300	-8.68059400
C	-17.76719200	3.40231000	-6.36880400
C	-19.13879100	3.13056900	-6.52992700
C	-17.34972700	4.74645500	-6.40075200
C	-20.05478600	4.16510800	-6.73995400
H	-19.50930400	2.10519600	-6.48009900
C	-18.26579800	5.77909500	-6.61460700
H	-16.29505600	4.99409300	-6.26368900
C	-19.62292700	5.49246200	-6.78773600
H	-21.11463200	3.92727000	-6.86039800
H	-17.91594400	6.81449000	-6.64061400
H	-20.34027200	6.30050100	-6.95131200
Cl	-15.42596900	-0.48642400	-4.09276700
C	-11.30655400	3.34012800	-2.70436000
H	-10.97921200	4.06033400	-3.46898400
H	-10.90513000	3.63540200	-1.72477900
H	-10.94462000	2.33878200	-2.96550600
P	-18.12975100	-0.76207500	-9.26056900
C	-18.08841900	-0.16178700	-11.02102800
C	-19.21814000	0.53845200	-11.48200400
C	-17.01609500	-0.36107700	-11.90707800
C	-19.26722300	1.04601200	-12.78316300
H	-20.07260600	0.68155900	-10.81328800
C	-17.06970100	0.13584900	-13.21308300
H	-16.13186100	-0.91127700	-11.57719700
C	-18.19209200	0.84354700	-13.65392400
H	-20.15269900	1.59099100	-13.12140100
H	-16.22738900	-0.03092700	-13.89022000
H	-18.23159900	1.23068500	-14.67552800
C	-16.79189000	-2.04418800	-9.24997000
C	-17.17722600	-3.35775700	-9.57851400
C	-15.44909800	-1.81097800	-8.90719700
C	-16.24818700	-4.40018000	-9.58707000
H	-18.22254800	-3.56410100	-9.82766200

C	-14.52036800	-2.85763700	-8.89942500
H	-15.11025400	-0.80810700	-8.64014400
C	-14.91491100	-4.15283100	-9.24354000
H	-16.56847000	-5.41160800	-9.85114800
H	-13.48278100	-2.65577700	-8.62063600
H	-14.18805500	-4.96926000	-9.23648800
C	-18.03998200	-1.44795500	-0.73865600
C	-16.91561400	-0.89941700	-1.26483300
H	-16.15164800	-1.58421700	-1.64303300
C	-16.60651700	0.52044600	-1.39856100
H	-15.54480600	0.73961500	-1.20683500
H	-17.26216700	1.19247400	-0.83968900
C	-18.19157100	-2.94443200	-0.66041900
H	-18.34566700	-3.27597500	0.38282800
H	-19.08334500	-3.27670700	-1.22299400
H	-17.31549200	-3.47136400	-1.06566900
C	-19.21130000	-0.66687700	-0.20894000
H	-19.05087600	0.41858100	-0.20504900
H	-20.10867100	-0.86374100	-0.82203400
H	-19.45801100	-0.98446700	0.82013600

UB3LYP-D3/def2-SVP-SMD(THF)//UB3LYP/def2-SVP(gas)
HF= -4404.1348902
UPBEPBE-D3/def2-SVP-SMD(THF)//UB3LYP/def2-SVP(gas)
HF= -4400.7947239

^{3}D''-dimethyl

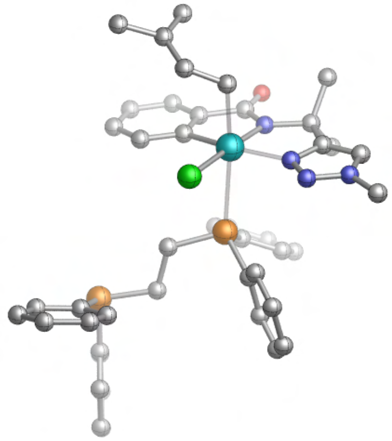

Zero-point correction= 0.807091 (Hartree/Particle)
Thermal correction to Energy= 0.861276
Thermal correction to Enthalpy= 0.862221
Thermal correction to Gibbs Free Energy= 0.710246
Sum of electronic and zero-point Energies= -4403.184691
Sum of electronic and thermal Energies= -4403.130505
Sum of electronic and thermal Enthalpies= -4403.129561
Sum of electronic and thermal Free Energies= -4403.281536

C	-18.27736600	0.62544300	-3.51655100
C	-19.21812100	1.56695300	-3.06829800
C	-20.59379500	1.32424900	-3.15942700
C	-21.03864500	0.11676700	-3.70317500
C	-20.10712300	-0.83605400	-4.14154800
C	-18.72979200	-0.58793500	-4.05022200
H	-21.28646900	2.08934100	-2.79853900
H	-22.10954800	-0.08910200	-3.78461900
H	-20.45704300	-1.78366800	-4.56226300
H	-18.01123700	-1.34042800	-4.38205800
C	-18.66998200	2.82913800	-2.48068900
O	-19.37433300	3.71963500	-2.00813100
C	-16.54089100	3.99725600	-2.03835800
C	-16.73269000	4.19393000	-0.51424400
H	-16.14893500	5.05723800	-0.15481300
H	-17.79564500	4.37408000	-0.30826500
H	-16.40462300	3.30378000	0.04345800
C	-16.92448800	5.29638700	-2.78549600
H	-17.97729300	5.53240700	-2.58833400
H	-16.29643400	6.13765700	-2.44855300
H	-16.79247800	5.16760400	-3.86840600
C	-15.09269000	3.67528200	-2.32477300
N	-14.76775800	2.48755600	-2.90771700
N	-13.47894800	2.37749400	-3.06775700
C	-13.90074900	4.34455600	-2.12049100
H	-13.66301200	5.31419800	-1.69279800
N	-12.94515500	3.49606100	-2.59548700
N	-17.29770000	2.83324600	-2.54204800

Fe	-16.42830600	1.25750500	-3.24038000
P	-16.48890200	2.07237800	-6.02858500
C	-17.28499800	0.77839400	-8.52024700
H	-17.90706500	1.64971200	-8.78326200
H	-16.30110400	0.94903800	-8.98455600
C	-17.17768600	0.63040500	-7.00186600
H	-16.52253300	-0.20683600	-6.71319900
H	-18.15676400	0.39699600	-6.55509900
C	-14.89444100	2.40101600	-6.92160500
C	-14.78849200	3.16789500	-8.09748400
C	-13.73536500	1.78719000	-6.41094600
C	-13.55533900	3.32854900	-8.73605800
H	-15.67327300	3.64352500	-8.52452600
C	-12.50517300	1.94266200	-7.05888900
H	-13.80156700	1.16945200	-5.51281700
C	-12.40908900	2.71741900	-8.21793600
H	-13.49346000	3.92773200	-9.64853600
H	-11.61735700	1.45142300	-6.65124900
H	-11.44619400	2.84069600	-8.72112300
C	-17.58900700	3.48665000	-6.47415900
C	-18.97610900	3.28235900	-6.60198500
C	-17.10584600	4.80778700	-6.53854000
C	-19.84399300	4.35751600	-6.81155700
H	-19.39389000	2.27660800	-6.52574800
C	-17.97432900	5.88145000	-6.75280200
H	-16.03766600	5.00468200	-6.42305600
C	-19.34743200	5.66094000	-6.89309600
H	-20.91689600	4.17176100	-6.90615400
H	-17.57388200	6.89745000	-6.80603500
H	-20.02691300	6.50087300	-7.05785900
Cl	-15.33006400	-0.56364800	-4.10666300
C	-11.50723200	3.69625100	-2.65736500
H	-11.25962500	4.48195300	-3.38649400
H	-11.12164500	3.97807100	-1.66731900
H	-11.05617200	2.74850600	-2.97374600
P	-18.15985200	-0.67508300	-9.33298300
C	-18.12026600	-0.08739700	-11.09764400
C	-19.23343100	0.64379500	-11.55065900
C	-17.06403800	-0.32370100	-11.99388500
C	-19.28154100	1.14507100	-12.85432300
H	-20.07568700	0.81634800	-10.87348300
C	-17.11694800	0.16703200	-13.30221900
H	-16.19330900	-0.89838900	-11.66978000
C	-18.22251000	0.90549200	-13.73526700
H	-20.15396500	1.71440300	-13.18635400
H	-16.28752700	-0.02885900	-13.98743300
H	-18.26157900	1.28772200	-14.75874400
C	-16.87436000	-2.01033100	-9.33426400
C	-17.31555400	-3.30810700	-9.65552600
C	-15.51998100	-1.83086600	-9.00497300
C	-16.42957300	-4.38725700	-9.67044500

H	-18.37078000	-3.47259800	-9.89385700
C	-14.63419400	-2.91420500	-9.00371200
H	-15.13844300	-0.84195000	-8.74312800
C	-15.08398700	-4.19314200	-9.34076500
H	-16.79305300	-5.38551100	-9.92875100
H	-13.58661100	-2.75380200	-8.73557000
H	-14.39057400	-5.03820900	-9.33890800
C	-17.96106400	-1.33375300	-0.47139700
C	-16.78194300	-0.99479400	-1.08230400
H	-16.21737000	-1.80006900	-1.56257500
C	-16.21383200	0.31307800	-1.20289500
H	-15.12769700	0.35121000	-1.32775900
H	-16.63851300	1.11964000	-0.60283500
C	-18.42121300	-2.76416700	-0.44893200
H	-18.59066600	-3.11317700	0.58595900
H	-19.39012400	-2.86501900	-0.97148800
H	-17.70063900	-3.44016000	-0.93129300
C	-18.88700100	-0.34999200	0.18413000
H	-19.11023300	-0.65691200	1.22168000
H	-18.49769700	0.67558600	0.20381500

UB3LYP-D3/def2-SVP-SMD(THF)//UB3LYP/def2-SVP(gas)
HF= -4404.1874473
UPBEPBE-D3/def2-SVP-SMD(THF)//UB3LYP/def2-SVP(gas)
HF= -4400.8275687

[5]D''-dimethyl

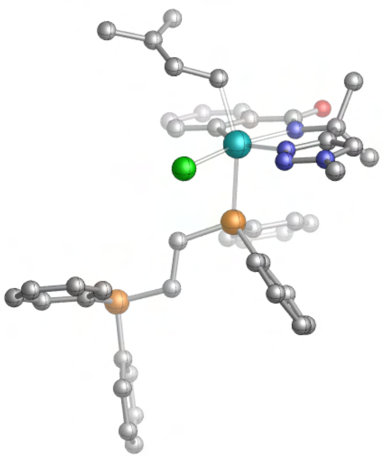

Zero-point correction= 0.806900 (Hartree/Particle)
Thermal correction to Energy= 0.861072
Thermal correction to Enthalpy= 0.862016
Thermal correction to Gibbs Free Energy= 0.709260
Sum of electronic and zero-point Energies= -4403.172673
Sum of electronic and thermal Energies= -4403.118501
Sum of electronic and thermal Enthalpies= -4403.117557
Sum of electronic and thermal Free Energies= -4403.270314

C	-18.49393500	1.18574800	-3.30866000
C	-19.07725400	2.40183800	-2.92874700
C	-20.47209000	2.54127600	-2.94437400
C	-21.27353100	1.47543300	-3.35948100
C	-20.68324400	0.26556600	-3.75000200
C	-19.28934000	0.11253400	-3.71220700
H	-20.89678400	3.49786100	-2.63004700
H	-22.36138700	1.58301200	-3.37966700
H	-21.31116300	-0.56856500	-4.07776100
H	-18.83353700	-0.84346300	-3.98667300
C	-18.19142500	3.54310400	-2.49846900
O	-18.67100300	4.59507300	-2.06318000
C	-15.86590000	4.21572700	-2.17491000
C	-15.99501700	4.45659700	-0.64895700
H	-15.20008500	5.12916600	-0.28702700
H	-16.97204300	4.90871600	-0.43605500
H	-15.91691500	3.50348000	-0.10358400
C	-15.92432000	5.56926700	-2.92428700
H	-16.92062400	6.01025400	-2.79481000
H	-15.16931500	6.26842000	-2.52816100
H	-15.72141000	5.42753300	-3.99672300
C	-14.52926200	3.56480400	-2.45097600
N	-14.47270500	2.30997800	-2.97132500
N	-13.23663000	1.92288600	-3.12827300
C	-13.22036300	3.97663400	-2.27897500
H	-12.77825800	4.89384700	-1.90073100
N	-12.47151900	2.92393000	-2.71230000
N	-16.86726000	3.25388200	-2.65202100

Fe	-16.44876900	1.34828700	-3.39898500
P	-16.94667400	1.99523100	-5.96854700
C	-17.20699600	0.59606900	-8.49687900
H	-17.75850400	1.47504800	-8.86803600
H	-16.15983800	0.74385800	-8.80429600
C	-17.32336100	0.47176700	-6.97436700
H	-16.64019200	-0.29090400	-6.57197800
H	-18.33245300	0.15288500	-6.66517700
C	-15.47674800	2.72881100	-6.82488500
C	-15.55189600	3.82902000	-7.69605800
C	-14.22217400	2.13271700	-6.58919500
C	-14.40016600	4.32275100	-8.31753500
H	-16.51275100	4.30616600	-7.89632600
C	-13.07718200	2.62396800	-7.22216800
H	-14.14382100	1.28039500	-5.90890100
C	-13.16054200	3.72127100	-8.08541900
H	-14.47730800	5.17899700	-8.99290400
H	-12.11257400	2.14433200	-7.03486000
H	-12.26279400	4.10568600	-8.57688900
C	-18.33269500	3.15561300	-6.31618700
C	-19.50736100	2.76389700	-6.98222200
C	-18.25960500	4.46462200	-5.80470900
C	-20.56611500	3.66228100	-7.14787000
H	-19.61617800	1.75212000	-7.37433500
C	-19.31572000	5.36106700	-5.97316200
H	-17.37354000	4.78353900	-5.25543800
C	-20.47371100	4.96296700	-6.64792700
H	-21.47022700	3.33675900	-7.66878300
H	-19.23849400	6.36891200	-5.55838800
H	-21.30456700	5.66167500	-6.77468500
Cl	-15.33143800	-0.51388500	-4.29695700
C	-11.02507500	2.80785000	-2.77791600
H	-10.61421100	3.52866900	-3.50034100
H	-10.58307700	2.98952400	-1.78766200
H	-10.79260500	1.78727200	-3.10413800
P	-17.98681500	-0.84630200	-9.41622800
C	-17.64419500	-0.29999400	-11.16110900
C	-18.69468200	0.32206000	-11.85844800
C	-16.40850700	-0.46048000	-11.81140600
C	-18.51224800	0.78920700	-13.16356800
H	-19.66906200	0.43506300	-11.37360600
C	-16.22785200	-0.00206900	-13.11931500
H	-15.58097000	-0.95192200	-11.29411600
C	-17.27742400	0.62623200	-13.79780900
H	-19.34024400	1.27217500	-13.68948600
H	-15.26101000	-0.13682800	-13.61201800
H	-17.13415500	0.98248700	-14.82137300
C	-16.77042200	-2.22666400	-9.19599000
C	-17.18329600	-3.50043000	-9.63179100
C	-15.50541100	-2.10612400	-8.59632700
C	-16.35091700	-4.61268300	-9.49564800

H	-18.17358600	-3.61943500	-10.08191400
C	-14.67686900	-3.22382400	-8.44379200
H	-15.15034500	-1.13928000	-8.23492400
C	-15.09369300	-4.47758700	-8.89659600
H	-16.68928400	-5.59109400	-9.84716700
H	-13.70135000	-3.10942300	-7.96405100
H	-14.44523400	-5.34928600	-8.77605400
C	-18.26119400	-1.61762400	-0.91965900
C	-17.14542600	-1.06482600	-1.46892200
H	-16.42793100	-1.74019700	-1.94495900
C	-16.79176100	0.34012300	-1.50950300
H	-15.70967900	0.51485100	-1.40179700
H	-17.38290100	1.02862000	-0.90251900
C	-18.46138900	-3.10932300	-0.94411200
H	-18.55351200	-3.51418300	0.08039000
H	-19.40289700	-3.37019200	-1.46117300
H	-17.63565200	-3.63067400	-1.44960200
C	-19.35724100	-0.83885900	-0.24780600
H	-19.50194800	-1.19022400	0.78995400
H	-19.17691100	0.24303800	-0.22465600
H	-20.31640600	-1.00180000	-0.76973700

UB3LYP-D3/def2-SVP-SMD(THF)//UB3LYP/def2-SVP(gas)
HF= -4404.1801422
UPBEPBE-D3/def2-SVP-SMD(THF)//UB3LYP/def2-SVP(gas)
HF= -4400.8096930

[1]D'-complex-dimethyl

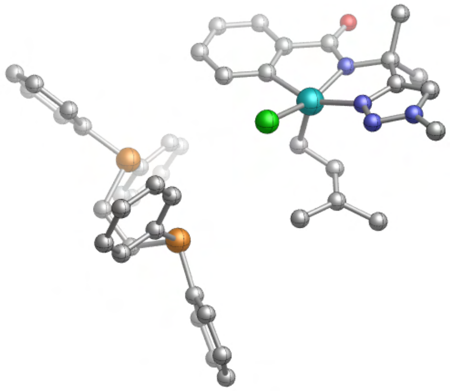

Zero-point correction= 0.807587 (Hartree/Particle)
Thermal correction to Energy= 0.861640
Thermal correction to Enthalpy= 0.862584
Thermal correction to Gibbs Free Energy= 0.705708
Sum of electronic and zero-point Energies= -4403.155416
Sum of electronic and thermal Energies= -4403.101362
Sum of electronic and thermal Enthalpies= -4403.100418
Sum of electronic and thermal Free Energies= -4403.257294

C	-17.93523300	-0.52219400	-5.69822000
C	-17.89282700	0.82129600	-6.10773700
C	-18.64244300	1.28051300	-7.19492700
C	-19.45366300	0.37929800	-7.88987700
C	-19.50438200	-0.96252900	-7.48934600
C	-18.74574200	-1.41920100	-6.40049400
H	-18.58357300	2.33763800	-7.46713000
H	-20.05092000	0.71894000	-8.74042300
H	-20.13831400	-1.66935200	-8.03227100
H	-18.78961900	-2.46985800	-6.11046200
C	-17.05484200	1.73220300	-5.27580800
O	-16.89352500	2.92604400	-5.51262000
C	-15.95004100	1.81639900	-3.06952100
C	-16.92409500	2.90151000	-2.54440200
H	-16.50067400	3.39840000	-1.65657900
H	-17.10221700	3.65189800	-3.32367800
H	-17.88572200	2.44561900	-2.26354500
C	-14.59985800	2.46080100	-3.45800600
H	-14.76691700	3.16877500	-4.28012700
H	-14.16058300	3.00187500	-2.60369400
H	-13.88094300	1.69666800	-3.78949400
C	-15.75848900	0.79475200	-1.97578100
N	-16.14030700	-0.49256700	-2.20383400
N	-15.94464800	-1.23701400	-1.15244500
C	-15.29298500	0.83772900	-0.67588900
H	-14.89941700	1.63984500	-0.05817700
N	-15.43275200	-0.44062200	-0.22195300
N	-16.53852400	1.05122200	-4.19014000
Fe	-16.76988700	-0.82876000	-4.13673300
P	-16.01605100	-6.18410100	-7.19574900

P	-18.31809500	-4.63495500	-9.28437200
C	-16.45178000	-6.81736100	-8.91941100
H	-16.31606500	-7.91108200	-8.96106000
H	-15.70034600	-6.38197100	-9.59828300
C	-17.86285500	-6.45660700	-9.40110100
H	-18.61477100	-6.96734700	-8.77774600
H	-18.01622500	-6.83129700	-10.42741500
C	-17.29799200	-7.02429400	-6.15314800
C	-17.97872400	-6.23896300	-5.20773800
C	-17.63897300	-8.38481000	-6.27313400
C	-18.97489800	-6.79924700	-4.39873200
H	-17.73292100	-5.18004800	-5.09178200
C	-18.63602300	-8.94242000	-5.46984600
H	-17.11597600	-9.01963300	-6.99362000
C	-19.30670000	-8.14935800	-4.53003800
H	-19.48637500	-6.16834300	-3.66735200
H	-18.89068100	-10.00066600	-5.57495600
H	-20.08649600	-8.58808700	-3.90153700
C	-14.49444400	-7.19619900	-6.86453000
C	-14.29801600	-7.87958600	-5.65040800
C	-13.43633000	-7.18320300	-7.79452400
C	-13.09427800	-8.54015700	-5.38452500
H	-15.09611300	-7.90416900	-4.90527300
C	-12.23701300	-7.84976400	-7.53150000
H	-13.54154400	-6.64212500	-8.73882000
C	-12.05998600	-8.53287200	-6.32423500
H	-12.96861900	-9.06952700	-4.43586200
H	-11.43478300	-7.83159200	-8.27433500
H	-11.12161900	-9.05387100	-6.11735500
C	-17.31772400	-3.86058200	-10.63973700
C	-17.07906000	-2.47752400	-10.53531800
C	-16.76843900	-4.55123400	-11.73456600
C	-16.32883100	-1.80303800	-11.50268200
H	-17.48622800	-1.92449900	-9.68396700
C	-16.00699000	-3.87977100	-12.69676900
H	-16.93700500	-5.62440700	-11.85064600
C	-15.78740800	-2.50358900	-12.58475400
H	-16.15994000	-0.72745100	-11.40446700
H	-15.58648500	-4.43502200	-13.53972800
H	-15.19271300	-1.97937300	-13.33752200
C	-20.02153700	-4.72499500	-10.02316500
C	-20.33901400	-4.39684700	-11.35181100
C	-21.06587100	-5.11297500	-9.16140400
C	-21.65986400	-4.46765500	-11.80779400
H	-19.55160500	-4.08082900	-12.03927500
C	-22.38220900	-5.19732100	-9.62102600
H	-20.84626700	-5.34562500	-8.11460200
C	-22.68412400	-4.87207000	-10.94754000
H	-21.88756400	-4.20519300	-12.84466700
H	-23.17702600	-5.50583600	-8.93657100
H	-23.71521200	-4.92675500	-11.30645600

Cl	-17.72989100	-2.78751700	-3.58582300
C	-13.92061100	-2.53364400	-3.97495700
C	-14.37112500	-1.44766000	-4.67606300
H	-13.84465000	-0.50100400	-4.51650600
C	-15.49388700	-1.40500800	-5.59810900
H	-15.44308200	-0.62774100	-6.36388700
H	-15.87605000	-2.35860700	-5.97462200
C	-15.10822400	-0.97440100	1.09006600
H	-15.38976900	-2.03396300	1.09167700
H	-14.03078000	-0.87642300	1.28858100
H	-15.67389800	-0.44215700	1.86849500
C	-14.51806300	-3.90297100	-4.07952000
H	-14.87582200	-4.13333100	-5.09359900
H	-13.79669200	-4.67730600	-3.77811400
H	-15.40275200	-3.96927700	-3.41847500
C	-12.78198600	-2.38301800	-3.00724800
H	-13.10589700	-2.69351100	-1.99676500
H	-11.94483400	-3.04993700	-3.27905700
H	-12.40469000	-1.35127500	-2.95336300

UB3LYP-D3/def2-SVP-SMD(THF)//UB3LYP/def2-SVP(gas)
HF= -4404.1188375
UPBEPBE-D3/def2-SVP-SMD(THF)//UB3LYP/def2-SVP(gas)
HF= -4400.7766071

[3]D'-complex-dimethyl

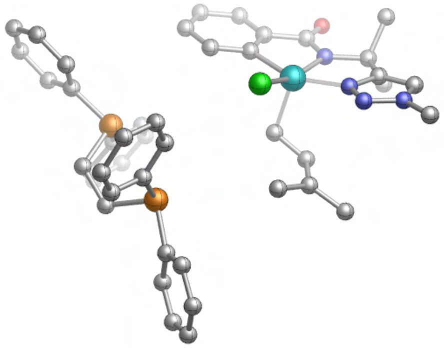

Zero-point correction= 0.806210 (Hartree/Particle)
Thermal correction to Energy= 0.860752
Thermal correction to Enthalpy= 0.861696
Thermal correction to Gibbs Free Energy= 0.703713
Sum of electronic and zero-point Energies= -4403.180749
Sum of electronic and thermal Energies= -4403.126207
Sum of electronic and thermal Enthalpies= -4403.125262
Sum of electronic and thermal Free Energies= -4403.283245

C	-18.15685800	-0.47540900	-5.75122100
C	-18.07196200	0.84992200	-6.20953200
C	-18.78296300	1.28112600	-7.33299100
C	-19.59567200	0.36914100	-8.01315400
C	-19.68968800	-0.95434400	-7.56287200
C	-18.97434400	-1.38212600	-6.43324400
H	-18.69241300	2.32447000	-7.64674400
H	-20.16205700	0.68742800	-8.89269100
H	-20.32620400	-1.66683000	-8.09541600
H	-19.05358200	-2.41716900	-6.09723700
C	-17.22196400	1.77359100	-5.39846700
O	-17.04940700	2.95853400	-5.67034200
C	-15.97894700	1.87831600	-3.26684900
C	-16.86646700	3.00542100	-2.68175100
H	-16.34848000	3.51624200	-1.85367100
H	-17.09696200	3.73735800	-3.46516800
H	-17.80911400	2.58589800	-2.29851500
C	-14.65065000	2.46737600	-3.79559200
H	-14.87048000	3.17612000	-4.60420300
H	-14.10563200	2.99409800	-2.99502100
H	-14.00234800	1.67067200	-4.19028500
C	-15.70518300	0.87603300	-2.17104400
N	-16.12922400	-0.40887400	-2.32366800
N	-15.84243500	-1.12951300	-1.27655500
C	-15.11026400	0.94278600	-0.92554900
H	-14.64165000	1.75192200	-0.37277900
N	-15.22513500	-0.32067400	-0.42498200
N	-16.69699100	1.11334800	-4.30696200
Fe	-16.98840300	-0.77423400	-4.19229200
P	-16.01737600	-6.21200100	-7.08389700
P	-18.28620500	-4.63508400	-9.18236600

C	-16.42845800	-6.82464600	-8.82152300
H	-16.29589300	-7.91846000	-8.87119400
H	-15.66538200	-6.38461700	-9.48428900
C	-17.83097900	-6.45466400	-9.32090500
H	-18.59331400	-6.97349100	-8.71727000
H	-17.96849500	-6.81426600	-10.35487500
C	-17.33603600	-7.03793100	-6.07613400
C	-18.05662400	-6.23938200	-5.17210100
C	-17.66902200	-8.40108100	-6.18859600
C	-19.08494500	-6.78952200	-4.39694000
H	-17.81874000	-5.17796900	-5.06269900
C	-18.69763000	-8.94854300	-5.41889100
H	-17.11499900	-9.04526600	-6.87694000
C	-19.40841900	-8.14238600	-4.52063700
H	-19.62752900	-6.14881000	-3.69718500
H	-18.94570100	-10.00894600	-5.51769300
H	-20.21265300	-8.57312700	-3.91795900
C	-14.52061900	-7.25203700	-6.72820900
C	-14.34736000	-7.91404700	-5.49861700
C	-13.45606100	-7.28162300	-7.65031300
C	-13.16038000	-8.59473100	-5.21003000
H	-15.15111100	-7.90594900	-4.75908400
C	-12.27272400	-7.96705700	-7.36377600
H	-13.54303200	-6.76011600	-8.60721600
C	-12.11899800	-8.62847500	-6.14150000
H	-13.05318400	-9.10715800	-4.24987100
H	-11.46507500	-7.98143200	-8.10080500
H	-11.19350900	-9.16484200	-5.91671000
C	-17.28473100	-3.84007700	-10.52446200
C	-17.07275100	-2.45304900	-10.41347700
C	-16.71012500	-4.51736500	-11.61446200
C	-16.32440200	-1.76214300	-11.37044300
H	-17.50032500	-1.90994900	-9.56577000
C	-15.94998100	-3.82914500	-12.56598000
H	-16.85732000	-5.59311800	-11.73542500
C	-15.75745800	-2.44951700	-12.44803600
H	-16.17722400	-0.68379300	-11.26762400
H	-15.50952000	-4.37423600	-13.40537800
H	-15.16409500	-1.91219800	-13.19256700
C	-19.98817200	-4.71519200	-9.92517900
C	-20.30051300	-4.38496800	-11.25451500
C	-21.03610800	-5.10020800	-9.06650700
C	-21.62035500	-4.45034600	-11.71416000
H	-19.50976800	-4.07161400	-11.93942400
C	-22.35158200	-5.17894900	-9.52981900
H	-20.81976600	-5.33525600	-8.01955000
C	-22.64851800	-4.85131600	-10.85686400
H	-21.84430300	-4.18638200	-12.75146300
H	-23.14949200	-5.48503500	-8.84788200
H	-23.67884400	-4.90161300	-11.21860200
Cl	-17.90370000	-2.73956100	-3.58119800

C	-13.84278500	-2.66394600	-3.96603400
C	-14.28000300	-1.57798300	-4.68595500
H	-13.68835800	-0.65872500	-4.60978900
C	-15.44683300	-1.51947400	-5.51448900
H	-15.47728200	-0.72824900	-6.26677200
H	-15.92419000	-2.45478900	-5.82060500
C	-14.77390000	-0.82826000	0.85978700
H	-15.09216100	-1.87510100	0.92879700
H	-13.67796200	-0.76828200	0.93289000
H	-15.22788400	-0.25042000	1.67767900
C	-14.57315600	-3.97299400	-3.94674600
H	-14.84285300	-4.30997300	-4.96081300
H	-13.97766600	-4.76337100	-3.46742500
H	-15.52852700	-3.86335700	-3.39974900
C	-12.60455100	-2.56548200	-3.12386800
H	-12.84284100	-2.79628100	-2.06899500
H	-11.85746000	-3.31677400	-3.43686100
H	-12.13825800	-1.57013600	-3.16870000

UB3LYP-D3/def2-SVP-SMD(THF)//UB3LYP/def2-SVP(gas)
HF= -4404.1677598
UPBEPBE-D3/def2-SVP-SMD(THF)//UB3LYP/def2-SVP(gas)
HF= -4400.8127106

[5]D'-complex-dimethyl

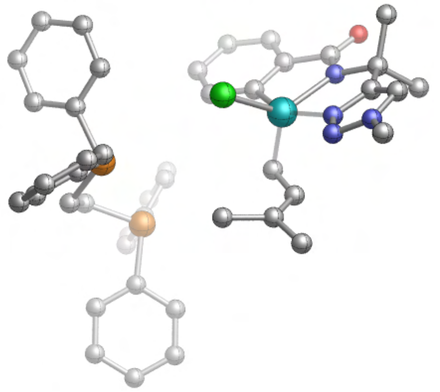

Zero-point correction= 0.806169 (Hartree/Particle)
Thermal correction to Energy= 0.860785
Thermal correction to Enthalpy= 0.861729
Thermal correction to Gibbs Free Energy= 0.704140
Sum of electronic and zero-point Energies= -4403.172527
Sum of electronic and thermal Energies= -4403.117911
Sum of electronic and thermal Enthalpies= -4403.116967
Sum of electronic and thermal Free Energies= -4403.274556

C	-18.39408300	-1.52819900	-4.90077800
C	-19.21820400	-0.49221000	-4.45408500
C	-20.59919900	-0.55403600	-4.68296800
C	-21.14693300	-1.65906900	-5.33663500
C	-20.31383900	-2.70346000	-5.75772100
C	-18.92743500	-2.63830700	-5.55255700
H	-21.21472600	0.27159300	-4.31645400
H	-22.22491900	-1.71626700	-5.50938200
H	-20.74052400	-3.58149000	-6.25066100
H	-18.28772600	-3.45435900	-5.89602900
C	-18.61792700	0.63163100	-3.65767400
O	-19.28220900	1.61187200	-3.31905400
C	-16.61038200	1.30579800	-2.44147900
C	-17.30740900	1.40446500	-1.06333000
H	-16.73041600	2.04699500	-0.37778000
H	-18.31014400	1.83058700	-1.19009200
H	-17.39944300	0.40471500	-0.61221100
C	-16.45768100	2.71393900	-3.06900400
H	-17.45340300	3.14486200	-3.23346000
H	-15.87942400	3.38048000	-2.40801700
H	-15.93624500	2.64623600	-4.03661400
C	-15.23874300	0.70228500	-2.24845100
N	-14.86770700	-0.38418200	-2.97452600
N	-13.65991600	-0.76549200	-2.66778400
C	-14.16630600	1.01009200	-1.43095800
H	-14.00561900	1.78150200	-0.68342600
N	-13.22498100	0.07296000	-1.73355600
N	-17.31174200	0.38206500	-3.34559800
Fe	-16.47610100	-1.26575600	-4.19667500
P	-16.85791100	-6.21416000	-6.62874500

P	-17.50598400	-4.23378000	-9.56297300
C	-17.01889900	-6.82539400	-8.38995900
H	-17.29328700	-7.89275300	-8.42719100
H	-16.00600200	-6.75644700	-8.82078600
C	-18.01887100	-6.01138200	-9.22971900
H	-18.98833400	-5.93630400	-8.70953100
H	-18.23147400	-6.52619000	-10.18196500
C	-18.37707800	-6.89934700	-5.79721900
C	-18.72830800	-6.32378100	-4.56128400
C	-19.18627400	-7.92596700	-6.31269300
C	-19.84555000	-6.77764900	-3.85434700
H	-18.12447900	-5.51002100	-4.14838600
C	-20.31189600	-8.36942600	-5.61121600
H	-18.94495300	-8.39722700	-7.26833300
C	-20.64265600	-7.79957700	-4.37834400
H	-20.09845100	-6.31992500	-2.89444900
H	-20.93065000	-9.16799200	-6.02987900
H	-21.52196300	-8.14832400	-3.83031800
C	-15.54411000	-7.33778100	-5.94280700
C	-14.92176600	-6.92193200	-4.75069900
C	-15.12685000	-8.54323300	-6.53240500
C	-13.92406600	-7.70013400	-4.15710200
H	-15.22197800	-5.97827600	-4.28513700
C	-14.11830000	-9.31380900	-5.94485200
H	-15.58857200	-8.89895000	-7.45604900
C	-13.51673400	-8.89710700	-4.75396300
H	-13.45788500	-7.36341800	-3.22720700
H	-13.80548800	-10.24826400	-6.41903700
H	-12.73102700	-9.50263900	-4.29433300
C	-16.22600900	-4.40701200	-10.89656700
C	-15.48363400	-3.25057800	-11.20378600
C	-15.92072300	-5.59274300	-11.58661200
C	-14.48933700	-3.27066000	-12.18382800
H	-15.69048000	-2.32206600	-10.66308900
C	-14.91414500	-5.61886000	-12.55890100
H	-16.46954600	-6.51281300	-11.37501100
C	-14.19899600	-4.45829800	-12.86391500
H	-13.93201300	-2.35798100	-12.41127000
H	-14.69155900	-6.55290900	-13.08192300
H	-13.41440000	-4.47950100	-13.62468800
C	-18.99624200	-3.67367200	-10.52332600
C	-19.20226600	-3.93067500	-11.88971300
C	-19.97584900	-2.95171100	-9.81829300
C	-20.36312900	-3.48691600	-12.52980800
H	-18.44855700	-4.47665000	-12.46223700
C	-21.14120800	-2.51640400	-10.45655500
H	-19.82297800	-2.72240600	-8.75972100
C	-21.33687600	-2.78202700	-11.81483800
H	-20.50722800	-3.69260300	-13.59407500
H	-21.89208000	-1.95823200	-9.89110300
H	-22.24334400	-2.43490400	-12.31775700

Cl	-16.14146700	-3.35349700	-3.36855700
C	-13.89693000	-1.69738000	-6.07845000
C	-14.93659100	-0.79850100	-6.09335000
H	-14.67898700	0.25308500	-5.92212900
C	-16.34172100	-1.09061300	-6.30006700
H	-16.95788800	-0.25546800	-6.64254800
H	-16.59885000	-2.04178300	-6.77391200
C	-11.89911600	-0.09304000	-1.16383600
H	-11.27400500	0.78578000	-1.38067400
H	-11.96749600	-0.23389000	-0.07538700
H	-11.45337800	-0.98462400	-1.62010600
C	-14.05919800	-3.17265500	-6.27519500
H	-15.02676600	-3.46036200	-6.70558900
H	-13.25274900	-3.56898400	-6.91409200
H	-13.98445400	-3.68138100	-5.29760900
C	-12.49291400	-1.22223700	-5.84291000
H	-12.06470200	-1.72940900	-4.96117300
H	-11.84750600	-1.48002700	-6.70177700
H	-12.43887400	-0.13679400	-5.67710500

UB3LYP-D3/def2-SVP-SMD(THF)//UB3LYP/def2-SVP(gas)
HF= -4404.1659633
UPBEPBE-D3/def2-SVP-SMD(THF)//UB3LYP/def2-SVP(gas)
HF= -4400.7974280

[1]D’-noligand-dimethyl

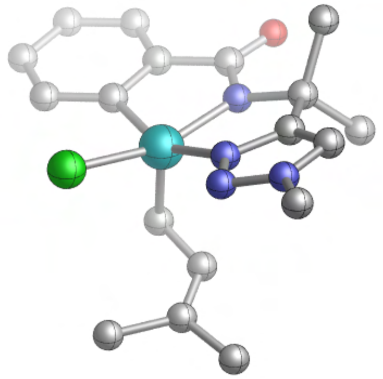

Zero-point correction= 0.384613 (Hartree/Particle)
Thermal correction to Energy= 0.411006
Thermal correction to Enthalpy= 0.411951
Thermal correction to Gibbs Free Energy= 0.328253
Sum of electronic and zero-point Energies= -2716.545927
Sum of electronic and thermal Energies= -2716.519533
Sum of electronic and thermal Enthalpies= -2716.518589
Sum of electronic and thermal Free Energies= -2716.602287

C	-18.56469500	-1.35243200	-4.52430700
C	-19.18423600	-0.09170700	-4.51266300
C	-20.53528300	0.06248500	-4.83787800
C	-21.28417200	-1.06645100	-5.18089200
C	-20.67674900	-2.32904900	-5.19143900
C	-19.31999000	-2.47810300	-4.86858800
H	-20.97587200	1.06237000	-4.80493000
H	-22.34269700	-0.96708400	-5.43588300
H	-21.26637600	-3.21201100	-5.45595700
H	-18.85460700	-3.46577000	-4.87927900
C	-18.33135600	1.05054200	-4.07087200
O	-18.71213500	2.21631000	-4.02550300
C	-16.17874000	1.50014600	-2.94293600
C	-16.87034600	2.10921500	-1.69693500
H	-16.15055500	2.70535800	-1.11300100
H	-17.69954400	2.75514500	-2.00874500
H	-17.26512700	1.30971500	-1.05173000
C	-15.62607600	2.62692100	-3.84534600
H	-16.46215500	3.24399000	-4.19952500
H	-14.92026900	3.26618000	-3.28990600
H	-15.10052900	2.20900100	-4.71711800
C	-15.06105400	0.60492700	-2.46647800
N	-15.10160500	-0.72077000	-2.77650700
N	-14.08776300	-1.35779400	-2.26237600
C	-13.92789900	0.78992800	-1.69829600
H	-13.49359300	1.66318300	-1.22009600
N	-13.37202100	-0.45210000	-1.60769900
N	-17.08472300	0.59612900	-3.68347800
Fe	-16.66776900	-1.21801700	-4.02311900
Cl	-16.44602800	-3.40679700	-3.61954100

C	-13.97695600	-1.78000900	-6.01184000
C	-14.99544500	-0.87002100	-5.95681500
H	-14.71714500	0.18107900	-5.82616100
C	-16.42110800	-1.15076000	-6.02780000
H	-17.04266500	-0.34252100	-6.42083500
H	-16.71726500	-2.13728300	-6.39462600
C	-12.16704200	-0.85332000	-0.90116800
H	-12.04427500	-1.93258700	-1.04936900
H	-11.29231800	-0.32126700	-1.30290900
H	-12.26514100	-0.63837500	0.17287100
C	-14.18788400	-3.25306700	-6.19673300
H	-15.01996300	-3.47449000	-6.88121400
H	-13.27954200	-3.74357000	-6.57786200
H	-14.45905100	-3.71271000	-5.22731100
C	-12.55345600	-1.33249800	-5.84170200
H	-12.10407900	-1.83917200	-4.96764900
H	-11.93976500	-1.62060200	-6.71342200
H	-12.46681900	-0.24563700	-5.69671900

UB3LYP-D3/def2-SVP-SMD(THF)//UB3LYP/def2-SVP(gas)
HF= -2716.9986940
UPBEPBE-D3/def2-SVP-SMD(THF)//UB3LYP/def2-SVP(gas)
HF= -2715.3302346

^{3}D’-noligand-dimethyl

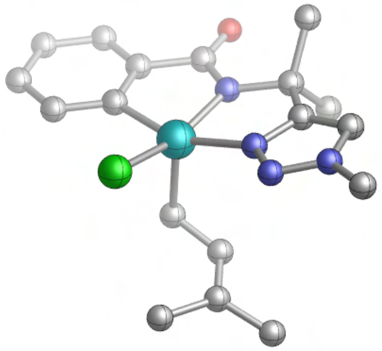

Zero-point correction= 0.383131 (Hartree/Particle)
Thermal correction to Energy= 0.410072
Thermal correction to Enthalpy= 0.411017
Thermal correction to Gibbs Free Energy= 0.324433
Sum of electronic and zero-point Energies= -2716.571297
Sum of electronic and thermal Energies= -2716.544356
Sum of electronic and thermal Enthalpies= -2716.543412
Sum of electronic and thermal Free Energies= -2716.629995

C	-18.68158200	-1.34033400	-4.48373900
C	-19.28226800	-0.07097400	-4.48496400
C	-20.60512500	0.10949400	-4.89856000
C	-21.34217000	-1.00112400	-5.31996900
C	-20.75353300	-2.27221900	-5.31755600
C	-19.42589200	-2.44923400	-4.89928200
H	-21.03350000	1.11502200	-4.87518600
H	-22.37860500	-0.88027900	-5.64662700
H	-21.33523500	-3.13984700	-5.64328600
H	-18.97536600	-3.44398000	-4.89612300
C	-18.44049800	1.05395000	-3.97450500
O	-18.81955900	2.22006400	-3.91706000
C	-16.27560400	1.49806000	-2.86934500
C	-16.92262800	2.11753400	-1.60546800
H	-16.18939800	2.73162300	-1.05761800
H	-17.77112800	2.74837300	-1.89627400
H	-17.28358500	1.32300900	-0.93477100
C	-15.75844900	2.61492500	-3.80554700
H	-16.60689800	3.22933200	-4.13340300
H	-15.02894500	3.25866900	-3.28716900
H	-15.27077400	2.18281600	-4.69257300
C	-15.13060300	0.61567000	-2.43209400
N	-15.15548700	-0.70904400	-2.74629000
N	-14.11166000	-1.32828400	-2.27186900
C	-13.97394400	0.81960900	-1.70411100
H	-13.53931500	1.69928900	-1.23817300
N	-13.39024200	-0.41146500	-1.64001800
N	-17.20866400	0.58697400	-3.56340300
Fe	-16.79763600	-1.24911900	-3.91315500
Cl	-16.55101800	-3.45365300	-3.59972000
C	-13.79587300	-1.84082100	-6.01234300

C	-14.78769000	-0.89376600	-5.94092900
H	-14.47430900	0.15000700	-5.82543600
C	-16.19960800	-1.13709900	-5.98465000
H	-16.83664400	-0.30279000	-6.28643700
H	-16.55115700	-2.11771400	-6.31580300
C	-12.15155400	-0.79059900	-0.98108100
H	-12.01742400	-1.86836600	-1.13037200
H	-11.30236600	-0.24613400	-1.41929600
H	-12.21046600	-0.57278000	0.09517900
C	-14.08734000	-3.30645600	-6.15840200
H	-14.63417000	-3.67550200	-5.27047400
H	-14.73739700	-3.50674800	-7.02639100
H	-13.16614200	-3.89454500	-6.27921600
C	-12.35286900	-1.43996200	-5.91787900
H	-11.87846300	-1.93325400	-5.04930500
H	-11.78945900	-1.77914000	-6.80558400
H	-12.22163800	-0.35254300	-5.81600500

UB3LYP-D3/def2-SVP-SMD(THF)//UB3LYP/def2-SVP(gas)
HF= -2717.0464797
UPBEPBE-D3/def2-SVP-SMD(THF)//UB3LYP/def2-SVP(gas)
HF= -2715.3646042

[5]D'-noligand-dimethyl

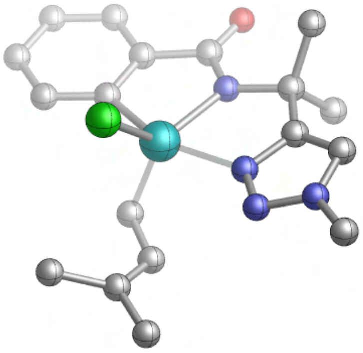

Zero-point correction= 0.383020 (Hartree/Particle)
Thermal correction to Energy= 0.410019
Thermal correction to Enthalpy= 0.410963
Thermal correction to Gibbs Free Energy= 0.323260
Sum of electronic and zero-point Energies= -2716.564303
Sum of electronic and thermal Energies= -2716.537304
Sum of electronic and thermal Enthalpies= -2716.536360
Sum of electronic and thermal Free Energies= -2716.624063

C	-18.64940800	-1.14794800	-4.85087600
C	-19.31875600	0.02219200	-4.48573500
C	-20.68407500	0.16059200	-4.76876300
C	-21.37235700	-0.87924100	-5.39571700
C	-20.69961200	-2.06259400	-5.72675800
C	-19.33129000	-2.20218300	-5.45673300
H	-21.17748800	1.08829400	-4.46738300
H	-22.43800400	-0.77741900	-5.61690000
H	-21.24111300	-2.88711000	-6.19946900
H	-18.81964200	-3.13492500	-5.70657900
C	-18.57224000	1.07904800	-3.72109300
O	-19.09421400	2.15145700	-3.41664500
C	-16.46270200	1.52912600	-2.56772400
C	-17.08031400	1.75821500	-1.16812700
H	-16.40628700	2.35621300	-0.53260000
H	-18.03264500	2.29250600	-1.27500300
H	-17.26737700	0.79373700	-0.67206200
C	-16.16896900	2.88471100	-3.25833600
H	-17.10942900	3.43304200	-3.39458200
H	-15.47847900	3.49328700	-2.65158800
H	-15.70956800	2.71625600	-4.24477200
C	-15.16287300	0.77380400	-2.41929400
N	-14.95718700	-0.36413500	-3.13342800
N	-13.78292500	-0.87316600	-2.88369100
C	-14.01663600	0.97561800	-1.67241300
H	-13.72625200	1.74143600	-0.95903700
N	-13.20488300	-0.06836900	-2.00098300
N	-17.31083200	0.66464100	-3.40099600
Fe	-16.73444800	-1.13833700	-4.13727900
Cl	-16.78019500	-3.11837300	-3.07105500
C	-14.43854400	-2.73612700	-5.99068700

C	-14.99217900	-1.48264900	-6.02801400
H	-14.31358000	-0.63937700	-5.86330500
C	-16.39473700	-1.16251700	-6.21836200
H	-16.62287500	-0.14060800	-6.53963500
H	-17.01404700	-1.90965200	-6.71890000
C	-11.87545000	-0.37119600	-1.49850700
H	-11.54112400	-1.29426300	-1.98611800
H	-11.18035500	0.44707400	-1.73677800
H	-11.90542500	-0.52148700	-0.40954600
C	-15.23582400	-3.99532700	-6.15764600
H	-16.11955300	-3.85713000	-6.79832500
H	-14.62041900	-4.80495300	-6.57977200
H	-15.60701900	-4.32577700	-5.16912200
C	-12.97276700	-2.91386800	-5.72323400
H	-12.82719100	-3.52916900	-4.81706000
H	-12.48367000	-3.45643900	-6.55218700
H	-12.45699400	-1.95568600	-5.57093800

UB3LYP-D3/def2-SVP-SMD(THF)//UB3LYP/def2-SVP(gas)
HF= -2717.0432373
UPBEPBE-D3/def2-SVP-SMD(THF)//UB3LYP/def2-SVP(gas)
HF= -2715.3503384

[1]D'-TS-dimethyl

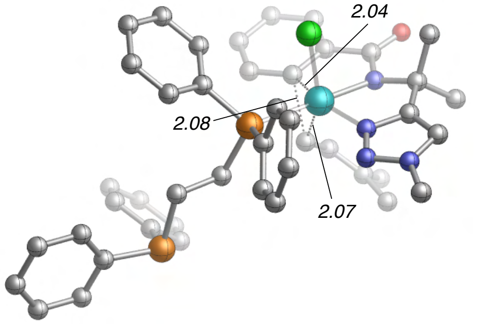

Zero-point correction= 0.807670 (Hartree/Particle)
Thermal correction to Energy= 0.860523
Thermal correction to Enthalpy= 0.861468
Thermal correction to Gibbs Free Energy= 0.714348
Sum of electronic and zero-point Energies= -4403.126455
Sum of electronic and thermal Energies= -4403.073602
Sum of electronic and thermal Enthalpies= -4403.072658
Sum of electronic and thermal Free Energies= -4403.219778

C	-19.99809800	-2.58392100	-3.40175400
C	-20.54996400	-1.48172600	-2.72050000
C	-21.80485100	-1.56206800	-2.11225000
C	-22.54750100	-2.74279100	-2.19268200
C	-22.02433200	-3.83658100	-2.89260000
C	-20.76837800	-3.75279600	-3.50538300
H	-22.16780900	-0.68222200	-1.57513200
H	-23.53145700	-2.81101500	-1.72154700
H	-22.60069500	-4.76263100	-2.97471200
H	-20.40679900	-4.61306900	-4.07504100
C	-19.72347100	-0.24231500	-2.61235900
O	-20.14199400	0.78185600	-2.06642000
C	-17.44541700	0.59528900	-2.89364700
C	-17.17361900	0.77531900	-1.37760900
H	-16.35472600	1.49616000	-1.21739000
H	-18.08187000	1.15510600	-0.89217000
H	-16.90353200	-0.18921800	-0.92669300
C	-17.80747700	1.95947200	-3.52727700
H	-18.73108100	2.33234900	-3.06889900
H	-17.00009100	2.69148700	-3.35795400
H	-17.96335700	1.85716000	-4.61074000
C	-16.20078800	0.05986500	-3.55150300
N	-16.17070800	-1.22106500	-4.01593100
N	-15.01997100	-1.48742100	-4.57012800
C	-14.96341000	0.60661100	-3.83289600
H	-14.53120700	1.58512100	-3.64571300
N	-14.27998800	-0.39319100	-4.45764100
N	-18.48322000	-0.42436900	-3.14981300
Fe	-18.00842700	-2.22085700	-3.67615100
Cl	-17.48203700	-2.84160600	-1.47757300
C	-19.97617500	-0.09981400	-6.54837500
C	-20.23274600	-1.19634700	-5.80277600

H	-21.28111100	-1.41424700	-5.57527900
C	-19.27131800	-2.20536300	-5.31164500
H	-19.54958900	-3.20610500	-5.65365500
H	-18.23464100	-1.99116300	-5.67050200
C	-12.93062800	-0.37085100	-4.99496200
H	-12.88133000	0.26186300	-5.89421300
H	-12.23298900	0.01519900	-4.23843600
H	-12.66049600	-1.40230400	-5.25081300
P	-17.11475900	-4.52828300	-4.20544600
C	-17.60300700	-5.16198600	-5.90245800
H	-17.23002100	-4.40436300	-6.61395500
H	-18.69962600	-5.06713600	-5.92801900
C	-17.21858300	-6.57514800	-6.34992000
H	-17.73572100	-7.32374200	-5.73118300
H	-16.13666800	-6.74653700	-6.23865600
P	-17.60690000	-6.83245400	-8.17242100
C	-19.46602800	-6.79994700	-8.12910600
C	-20.11706900	-5.71038000	-8.73186700
C	-20.24629800	-7.79250900	-7.50905900
C	-21.51237200	-5.60465300	-8.70459200
H	-19.52376100	-4.93917900	-9.23155900
C	-21.63908100	-7.69036100	-7.48521300
H	-19.76191500	-8.65712700	-7.04751100
C	-22.27521800	-6.59496300	-8.08115000
H	-22.00283700	-4.74896500	-9.17604300
H	-22.23247100	-8.47021500	-7.00043100
H	-23.36534300	-6.51703700	-8.06158000
C	-17.27182900	-8.64071200	-8.39645100
C	-17.55017200	-9.18742800	-9.66475500
C	-16.69317200	-9.47567700	-7.42572900
C	-17.28001800	-10.52682600	-9.94632700
H	-17.98691500	-8.55300500	-10.44201500
C	-16.41016800	-10.81684800	-7.71237400
H	-16.45855900	-9.09247400	-6.43105700
C	-16.70509500	-11.34784700	-8.96927900
H	-17.51186400	-10.93075100	-10.93541400
H	-15.95960300	-11.44906100	-6.94250700
H	-16.48571900	-12.39560600	-9.18992600
C	-17.48381400	-5.95702700	-3.08024800
C	-18.53982200	-5.90233300	-2.15773600
C	-16.70236000	-7.12686100	-3.14373700
C	-18.83178200	-7.00555300	-1.34880700
H	-19.11131100	-4.98423400	-2.04108100
C	-16.99389800	-8.22549400	-2.33196400
H	-15.84357600	-7.17813900	-3.81528800
C	-18.06655200	-8.17015000	-1.43631800
H	-19.65594600	-6.94102000	-0.63393000
H	-16.37380000	-9.12375100	-2.39376800
H	-18.29327400	-9.02787800	-0.79745800
C	-15.26759000	-4.54869900	-4.22205400
C	-14.50862200	-4.65263400	-5.39769500

C	-14.59916000	-4.39009100	-2.99373800
C	-13.11046400	-4.62582200	-5.34760900
H	-14.99310300	-4.74686300	-6.37110200
C	-13.20456400	-4.35962100	-2.94751400
H	-15.18175300	-4.25979200	-2.07840400
C	-12.45398600	-4.48360600	-4.12251400
H	-12.53558200	-4.72242500	-6.27275000
H	-12.70059900	-4.23815800	-1.98492900
H	-11.36128300	-4.47272900	-4.08187000
C	-21.10387900	0.78248900	-7.01876700
H	-20.99061900	1.80505300	-6.61620700
H	-21.10306100	0.87869600	-8.11966100
H	-22.08608100	0.40099800	-6.70479500
C	-18.60812900	0.33112300	-7.00851700
H	-17.78806000	-0.22132500	-6.53151900
H	-18.51344700	0.20366000	-8.10249000
H	-18.45277700	1.40468400	-6.80883300

UB3LYP-D3/def2-SVP-SMD(THF)//UB3LYP/def2-SVP(gas)
HF= -4404.1226755
UPBEPBE-D3/def2-SVP-SMD(THF)//UB3LYP/def2-SVP(gas)
HF= -4400.7724656

^{3}D'-TS-dimethyl

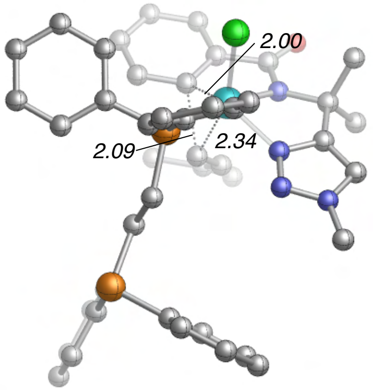

Zero-point correction= 0.808353 (Hartree/Particle)
Thermal correction to Energy= 0.861327
Thermal correction to Enthalpy= 0.862272
Thermal correction to Gibbs Free Energy= 0.714282
Sum of electronic and zero-point Energies= -4403.148696
Sum of electronic and thermal Energies= -4403.095722
Sum of electronic and thermal Enthalpies= -4403.094777
Sum of electronic and thermal Free Energies= -4403.242767

C	-20.18679600	-2.74663300	-4.86197600
C	-20.74308500	-1.61097800	-4.22511800
C	-22.08064900	-1.59312400	-3.81654700
C	-22.89336000	-2.70580000	-4.03425000
C	-22.35548700	-3.84033100	-4.66081900
C	-21.02453500	-3.85785500	-5.08023300
H	-22.44815100	-0.69646500	-3.31112000
H	-23.93910300	-2.69805500	-3.71619600
H	-22.98275700	-4.71964800	-4.83293000
H	-20.64363100	-4.74584900	-5.58957400
C	-19.83630900	-0.46516600	-3.91609500
O	-20.22655400	0.61086200	-3.46299900
C	-17.42089200	0.10644500	-3.91600300
C	-17.22204500	0.31879500	-2.39639400
H	-16.34659300	0.96002600	-2.20062700
H	-18.11844400	0.80646100	-1.98907000
H	-17.08838400	-0.64848200	-1.89252600
C	-17.60237900	1.47062300	-4.62217400
H	-18.50589400	1.95869900	-4.23831900
H	-16.73106600	2.11837300	-4.43034800
H	-17.70470900	1.34120800	-5.71038700
C	-16.21939200	-0.59484200	-4.50916300
N	-16.34501800	-1.88209500	-4.93718500
N	-15.22873500	-2.31617200	-5.45695400
C	-14.91681400	-0.22253000	-4.78402300
H	-14.36659900	0.70052400	-4.62704700
N	-14.35766400	-1.31941000	-5.37017000
N	-18.54354700	-0.81008600	-4.18818700
Fe	-18.23142400	-2.66597900	-4.44721700
P	-17.41079100	-5.04906900	-4.93913200

C	-16.58861200	-5.41363400	-6.58854200
H	-16.25592100	-6.46408600	-6.55269300
H	-15.67469700	-4.80000800	-6.55395700
C	-18.63945500	-6.40922000	-4.70084900
C	-19.36613600	-6.42028200	-3.49453900
C	-18.87533900	-7.42129000	-5.64619500
C	-20.30006300	-7.42685400	-3.24433500
H	-19.19559800	-5.63255500	-2.75544900
C	-19.82118500	-8.42248500	-5.39409200
H	-18.32252600	-7.45079600	-6.58638500
C	-20.53380900	-8.42849500	-4.19333600
H	-20.85352600	-7.42369600	-2.30189900
H	-19.99423500	-9.20162400	-6.14109200
H	-21.27001700	-9.21213300	-3.99617900
C	-16.01115100	-5.62935900	-3.85583300
C	-15.25574500	-4.73769100	-3.07969000
C	-15.66132800	-6.99155200	-3.85288900
C	-14.16532200	-5.19737400	-2.33449600
H	-15.54705300	-3.69143800	-3.02121700
C	-14.57180800	-7.44867600	-3.10752900
H	-16.25092900	-7.71213200	-4.42465600
C	-13.81701800	-6.55003200	-2.34813400
H	-13.59513200	-4.49121600	-1.72497700
H	-14.31916500	-8.51221800	-3.11471000
H	-12.96820400	-6.90642600	-1.75843400
Cl	-18.07142700	-3.27429300	-2.19949000
C	-20.69646900	-0.84845700	-7.80298900
C	-19.55576300	-1.12189900	-7.12833100
H	-18.87995600	-0.28343000	-6.93954200
C	-19.09673200	-2.42035700	-6.61221800
H	-19.58012500	-3.29100800	-7.05730900
H	-18.01309600	-2.50661100	-6.73806200
P	-16.47583200	-5.90347100	-9.35879700
C	-17.51505600	-5.33728400	-10.78464600
C	-17.21591100	-5.90112900	-12.04006000
C	-18.59299300	-4.43925200	-10.69813800
C	-17.95404300	-5.56297200	-13.17548200
H	-16.39351100	-6.61784200	-12.12562700
C	-19.34190600	-4.11016200	-11.83407800
H	-18.86141500	-3.97964600	-9.74518000
C	-19.02341200	-4.66635700	-13.07493500
H	-17.70042600	-6.00869600	-14.14093900
H	-20.17772700	-3.41127100	-11.74437300
H	-19.60898300	-4.40694900	-13.96066600
C	-17.35478100	-5.13170300	-7.88575700
H	-17.47551200	-4.05115100	-8.03752100
H	-18.36379300	-5.57467500	-7.86803500
C	-14.99790000	-4.77725000	-9.49074800
C	-13.76204000	-5.26986500	-9.03744200
C	-15.05103100	-3.47194900	-10.01104600
C	-12.61070600	-4.47611100	-9.08718800

H	-13.69923300	-6.29039200	-8.64908800
C	-13.90091700	-2.68055600	-10.06940800
H	-15.99735900	-3.07216100	-10.38380100
C	-12.67746100	-3.17989200	-9.60675300
H	-11.65742900	-4.87889200	-8.73429700
H	-13.95820700	-1.67111100	-10.48590200
H	-11.77560000	-2.56431100	-9.66608600
C	-12.99057100	-1.50849600	-5.82764600
H	-12.36903900	-1.92802300	-5.02202400
H	-13.00049300	-2.20009200	-6.67969700
H	-12.57830000	-0.54125300	-6.14315200
C	-20.98743500	0.55034200	-8.28156400
H	-21.12792200	0.57780500	-9.37768800
H	-21.92735300	0.92721700	-7.83916000
H	-20.18394800	1.25388900	-8.01861000
C	-21.76281800	-1.86121700	-8.12427700
H	-21.97602200	-1.87439400	-9.20869100
H	-21.50884500	-2.88101700	-7.80851700
H	-22.70934400	-1.58994200	-7.62434900

UB3LYP-D3/def2-SVP-SMD(THF)//UB3LYP/def2-SVP(gas)
HF= -4404.1608729
UPBEPBE-D3/def2-SVP-SMD(THF)//UB3LYP/def2-SVP(gas)
HF= -4400.8043464

^{5}D'-TS-dimethyl

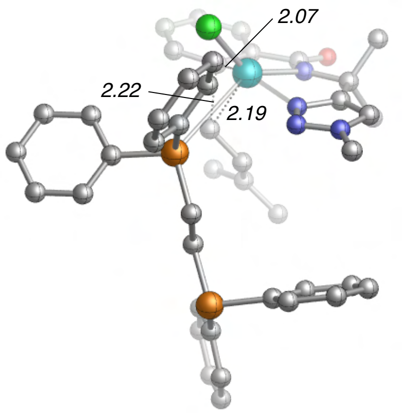

Zero-point correction= 0.806232 (Hartree/Particle)
Thermal correction to Energy= 0.860195
Thermal correction to Enthalpy= 0.861139
Thermal correction to Gibbs Free Energy= 0.706584
Sum of electronic and zero-point Energies= -4403.168981
Sum of electronic and thermal Energies= -4403.115017
Sum of electronic and thermal Enthalpies= -4403.114073
Sum of electronic and thermal Free Energies= -4403.268628

C	-20.77438800	-1.77511600	-3.96451600
C	-20.97647700	-0.38336200	-3.94700700
C	-22.21478300	0.12823800	-3.53181100
C	-23.22716200	-0.72933100	-3.10174500
C	-22.99863100	-2.11117400	-3.06703500
C	-21.77807100	-2.63485200	-3.50422000
H	-22.33618600	1.21444300	-3.53272300
H	-24.18883400	-0.32467600	-2.77597200
H	-23.77763600	-2.78893500	-2.70708900
H	-21.61508000	-3.71587500	-3.48790800
C	-19.84332300	0.57381500	-4.24107800
O	-20.04836900	1.77745500	-4.40205600
C	-17.40196700	0.66158700	-4.53396100
C	-17.01792700	1.58853800	-3.35820100
H	-16.06168200	2.10404100	-3.54757300
H	-17.80512300	2.34426000	-3.22945200
H	-16.92692300	1.00968300	-2.42660400
C	-17.47842200	1.47592900	-5.84984100
H	-18.24207100	2.25694400	-5.75538600
H	-16.50398600	1.94133500	-6.07072800
H	-17.74954700	0.82072900	-6.69133300
C	-16.35226800	-0.41931800	-4.71213900
N	-16.70369200	-1.73546300	-4.67817000
N	-15.66517400	-2.50338000	-4.87014900
C	-14.98892700	-0.39025000	-4.94536200
H	-14.27647600	0.42293100	-5.04867600
N	-14.62462700	-1.69997000	-5.03186600
N	-18.64276200	-0.06443400	-4.24180700
Fe	-18.72522600	-2.03801400	-3.88224800

P	-17.08960500	-5.84713700	-5.18862600
C	-16.19113200	-6.05932300	-6.82016300
H	-15.83477700	-7.09317800	-6.96469000
H	-15.29048800	-5.43179600	-6.71643800
C	-18.27566400	-7.27389100	-5.14593600
C	-19.23085200	-7.24416600	-4.11050200
C	-18.31801800	-8.33455200	-6.06605600
C	-20.18504800	-8.25630700	-3.98904100
H	-19.22070900	-6.41486400	-3.39549000
C	-19.28417500	-9.34117000	-5.95206300
H	-17.59453300	-8.38951800	-6.88323400
C	-20.21700900	-9.30720200	-4.91267500
H	-20.91241200	-8.22075300	-3.17351800
H	-19.30348800	-10.15741100	-6.67945200
H	-20.96944100	-10.09521200	-4.82394400
C	-15.77449000	-6.42992200	-4.00944400
C	-15.39876200	-5.57024000	-2.96353500
C	-15.14412400	-7.68197900	-4.11901400
C	-14.40166900	-5.95116500	-2.05723500
H	-15.90880800	-4.61129100	-2.84762500
C	-14.14734400	-8.05923900	-3.21630400
H	-15.44202700	-8.37818200	-4.90760200
C	-13.77159500	-7.19168000	-2.18402500
H	-14.12654000	-5.27724800	-1.24123800
H	-13.66731000	-9.03699900	-3.31287800
H	-12.99572000	-7.48960900	-1.47349500
Cl	-18.42249300	-3.42598300	-2.14349600
C	-20.34218400	-1.50493200	-7.63583100
C	-19.45885000	-2.06350700	-6.77117000
H	-18.39519500	-1.87855400	-6.95251000
C	-19.77400300	-2.88978800	-5.60242700
H	-20.74815800	-3.37413400	-5.62857300
H	-19.00431500	-3.65715800	-5.38390900
P	-16.11063900	-5.80204100	-9.64393900
C	-17.31339500	-5.10905000	-10.87255700
C	-16.93396600	-5.15898600	-12.22833200
C	-18.58821200	-4.60835500	-10.55704600
C	-17.78866600	-4.70372300	-13.23312400
H	-15.95380800	-5.56346100	-12.49877700
C	-19.45195000	-4.16595100	-11.56667000
H	-18.92412100	-4.55326200	-9.52013700
C	-19.05517300	-4.20709300	-12.90487400
H	-17.46996900	-4.74662900	-14.27801000
H	-20.44199200	-3.78608300	-11.30017400
H	-19.73090700	-3.85983400	-13.69067600
C	-17.02477100	-5.58968700	-8.01847800
H	-17.33724700	-4.54177200	-7.87765600
H	-17.94343600	-6.19378100	-8.10535000
C	-14.86700400	-4.41989100	-9.52334100
C	-13.53288300	-4.76580300	-9.24697100
C	-15.18529400	-3.06109000	-9.69815100

C	-12.54293800	-3.78262900	-9.13761200
H	-13.26545200	-5.81980200	-9.12905200
C	-14.19761700	-2.07775000	-9.59653700
H	-16.21314300	-2.76968300	-9.92893700
C	-12.87305000	-2.43569400	-9.31612300
H	-11.50924400	-4.07246000	-8.93005000
H	-14.46059800	-1.02701000	-9.74680300
H	-12.09822500	-1.66661200	-9.25300800
C	-13.31325600	-2.26244600	-5.31679000
H	-13.15797300	-2.34544800	-6.40317400
H	-12.53967000	-1.61873100	-4.87779700
H	-13.26560400	-3.25987700	-4.86282900
C	-21.83723500	-1.61358100	-7.51021000
H	-22.28015300	-0.60994500	-7.38491200
H	-22.27585200	-2.03419800	-8.43271700
H	-22.16449400	-2.22456600	-6.65966500
C	-19.85818800	-0.69833500	-8.81143500
H	-20.22092200	-1.12658300	-9.76306500
H	-20.24950500	0.33380600	-8.76520100
H	-18.76040800	-0.64762800	-8.85656200

UB3LYP-D3/def2-SVP-SMD(THF)//UB3LYP/def2-SVP(gas)
HF= -4404.1643711
UPBEPBE-D3/def2-SVP-SMD(THF)//UB3LYP/def2-SVP(gas)
HF= -4400.7900354

¹D'-complex-TS-dimethyl

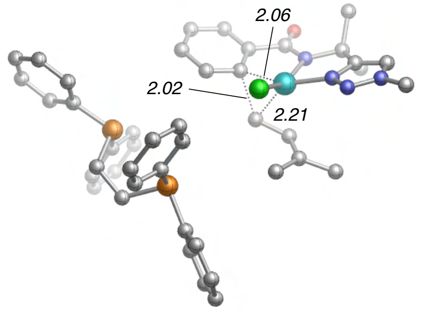

Zero-point correction=	0.807845 (Hartree/Particle)		
Thermal correction to Energy=	0.861045		
Thermal correction to Enthalpy=	0.861989		
Thermal correction to Gibbs Free Energy=	0.708838		
Sum of electronic and zero-point Energies=	-4403.149259		
Sum of electronic and thermal Energies=	-4403.096059		
Sum of electronic and thermal Enthalpies=	-4403.095115		
Sum of electronic and thermal Free Energies=	-4403.248266		
C	-17.44785900	-0.66258200	-5.78872500
C	-17.58085100	0.71420500	-6.06561900
C	-18.52133600	1.17214900	-6.99182100
C	-19.35416500	0.26070600	-7.64706700
C	-19.24462200	-1.10493800	-7.35821000
C	-18.29091600	-1.56845700	-6.44344100
H	-18.59470100	2.24966400	-7.15998900
H	-20.09359300	0.61164500	-8.37169300
H	-19.89773300	-1.82714100	-7.85507100
H	-18.20600000	-2.63866600	-6.24974900
C	-16.79925800	1.67982600	-5.22948700
O	-16.71368700	2.87982100	-5.48760600
C	-15.80272200	1.84832100	-2.98519800
C	-16.84347800	2.89259600	-2.51026600
H	-16.48075500	3.41788600	-1.61173400
H	-17.02337400	3.62511000	-3.30645200
H	-17.79461500	2.39624300	-2.26423300
C	-14.46499600	2.54680600	-3.31931600
H	-14.62623900	3.23442800	-4.16016600
H	-14.08728100	3.11946200	-2.45599100
H	-13.69927500	1.80959600	-3.60594600
C	-15.61392300	0.83480500	-1.88212000
N	-15.92439800	-0.47123400	-2.12878300
N	-15.73417100	-1.21080800	-1.07183100
C	-15.20698000	0.89226300	-0.56387400
H	-14.88026900	1.71038700	0.07159000
N	-15.30174100	-0.39340200	-0.11924700
N	-16.29986900	1.04864300	-4.12194500
Fe	-16.44799400	-0.86322000	-4.00415100
P	-16.28067700	-6.12151300	-7.14757000
P	-18.51314200	-4.69012400	-9.40720200
C	-16.69321800	-6.86464300	-8.83138400

H	-16.56486400	-7.95967400	-8.80353900
H	-15.91658500	-6.48221900	-9.51406200
C	-18.08797100	-6.52262900	-9.37048900
H	-18.86309400	-6.97347400	-8.72915200
H	-18.22485300	-6.96971600	-10.36968400
C	-17.48572400	-6.97728300	-6.03078800
C	-18.01067000	-6.22789900	-4.96323500
C	-17.91851400	-8.30610900	-6.19905000
C	-18.93662300	-6.79562200	-4.08066600
H	-17.70233900	-5.18947400	-4.81021200
C	-18.84824700	-8.87010400	-5.32167700
H	-17.52365200	-8.91371700	-7.01752600
C	-19.35863000	-8.11516700	-4.25890600
H	-19.32780600	-6.19197000	-3.25780600
H	-19.17533100	-9.90338200	-5.46700100
H	-20.08692300	-8.55766800	-3.57379300
C	-14.68264200	-7.01552800	-6.82726500
C	-14.49270300	-7.95180600	-5.79676400
C	-13.57096000	-6.66225200	-7.61790700
C	-13.23679900	-8.52906300	-5.57685300
H	-15.33112400	-8.23728600	-5.15850000
C	-12.32137400	-7.24864000	-7.40728600
H	-13.67987700	-5.91105300	-8.40654200
C	-12.14868100	-8.18602300	-6.38292300
H	-13.11246100	-9.25618800	-4.76952900
H	-11.47601800	-6.96518800	-8.04034400
H	-11.16978700	-8.64150200	-6.21196600
C	-17.42761800	-4.03627800	-10.76105700
C	-17.12679000	-2.66227500	-10.72133500
C	-16.87232500	-4.81131400	-11.79483200
C	-16.30980300	-2.07711300	-11.69310200
H	-17.53677000	-2.04640300	-9.91569700
C	-16.04489300	-4.22976600	-12.76077600
H	-17.08772500	-5.88051500	-11.85863400
C	-15.76380800	-2.86080800	-12.71394600
H	-16.09273600	-1.00669900	-11.64643100
H	-15.62050100	-4.84927900	-13.55559300
H	-15.11767800	-2.40700500	-13.47005800
C	-20.17967900	-4.80257800	-10.22231700
C	-20.43275900	-4.51512800	-11.57386000
C	-21.26611900	-5.15368800	-9.39757100
C	-21.73223700	-4.58881700	-12.08739600
H	-19.61146200	-4.22901800	-12.23405400
C	-22.56075100	-5.24145500	-9.91405600
H	-21.09728800	-5.35372800	-8.33462200
C	-22.79868000	-4.95620900	-11.26275400
H	-21.90968500	-4.35800500	-13.14145900
H	-23.38893800	-5.52122100	-9.25737000
H	-23.81284000	-5.01346900	-11.66661800
Cl	-17.50549700	-2.79676500	-3.44904900
C	-13.93797200	-2.39472300	-4.15094500

C	-14.44771100	-1.37003300	-4.90704900
H	-13.95145200	-0.39858100	-4.82196200
C	-15.57726300	-1.42936100	-5.85149900
H	-15.42245900	-0.78036300	-6.71689000
H	-15.87977000	-2.43645100	-6.14975900
C	-15.00678500	-0.91648300	1.20375000
H	-15.23535200	-1.98864100	1.19103300
H	-13.94495900	-0.76616200	1.44863800
H	-15.63093000	-0.41675200	1.95875200
C	-14.41132100	-3.81539500	-4.19707800
H	-15.00538100	-4.04618000	-5.08994400
H	-13.55049500	-4.50249800	-4.15718300
H	-15.05595900	-4.01980400	-3.32503100
C	-12.81396100	-2.12208300	-3.19081600
H	-13.12215900	-2.40533800	-2.16842700
H	-11.93289900	-2.74116100	-3.43389900
H	-12.51015600	-1.06531500	-3.18479600

UB3LYP-D3/def2-SVP-SMD(THF)//UB3LYP/def2-SVP(gas)
HF= -4404.1185192
UPBEPBE-D3/def2-SVP-SMD(THF)//UB3LYP/def2-SVP(gas)
HF= -4400.7676457

^{3}D'-complex-TS-dimethyl

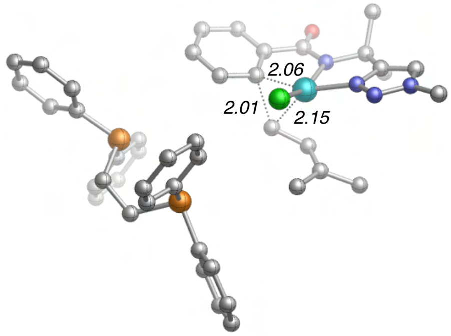

Zero-point correction= 0.807728 (Hartree/Particle)
Thermal correction to Energy= 0.861010
Thermal correction to Enthalpy= 0.861954
Thermal correction to Gibbs Free Energy= 0.707796
Sum of electronic and zero-point Energies= -4403.164506
Sum of electronic and thermal Energies= -4403.111224
Sum of electronic and thermal Enthalpies= -4403.110280
Sum of electronic and thermal Free Energies= -4403.264438

C	-17.44811300	-0.66980300	-5.79063500
C	-17.57007600	0.70843400	-6.07234700
C	-18.50670100	1.17021400	-6.99983400
C	-19.34881900	0.26314500	-7.64989300
C	-19.25335100	-1.10246100	-7.35430300
C	-18.30321900	-1.57095000	-6.43925900
H	-18.57043000	2.24752100	-7.17312200
H	-20.08547200	0.61779500	-8.37555000
H	-19.91509700	-1.81992500	-7.84649800
H	-18.22759100	-2.64086000	-6.23996000
C	-16.78183200	1.67185600	-5.23845400
O	-16.68235300	2.86894400	-5.50594400
C	-15.79321400	1.84322700	-2.99046300
C	-16.82202500	2.90538600	-2.52849300
H	-16.45603900	3.43357600	-1.63300400
H	-16.98907600	3.63303500	-3.33193400
H	-17.78059700	2.42364400	-2.28217800
C	-14.44462500	2.52149200	-3.32189500
H	-14.59211700	3.20381000	-4.16965100
H	-14.06498900	3.09729700	-2.46149100
H	-13.68625400	1.77252500	-3.59732400
C	-15.62514200	0.83640800	-1.87744300
N	-15.96657600	-0.46554300	-2.10685900
N	-15.79059100	-1.19606300	-1.04098800
C	-15.21290600	0.90038800	-0.56118500
H	-14.86415600	1.71825500	0.06278800
N	-15.33636400	-0.37712500	-0.10019500
N	-16.29645000	1.04386600	-4.12480300
Fe	-16.48169000	-0.86934900	-3.98743900
P	-16.29019200	-6.13261300	-7.13672600
P	-18.51502800	-4.68707000	-9.40070300
C	-16.69765600	-6.86413000	-8.82683800

H	-16.56815100	-7.95919000	-8.80643100
H	-15.91913300	-6.47632500	-9.50434300
C	-18.09120800	-6.51991300	-9.36777800
H	-18.86801700	-6.97216300	-8.72946900
H	-18.22629400	-6.96405900	-10.36853200
C	-17.49233900	-7.00288100	-6.02783000
C	-18.01160800	-6.26770700	-4.94763900
C	-17.92888800	-8.32814900	-6.21339600
C	-18.93535100	-6.84598700	-4.06969800
H	-17.70056400	-5.23197600	-4.78238600
C	-18.85657700	-8.90259600	-5.34056100
H	-17.53905000	-8.92487900	-7.04220100
C	-19.36105600	-8.16195500	-4.26508500
H	-19.32259200	-6.25352500	-3.23688200
H	-19.18669700	-9.93289500	-5.49953600
H	-20.08779200	-8.61254600	-3.58361000
C	-14.68837500	-7.02244900	-6.82351400
C	-14.49876400	-7.98118100	-5.81394500
C	-13.57469400	-6.64429800	-7.59959100
C	-13.24072500	-8.55616700	-5.60053300
H	-15.33896900	-8.28579900	-5.18702400
C	-12.32289600	-7.22845200	-7.39595000
H	-13.68437800	-5.87514300	-8.37066700
C	-12.15042500	-8.18841500	-6.39264300
H	-13.11631800	-9.30092200	-4.80945500
H	-11.47570000	-6.92547800	-8.01739200
H	-11.16981700	-8.64211900	-6.22682600
C	-17.42785700	-4.03122300	-10.75230500
C	-17.12724800	-2.65723900	-10.71035800
C	-16.87110900	-4.80472800	-11.78643500
C	-16.30913600	-2.07058100	-11.68026800
H	-17.53843000	-2.04256500	-9.90442900
C	-16.04255800	-4.22171400	-12.75053600
H	-17.08611700	-5.87389400	-11.85196900
C	-15.76173300	-2.85279100	-12.70149900
H	-16.09226500	-1.00020400	-11.63184700
H	-15.61703600	-4.84010400	-13.54561900
H	-15.11468600	-2.39786400	-13.45614500
C	-20.18093300	-4.79610800	-10.21764700
C	-20.43252200	-4.50472500	-11.56862800
C	-21.26852100	-5.14830200	-9.39485600
C	-21.73164500	-4.57548300	-12.08348800
H	-19.61030800	-4.21783500	-12.22734100
C	-22.56281600	-5.23312700	-9.91268500
H	-21.10086000	-5.35163600	-8.33234300
C	-22.79925100	-4.94384100	-11.26078600
H	-21.90789800	-4.34163100	-13.13708100
H	-23.39188400	-5.51381800	-9.25750400
H	-23.81312500	-4.99879300	-11.66568600
Cl	-17.49686200	-2.82873400	-3.44549400
C	-13.92173500	-2.37104500	-4.16690900

C	-14.44939200	-1.36705200	-4.93039500
H	-13.97142200	-0.38543500	-4.85901800
C	-15.59803900	-1.44699600	-5.85570000
H	-15.44612400	-0.82282000	-6.73976000
H	-15.90001600	-2.46171000	-6.12773400
C	-15.05000400	-0.89024600	1.22861000
H	-15.29026400	-1.95991400	1.22590200
H	-13.98701400	-0.74916600	1.47368900
H	-15.66985400	-0.37618300	1.97757200
C	-14.37797900	-3.79924000	-4.18623500
H	-14.99896200	-4.04534800	-5.05623100
H	-13.50590700	-4.47346800	-4.17300000
H	-14.98675000	-4.00856500	-3.29039900
C	-12.78647200	-2.07369800	-3.22610500
H	-13.07565400	-2.34653100	-2.19530400
H	-11.90154500	-2.68548200	-3.47352800
H	-12.49460100	-1.01357600	-3.23842100

UB3LYP-D3/def2-SVP-SMD(THF)//UB3LYP/def2-SVP(gas)
HF= -4404.1582902
UPBEPBE-D3/def2-SVP-SMD(THF)//UB3LYP/def2-SVP(gas)
HF= -4400.8005252

[5]D'-complex-TS-dimethyl

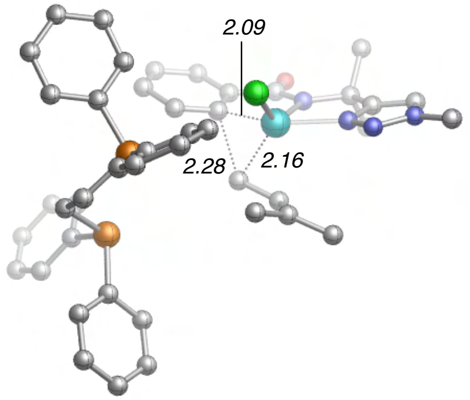

Zero-point correction= 0.806244 (Hartree/Particle)
Thermal correction to Energy= 0.860077
Thermal correction to Enthalpy= 0.861022
Thermal correction to Gibbs Free Energy= 0.705920
Sum of electronic and zero-point Energies= -4403.171948
Sum of electronic and thermal Energies= -4403.118115
Sum of electronic and thermal Enthalpies= -4403.117171
Sum of electronic and thermal Free Energies= -4403.272273

C	-18.38668300	-1.56262200	-4.96729500
C	-19.16912100	-0.48203500	-4.54224200
C	-20.56173400	-0.53731100	-4.68279400
C	-21.16758800	-1.67613500	-5.21767000
C	-20.37826500	-2.76527200	-5.60691900
C	-18.98150000	-2.70690400	-5.49814200
H	-21.14168100	0.32263900	-4.33801100
H	-22.25499900	-1.72333900	-5.31945200
H	-20.84597900	-3.67109000	-6.00270100
H	-18.37366300	-3.55371000	-5.82420800
C	-18.52280300	0.67670500	-3.83247600
O	-19.14765500	1.71096600	-3.58987800
C	-16.52096100	1.33815100	-2.60280900
C	-17.26330600	1.57379100	-1.26464100
H	-16.67879100	2.23447000	-0.60323200
H	-18.23660700	2.03908000	-1.46253400
H	-17.42507000	0.61563300	-0.74754300
C	-16.26847100	2.68782600	-3.31882200
H	-17.23326300	3.15380800	-3.55697300
H	-15.68454300	3.37171200	-2.68084200
H	-15.71043900	2.52766700	-4.25471900
C	-15.19275500	0.67868300	-2.30287400
N	-14.85606400	-0.48937500	-2.90970600
N	-13.68608100	-0.90470100	-2.51290500
C	-14.13929600	1.00245700	-1.46636700
H	-13.96669000	1.82904600	-0.78320200
N	-13.24192000	-0.00766600	-1.63783800
N	-17.24013900	0.38884500	-3.46542600
Fe	-16.45921000	-1.35002600	-4.18217800

P	-16.85133400	-6.24721000	-6.62401800
P	-17.52150100	-4.22192200	-9.50778200
C	-17.01201000	-6.83672800	-8.39321200
H	-17.28598800	-7.90355900	-8.44492200
H	-15.99897700	-6.76200500	-8.82265400
C	-18.01309200	-6.01363600	-9.22249300
H	-18.98779000	-5.96199200	-8.70927500
H	-18.21185700	-6.50775000	-10.18860800
C	-18.36770300	-6.94874900	-5.80122200
C	-18.70947600	-6.40379500	-4.54867700
C	-19.18549300	-7.95685300	-6.33917600
C	-19.82630700	-6.86922500	-3.84876600
H	-18.09845800	-5.60482300	-4.11772400
C	-20.31041500	-8.41209000	-5.64391500
H	-18.95204400	-8.40434100	-7.30807100
C	-20.63221300	-7.87246500	-4.39530100
H	-20.07177500	-6.43540200	-2.87590700
H	-20.93580100	-9.19587800	-6.08029200
H	-21.51108100	-8.23009400	-3.85231700
C	-15.53083500	-7.37399500	-5.95578100
C	-14.85621600	-6.93788200	-4.80018600
C	-15.16227600	-8.60520300	-6.52448400
C	-13.85254000	-7.72079100	-4.22207400
H	-15.12124100	-5.97645300	-4.34977000
C	-14.14867100	-9.38061600	-5.95300300
H	-15.66761800	-8.97697700	-7.41859000
C	-13.49334500	-8.94272300	-4.79833200
H	-13.34502900	-7.36844200	-3.32003300
H	-13.87402800	-10.33528100	-6.41026600
H	-12.70377100	-9.55218100	-4.35062900
C	-16.22145400	-4.34391200	-10.82715200
C	-15.48680200	-3.17200700	-11.09148400
C	-15.89453900	-5.50666900	-11.54576700
C	-14.47841000	-3.15481700	-12.05717100
H	-15.71154400	-2.26076100	-10.52899400
C	-14.87389400	-5.49567200	-12.50353100
H	-16.43744700	-6.43740900	-11.36853600
C	-14.16616000	-4.32011900	-12.76553700
H	-13.92749200	-2.23064000	-12.25137700
H	-14.63443500	-6.41243800	-13.04916500
H	-13.37053500	-4.31245700	-13.51501800
C	-19.00877600	-3.66035500	-10.47132000
C	-19.18584300	-3.86706600	-11.85016700
C	-20.01665500	-2.98966500	-9.75523300
C	-20.34562100	-3.42365000	-12.49259100
H	-18.41053000	-4.37329500	-12.43023400
C	-21.18059300	-2.55505300	-10.39632400
H	-19.88775300	-2.80025100	-8.68547700
C	-21.34696300	-2.76969200	-11.76755900
H	-20.46704200	-3.58953600	-13.56658600
H	-21.95356300	-2.03705900	-9.82251300

H	-22.25234900	-2.42243900	-12.27231500
Cl	-16.15351900	-3.45753000	-3.40049300
C	-14.10869600	-1.61263400	-6.08529300
C	-15.21283000	-0.79695200	-6.11960700
H	-15.04007000	0.27375500	-5.96233900
C	-16.59857300	-1.19908700	-6.33239900
H	-17.24269300	-0.41484100	-6.73455800
H	-16.76470000	-2.17166600	-6.80223000
C	-11.95486600	-0.19488100	-0.99132100
H	-11.27201300	0.62935900	-1.24525700
H	-12.07963200	-0.24302300	0.10026800
H	-11.53987700	-1.14294500	-1.35317700
C	-14.14046200	-3.09362400	-6.31209300
H	-15.13235400	-3.49044700	-6.55833300
H	-13.44075700	-3.35985900	-7.12365000
H	-13.79559100	-3.61741600	-5.40486400
C	-12.75124600	-1.02711000	-5.82269100
H	-12.34307500	-1.43963300	-4.88371900
H	-12.04639600	-1.30059700	-6.62711200
H	-12.77746300	0.06852300	-5.73457000

UB3LYP-D3/def2-SVP-SMD(THF)//UB3LYP/def2-SVP(gas)
HF= -4404.1661902
UPBEPBE-D3/def2-SVP-SMD(THF)//UB3LYP/def2-SVP(gas)
HF= -4400.7948116

^{1}D’-TS-noligand-dimethyl

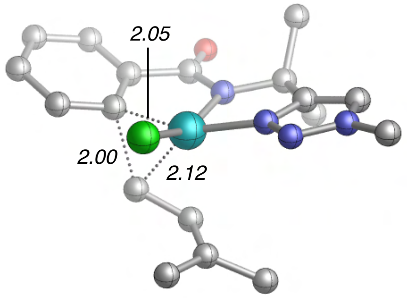

Zero-point correction= 0.384935 (Hartree/Particle)
Thermal correction to Energy= 0.410488
Thermal correction to Enthalpy= 0.411432
Thermal correction to Gibbs Free Energy= 0.330002
Sum of electronic and zero-point Energies= -2716.538603
Sum of electronic and thermal Energies= -2716.513051
Sum of electronic and thermal Enthalpies= -2716.512107
Sum of electronic and thermal Free Energies= -2716.593537

C	-18.35290600	-1.32442100	-4.87954500
C	-18.98695000	-0.07853500	-4.69385200
C	-20.37665300	0.03630900	-4.77709900
C	-21.15557300	-1.09647100	-5.03042300
C	-20.53448200	-2.34219100	-5.17944700
C	-19.14118200	-2.45785500	-5.11561300
H	-20.82230400	1.02073200	-4.61285200
H	-22.24349800	-1.01337500	-5.09585300
H	-21.13880900	-3.23731500	-5.35173800
H	-18.66309900	-3.43235900	-5.23283500
C	-18.14656400	1.07751600	-4.24236900
O	-18.55798900	2.23620600	-4.20799400
C	-16.08051000	1.51511900	-2.97638200
C	-16.84910600	2.10591100	-1.76850200
H	-16.17050700	2.69300100	-1.12847600
H	-17.65708800	2.75581300	-2.12562100
H	-17.28629900	1.29662900	-1.16396900
C	-15.46157400	2.65332300	-3.81914800
H	-16.27198700	3.27028200	-4.22984700
H	-14.80292800	3.29053800	-3.20599900
H	-14.86879300	2.24569200	-4.65250300
C	-15.00364500	0.59691200	-2.44875700
N	-15.03918300	-0.72430500	-2.79048700
N	-14.06018600	-1.38347000	-2.23579900
C	-13.91556600	0.74951200	-1.61243600
H	-13.50245900	1.60737100	-1.08963400
N	-13.37637800	-0.49956800	-1.51937600
N	-16.93238200	0.63004400	-3.79501800
Fe	-16.46262700	-1.21158900	-4.08219700
Cl	-16.37780300	-3.41696300	-3.59772200
C	-14.29202300	-1.72531600	-6.01102900
C	-15.40761600	-0.93123200	-6.05875400
H	-15.24390600	0.15031300	-6.03948600

C	-16.81653000	-1.35904500	-6.16465000
H	-17.40088000	-0.66993800	-6.77977000
H	-16.95716600	-2.39492600	-6.48119500
C	-12.21832900	-0.92947600	-0.75423100
H	-12.09734400	-2.00644400	-0.91964500
H	-11.31660700	-0.39791000	-1.09204200
H	-12.37669200	-0.73811000	0.31710500
C	-14.28749000	-3.21949200	-6.13313800
H	-15.27703700	-3.64796700	-6.32340800
H	-13.59552800	-3.51978700	-6.93960900
H	-13.92610600	-3.67206000	-5.19506200
C	-12.93656500	-1.09607800	-5.84408800
H	-12.46990700	-1.46104400	-4.91152500
H	-12.26124500	-1.38902300	-6.66663000
H	-12.98388200	0.00169100	-5.80363800

UB3LYP-D3/def2-SVP-SMD(THF)//UB3LYP/def2-SVP(gas)
HF= -2716.9972074
UPBEPBE-D3/def2-SVP-SMD(THF)//UB3LYP/def2-SVP(gas)
HF= -2715.3197293

^{3}D'-TS-noligand-dimethyl

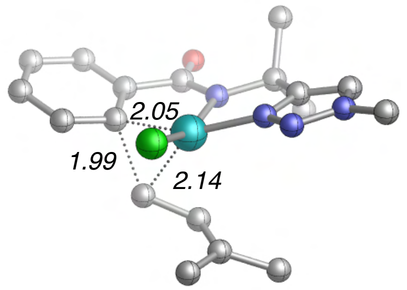

Zero-point correction= 0.384828 (Hartree/Particle)
Thermal correction to Energy= 0.410445
Thermal correction to Enthalpy= 0.411389
Thermal correction to Gibbs Free Energy= 0.328709
Sum of electronic and zero-point Energies= -2716.553979
Sum of electronic and thermal Energies= -2716.528361
Sum of electronic and thermal Enthalpies= -2716.527417
Sum of electronic and thermal Free Energies= -2716.610097

C	-18.35704300	-1.33374400	-4.87315500
C	-18.98114600	-0.07972200	-4.69850500
C	-20.36924200	0.04595100	-4.78463000
C	-21.15774600	-1.08305800	-5.02670200
C	-20.54806100	-2.33643800	-5.16094300
C	-19.15637400	-2.46392400	-5.09553100
H	-20.80675000	1.03570900	-4.63087300
H	-22.24484800	-0.99104100	-5.09391900
H	-21.16074600	-3.22783800	-5.32251400
H	-18.68623300	-3.44349300	-5.20239000
C	-18.13153800	1.07321000	-4.25403700
O	-18.53278700	2.23600700	-4.23442600
C	-16.06716600	1.50193700	-2.98135500
C	-16.83629000	2.11574100	-1.78505400
H	-16.15531400	2.70359300	-1.14827800
H	-17.63547500	2.76928900	-2.15516400
H	-17.28545000	1.31857400	-1.17321000
C	-15.42965200	2.62346600	-3.83238000
H	-16.23060200	3.24350500	-4.25687400
H	-14.76974500	3.26174800	-3.22168200
H	-14.83420200	2.20007500	-4.65585300
C	-15.00427900	0.57814400	-2.43453800
N	-15.05722000	-0.75009900	-2.74723700
N	-14.08806000	-1.41024200	-2.17639900
C	-13.91508500	0.73389500	-1.60027000
H	-13.49027100	1.59745300	-1.09658700
N	-13.39334600	-0.52022000	-1.47874600
N	-16.92596300	0.61868800	-3.79483200
Fe	-16.47689700	-1.23879700	-4.05070800
Cl	-16.36562000	-3.45329400	-3.61295600
C	-14.29850700	-1.69387500	-6.03274700
C	-15.43137400	-0.93212200	-6.07900300
H	-15.29777600	0.15359100	-6.06505600

C	-16.83474200	-1.39425300	-6.15714300
H	-17.43158700	-0.73486200	-6.79255600
H	-16.95366100	-2.44061300	-6.44740800
C	-12.24216100	-0.94900600	-0.70259500
H	-12.13005700	-2.02928600	-0.85188100
H	-11.33469400	-0.43035600	-1.04492300
H	-12.40273200	-0.74035600	0.36520900
C	-14.25400700	-3.18967400	-6.13847900
H	-15.23764600	-3.65052100	-6.27506900
H	-13.59306600	-3.47758400	-6.97525000
H	-13.83075600	-3.62118200	-5.21667600
C	-12.95628800	-1.02736600	-5.89784400
H	-12.45918100	-1.37111600	-4.97302400
H	-12.29045600	-1.30895600	-6.73209100
H	-13.03204000	0.06911000	-5.86599900

UB3LYP-D3/def2-SVP-SMD(THF)//UB3LYP/def2-SVP(gas)
HF= -2717.0377497
UPBEPBE-D3/def2-SVP-SMD(THF)//UB3LYP/def2-SVP(gas)
HF= -2715.3535706

^{5}D'-TS-noligand-dimethyl

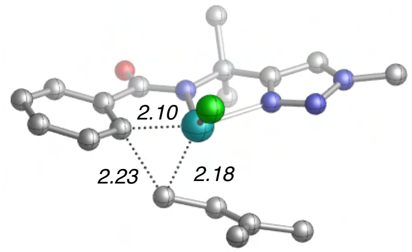

Zero-point correction= 0.383057 (Hartree/Particle)
Thermal correction to Energy= 0.409304
Thermal correction to Enthalpy= 0.410248
Thermal correction to Gibbs Free Energy= 0.324854
Sum of electronic and zero-point Energies= -2716.562332
Sum of electronic and thermal Energies= -2716.536084
Sum of electronic and thermal Enthalpies= -2716.535140
Sum of electronic and thermal Free Energies= -2716.620534

C	-18.68150900	-1.26997000	-4.90616600
C	-19.24944600	-0.03337900	-4.57470300
C	-20.63318900	0.14651100	-4.69558200
C	-21.44458000	-0.90961100	-5.11581800
C	-20.87182200	-2.15455300	-5.40250000
C	-19.48609200	-2.33654300	-5.30880800
H	-21.04363000	1.12332800	-4.42711600
H	-22.52482200	-0.76990400	-5.20667900
H	-21.50395200	-2.99298700	-5.70843900
H	-19.04485800	-3.31058500	-5.53391600
C	-18.39175100	1.04680100	-3.97138600
O	-18.81082600	2.19737700	-3.83276400
C	-16.28908700	1.43140300	-2.79125100
C	-16.95863100	1.92519000	-1.48568600
H	-16.25306400	2.52234800	-0.88474700
H	-17.82904700	2.54534600	-1.73258400
H	-17.29220700	1.06686700	-0.88272300
C	-15.79625200	2.63790500	-3.62767000
H	-16.65882600	3.25411600	-3.91190200
H	-15.08547900	3.25463300	-3.05318000
H	-15.29124100	2.29104200	-4.54292300
C	-15.10418200	0.56413300	-2.42448200
N	-15.00384600	-0.70012800	-2.91299100
N	-13.92536300	-1.28879200	-2.47747100
C	-13.99349200	0.76204200	-1.62366900
H	-13.65550300	1.60327200	-1.02542600
N	-13.30507800	-0.41124400	-1.69503800
N	-17.18362600	0.55842300	-3.56593900
Fe	-16.74515900	-1.35574200	-4.10802800
Cl	-16.81906900	-3.44727800	-3.27962700
C	-14.58825600	-2.25958300	-6.04490100
C	-15.47507400	-1.21288300	-6.09325300
H	-15.05971300	-0.20677700	-5.96822800
C	-16.92251600	-1.29244300	-6.27686600

H	-17.37550700	-0.39858400	-6.71072800
H	-17.30233700	-2.21675400	-6.71715000
C	-12.06307400	-0.77345800	-1.03453300
H	-11.84071100	-1.81374000	-1.29969800
H	-11.24330100	-0.12182100	-1.37131700
H	-12.17261200	-0.68903100	0.05644100
C	-14.97279400	-3.69832400	-6.22664600
H	-15.94297900	-3.82542500	-6.72562300
H	-14.20353200	-4.23266200	-6.80714100
H	-15.05682200	-4.18668000	-5.23980500
C	-13.13244600	-2.00257900	-5.78448900
H	-12.82630600	-2.50519000	-4.85038300
H	-12.51022500	-2.42188100	-6.59433800
H	-12.90812100	-0.93098300	-5.68588500

UB3LYP-D3/def2-SVP-SMD(THF)//UB3LYP/def2-SVP(gas)
HF= -2717.0443573
UPBEPBE-D3/def2-SVP-SMD(THF)//UB3LYP/def2-SVP(gas)
HF= -2715.3467163

^{1}D''-TS-dimethyl

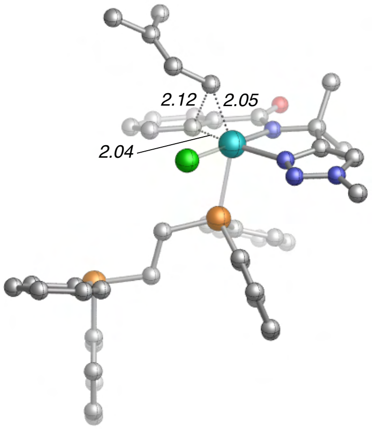

Zero-point correction= 0.808540 (Hartree/Particle)
Thermal correction to Energy= 0.861090
Thermal correction to Enthalpy= 0.862034
Thermal correction to Gibbs Free Energy= 0.715981
Sum of electronic and zero-point Energies= -4403.149124
Sum of electronic and thermal Energies= -4403.096574
Sum of electronic and thermal Enthalpies= -4403.095630
Sum of electronic and thermal Free Energies= -4403.241683

C	-18.33404400	0.95960900	-3.42607300
C	-19.13524600	2.07721400	-3.12389200
C	-20.51281100	2.05849100	-3.36208600
C	-21.10395600	0.92049800	-3.91847200
C	-20.31100800	-0.19557600	-4.22338300
C	-18.93515900	-0.18646200	-3.96064200
H	-21.09313100	2.94877300	-3.10681900
H	-22.18023200	0.89549700	-4.10913800
H	-20.77026100	-1.08872700	-4.65737000
H	-18.32659700	-1.07082100	-4.16292000
C	-18.44608100	3.27756300	-2.54295100
O	-19.06500700	4.22093800	-2.04301200
C	-16.20589600	4.19057400	-2.09004800
C	-16.29136600	4.20235100	-0.54245600
H	-15.60340000	4.94749700	-0.11019100
H	-17.31876500	4.45488800	-0.24660000
H	-16.03421700	3.21457400	-0.12958100
C	-16.49459200	5.60924500	-2.63169800
H	-17.51255200	5.90584800	-2.35235700
H	-15.77540300	6.33343300	-2.21421200
H	-16.41355900	5.62927300	-3.72650100
C	-14.81885100	3.76701200	-2.51249700
N	-14.64594500	2.57518700	-3.15716900
N	-13.38469500	2.35642700	-3.40479000
C	-13.55997100	4.31593900	-2.36792700
H	-13.20640800	5.24185900	-1.92369900
N	-12.72049900	3.40271400	-2.93402800
N	-17.09241100	3.15371600	-2.64926500
Fe	-16.36130100	1.48946700	-3.34185100

P	-16.60316700	2.01674000	-5.85292100
C	-17.27563700	0.68578500	-8.34674000
H	-17.91742800	1.53366900	-8.63601000
H	-16.28460500	0.88828500	-8.78140200
C	-17.21042100	0.54320500	-6.82473600
H	-16.53244700	-0.26602700	-6.51235700
H	-18.19373900	0.27892600	-6.40800600
C	-15.04955700	2.44103200	-6.78158200
C	-14.99400000	3.38390100	-7.82459700
C	-13.88579500	1.71256600	-6.46995100
C	-13.80347800	3.60711400	-8.52343800
H	-15.88439500	3.94632100	-8.10844700
C	-12.70051700	1.93304400	-7.17887400
H	-13.91375200	0.96390000	-5.67506100
C	-12.65152100	2.88390600	-8.20228500
H	-13.78325600	4.34369500	-9.33113900
H	-11.80902400	1.35302100	-6.92465300
H	-11.72285400	3.05567100	-8.75331300
C	-17.78801300	3.36772900	-6.26852400
C	-19.12916200	3.08449300	-6.58045200
C	-17.40346700	4.71668800	-6.15725200
C	-20.04671300	4.11615000	-6.80136000
H	-19.47741300	2.05222600	-6.63880300
C	-18.31889300	5.74698100	-6.38055900
H	-16.37321300	4.96859200	-5.89800100
C	-19.64526900	5.45019400	-6.70774500
H	-21.08413500	3.86989600	-7.04121700
H	-17.99517700	6.78716300	-6.29070500
H	-20.36357400	6.25576100	-6.87935900
Cl	-15.30070900	-0.45321100	-4.06264700
C	-11.27987400	3.48070800	-3.10326200
H	-11.02277400	4.19523200	-3.89972800
H	-10.80830900	3.79376500	-2.16134500
H	-10.92416000	2.48164800	-3.38119900
P	-18.07734300	-0.79979700	-9.17594800
C	-18.02833900	-0.21053500	-10.93971800
C	-19.16559700	0.46531200	-11.41784300
C	-16.94229700	-0.39528700	-11.81215400
C	-19.20967400	0.96293700	-12.72309200
H	-20.02978900	0.59673300	-10.75935600
C	-16.99031600	0.09204600	-13.12187300
H	-16.05185500	-0.92682800	-11.46851100
C	-18.12109300	0.77505600	-13.58017800
H	-20.10140000	1.48870000	-13.07505500
H	-16.13726700	-0.06272800	-13.78830600
H	-18.15634600	1.15451200	-14.60481900
C	-16.74306000	-2.08547900	-9.15420600
C	-17.12549800	-3.39467800	-9.50344300
C	-15.40659900	-1.85943100	-8.78334900
C	-16.19914400	-4.43936300	-9.50552700
H	-18.16638000	-3.59572500	-9.77464900

C	-14.48086400	-2.90881300	-8.76925900
H	-15.06987400	-0.86075900	-8.49868100
C	-14.87182200	-4.19914900	-9.13456000
H	-16.51684800	-5.44707400	-9.78646100
H	-13.44834800	-2.71250200	-8.46862300
H	-14.14713500	-5.01746100	-9.12280900
C	-18.23536000	-1.60518200	-0.82983100
C	-17.25141700	-0.97942500	-1.52010200
H	-16.45418500	-1.58522700	-1.95675800
C	-17.13564500	0.46407500	-1.74379100
H	-16.06898500	0.78255800	-1.57699400
H	-17.78193300	1.07645000	-1.11325600
C	-18.19244300	-3.09808800	-0.63609600
H	-18.20134500	-3.35786600	0.43796000
H	-19.08759800	-3.57497800	-1.07501000
H	-17.30135500	-3.54983600	-1.09491800
C	-19.43303300	-0.92278500	-0.22734800
H	-19.44610200	0.16408400	-0.37657800
H	-20.35925500	-1.32736400	-0.67213600
H	-19.49370500	-1.12714500	0.85652700

UB3LYP-D3/def2-SVP-SMD(THF)//UB3LYP/def2-SVP(gas)
HF= -4404.1472856
UPBEPBE-D3/def2-SVP-SMD(THF)//UB3LYP/def2-SVP(gas)
HF= -4400.7972165

^{3}D''-TS-dimethyl

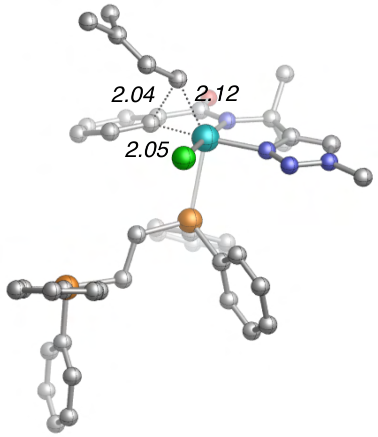

Zero-point correction= 0.807569 (Hartree/Particle)
Thermal correction to Energy= 0.860634
Thermal correction to Enthalpy= 0.861578
Thermal correction to Gibbs Free Energy= 0.711640
Sum of electronic and zero-point Energies= -4403.164156
Sum of electronic and thermal Energies= -4403.111091
Sum of electronic and thermal Enthalpies= -4403.110147
Sum of electronic and thermal Free Energies= -4403.260086

C	-18.22840200	0.80416600	-3.29099600
C	-19.08840200	1.89242900	-3.03266300
C	-20.43720900	1.84413100	-3.39399200
C	-20.94240800	0.70931400	-4.03602000
C	-20.09044500	-0.37085700	-4.30983000
C	-18.74547500	-0.33433300	-3.92529400
H	-21.06294800	2.71234400	-3.17239100
H	-21.99626300	0.66169900	-4.32272600
H	-20.47952300	-1.25627700	-4.82077700
H	-18.09338800	-1.18938000	-4.11397200
C	-18.48297600	3.11635100	-2.41138500
O	-19.16159700	4.02918900	-1.93945600
C	-16.30711300	4.19523800	-1.98483500
C	-16.41436000	4.31409900	-0.44535400
H	-15.77973400	5.13113100	-0.06433200
H	-17.45868400	4.52197500	-0.17613100
H	-16.10096800	3.37633600	0.03907800
C	-16.68736000	5.54030800	-2.64587400
H	-17.71912600	5.80069300	-2.38122800
H	-16.01102900	6.34153600	-2.30492400
H	-16.61773300	5.46248000	-3.73965000
C	-14.89053100	3.83939300	-2.37015900
N	-14.64378000	2.65786200	-3.00887200
N	-13.37000400	2.50962000	-3.24380200
C	-13.66605700	4.45844100	-2.21314600
H	-13.36967800	5.40383900	-1.76807200
N	-12.77064900	3.59328700	-2.76893600
N	-17.11858000	3.06516800	-2.47824700

Fe	-16.29805000	1.48275300	-3.20896200
P	-16.51118700	2.13999800	-5.94033600
C	-17.28477000	0.74958600	-8.38433400
H	-17.90401300	1.61157100	-8.68224500
H	-16.29862500	0.90332400	-8.84974100
C	-17.18391700	0.65883100	-6.86062300
H	-16.52369200	-0.16158100	-6.53703400
H	-18.16353700	0.43531600	-6.41089400
C	-14.95462700	2.49024500	-6.89216800
C	-14.90171800	3.30338600	-8.03983500
C	-13.77714400	1.84563900	-6.46858600
C	-13.70213700	3.47989300	-8.73567600
H	-15.80251400	3.80158100	-8.40221600
C	-12.58145300	2.01739000	-7.17440100
H	-13.80170700	1.19450300	-5.59197800
C	-12.53702300	2.83824700	-8.30463200
H	-13.68270600	4.11505300	-9.62544000
H	-11.67889200	1.50248800	-6.83370700
H	-11.60052700	2.97345500	-8.85261100
C	-17.65238700	3.52015000	-6.39020100
C	-19.02259600	3.28213800	-6.60208500
C	-17.20633300	4.85617500	-6.38413100
C	-19.90941700	4.34009800	-6.82396900
H	-19.41602700	2.26417200	-6.58423300
C	-18.09225800	5.91220500	-6.60970000
H	-16.15112600	5.07748700	-6.20755100
C	-19.44885700	5.65852500	-6.83332800
H	-20.96926000	4.12753900	-6.98606100
H	-17.71986000	6.94010300	-6.60579600
H	-20.14302900	6.48454100	-7.00692600
Cl	-15.16641100	-0.36612400	-3.98909800
C	-11.33329000	3.74750600	-2.91258600
H	-11.10139300	4.52798300	-3.65268100
H	-10.88373500	4.01267700	-1.94522000
H	-10.93135400	2.78724500	-3.25627500
P	-18.16150000	-0.72918900	-9.14613400
C	-18.11981000	-0.20714900	-10.93139800
C	-19.25964000	0.44590000	-11.43377200
C	-17.03203800	-0.41749300	-11.79600100
C	-19.30538000	0.89529100	-12.75663100
H	-20.12426400	0.59749000	-10.78035100
C	-17.08104100	0.02208800	-13.12226700
H	-16.13979900	-0.93265900	-11.43248900
C	-18.21529400	0.68217100	-13.60556400
H	-20.19945000	1.40349100	-13.12794100
H	-16.22678800	-0.15229800	-13.78228700
H	-18.25169900	1.02411900	-14.64329800
C	-16.88087100	-2.06849600	-9.10215000
C	-17.32131400	-3.36932600	-9.41251100
C	-15.53263700	-1.88945100	-8.74859700
C	-16.43995700	-4.45194600	-9.39406000

H	-18.37225600	-3.53303200	-9.66965500
C	-14.65169200	-2.97642600	-8.71408300
H	-15.15185700	-0.89861800	-8.49331100
C	-15.10016800	-4.25835400	-9.04080800
H	-16.80248400	-5.45247100	-9.64487700
H	-13.60886600	-2.81603900	-8.42804900
H	-14.41049800	-5.10601700	-9.01300900
C	-18.16809400	-1.84857400	-0.86369500
C	-17.20168800	-1.14714100	-1.49799500
H	-16.36466000	-1.69328300	-1.93992300
C	-17.13608100	0.31847800	-1.64357400
H	-16.09969900	0.64753900	-1.38911400
H	-17.82092600	0.86721600	-0.99508500
C	-18.06081400	-3.34618200	-0.73645300
H	-18.07557600	-3.65572100	0.32425000
H	-18.92476400	-3.84277300	-1.21438700
H	-17.14163700	-3.73562600	-1.19733400
C	-19.40882700	-1.24959200	-0.25717200
H	-19.48101400	-1.50269700	0.81569800
H	-19.46921900	-0.15849600	-0.35834700
H	-20.30715300	-1.67558500	-0.73794000

UB3LYP-D3/def2-SVP-SMD(THF)//UB3LYP/def2-SVP(gas)
HF= -4404.1680645
UPBEPBE-D3/def2-SVP-SMD(THF)//UB3LYP/def2-SVP(gas)
HF= -4400.8098175

^{5}D''-TS-dimethyl

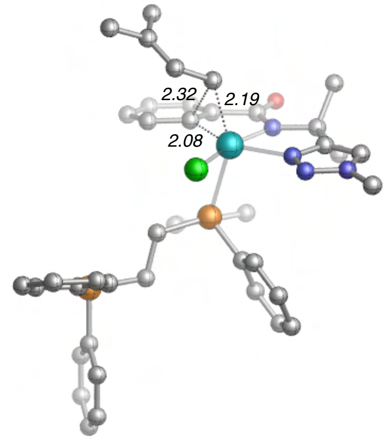

Zero-point correction= 0.806337 (Hartree/Particle)
Thermal correction to Energy= 0.859949
Thermal correction to Enthalpy= 0.860893
Thermal correction to Gibbs Free Energy= 0.707768
Sum of electronic and zero-point Energies= -4403.172367
Sum of electronic and thermal Energies= -4403.118755
Sum of electronic and thermal Enthalpies= -4403.117811
Sum of electronic and thermal Free Energies= -4403.270936

C	-18.45479900	1.15734100	-3.25601200
C	-19.05046500	2.38022200	-2.91403000
C	-20.43608500	2.53756800	-3.04731900
C	-21.21574700	1.49114300	-3.54599200
C	-20.60928600	0.28042800	-3.90578400
C	-19.22763100	0.10316000	-3.74411900
H	-20.86962700	3.49878100	-2.76050800
H	-22.29610600	1.61572200	-3.65677400
H	-21.21547300	-0.53967500	-4.30214700
H	-18.76355000	-0.85665800	-3.98639100
C	-18.18933000	3.51745200	-2.42605400
O	-18.69538300	4.53935700	-1.95030700
C	-15.88071400	4.22643800	-2.07857800
C	-16.00555700	4.39818100	-0.54322600
H	-15.22583900	5.07448300	-0.15582400
H	-16.99218500	4.81624300	-0.30572200
H	-15.90040000	3.42473800	-0.03962600
C	-15.98173500	5.60871000	-2.76871500
H	-16.98807400	6.01705800	-2.61271400
H	-15.24055700	6.30979700	-2.35058500
H	-15.78684500	5.51688300	-3.84801800
C	-14.52756100	3.62612600	-2.39205200
N	-14.43628500	2.40674900	-2.98731700
N	-13.19009000	2.06426400	-3.16448500
C	-13.23041500	4.06249200	-2.19252100
H	-12.81400000	4.96643900	-1.75744000
N	-12.45241600	3.05949100	-2.68778600
N	-16.86197000	3.26067300	-2.59016100

Fe	-16.39287000	1.40946600	-3.43191700
P	-16.91063800	2.03889800	-5.98134100
C	-17.21210900	0.59945100	-8.48678400
H	-17.75657700	1.47541400	-8.87520600
H	-16.16475300	0.73073900	-8.80132000
C	-17.32364900	0.50765600	-6.96178500
H	-16.65292800	-0.26070800	-6.54905300
H	-18.33725500	0.21393500	-6.64298100
C	-15.44815500	2.74364900	-6.87080300
C	-15.53622800	3.79863100	-7.79503500
C	-14.18862200	2.16986000	-6.61012500
C	-14.39113300	4.27041700	-8.44464800
H	-16.50251900	4.25682500	-8.01357400
C	-13.04967400	2.63936200	-7.27028300
H	-14.10201800	1.35109000	-5.89072300
C	-13.14552600	3.69180700	-8.18630700
H	-14.47729300	5.09119900	-9.16167800
H	-12.08016400	2.17867000	-7.06211800
H	-12.25251300	4.05948300	-8.69880100
C	-18.28590300	3.21547800	-6.32231200
C	-19.47066000	2.83954000	-6.97878900
C	-18.19066400	4.52403300	-5.81287600
C	-20.51921700	3.75160000	-7.13570800
H	-19.59547400	1.82907900	-7.36961700
C	-19.23622700	5.43426800	-5.97281300
H	-17.29491500	4.83240400	-5.27225100
C	-20.40552900	5.05094100	-6.63702700
H	-21.43183000	3.43799900	-7.64909100
H	-19.14176100	6.44206300	-5.56129300
H	-21.22799600	5.76068400	-6.75707100
Cl	-15.28171900	-0.47296900	-4.28419300
C	-11.00338500	2.98541300	-2.75311400
H	-10.60800600	3.76847200	-3.41698600
H	-10.56988100	3.10304500	-1.74941900
H	-10.74345100	1.99826800	-3.15317800
P	-18.00843900	-0.85737400	-9.36857300
C	-17.67370000	-0.35505000	-11.12797800
C	-18.71249700	0.29283300	-11.81963600
C	-16.45693300	-0.57471000	-11.79620600
C	-18.53595100	0.72789100	-13.13649300
H	-19.67356700	0.45140500	-11.32103800
C	-16.28281400	-0.14881300	-13.11612600
H	-15.63930600	-1.08687900	-11.28337800
C	-17.31972800	0.50594100	-13.78876600
H	-19.35451400	1.23188400	-13.65743600
H	-15.33068200	-0.32975700	-13.62246900
H	-17.18164400	0.83697800	-14.82145800
C	-16.80029500	-2.24013600	-9.12143800
C	-17.22497400	-3.52261600	-9.51877000
C	-15.52919900	-2.11152500	-8.53657000
C	-16.39826300	-4.63612000	-9.35959700

H	-18.21990500	-3.64744000	-9.95689400
C	-14.70646700	-3.22997500	-8.36039400
H	-15.16454100	-1.13770900	-8.20475500
C	-15.13501500	-4.49286700	-8.77532000
H	-16.74577600	-5.62153300	-9.68137900
H	-13.72640600	-3.10864000	-7.89183600
H	-14.49098900	-5.36516100	-8.63659100
C	-18.33454300	-1.70428100	-1.00052700
C	-17.27532800	-1.07623700	-1.57182300
H	-16.51705200	-1.69412600	-2.06158900
C	-17.02603200	0.35897600	-1.61321200
H	-15.95277700	0.58647000	-1.47203300
H	-17.63391400	0.98531700	-0.95909000
C	-18.42494000	-3.20730300	-1.01267300
H	-18.47157200	-3.60933400	0.01579300
H	-19.35184400	-3.54060700	-1.51408600
H	-17.57002800	-3.66992700	-1.52658000
C	-19.47961300	-1.00357300	-0.32294500
H	-19.59760300	-1.36542100	0.71427900
H	-19.37521700	0.08835300	-0.29856800
H	-20.42666500	-1.23240100	-0.84276300

UB3LYP-D3/def2-SVP-SMD(THF)//UB3LYP/def2-SVP(gas)
HF= -4404.1790291
UPBEPBE-D3/def2-SVP-SMD(THF)//UB3LYP/def2-SVP(gas)
HF= -4400.8063315

[1]D’’-outer-TS-dimethyl

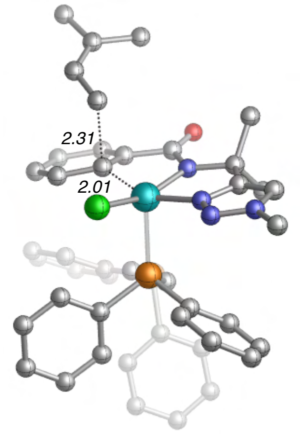

Zero-point correction= 0.805694 (Hartree/Particle)
Thermal correction to Energy= 0.858579
Thermal correction to Enthalpy= 0.859523
Thermal correction to Gibbs Free Energy= 0.712502
Sum of electronic and zero-point Energies= -4403.131454
Sum of electronic and thermal Energies= -4403.078569
Sum of electronic and thermal Enthalpies= -4403.077625
Sum of electronic and thermal Free Energies= -4403.224647

C	-18.99159500	1.36395500	-2.14510800
C	-18.89667200	2.72463600	-2.53927500
C	-19.85948300	3.32991900	-3.34558400
C	-20.93464300	2.56549000	-3.81700200
C	-21.02209500	1.20048900	-3.49862400
C	-20.06788100	0.60804900	-2.66885800
H	-19.74330000	4.38436600	-3.61013600
H	-21.69712500	3.02798400	-4.44983300
H	-21.85326600	0.60428600	-3.88719700
H	-20.16276100	-0.44380700	-2.38652600
C	-17.68215600	3.46132800	-2.06493700
O	-17.58838200	4.69357600	-2.09368700
C	-15.49231400	3.06020100	-1.02013600
C	-15.75481100	3.99393600	0.18724800
H	-14.80513200	4.29687300	0.65776600
H	-16.28952100	4.88953600	-0.15217900
H	-16.36493100	3.47785400	0.94458100
C	-14.60249000	3.78293000	-2.05785700
H	-15.14920700	4.64533500	-2.46207100
H	-13.66559800	4.13675900	-1.59706200
H	-14.33999300	3.10259400	-2.88166800
C	-14.80224500	1.81168800	-0.52361700
N	-15.40505400	0.60058500	-0.70600500
N	-14.69791400	-0.35856800	-0.17715300
C	-13.63135200	1.56409800	0.16447300
H	-12.83962100	2.21119100	0.53059000
N	-13.62336200	0.21328600	0.35065600
N	-16.74897900	2.57298200	-1.61845100
Fe	-17.15221200	0.66693700	-1.73546700

P	-16.17990600	0.07873000	-3.98090300
P	-17.11785000	3.31578200	-7.04670800
C	-16.02459600	1.47510700	-5.21016600
H	-15.27535900	1.19771100	-5.96840000
H	-15.62237800	2.33655200	-4.65377400
C	-17.34539100	1.85313800	-5.88885700
H	-18.08914200	2.15801800	-5.13652000
H	-17.75730900	0.99096700	-6.43629100
C	-16.90010000	-1.28484200	-5.00793300
C	-17.98204500	-2.03843700	-4.52983000
C	-16.36833800	-1.57870400	-6.27790500
C	-18.52867500	-3.06109300	-5.31509400
H	-18.36998000	-1.84943500	-3.52718600
C	-16.91920500	-2.59586400	-7.05853400
H	-15.51465000	-1.01684900	-6.66485400
C	-18.00468900	-3.33864800	-6.57870500
H	-19.36757100	-3.64534700	-4.92801000
H	-16.49698200	-2.81168900	-8.04350000
H	-18.43467000	-4.13687600	-7.18968900
C	-14.43940000	-0.51340700	-3.77298700
C	-13.31030900	0.18944000	-4.22461600
C	-14.25219700	-1.72368500	-3.07866500
C	-12.02345000	-0.31829400	-4.00893100
H	-13.41831900	1.13672600	-4.75605700
C	-12.96876000	-2.23271700	-2.87580300
H	-15.11849500	-2.25980900	-2.68241400
C	-11.84898200	-1.53341100	-3.34247400
H	-11.15600400	0.23786100	-4.37443000
H	-12.84329800	-3.18128400	-2.34696300
H	-10.84424500	-1.93614000	-3.18820500
C	-16.06107700	2.53426700	-8.36731400
C	-16.50813600	1.50603100	-9.21696200
C	-14.73990900	2.99213600	-8.50531300
C	-15.65350400	0.94770700	-10.17050000
H	-17.53655500	1.14387100	-9.13798500
C	-13.88086100	2.43124000	-9.45764200
H	-14.38429700	3.80230600	-7.86201800
C	-14.33672200	1.40782300	-10.29187900
H	-16.01717900	0.15164200	-10.82601500
H	-12.85614100	2.80076200	-9.55161900
H	-13.67035100	0.97151500	-11.04070700
C	-18.75742200	3.39160000	-7.90783400
C	-19.89177600	2.65242300	-7.53224200
C	-18.88395800	4.31685200	-8.96270100
C	-21.11047000	2.82528600	-8.20043700
H	-19.84242700	1.93596700	-6.71090900
C	-20.09534600	4.48103100	-9.63499000
H	-18.01874600	4.91625300	-9.26175500
C	-21.21667500	3.73459800	-9.25440000
H	-21.98058200	2.23898200	-7.89251900
H	-20.16748400	5.20026500	-10.45527900

H	-22.16850100	3.86599000	-9.77552500
Cl	-17.81654800	-1.42093400	-1.09159200
C	-21.16557600	3.55598900	0.21681900
C	-20.98848000	2.20584300	0.02151300
H	-21.88531700	1.61569500	-0.19802700
C	-19.75728700	1.50313900	0.03117000
H	-18.85102000	1.99763700	0.38564000
H	-19.74929400	0.41507200	0.10326300
C	-12.59743400	-0.60958700	0.96586700
H	-13.05539100	-1.56819000	1.23747700
H	-11.77164400	-0.78700400	0.26009800
H	-12.21354200	-0.11332100	1.86769100
C	-22.53535200	4.16616400	0.13419400
H	-22.55998000	4.96661700	-0.62805100
H	-23.30858000	3.42624800	-0.11921700
H	-22.81314700	4.64783300	1.08996300
C	-20.03694900	4.50894600	0.49125400
H	-19.17540500	4.02109600	0.96872800
H	-19.66049500	4.96044100	-0.44545600
H	-20.36789000	5.33575100	1.14102500

UB3LYP-D3/def2-SVP-SMD(THF)//UB3LYP/def2-SVP(gas)
HF= -4404.1258367
UPBEPBE-D3/def2-SVP-SMD(THF)//UB3LYP/def2-SVP(gas)
HF= -4400.7659135

^{3}D''-outer-TS-dimethyl

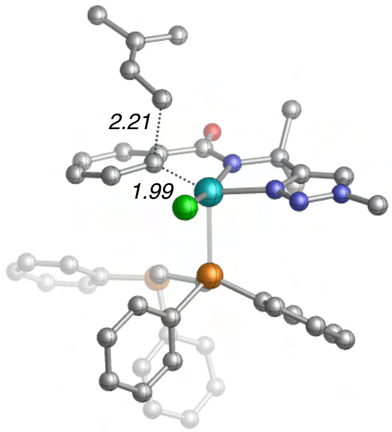

Zero-point correction= 0.805676 (Hartree/Particle)
Thermal correction to Energy= 0.858787
Thermal correction to Enthalpy= 0.859731
Thermal correction to Gibbs Free Energy= 0.708413
Sum of electronic and zero-point Energies= -4403.152569
Sum of electronic and thermal Energies= -4403.099458
Sum of electronic and thermal Enthalpies= -4403.098514
Sum of electronic and thermal Free Energies= -4403.249832

C	-19.10229400	1.42376200	-2.02944700
C	-18.95620500	2.76268200	-2.49400800
C	-19.90439500	3.36744900	-3.31865400
C	-20.99684800	2.61865400	-3.77135800
C	-21.10895400	1.26049900	-3.41963300
C	-20.17345900	0.66471300	-2.57598200
H	-19.75541900	4.40802800	-3.61973900
H	-21.74431400	3.07841500	-4.42338400
H	-21.94421300	0.66848600	-3.80619800
H	-20.28225900	-0.38319400	-2.28637500
C	-17.71225800	3.46719200	-2.06726300
O	-17.55374600	4.68772200	-2.14346900
C	-15.53013900	3.00985000	-1.00603900
C	-15.75707000	3.99240200	0.16876100
H	-14.79575200	4.26848600	0.63197200
H	-16.25065300	4.89947000	-0.20046200
H	-16.39046200	3.52900300	0.94055600
C	-14.60962500	3.66027500	-2.06442200
H	-15.11812200	4.53269600	-2.49577600
H	-13.65819800	3.98636300	-1.61308800
H	-14.37815900	2.94297100	-2.86551900
C	-14.89158400	1.75272400	-0.46617600
N	-15.54239800	0.56415000	-0.61422600
N	-14.87560100	-0.40962200	-0.06028600
C	-13.73224600	1.47710400	0.23092700
H	-12.91436300	2.10058600	0.58001300
N	-13.77957400	0.13233400	0.45357200
N	-16.80410800	2.55335900	-1.59671500

Fe	-17.27660500	0.68011400	-1.71536500
P	-16.13689700	0.05053800	-4.07456600
P	-17.15802600	3.28956400	-7.10268900
C	-15.99385200	1.45100800	-5.30033800
H	-15.25897800	1.17729400	-6.07411300
H	-15.58593200	2.31469100	-4.75098800
C	-17.33386300	1.81847000	-5.94679000
H	-18.06311000	2.11264200	-5.17497600
H	-17.75052200	0.95284600	-6.48572900
C	-16.79261600	-1.32612300	-5.12531800
C	-17.93812500	-2.02922400	-4.72263000
C	-16.16616200	-1.67170700	-6.33740100
C	-18.45378300	-3.05313500	-5.52678100
H	-18.40646200	-1.79744300	-3.76416200
C	-16.68505100	-2.69127400	-7.13663800
H	-15.26372600	-1.14769200	-6.66258600
C	-17.83359800	-3.38299700	-6.73317100
H	-19.34392900	-3.59689500	-5.20002100
H	-16.18941100	-2.94944200	-8.07618600
H	-18.23870000	-4.18279700	-7.35889400
C	-14.38936900	-0.48725600	-3.80567500
C	-13.26499300	0.21068600	-4.27690300
C	-14.19241700	-1.65200000	-3.03877800
C	-11.97313900	-0.25589600	-4.00549400
H	-13.38122500	1.12193100	-4.86660000
C	-12.90336700	-2.12054000	-2.78025600
H	-15.05671100	-2.18474500	-2.63273500
C	-11.78826600	-1.42529900	-3.26445900
H	-11.10944900	0.29600000	-4.38593900
H	-12.76978300	-3.03513100	-2.19623500
H	-10.77904200	-1.79597000	-3.06580900
C	-16.09220800	2.54699200	-8.43775800
C	-16.50248800	1.48671000	-9.26636600
C	-14.80000300	3.07134300	-8.60864600
C	-15.64005800	0.96333300	-10.23253700
H	-17.50826100	1.07130500	-9.16136700
C	-13.93317500	2.54610800	-9.57410400
H	-14.47387500	3.90543300	-7.98052000
C	-14.35236100	1.49089200	-10.38769100
H	-15.97487200	0.14141900	-10.87129000
H	-12.93159100	2.96784300	-9.69424300
H	-13.68015800	1.08169900	-11.14659300
C	-18.80905500	3.32005500	-7.94420300
C	-19.93264100	2.59485100	-7.51277800
C	-18.96034100	4.19746800	-9.03602300
C	-21.16518700	2.73428400	-8.16297800
H	-19.86423900	1.91611200	-6.66109700
C	-20.18589800	4.32803500	-9.68963800
H	-18.10361700	4.78474500	-9.37995600
C	-21.29636500	3.59573600	-9.25366700
H	-22.02648300	2.15978400	-7.81125700

H	-20.27738800	5.00955100	-10.53968200
H	-22.25900300	3.70095200	-9.76068000
Cl	-17.95843600	-1.43263500	-1.21940000
C	-21.05099700	3.68113900	0.32515900
C	-20.93047400	2.33099100	0.11524200
H	-21.85506500	1.78214000	-0.10188100
C	-19.72800700	1.56552300	0.08593300
H	-18.80659800	2.00984600	0.46802400
H	-19.78905500	0.48185000	0.19977100
C	-12.79214300	-0.71055700	1.10453800
H	-13.27155100	-1.67045000	1.33024000
H	-11.93158800	-0.87786700	0.43936900
H	-12.45285500	-0.23772700	2.03659800
C	-22.41648300	4.32800000	0.25162500
H	-22.36744200	5.41194600	0.43326900
H	-22.87767600	4.17662700	-0.73935900
H	-23.10932600	3.89672500	0.99530300
C	-19.88761200	4.58823900	0.60637200
H	-18.97914700	4.04338100	0.89469000
H	-19.62728900	5.18783700	-0.28552000
H	-20.13330700	5.30153800	1.41204200

UB3LYP-D3/def2-SVP-SMD(THF)//UB3LYP/def2-SVP(gas)
HF= -4208.3905090
UPBEPBE-D3/def2-SVP-SMD(THF)//UB3LYP/def2-SVP(gas)
HF= -4400.7950856

[5]D''-outer-TS-dimethyl

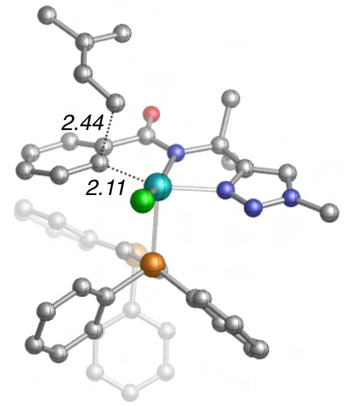

Zero-point correction= 0.805592 (Hartree/Particle)
Thermal correction to Energy= 0.859541
Thermal correction to Enthalpy= 0.860485
Thermal correction to Gibbs Free Energy= 0.708505
Sum of electronic and zero-point Energies= -4403.168913
Sum of electronic and thermal Energies= -4403.114964
Sum of electronic and thermal Enthalpies= -4403.114020
Sum of electronic and thermal Free Energies= -4403.266001

C	-18.88209500	1.01978900	-2.12729700
C	-18.96159700	2.36897700	-2.51511800
C	-20.04398300	2.82789900	-3.27215700
C	-21.03905500	1.93039500	-3.67508900
C	-20.94410500	0.57638700	-3.32148500
C	-19.87478300	0.12086500	-2.54218000
H	-20.07760200	3.88688100	-3.54219900
H	-21.88988500	2.28277700	-4.26470100
H	-21.71921100	-0.12688300	-3.64034500
H	-19.82922200	-0.92567500	-2.23070100
C	-17.84938300	3.29133800	-2.10215000
O	-17.97343200	4.51994000	-2.14916800
C	-15.55832400	3.33432400	-1.23137100
C	-15.86392600	4.19076400	0.02242200
H	-14.95564600	4.70387300	0.37872100
H	-16.62211300	4.94311200	-0.22881100
H	-16.24126400	3.55700900	0.84008300
C	-14.96500500	4.23145500	-2.34411200
H	-15.69942900	4.99625000	-2.62578900
H	-14.04301500	4.72691900	-1.99812000
H	-14.72142100	3.63352100	-3.23501900
C	-14.54819500	2.27168700	-0.85411400
N	-14.86541300	0.95061400	-0.95330500
N	-13.87748000	0.20122200	-0.55487200
C	-13.25723300	2.32637800	-0.36182000
H	-12.59325800	3.15226600	-0.12341600
N	-12.89672200	1.02256700	-0.19865900
N	-16.74708600	2.59788800	-1.69704600
Fe	-16.86844600	0.56905900	-1.66949400

P	-15.95721700	-0.24402300	-4.02541200
P	-16.45686200	3.26354700	-6.88543200
C	-15.64396100	1.12047500	-5.25298800
H	-15.09369600	0.71968100	-6.11817300
H	-14.96235200	1.82102700	-4.74275800
C	-16.90411400	1.85380700	-5.72366300
H	-17.42963600	2.29755700	-4.86404200
H	-17.60247000	1.15459800	-6.21030400
C	-16.91081400	-1.50967300	-4.98627200
C	-17.74837500	-2.39126100	-4.28214200
C	-16.80167600	-1.64295400	-6.38164700
C	-18.45906400	-3.38570600	-4.96227000
H	-17.83161200	-2.30690100	-3.19582000
C	-17.52103200	-2.63140900	-7.05799700
H	-16.15261900	-0.97991100	-6.95758600
C	-18.35150400	-3.50601500	-6.34988600
H	-19.10272500	-4.06748200	-4.40019800
H	-17.42757200	-2.71929600	-8.14366700
H	-18.91198500	-4.28035100	-6.88041400
C	-14.29754900	-1.06361100	-3.87992100
C	-13.17903600	-0.71678200	-4.65638700
C	-14.16615300	-2.08958100	-2.92595600
C	-11.96320100	-1.39027200	-4.49448900
H	-13.23908100	0.07775800	-5.40187200
C	-12.95537000	-2.76900400	-2.77720400
H	-15.01081200	-2.34371000	-2.28044800
C	-11.84936800	-2.42286700	-3.56038500
H	-11.10465700	-1.10877300	-5.11040200
H	-12.87588600	-3.56888600	-2.03627400
H	-10.90174700	-2.95549900	-3.44259900
C	-15.81151100	2.29960000	-8.34423600
C	-16.61896200	1.48383700	-9.15774700
C	-14.43926000	2.38658400	-8.63395500
C	-16.06554300	0.76882500	-10.22289000
H	-17.69230900	1.41438400	-8.96261100
C	-13.88216000	1.66754800	-9.69799600
H	-13.80260100	3.03190100	-8.02158800
C	-14.69481400	0.85705300	-10.49415900
H	-16.70748500	0.14238500	-10.84833200
H	-12.81237600	1.74799000	-9.90895100
H	-14.26410600	0.29864500	-11.32946800
C	-18.10932200	3.80206700	-7.52552500
C	-19.34384500	3.35585600	-7.02558500
C	-18.11365300	4.78324600	-8.53664600
C	-20.54532700	3.86653500	-7.53241800
H	-19.38873700	2.61066900	-6.23016900
C	-19.31113700	5.28346500	-9.04847100
H	-17.16400500	5.15928900	-8.92927000
C	-20.53502300	4.82618100	-8.54612500
H	-21.49485000	3.50535400	-7.12817800
H	-19.29001700	6.03953500	-9.83795200

H	-21.47451900	5.22159000	-8.94085500
Cl	-17.17173300	-1.52514100	-0.76219800
C	-20.90449400	2.88346600	0.62791800
C	-20.51958500	1.57143100	0.44662200
H	-21.32273000	0.82900800	0.37650400
C	-19.20386500	1.08476100	0.29414100
H	-18.34734900	1.74164000	0.45540000
H	-19.00670400	0.01454400	0.34622100
C	-11.64840700	0.48192200	0.30988100
H	-10.79845400	0.93650900	-0.21837700
H	-11.55420100	0.67207700	1.38972100
H	-11.65969400	-0.59882000	0.12600900
C	-22.35910700	3.24406300	0.71639400
H	-23.01599700	2.37095600	0.59172800
H	-22.59095100	3.72017500	1.68707000
H	-22.62201700	3.98814900	-0.05783300
C	-19.93870700	4.02848500	0.72111500
H	-18.93411100	3.71578300	1.03655300
H	-19.81931100	4.52506100	-0.25963700
H	-20.30177900	4.79185300	1.42924600

UB3LYP-D3/def2-SVP-SMD(THF)//UB3LYP/def2-SVP(gas)
HF= -4404.1767138
UPBEPBE-D3/def2-SVP-SMD(THF)//UB3LYP/def2-SVP(gas)
HF= -4400.7950763

[1]E-dimethyl

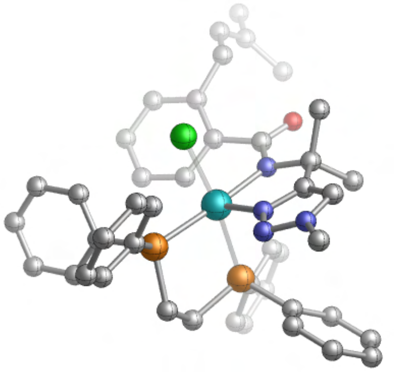

Zero-point correction= 0.811968 (Hartree/Particle)
Thermal correction to Energy= 0.864324
Thermal correction to Enthalpy= 0.865268
Thermal correction to Gibbs Free Energy= 0.720555
Sum of electronic and zero-point Energies= -4403.208341
Sum of electronic and thermal Energies= -4403.155985
Sum of electronic and thermal Enthalpies= -4403.155041
Sum of electronic and thermal Free Energies= -4403.299754

C	-20.19247000	1.19926800	-1.48937400
C	-19.17054700	1.51691300	-2.41981200
C	-19.11632100	0.79166900	-3.62143400
C	-20.04130000	-0.19719500	-3.95163600
C	-21.05639000	-0.49537600	-3.04344600
C	-21.11327300	0.19393900	-1.83244400
H	-18.36300700	1.05879600	-4.37390600
H	-19.96864300	-0.72568000	-4.90444500
H	-21.79813200	-1.26494200	-3.27313800
H	-21.90335300	-0.05318800	-1.11897500
C	-18.14014100	2.60471400	-2.15701300
O	-18.48815200	3.63885600	-1.54975900
C	-15.80383400	3.22241600	-2.08951200
C	-15.76089400	3.19587400	-0.53934000
H	-14.87937000	3.74387400	-0.16685600
H	-16.66532700	3.66606400	-0.13610700
H	-15.70934800	2.15349600	-0.19260000
C	-15.87908400	4.68214600	-2.59769900
H	-16.82357600	5.12516800	-2.25908300
H	-15.03859100	5.27929800	-2.20534300
H	-15.83368800	4.71745000	-3.69635900
C	-14.52105000	2.59066000	-2.57858000
N	-14.55046500	1.46941700	-3.34900100
N	-13.34626000	1.06100800	-3.63904600
C	-13.18466500	2.88558500	-2.38303800
H	-12.68033700	3.67362400	-1.83162500
N	-12.50949300	1.91109100	-3.05217500
N	-16.89532700	2.36921200	-2.61477800
Fe	-16.36621000	0.78562000	-3.71747600
P	-15.85238500	-1.12857600	-4.95199500
P	-16.39245400	1.92040300	-5.87295700

C	-15.37870000	-0.60264900	-6.68193700
H	-15.42151800	-1.43715000	-7.39841100
H	-14.31871200	-0.31553000	-6.61149000
C	-16.23929800	0.56959800	-7.16722500
H	-17.26952800	0.22588400	-7.35344900
H	-15.85283900	0.98223000	-8.11233400
C	-17.25270200	-2.31611700	-5.19520000
C	-17.80397200	-2.90785000	-4.04287100
C	-17.78759000	-2.64920600	-6.45061900
C	-18.84941500	-3.82607800	-4.15372600
H	-17.42244700	-2.62298000	-3.05848500
C	-18.84401100	-3.56159100	-6.55546300
H	-17.38834800	-2.20863200	-7.36644600
C	-19.37342700	-4.15646800	-5.40804700
H	-19.26498700	-4.27759700	-3.24934900
H	-19.24888700	-3.80891400	-7.54043000
H	-20.19542000	-4.87234800	-5.49060600
C	-14.46597900	-2.27624800	-4.51271200
C	-14.03740400	-3.25160500	-5.43135400
C	-13.83179600	-2.18955400	-3.26413800
C	-12.98188000	-4.11022900	-5.11706300
H	-14.53685200	-3.35951600	-6.39751600
C	-12.78018000	-3.05764000	-2.95012700
H	-14.19394300	-1.46684300	-2.53115800
C	-12.34832800	-4.01269400	-3.87361800
H	-12.66026400	-4.86338600	-5.84130000
H	-12.30164400	-2.98879600	-1.96942600
H	-11.52677600	-4.68926400	-3.62294000
C	-15.00947300	3.07820600	-6.32293500
C	-15.23639400	4.44921000	-6.53682700
C	-13.69348800	2.59398800	-6.44678200
C	-14.18315800	5.30669900	-6.86980000
H	-16.24476600	4.85658800	-6.45274500
C	-12.64455000	3.45068800	-6.79201900
H	-13.47126700	1.54305500	-6.26175900
C	-12.88410700	4.81167500	-7.00343000
H	-14.38606700	6.36830000	-7.03251600
H	-11.63337700	3.04814400	-6.89875100
H	-12.06342500	5.48139200	-7.27278300
C	-17.87741100	2.86793300	-6.42972000
C	-18.35066200	2.82446200	-7.75383000
C	-18.54840600	3.67974600	-5.49785400
C	-19.47134000	3.56792200	-8.13251400
H	-17.84605900	2.21637900	-8.50687900
C	-19.66602200	4.42701900	-5.88164800
H	-18.20286700	3.72757700	-4.46446400
C	-20.13208000	4.37053000	-7.19733300
H	-19.82814300	3.52003300	-9.16465900
H	-20.17672500	5.04469900	-5.13920200
H	-21.01078700	4.94861100	-7.49460200
Cl	-16.43162000	-0.53584800	-1.79578800

C	-22.36202100	3.33737900	0.06118000
C	-21.71062200	2.18985000	0.32679800
H	-22.24870300	1.43485500	0.91391800
C	-20.30347700	1.83354300	-0.10382600
H	-19.65480300	2.71331100	-0.06669900
H	-19.89179600	1.10331700	0.61626400
C	-11.07667500	1.74815900	-3.21306800
H	-10.57277700	1.90468500	-2.24918600
H	-10.89264100	0.72428000	-3.56023900
H	-10.68445200	2.46420500	-3.95167700
C	-23.76855000	3.56695200	0.55774600
H	-23.82169300	4.46640300	1.19823000
H	-24.46245600	3.74752500	-0.28369200
H	-24.14991400	2.71227700	1.13699700
C	-21.77198100	4.47031100	-0.74084500
H	-20.74716300	4.27306000	-1.08446300
H	-22.40088300	4.67640100	-1.62678700
H	-21.76727000	5.40298400	-0.14702500

UB3LYP-D3/def2-SVP-SMD(THF)//UB3LYP/def2-SVP(gas)
HF= -4404.2348647
UPBEPBE-D3/def2-SVP-SMD(THF)//UB3LYP/def2-SVP(gas)
HF= -4400.8697752

[3]E-dimethyl

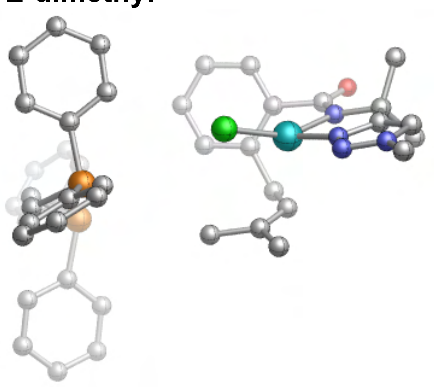

Zero-point correction=	0.809469 (Hartree/Particle)		
Thermal correction to Energy=	0.863567		
Thermal correction to Enthalpy=	0.864511		
Thermal correction to Gibbs Free Energy=	0.707198		
Sum of electronic and zero-point Energies=	-4403.202765		
Sum of electronic and thermal Energies=	-4403.148667		
Sum of electronic and thermal Enthalpies=	-4403.147723		
Sum of electronic and thermal Free Energies=	-4403.305036		
C	-17.79039500	-0.57920800	-5.88769500
C	-18.45557300	-0.14245000	-4.71775900
C	-19.67069100	-0.74293300	-4.33944600
C	-20.21395700	-1.79244800	-5.07488900
C	-19.55038200	-2.24336400	-6.22061900
C	-18.36335300	-1.63338500	-6.62290300
H	-20.18809300	-0.36368100	-3.45476600
H	-21.15198800	-2.25676400	-4.76038600
H	-19.95695700	-3.07270000	-6.80408100
H	-17.87633400	-1.98275400	-7.53647200
C	-17.96120800	0.99697400	-3.83818500
O	-18.59666100	2.04599100	-3.74472500
C	-16.29246800	1.53116000	-2.10675300
C	-17.32661900	1.79851100	-0.98771600
H	-16.88493600	2.40570900	-0.18071400
H	-18.18599900	2.33464100	-1.41009000
H	-17.67713800	0.84768300	-0.55786600
C	-15.78136100	2.86687700	-2.69223000
H	-16.62472700	3.40156900	-3.14895400
H	-15.33971500	3.50080600	-1.90576800
H	-15.01550500	2.68770900	-3.46253600
C	-15.13804000	0.73918500	-1.53370500
N	-14.87381500	-0.50577600	-2.03507700
N	-13.85952000	-1.05819000	-1.42769100
C	-14.20466600	0.95451300	-0.53967800
H	-14.02575400	1.78818600	0.13328900
N	-13.44797400	-0.18034600	-0.52189400
N	-16.84430500	0.64974000	-3.15456100
Fe	-16.06933200	-1.08369600	-3.46512100
P	-16.67583600	-6.17629400	-6.97204300

P	-17.91177400	-4.58944100	-9.94543300
C	-17.04025000	-7.01586600	-8.60727500
H	-17.26914100	-8.08485400	-8.46112100
H	-16.09684300	-6.97702400	-9.17696000
C	-18.17886600	-6.37326400	-9.41562200
H	-19.10558800	-6.35201600	-8.81798300
H	-18.40847800	-6.99316700	-10.29925800
C	-18.16371900	-6.63815100	-5.94939800
C	-18.59208700	-5.70969400	-4.98484300
C	-18.86340000	-7.85128100	-6.07502600
C	-19.69083300	-5.99139400	-4.16468100
H	-18.05277700	-4.76741100	-4.85675300
C	-19.96722200	-8.12648400	-5.26431300
H	-18.54344900	-8.60016700	-6.80408600
C	-20.38389300	-7.19575200	-4.30571800
H	-20.00162300	-5.26021500	-3.41379800
H	-20.50082100	-9.07444800	-5.37634100
H	-21.24557600	-7.41343700	-3.66878800
C	-15.39029200	-7.30447900	-6.24220600
C	-14.86633600	-6.92937700	-4.98897700
C	-14.87133100	-8.45110900	-6.86653900
C	-13.87428000	-7.69335000	-4.37185400
H	-15.23454700	-6.02666400	-4.49145100
C	-13.86669800	-9.20818200	-6.25213300
H	-15.24454000	-8.77209600	-7.84100900
C	-13.36832500	-8.83587400	-5.00200600
H	-13.48831300	-7.38764000	-3.39564000
H	-13.47610000	-10.09690700	-6.75584400
H	-12.58593200	-9.42984600	-4.52209700
C	-16.62292700	-4.73304100	-11.27510700
C	-15.99007400	-3.53843700	-11.66671600
C	-16.22002000	-5.92974500	-11.89323300
C	-15.00477600	-3.53434200	-12.65657500
H	-16.27606200	-2.59861300	-11.18524600
C	-15.22353800	-5.93021200	-12.87572800
H	-16.68438000	-6.87855000	-11.61595600
C	-14.61578500	-4.73315500	-13.26293900
H	-14.53184900	-2.59299200	-12.94885200
H	-14.92346100	-6.87270700	-13.34176400
H	-13.83783400	-4.73466100	-14.03080100
C	-19.46757900	-4.35551200	-10.94163100
C	-19.66222100	-4.86840700	-12.23579800
C	-20.51012000	-3.62419600	-10.34631900
C	-20.86996000	-4.66501500	-12.90904800
H	-18.86135600	-5.42570500	-12.72766400
C	-21.72233400	-3.42734500	-11.01529000
H	-20.36798500	-3.19598300	-9.35008300
C	-21.90443300	-3.94726800	-12.29952600
H	-21.00341300	-5.06795100	-13.91679000
H	-22.52171500	-2.85729600	-10.53450800
H	-22.84791300	-3.78807100	-12.82828300

Cl	-16.00872500	-3.32440100	-3.36239100
C	-14.48157700	-1.35109500	-5.83604300
C	-15.23445700	-0.23075200	-5.67870700
H	-14.81239100	0.57232800	-5.06662200
C	-16.52773100	0.10576100	-6.40577300
H	-16.67480100	1.19554000	-6.35181000
H	-16.42495200	-0.13990700	-7.47475400
C	-12.32358800	-0.50372200	0.34030200
H	-11.52534300	0.24334400	0.22212500
H	-12.64486900	-0.53521600	1.39171600
H	-11.95330700	-1.49088500	0.04006000
C	-14.85837800	-2.49928400	-6.72823300
H	-15.61359700	-2.23591700	-7.47897500
H	-13.96915000	-2.88680600	-7.25106400
H	-15.26591100	-3.32915100	-6.12684600
C	-13.17485700	-1.51753800	-5.10228800
H	-13.21708100	-2.41058200	-4.45669900
H	-12.35208600	-1.67352200	-5.82154700
H	-12.92850500	-0.64800200	-4.47578100

UB3LYP-D3/def2-SVP-SMD(THF)//UB3LYP/def2-SVP(gas)
HF= -4404.2041999
UPBEPBE-D3/def2-SVP-SMD(THF)//UB3LYP/def2-SVP(gas)
HF= -4400.8177125

^{5}E-dimethyl

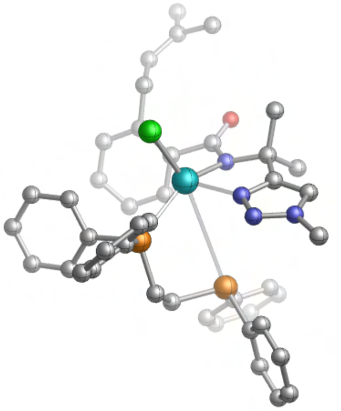

Zero-point correction= 0.809250 (Hartree/Particle)
Thermal correction to Energy= 0.863397
Thermal correction to Enthalpy= 0.864341
Thermal correction to Gibbs Free Energy= 0.708952
Sum of electronic and zero-point Energies= -4403.248253
Sum of electronic and thermal Energies= -4403.194106
Sum of electronic and thermal Enthalpies= -4403.193162
Sum of electronic and thermal Free Energies= -4403.348551

C	-20.25857600	0.54612600	-1.86482200
C	-19.50502700	1.46203100	-2.63225800
C	-19.55557600	1.40121600	-4.03693900
C	-20.36252300	0.46814800	-4.69218100
C	-21.10783200	-0.44060200	-3.93828200
C	-21.04077400	-0.40160400	-2.54379200
H	-18.97420900	2.12507700	-4.61366500
H	-20.41119900	0.45388100	-5.78425200
H	-21.73321200	-1.18714400	-4.43433200
H	-21.61117300	-1.13011900	-1.95932400
C	-18.70575400	2.58773300	-1.99448500
O	-19.29530900	3.45669000	-1.34649000
C	-16.50261900	3.61042200	-1.78778500
C	-16.48613600	3.70293200	-0.23959100
H	-15.77018700	4.46806800	0.10330600
H	-17.49106600	3.96300600	0.11542400
H	-16.19652000	2.73177000	0.19042300
C	-16.89923100	4.96878400	-2.40568900
H	-17.91350900	5.23011500	-2.07949900
H	-16.20848600	5.76771000	-2.08792700
H	-16.88392300	4.90505300	-3.50429200
C	-15.10328000	3.24544800	-2.24164700
N	-14.79741800	1.97652300	-2.62769100
N	-13.53361500	1.87292500	-2.93758800
C	-13.92655200	3.96879900	-2.31891100
H	-13.68629500	5.00698300	-2.10957200
N	-12.99505600	3.07390100	-2.74768700
N	-17.37741200	2.51994800	-2.25679700
Fe	-16.56008600	0.71910900	-2.61645200

P	-16.08706200	-0.95479300	-4.48662800
P	-16.17919300	2.40305600	-6.30324300
C	-15.61431100	-0.39265800	-6.20280900
H	-15.52855300	-1.28863600	-6.83802000
H	-14.58719100	-0.00575000	-6.10836700
C	-16.54155000	0.64389700	-6.86041100
H	-17.59632500	0.43980900	-6.61397200
H	-16.44490200	0.57512500	-7.95633700
C	-17.45722100	-2.16531500	-4.75678700
C	-18.04672600	-2.72946200	-3.60996800
C	-17.92174300	-2.55941600	-6.02255500
C	-19.05903500	-3.68279600	-3.73373200
H	-17.71359300	-2.41487000	-2.61676000
C	-18.94505400	-3.50626400	-6.14094300
H	-17.49308900	-2.13658400	-6.93308400
C	-19.51145800	-4.07475400	-4.99773500
H	-19.50237400	-4.11530700	-2.83332800
H	-19.29612700	-3.80128400	-7.13337400
H	-20.30728300	-4.81828400	-5.09135900
C	-14.65125200	-2.05706700	-4.09810100
C	-14.42744200	-3.23908000	-4.82637600
C	-13.75683500	-1.70546200	-3.07577600
C	-13.32061800	-4.04364900	-4.54911300
H	-15.12913900	-3.54468200	-5.60664800
C	-12.64957700	-2.51454000	-2.79984500
H	-13.92921000	-0.80855700	-2.48114000
C	-12.42750600	-3.68102600	-3.53489700
H	-13.15962500	-4.96121100	-5.12108700
H	-11.96418400	-2.23268200	-1.99647900
H	-11.56459100	-4.31443800	-3.31283500
C	-14.64073900	2.74005300	-7.29550600
C	-14.64299700	3.21585500	-8.61842300
C	-13.40207200	2.50027700	-6.67118300
C	-13.44217900	3.43611600	-9.29944700
H	-15.59080000	3.42085300	-9.12164500
C	-12.20129300	2.70517300	-7.35905700
H	-13.37856100	2.14920400	-5.63533000
C	-12.21811200	3.17779100	-8.67428400
H	-13.46343800	3.81022400	-10.32660700
H	-11.24850400	2.49910500	-6.86339800
H	-11.28065900	3.34748200	-9.21022200
C	-17.45862800	3.37697300	-7.21949800
C	-18.35151500	2.85028600	-8.16910900
C	-17.56642700	4.74189600	-6.88941700
C	-19.31660200	3.66477600	-8.77261300
H	-18.30486100	1.79706600	-8.45186000
C	-18.51987800	5.55774500	-7.50062300
H	-16.89605900	5.16815700	-6.13732400
C	-19.40162600	5.01954200	-8.44394500
H	-20.00477500	3.23461200	-9.50513200
H	-18.58434900	6.61453900	-7.22915500

H	-20.15726300	5.65328900	-8.91493200
Cl	-16.51283100	-0.72737000	-0.87174100
C	-21.59287900	2.21845300	1.09836300
C	-21.50192300	1.20020500	0.22557800
H	-22.44264200	0.77007100	-0.14256500
C	-20.25931400	0.55728200	-0.34414000
H	-19.34737500	1.03577800	0.03152100
H	-20.21245900	-0.49441100	-0.00620400
C	-11.59018900	3.29948000	-3.03869200
H	-11.11090800	2.31886400	-3.14440400
H	-11.47512000	3.86793300	-3.97414600
H	-11.12013900	3.85194000	-2.21313300
C	-22.94182500	2.73835400	1.53319100
H	-23.06353600	2.66935600	2.62971700
H	-23.05096700	3.80776100	1.27607900
H	-23.77024600	2.18878500	1.06125300
C	-20.41858300	2.94957700	1.69478900
H	-19.45347500	2.49094700	1.44872500
H	-20.39032600	3.98802500	1.32269300
H	-20.50960700	2.99863900	2.79471600

UB3LYP-D3/def2-SVP-SMD(THF)//UB3LYP/def2-SVP(gas)
HF= -4404.2658221
UPBEPBE-D3/def2-SVP-SMD(THF)//UB3LYP/def2-SVP(gas)
HF= -4400.8652789

[1]E'-dimethyl

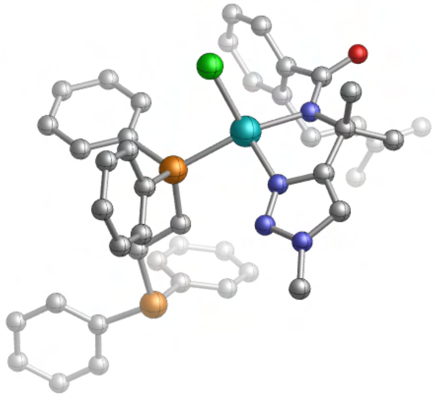

Zero-point correction= 0.810270 (Hartree/Particle)
Thermal correction to Energy= 0.864067
Thermal correction to Enthalpy= 0.865011
Thermal correction to Gibbs Free Energy= 0.712304
Sum of electronic and zero-point Energies= -4403.201649
Sum of electronic and thermal Energies= -4403.147852
Sum of electronic and thermal Enthalpies= -4403.146908
Sum of electronic and thermal Free Energies= -4403.299615

C	-20.79493800	-2.17877600	-3.75895800
C	-20.16223600	-1.64979800	-2.60987600
C	-20.49661900	-2.14992300	-1.34038300
C	-21.42851900	-3.17708500	-1.19649500
C	-22.05035700	-3.71208200	-2.32993000
C	-21.73326100	-3.21199400	-3.59341000
H	-20.00022100	-1.73342700	-0.46240900
H	-21.66944800	-3.55912300	-0.20109100
H	-22.78718300	-4.51388800	-2.22951000
H	-22.23252600	-3.61914500	-4.47763800
C	-19.23385600	-0.44676800	-2.69776100
O	-19.66712900	0.65678400	-2.35765100
C	-17.06967500	0.40674600	-3.45919000
C	-16.52214000	0.98481600	-2.13215400
H	-15.75461600	1.75528200	-2.31444600
H	-17.35596300	1.43507900	-1.57641600
H	-16.08872000	0.18109500	-1.51838000
C	-17.68166700	1.53067100	-4.32595800
H	-18.50669900	2.00665900	-3.78448900
H	-16.91852400	2.29127100	-4.56098800
H	-18.07067900	1.12521600	-5.27287400
C	-15.94720200	-0.23764600	-4.24262100
N	-15.89854600	-1.59919200	-4.33653400
N	-14.88187200	-1.98740900	-5.05988400
C	-14.86502000	0.23340600	-4.96030000
H	-14.49816300	1.23500900	-5.16458000
N	-14.24855700	-0.88537100	-5.43748100
N	-18.00054400	-0.71199100	-3.19671300
Fe	-17.20516400	-2.49002700	-3.13623600
Cl	-17.10919500	-2.96249600	-0.92384300

C	-22.26591800	-0.15423900	-6.42812700
C	-21.43013800	-0.38515600	-5.39904600
H	-21.35176300	0.38574900	-4.62313100
C	-20.56373700	-1.59825100	-5.14712700
H	-20.74378700	-2.37828000	-5.90146400
H	-19.50518600	-1.30360500	-5.23807800
C	-13.07941900	-0.97682100	-6.29558400
H	-13.34278500	-0.74255900	-7.33827000
H	-12.30839300	-0.27661200	-5.94625600
H	-12.69837600	-2.00302900	-6.23494200
P	-16.53617700	-4.68245200	-3.68629800
C	-16.72531800	-4.98852600	-5.51908300
H	-16.01914600	-4.29409900	-6.00412900
H	-17.73060300	-4.60085800	-5.75468500
C	-16.57787900	-6.41542700	-6.05508900
H	-17.36428400	-7.06320100	-5.63805000
H	-15.60747100	-6.84722400	-5.76150400
P	-16.61740000	-6.44316900	-7.93411100
C	-18.40919000	-6.04891900	-8.24148100
C	-18.71862200	-4.79990100	-8.80553200
C	-19.46305600	-6.92554700	-7.92629400
C	-20.04767800	-4.42772300	-9.03800200
H	-17.90824100	-4.11456700	-9.07012100
C	-20.78953500	-6.55750900	-8.16176200
H	-19.24513700	-7.90854500	-7.50058600
C	-21.08522200	-5.30680500	-8.71670800
H	-20.27172400	-3.45216300	-9.47738200
H	-21.59763200	-7.25059100	-7.91307500
H	-22.12372800	-5.02097800	-8.90283900
C	-16.56772800	-8.25885700	-8.30374200
C	-16.59705400	-8.63090300	-9.66205100
C	-16.43654500	-9.27280300	-7.33980200
C	-16.52047700	-9.97094200	-10.04303600
H	-16.68214200	-7.85711900	-10.43109900
C	-16.34636800	-10.61674000	-7.72239200
H	-16.40718700	-9.02843100	-6.27652800
C	-16.39189800	-10.97081900	-9.07211200
H	-16.55288400	-10.23667700	-11.10302500
H	-16.24469100	-11.38994500	-6.95607600
H	-16.32401300	-12.02061100	-9.36872000
C	-17.48187100	-6.05145700	-2.89535300
C	-18.77250400	-5.78558000	-2.41217300
C	-16.96546300	-7.35486300	-2.78490100
C	-19.54129000	-6.81009400	-1.85196400
H	-19.17763800	-4.77282400	-2.44788400
C	-17.73331500	-8.37485300	-2.21783800
H	-15.95209200	-7.57316000	-3.12937200
C	-19.02537400	-8.10472600	-1.75448900
H	-20.54293800	-6.58400000	-1.47914800
H	-17.31857900	-9.38272600	-2.13211100
H	-19.62476300	-8.90229000	-1.30754900

C	-14.78005800	-5.06400600	-3.28143900
C	-13.78013700	-5.22047100	-4.25524800
C	-14.41966000	-5.12198400	-1.92151900
C	-12.45317700	-5.45640800	-3.87949100
H	-14.02325300	-5.15394500	-5.31663300
C	-13.09359000	-5.35550000	-1.55165900
H	-15.18052700	-4.95767800	-1.15477500
C	-12.10672100	-5.52818700	-2.52797200
H	-11.68860500	-5.58679600	-4.65020500
H	-12.83027900	-5.39691900	-0.49160400
H	-11.06996900	-5.71419300	-2.23551500
C	-23.05684600	1.13001500	-6.50057500
H	-22.82701000	1.68978400	-7.42581000
H	-24.14302500	0.92638200	-6.52445500
H	-22.85140600	1.78689700	-5.64275300
C	-22.50377900	-1.09915900	-7.58016500
H	-21.94033000	-2.03873600	-7.50027000
H	-23.57625400	-1.35267900	-7.66112800
H	-22.22876000	-0.62180500	-8.53853200

UB3LYP-D3/def2-SVP-SMD(THF)//UB3LYP/def2-SVP(gas)
HF= -4404.1905511
UPBEPBE-D3/def2-SVP-SMD(THF)//UB3LYP/def2-SVP(gas)
HF= -4400.8110272

[3]E'-dimethyl

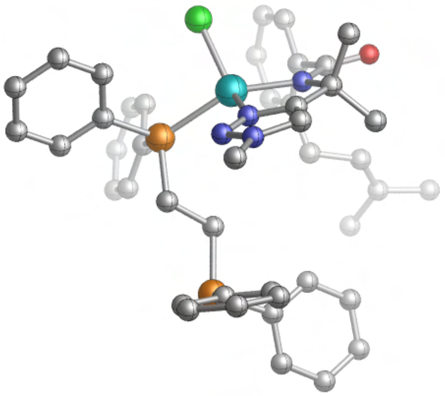

Zero-point correction= 0.810532 (Hartree/Particle)
Thermal correction to Energy= 0.864139
Thermal correction to Enthalpy= 0.865083
Thermal correction to Gibbs Free Energy= 0.713795
Sum of electronic and zero-point Energies= -4403.220047
Sum of electronic and thermal Energies= -4403.166440
Sum of electronic and thermal Enthalpies= -4403.165496
Sum of electronic and thermal Free Energies= -4403.316784

C	-21.01693600	-2.65797200	-5.18976900
C	-20.45863700	-1.83401700	-4.18984200
C	-20.97744800	-1.87729100	-2.88595600
C	-22.01556800	-2.74563400	-2.55509000
C	-22.55913100	-3.58007500	-3.53725600
C	-22.06316200	-3.52781600	-4.83957800
H	-20.54559400	-1.22777200	-2.12268700
H	-22.39798400	-2.77383000	-1.53152200
H	-23.37453400	-4.26563100	-3.29052200
H	-22.50152400	-4.16687300	-5.61164500
C	-19.42855000	-0.76774200	-4.51905000
O	-19.83805100	0.38661300	-4.67791900
C	-17.14112100	-0.12885100	-5.02472300
C	-16.98415000	0.93145500	-3.90779500
H	-16.19094100	1.65538900	-4.15843400
H	-17.93316400	1.46828100	-3.78729900
H	-16.72813100	0.44279300	-2.95539300
C	-17.46897600	0.54991000	-6.37722300
H	-18.41186400	1.10143900	-6.29288200
H	-16.66638600	1.25008400	-6.66249100
H	-17.56681100	-0.20514500	-7.17288800
C	-15.83037000	-0.86098900	-5.17791700
N	-15.77263000	-2.19432800	-4.91517300
N	-14.55959900	-2.66109600	-5.07322900
C	-14.54525300	-0.48568100	-5.52453800
H	-14.10720600	0.46720400	-5.80641400
N	-13.81237300	-1.63183700	-5.43692400
N	-18.13315800	-1.17080900	-4.64790300
Fe	-17.43977900	-2.93772100	-4.13858000
P	-17.00044500	-5.23089000	-4.59567600

C	-16.20108600	-5.45613800	-6.26538300
H	-16.06028200	-6.53058500	-6.46417700
H	-15.19541700	-5.02143200	-6.15362400
C	-18.48217900	-6.33179300	-4.62361100
C	-19.43624300	-6.16945900	-3.60261900
C	-18.68628500	-7.32579100	-5.59716100
C	-20.55801300	-7.00049800	-3.55074300
H	-19.29966600	-5.38529700	-2.85263700
C	-19.81517000	-8.14886500	-5.54395000
H	-17.97110600	-7.47143900	-6.40922700
C	-20.75092100	-7.99116800	-4.51792500
H	-21.29050500	-6.86112600	-2.75209800
H	-19.96138900	-8.91559200	-6.30932600
H	-21.63281800	-8.63586000	-4.47695200
C	-15.81524500	-6.11231100	-3.48490400
C	-15.27550500	-5.44568500	-2.37400500
C	-15.43364100	-7.44171200	-3.73812600
C	-14.35511300	-6.09423100	-1.54348800
H	-15.60701000	-4.43315800	-2.13644000
C	-14.51300400	-8.08471300	-2.90859900
H	-15.86580400	-7.98993700	-4.57911900
C	-13.96829700	-7.40937000	-1.81065000
H	-13.94735500	-5.56725600	-0.67698600
H	-14.22551500	-9.11891600	-3.11550500
H	-13.25082300	-7.91451600	-1.15854000
Cl	-18.06548500	-3.25233000	-1.98057200
C	-22.19318100	-1.60435700	-8.42329400
C	-21.35423900	-1.47462500	-7.37780300
H	-21.21629500	-0.47255900	-6.95438400
C	-20.59031400	-2.55525900	-6.64716500
H	-20.72375100	-3.53264700	-7.13372100
H	-19.51306000	-2.32188800	-6.68542100
P	-16.30646800	-5.29274400	-9.09597500
C	-17.32453300	-4.26327500	-10.25003800
C	-17.17329700	-4.52252300	-11.62614300
C	-18.25350800	-3.28606200	-9.85410800
C	-17.90657600	-3.80997200	-12.57627400
H	-16.47126300	-5.29457700	-11.95557100
C	-18.99807200	-2.57886800	-10.80591900
H	-18.41261300	-3.06043600	-8.79877000
C	-18.82334400	-2.83450900	-12.16791100
H	-17.76937700	-4.02331800	-13.63968300
H	-19.71465100	-1.82557400	-10.46910500
H	-19.40377000	-2.28052700	-12.91016600
C	-16.95756500	-4.77344600	-7.41142700
H	-16.92777400	-3.67945800	-7.28780600
H	-18.01943600	-5.07163900	-7.40937400
C	-14.66706800	-4.41031700	-9.16166200
C	-13.51106800	-5.16809500	-8.90182700
C	-14.51598500	-3.05476300	-9.50472000
C	-12.23970200	-4.58957400	-8.97253200

H	-13.60804600	-6.23026700	-8.65903600
C	-13.24534400	-2.47773000	-9.58975500
H	-15.39725100	-2.44871700	-9.72818200
C	-12.10319600	-3.24342200	-9.32545600
H	-11.35356200	-5.19796500	-8.77285100
H	-13.14611200	-1.42743600	-9.87827500
H	-11.10909000	-2.79578800	-9.41059600
C	-12.39992900	-1.82621200	-5.72588000
H	-11.84066800	-0.93240500	-5.41954500
H	-12.05316400	-2.69445900	-5.15279800
H	-12.25135800	-2.01460500	-6.79933500
C	-22.89724600	-0.39652500	-8.99506500
H	-22.66193900	-0.26494200	-10.06719400
H	-23.99464900	-0.51257200	-8.93273700
H	-22.62195500	0.52805800	-8.46686900
C	-22.53706000	-2.90612200	-9.10460700
H	-22.30277900	-2.86007800	-10.18317300
H	-22.00840000	-3.77311600	-8.68681800
H	-23.62160700	-3.10478900	-9.03254400

UB3LYP-D3/def2-SVP-SMD(THF)//UB3LYP/def2-SVP(gas)
HF= -4404.2340409
UPBEPBE-D3/def2-SVP-SMD(THF)//UB3LYP/def2-SVP(gas)
HF= -4400.8496583

^{5}E'-dimethyl

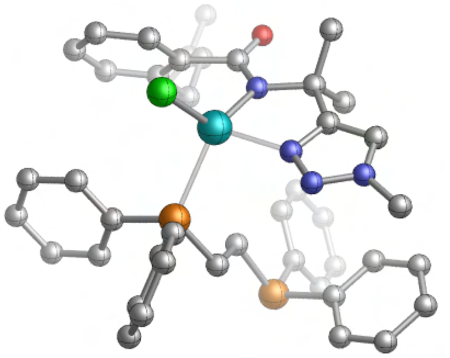

Zero-point correction= 0.810158 (Hartree/Particle)
Thermal correction to Energy= 0.864077
Thermal correction to Enthalpy= 0.865022
Thermal correction to Gibbs Free Energy= 0.711436
Sum of electronic and zero-point Energies= -4403.248615
Sum of electronic and thermal Energies= -4403.194695
Sum of electronic and thermal Enthalpies= -4403.193751
Sum of electronic and thermal Free Energies= -4403.347337

C	-21.11933100	-2.59836000	-5.58671600
C	-20.20055000	-1.94588400	-4.73278800
C	-20.35466800	-2.05197200	-3.34118400
C	-21.39770600	-2.79676000	-2.78507700
C	-22.29935700	-3.45259100	-3.62483200
C	-22.15163400	-3.35233800	-5.01042500
H	-19.65366000	-1.54051600	-2.68009000
H	-21.49671000	-2.86143100	-1.69863600
H	-23.11958100	-4.04131200	-3.20495400
H	-22.85979400	-3.86704000	-5.66653900
C	-19.14518400	-1.01484400	-5.30166400
O	-19.53800400	-0.05889300	-5.98407100
C	-16.82420500	-0.33856300	-5.47038500
C	-16.97400900	1.02463000	-4.75185200
H	-16.18161100	1.72729800	-5.05883400
H	-17.94934500	1.45876000	-5.00635500
H	-16.91593800	0.88878900	-3.66095800
C	-16.84481300	-0.13677600	-7.00722300
H	-17.79691500	0.31300000	-7.30772400
H	-16.01646900	0.51923500	-7.32180400
H	-16.72966700	-1.10279500	-7.52251700
C	-15.47056100	-0.92690600	-5.11943000
N	-15.35563000	-2.11376100	-4.46478300
N	-14.10272300	-2.43745300	-4.28265200
C	-14.17644400	-0.49254600	-5.35172900
H	-13.77261400	0.39019000	-5.83839000
N	-13.38282400	-1.46094800	-4.81766000
N	-17.85123000	-1.30137000	-4.99624500
Fe	-17.22887100	-2.83370000	-3.80355300
P	-16.94105500	-5.30205400	-4.52704500
C	-16.20325100	-5.53414100	-6.21896000

H	-16.11730200	-6.61095900	-6.43885000
H	-15.17264000	-5.15568200	-6.11768000
C	-18.48074200	-6.30648300	-4.48046000
C	-19.33216900	-6.11011000	-3.37765600
C	-18.83008200	-7.25225300	-5.45861100
C	-20.50150400	-6.86259500	-3.25157500
H	-19.07972600	-5.36120900	-2.62079500
C	-20.00738200	-7.99608600	-5.33105000
H	-18.19080300	-7.42047100	-6.32802700
C	-20.84169200	-7.80600600	-4.22629400
H	-21.15484400	-6.70102400	-2.39103100
H	-20.27111300	-8.72715900	-6.09969400
H	-21.76117200	-8.38882400	-4.12841000
C	-15.76448000	-6.25097400	-3.46498200
C	-15.11929100	-5.61163900	-2.39501100
C	-15.49702500	-7.60883100	-3.71735200
C	-14.20777100	-6.31850500	-1.60269000
H	-15.35167400	-4.57185500	-2.15743800
C	-14.58541900	-8.30877500	-2.92562200
H	-16.01171200	-8.13221200	-4.52742100
C	-13.93587900	-7.66221000	-1.86757100
H	-13.71610500	-5.81212400	-0.76822400
H	-14.38652900	-9.36402300	-3.13012100
H	-13.22525200	-8.21188700	-1.24467400
Cl	-17.52771200	-3.01393400	-1.55286500
C	-22.97783700	-1.53740300	-8.50407000
C	-21.94312600	-1.40774800	-7.65260500
H	-21.68964300	-0.41008500	-7.27919200
C	-21.04302500	-2.48843900	-7.10278500
H	-21.28433200	-3.46752400	-7.54360400
H	-20.00799700	-2.24947900	-7.39415600
P	-16.17891500	-5.19934200	-9.01776500
C	-17.16281900	-4.14657400	-10.17703700
C	-16.86178000	-4.25803000	-11.54866300
C	-18.22085800	-3.30629100	-9.79333200
C	-17.57918100	-3.53334600	-12.50146000
H	-16.05497900	-4.92233700	-11.87302900
C	-18.95086900	-2.58982100	-10.74949100
H	-18.49389400	-3.19798000	-8.74312800
C	-18.62928000	-2.69736800	-12.10411200
H	-17.32468500	-3.62919200	-13.56036600
H	-19.77172400	-1.94503400	-10.42463900
H	-19.19771300	-2.13621800	-12.85019900
C	-16.93686700	-4.79534700	-7.34340300
H	-16.95023100	-3.71182500	-7.14321400
H	-17.98700600	-5.12626900	-7.40121300
C	-14.57022900	-4.26612500	-8.91951600
C	-13.41259100	-4.99932000	-8.60242200
C	-14.44344000	-2.88790700	-9.16862000
C	-12.16469200	-4.37296600	-8.51848300
H	-13.48784300	-6.07754000	-8.43398300

C	-13.19542100	-2.26236900	-9.09711700
H	-15.32447100	-2.29967500	-9.43538800
C	-12.05266100	-3.00233700	-8.77088900
H	-11.27634000	-4.96164300	-8.27441400
H	-13.11415900	-1.19266300	-9.30903000
H	-11.07422700	-2.51506600	-8.73332900
C	-11.93272600	-1.55167100	-4.83169800
H	-11.57853500	-1.83050600	-5.83544700
H	-11.49541400	-0.58774600	-4.53719200
H	-11.64124600	-2.32582900	-4.11209500
C	-23.46024000	-2.83861500	-9.09740400
H	-24.51504900	-3.02297100	-8.82411900
H	-23.43193000	-2.80062600	-10.20140300
H	-22.87398000	-3.71085400	-8.77811200
C	-23.76964400	-0.32581300	-8.93575300
H	-23.74960500	-0.20488400	-10.03444200
H	-24.83437600	-0.42698400	-8.65608500
H	-23.38369000	0.59866400	-8.48195300

UB3LYP-D3/def2-SVP-SMD(THF)//UB3LYP/def2-SVP(gas)
HF= -4404.2660114
UPBEPBE-D3/def2-SVP-SMD(THF)//UB3LYP/def2-SVP(gas)
HF= -4400.8654964

[1]E'-complex-dimethyl

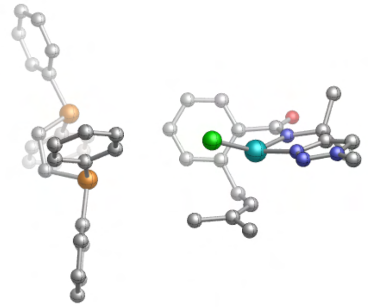

Zero-point correction= 0.809512 (Hartree/Particle)
Thermal correction to Energy= 0.863603
Thermal correction to Enthalpy= 0.864547
Thermal correction to Gibbs Free Energy= 0.707786
Sum of electronic and zero-point Energies= -4403.189752
Sum of electronic and thermal Energies= -4403.135661
Sum of electronic and thermal Enthalpies= -4403.134717
Sum of electronic and thermal Free Energies= -4403.291478

C	-15.63337900	-0.79044200	-5.97487700
C	-16.60961600	0.17999500	-5.64587500
C	-17.89483400	0.09364200	-6.21139200
C	-18.24089300	-0.95469100	-7.05967700
C	-17.28730600	-1.92588900	-7.38075600
C	-16.00077100	-1.82715300	-6.85214200
H	-18.62206500	0.87396700	-5.97393300
H	-19.24912400	-1.01435700	-7.47740300
H	-17.54034400	-2.75611700	-8.04476600
H	-15.25758800	-2.57427300	-7.14182900
C	-16.35369600	1.37628800	-4.74232700
O	-16.41588700	2.52184600	-5.18744000
C	-16.05082600	2.03501000	-2.39298900
C	-17.33038100	2.90093000	-2.31421300
H	-17.26925600	3.61970700	-1.48059700
H	-17.45080200	3.45310200	-3.25493600
H	-18.21385100	2.26232500	-2.16123400
C	-14.80720600	2.93389000	-2.58044700
H	-14.89855500	3.47636500	-3.53044000
H	-14.71850300	3.66285200	-1.75814300
H	-13.88860700	2.32736900	-2.60495000
C	-15.91801300	1.23416000	-1.11619300
N	-15.91439800	-0.13099400	-1.18399500
N	-15.80786700	-0.66650000	0.00106100
C	-15.80385300	1.54877500	0.22338300
H	-15.76923500	2.49430800	0.75690500
N	-15.73895900	0.34342000	0.85878900
N	-16.15315700	1.01070800	-3.45143500
Fe	-16.12938400	-0.86019100	-2.98808700
P	-16.44054100	-5.94347900	-7.78038400
P	-18.82490800	-4.78839100	-10.09791700
C	-17.11431900	-6.94905900	-9.22460100

H	-17.04791600	-8.02726400	-9.00267300
H	-16.41332000	-6.76727500	-10.05577100
C	-18.54537100	-6.59601500	-9.64975300
H	-19.24911500	-6.79735600	-8.82499000
H	-18.86068900	-7.24713800	-10.48247400
C	-17.44819600	-6.55990900	-6.35298400
C	-17.70411000	-5.65204400	-5.30978500
C	-17.97609400	-7.86105600	-6.25389000
C	-18.44963200	-6.03564200	-4.18954800
H	-17.32447400	-4.62820500	-5.36100400
C	-18.73335200	-8.24144200	-5.14274800
H	-17.79131100	-8.59283400	-7.04441200
C	-18.96855100	-7.33002700	-4.10682800
H	-18.61478600	-5.30870400	-3.39099600
H	-19.13792200	-9.25572900	-5.08356400
H	-19.55815100	-7.63097000	-3.23655300
C	-14.81271700	-6.81481100	-7.56604200
C	-14.48372200	-7.62447600	-6.46608900
C	-13.82275900	-6.58060400	-8.54091700
C	-13.21087000	-8.19596500	-6.35473200
H	-15.22598400	-7.81478200	-5.68833800
C	-12.55782000	-7.16263000	-8.43697400
H	-14.04107700	-5.92713600	-9.39167200
C	-12.24604500	-7.97351100	-7.34021200
H	-12.97610700	-8.82351900	-5.49044900
H	-11.80851800	-6.97321700	-9.21050400
H	-11.25386500	-8.42391000	-7.25268700
C	-17.85639200	-4.60488200	-11.66880500
C	-17.33608800	-3.32828300	-11.94689600
C	-17.59011500	-5.64577700	-12.57685600
C	-16.58571800	-3.09249400	-13.10285600
H	-17.51770300	-2.51157200	-11.24251500
C	-16.83125900	-5.41505500	-13.72812200
H	-17.98120100	-6.64947500	-12.39356200
C	-16.32930100	-4.13711800	-13.99520900
H	-16.19335800	-2.09166000	-13.30128000
H	-16.63289400	-6.23720000	-14.42113200
H	-15.73608000	-3.95797400	-14.89579200
C	-20.58231600	-4.90627300	-10.68797000
C	-20.98002700	-4.87286500	-12.03462300
C	-21.58035900	-4.97735600	-9.69679400
C	-22.33523300	-4.92577100	-12.37932400
H	-20.22938200	-4.80387800	-12.82453900
C	-22.93177400	-5.04495900	-10.04167700
H	-21.29670200	-4.97140900	-8.63958400
C	-23.31438200	-5.01767700	-11.38705800
H	-22.62520900	-4.89634700	-13.43329000
H	-23.68963600	-5.10642600	-9.25603700
H	-24.37217800	-5.06042100	-11.65933300
Cl	-17.20079000	-2.81131800	-2.70643500
C	-13.82753300	-2.34606100	-3.52667800

C	-13.91645000	-1.08880500	-4.03927700
H	-13.61706600	-0.26684300	-3.38107000
C	-14.18799000	-0.69799200	-5.48652400
H	-13.84076700	0.33809600	-5.62111200
H	-13.56766200	-1.31829200	-6.15322500
C	-15.62874200	0.07758700	2.28328600
H	-15.56818100	-1.00953400	2.41071300
H	-14.72302800	0.54990400	2.69048300
H	-16.51285800	0.46135200	2.81320000
C	-14.08727400	-3.60425700	-4.30622600
H	-14.17664300	-3.43957000	-5.38606000
H	-13.27743900	-4.33200200	-4.13160500
H	-15.02538800	-4.06409000	-3.95214000
C	-13.43891600	-2.57062200	-2.08666500
H	-14.24848700	-3.09698300	-1.55408700
H	-12.54121600	-3.21085500	-2.03161800
H	-13.22731900	-1.63145400	-1.55572400

UB3LYP-D3/def2-SVP-SMD(THF)//UB3LYP/def2-SVP(gas)
HF= -4404.1601730
UPBEPBE-D3/def2-SVP-SMD(THF)//UB3LYP/def2-SVP(gas)
HF= -4400.7774363

^{3}E’-complex-dimethyl

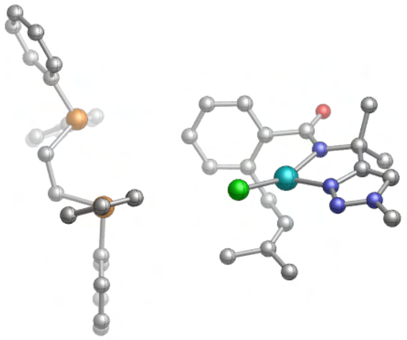

Zero-point correction= 0.809662 (Hartree/Particle)
Thermal correction to Energy= 0.863717
Thermal correction to Enthalpy= 0.864661
Thermal correction to Gibbs Free Energy= 0.707328
Sum of electronic and zero-point Energies= -4403.204316
Sum of electronic and thermal Energies= -4403.150260
Sum of electronic and thermal Enthalpies= -4403.149316
Sum of electronic and thermal Free Energies= -4403.306650

C	-15.53867600	-0.77404700	-6.00694200
C	-16.50745400	0.21108400	-5.69911300
C	-17.80010700	0.11346200	-6.24825300
C	-18.15727300	-0.95779400	-7.06193800
C	-17.21129200	-1.94484200	-7.35910800
C	-15.92015300	-1.83867000	-6.84427200
H	-18.52314900	0.90248000	-6.02627400
H	-19.16903500	-1.02568600	-7.46964700
H	-17.47585100	-2.79446000	-7.99348600
H	-15.18489000	-2.60170600	-7.11150100
C	-16.23115500	1.42591400	-4.82413400
O	-16.21996600	2.56319200	-5.29135200
C	-16.02866800	2.08030700	-2.45303400
C	-17.27219000	3.00035900	-2.45269300
H	-17.23232600	3.71693600	-1.61603300
H	-17.30981700	3.55580600	-3.39869800
H	-18.19020000	2.40060100	-2.35635000
C	-14.73949400	2.92613100	-2.55536400
H	-14.74591200	3.47070500	-3.50888800
H	-14.67572300	3.65290100	-1.72882700
H	-13.84635600	2.28341900	-2.52028700
C	-16.01666400	1.25933100	-1.18236300
N	-16.09004700	-0.10424800	-1.27536400
N	-16.08315000	-0.66253400	-0.09590100
C	-15.96176800	1.54591200	0.16662000
H	-15.90175300	2.47967500	0.71836600
N	-16.00448300	0.32925000	0.78238000
N	-16.10676200	1.06880300	-3.52354100
Fe	-16.18989600	-0.79867100	-3.09151800
P	-16.42972900	-5.99337300	-7.76188900
P	-18.83854600	-4.80138100	-10.03599400

C	-17.13323100	-6.98229600	-9.20342000
H	-17.07257500	-8.06261400	-8.99034600
H	-16.44377300	-6.80026500	-10.04408800
C	-18.56755300	-6.61442100	-9.60422400
H	-19.26044700	-6.81557400	-8.77027400
H	-18.90097500	-7.25693500	-10.43655500
C	-17.42229500	-6.61344000	-6.32547200
C	-17.66122600	-5.71013100	-5.27446600
C	-17.95593300	-7.91241200	-6.22794300
C	-18.39761200	-6.09618800	-4.14897600
H	-17.27527800	-4.68839200	-5.32197100
C	-18.70321200	-8.29512800	-5.11094000
H	-17.78343100	-8.64068700	-7.02445500
C	-18.92292300	-7.38802900	-4.06778700
H	-18.55174500	-5.37265100	-3.34509800
H	-19.11239500	-9.30763100	-5.05292900
H	-19.50533400	-7.69056200	-3.19320400
C	-14.80623500	-6.87926600	-7.57828900
C	-14.46631800	-7.69418000	-6.48562800
C	-13.83046800	-6.65122600	-8.56876700
C	-13.19705000	-8.27728900	-6.39657400
H	-15.19721500	-7.87918100	-5.69595500
C	-12.56918300	-7.24462000	-8.48688400
H	-14.05679300	-5.99362400	-9.41421500
C	-12.24670900	-8.06107000	-7.39736500
H	-12.95356900	-8.90870900	-5.53754200
H	-11.83096000	-7.05981400	-9.27209400
H	-11.25726400	-8.52043700	-7.32725400
C	-17.88859300	-4.61487200	-11.61787500
C	-17.35866000	-3.34155200	-11.89267800
C	-17.64512000	-5.65106500	-12.53770500
C	-16.62119200	-3.10419300	-13.05661900
H	-17.52230000	-2.52868200	-11.17954200
C	-16.89953300	-5.41880300	-13.69727600
H	-18.04373500	-6.65230900	-12.35713000
C	-16.38781300	-4.14399400	-13.96080500
H	-16.22093100	-2.10592300	-13.25216100
H	-16.71904500	-6.23732600	-14.39939900
H	-15.80483800	-3.96361100	-14.86780700
C	-20.60399900	-4.90238200	-10.60507400
C	-21.01810800	-4.85674200	-11.94642300
C	-21.59039200	-4.97345600	-9.60224800
C	-22.37776400	-4.89821800	-12.27481200
H	-20.27685300	-4.78713000	-12.74510000
C	-22.94637900	-5.02954700	-9.93095000
H	-21.29387900	-4.97687100	-8.54854800
C	-23.34531100	-4.99049200	-11.27127600
H	-22.68038100	-4.85951400	-13.32491600
H	-23.69494100	-5.09136900	-9.13645900
H	-24.40663500	-5.02433400	-11.53078300
Cl	-17.11030700	-2.82685000	-2.83612700

C	-13.73008600	-2.25705900	-3.52479300
C	-13.84250600	-1.01728800	-4.06664300
H	-13.61757400	-0.16874300	-3.41338300
C	-14.09236600	-0.67114100	-5.52681000
H	-13.74050600	0.35854900	-5.69554300
H	-13.47507400	-1.31692100	-6.17114000
C	-15.98683100	0.03517000	2.20539200
H	-16.00837900	-1.05536500	2.31492100
H	-15.07259900	0.43602400	2.66677300
H	-16.86823100	0.47204000	2.69735000
C	-13.91363300	-3.53866800	-4.28814400
H	-13.94369500	-3.39899400	-5.37532900
H	-13.10006300	-4.24412200	-4.05088400
H	-14.86126800	-4.01130200	-3.97879000
C	-13.40453300	-2.43755100	-2.06331300
H	-14.22285200	-2.97782600	-1.55867500
H	-12.49068000	-3.04614100	-1.94791700
H	-13.24988300	-1.47917200	-1.54670000

UB3LYP-D3/def2-SVP-SMD(THF)//UB3LYP/def2-SVP(gas)
HF= -4404.2021638
UPBEPBE-D3/def2-SVP-SMD(THF)//UB3LYP/def2-SVP(gas)
HF= -4400.8172635

[5]E'-complex-dimethyl

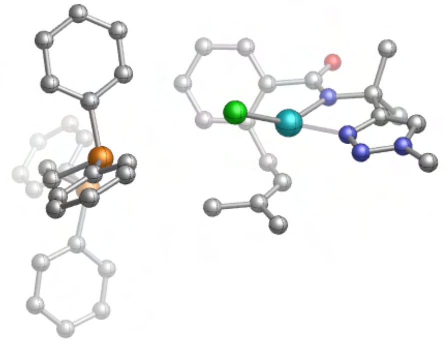

Zero-point correction= 0.809132 (Hartree/Particle)
Thermal correction to Energy= 0.863313
Thermal correction to Enthalpy= 0.864257
Thermal correction to Gibbs Free Energy= 0.706750
Sum of electronic and zero-point Energies= -4403.235585
Sum of electronic and thermal Energies= -4403.181404
Sum of electronic and thermal Enthalpies= -4403.180460
Sum of electronic and thermal Free Energies= -4403.337966

C	-17.90630900	-0.66979300	-5.75254000
C	-18.58105700	-0.31923400	-4.55790000
C	-19.79205000	-0.95308100	-4.22970900
C	-20.32186700	-1.95988500	-5.03248800
C	-19.64912000	-2.32833300	-6.20100700
C	-18.46727200	-1.67912000	-6.55631800
H	-20.31676200	-0.63233800	-3.32673800
H	-21.25698200	-2.45200500	-4.75334100
H	-20.04546000	-3.12018800	-6.84087700
H	-17.97615300	-1.96061100	-7.49096600
C	-18.11311600	0.76628300	-3.59978700
O	-18.83402100	1.74318300	-3.39018200
C	-16.36709600	1.41840100	-2.01645800
C	-17.29443800	1.53893500	-0.78299700
H	-16.85200300	2.19795100	-0.01794500
H	-18.26019500	1.95436600	-1.09753200
H	-17.46175900	0.54746900	-0.33474300
C	-16.10188700	2.81749200	-2.62287400
H	-17.05168600	3.24883800	-2.96331900
H	-15.64895700	3.49108300	-1.87698800
H	-15.41582800	2.74345600	-3.48111800
C	-15.04463500	0.81986200	-1.57189900
N	-14.58866300	-0.35563200	-2.09118400
N	-13.44020200	-0.68683700	-1.56834300
C	-14.09164100	1.23263100	-0.65800400
H	-14.02405100	2.09859200	-0.00581800
N	-13.13082200	0.26838400	-0.69813300
N	-16.92177700	0.48530900	-3.01410900
Fe	-16.00044700	-1.24379200	-3.44858300
P	-16.70313200	-6.23209000	-6.96079300
P	-17.84596300	-4.54184800	-9.92316900

C	-17.04622900	-7.01634300	-8.62795600
H	-17.29701600	-8.08455000	-8.51578500
H	-16.09117100	-6.97828300	-9.17790400
C	-18.15766900	-6.33179300	-9.43960800
H	-19.09405500	-6.30653700	-8.85726600
H	-18.38357300	-6.92551500	-10.34192800
C	-18.21484200	-6.71406600	-5.98394300
C	-18.70859100	-5.78065100	-5.05690200
C	-18.86964100	-7.95226300	-6.10840300
C	-19.82917000	-6.07998300	-4.27303100
H	-18.20423600	-4.81948500	-4.93017300
C	-19.99396900	-8.24646000	-5.33386600
H	-18.49619500	-8.70532300	-6.80727700
C	-20.47678900	-7.30926500	-4.41296500
H	-20.19232200	-5.34369000	-3.55126500
H	-20.49188700	-9.21387000	-5.44366500
H	-21.35452300	-7.54169300	-3.80380300
C	-15.44155100	-7.39531600	-6.24561600
C	-14.93418900	-7.06235000	-4.97387500
C	-14.92514800	-8.52966000	-6.89417400
C	-13.96093000	-7.85472500	-4.36274200
H	-15.30152500	-6.16995300	-4.45764300
C	-13.93928400	-9.31546400	-6.28538400
H	-15.28554200	-8.81865400	-7.88338100
C	-13.45746900	-8.98461100	-5.01727500
H	-13.58756900	-7.58162300	-3.37208900
H	-13.55029500	-10.19398000	-6.80785700
H	-12.68972100	-9.60092300	-4.54197000
C	-16.52616800	-4.68081800	-11.22295900
C	-15.85585700	-3.49183900	-11.56617300
C	-16.13617200	-5.86987400	-11.86384400
C	-14.84650700	-3.48484700	-12.53156600
H	-16.13167800	-2.55869500	-11.06612600
C	-15.11627300	-5.86790300	-12.82194300
H	-16.62895600	-6.81442500	-11.62345700
C	-14.47110300	-4.67586700	-13.16130800
H	-14.34462300	-2.54764400	-12.78652500
H	-14.82700700	-6.80445300	-13.30642100
H	-13.67481100	-4.67548900	-13.91012300
C	-19.37129100	-4.25759100	-10.95323400
C	-19.54631700	-4.74819000	-12.25888000
C	-20.41076000	-3.50986900	-10.37317300
C	-20.73198200	-4.50685000	-12.95822500
H	-18.74738200	-5.31778000	-12.73966300
C	-21.60109200	-3.27461500	-11.06863800
H	-20.28386600	-3.09940400	-9.36755100
C	-21.76377200	-3.77248900	-12.36408000
H	-20.85007300	-4.89288000	-13.97445200
H	-22.39831800	-2.69224800	-10.59921600
H	-22.68987400	-3.58347700	-12.91332800
Cl	-16.05863600	-3.47960000	-3.30592400

C	-14.56383700	-1.41473600	-5.87364000
C	-15.32082500	-0.31517000	-5.61016900
H	-14.86557800	0.45698300	-4.98054400
C	-16.65463700	0.06221500	-6.24460100
H	-16.80056600	1.14220000	-6.09127900
H	-16.59097200	-0.08793000	-7.33445700
C	-11.89950100	0.18250600	0.06831100
H	-11.26884700	1.06334800	-0.12081900
H	-12.12129100	0.11469800	1.14346100
H	-11.37425600	-0.72372400	-0.25552100
C	-14.98421300	-2.51899400	-6.80200300
H	-15.75207700	-2.21224500	-7.52326700
H	-14.11584200	-2.90190400	-7.36126300
H	-15.39361400	-3.36814800	-6.22914300
C	-13.21309600	-1.60508700	-5.23001800
H	-13.19631400	-2.54912400	-4.65957300
H	-12.43278400	-1.68561300	-6.00679000
H	-12.94615600	-0.78618100	-4.54763800

UB3LYP-D3/def2-SVP-SMD(THF)//UB3LYP/def2-SVP(gas)
HF= -4404.2348652
UPBEPBE-D3/def2-SVP-SMD(THF)//UB3LYP/def2-SVP(gas)
HF= -4400.8355613

^{1}E'-noligand-dimethyl

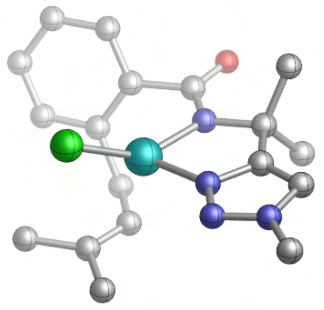

Zero-point correction= 0.386655 (Hartree/Particle)
Thermal correction to Energy= 0.413021
Thermal correction to Enthalpy= 0.413965
Thermal correction to Gibbs Free Energy= 0.329342
Sum of electronic and zero-point Energies= -2716.578925
Sum of electronic and thermal Energies= -2716.552560
Sum of electronic and thermal Enthalpies= -2716.551616
Sum of electronic and thermal Free Energies= -2716.636239

C	-18.10943000	-1.02233400	-5.98620300
C	-18.61317400	-0.36745100	-4.83851700
C	-19.85262400	-0.75942100	-4.30280500
C	-20.58470200	-1.80425400	-4.86107200
C	-20.09676700	-2.45160100	-5.99953500
C	-18.88194200	-2.05096100	-6.55402300
H	-20.24181500	-0.21606300	-3.43864800
H	-21.53906900	-2.10275600	-4.42017300
H	-20.66695300	-3.26044200	-6.46344900
H	-18.52761600	-2.53911900	-7.46579200
C	-17.95137900	0.83644400	-4.18527500
O	-18.53977700	1.91858200	-4.18118500
C	-16.06885700	1.61050600	-2.82461300
C	-16.92996800	2.11677900	-1.64257300
H	-16.37370600	2.85273500	-1.03906100
H	-17.84002500	2.58877600	-2.03326800
H	-17.21575600	1.27639600	-0.99151500
C	-15.64445900	2.79363600	-3.72474300
H	-16.54258000	3.26077600	-4.14882100
H	-15.08632700	3.54862900	-3.14697300
H	-15.00224800	2.44340500	-4.54775600
C	-14.83798800	0.92724100	-2.27186400
N	-14.62775900	-0.39400800	-2.54478100
N	-13.53409100	-0.82974000	-1.98135100
C	-13.78180500	1.32384400	-1.47516300
H	-13.52471800	2.27198400	-1.01172900
N	-13.01554100	0.20455000	-1.33246100
N	-16.76274600	0.55606600	-3.59614600
Fe	-16.03621100	-1.23913600	-3.61771500
Cl	-16.21708300	-3.34748600	-2.90762200
C	-14.96074900	-2.25247600	-5.93748600
C	-15.50078200	-1.00432000	-5.99983100

H	-14.85954300	-0.17370900	-5.68663700
C	-16.81018500	-0.59696500	-6.66551700
H	-16.80737700	0.49984500	-6.75746600
H	-16.82468000	-0.98835400	-7.69658600
C	-11.77982900	0.04507800	-0.58405900
H	-11.46021600	-0.99750100	-0.69626900
H	-11.00350400	0.71575900	-0.98032700
H	-11.94789100	0.26621600	0.47996900
C	-15.65066200	-3.49625200	-6.42720000
H	-16.45203700	-3.28863500	-7.14651400
H	-14.92306700	-4.17585300	-6.89920200
H	-16.08869900	-4.02740300	-5.56410400
C	-13.58614400	-2.47880300	-5.35886600
H	-13.64528800	-3.18887300	-4.51773600
H	-12.92552100	-2.92585500	-6.12244900
H	-13.12059100	-1.55082800	-4.99717600

UB3LYP-D3/def2-SVP-SMD(THF)//UB3LYP/def2-SVP(gas)
HF= -2717.0406983
UPBEPBE-D3/def2-SVP-SMD(THF)//UB3LYP/def2-SVP(gas)
HF= -2715.3314814

[3]E’-noligand-dimethyl

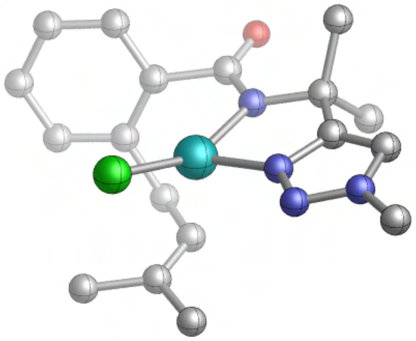

Zero-point correction= 0.386626 (Hartree/Particle)
Thermal correction to Energy= 0.412995
Thermal correction to Enthalpy= 0.413939
Thermal correction to Gibbs Free Energy= 0.328358
Sum of electronic and zero-point Energies= -2716.593593
Sum of electronic and thermal Energies= -2716.567223
Sum of electronic and thermal Enthalpies= -2716.566279
Sum of electronic and thermal Free Energies= -2716.651861

C	-18.14162700	-1.04766200	-5.97402300
C	-18.60444600	-0.36195900	-4.82779700
C	-19.81235900	-0.75366700	-4.22342200
C	-20.55066800	-1.82745000	-4.71492500
C	-20.09989700	-2.50872100	-5.84926200
C	-18.91667800	-2.11050500	-6.47014600
H	-20.17256500	-0.18958500	-3.35965300
H	-21.48045900	-2.12476000	-4.22360800
H	-20.67471500	-3.34394400	-6.25727300
H	-18.59019300	-2.62791700	-7.37615700
C	-17.91988700	0.86519600	-4.24300400
O	-18.47574900	1.96213000	-4.30210100
C	-16.03676800	1.62560900	-2.86595100
C	-16.91384400	2.20133700	-1.72849500
H	-16.34823500	2.93379300	-1.12960400
H	-17.79240200	2.69516500	-2.16226400
H	-17.25227000	1.39290200	-1.06240400
C	-15.54017000	2.76298800	-3.78736500
H	-16.40684600	3.24739100	-4.25566000
H	-14.97444700	3.51690100	-3.21557000
H	-14.88461300	2.36551400	-4.57768300
C	-14.85472800	0.90745800	-2.25481600
N	-14.69722600	-0.43021500	-2.48544600
N	-13.64051900	-0.89800500	-1.87873100
C	-13.80472600	1.27869500	-1.43856900
H	-13.51793900	2.22738600	-0.99403700
N	-13.09429200	0.13065500	-1.24350400
N	-16.74917400	0.57601000	-3.62606100
Fe	-16.08082100	-1.23692000	-3.60469600
Cl	-16.12744200	-3.38281200	-2.99744800
C	-14.95465300	-2.19053700	-6.00206600

C	-15.54854200	-0.96923100	-6.05630400
H	-14.95272700	-0.11333900	-5.72364200
C	-16.87740400	-0.62301400	-6.71394800
H	-16.90653900	0.46828800	-6.85681100
H	-16.90948600	-1.06446100	-7.72361700
C	-11.88822800	-0.06300400	-0.45590500
H	-11.60937200	-1.12002300	-0.53765300
H	-11.07374700	0.56639200	-0.84314200
H	-12.07686600	0.18775200	0.59810700
C	-15.58600800	-3.45950100	-6.50496200
H	-16.42639400	-3.28242300	-7.18737800
H	-14.83726700	-4.07927100	-7.02461200
H	-15.95715100	-4.04029300	-5.64253400
C	-13.57248000	-2.35905200	-5.42193500
H	-13.60108400	-3.07010400	-4.58018200
H	-12.89135200	-2.77610200	-6.18446700
H	-13.14887400	-1.41043600	-5.06155700

UB3LYP-D3/def2-SVP-SMD(THF)//UB3LYP/def2-SVP(gas)
HF= -2717.0849375
UPBEPBE-D3/def2-SVP-SMD(THF)//UB3LYP/def2-SVP(gas)
HF= -2715.3716533

^{5}E'-noligand-dimethyl

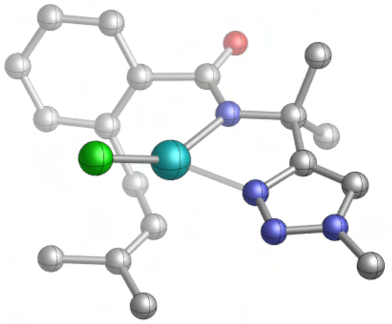

Zero-point correction= 0.386437 (Hartree/Particle)
Thermal correction to Energy= 0.412884
Thermal correction to Enthalpy= 0.413828
Thermal correction to Gibbs Free Energy= 0.327891
Sum of electronic and zero-point Energies= -2716.626420
Sum of electronic and thermal Energies= -2716.599972
Sum of electronic and thermal Enthalpies= -2716.599028
Sum of electronic and thermal Free Energies= -2716.684965

C	-18.40319400	-1.01076200	-5.89041900
C	-18.88329500	-0.33761700	-4.74133100
C	-20.14889500	-0.66364900	-4.22345400
C	-20.93061900	-1.66457800	-4.79455000
C	-20.46450200	-2.33353100	-5.92935100
C	-19.22419100	-1.99551400	-6.46897900
H	-20.51626400	-0.10343800	-3.36053800
H	-21.90494500	-1.91242500	-4.36607800
H	-21.07149700	-3.10895700	-6.40348600
H	-18.88910100	-2.49707400	-7.38062100
C	-18.15341500	0.80951500	-4.05655800
O	-18.68734900	1.91921900	-4.01675700
C	-16.17793700	1.45569300	-2.77056500
C	-16.94453000	1.98522900	-1.53408100
H	-16.32799200	2.69785200	-0.96189800
H	-17.85979600	2.49101400	-1.86647000
H	-17.21913100	1.15122400	-0.86995000
C	-15.76236400	2.63362900	-3.68415900
H	-16.66334600	3.14450400	-4.04655500
H	-15.13657200	3.35621800	-3.13517500
H	-15.18753600	2.26793200	-4.54928200
C	-14.92617900	0.74083800	-2.29397200
N	-14.72360900	-0.58235200	-2.55365300
N	-13.59326000	-0.99333500	-2.04829900
C	-13.82610700	1.16405300	-1.56978500
H	-13.55081600	2.12454900	-1.14376700
N	-13.04254400	0.05685800	-1.44883200
N	-16.96977900	0.44872000	-3.50192500
Fe	-16.40104000	-1.47328400	-3.55916200
Cl	-16.80352300	-3.57674100	-2.93976900
C	-15.31030200	-2.43879800	-5.96099900
C	-15.79056700	-1.16669000	-5.94679400

H	-15.11823300	-0.39221000	-5.56250700
C	-17.08215600	-0.65194500	-6.57449700
H	-17.00582500	0.44426500	-6.63285300
H	-17.14037300	-1.00987900	-7.61584700
C	-11.76424200	-0.07985400	-0.77185000
H	-11.44310500	-1.12200000	-0.88493600
H	-11.01725900	0.58877300	-1.22415800
H	-11.87020200	0.15847800	0.29655300
C	-16.06247100	-3.61246800	-6.52790500
H	-16.82631400	-3.31888500	-7.25874800
H	-15.36798600	-4.31787600	-7.01124200
H	-16.56359700	-4.15204800	-5.70595300
C	-13.96044100	-2.77320400	-5.37726800
H	-14.07215100	-3.52596800	-4.57874800
H	-13.31145000	-3.21627600	-6.15286600
H	-13.45195100	-1.89598300	-4.95371600

UB3LYP-D3/def2-SVP-SMD(THF)//UB3LYP/def2-SVP(gas)
HF= -2717.1152968
UPBEPBE-D3/def2-SVP-SMD(THF)//UB3LYP/def2-SVP(gas)
HF= -2715.3894765

[1]E''-dimethyl

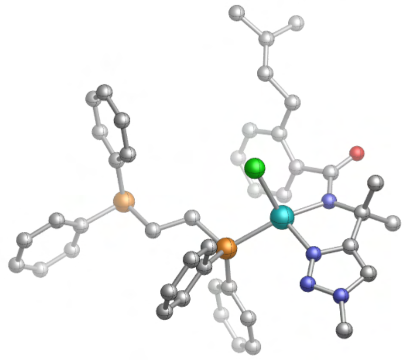

Zero-point correction= 0.810377 (Hartree/Particle)
Thermal correction to Energy= 0.864136
Thermal correction to Enthalpy= 0.865081
Thermal correction to Gibbs Free Energy= 0.711726
Sum of electronic and zero-point Energies= -4403.212136
Sum of electronic and thermal Energies= -4403.158377
Sum of electronic and thermal Enthalpies= -4403.157433
Sum of electronic and thermal Free Energies= -4403.310788

C	-18.84646200	0.18098800	-2.67381200
C	-18.67167700	1.58497700	-2.70189400
C	-19.35543000	2.35641000	-3.65580500
C	-20.23043500	1.76232900	-4.57241500
C	-20.40648300	0.37880200	-4.54770400
C	-19.71323700	-0.39616400	-3.61058200
H	-19.22127200	3.44087800	-3.65352200
H	-20.77326800	2.38084700	-5.29224400
H	-21.08139800	-0.10460300	-5.25921600
H	-19.84626900	-1.48026400	-3.60386800
C	-17.82721600	2.29837800	-1.66236900
O	-18.08175200	2.16147800	-0.46401100
C	-16.05567600	3.95494200	-1.32126600
C	-15.17625200	3.12085000	-0.35584900
H	-14.51475300	3.77050300	0.24081100
H	-15.82937800	2.55227400	0.31845300
H	-14.55839700	2.41179400	-0.92761500
C	-16.92560500	4.95913500	-0.53470600
H	-17.59754100	4.40846000	0.13526800
H	-16.29786400	5.63570200	0.06889700
H	-17.53152300	5.56626500	-1.22497500
C	-15.15150400	4.70825600	-2.27092300
N	-15.06725900	4.30371400	-3.57214900
N	-14.22621400	5.04333000	-4.24447800
C	-14.28585000	5.77729900	-2.14596100
H	-14.01803800	6.41272600	-1.30679500
N	-13.74566900	5.93545800	-3.38807800
N	-16.86022200	3.08796300	-2.20219600
Fe	-16.16726400	2.69561700	-3.97642600
P	-16.29003700	2.72668800	-6.34980400
C	-17.11127400	0.91344300	-8.46370600

H	-17.42942800	1.78737500	-9.05581600
H	-16.08197800	0.68712800	-8.78415000
C	-17.17199000	1.20972300	-6.96361200
H	-16.72491100	0.40107700	-6.36453800
H	-18.20978200	1.29196900	-6.59982500
C	-14.68106300	2.73540800	-7.26057500
C	-14.51887500	3.34812000	-8.51528900
C	-13.58672500	2.07088700	-6.67816000
C	-13.28668500	3.29864500	-9.17344200
H	-15.35440200	3.87129700	-8.98485600
C	-12.35839900	2.01981500	-7.34379000
H	-13.70613000	1.58176600	-5.70770200
C	-12.20359700	2.63462000	-8.58962200
H	-13.17552600	3.77882500	-10.14927400
H	-11.51718800	1.49697000	-6.88109400
H	-11.24099900	2.59581800	-9.10648200
C	-17.21374800	4.17171400	-7.02545000
C	-18.51730300	4.06600300	-7.53941200
C	-16.63208100	5.45035700	-6.92160500
C	-19.21532900	5.20657000	-7.95202800
H	-19.00477700	3.09289300	-7.61891900
C	-17.32899200	6.58541400	-7.33926600
H	-15.62668800	5.55396200	-6.50560000
C	-18.62364900	6.46776800	-7.85706000
H	-20.22771800	5.10371500	-8.35101300
H	-16.86053000	7.56980800	-7.25638900
H	-19.16947400	7.35728600	-8.18164400
Cl	-15.37788500	0.54571700	-3.94917000
C	-12.78399400	6.92905200	-3.83448100
H	-13.24026000	7.93028700	-3.84746500
H	-11.91095500	6.93394700	-3.16666200
H	-12.46937100	6.65438400	-4.84817900
P	-18.29931800	-0.44489500	-8.99273600
C	-17.91146200	-0.47376300	-10.81201000
C	-18.76725200	0.24675600	-11.66383200
C	-16.82263100	-1.15920900	-11.37780700
C	-18.53151500	0.29976100	-13.04093300
H	-19.63346700	0.76587900	-11.24227500
C	-16.59144100	-1.11446400	-12.75563500
H	-16.15207900	-1.73783000	-10.73796500
C	-17.44252000	-0.38298400	-13.59043100
H	-19.20712200	0.86653400	-13.68730400
H	-15.74115700	-1.65551700	-13.17983300
H	-17.26084900	-0.35111800	-14.66796600
C	-17.48756700	-1.99910600	-8.39613700
C	-18.24865400	-3.17983300	-8.49478000
C	-16.20883700	-2.07799800	-7.81916900
C	-17.74067700	-4.40278600	-8.05346600
H	-19.25569300	-3.13648700	-8.92075200
C	-15.70570900	-3.30053100	-7.35957800
H	-15.58805000	-1.18632200	-7.71397800

C	-16.46617600	-4.46589300	-7.47958400
H	-18.34684600	-5.30795900	-8.14544700
H	-14.71319100	-3.33659700	-6.90311300
H	-16.07130300	-5.41958800	-7.11993400
C	-18.46834300	-3.19490600	-1.36935300
C	-17.97304600	-2.12372000	-2.01681600
H	-17.37678700	-2.30770200	-2.91972400
C	-18.10216200	-0.67068100	-1.65471900
H	-17.08511800	-0.25503700	-1.55947500
H	-18.57269600	-0.52227000	-0.67086700
C	-18.19542300	-4.59414000	-1.86692600
H	-17.66485100	-5.19149800	-1.10286600
H	-19.13705400	-5.13271700	-2.07962500
H	-17.58660800	-4.59355000	-2.78331900
C	-19.31148900	-3.12598400	-0.12034000
H	-19.50805700	-2.09820700	0.21202900
H	-20.28519500	-3.62333700	-0.27993000
H	-18.82306600	-3.66436700	0.71205600

UB3LYP-D3/def2-SVP-SMD(THF)//UB3LYP/def2-SVP(gas)
HF= -4404.1945306
UPBEPBE-D3/def2-SVP-SMD(THF)//UB3LYP/def2-SVP(gas)
HF= -4400.8149945

^{3}E’’-dimethyl

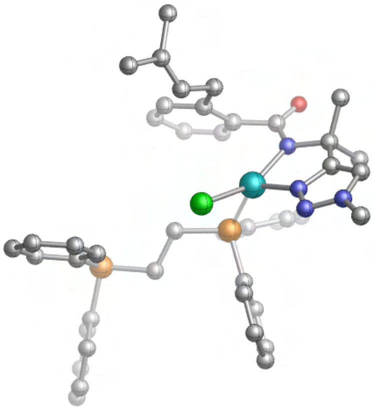

Zero-point correction= 0.810496 (Hartree/Particle)
Thermal correction to Energy= 0.864209
Thermal correction to Enthalpy= 0.865153
Thermal correction to Gibbs Free Energy= 0.712045
Sum of electronic and zero-point Energies= -4403.221120
Sum of electronic and thermal Energies= -4403.167407
Sum of electronic and thermal Enthalpies= -4403.166463
Sum of electronic and thermal Free Energies= -4403.319571

C	-18.85260000	0.75540100	-2.89550800
C	-18.98787300	2.12204600	-3.24997400
C	-19.99907100	2.50440800	-4.14926100
C	-20.82087700	1.56097500	-4.76352000
C	-20.65390400	0.20655200	-4.46039300
C	-19.69015500	-0.17984900	-3.52869800
H	-20.13213500	3.56716300	-4.35848200
H	-21.59098500	1.88183300	-5.46976300
H	-21.28660000	-0.54860500	-4.93450200
H	-19.58745600	-1.23434900	-3.26283200
C	-18.20220900	3.24816000	-2.59760700
O	-18.82371200	4.11403300	-1.97779300
C	-16.02301200	4.10435700	-1.90867000
C	-16.34068200	3.95837700	-0.40062600
H	-15.65734200	4.58168500	0.19899000
H	-17.37194100	4.27527400	-0.20647600
H	-16.22274000	2.91174700	-0.08112400
C	-16.16674400	5.58471000	-2.33666400
H	-17.22013600	5.88005500	-2.24946900
H	-15.55418600	6.24074600	-1.69641500
H	-15.83354400	5.72149500	-3.37702800
C	-14.59620800	3.66482700	-2.13744500
N	-14.33007300	2.72304600	-3.08539900
N	-13.05242200	2.46651300	-3.15654900
C	-13.37712500	4.01142600	-1.58758300
H	-13.09206100	4.70773400	-0.80430900
N	-12.46848700	3.24359900	-2.25513700
N	-16.84734900	3.19605100	-2.74786900
Fe	-15.94450400	2.03737900	-4.02848000

P	-16.82942000	2.50682000	-6.25186300
C	-17.27228100	0.75259300	-8.53094200
H	-17.57118800	1.62498300	-9.13474300
H	-16.19334100	0.61899600	-8.70771600
C	-17.56150100	0.97975400	-7.04473400
H	-17.16265700	0.15635400	-6.43646400
H	-18.64284100	1.01861000	-6.83617900
C	-15.41293900	3.01261000	-7.34553400
C	-15.56319000	3.97582100	-8.35898000
C	-14.15132600	2.41726700	-7.15993400
C	-14.48133500	4.33097400	-9.17003000
H	-16.52918900	4.45684500	-8.52044400
C	-13.07447200	2.77238700	-7.97734100
H	-14.01314000	1.66622800	-6.37890700
C	-13.23392800	3.72978400	-8.98316000
H	-14.61835400	5.08092900	-9.95355900
H	-12.10308300	2.29584900	-7.82106700
H	-12.38903300	4.00785200	-9.61876700
C	-18.05279200	3.85406000	-6.55320800
C	-19.13240100	3.73517800	-7.44409700
C	-17.89140300	5.05929100	-5.84892000
C	-20.02354000	4.79740400	-7.62777100
H	-19.29520800	2.81067600	-8.00017700
C	-18.77974100	6.12096600	-6.03391300
H	-17.07280600	5.16200700	-5.13468600
C	-19.85005100	5.99261200	-6.92452800
H	-20.85930300	4.68633000	-8.32344700
H	-18.64190200	7.04663300	-5.46969700
H	-20.55041900	6.81975100	-7.06540200
Cl	-15.10613300	0.04125800	-4.72194200
C	-11.02709600	3.19136600	-2.07822300
H	-10.57995900	4.17373100	-2.28940900
H	-10.78009800	2.88886600	-1.05017400
H	-10.63679300	2.44856100	-2.78366600
P	-18.24896800	-0.67603900	-9.26695600
C	-17.69161800	-0.52206200	-11.03536200
C	-18.59594100	0.03848000	-11.95356200
C	-16.42228700	-0.91450900	-11.49527300
C	-18.23895300	0.21773800	-13.29401800
H	-19.59376900	0.33117900	-11.61327800
C	-16.06652700	-0.74130100	-12.83536600
H	-15.70822600	-1.36539200	-10.80146800
C	-16.97271000	-0.17304900	-13.73752900
H	-18.95535600	0.65535500	-13.99457000
H	-15.07602400	-1.05283200	-13.17814300
H	-16.69305900	-0.04088200	-14.78611700
C	-17.32186200	-2.18064400	-8.71053800
C	-17.81672400	-3.42345200	-9.15330600
C	-16.22151000	-2.17524700	-7.83690700
C	-17.21667300	-4.61925800	-8.75736200
H	-18.68502200	-3.45229100	-9.81886300

C	-15.63177000	-3.37648500	-7.42440100
H	-15.80948000	-1.24361200	-7.44637000
C	-16.12106100	-4.59961800	-7.88621100
H	-17.61242600	-5.57155800	-9.12078600
H	-14.78655800	-3.34336200	-6.73216100
H	-15.65772200	-5.53581300	-7.56386800
C	-17.64760700	-2.16710500	-1.06832600
C	-17.42994900	-1.11968600	-1.88580800
H	-16.78125400	-1.27685400	-2.75559100
C	-17.94978400	0.28659200	-1.75493200
H	-17.09444200	0.97340100	-1.67110100
H	-18.51672500	0.41187800	-0.81653200
C	-17.00319300	-3.50447700	-1.34509300
H	-16.36541100	-3.82064900	-0.49930800
H	-17.76507100	-4.29556300	-1.46888600
H	-16.38174500	-3.48132900	-2.25215800
C	-18.51247400	-2.13522700	0.16771500
H	-17.91659900	-2.37030100	1.06833400
H	-19.00401300	-1.16712500	0.33357300
H	-19.30014000	-2.90770900	0.11048400

UB3LYP-D3/def2-SVP-SMD(THF)//UB3LYP/def2-SVP(gas)
HF= -4404.2314006
UPBEPBE-D3/def2-SVP-SMD(THF)//UB3LYP/def2-SVP(gas)
HF= -4400.8441398

^{5}E''-dimethyl

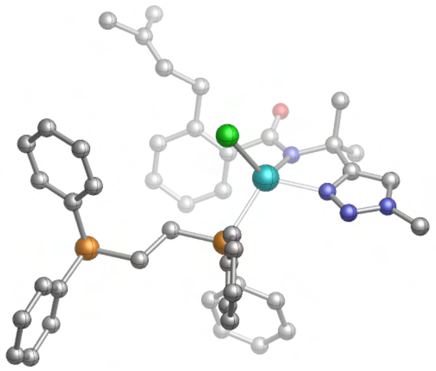

Zero-point correction= 0.810002 (Hartree/Particle)
Thermal correction to Energy= 0.863979
Thermal correction to Enthalpy= 0.864923
Thermal correction to Gibbs Free Energy= 0.710124
Sum of electronic and zero-point Energies= -4403.249782
Sum of electronic and thermal Energies= -4403.195805
Sum of electronic and thermal Enthalpies= -4403.194861
Sum of electronic and thermal Free Energies= -4403.349660

C	-18.85547000	0.30863700	-2.92660700
C	-18.71261300	1.71516500	-2.91146100
C	-19.42022000	2.50049800	-3.83356800
C	-20.29159000	1.91540700	-4.75777300
C	-20.43664100	0.52649100	-4.77814100
C	-19.71474300	-0.26222200	-3.87585800
H	-19.29390700	3.58610400	-3.80994000
H	-20.85404100	2.54349400	-5.45384800
H	-21.10840900	0.05195200	-5.49844200
H	-19.82371600	-1.34885300	-3.90003400
C	-17.91325100	2.41047900	-1.82186200
O	-18.32275300	2.33536500	-0.65926600
C	-16.08396900	3.92349900	-1.28707100
C	-15.41101200	3.06150400	-0.18795400
H	-14.81555000	3.68430200	0.50004600
H	-16.19256900	2.53976200	0.37889600
H	-14.74732400	2.31200600	-0.64655100
C	-16.98747600	5.00190800	-0.64842300
H	-17.78180600	4.51481800	-0.07030600
H	-16.40750900	5.65433800	0.02553800
H	-17.44767700	5.62650100	-1.42973200
C	-14.99252500	4.61818200	-2.08156200
N	-14.65083000	4.19201800	-3.33077100
N	-13.67401900	4.90425900	-3.82445700
C	-14.14745600	5.67542100	-1.79470100
H	-14.04741000	6.32165800	-0.92760700
N	-13.36292600	5.80594200	-2.90077500
N	-16.82110400	3.08832700	-2.25927600
Fe	-15.79321000	2.49404800	-3.89212200
P	-16.32031600	2.70598300	-6.37818900
C	-17.20166000	0.84168100	-8.41990300

H	-17.57288200	1.69343500	-9.01289300
H	-16.18194500	0.64061300	-8.78451200
C	-17.20067100	1.16272900	-6.92311100
H	-16.70806600	0.36776800	-6.34074200
H	-18.22087500	1.23329100	-6.51113400
C	-14.78683000	2.76240300	-7.40610600
C	-14.69730800	3.49731600	-8.60037400
C	-13.67750300	2.00803900	-6.98038300
C	-13.52077300	3.47857800	-9.35500500
H	-15.54694300	4.08966600	-8.94616700
C	-12.50667000	1.98808900	-7.74376000
H	-13.73227300	1.43047600	-6.05271300
C	-12.42380800	2.72355500	-8.92982900
H	-13.46465100	4.05458400	-10.28242700
H	-11.65286000	1.39704000	-7.40254500
H	-11.50514500	2.70947900	-9.52216300
C	-17.31511300	4.14737800	-6.93628900
C	-18.59490100	4.03228800	-7.50294100
C	-16.79373400	5.43361000	-6.69613700
C	-19.32865200	5.17661400	-7.83588300
H	-19.03600200	3.05067800	-7.68255600
C	-17.52555100	6.57246400	-7.03488000
H	-15.80643500	5.54325000	-6.23816400
C	-18.79648000	6.44724700	-7.60713700
H	-20.32308200	5.06948800	-8.27656100
H	-17.10501200	7.56344700	-6.84528200
H	-19.37171000	7.33903200	-7.86832300
Cl	-14.96455700	0.36938400	-3.91622300
C	-12.30288000	6.76746300	-3.15032100
H	-12.70276400	7.79207000	-3.13672200
H	-11.51523100	6.67217500	-2.38887500
H	-11.88548600	6.54743500	-4.13996900
P	-18.37072100	-0.56840000	-8.85384500
C	-18.02989500	-0.67565000	-10.67921200
C	-18.90973200	0.01160700	-11.53453600
C	-16.96301300	-1.39126600	-11.24886900
C	-18.71754400	0.00411400	-12.91911800
H	-19.76109600	0.55203200	-11.10931700
C	-16.77610100	-1.40787900	-12.63420500
H	-16.27419100	-1.94547300	-10.60708300
C	-17.64963100	-0.70818400	-13.47272400
H	-19.41106500	0.54616200	-13.56769900
H	-15.94255400	-1.97237700	-13.06098800
H	-17.50236600	-0.72417200	-14.55581500
C	-17.50186800	-2.06291400	-8.18760300
C	-18.27604000	-3.23118400	-8.05798700
C	-16.16030000	-2.10245000	-7.77005100
C	-17.72332700	-4.40743000	-7.54698400
H	-19.32849600	-3.21344400	-8.35652500
C	-15.60981300	-3.27508900	-7.24190300
H	-15.52749600	-1.21589900	-7.84655700

C	-16.38738800	-4.43091200	-7.13311000
H	-18.34129400	-5.30484300	-7.45839500
H	-14.56854100	-3.28036800	-6.90979900
H	-15.95605500	-5.34585300	-6.71883300
C	-18.44990200	-3.07883900	-1.67483700
C	-17.94773700	-1.99348600	-2.29270700
H	-17.32243700	-2.15862500	-3.17918500
C	-18.10522100	-0.54746200	-1.91440000
H	-17.09509000	-0.11860300	-1.80212600
H	-18.59130700	-0.42082300	-0.93474300
C	-18.14865600	-4.46756900	-2.18551000
H	-17.62660000	-5.06882000	-1.41858300
H	-19.07890600	-5.01466000	-2.42501300
H	-17.52125900	-4.44618600	-3.08900100
C	-19.32798500	-3.03714300	-0.44890600
H	-18.85983200	-3.58815900	0.38687800
H	-19.54013000	-2.01675300	-0.10353100
H	-20.29402100	-3.53646100	-0.64468000

UB3LYP-D3/def2-SVP-SMD(THF)//UB3LYP/def2-SVP(gas)
HF= -4404.2589331
UPBEPBE-D3/def2-SVP-SMD(THF)//UB3LYP/def2-SVP(gas)
HF= -4400.8592516

Figure S33
[1]D''-dimethyl'

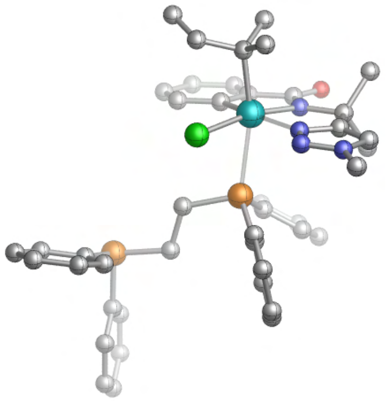

Zero-point correction= 0.810367 (Hartree/Particle)
Thermal correction to Energy= 0.862871
Thermal correction to Enthalpy= 0.863815
Thermal correction to Gibbs Free Energy= 0.718172
Sum of electronic and zero-point Energies= -4403.122299
Sum of electronic and thermal Energies= -4403.069795
Sum of electronic and thermal Enthalpies= -4403.068851
Sum of electronic and thermal Free Energies= -4403.214494

C	-18.44941700	1.01699900	-3.31722800
C	-19.28277100	2.10145300	-2.99778500
C	-20.67555400	2.02850600	-3.12682700
C	-21.25312000	0.85268800	-3.60630000
C	-20.43217500	-0.23495400	-3.94097100
C	-19.04047900	-0.16188900	-3.79111900
H	-21.27486400	2.90015700	-2.85181300
H	-22.33783800	0.77453700	-3.71766800
H	-20.88231400	-1.15934100	-4.31519600
H	-18.42225600	-1.02778400	-4.03540600
C	-18.59454200	3.32206400	-2.50297300
O	-19.16898400	4.34318800	-2.14085400
C	-16.34353200	4.28069300	-2.11147000
C	-16.52908300	4.68430000	-0.62693900
H	-15.89313900	5.55336900	-0.39362200
H	-17.57715200	4.95770700	-0.45167200
H	-16.24780400	3.86826900	0.05219200
C	-16.56744900	5.52543500	-3.00118500
H	-17.59109400	5.89630400	-2.87280000
H	-15.85812900	6.32058700	-2.71976300
H	-16.41225600	5.27830200	-4.05767300
C	-14.94111200	3.77615700	-2.32253700
N	-14.75688500	2.49468000	-2.73148700
N	-13.49023800	2.21695000	-2.84558600
C	-13.68238900	4.32809300	-2.17676500
H	-13.33722600	5.31236200	-1.87397000
N	-12.83012300	3.31933300	-2.51109100
N	-17.21718700	3.14591300	-2.50885200
Fe	-16.56506700	1.43593000	-3.02273100

P	-16.65292900	2.09907100	-5.67290900
C	-17.38204600	0.78320100	-8.17663000
H	-18.04131100	1.62906800	-8.43125900
H	-16.40916700	0.99325700	-8.64705800
C	-17.26228500	0.62533600	-6.66046800
H	-16.56710100	-0.18167200	-6.38651700
H	-18.22957500	0.34235100	-6.21941400
C	-15.07685100	2.49910500	-6.58350400
C	-15.03418800	3.23601000	-7.78328300
C	-13.87429400	1.95928300	-6.09090300
C	-13.82549200	3.44877400	-8.45264800
H	-15.95036600	3.64574600	-8.21013000
C	-12.66747000	2.16733700	-6.76768400
H	-13.88292700	1.35295200	-5.18502200
C	-12.63576000	2.91830600	-7.94538300
H	-13.81845700	4.02457500	-9.38198800
H	-11.74736700	1.73030800	-6.36975600
H	-11.69179200	3.08165800	-8.47225800
C	-17.83460300	3.43356900	-6.17382400
C	-19.20802000	3.13578700	-6.26161400
C	-17.43669000	4.76916600	-6.37061000
C	-20.14266100	4.13222400	-6.55488300
H	-19.56506100	2.11896900	-6.09100000
C	-18.37164600	5.76521400	-6.66510400
H	-16.38207700	5.04185700	-6.30395000
C	-19.72954100	5.45060800	-6.76097400
H	-21.20232400	3.87185000	-6.61477500
H	-18.03389600	6.79364300	-6.81812600
H	-20.46152300	6.22924500	-6.98957800
Cl	-15.49786500	-0.35463100	-3.92971700
C	-11.37968900	3.34819600	-2.59067400
H	-11.05337800	3.86942800	-3.50335100
H	-10.96799800	3.85711400	-1.70835500
H	-11.02648500	2.31049600	-2.61678800
P	-18.20566400	-0.69985800	-8.99053400
C	-18.19183300	-0.10815300	-10.75429000
C	-19.33749400	0.57103500	-11.20725900
C	-17.12602400	-0.29571500	-11.65068100
C	-19.40894900	1.06873100	-12.51127800
H	-20.18678300	0.70475200	-10.53015600
C	-17.20152300	0.19168900	-12.95914100
H	-16.23000300	-0.83030800	-11.32704000
C	-18.34020100	0.87791200	-13.39234700
H	-20.30691200	1.59677200	-12.84338900
H	-16.36413900	0.03382400	-13.64447200
H	-18.39725600	1.25717500	-14.41607100
C	-16.87387500	-1.98918300	-8.99728200
C	-17.27427600	-3.30347500	-9.30406500
C	-15.52198100	-1.76077500	-8.68830800
C	-16.35172300	-4.35148700	-9.32478600
H	-18.32650300	-3.50559900	-9.52585300

C	-14.59910500	-2.81266800	-8.69364700
H	-15.17195800	-0.75730900	-8.43779500
C	-15.00919800	-4.10873100	-9.01595100
H	-16.68400700	-5.36352000	-9.57106000
H	-13.55361000	-2.61456000	-8.44291300
H	-14.28694400	-4.92925800	-9.01898300
C	-18.06219000	-1.60011900	-1.01588800
H	-18.95692300	-1.09883300	-0.64010800
H	-18.12766500	-2.68505200	-1.13924100
C	-16.93503400	-0.94920600	-1.34020000
H	-16.10084700	-1.54149600	-1.72310300
C	-16.64112400	0.51256100	-1.16880000
C	-17.66635500	1.17354500	-0.24129200
H	-18.69160400	1.13309800	-0.62830700
H	-17.66048200	0.63777200	0.72534300
H	-17.42712700	2.22186200	-0.03939800
C	-15.23798900	0.63132600	-0.55254400
H	-14.46034900	0.21096700	-1.20517900
H	-14.97095500	1.66964000	-0.30901400
H	-15.22254200	0.06427100	0.39717600

UB3LYP-D3/def2-SVP-SMD(THF)//UB3LYP/def2-SVP(gas)
HF= -4404.1165424
UPBEPBE-D3/def2-SVP-SMD(THF)//UB3LYP/def2-SVP(gas)
HF= -4400.7758402

^{3}D''-dimethyl'

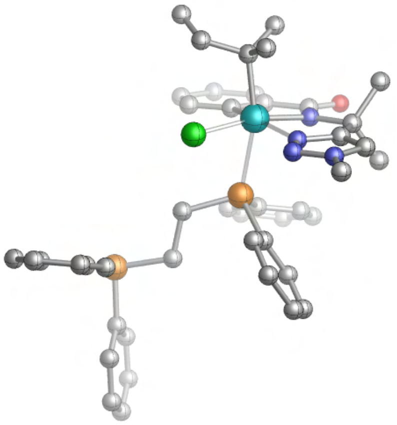

Zero-point correction=	0.810723 (Hartree/Particle)		
Thermal correction to Energy=	0.862109		
Thermal correction to Enthalpy=	0.863053		
Thermal correction to Gibbs Free Energy=	0.721145		
Sum of electronic and zero-point Energies=	-4403.144011		
Sum of electronic and thermal Energies=	-4403.092625		
Sum of electronic and thermal Enthalpies=	-4403.091681		
Sum of electronic and thermal Free Energies=	-4403.233589		
C	-18.32932500	1.19296100	-3.41830100
C	-19.00681100	2.37029300	-3.05796900
C	-20.40471200	2.42962300	-3.06580100
C	-21.13428700	1.30338000	-3.45256400
C	-20.46338700	0.13076700	-3.82784400
C	-19.06275300	0.06864400	-3.80894300
H	-20.89071500	3.36284600	-2.77047000
H	-22.22708300	1.33408300	-3.46480900
H	-21.03688300	-0.75142900	-4.12791100
H	-18.54979300	-0.85785000	-4.07562200
C	-18.16632900	3.54387000	-2.66808500
O	-18.62993200	4.62563600	-2.30902900
C	-15.80721500	4.26779400	-2.44110000
C	-15.84612900	4.68937400	-0.95110500
H	-15.09266000	5.46906900	-0.75457600
H	-16.83997300	5.09283800	-0.71732100
H	-15.63571900	3.84179100	-0.28532100
C	-15.92833500	5.54143200	-3.31575200
H	-16.93158600	5.97079100	-3.20727700
H	-15.18408700	6.28765000	-2.99436300
H	-15.72974600	5.31171200	-4.37175900
C	-14.48762600	3.61169100	-2.75822400
N	-14.47110300	2.34313600	-3.24810900
N	-13.25111200	1.94021200	-3.46349900
C	-13.16943700	4.01669900	-2.67426800
H	-12.69864100	4.93906900	-2.34686900
N	-12.45667500	2.94407300	-3.12113100
N	-16.82903300	3.24321400	-2.77109300
Fe	-16.37742800	1.43269000	-3.25151300

P	-16.78856100	1.94376500	-5.74159100
C	-17.14609000	0.64444100	-8.31361800
H	-17.80467000	1.48167300	-8.59394900
H	-16.14753600	0.90096400	-8.69975800
C	-17.12020400	0.43314400	-6.79655900
H	-16.34711700	-0.28831500	-6.50143000
H	-18.06534100	0.00432900	-6.42962600
C	-15.33431700	2.66313900	-6.65331200
C	-15.38575400	3.79784800	-7.48048400
C	-14.11159700	1.97269100	-6.53284200
C	-14.24392300	4.23880600	-8.15913100
H	-16.32328200	4.33748000	-7.61746300
C	-12.97718200	2.41066300	-7.22129800
H	-14.05059800	1.08609300	-5.89731800
C	-13.03625400	3.54859500	-8.03298100
H	-14.30746800	5.12175700	-8.80073200
H	-12.04098100	1.85392900	-7.12312800
H	-12.14813700	3.88997500	-8.57145100
C	-18.22259800	3.04099500	-6.16246700
C	-19.42397600	2.50627400	-6.66572300
C	-18.18473100	4.41950300	-5.88852600
C	-20.53133400	3.32395100	-6.90414200
H	-19.51929400	1.43939400	-6.86473200
C	-19.29120400	5.23801700	-6.12640900
H	-17.28681800	4.86654800	-5.46950900
C	-20.47035100	4.69393500	-6.63956000
H	-21.45106100	2.87936400	-7.29279300
H	-19.23079900	6.30430500	-5.89525700
H	-21.33908300	5.33131500	-6.82269400
Cl	-15.46961500	-0.51420700	-4.15287200
C	-11.01885500	2.82191800	-3.29170700
H	-10.50290200	3.17595100	-2.38844500
H	-10.79353700	1.76142700	-3.45491100
H	-10.68414800	3.40701500	-4.16138100
P	-17.87105500	-0.82842500	-9.23106300
C	-17.73404300	-0.18867900	-10.97206300
C	-18.83485900	0.52940000	-11.47420100
C	-16.62211500	-0.38126500	-11.80905700
C	-18.81710000	1.06077800	-12.76643700
H	-19.72010300	0.66760300	-10.84569700
C	-16.60879900	0.13950800	-13.10688100
H	-15.75924200	-0.94494700	-11.44738000
C	-17.70286100	0.86469600	-13.58819400
H	-19.68103100	1.61938300	-13.13661100
H	-15.73612400	-0.02249600	-13.74559900
H	-17.69009500	1.27046500	-14.60320400
C	-16.52304600	-2.09771700	-9.16319800
C	-16.88569800	-3.41833100	-9.48804900
C	-15.19282600	-1.84616700	-8.78571300
C	-15.94619100	-4.45121000	-9.45839800
H	-17.92163300	-3.63756900	-9.76349200

C	-14.25395500	-2.88249300	-8.74098600
H	-14.87423100	-0.83586300	-8.52083000
C	-14.62589600	-4.18573700	-9.08095000
H	-16.24856600	-5.46898600	-9.71901200
H	-13.22611300	-2.66771700	-8.43673400
H	-13.89116500	-4.99427700	-9.04430300
C	-18.07163400	-1.39038700	-1.05226600
H	-18.88763200	-0.80642300	-0.62067700
H	-18.22974300	-2.47061100	-1.12114600
C	-16.93193700	-0.84527200	-1.50334200
H	-16.18586700	-1.51399400	-1.93699500
C	-16.51946800	0.59690000	-1.42794600
C	-17.39933100	1.36724300	-0.43684900
H	-18.45544600	1.39358300	-0.73059900
H	-17.34450100	0.86326900	0.54456400
H	-17.06746100	2.40002400	-0.29884400
C	-15.05420300	0.62757100	-0.92889900
H	-14.36736600	0.10965900	-1.61167300
H	-14.68649300	1.64810200	-0.75346900
H	-15.02173600	0.10297000	0.04361000

UB3LYP-D3/def2-SVP-SMD(THF)//UB3LYP/def2-SVP(gas)
HF= -4404.1573973
UPBEPBE-D3/def2-SVP-SMD(THF)//UB3LYP/def2-SVP(gas)
HF= -4400.8119885

^{5}D''-dimethyl'

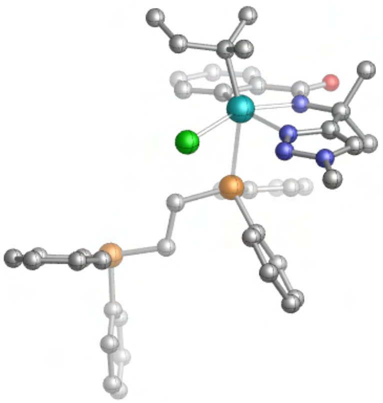

Zero-point correction= 0.808034 (Hartree/Particle)
Thermal correction to Energy= 0.859695
Thermal correction to Enthalpy= 0.860639
Thermal correction to Gibbs Free Energy= 0.717174
Sum of electronic and zero-point Energies= -4403.140884
Sum of electronic and thermal Energies= -4403.089223
Sum of electronic and thermal Enthalpies= -4403.088279
Sum of electronic and thermal Free Energies= -4403.231744

C	-18.42771400	1.25199200	-3.29762200
C	-18.98138200	2.49288000	-2.95806600
C	-20.36908900	2.67465900	-3.01682000
C	-21.19184300	1.62691400	-3.43568800
C	-20.62770900	0.39786100	-3.79999900
C	-19.24036200	0.20293400	-3.72497300
H	-20.76966900	3.65114400	-2.73414100
H	-22.27497400	1.76633900	-3.48531200
H	-21.26915000	-0.42198200	-4.13677800
H	-18.81213100	-0.76866900	-3.98317900
C	-18.07598200	3.62200400	-2.54508300
O	-18.53408500	4.70952100	-2.18189600
C	-15.73956300	4.29587100	-2.32125800
C	-15.78032000	4.68111300	-0.82053100
H	-14.98569300	5.40561900	-0.57799500
H	-16.75527700	5.13147800	-0.59271900
H	-15.64011600	3.79420100	-0.18432000
C	-15.85068800	5.57846200	-3.18292400
H	-16.83834500	6.03305000	-3.03983900
H	-15.07596700	6.30552200	-2.88918300
H	-15.70171200	5.34281900	-4.24689900
C	-14.41909500	3.63453200	-2.63721100
N	-14.38799800	2.34566500	-3.06389900
N	-13.16362000	1.95720100	-3.28678500
C	-13.10676500	4.06945200	-2.60059200
H	-12.64969300	5.01751500	-2.33221100
N	-12.38038100	2.99247800	-3.00978400
N	-16.75830100	3.28659500	-2.64612300
Fe	-16.38742200	1.28661300	-3.15223100

P	-16.89737500	1.98834600	-5.90628800
C	-17.19139500	0.60169100	-8.44472800
H	-17.82083700	1.44025300	-8.78363200
H	-16.17199900	0.82722900	-8.79512700
C	-17.23338400	0.45629600	-6.92020400
H	-16.49349900	-0.27351200	-6.56073400
H	-18.20840500	0.07525300	-6.57502800
C	-15.45652200	2.74689700	-6.79670600
C	-15.56511500	3.82228000	-7.69562800
C	-14.18560800	2.18393200	-6.56649400
C	-14.43266100	4.32531300	-8.34482300
H	-16.53869600	4.27034400	-7.90030600
C	-13.05959900	2.68316800	-7.22713700
H	-14.08123000	1.34822100	-5.87004600
C	-13.17681400	3.75775300	-8.11478200
H	-14.53829400	5.16085900	-9.04199100
H	-12.08310000	2.22574900	-7.04461900
H	-12.29441300	4.14841900	-8.62867200
C	-18.32246900	3.09868000	-6.28450100
C	-19.51423600	2.62518900	-6.86297300
C	-18.27370000	4.44554400	-5.87854500
C	-20.60825800	3.47589500	-7.04751600
H	-19.60888800	1.58351900	-7.17067400
C	-19.36520400	5.29526800	-6.06481700
H	-17.37649300	4.83664200	-5.39971400
C	-20.53818000	4.81365100	-6.65297600
H	-21.52270900	3.08376900	-7.50015900
H	-19.30138100	6.33435200	-5.73290400
H	-21.39587900	5.47607900	-6.79496400
Cl	-15.23905000	-0.45477600	-4.28788600
C	-10.94412800	2.88746400	-3.19916000
H	-10.41758200	3.22608700	-2.29560900
H	-10.71313400	1.83240000	-3.38800400
H	-10.62396000	3.49375300	-4.05972200
P	-17.90260400	-0.89048900	-9.34034800
C	-17.69000000	-0.31090800	-11.09497000
C	-18.79266400	0.32028200	-11.69818100
C	-16.51042500	-0.46037000	-11.84407300
C	-18.71409800	0.80824300	-13.00566800
H	-19.72656700	0.42379600	-11.13725500
C	-16.43457900	0.01761900	-13.15570400
H	-15.64404600	-0.95770900	-11.40180900
C	-17.53369100	0.65584300	-13.73904100
H	-19.58115000	1.29875100	-13.45629900
H	-15.50990500	-0.10935100	-13.72543000
H	-17.47220000	1.02800000	-14.76510700
C	-16.57533200	-2.17415900	-9.18719000
C	-16.92179800	-3.48446000	-9.56855800
C	-15.28102000	-1.94601900	-8.68999000
C	-15.99902100	-4.52833000	-9.47881200
H	-17.93165200	-3.68678200	-9.93812200

C	-14.36035500	-2.99462900	-8.58405800
H	-14.97510600	-0.94645100	-8.37528200
C	-14.71381200	-4.28609700	-8.98207700
H	-16.28766300	-5.53703600	-9.78623600
H	-13.36204500	-2.79725000	-8.18484000
H	-13.99337800	-5.10392100	-8.89804900
C	-18.15638300	-1.77145900	-1.33478000
H	-18.98418100	-1.28024400	-0.81834300
H	-18.27395900	-2.83840500	-1.54460700
C	-17.04112800	-1.12636500	-1.71294500
H	-16.26915500	-1.69284200	-2.23996400
C	-16.70279400	0.30442400	-1.45464100
C	-17.62094400	0.98121400	-0.43722300
H	-18.68018900	0.92572400	-0.71324900
H	-17.49961700	0.47598000	0.53827200
H	-17.36414500	2.03924000	-0.30134600
C	-15.23832700	0.38604700	-0.94707000
H	-14.50964800	-0.03462800	-1.65231400
H	-14.94197300	1.40981000	-0.68201100
H	-15.19566200	-0.21594300	-0.02123500

UB3LYP-D3/def2-SVP-SMD(THF)//UB3LYP/def2-SVP(gas)
HF= -4404.1593820
UPBEPBE-D3/def2-SVP-SMD(THF)//UB3LYP/def2-SVP(gas)
HF= -4400.7895327

[1]D''-TS-dimethyl'

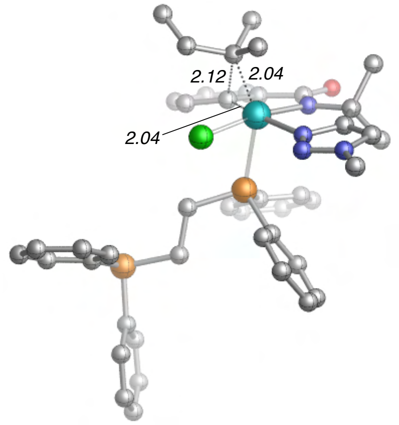

Zero-point correction= 0.809970 (Hartree/Particle)
Thermal correction to Energy= 0.860831
Thermal correction to Enthalpy= 0.861775
Thermal correction to Gibbs Free Energy= 0.722316
Sum of electronic and zero-point Energies= -4403.108023
Sum of electronic and thermal Energies= -4403.057162
Sum of electronic and thermal Enthalpies= -4403.056218
Sum of electronic and thermal Free Energies= -4403.195677

C	-18.35280100	1.13877200	-3.33633100
C	-19.02688800	2.34275100	-3.04900600
C	-20.40663700	2.45856600	-3.23672800
C	-21.13157400	1.38132800	-3.75123700
C	-20.45927600	0.20238900	-4.09536000
C	-19.07896800	0.07721100	-3.88774600
H	-20.87996500	3.41131000	-2.98729800
H	-22.21128700	1.46225200	-3.90067000
H	-21.01322300	-0.63874800	-4.52246500
H	-18.57801200	-0.85643200	-4.14575700
C	-18.21258400	3.50615900	-2.57455800
O	-18.71719500	4.55855100	-2.17264800
C	-15.88618400	4.26583000	-2.29750800
C	-15.92295500	4.54710800	-0.77499800
H	-15.15265600	5.28213200	-0.48974000
H	-16.91072100	4.95133300	-0.51548400
H	-15.75303700	3.62989300	-0.19271900
C	-16.05193400	5.60925200	-3.05336400
H	-17.06693800	5.99468600	-2.89945300
H	-15.32573200	6.34622600	-2.67379800
H	-15.85990800	5.48206600	-4.12827400
C	-14.55997900	3.67040000	-2.69916000
N	-14.53302700	2.45507000	-3.31779200
N	-13.30999000	2.10189100	-3.59872900
C	-13.24820800	4.09201700	-2.60911400
H	-12.78751700	4.98846600	-2.20438200
N	-12.52734500	3.08585800	-3.18022800
N	-16.87920300	3.23789600	-2.67709600
Fe	-16.33940000	1.47937400	-3.31303100

P	-16.83564600	2.00931700	-5.78265000
C	-17.09556400	0.66090000	-8.34115400
H	-17.61323200	1.57063300	-8.68498000
H	-16.04862600	0.76991600	-8.66395600
C	-17.19380900	0.49604200	-6.82020800
H	-16.49749900	-0.26694200	-6.44802100
H	-18.19336500	0.15328800	-6.51193300
C	-15.39987900	2.75211500	-6.70023900
C	-15.47339000	3.93071500	-7.46156300
C	-14.17487000	2.05781200	-6.64768500
C	-14.35080200	4.40784800	-8.14769600
H	-16.41285600	4.47954600	-7.53682400
C	-13.06037900	2.53287000	-7.34359400
H	-14.09714600	1.14336400	-6.05428800
C	-13.14145900	3.71132800	-8.09313800
H	-14.43067100	5.32494500	-8.73752700
H	-12.12130500	1.97409400	-7.29925200
H	-12.26833600	4.08086100	-8.63765000
C	-18.26379400	3.12115500	-6.17833800
C	-19.41310600	2.66154500	-6.84715300
C	-18.25015600	4.45653300	-5.73789400
C	-20.49609700	3.51388700	-7.08149600
H	-19.48778900	1.62942700	-7.18774000
C	-19.33050100	5.30902400	-5.97187800
H	-17.39066400	4.83724600	-5.19116900
C	-20.45965000	4.84081500	-6.64852300
H	-21.37676200	3.12966000	-7.60262700
H	-19.29241900	6.33859100	-5.60794400
H	-21.30949800	5.50399900	-6.82912700
Cl	-15.31564300	-0.43307700	-4.26921800
C	-11.08962600	2.99942100	-3.37078100
H	-10.74234500	3.81201000	-4.02543900
H	-10.57205800	3.05977900	-2.40237300
H	-10.87880200	2.03218700	-3.84154900
P	-17.95415500	-0.72539500	-9.27587800
C	-17.61435300	-0.16595000	-11.01688400
C	-18.60613800	0.60879500	-11.64479500
C	-16.44284800	-0.46429400	-11.73333700
C	-18.42525300	1.09080100	-12.94426700
H	-19.53450400	0.83095600	-11.10973300
C	-16.26589700	0.00811400	-13.03725300
H	-15.66292500	-1.07409000	-11.27141300
C	-17.25381100	0.78916700	-13.64514400
H	-19.20652800	1.69421000	-13.41433200
H	-15.34933000	-0.23562200	-13.58153500
H	-17.11349200	1.15672700	-14.66510400
C	-16.80384900	-2.16602700	-9.09383400
C	-17.29704100	-3.42011800	-9.50174800
C	-15.50942100	-2.10452700	-8.55059800
C	-16.51501400	-4.57152900	-9.39246100
H	-18.31062700	-3.49232200	-9.90764700

C	-14.73071200	-3.26048700	-8.42505400
H	-15.09344600	-1.15307200	-8.21409100
C	-15.22797400	-4.49498100	-8.84940500
H	-16.91590500	-5.53419500	-9.72091600
H	-13.73042800	-3.19195500	-7.98952100
H	-14.61837300	-5.39688700	-8.74998200
C	-18.21521400	-1.80799700	-1.21982500
H	-18.95080600	-1.40777600	-0.51820200
H	-18.22556000	-2.89180300	-1.36775500
C	-17.34130800	-1.04997600	-1.89361100
H	-16.64781300	-1.53641200	-2.58236400
C	-17.13916800	0.44083000	-1.74706100
C	-18.06660100	1.02580100	-0.66768500
H	-19.12841800	0.85151800	-0.87520100
H	-17.82878900	0.53803200	0.29225000
H	-17.91566600	2.10211300	-0.53396700
C	-15.67532100	0.60822900	-1.17192800
H	-14.88408100	0.20442900	-1.81657900
H	-15.44577400	1.63971100	-0.87719100
H	-15.66652800	0.00332800	-0.25105800

UB3LYP-D3/def2-SVP-SMD(THF)//UB3LYP/def2-SVP(gas)
HF= -4404.1179937
UPBEPBE-D3/def2-SVP-SMD(THF)//UB3LYP/def2-SVP(gas)
HF= -4400.7680769

^{3}D''-TS-dimethyl'

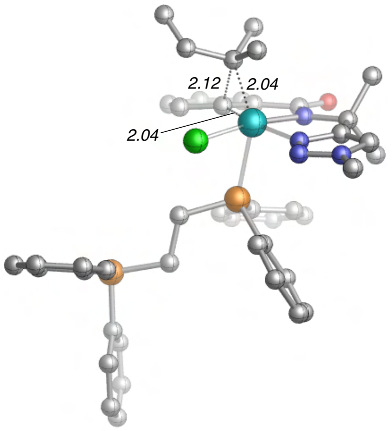

Zero-point correction= 0.810170 (Hartree/Particle)
Thermal correction to Energy= 0.860026
Thermal correction to Enthalpy= 0.860970
Thermal correction to Gibbs Free Energy= 0.723408
Sum of electronic and zero-point Energies= -4403.121654
Sum of electronic and thermal Energies= -4403.071798
Sum of electronic and thermal Enthalpies= -4403.070854
Sum of electronic and thermal Free Energies= -4403.208416

C	-18.39608100	1.13878300	-3.31907300
C	-19.05207200	2.36398700	-3.08372500
C	-20.43382600	2.49096100	-3.25580400
C	-21.17735100	1.40189500	-3.71344500
C	-20.52120100	0.20335300	-4.02303300
C	-19.13974400	0.06962800	-3.83314800
H	-20.89324000	3.45884900	-3.04137000
H	-22.25825900	1.48811400	-3.85064000
H	-21.09025300	-0.64572300	-4.41313900
H	-18.65205400	-0.87566700	-4.07309900
C	-18.21728400	3.53069400	-2.66528500
O	-18.69413600	4.61064100	-2.31382800
C	-15.87488300	4.25074400	-2.34713500
C	-15.97625600	4.58956800	-0.83971400
H	-15.19994400	5.31613100	-0.54966100
H	-16.96259400	5.02746100	-0.63734900
H	-15.85295400	3.69052300	-0.21898700
C	-15.97354800	5.56283100	-3.16493100
H	-16.98116000	5.98408700	-3.06694700
H	-15.24009900	6.29492700	-2.78988500
H	-15.74651500	5.38058600	-4.22489800
C	-14.54416400	3.61115700	-2.65363700
N	-14.51317200	2.37486200	-3.22279100
N	-13.28595900	1.98461300	-3.42936800
C	-13.22870900	4.00853100	-2.51068800
H	-12.76809700	4.90954300	-2.11664400
N	-12.50386800	2.96655200	-3.00809100
N	-16.87988600	3.23363400	-2.73919400
Fe	-16.38116200	1.47109800	-3.31701100

P	-16.81463500	1.98074000	-5.80266700
C	-17.16723200	0.64927000	-8.36154700
H	-17.78104500	1.51022500	-8.67055200
H	-16.15037700	0.85465500	-8.73053800
C	-17.18081100	0.46721800	-6.84042000
H	-16.44146800	-0.27641900	-6.51421700
H	-18.15013800	0.08379800	-6.48489400
C	-15.35564100	2.68350400	-6.71677900
C	-15.40894800	3.80214400	-7.56554600
C	-14.12790400	2.00757500	-6.56931500
C	-14.26470500	4.24049100	-8.24149800
H	-16.34961800	4.33295300	-7.71728800
C	-12.99104900	2.44232900	-7.25605200
H	-14.06551900	1.13486600	-5.91463800
C	-13.05238700	3.56325200	-8.09070600
H	-14.32914100	5.11133000	-8.89941500
H	-12.05063700	1.89642300	-7.13798400
H	-12.16185900	3.90260400	-8.62651100
C	-18.22386600	3.10800500	-6.21937200
C	-19.42064000	2.62513100	-6.78023900
C	-18.15925400	4.47296400	-5.88641600
C	-20.49987900	3.48088500	-7.01690500
H	-19.53457600	1.57026200	-7.02819300
C	-19.23638800	5.32920200	-6.12292700
H	-17.26064100	4.87701600	-5.42464900
C	-20.41272100	4.83642800	-6.69318300
H	-21.41800200	3.07754100	-7.45174700
H	-19.15739800	6.38333700	-5.84613900
H	-21.25924800	5.50332500	-6.87550100
Cl	-15.42599900	-0.47210300	-4.20425900
C	-11.05954000	2.82721200	-3.10270300
H	-10.59199400	3.78261300	-2.83463800
H	-10.70956000	2.04056300	-2.41900600
H	-10.78678600	2.56014100	-4.13230300
P	-17.93802900	-0.80481700	-9.27037400
C	-17.74805100	-0.19654800	-11.01782200
C	-18.82480300	0.52747300	-11.56134100
C	-16.61479300	-0.41446400	-11.81945700
C	-18.76352500	1.03944400	-12.86024900
H	-19.72540300	0.68573600	-10.96004700
C	-16.55762100	0.08718400	-13.12344700
H	-15.76987800	-0.98334300	-11.42477100
C	-17.62869400	0.81809600	-13.64644400
H	-19.60946600	1.60286800	-13.26324300
H	-15.66895600	-0.09415400	-13.73431600
H	-17.58175300	1.20884000	-14.66634700
C	-16.64461800	-2.12750100	-9.16791900
C	-17.04613100	-3.42902700	-9.52357700
C	-15.32200200	-1.93405000	-8.73424800
C	-16.15040700	-4.49892000	-9.46997800
H	-18.07790100	-3.60363700	-9.84322500

C	-14.42764900	-3.00796000	-8.66509800
H	-14.97408700	-0.94098000	-8.44299600
C	-14.83677100	-4.29111200	-9.03658500
H	-16.48216900	-5.50065300	-9.75589100
H	-13.40562500	-2.83776700	-8.31640200
H	-14.13683500	-5.12892900	-8.98107900
C	-18.35169800	-1.67501900	-1.09737500
H	-19.07527600	-1.22317200	-0.41498300
H	-18.39870600	-2.76277400	-1.20356800
C	-17.45037300	-0.97415800	-1.79634700
H	-16.77305500	-1.51524200	-2.46007900
C	-17.18154200	0.51378400	-1.70040800
C	-18.09966500	1.16725700	-0.64993100
H	-19.16595500	1.02588000	-0.85960800
H	-17.88823500	0.70349100	0.32775500
H	-17.91082700	2.24105800	-0.54715900
C	-15.73236600	0.59016500	-1.06202500
H	-14.95307200	0.12975500	-1.68258800
H	-15.44296800	1.61675100	-0.80119200
H	-15.77698300	0.01640700	-0.12176500

UB3LYP-D3/def2-SVP-SMD(THF)//UB3LYP/def2-SVP(gas)
HF= -4404.1431899
UPBEPBE-D3/def2-SVP-SMD(THF)//UB3LYP/def2-SVP(gas)
HF= -4400.7885892

^{5}D''-TS-dimethyl'

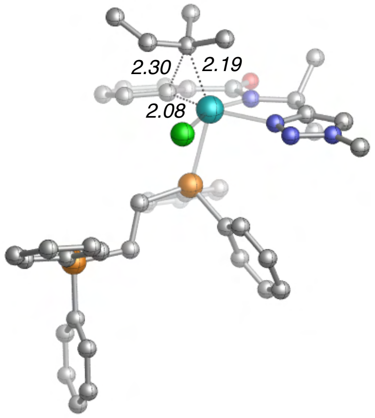

Zero-point correction= 0.808043 (Hartree/Particle)
Thermal correction to Energy= 0.858919
Thermal correction to Enthalpy= 0.859863
Thermal correction to Gibbs Free Energy= 0.717949
Sum of electronic and zero-point Energies= -4403.142518
Sum of electronic and thermal Energies= -4403.091643
Sum of electronic and thermal Enthalpies= -4403.090698
Sum of electronic and thermal Free Energies= -4403.232613

C	-18.50253800	1.25650400	-3.22825700
C	-19.02635800	2.52843700	-2.94468900
C	-20.39402200	2.77141700	-3.11817200
C	-21.23070100	1.76902300	-3.61302700
C	-20.69119400	0.52253100	-3.94961900
C	-19.32803600	0.26039300	-3.75104200
H	-20.76605000	3.76777400	-2.86804300
H	-22.29807300	1.96039600	-3.74988300
H	-21.33347700	-0.26297700	-4.35853400
H	-18.93016700	-0.72818800	-3.98669500
C	-18.11885600	3.64150800	-2.48900800
O	-18.58194800	4.71476300	-2.08883500
C	-15.78645600	4.30365700	-2.21742700
C	-15.86294400	4.63392500	-0.70563000
H	-15.06353300	5.33485500	-0.41417800
H	-16.83579900	5.09286300	-0.48691400
H	-15.75630200	3.72253700	-0.09799900
C	-15.87561900	5.61962600	-3.03142800
H	-16.86974600	6.06435000	-2.90055500
H	-15.11357000	6.33754200	-2.68652900
H	-15.69294800	5.42745700	-4.09907900
C	-14.45665700	3.65327200	-2.52763800
N	-14.40997100	2.41555900	-3.08492900
N	-13.17651800	2.03969100	-3.28416300
C	-13.14518300	4.06841400	-2.37975600
H	-12.69765600	4.97787400	-1.98986900
N	-12.40418900	3.03339500	-2.86488400
N	-16.80293900	3.31449100	-2.60913000
Fe	-16.42489400	1.38564200	-3.30553200

P	-16.89656400	1.99855000	-5.86430900
C	-17.18394400	0.58359000	-8.38735500
H	-17.73282800	1.46217600	-8.76292900
H	-16.13842000	0.72201500	-8.70447500
C	-17.28880800	0.46981500	-6.86313800
H	-16.60585300	-0.29437900	-6.46561600
H	-18.29682300	0.15596900	-6.54680800
C	-15.43753600	2.71105400	-6.76228900
C	-15.52820200	3.78969500	-7.65908800
C	-14.18104200	2.11146700	-6.54901700
C	-14.39108000	4.26023900	-8.32400600
H	-16.49092500	4.26677400	-7.84872100
C	-13.05057300	2.57925700	-7.22474200
H	-14.08945700	1.27440000	-5.85276100
C	-13.14920900	3.65652700	-8.11108400
H	-14.48221700	5.09927800	-9.01896900
H	-12.08507700	2.09489700	-7.05399500
H	-12.26289200	4.02211500	-8.63654800
C	-18.28354700	3.15519900	-6.24867400
C	-19.45933500	2.74338100	-6.90063800
C	-18.20706600	4.48517400	-5.79557000
C	-20.51306200	3.63886000	-7.10845500
H	-19.57419800	1.71695700	-7.24950800
C	-19.25792500	5.37939800	-6.00479600
H	-17.32038100	4.82679400	-5.26259900
C	-20.41635100	4.95934600	-6.66499200
H	-21.41680900	3.29515800	-7.61828600
H	-19.17475400	6.40420000	-5.63501000
H	-21.24264100	5.65658300	-6.82552200
Cl	-15.27476700	-0.46470500	-4.25779400
C	-10.95869600	2.91220400	-2.95623300
H	-10.50123300	3.85776100	-2.63999300
H	-10.60622900	2.09784100	-2.30727600
H	-10.67273200	2.69517500	-3.99439800
P	-17.97947500	-0.86496200	-9.28308800
C	-17.65896300	-0.33947800	-11.03834000
C	-18.70116700	0.32391100	-11.71001100
C	-16.45077700	-0.55697200	-11.72255100
C	-18.53599700	0.77618900	-13.02246400
H	-19.65600200	0.48116500	-11.19916100
C	-16.28821200	-0.11400000	-13.03837500
H	-15.63073100	-1.08068100	-11.22555800
C	-17.32815500	0.55609100	-13.69081600
H	-19.35701100	1.29220100	-13.52754600
H	-15.34260900	-0.29356000	-13.55730300
H	-17.19909800	0.90054300	-14.72028400
C	-16.76457900	-2.24616800	-9.06130000
C	-17.18939800	-3.52665800	-9.46469600
C	-15.48689500	-2.11795700	-8.49082700
C	-16.35681200	-4.63850100	-9.32533500
H	-18.18915700	-3.65125400	-9.89172400

C	-14.65791700	-3.23476700	-8.33484100
H	-15.12222200	-1.14542100	-8.15520000
C	-15.08697300	-4.49563100	-8.75552500
H	-16.70472100	-5.62237700	-9.65135000
H	-13.67226100	-3.11403800	-7.87793100
H	-14.43806800	-5.36667300	-8.63253600
C	-18.44726500	-1.87444600	-1.37927600
H	-19.26928300	-1.47525100	-0.78011200
H	-18.47871700	-2.94505000	-1.60092300
C	-17.43942500	-1.11830700	-1.83878800
H	-16.66043300	-1.59506600	-2.43983900
C	-17.21994400	0.33378100	-1.56227000
C	-18.18650100	0.92838100	-0.54086800
H	-18.01304300	2.00299600	-0.40291500
H	-19.24080200	0.78459300	-0.80405800
H	-18.00990100	0.43514800	0.43173100
C	-15.78024100	0.50275000	-1.00072400
H	-14.99568100	0.10640400	-1.66047800
H	-15.55660500	1.54469300	-0.73733200
H	-15.73699300	-0.08668300	-0.06750000

UB3LYP-D3/def2-SVP-SMD(THF)//UB3LYP/def2-SVP(gas)
HF= -4404.1599822
UPBEPBE-D3/def2-SVP-SMD(THF)//UB3LYP/def2-SVP(gas)
HF= -4400.7868377

1E''-dimethyl'

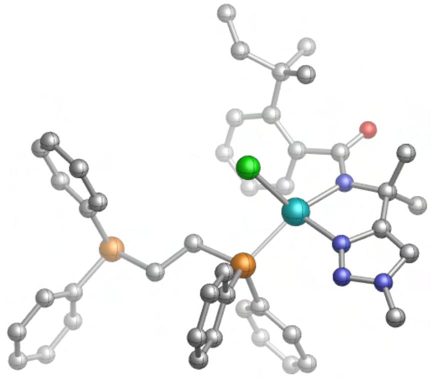

Zero-point correction= 0.810419 (Hartree/Particle)
Thermal correction to Energy= 0.863744
Thermal correction to Enthalpy= 0.864688
Thermal correction to Gibbs Free Energy= 0.713641
Sum of electronic and zero-point Energies= -4403.193356
Sum of electronic and thermal Energies= -4403.140031
Sum of electronic and thermal Enthalpies= -4403.139087
Sum of electronic and thermal Free Energies= -4403.290133

C	-18.56355200	-0.06780300	-2.41802400
C	-18.43327600	1.33146600	-2.61249000
C	-19.11279100	1.95557300	-3.67781600
C	-19.94161600	1.24059200	-4.54434800
C	-20.07727800	-0.13394200	-4.36090900
C	-19.39173900	-0.76215100	-3.31898100
H	-19.01982200	3.03838300	-3.79145400
H	-20.48366300	1.75767200	-5.34055000
H	-20.71566000	-0.72371200	-5.02394600
H	-19.50808000	-1.83979200	-3.19290200
C	-17.71749400	2.28705400	-1.67362700
O	-18.11193100	2.41332600	-0.51025300
C	-16.07232500	4.08089800	-1.49675900
C	-15.30847500	3.53682000	-0.26601900
H	-14.75186500	4.34343300	0.23936900
H	-16.02588000	3.10227300	0.44099800
H	-14.59587200	2.75724800	-0.57472800
C	-17.07998800	5.17443700	-1.06943100
H	-17.82415400	4.73748300	-0.39236500
H	-16.56667000	6.00140700	-0.55142400
H	-17.59690100	5.58340400	-1.95144800
C	-15.07643500	4.69063100	-2.45573400
N	-14.92435900	4.15684300	-3.70123000
N	-14.02728900	4.81031400	-4.39121000
C	-14.19337300	5.75138900	-2.38342100
H	-13.96065400	6.46207500	-1.59566400
N	-13.57777700	5.77514500	-3.59909000
N	-16.72549300	3.00050500	-2.26774100
Fe	-16.00826000	2.52743400	-4.02951000
P	-16.27663300	2.63025300	-6.40504100

C	-17.12448600	0.91473200	-8.58771800
H	-17.56469600	1.79500400	-9.08434100
H	-16.10910800	0.80923200	-9.00101300
C	-17.09039700	1.09650500	-7.06842600
H	-16.54040900	0.28466400	-6.56651500
H	-18.09990200	1.07009600	-6.62642100
C	-14.72401500	2.78631300	-7.39565700
C	-14.66917700	3.47676500	-8.61874500
C	-13.56435100	2.15127000	-6.91638100
C	-13.47721500	3.53379500	-9.34675300
H	-15.55819500	3.97641900	-9.00883900
C	-12.37712800	2.20516300	-7.65258900
H	-13.60041900	1.60425900	-5.97058200
C	-12.32862000	2.89793200	-8.86586100
H	-13.44942300	4.07373100	-10.29693800
H	-11.48401900	1.70378600	-7.27042600
H	-11.39783000	2.94131600	-9.43771900
C	-17.31816500	4.05169800	-6.94672500
C	-18.64679400	3.89689300	-7.37732100
C	-16.80234800	5.35563000	-6.81216400
C	-19.43396800	5.01417100	-7.67797600
H	-19.08312300	2.90190300	-7.48033600
C	-17.58823300	6.46806900	-7.11756800
H	-15.77529900	5.49748300	-6.46594200
C	-18.90787700	6.30146200	-7.55208700
H	-20.46448900	4.87268100	-8.01358100
H	-17.16961300	7.47254000	-7.01258100
H	-19.52357800	7.17287100	-7.78860700
Cl	-15.11813700	0.42365700	-4.15859700
C	-12.55007900	6.68596400	-4.07477200
H	-12.93286400	7.71684200	-4.09970300
H	-11.66813800	6.63869100	-3.41974200
H	-12.27268500	6.36986200	-5.08722500
P	-18.22779100	-0.50986500	-9.12776500
C	-17.99807900	-0.37054400	-10.96862200
C	-18.97423700	0.34639400	-11.68335900
C	-16.92102000	-0.92810400	-11.67862600
C	-18.86801000	0.52091300	-13.06630000
H	-19.83236400	0.76565300	-11.14910400
C	-16.81934800	-0.76282100	-13.06307300
H	-16.15709400	-1.50083300	-11.14753300
C	-17.78961400	-0.03571900	-13.76015400
H	-19.63600900	1.08301100	-13.60451900
H	-15.97651800	-1.20581800	-13.60069100
H	-17.70877700	0.09062200	-14.84300900
C	-17.23015500	-2.01530100	-8.71687300
C	-17.90219800	-3.25040600	-8.78632300
C	-15.88501300	-2.01072100	-8.31044600
C	-17.24591800	-4.44463100	-8.48245000
H	-18.95618500	-3.27301100	-9.07932900
C	-15.23084300	-3.20544600	-7.98973900

H	-15.32955300	-1.07387600	-8.23455900
C	-15.90654600	-4.42473100	-8.07925000
H	-17.78546900	-5.39319600	-8.54785000
H	-14.18746000	-3.17853200	-7.66507500
H	-15.39424600	-5.35671600	-7.82683900
C	-18.08206200	-3.44627900	-1.22795600
H	-18.76389400	-3.48482900	-0.37405900
H	-17.76302700	-4.40681200	-1.64321100
C	-17.64537900	-2.29599700	-1.74929800
H	-16.96195300	-2.33168100	-2.60753300
C	-17.92649500	-0.88065500	-1.25868300
C	-18.91079100	-0.84800900	-0.06883100
H	-19.88185300	-1.29465300	-0.33461300
H	-18.50062400	-1.39981600	0.79267900
H	-19.07822600	0.19547800	0.23269900
C	-16.54672800	-0.34790700	-0.79111600
H	-15.86383900	-0.20037000	-1.63983000
H	-16.64628700	0.59307000	-0.23933300
H	-16.09627400	-1.08611000	-0.10920800

UB3LYP-D3/def2-SVP-SMD(THF)//UB3LYP/def2-SVP(gas)
HF= -4404.1179937
UPBEPBE-D3/def2-SVP-SMD(THF)//UB3LYP/def2-SVP(gas)
HF= -4400.7998702

^{3}E''-dimethyl'

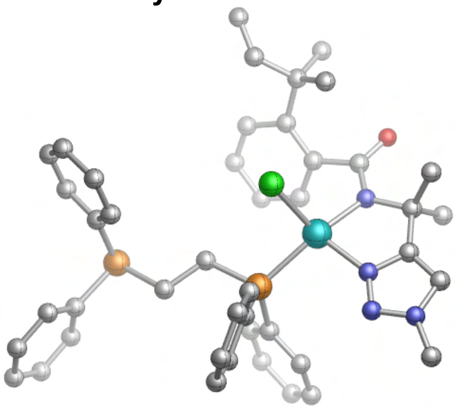

Zero-point correction= 0.810515 (Hartree/Particle)
Thermal correction to Energy= 0.863771
Thermal correction to Enthalpy= 0.864716
Thermal correction to Gibbs Free Energy= 0.712970
Sum of electronic and zero-point Energies= -4403.206653
Sum of electronic and thermal Energies= -4403.153396
Sum of electronic and thermal Enthalpies= -4403.152452
Sum of electronic and thermal Free Energies= -4403.304198

C	-18.63207500	-0.04442000	-2.38973000
C	-18.40046500	1.34771800	-2.53854000
C	-19.06056900	2.05965400	-3.56061300
C	-19.95508500	1.44076600	-4.43615900
C	-20.18492500	0.07368900	-4.30145500
C	-19.52818700	-0.64008800	-3.29648000
H	-18.89935500	3.13790700	-3.63015700
H	-20.47468600	2.02693500	-5.19895100
H	-20.87650800	-0.44414600	-4.97127800
H	-19.72200700	-1.71018700	-3.20727200
C	-17.57951200	2.20802100	-1.59604600
O	-17.87715800	2.26181200	-0.39764300
C	-15.90968000	3.97528200	-1.41891000
C	-15.00229800	3.33789600	-0.34071500
H	-14.40648000	4.10459400	0.18220300
H	-15.63095500	2.81668700	0.39229500
H	-14.31765100	2.61034700	-0.80206800
C	-16.87566500	5.00208600	-0.77756500
H	-17.52698400	4.49211600	-0.05847900
H	-16.31179600	5.79331400	-0.25633000
H	-17.50093200	5.47353800	-1.55171300
C	-15.04891300	4.70887200	-2.41950100
N	-15.04336500	4.31521000	-3.72414400
N	-14.24168100	5.05984300	-4.44062800
C	-14.17819300	5.77925400	-2.33863300
H	-13.86055600	6.40890400	-1.51261500
N	-13.71396700	5.94610500	-3.60958300
N	-16.62029800	2.94853700	-2.21186500
Fe	-16.04174000	2.61197200	-4.05515400
P	-16.20758800	2.64565600	-6.41896100

C	-17.12689800	0.94712700	-8.58136000
H	-17.51518400	1.84344500	-9.09231900
H	-16.11572500	0.78042900	-8.98501900
C	-17.09453800	1.14474000	-7.06419300
H	-16.59268100	0.31279500	-6.54604900
H	-18.10725500	1.17656300	-6.62941000
C	-14.61167000	2.66913300	-7.35016800
C	-14.46301900	3.29719200	-8.59875600
C	-13.51294200	1.99187200	-6.79072700
C	-13.23833700	3.25440000	-9.27138300
H	-15.30382800	3.82595600	-9.05210800
C	-12.29233300	1.94693200	-7.47091800
H	-13.62517100	1.48379600	-5.82895700
C	-12.15020200	2.58005700	-8.70915400
H	-13.13735500	3.74678400	-10.24220500
H	-11.44777900	1.41373400	-7.02655700
H	-11.19374800	2.54615300	-9.23759500
C	-17.15358300	4.09841600	-7.05170500
C	-18.49877100	3.99302300	-7.44685900
C	-16.55866800	5.37463000	-7.02935100
C	-19.22376900	5.12861000	-7.82364200
H	-18.99727900	3.02236300	-7.46234400
C	-17.28347000	6.50545400	-7.41078100
H	-15.52168800	5.48169700	-6.70353500
C	-18.61882900	6.38716100	-7.81053900
H	-20.26767300	5.02405000	-8.13017300
H	-16.80314900	7.48747800	-7.39267200
H	-19.18555700	7.27323500	-8.10745900
Cl	-15.38968400	0.41784600	-4.12669700
C	-12.77492100	6.93995200	-4.10303100
H	-13.22348300	7.94391700	-4.06818700
H	-11.85983400	6.92640800	-3.49436100
H	-12.53053400	6.68038400	-5.13972900
P	-18.30306600	-0.42101300	-9.11343800
C	-18.05564000	-0.31312100	-10.95423000
C	-18.98657700	0.44965700	-11.68176500
C	-17.00736700	-0.93662000	-11.65235100
C	-18.86334000	0.60407200	-13.06567900
H	-19.82289300	0.92131600	-11.15669900
C	-16.88910700	-0.79113900	-13.03772100
H	-16.27941100	-1.54550700	-11.11105200
C	-17.81374000	-0.01842500	-13.74763900
H	-19.59603900	1.20243900	-13.61392600
H	-16.06929400	-1.28562400	-13.56597400
H	-17.72018300	0.09230300	-14.83118500
C	-17.39193300	-1.97482300	-8.68208400
C	-18.13081900	-3.17159800	-8.74249100
C	-16.05046200	-2.04023200	-8.26892600
C	-17.54279700	-4.39702800	-8.42348700
H	-19.18324400	-3.13898200	-9.04010500
C	-15.46456300	-3.26587300	-7.93334800

H	-15.44502800	-1.13440400	-8.19922800
C	-16.20609200	-4.44691800	-8.01412400
H	-18.13365200	-5.31498700	-8.48181800
H	-14.42265300	-3.29341600	-7.60397300
H	-15.74711000	-5.40311500	-7.74995300
C	-18.38342200	-3.50128100	-1.36124800
H	-19.05222000	-3.52999800	-0.49670400
H	-18.14241800	-4.46211600	-1.82560800
C	-17.87224700	-2.36252400	-1.83851700
H	-17.20774700	-2.40797500	-2.71100000
C	-18.03868800	-0.95459900	-1.27849800
C	-19.00325900	-0.90549300	-0.07306200
H	-19.08474000	0.13211400	0.27956900
H	-20.00906000	-1.26395700	-0.34340300
H	-18.62550000	-1.52722300	0.75503000
C	-16.61641200	-0.55526300	-0.80349000
H	-15.93777300	-0.39972200	-1.65390800
H	-16.64075400	0.35340300	-0.19280700
H	-16.21263000	-1.36839400	-0.17997600

UB3LYP-D3/def2-SVP-SMD(THF)//UB3LYP/def2-SVP(gas)
HF= -4404.2163776
UPBEPBE-D3/def2-SVP-SMD(THF)//UB3LYP/def2-SVP(gas)
HF= -4400.8304162

^{5}E''-dimethyl'

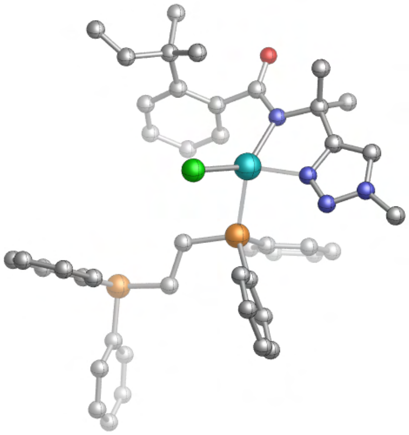

Zero-point correction= 0.809964 (Hartree/Particle)
Thermal correction to Energy= 0.863548
Thermal correction to Enthalpy= 0.864492
Thermal correction to Gibbs Free Energy= 0.710854
Sum of electronic and zero-point Energies= -4403.239239
Sum of electronic and thermal Energies= -4403.185655
Sum of electronic and thermal Enthalpies= -4403.184711
Sum of electronic and thermal Free Energies= -4403.338349

C	-18.72370300	0.12834000	-2.28860800
C	-18.53699200	1.50025300	-2.60230800
C	-19.14757800	2.04267100	-3.75066600
C	-19.96585200	1.27629300	-4.58239000
C	-20.15939900	-0.07013200	-4.27928700
C	-19.53857700	-0.62150100	-3.15659800
H	-19.00561800	3.10542500	-3.96300300
H	-20.45570900	1.73353500	-5.44589600
H	-20.79330700	-0.69720600	-4.91169200
H	-19.69943600	-1.67806000	-2.93637000
C	-17.83452700	2.52317700	-1.71897000
O	-18.32916700	2.81983100	-0.62647900
C	-16.04272400	4.17338700	-1.59713700
C	-15.51600500	3.74987700	-0.20318300
H	-14.93893000	4.56659700	0.26078500
H	-16.36356600	3.49620300	0.44496600
H	-14.86099800	2.87027300	-0.29555000
C	-16.95762700	5.41374800	-1.47083700
H	-17.83164000	5.15661500	-0.85881400
H	-16.42584500	6.25372300	-0.99387700
H	-17.30109100	5.73801300	-2.46540800
C	-14.84571500	4.52971000	-2.45777600
N	-14.51793200	3.79329100	-3.55595800
N	-13.44660100	4.26320800	-4.13426000
C	-13.89097100	5.52675700	-2.36182800
H	-13.74192900	6.33722100	-1.65422900
N	-13.05740300	5.31198300	-3.41698600
N	-16.73542500	3.07013500	-2.29217800
Fe	-15.90147400	2.17224400	-3.89396100

P	-16.31344800	2.48478500	-6.36658400
C	-17.11771500	0.81494000	-8.60849700
H	-17.53521200	1.71093000	-9.09621100
H	-16.09455900	0.70662900	-9.00107400
C	-17.11417400	0.96574100	-7.08509400
H	-16.57612300	0.13798500	-6.59551800
H	-18.13261800	0.93234800	-6.66426200
C	-14.77557900	2.69493800	-7.36936000
C	-14.72770100	3.46481600	-8.54431300
C	-13.61659700	2.01983600	-6.94611600
C	-13.54407200	3.55670300	-9.28212900
H	-15.61622200	3.99849200	-8.88804100
C	-12.43851900	2.10699400	-7.69353700
H	-13.63962200	1.41935600	-6.03272400
C	-12.39768800	2.87649900	-8.85990100
H	-13.52096900	4.15854600	-10.19448900
H	-11.54621200	1.57404900	-7.35500500
H	-11.47374500	2.94693200	-9.44006700
C	-17.36935600	3.91776700	-6.83674100
C	-18.66613100	3.78379400	-7.36009000
C	-16.88819100	5.21134500	-6.55412000
C	-19.45600800	4.91319100	-7.60443700
H	-19.07545000	2.79655700	-7.58087100
C	-17.67522500	6.33634000	-6.80440300
H	-15.88563800	5.33650400	-6.13591600
C	-18.96391100	6.19079800	-7.33042700
H	-20.46218100	4.78869300	-8.01274700
H	-17.28333700	7.33236400	-6.58243600
H	-19.58232700	7.07117600	-7.52208500
Cl	-14.96367600	0.11022600	-4.05759900
C	-11.86508700	6.04943200	-3.79727900
H	-11.09201100	5.95758200	-3.02004700
H	-11.49662800	5.61573200	-4.73445800
H	-12.10531400	7.11159700	-3.95008900
P	-18.22607000	-0.58558100	-9.19979000
C	-17.94212800	-0.42760600	-11.03118400
C	-18.87896100	0.32618300	-11.76066400
C	-16.86317600	-1.00625200	-11.72099900
C	-18.73159600	0.51639000	-13.13765900
H	-19.73907900	0.76207900	-11.24315400
C	-16.72083200	-0.82531400	-13.09994200
H	-16.12972000	-1.60766100	-11.17888800
C	-17.65146600	-0.06135200	-13.81133400
H	-19.46911400	1.10736200	-13.68742400
H	-15.87712600	-1.28503800	-13.62187500
H	-17.53873100	0.07732700	-14.88980100
C	-17.26324700	-2.10957000	-8.77429200
C	-17.96086500	-3.33047400	-8.83972700
C	-15.91992200	-2.13096800	-8.36186100
C	-17.33134300	-4.53644100	-8.52507100
H	-19.01367900	-3.33233600	-9.13763800

C	-15.29265700	-3.33695300	-8.03031500
H	-15.34492100	-1.20544300	-8.29091700
C	-15.99395200	-4.54218700	-8.11529900
H	-17.89043100	-5.47382700	-8.58722700
H	-14.25045500	-3.33028500	-7.70090000
H	-15.50274800	-5.48312500	-7.85436800
C	-18.46132100	-3.11480100	-0.71019600
H	-19.16790900	-3.01881800	0.11855900
H	-18.18456700	-4.13210900	-1.00213200
C	-17.94711100	-2.05697400	-1.34416600
H	-17.24284000	-2.22876500	-2.16865900
C	-18.16350900	-0.58194400	-1.02834800
C	-19.17016300	-0.36129800	0.12212800
H	-19.29156400	0.71693500	0.29681800
H	-20.15642600	-0.78778800	-0.11925700
H	-18.80926300	-0.82977600	1.05225400
C	-16.76837600	-0.06523500	-0.59182900
H	-16.05468800	-0.07892700	-1.42829200
H	-16.82446500	0.94819200	-0.17940500
H	-16.37814300	-0.72240900	0.20086800

UB3LYP-D3/def2-SVP-SMD(THF)//UB3LYP/def2-SVP(gas)
HF= -4404.2487177
UPBEPBE-D3/def2-SVP-SMD(THF)//UB3LYP/def2-SVP(gas)
HF= -4400.8477071

4.4 Isomer Shift Calculations for 1b, 2b, 3b, and 4b

Calculations of Mössbauer isomer shifts were performed with ORCA package[15], using optimized structures. Calculations were performed at B3LYP[8] level of theory with core polarized CP(PPP)[16] basis set for iron atom and TZVP[17] basis set for all other atoms. Isomer shifts were calculated from electron densities at the iron nucleus using a previously reported procedure.[18] Table S2 shows comparison of experimental and calculated isomer shifts. The observed deviations in isomer shift values between experiment and theory are within the errors observed in calculating isomer shifts for organoiron species.[19]

Table S2. Comparison of experimental and calculated isomer shifts.

Complex	Experimental isomer shift (mm/s)[a]	Calculated isomer shift (mm/s)
1b	0.94	0.84[b]/0.82[c]
2b	0.33	0.43
3b	0.27	0.23
4b	0.16	0.27

[a] 80 K frozen solution parameters
[b] L = Br
[c] L = THF

4. References

1. T. Matsubara, S. Asako, L. Ilies and E. Nakamura, *J. Am. Chem. Soc.*, 2014, **136**, 646-649.
2. Q. Gu, H. H. Al Mamari, K. Graczyk, E. Diers and L. Ackermann, *Angew. Chem. Int. Ed.*, 2014, **53**, 3868-3871.
3. G. Cera, T. Haven and L. Ackermann, *Angew. Chem. Int. Ed.*, 2016, **55**, 1484-1488.
4. M. G. T. C. Ribeiro and A. A. S. C. Machado, *J. Chem. Ed.*, 2011, **88**, 947-953.
5. G. Winter, D. W. Thompson and J. R. Loehe, in *Inorganic Syntheses*, eds. A. Wold and J.K. Ruff, John Wiley & Sons, Inc., 1973, ch. 20, pp. 99-104.
6. T. E. Boddie, S. H. Carpenter, T. M. Baker, J. C. DeMuth, G. Cera, W. W. Brennessel, L. Ackermann and M. L. Neidig, *J. Am. Chem. Soc.*, 2019, **141**, 12338-12345.
7. R. B. Bedford, P. B. Brenner, E. Carter, J. Clifton, P. M. Cogswell, N. J. Gower, M. F. Haddow, J. N. Harvey, J. A. Kehl, D. M. Murphy, E. C. Neeve, M. L. Neidig, J. Nunn, B. E. R. Snyder and J. Taylor, *Organometallics*, 2014, **33**, 5767-5780.
8. (a) C. Lee, W. Yang and R. G. Parr, *Phys. Rev. B.* 1988, **37**, 785–789. (b) A. D. Becke, *J. Chem. Phys.* 1993, *98*, 5648–5652.
9. (a) F. Weigend, R. Ahlrichs, *Phys. Chem. Chem. Phys.* 2005, **7**, 3297-3305. (b) F. Weigend, *Phys. Chem. Chem. Phys.* 2006, **8**, 1057-1065.
10. Gaussian 09, Revision A.02, M. J. Frisch, G. W. Trucks, H. B. Schlegel, G. E. Scuseria, M. A. Robb, J. R. Cheeseman, G. Scalmani, V. Barone, G. A. Petersson, H. Nakatsuji, X. Li, M. Caricato, A. Marenich, J. Bloino, B. G. Janesko, R. Gomperts, B. Mennucci, H. P. Hratchian, J. V. Ortiz, A. F. Izmaylov, J. L. Sonnenberg, D. Williams-Young, F. Ding, F. Lipparini, F. Egidi, J. Goings, B. Peng, A. Petrone, T. Henderson, D. Ranasinghe, V. G. Zakrzewski, J. Gao, N. Rega, G. Zheng, W. Liang, M. Hada, M. Ehara, K. Toyota, R. Fukuda, J. Hasegawa, M. Ishida, T. Nakajima, Y. Honda, O. Kitao, H. Nakai, T. Vreven, K. Throssell, J. A. Montgomery, Jr., J. E. Peralta, F. Ogliaro, M. Bearpark, J. J. Heyd, E. Brothers, K. N. Kudin, V. N. Staroverov, T. Keith, R. Kobayashi, J. Normand, K. Raghavachari, A. Rendell, J. C. Burant, S. S. Iyengar, J. Tomasi, M. Cossi, J. M. Millam, M. Klene, C. Adamo, R. Cammi, J. W. Ochterski, R. L. Martin, K. Morokuma, O. Farkas, J. B. Foresman, and D. J. Fox, Gaussian, Inc., Wallingford CT, 2016.
11. Gaussian 16, Revision C.01, M. J. Frisch, G. W. Trucks, H. B. Schlegel, G. E. Scuseria, M. A. Robb, J. R. Cheeseman, G. Scalmani, V. Barone, G. A. Petersson, H. Nakatsuji, X. Li, M. Caricato, A. V. Marenich, J. Bloino, B. G. Janesko, R. Gomperts, B. Mennucci, H. P. Hratchian, J. V. Ortiz, A. F. Izmaylov, J. L. Sonnenberg, D. Williams-Young, F. Ding, F. Lipparini, F. Egidi, J. Goings, B. Peng, A. Petrone, T. Henderson, D. Ranasinghe, V. G. Zakrzewski, J. Gao, N. Rega, G. Zheng, W. Liang, M. Hada, M. Ehara, K. Toyota, R. Fukuda, J. Hasegawa, M. Ishida, T. Nakajima, Y. Honda, O. Kitao, H. Nakai, T. Vreven, K. Throssell, J. A. Montgomery, Jr., J. E. Peralta, F. Ogliaro, M. J. Bearpark, J. J. Heyd, E. N. Brothers, K. N. Kudin, V. N. Staroverov, T. A. Keith, R. Kobayashi, J. Normand, K. Raghavachari, A. P. Rendell, J. C. Burant, S. S. Iyengar, J. Tomasi, M. Cossi, J. M. Millam, M. Klene, C. Adamo, R. Cammi, J. W. Ochterski, R. L. Martin, K. Morokuma, O. Farkas, J. B. Foresman, and D. J. Fox, Gaussian, Inc., Wallingford CT, 2016.
12. (a) J. P. Perdew, K. Burke and M. Ernzerhof, *Phys. Rev. Lett.* 1996, **77**, 3865-3868. (b) J. P. Perdew, K. Burke and M. Ernzerhof, *Phys. Rev. Lett.* 1997, **78**, 1396-1396.
13. (a) A. Klamt, G. Schüürmann, *J. Chem. Soc. Perkin Trans.,* 1993, **2**, 799-805. (b) J. Tomasi, M. Persico, *Chem. Rev.*, 1994, **94**, 2027-2094. (c) J. Andzelm, C. Kölmel and A. Klamt, *J. Chem. Phys.,* 1995*,* **103**, 9312-9320. (d) V. Barone, M. Cossi, *J. Phys. Chem. A.,* 1992*,* **102**,

1995-2001. (e) M. Cossi, N. Rega, G. Scalmani and V. Barone, *J. Comput. Chem.* 2003, **24**, 669-681.
14. CYLview, 1.0b; C. Y. Legault, Université de Sherbrooke, 2009 (http://www.cylview.org).
15. Neese, F. *WIREs Comput. Mol. Sci.* 2012, **2**, 73-78
16. (a) Neese, F. *Inorg. Chim. Acta* 2002, **337**, 181. (b) Sinnecker, S.; Slep, L. D.; Bill, E.; Neese, F. *Inorg, Chem.* 2005, **44**, 2245.
17. Schäfer, A.; Huber, C.; Ahlrichs, R. *J. Chem. Phys.* 1994, **100**, 5829.
18. Römelt, M.; Ye, S.; Neese, F. *Inorg. Chem.* 2009, **48**, 784-785.
19. McWilliams, S. F.; Brennan-Wydra, E.; Macleod, K. C.; Holland, P. L., *ACS Omega*, 2017, 2, 2594-2606.
